# Transcriptome, Phenotypic, and Virulence Analysis of *Streptococcus sanguinis* SK36 Wild Type and Its CcpA-Null Derivative (ΔCcpA)

**DOI:** 10.3389/fcimb.2019.00411

**Published:** 2019-12-04

**Authors:** Yibo Bai, Mengmeng Shang, Mengya Xu, Anyi Wu, Luning Sun, Lanyan Zheng

**Affiliations:** ^1^Department of Pathogen Biology, College of Basic Medical Sciences, China Medical University, Shenyang, China; ^2^Department of Scientific Research, Peking Union Medical College Hospital (East), Beijing, China; ^3^Department of Pathophysiology, College of Basic Medical Science, China Medical University, Shenyang, China

**Keywords:** CcpA, RNA-seq, DEGs, EPS, biofilm, *Streptococcus sanguinis*

## Abstract

Catabolic control protein (CcpA) is linked to complex carbohydrate utilization and virulence factor in many bacteria species, influences the transcription of target genes by many mechanisms. To characterize the activity and regulatory mechanisms of CcpA in *Streptococcus sanguinis*, here, we analyzed the transcriptome of *Streptococcus sanguinis* SK36 and its CcpA-null derivative (ΔCcpA) using RNA-seq. Compared to the regulon of CcpA in SK36 in the RegPrecise database, we found that only minority of differentially expressed genes (DEGs) contained putative catabolite response element (cre) in their regulatory regions, indicating that many genes could have been affected indirectly by the loss of CcpA and analyzing the sequence of the promoter region using prediction tools is not a desirable method to recognize potential target genes of global regulator CcpA. Gene ontology and pathway analysis of DEGs revealed that CcpA exerts an influence predominantly involved in carbon catabolite metabolism and some amino acid catabolite pathways, which has been linked to expression of virulence genes in many pathogens and coordinately regulate the disease progression *in vivo* studies. However, in some scenarios, differences observed at the transcript level could not reflect the real differences at the protein level. Therefore, to confirm the differences in phenotype and virulence of SK36 and ΔCcpA, we characterized the role of CcpA in the regulation of biofilm development, EPS production and the virulence of *Streptococcus sanguinis*. Results showed CcpA inactivation impaired biofilm and EPS formation, and CcpA also involved in virulence in rabbit infective endocarditis model. These findings will undoubtedly contribute to investigate the mechanistic links between the global regulator CcpA and the virulence of *Streptococcus sanguinis*, further broaden our understanding of the relationship between basic metabolic processes and virulence.

## Introduction

Infective endocarditis (IE) caused by microorganisms has long been regarded as dependent upon biofilms (Costerton et al., 1999; Douglas, 2003; Parsek and Singh, 2003; Xu et al., 2003). *Streptococcus sanguinis* was recognized as one of the most common causes of endocarditis, alongside *Staphylococcus aureus, Enterococcus* spp., and *Streptococcus bovis* (Vogkou et al., 2016; Zhu et al., 2018). It was well-known that biofilm formation is tightly interconnected with EPS production and the degree of exposure of *S. sanguinis* surface adhesion molecules for the initial colonization (Flemming and Wingender, 2010). Although various factors related to the virulence of *S. sanguinis* have been identified, the regulatory mechanisms of colonization and biofilm development are still elusive.

Catabolic control protein (CcpA) is linked to complex carbohydrate utilization and virulence factor production in many bacteria species in response to changes in overall energy levels and amount of carbohydrate as the global regulator of carbon catabolite repression (CCR) (Abranches et al., 2008; Willenborg et al., 2014). For example, CcpA has been reported to influence the expression of diverse virulence factors of *S. aureus, Streptococcus mutans*, and *Enterococcus faecium* in response to various kinds and concentrations of carbohydrate (Somarajan et al., 2014; Bischoff et al., 2017; Bauer et al., 2018). In *Streptococcus suis*, CcpA was found to be indispensable for capsule production in glucose-affluent conditions and virulence-associated gene expression (Willenborg et al., 2011). In *Streptococcus pneumoniae*, capsule expression was also regulated by RegM/CcpA (Giammarinaro and Paton, 2002). Earlier studies have shown that glucan-producing *S. sanguinis* was found to be more difficultly cleaned from the circulation than glucan-negative mutants (Parker and Ball, 1976; Ramirez-Ronda, 1978). Pioneering work by Bin Zhu and his coworkers reported that insoluble glucan is the major component of *S. sanguinis* biofilms (Zhu et al., 2017). Skov Sorensen and his coworkers demonstrated that these polysaccharides are similar to *S. pneumonia* capsular polysaccharide (CPS) in genetic and antigenic aspect, even they are the equivalent of capsular polysaccharides of *pneumococci* (Skov Sorensen et al., 2016). Considering that CcpA is widely conserved, and the overall CPS structural similarity that exists between viridans group suggests a common biosynthetic pathway for these molecules (Xu et al., 2003; Yang et al., 2009; Skov Sorensen et al., 2016), which prompted us to characterize the role CcpA plays in the biofilm development, EPS production and virulence of *S. sanguinis* and further to identify potential targets of global regulator CcpA regulated that contribute to cause IE.

It is well-known that CcpA exerts its regulatory role by binding to a typical consensus site called catabolite response element (cre) in the promotor regions (Weickert and Chambliss, 1990). Recently, a novel mode of regulation of the *S. aureus* CcpA mediated by Stk1 protein phosphorylation was found (Leiba et al., 2012), and the study by Chen et al. (2019) reported that the pilin genes cluster of *S. sanguinis* SK36 occurred in a CcpA-dependent manner, although a typical cre is absent in the target region (SSA_2318). Furthermore, some researchers have reported the distinct regulatory role of CcpA in *S. sanguinis*, may involve in the key metabolic pathways through specific metabolic product (Redanz et al., 2018). All these suggested that there may be other unknown regulation model exists. Consequently, analyzing the sequence of the promoter region using the prediction tools is not a desirable method to recognize the CcpA regulated target genes.

In this study, we analyzed the whole transcriptome of wild-type *S. sanguinis* SK36 and its CcpA-null derivative (ΔCcpA) using high throughput sequencing technologies (RNA-seq). We not only revealed the potential target genes of CcpA in *S. sanguinis* and showed that some amino acid catabolic pathways are regulated by CcpA in *S. sanguinis*, we also characterize the role of CcpA in the regulation of EPS production, biofilm formation and the virulence of *S. sanguinis*. We found that CcpA inactivation impaired EPS production and biofilm formation *in vitro*, and CcpA also involved in virulence in a rabbit IE model. These findings will undoubtedly contribute to investigate the mechanistic links between the global regulator CcpA and the virulence of *S. sanguinis*, further broaden our understanding of the relationship between basic metabolic processes and virulence.

## Materials and Methods

### Bacterial Strains and Culture Conditions

*S. sanguinis* strains were obtained from Zheng et al. (2011), including SK36, the CcpA mutant (ΔCcpA), and the complement of the ΔCcpA (CcpA+). We grew strains as static cultures at 37°C in brain heart infusion (BHI; Difco, Sparks, MD) or on BHI agar plates in an anaerobic chamber (90% N_2_, 5% CO_2_, 5% H_2_). All the strains grew well and reached the same plateau though CcpA mutant showed a decreased growth. So, we collected the cells at the mid-exponential phase (A 600 = 0.5).When required for selection, antibiotics were added to the medium as follows: spectinomycin at 500 μg/ml and erythromycin at 2 μg/ml for *S. sanguinis*.

### RNA Isolation and Sequencing

RNA was isolated using the KANGWEI Ultrapure RNA Kit (CWBIO, China) and the isolated RNA was subjected to DNase I (Promega, Beijing, China) treatment and purified with the TIANGEN RNAclean kit (TIANGEN, China). The clean RNA samples were aliquoted into two tubes and frozed at −80°C for further RT-PCR or RNA-Seq processing. The Ribo-Zero TM Magnetic Kit (Gram-Positive Bacteria) was used to remove ribosomal RNA (rRNA) and enrich the mRNA to compensate for the low-input samples. The average RNA Integrity number (RIN) was 8, and the average RNA yield was 100 ng/μl. The library preparation, sequencing, and initial quality check were performed by Berry Genomics Corporation, Genomics and Bioinformatics Service, China (http://www.berrygenomics.com/tech-services/illumina). Samples were then sequenced using the Illumina Hiseq^TM^ 2500 Next Generation Sequencer at Berry Genomics Corporation, China. Three independent experiments of each group were performed and sequenced. The resulting sequences were then aligned to the reference genome of strain SK36 (GenBank Accession: NC_009009) in order to create a transcriptome map using EDGE-pro. Gene quantification was calculated by the reads per kilobase per million mapped reads method (RPKM), using RSEM (V1.2.15) software.

### Differentially Expressed Genes (DEGs) Analysis

Differences in gene expression profiles were performed using EdgeR statistics including a Benjamini and Hochberg false discovery rate correction. Each group had three biological replicates. False Discovery Rate (FDR) < 0.05 and |log2RPKM ratios (ΔCcpA/WT)|>1 were taken as the thresholds to ascertain the significance of differences in gene expression. To further investigate the DEGs, these DEGs were clustered and presented as heat maps.

### Quantitative Real-Time PCR (qRT-PCR)

SSA-1575, SSA-2379, SSA-0016, and SSA-0391, randomly selected DEGs, were performed qRT-PCR to validate the results of RNA-seq. cDNA was synthesized from 2 μg of RNA using the SuperScript II reverse transcriptase (Invitrogen) followed the manufacturer's recommendation. The primer sequences are listed in Table 1. The gyrA gene was used as reference gene for calculation of the relative target gene expression using the 2^−ΔΔCt^ method. All qPCR results are presented as ratios of the ΔCcpA/wild type levels relative to gyrA transcripts. The experiments were repeated three times independently, and three replicates were involved in each sample. Data were analyzed with GraphPad Prism (version 5.0) software using Student's *t*-test (*P*-value below 0.05 was considered statistical significance).

**Table 1:** List of the primer sequences used in qPCR analysis.

**Gene**	**Forward primer (5^**′**^-3^**′**^)**	**Reverse primer (5^**′**^-3^**′**^)**
gyrA	GCCGTGAGCGAATTGTCGTAAC	CGAACAGCAGTGATACCGTCAATG
Com C	TGAAAATCTATTCTTTTCAAATTGC	CAATCCCATGGATTTGGAAT
Com D	GCGTTTGCG TCAAAAAGAAT	ACAACTTGATTGGAAGGCGTTC
Com X	CAAGAAAGCCAAAAGCGAAA	TCGCTTCTCTGAAGGCAACT
SpxB	AATTCGGCGGCTCAATCG	AAGGATAGCAAGGAATGGAGTG

### Gene Ontology (GO) and Pathway Analysis

To characterize the GO terms, including molecular functions, biological processes, cellular components, and functional pathways of DEGs, significantly enriched GO terms was analyzed by a hypergeometric test, based on “GO Term Finder” (Boyle et al., 2004; Lang et al., 2015). All the DEGs were mapped to the terms in KO (KEGG Orthology) identifier (Kanehisa et al., 2012; Zeng et al., 2013) using KOBAS 2.0 to identify which pathways the significant DEGs belonged to.

### Biofilm Development and Quantification

Biofilm formation was measured as reported previously (Zheng et al., 2012), however, some procedures were modified. Briefly, SK36, ΔCcpA, and CcpA+ overnight grown cultures were diluted 1:60 in fresh BHI media supplemented with 0.2% sucrose and inoculated into microtiter plates(96-well cell culture plates; Thermo Fisher scientific), then anaerobically grown as static cultures for 48 h at 37°C to form biofilms. After removing the residual medium and air-drying the microtiter dish, the residual cells were resuspended in the well with 90% ethanol and then transferred to a new-flat bottomed microtiter dish with 100 mL of ethanol where it was measured at 570 nm using a microplate reader. Data were analyzed with GraphPad Prism (version 5.0) software. Statistical significance was indicated when the *P*-value was below 0.05.

### Detection of EPS-Producing Phenotype of the Strains by the Congo Red Agar (CRA) Plate Test

CRA was prepared by adding 0.8 g of Congo red and 50 g of sucrose to 1 L of brain heart infusion agar as described previously (Mathur et al., 2006; Zheng et al., 2012). SK36, ΔCcpA, and CcpA+ overnight cultures grown in BHI medium were streaked on CRA plates and anaerobically cultured for 21 h. Then, plates were scanned with an HP scanner or photographed with a digital camera. The interaction of the direct dye Congo red with intact β-D-glucans (one molecule of Congo red is bound to six D-glucose residues of the D-glucan chain) provides the basis for a rapid and sensitive assay system for detection of EPS production of bacteria (Ogawa and Hatano, 1978).

### Rabbit Model of IE

In this study, to study the role of global regulator CcpA in the virulence of *S. sanguinis*, we used the rabbit endocarditis model. The experiment was performed using a modification of transaortic catheterization models of endocarditis as previously described (Fan et al., 2012) and was reviewed and approved by the IACUC of the China Medical University Laboratory Animal Center. Adult Rex rabbits (2–3 kg; obtained from China Medical University Laboratory Animal Center) were used. Twenty-four hours after the positioning of the catheter, a total of 44 rabbits were assigned randomly to the following treatment groups: A, SK36 (*n* = 12); B, ΔCcpA (*n* = 12); C, CcpA+(*n* = 12); D, control (*n* = 8). Viable *S. sanguinis* (~10^9^ CFU) or PBS was injected intravenously via the marginal ear vein. SK36, ΔCcpA, or CcpA+ strains were collected at cell densities (*A*_600_ = 0.63) and then washed and resuspended with PBS to the desired cell density as inoculated organisms. Twenty-four hours after injection, 200 μl of arterial blood was extracted from the ear of each animal to be quantitatively cultured in duplicate on BHI plates to determine the number of bacteria present in the blood. Three days after injection, the animals were euthanized. At autopsy, the proper positioning of the catheter was verified. Vegetations were excised, weighed, and homogenized in PBS before being quantitatively cultured in duplicate on BHI plates after serial dilutions in BHI broth. After 24 h incubation at 37°C, the colonies growing on BHI plates were expressed as log_10_ CFU. Bacterial load from vegetations was expressed by its mean value standard error (SE), and comparisons between groups were performed using one-way analysis of variance (ANOVA). The correlation between CFU and vegetation mass were assessed using Prism (GraphPad Software, La Jolla, CA).

## Results

### Reads Obtaining and Differentially Expressed Genes (DEGs) Analysis

**Figure 1:**
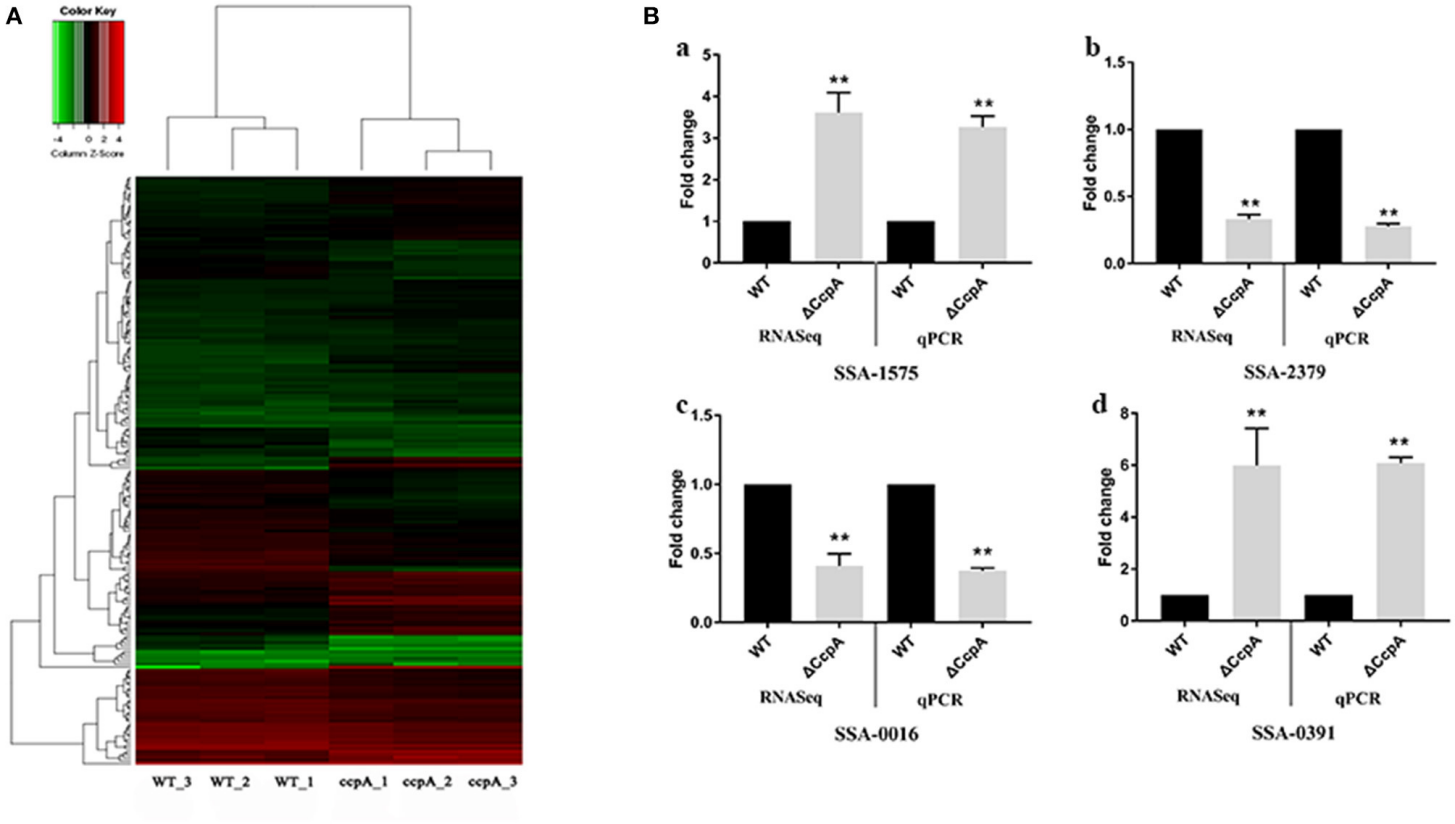
Analysis and validation of DEGs obtained by the RNA-Seq experiments. **(A)** Hierarchical clustering analysis and heatmap of the 195 DEGs with the smallest q-values. Three biological replicates of each group were analyzed separately. **(B)** qRT-PCR validation of selected DEGs, with expression level in ΔCcpA mutant normalized to the SK36 wild type. The transcript levels of (a) SSAA-1575, (b) SSA-2379, (c) SSA-0016, and (d) SSA-0391 of ΔCcpA mutant were detected by qRT-PCR. The data presented are averages and standard deviations of three independent experiments with similar results, triplicate in each experiment. **Indicates the significant difference at *P* < 0.01 compared to the SK36.

In this report, we characterized the transcriptional profile of ΔCcpA mutant by analyzing the RNA-Seq data. After performing quality control, we obtained an average of over 20 million clean reads from each group (*n* = 3). The first group (ΔCcpA) was composed of 7.4, 6.8, and 7.0 million reads and the second group (SK36 WT) contained 7.1, 6.9, and 7.5 million reads. Overall, 76–82% of reads were mapped to the SK36 genome by using EDGE-pro. The saturation curves showed that the sequencing became saturated and the gene coverage indicated adequate sequencing depth (Figures S1–S3 in Supplementary File 1). Data were deposited with the Sequence Read Archive (SRA) at the National Center for Biotechnology Institute (accession PRJNA564466). Differentially expressed genes (DEGs) analysis using EdgeR statistics revealed that the deletion of CcpA in *S. sanguinis* significantly down-regulated 85 unigenes expression and up-regulated 84 unigenes expression of identified 897 unigenes (Supplementary File 2) compared to wild type SK36 (Table 2), according to the standard of a significant difference in expression levels. When compared the differentially expressed genes (DEGs) to the regulon of CcpA in *S. sanguinis* SK36 in the RegPrecise database (http://regprecise.lbl.gov, a web resource for collection, visualization and analysis of transcriptional regulons reconstructed by comparative genomics, Supplementary File 3), as a result, 29 of the 84 up-regulated DEGs, and 5 of the 85 down-regulation of DEGs were in the list of the regulon of CcpA (Table 2). To further investigate the nature of the DEGs, we performed Hierarchical clustering analysis and heatmap of the 173 DEGs with the smallest q-values using a Pearson correlation distance metric. The triplicates were analyzed in each group (Figure 1A).

**Table 2:** The DEGs of ΔCcpA mutant.

**Gene**	**Locus tag**	**FPKM** **(ctrl)**	**FPKM** **(case)**	**log2Fold** **Change**	**FDR**
**(1) Up-regulated DEGs**					
c185_g93	SSA_0778	32.64	2,011.40	5.79	0.00000
c43_g1	SSA_2020^a^	7.32	460.67	6.02	0.00000
c185_g109^b^^,^^m^	SSA_0779^a^	132.91	1,892.65	3.49	0.00000
c185_g52^b^^,^^m^	SSA_0776^a^	17.59	1,037.19	5.70	0.00000
c184_g184^b^^,^^c^^,^^m^	SSA_1615^a^	270.81	4,128.01	4.03	0.00000
c157_g1^b^^,^^c^^,^^m^	SSA_1588	21.48	142.36	2.52	0.00000
c184_g183	SSA_0393	79.26	764.76	2.85	0.00000
c246_g1^b^^,^^c^^,^^m^	SSA_1298^a^	487.48	4,096.15	2.99	0.00000
c184_g141^b^^,^^c^^,^^m^	SSA_1749^a^	448.60	2,713.78	2.43	0.00000
c185_g218^b^^,^^m^	SSA_0631	60.92	367.74	2.31	0.00000
c71_g1	SSA_1063	18.64	121.58	2.29	0.00000
c184_g82^b^^,^^c^^,^^m^	SSA_1918^a^	1,391.90	7,543.70	2.24	0.00000
c185_g62^b^^,^^c^^,^^m^	SSA_1259^a^	158.49	817.17	2.09	0.00000
c184_g294^b^^,^^c^^,^^m^	SSA_0391^a^	2,312.95	14,153.22	2.34	0.00000
c184_g18^b^^,^^c^^,^^m^	SSA_0300	67.70	371.66	2.21	0.00000
c183_g84^b^^,^^c^	SSA_1949^a^	132.20	609.00	2.11	0.00000
c183_g30^m^	SSA_0509	52.34	358.25	2.40	0.00000
c184_g19^b^^,^^m^	SSA_0572^a^	72.24	442.42	2.49	0.00000
c185_g150^b^^,^^m^	SSA_1261^a^	70.60	393.52	2.42	0.00000
c449_g1^b^^,^^m^	SSA_2078	69.32	291.42	1.77	0.00000
c185_g56	SSA_2352	20.18	246.58	3.06	0.00000
c185_g235^b^^,^^m^	SSA_1035	57.53	273.58	1.95	0.00000
c184_g71^b^^,^^m^	SSA_0077^a^	21.92	133.45	2.25	0.00000
c183_g25	SSA_0508	69.03	302.99	1.91	0.00000
c185_g234	SSA_1252	22.55	132.42	2.21	0.00000
c185_g254^b^^,^^c^^,^^m^	SSA_0192^a^	480.54	1,520.49	1.46	0.00000
c534_g1^b^^,^^m^	SSA_2083	1.19	10.18	2.52	0.00000
c185_g1^b^^,^^c^^,^^m^	SSA_1256	62.52	312.53	2.23	0.00000
c183_g13	SSA_1207	164.69	548.76	1.37	0.00000
c185_g28	SSA_1251	17.41	105.04	2.32	0.00000
c183_g77^b^^,^^c^^,^^m^	SSA_0342	2,740.47	9,026.47	1.69	0.00000
c184_g292^b^^,^^c^	SSA_0076^a^	29.15	148.31	1.92	0.00000
c184_g132^c^	SSA_0075^a^	25.22	154.85	2.10	0.00000
c185_g3^b^^,^^c^^,^^m^	SSA_1034	90.45	261.42	1.38	0.00000
c241_g1^b^^,^^c^^,^^m^	SSA_0068^a^	94.25	281.24	1.21	0.00001
c186_g1^m^	SSA_1012	330.19	900.52	1.16	0.00001
c386_g1^b^^,^^c^^,^^m^	SSA_1009^a^	51.41	151.27	1.28	0.00001
c154_g1^b^^,^^c^^,^^m^	SSA_0453^a^	98.37	273.16	1.17	0.00001
c184_g259	SSA_1750	29.82	99.50	1.59	0.00001
c184_g324^c^	SSA_1098^a^	196.99	699.14	1.69	0.00002
c184_g290^b^^,^^c^^,^^m^	SSA_0836	85.66	253.80	1.21	0.00002
c185_g121^b^^,^^m^	SSA_0637	43.18	200.86	1.63	0.00003
c184_g170^b^^,^^c^^,^^m^	SSA_0072^a^	32.86	179.23	1.71	0.00003
c185_g32^b^^,^^c^^,^^m^	SSA_1574	138.44	376.16	1.15	0.00004
c276_g1^b^^,^^c^^,^^m^	SSA_2084	1.56	9.42	2.72	0.00006
c184_g238	SSA_0607	53.38	181.86	1.75	0.00007
c184_g130	SSA_0394	629.76	1552.53	1.12	0.00009
c184_g185^b^^,^^c^	SSA_0833	61.53	173.14	1.20	0.00010
c184_g156^b^^,^^c^^,^^m^	SSA_1809^a^	504.45	1,583.03	1.28	0.00022
c185_g153^b^^,^^c^^,^^m^	SSA_1039	375.93	842.09	1.07	0.00023
c183_g35^b^^,^^m^	SSA_0512	101.31	296.07	1.05	0.00027
c184_g9^b^^,^^m^	SSA_2017	31.59	126.87	1.53	0.00028
c184_g102^m^	SSA_0831	44.88	122.92	1.20	0.00033
c184_g310^b^^,^^c^^,^^m^	SSA_0091	81.21	261.34	1.27	0.00035
c184_g3^b^^,^^c^^,^^m^	SSA_0834	51.54	146.79	1.14	0.00037
c185_g148^c^^,^^m^	SSA_2066	64.52	174.51	1.14	0.00037
c60_g1^m^	SSA_2106	3.00	15.43	2.04	0.00046
c184_g125^b^^,^^c^^,^^m^	SSA_0127	73.10	191.76	1.13	0.00054
c184_g248^b^^,^^c^^,^^m^	SSA_1752^a^	828.77	2,044.10	1.05	0.00073
c184_g281^b^^,^^c^^,^^m^	SSA_1913	11.03	75.75	2.43	0.00112
c192_g1^b^^,^^c^^,^^m^	SSA_0297	31.96	98.24	1.60	0.00124
c183_g53^b^^,^^c^^,^^m^	SSA_2175	16.52	52.95	1.31	0.00138
c185_g127^b^^,^^m^	SSA_0638	37.89	135.65	1.34	0.00144
c185_g242^b^^,^^m^	SSA_1575	150.63	397.53	1.27	0.00177
c183_g96^b^^,^^c^^,^^m^	SSA_0980	21.52	85.59	1.29	0.00293
c184_g57^m^	SSA_1053	28.13	84.64	1.40	0.00344
c184_g139	SSA_1746	473.11	1,073.63	1.00	0.00351
c184_g63^b^^,^^c^^,^^m^	SSA_1917^a^	27.34	82.31	1.08	0.00365
c185_g187^b^^,^^c^^,^^m^	SSA_0644	5,202.87	13,437.05	1.40	0.00384
c184_g89^b^^,^^m^	SSA_0090	88.75	240.77	1.01	0.00393
c183_g47^b^^,^^c^^,^^m^	SSA_2167	44.53	112.60	1.01	0.00551
c183_g108^c^^,^^m^	SSA_0318	22.74	75.52	1.03	0.00557
c134_g2^b^^,^^c^^,^^m^	SSA_1003^a^	1.83	5.63	1.10	0.00564
c314_g1^b^^,^^m^	SSA_2096	3.00	16.95	2.59	0.00568
c183_g45^b^^,^^c^^,^^m^	SSA_0502	32.48	78.56	1.02	0.00650
c185_g230	SSA_2060	33.36	101.01	1.27	0.00765
c183_g90^b^^,^^c^^,^^m^	SSA_0500	36.50	107.52	1.10	0.00768
c184_g5^b^^,^^c^^,^^m^	SSA_2230	43.68	123.43	1.03	0.00780
c184_g168	SSA_2018	25.70	75.28	1.21	0.00962
c185_g136	SSA_1614	69.83	152.95	1.00	0.01933
c183_g41	SSA_2177	14.39	37.66	1.34	0.02387
c185_g161	SSA_0667	10.25	27.86	1.33	0.03003
c525_g1^b^^,^^c^^,^^m^	SSA_1008^a^	31.73	70.29	1.17	0.03449
c185_g144^b^	SSA_1306	47.73	125.80	1.02	0.04701
**(2) Down-regulated DEGs**					
c185_g162^b^	SSA_1576^a^	2,355.32	72.38	−5.27	0.00000
c182_g16	SSA_1889	1,449.35	52.64	−4.97	0.00000
c183_G109^b^^,^^c^	SSA_1950	2,214.11	206.75	−3.36	0.00000
c185_G146^b^^,^^c^	SSA_0650^a^	1,238.80	394.95	−1.96	0.00000
c184_g210	SSA_0094	1,330.37	327.41	−2.23	0.00000
c184_G204^b^^,^^c^	SSA_0760	74.75	6.52	−3.50	0.00000
c184_g309	SSA_1052	1,412.90	243.16	−2.55	0.00000
c184_G315^b^^,^^c^^,^^m^	SSA_0757	44.03	4.06	−3.35	0.00000
c184_G202^b^^,^^c^^,^^m^	SSA_0758	56.88	5.36	−3.40	0.00000
c185_g54^c^	SSA_1398	1,209.44	383.14	−2.00	0.00000
c28_g1^c^	SSA_2094	11,083.50	2,829.00	−2.22	0.00000
c184_g303^c^	SSA_0684	2,379.11	731.09	−1.82	0.00000
c183_g63^b^^,^^c^	SSA_2151	914.26	320.17	−1.63	0.00000
c185_g76^b^^,^^c^	SSA_0886^a^	21,601.58	4,635.68	−2.24	0.00000
c242_g1^b^^,^^c^^,^^m^	SSA_1567	495.17	111.10	−2.08	0.00000
c184_g60^b^^,^^c^	SSA_0687	1,880.60	618.04	−1.67	0.00000
c183_g121	SSA_0918	1,791.45	708.64	−1.61	0.00000
c307_g1	SSA_2379	375.43	153.25	−1.68	0.00000
c185_g39	SSA_2364	2,107.33	1,058.92	−1.35	0.00000
c185_g185	SSA_2371	215.79	93.91	−1.52	0.00001
c184_g230^b^^,^^c^	SSA_0759	35.82	2.48	−3.58	0.00001
c184_g222	SSA_2242	522.98	169.18	−1.70	0.00001
c185_g110^b^^,^^c^	SSA_0860	2,286.06	878.21	−1.48	0.00001
c185_g14	SSA_0848^a^	3,980.18	1,824.31	−1.29	0.00002
c185_g95	SSA_0195	93.96	32.03	−2.09	0.00002
c185_g65^b^^,^^c^^,^^m^	SSA_0753	3,079.98	1,410.53	−1.36	0.00002
c185_g189^b^^,^^c^	SSA_1265	20,414.68	8,368.19	−1.37	0.00003
c185_g259	SSA_0193	167.85	60.49	−1.76	0.00004
c185_g238	SSA_0682	3,591.24	1,442.97	−1.41	0.00005
c185_g225	SSA_0170	322.87	127.58	−1.39	0.00005
c185_g78^b^^,^^c^	SSA_1066	2,400.31	795.49	−1.54	0.00005
c184_g80^b^^,^^c^	SSA_2141	159.49	35.60	−2.04	0.00006
c184_g2^b^^,^^c^^,^^m^	SSA_1349	397.55	162.35	−1.43	0.00007
c183_g117	SSA_0822	2,617.79	1,302.75	−1.19	0.00008
c182_g14	SSA_1888	111.54	44.66	−1.36	0.00008
c184_g325^b^^,^^c^	SSA_2318	222.14	140.91	−1.05	0.00009
c184_g167	SSA_2204	379.26	146.64	−1.43	0.00013
c184_g171	SSA_2327	725.33	183.46	−1.77	0.00015
c185_g226^c^	SSA_1536	362.28	108.70	−1.67	0.00016
c184_g295^b^^,^^c^	SSA_1943	486.44	219.83	−1.28	0.00016
c184_g192^b^	SSA_1792	2,361.01	1,048.15	−1.30	0.00023
c368_g1^b^	SSA_1686	698.70	346.62	−1.19	0.00034
c183_g102	SSA_1971	163.74	43.06	−1.77	0.00039
c185_g11^b^^,^^c^^,^^m^	SSA_1538	501.04	212.97	−1.29	0.00045
c185_g232^c^	SSA_1319	449.03	197.37	−1.25	0.00054
c185_g249^b^^,^^c^	SSA_2347	1,513.35	707.94	−1.17	0.00057
c451_g1	SSA_2206	49.12	23.81	−1.43	0.00058
c185_g116^m^	SSA_0447	837.36	429.13	−1.09	0.00073
c184_g217^b^^,^^c^	SSA_0586	571.73	297.29	−1.07	0.00073
c239_g1	SSA_2184	105.81	52.60	−1.22	0.00084
c185_g204^b^	SSA_1996	3,713.76	1,794.79	−1.12	0.00096
c143_g1^c^	SSA_0829	468.71	243.16	−1.17	0.00099
c185_g126^b^	SSA_0441	749.31	344.66	−1.25	0.00099
c185_g99^b^^,^^c^^,^^m^	SSA_1998	6,630.45	3,358.26	−1.18	0.00115
c185_g158^b^^,^^c^	SSA_2035	8,721.98	3,613.72	−1.39	0.00122
c185_g119	SSA_2345	1,569.92	800.80	−1.27	0.00123
c183_g133	SSA_0818	79.50	48.38	−1.08	0.00136
c184_g265^c^	SSA_2188	422.17	187.22	−1.20	0.00138
c184_g124	SSA_1645	205.11	110.75	−1.06	0.00144
c185_g13^b^^,^^m^	SSA_0851	315.14	121.60	−1.38	0.00149
c183_g68^b^	SSA_0314	123.09	66.54	−1.06	0.00156
c185_g248	SSA_0169	686.51	304.62	−1.35	0.00165
c184_g269	SSA_0844	511.20	217.67	−1.23	0.00187
c183_g105^b^^,^^c^	SSA_1223	6,575.54	2,568.83	−1.44	0.00189
c185_g132	SSA_0885^a^	302.27	159.87	−1.04	0.00198
c185_g214^b^	SSA_1992	6,628.39	2,941.07	−1.27	0.00261
c185_g31	SSA_0227	162.37	80.65	−1.07	0.00280
c184_g311	SSA_0701	1,885.59	1,157.84	−1.17	0.00304
c184_g332^c^	SSA_2249	83.46	50.01	−1.12	0.00304
c184_g58^b^	SSA_2205	3,704.78	1,616.27	−1.28	0.00339
c184_g126^b^^,^^c^	SSA_1060	9,769.74	4,519.35	−1.29	0.00381
c377_g1^b^^,^^c^^,^^m^	SSA_0716	176.63	98.16	−1.06	0.00384
c308_g1^c^	SSA_0617	427.06	209.58	−1.06	0.00402
c204_g1	SSA_0016	34.59	19.85	−1.51	0.00570
c184_g117^b^	SSA_2210	122.64	61.28	−1.05	0.00747
c185_g72^c^	SSA_0167	148.20	77.07	−1.03	0.00815
c185_g137^b^	SSA_2061	1,259.10	646.43	−1.06	0.00859
c185_g212^b^^,^^c^	SSA_1520	63,068.97	28,654.30	−1.14	0.01051
c197_g1	SSA_1671	48.54	30.95	−1.02	0.01211
c185_g202	SSA_2067	367.77	186.16	−1.03	0.01220
c185_g63^b^^,^^c^	SSA_1310	7,265.45	3,285.79	−1.11	0.01265
c184_g232	SSA_0723	193.60	102.67	−1.15	0.01310
c185_g257^b^	SSA_2374	169.52	84.74	−1.01	0.01422
c184_g26^b^^,^^c^^,^^m^	SSA_2133	145.08	92.99	−1.01	0.01598
c184_g30^b^^,^^c^	SSA_2142	36.71	10.44	−1.55	0.03134

a *Genes associated with putative cre-sites*.

b *GO classification:biological process(BP)*.

c *GO classification: cellular component(CC)*.

m *GO classification: molecular function(MF)*.

### Confirmation of RNA-Seq Results by qRT-PCR

To validate the DEGs observed by the RNA-Seq experiments, we randomly selected four DEGs (SSA-1575, SSA-2379, SSA-0016, and SSA-0391) to examine the transcript levels by qRT-PCR. The results of qRT-PCR matched those of the RNA-seq: the expression of SSA-1575, SSA-2379, SSA-0016, and SSA-0391 in ΔCcpA mutant were 3.27-, 0.28-, 0.37-, 6.08-fold compared to wild type SK36, respectively (Figure 1B). The consistent results revealed that the DEGs obtained by RNA-Seq data is reliable and efficient.

### Gene Ontology (GO) and Pathway Analysis of DEGs

**Figure 2:**
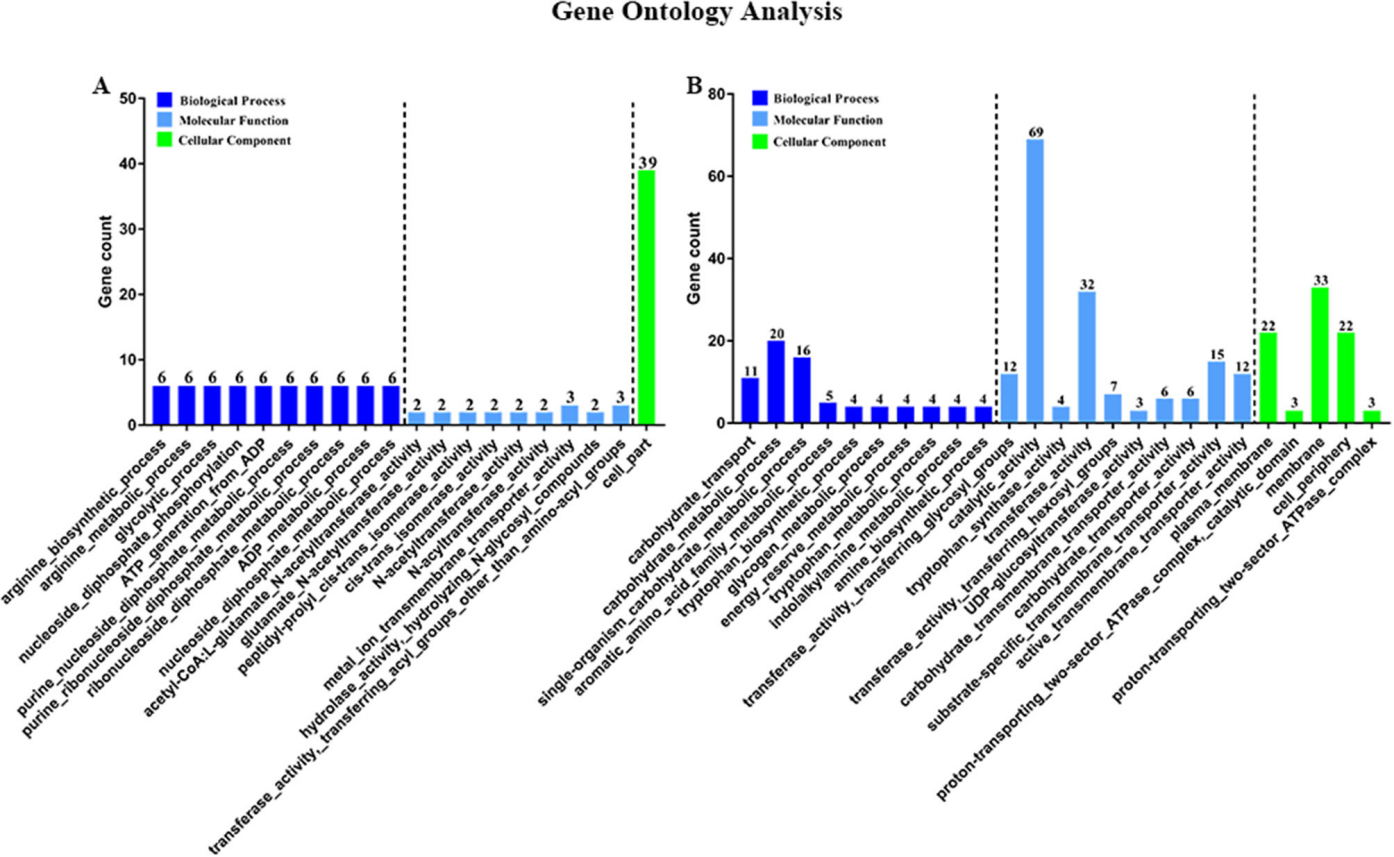
Gene Ontology (GO) classification of DEGs. Genes were annotated in three categories: biological process, molecular function, and cellular component. Y-axis represents the gene counts of a specific category of DEGs within that main category. X-axis represents the top 10 significant (*p* < 0.05) enrichment pathway Term (If less than 10,it represents all enrichment pathway Terms). **(A,B)** Represent CcpA case vs. WT ctrl down-regulated genes GO Term and CcpA case vs. WT ctrl up-regulated genes GO Term, respectively.

**Figure 3:**
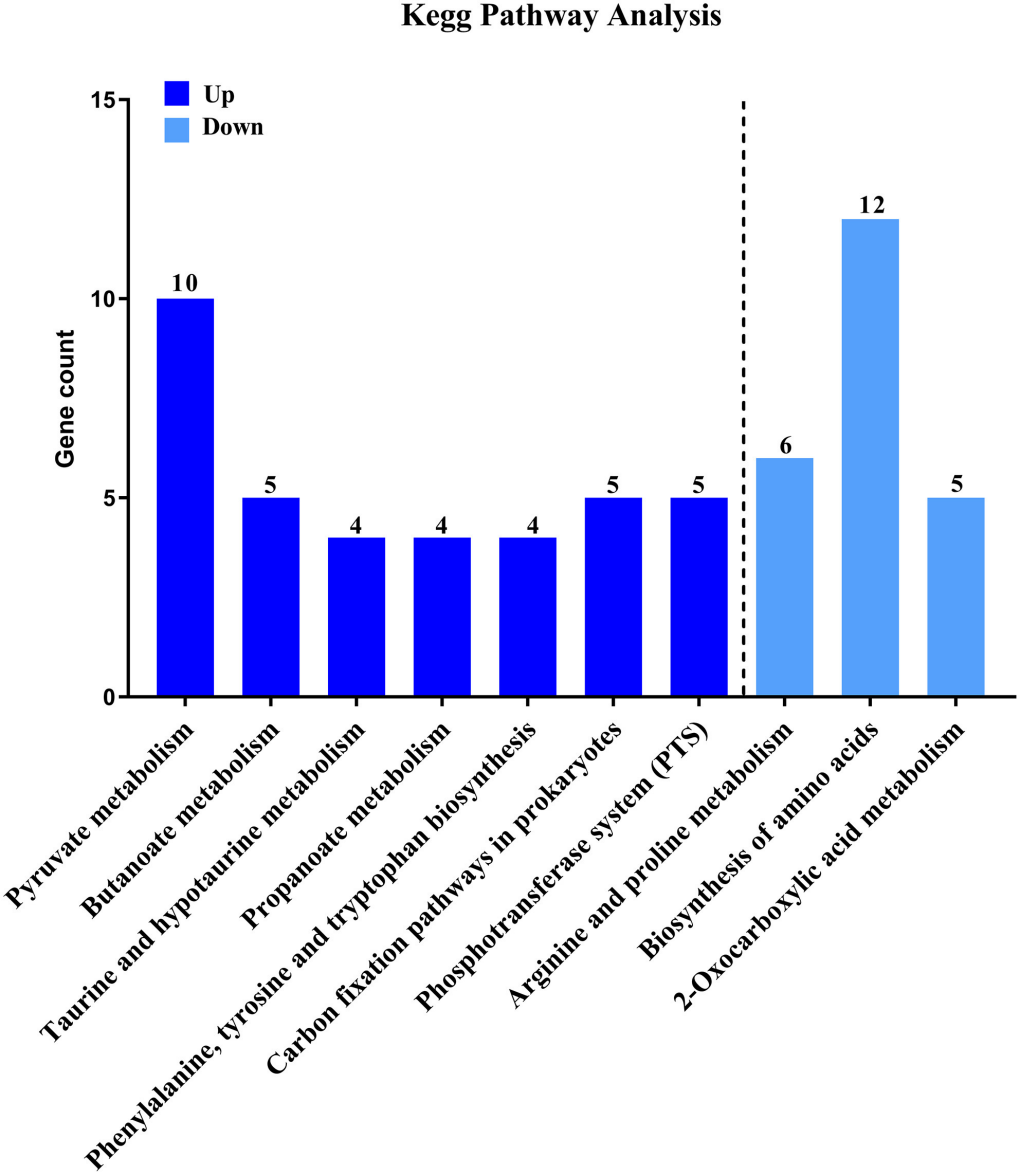
KEGG pathway analysis of DEGs. Here represents CcpA case vs. WT ctrl significantly (*P* < 0.05) up-regulated genes pathway enrichment and CcpA case vs. WT ctrl significantly (*P* < 0.05) down-regulated genes pathway enrichment, respectively.

Then, to characterize DEGs in functional groups and identify pathways that were significantly regulated by *S. sanguinis* CcpA, we performed GO terms and pathway analyses. Of the 85-down regulated DEGs, 56 DEGs could be assigned to a significant GO classification. Of the 84-up regulated DEGs, 74 DEGs could be assigned to a significant GO classification (Figure 2, Supplementary File 4). These results suggested that DEGs predominately involved in basic metabolic processes. Then KEGG pathway enrichment analysis was performed to identify pathways that regulated by CcpA, among the 85 down-regulated and 84 up-regulated DEGs, 27 and 48 DEGs were mapped to 24 and 33 KEGG pathways, respectively (Supplementary File 5). Seven pathways namely-“Pyruvate metabolism,” “Butanoate metabolism,” “Taurine and hypotaurine metabolism,” “Propanoate metabolism,” “Phenylalanine, tyrosine, and tryptophan biosynthesis,” “Carbon fixation pathways in prokaryotes,” and “Phosphotransferase system (PTS)” were the most significant (*P* < 0.05) pathway represented by the up-regulated DEGs, while the down-regulated DEGs predominantly involved in the “Arginine and proline metabolism,” “Biosynthesis of amino acids,” and “2-Oxocarboxylic acid metabolism” pathway (Figure 3).

### ΔCcpA Mutant Shows Decreased Formation of Biofilm

**Figure 4:**
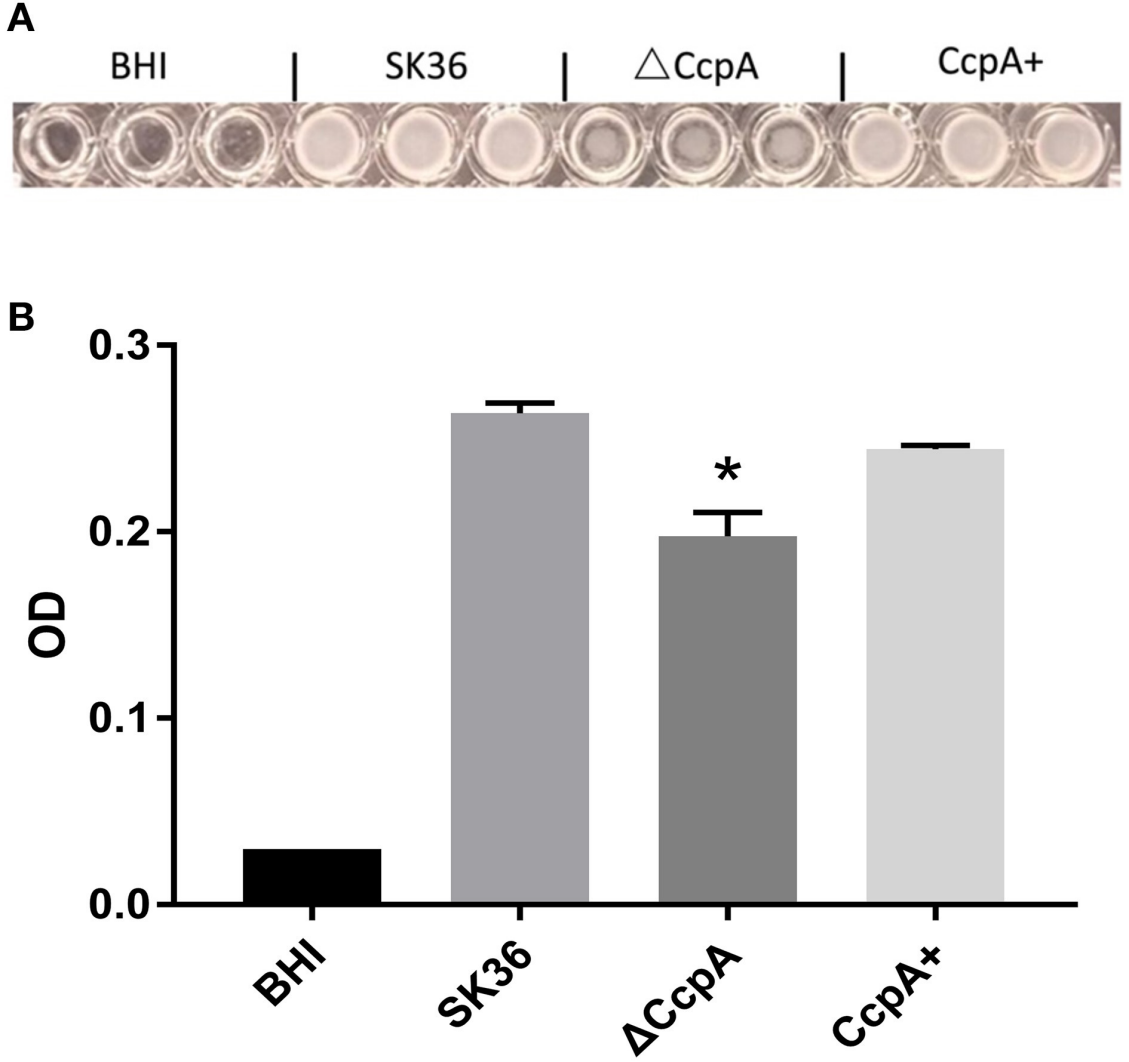
The effect of CcpA on the formation of biofilm. SK36, ΔCcpA, and CcpA+ strains cultures were inoculated into microtiter plates and anaerobically grown as static cultures for 48 h at 37°C to form biofilms. **(A)** Representative of microtiter plate wells from each experiment showing the respective biofilm formation of each *S. sanguinis* strain. **(B)** Quantitative analysis of biofilm production measuring at 570 nm using a microplate reader. The data presented are averages and standard deviations of three independent experiments with similar results, triplicate in each experiment. *Indicates the significant difference at *P* < 0.05 compared to the SK36.

Regarding of the importance of biofilm formation in infective endocarditis (Moser et al., 2017), we further explored the potential role of CcpA in the formation of biofilm. We observed biofilm development *in vitro* by a microtiter plate assay. As expected, quantitative analysis of biofilm production of SK36, ΔCcpA, and CcpA+ grown on the microtiter plate surface indicated that ΔCcpA showed weakened biofilm-forming ability compared to SK36 and CcpA+ (Figure 4).

### ΔCcpA Shows Impaired EPS Production

**Figure 5:**
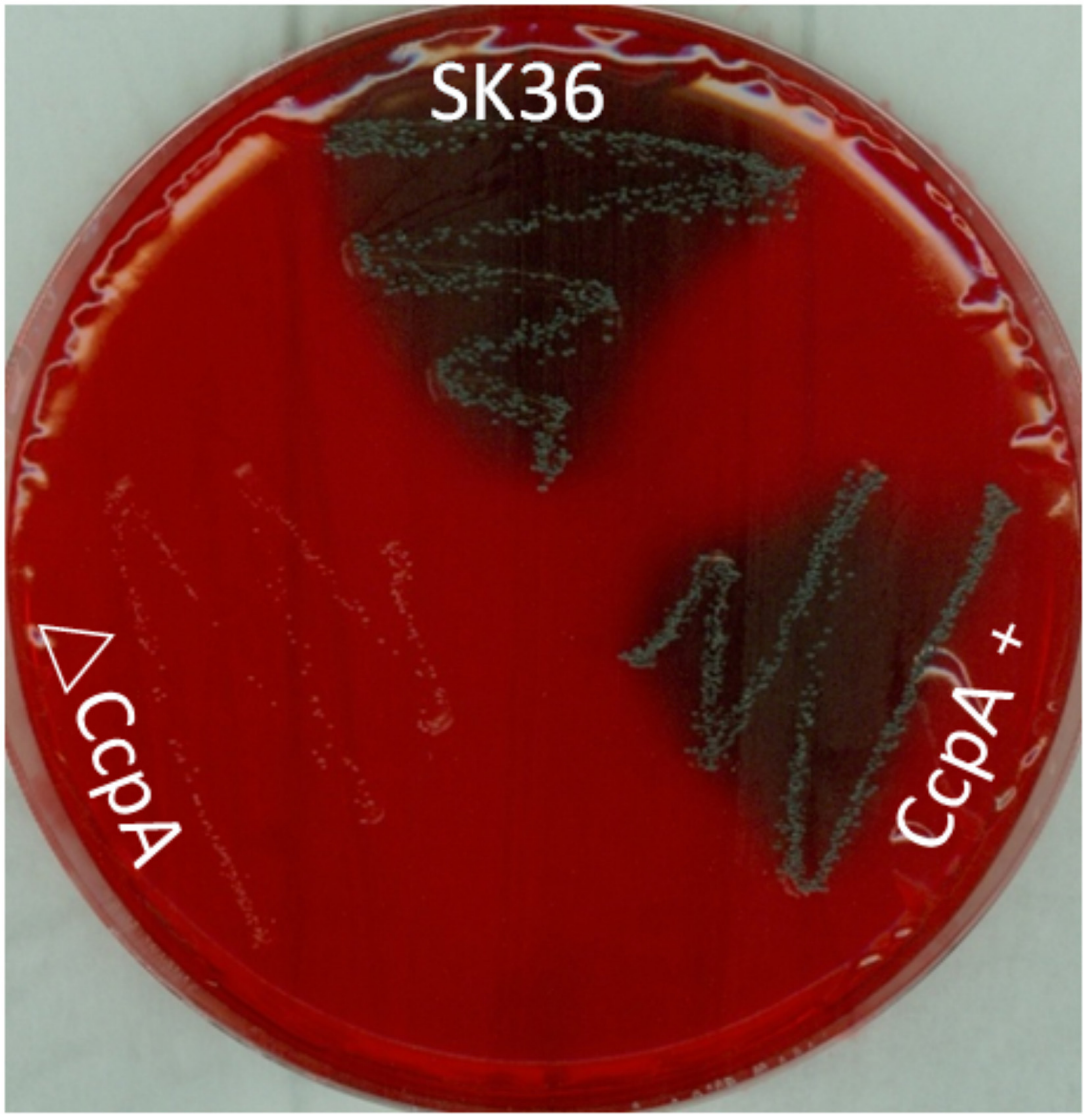
Detection of EPS production by CRA plate test. SK36, ΔCcpA, and CcpA+strains were inoculated on the CRA plates. Through the different color of colonies formed on the solid medium to recognize the EPS-producing strains (characterized by black colonies on the red agar) and non-EPS-producing strains (pink/red colored colonies).

To investigate the role that CcpA plays in EPS production in *S. sanguinis*, the Congo red agar (CRA) plate test was performed. The direct analysis of the colonies formed on CRA plate allows the recognition of EPS-producing strains (Teather and Wood, 1982; Freeman et al., 1989). Results showed that there was an obvious distinction in the appearance of ΔCcpA compared to SK36. ΔCcpA produced pink colonies, whereas SK36 and CcpA + formed black colonies, suggesting that CcpA is required for EPS production in *S. sanguinis* (Figure 5).

### CcpA Contributes to the Virulence of *S. sanguinis* in IE

**Figure 6:**
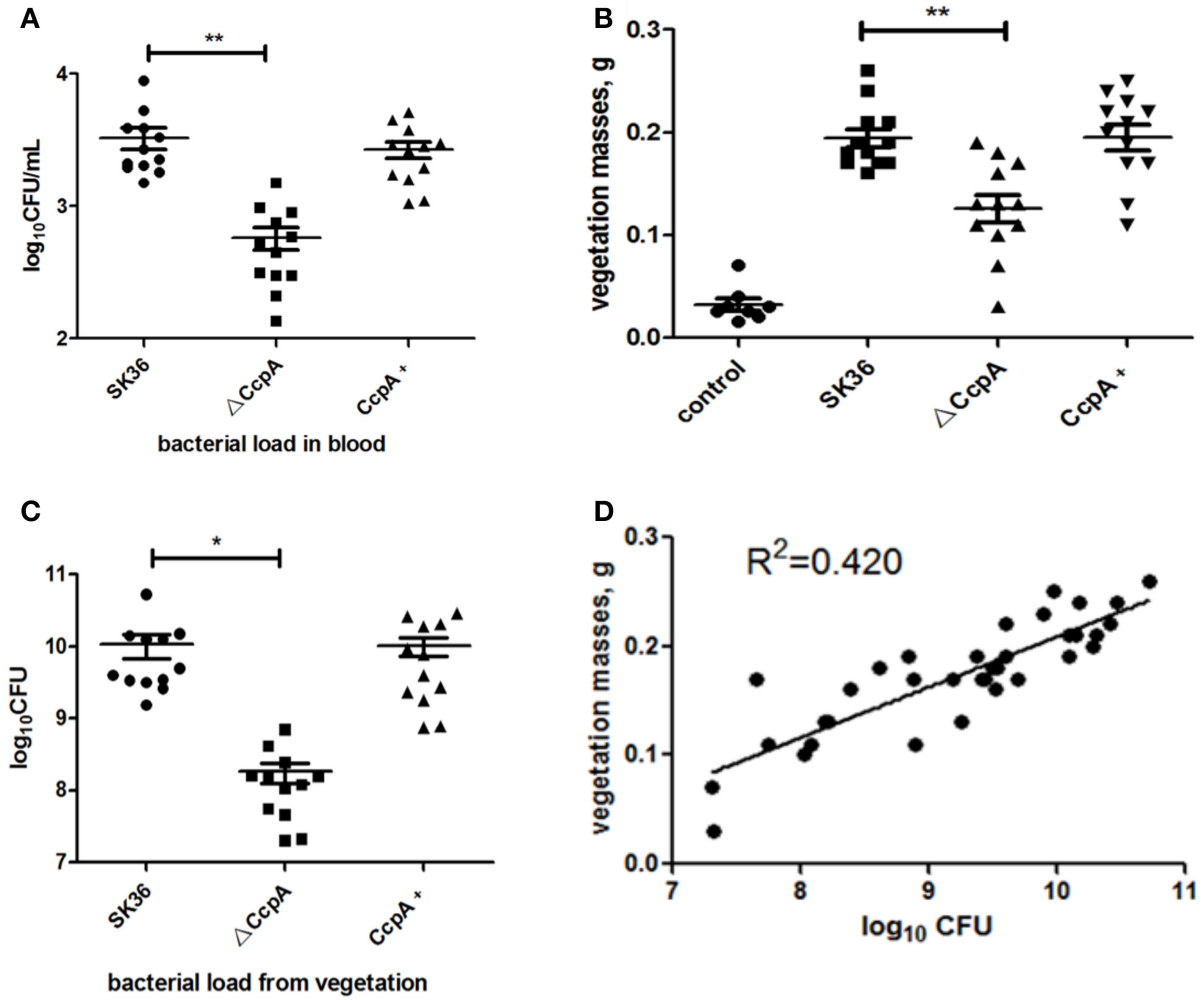
CcpA affects bacterial load and vegetation weight in a *S. sanguinis* rabbit IE model. **(A)** Bacterial loads in blood of the rabbit IE model were enumerated as log10 total CFU 24 h after infection. **(B)** Vegetation masses from the valves of each rabbit IE model were weighed 3 days after infection. **(C)** Bacterial loads in vegetations of the rabbit IE model were enumerated as log10 total CFU. The data presented are mean value standard error (SE). *,**Indicate the significant difference at *P* < 0.05 and <0.01, respectively compared to the SK36. **(D)** Plot represents the correlation between the vegetation bacterial load (total CFU) and vegetation mass. *R*^2^ = 0.420 (*n* = 36) indicated that there was a correlation between them.

Some studies demonstrated that disruptions in biofilm formation result in attenuation of virulence in some streptococcal species (Shenoy et al., 2017). However, the relationship between *S. sanguinis* biofilm formation and its pathogenicity in endocarditis was controversy, previous works have shown that there was no correlation between biofilm formation *in vitro* and virulence *in vivo* for *S. sanguinis* (Ge et al., 2008; Zhu et al., 2018; Baker et al., 2019). We then further probe the role CcpA plays in virulence of *S. sanguinis* in a rabbit infective endocarditis model. In this study, rabbits were inoculated with 1 × 10^9^ CFU of SK36, ΔCcpA, or CcpA+ strains by intravenous. Twenty-four hours after inoculation, bacteria recovered from the blood ranged from 10^2^ to 10^3^ CFU per 1 ml of blood. The counts of ΔCcpA were obviously fewer than SK36 and CcpA+, *P* < 0.01 (Figure 6A). Three days after injection, the vegetation masses from the control group were barely observable (mean = 0.032 g), while the resulting vegetations from SK36, ΔCcpA, and CcpA+ were apparent in macroscopic lesions, the means of the vegetation masses were 0.194, 0.126, and 0.195 g, respectively, there was a significant difference between ΔCcpA with SK36 (*P* < 0.01; Figure 6B). Bacterial load from the vegetations per rabbit varied from 10^7^ to 10^10^ CFU, and the bacterial load of ΔCcpA was reduced compared to SK36 and CcpA+ (Figure 6C). Furthermore, the bacterial load and vegetation masses were significantly correlated, *R*^2^ = 0.420, *N* = 36 (Figure 6D). According to this data, CcpA seems to be involved in the virulence of SK36 and CcpA+ strains.

## Discussion

CcpA as one of the global regulators, influences the transcription of target genes by many mechanisms, but our understanding of CcpA activity and the mechanism by which CcpA exerts its regulation in *S. sanguinis* are limited. Here, we analyzed the transcriptional profile of *S. sanguinis* SK36 wild-type and its CcpA-null derivative (ΔCcpA) by RNA-seq and provided a global view of potential targets that regulator CcpA regulated. We found that 169 unigenes were significantly differentially expressed when deleted CcpA in *S. sanguinis* (down-regulated 85 unigenes and up-regulated 84 unigenes expression) comparing to wild type SK36 (Table 2). When we compared the DEGs of ΔCcpA mutant with Regulon of CcpA in *S. sanguinis* SK36 in the RegPrecise database (the Regprecise search of the *S. sanguinis* SK36 genome yielded 133 genes in 66 operons with potential cre-sites detected in their promoter regions), found 33 operons (60 genes) of the 66 predicted cre sites (133genes) (Supplementary File 1) that matched the 173 genes differentially expressed, 28 of the 66 predicted operons by Regprecise were not among the genes found to be differentially expressed in a CcpA mutant, and 5 of 66 the operons (SSA_1127, SSA_0219 to SSA_0224, SSA_0267, SSA_0395, SSA_1065) were not detected in this study. Here we provide the complete list of Regulon of CcpA in *S. sanguinis* SK36 and the genes found to be differentially expressed in a CcpA mutant (Supplementary File 3). This means that less than half of the differentially expressed genes have a cre site and may therefore be directly regulated by CcpA. In agreement with data from a previous study of *S. mutans* (Zeng et al., 2013) in which about half of the operons predicted by Regprecise were not among the genes found to be differentially expressed in a CcpA mutant. When referred to the literature, we also have known that only 38 of the 226 genes (17%) in *S. aureus* and 76 of the 124 genes (61%) in group A *streptococcus* (GAS) regulated by CcpA present a predicted cre in the beginning of an operon (Kinkel and McIver, 2008; Seidl et al., 2009), all these support our data. Therefore, the regulatory mechanism of CcpA is complicated and analyzing the sequence of the promoter region using prediction tools is not a desirable method to recognize potential target genes of global regulator CcpA.

Although RNA-seq is an efficient method for determining transcriptional variations between biotypes, however, in some scenarios, differences observed at the transcript level are not seen at final protein expression, reflecting the importance of post-transcriptional and post-translational influence on the protein level. Therefore, to characterize the activity of CcpA in *S. sanguinis* is necessary. When analyzed the down regulated DEGs, we found a subset of genes, argB, argC, argG, argH, and argJ, all of these arg genes were associated with arginine biosynthesis. Many studies have reported the association between the arginine metabolism and biofilm development in *S. sanguinis* (Zhu et al., 2017; Nascimento et al., 2019), *Streptococcus gordonii* (Robinson et al., 2018)*, S. aureus* and *S. mutans* (Zhu et al., 2007; Sharma et al., 2014; Huang et al., 2017). Furthermore, another subset of regulated DEGs, comD, comEC, and comX related to the genetic competence system, can also influence biofilm formation which have been reported in *S. mutans* (Li et al., 2001; Aspiras et al., 2004; Perry et al., 2009). When we performed GO terms and pathway analyses Kyoto Encyclopedia of Genes and Genomes (KEGG) pathway analyzes, we found that seven pathways were the most significant pathway represented by the up-regulated DEGs, the down-regulated DEGs predominantly involved in the “Arginine and proline metabolism,” “Biosynthesis of amino acids,” and “2-Oxocarboxylic acid metabolism” pathway, providing that CcpA not only regulated carbon catabolite metabolism, but also involved in some amino acid catabolite pathways. As we all known, many pathogens have been shown that specific metabolic pathways are associated with expression of virulence genes and coordinately regulate the disease progression *in vivo* studies (Somarajan et al., 2014; Bauer et al., 2018; Valdes et al., 2018). Thus, we were encouraged to investigate a possible role for CcpA in biofilm formation of *S. sanguinis*. As expected, by microtiter plate assay, we observed that CcpA inactivation impaired biofilm formation.

Recently, some researchers demonstrated that the arginine biosynthetic genes, especially argB gene, mutation reduced polysaccharide production, resulting in the formation of a fragile biofilm in *S. sanguinis* (Zhu et al., 2017). Exopolysaccharides (EPS) are the primary part of the biofilm matrix, and EPS absence results in a biofilm deficient in some bacterial species (Munro and Macrina, 1993; Yang et al., 2009). However, when we looked into the DEGs, we found that the GtfP, responsible for glucan synthesis in *S. sanguinis*, is not in the list of the down regulated DEGs. When referred to the literature, we found that inactivating the GtfP gene did a marked reduction in the amount of water-soluble glucans in the culture supernatant, but not in the amount of water-insoluble glucans expressed on the bacterial cell surface (Yoshida et al., 2014), while the insoluble glucan is the major component of *S. sanguinis* biofilms reported by Bin Zhu and his coworkers (Zhu et al., 2017). Therefore, it is still necessary to characterize the role of CcpA play in EPS production in *S. sanguinis*. To our surprise, the result of Congo red agar (CRA) plate test showed that CcpA is indeed required for EPS production in *S. sanguinis*.

Some studies demonstrated that disruptions in biofilm formation result in attenuation of virulence in some streptococcal species (Shenoy et al., 2017). However, the relationship between *S. sanguinis* biofilm formation and its pathogenicity in endocarditis was controversy, previous works have shown that there was no correlation between biofilm formation *in vitro* and virulence *in vivo* for *S. sanguinis* (Ge et al., 2008; Zhu et al., 2018; Baker et al., 2019), which prompt us to further probe the role CcpA plays in virulence of *S. sanguinis*. BLASTP analysis of the down regulated DEGs, indicated that SSA_0684 encode fibril-like structure subunit FibA and SSA_0829 (srpA), encode platelet-binding glycoprotein, which mediates the binding of *S. sanguinis* to human platelets (Loukachevitch et al., 2016); SSA_0391 (spxB), encode pyruvate oxidase, involved in survival of *S. sanguinis* in human blood (Sumioka et al., 2017), all of these genes correlate with virulence for *S. sanguinis* SK36. Thus, we characterized the role of CcpA plays in virulence of *S. sanguinis* in a rabbit infective endocarditis model. The results showed that vegetation masses and the bacterial load from the blood and vegetations of ΔCcpA group were reduced compared to SK36 and CcpA+. In this study, we present data showing that CcpA is a global regulator involved in many metabolic processes and related to virulence, these findings will undoubtedly contribute to investigate the mechanistic links between the global regulator CcpA and the virulence of *S. sanguinis*.

Regarding the data from the previous report, CcpA was expressed in wild-type SK36 at different growth phases at a similar level (Chen C. et al., 2019), combined with the growth profiles of CcpA mutant, we selected the mid-logarithmic phase for transcriptome analysis, encouraged by previous studies (Lu et al., 2018; Chen C. et al., 2019). Even though we noticed that some gene expression was influenced by growth phase, for example, previous work has shown that a larger amount of SSA_2315 protein was detected from cultures at late log phase than from cultures at early log phase, suggesting that the expression of SSA_2315 was influenced by growth phase, thus missing some genes is unescapable. Maybe, that is why the SSA_2315 was not detected in this study. Furthermore, several phenotypes, including biofilm and virulence, were shown to be affected by growth phase, therefore, we still could not exclude the contribution of the slow growth of CcpA mutants to affect the phenotypes. This concern was encouraged by a study of *Streptococcus pyogenes* (Paluscio et al., 2018) in which they proposed the pathogen temporal regulation mode that growth/damage balance can be actively controlled by the pathogen and implicate CcpA as a master regulator of this relationship.

## Data Availability Statement

Data were deposited with the Sequence Read Archive (SRA) at the National Center for Biotechnology Institute (accession PRJNA564466).

## Ethics Statement

The animal study was reviewed and approved by the IACUC of the China Medical University Laboratory Animal Center.

## Author Contributions

LZ and MS designed the study. YB, MS, AW, and MX were responsible for the acquisition of data. LS and LZ analyzed the experimental data. YB and LZ were the major contributors in drafting and revising the manuscript. All authors read and approved the final manuscript.

### Conflict of Interest

The authors declare that the research was conducted in the absence of any commercial or financial relationships that could be construed as a potential conflict of interest.
