# Corrigendum: Transcriptome, Phenotypic, and Virulence Analysis of *Streptococcus sanguinis* SK36 Wild Type and Its CcpA-Null Derivative (ΔCcpA)

**DOI:** 10.3389/fcimb.2019.00438

**Published:** 2020-01-10

**Authors:** Yibo Bai, Mengmeng Shang, Mengya Xu, Anyi Wu, Luning Sun, Lanyan Zheng

**Affiliations:** ^1^Department of Pathogen Biology, College of Basic Medical Sciences, China Medical University, Shenyang, China; ^2^Department of Scientific Research, Peking Union Medical College Hospital (East), Beijing, China; ^3^Department of Pathophysiology, College of Basic Medical Science, China Medical University, Shenyang, China

**Keywords:** CcpA, RNA-seq, DEGs, EPS, biofilm, *Streptococcus sanguinis*

There is an error in the order of the authorship list in the published article. Instead of “Lanyan Zheng^1^^*^, Yibo Bai^1†^, Mengmeng Shang^2†^, Mengya Xu^1^, Anyi Wu^1^ and Luning Sun^3^” it should be “Yibo Bai^1†^, Mengmeng Shang^2†^, Mengya Xu^1^, Anyi Wu^1^, Luning Sun^3^ and Lanyan Zheng^1^^*^.”

The authors apologize for this error and state that this does not change the scientific conclusions of the article in any way. The original article has been updated.

