# Supplementary material for: Transcriptome, Phenotypic, and Virulence Analysis of Streptococcus sanguinis SK36 Wild Type and Its CcpA-Null Derivative (ΔCcpA)
Source: Front Cell Infect Microbiol. 2019 Dec 4;9:411. doi: 10.3389/fcimb.2019.00411 (PMC6904348; doi:10.3389/fcimb.2019.00411)
Supplement: Supplementary file 1 [file Data_Sheet_1.docx]

Supplementary file 1. Reads quantification

Table 1. Summary of the RNA-seq reads and mapping of the 6 libraries with the reference genomes of SK36.

| Samle | Total high quality  raw reads | Percentage of reads mapped with SK36 Genome |
| --- | --- | --- |
| WT_1 | 7147416 | 79.64% |
| WT_2 | 6990099 | 81.44% |
| WT_3 | 7563082 | 81.84% |
| ΔccpA_1 | 7464982 | 82.43% |
| ΔccpA_2 | 6883000 | 77.38% |
| ΔccpA_3 | 7026308 | 76.14% |

Figure 1. The sequencing saturation analysis of transcriptome data.


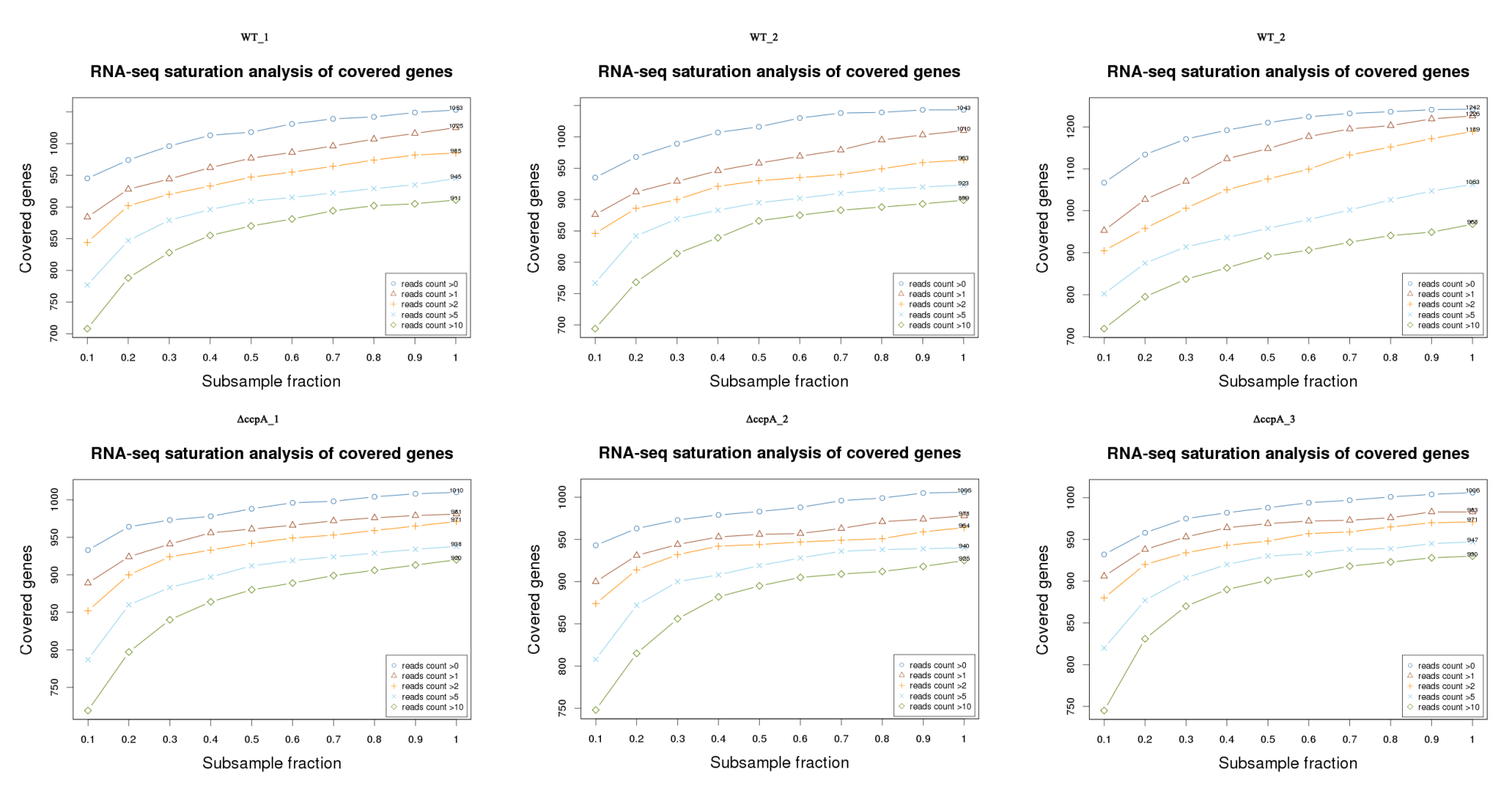


Figure 2. Gene expression level analysis


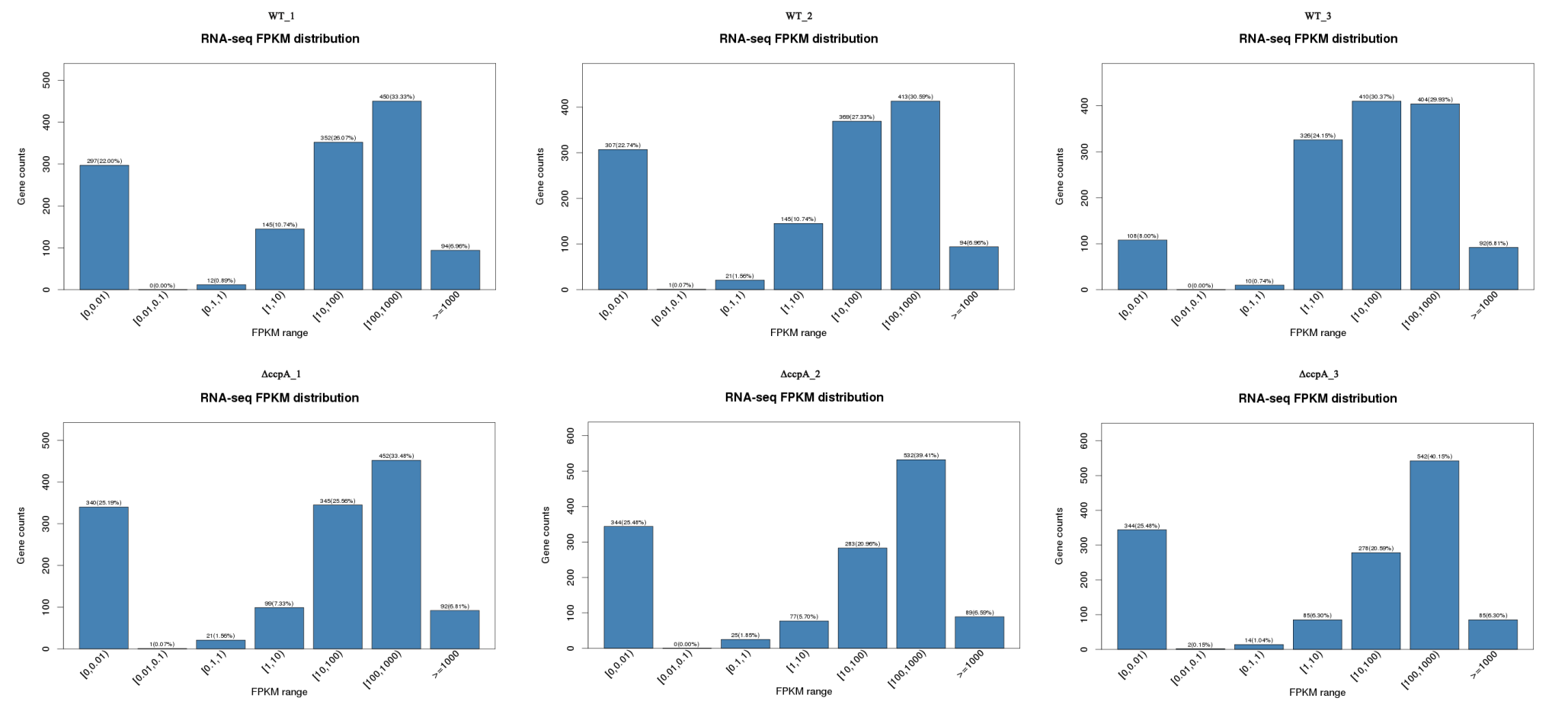


Figure 3. Gene coverage distribution of transcriptome data


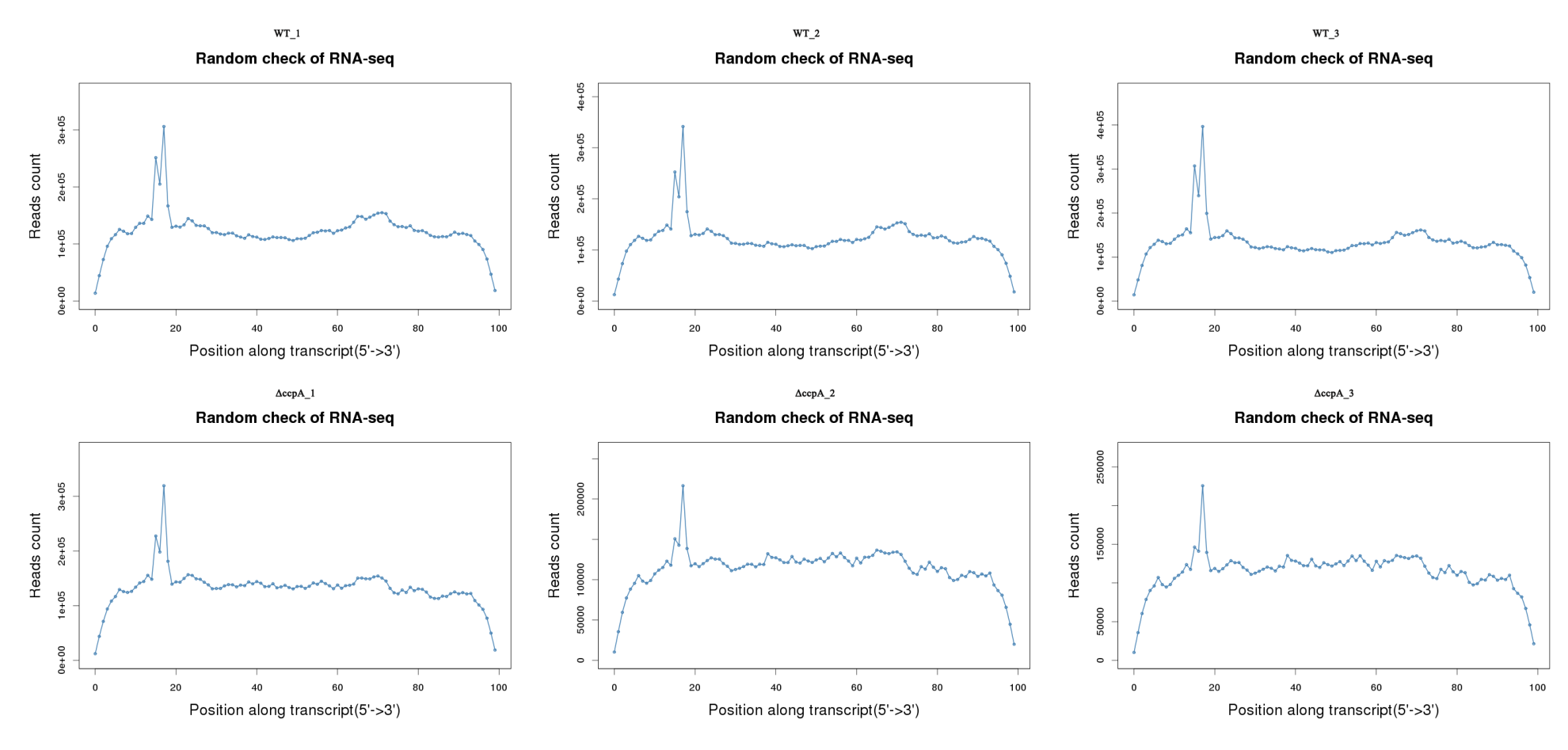


**Assembly transcript data**

>c107_g1

CCAAAATAAAAAAGACTGAGACAAAAATGCCTCAGTCTAGCTTTATTATGTTCAAAAAATATTGTGGCTGAGACAATTTAAATTGTCTATCAGACCTTTTAAACCTGAACCTCAAAATAAGTGGGCCAAGCTAAATTTTAAAAAATCAAGTTCTGTCCTACTGTCTTCTACTGAACCTTCTTGGCCAGCATAGTCGCAAAGCGCAGCTGAATTCGATTGCCGCTTTCATCATGGCGATGGAGGTGACCAGGATTTTCATTGTACTTAACCAGCTCCCAGTCCTTGTAATACTCGGCAAGCTCACCTTCCTTAAAAGTAAAAGGAAAGGGAACAGAGCAAGGGAAGTCTTCTGTATCCATAGCACAGACGATGAGATTGTAGCCACCAAGCGCTGTGTGCTCTTGCATATTGCGGATAATATCCGGAATCCGCTCCGCCTGCAAGAACATAAGGACAACAGTAGAAACGATCAAATCATAAGTCTGGGTCAGACTAGCTGAGTTGATATCGTAACTACCCGCTGGAAGATCCAAATCCTCCTGCTCCACAATGCTACGTAAGATCTCCAGTGCCAGCTCATTCTGATCTACGGCCGTCACTTCAAAGCCCTGCTTGGCTAGAAAGAGAGCATTACGACCTTGACCACAGCCTAGATCTAATGCCCGCCCTGGTCGGACTGTTTGCATAGCTTCCAAAACCTCTGAGTGAACTGGATTGCTGCCGTATTTCTTTGGGAAGTAATCCTCAGGCCGACAGTAAAACTCCAAATACCACTCTAGGTCATCAGTCAGGGCTTCGACCCGATGCCAAGCTTGGGGCTGGGCAAAAGGATTATCCTGACCAGCCTCAAAAATGTGCTCAGCCAACTCCTCACCTTCCTCTGACATCTCGACAAACTTAAGGCGTCCCTTGAGAACAGTGATTTTGCCCCAAGTTCCCTCTTTGGTATTGTGCTTTCTCTTTACCGCTTCTGGCATGGTCTCAGCCGTCCACAGCGGCATACGTTTATAAGCTAGCAATTCTTCTGTCATAAAAAATCCTTTCTCTAAAAGCTACGCTACGAATAATCTAACTGTCTTTATCATACTCGAAAAGCACAGACTTGACAAGTGGCAAATCGAACGGACTTCCCTGCAAAAAGAAAGAACTGGTCAATCAGCCAGTTCTTTCTCACATAGGAAGTTTACTTTCTTTTACTTTTCAGCATCTATCTCTTTACGACTCATCTAATTATCCTTTCTATTCATCTTAAACATCTAGTCTTTTTAAGCTGATAGCTTAAAGCTATTAGTTACTTCTTAGAAGGTGATACTTGTTCAAAATTATAATACTTGACAAACTCTTCTTGGCTGTTGACAATATGTTCCTGACCATCTATTGAGCTATCTGCTGGAATCGTCACATTTTCAAATAAATTGACTCCTTTTTTATACAGTAACTGTAAAAACGAAAGACTGTTTTCAAAATCAGGTTTGTCAGTCAAAATAGCAATCATACCATTCTCGTCTTCTAAAAGTGCAGCTGATTTAGGATATTTTTGGCGGATAAGGGCAGCCGAAGCTAGTTTTTTACCTTTATTAAGATACAATCCAGAGTTATGTAAAAAATAATCTGATGATAAAGGTAATAACTGACTAAAATACGAAGAGCTTTCAGCTGTCCATTTATCCTGTTCATCACCGGCCTCAACAATCTTACCACTGTTCAAATCAATAGATTTAAGGAAACCGTCTGTATAATTATTTGCTGATGTCTTAAGATAAATATCTAATAACTCTCTTCCCTTACTTACATATACAGTATTTTGTATTCTACTTGGGACAGCATCTGAATCATATTCTCTAACCATCTTAAAAATATCATAATCCTTAGAAGTCAGTTTCCCCCCTTTTGTATTGTAGACGGTAATCCCCCAATACTCTTGCCCTTCTGCTACATTATGAATATTTCCATTACTATTTAGCTTTTGATACTCTTGTACAATTTTTGATCCACCCACTCGTGCATCCTGAGGATACAATCTTTTATTTGCTTTTGTTAACCAATAATCCAAGGGGTTTTCATAAGACCATTTCATTACCAAAGCATTATTATTTTCAATAAATTGATAAGATTGAATTTTCTTTTTTAATGTAATTTTATAATATGGGGTTATATAGTATGCCACATATCCCTGATAAGCTAGATAGGGTAAGAACATAAGCAACAGCAGCACAACGATAATCAACTTACTTTTTTTCGACAATTTCATATATAAACCTCCAGATACTCATTAGAC

>c110_g1

CGAGGTACTTGCGCTCACTGACGCGCTCGTGGAGGCTGAGGTACTTGCAGATACAGATGCACTCGTGGAGGCGGACTCGCTCGCTTTCGCAGATGAGCTTGTTGAAGCCGACGTGCTTGCTGAAACTGAAGCACTCGTCGACGCGGACGTACTTGCGGATACTGACGCACTTGTTGATGCAGATGTACTTGCTGATACAGAGGCACTTGTGGACGCTGACGTGCTGGCGCTCACGGACGCACTAGTGGAAGCTGAGGTGCTTGCACTTACTGACGCACTCGTGGACGCTGACGTACTTGCACTTACTGAGGCACTTGTCGACGCGGACGTACTTGCAGAAACTGAAGCACTCGTTGAAGCGGATGTGCTGGCGCTTACTGAGGCGCTCGTGGACGCGGACGTACTTGCGCTTACGGAT

>c111_g1

AAGCGTGCGTGGGTTCGAATCCCATGTCTTCCGTCATCATATGCAAGAAACGCTGTTTGGCGTTTTTTCTATTTGAAAAATGTTATGAAAGAAAGGCGATTCTTTTTGAAAATGAGTTTTTTGGAAATGCTTGAAAACAATTTCAGCAAAAAAAATAAGTAAATTATTGTTCATTTTATATAATAGTGTGGTAAAATGGGATTAATGGATTTTTAAAAAGGATATAAATTAAAGGGAGAATTATGTTTTTTAAACGTCAAAAAGGTAAGTACCACGAAGTAGAGCGTGTGACTCGTTTTAAGCTGATTAAATCGGGTAAGCATTGGCTGCGTGCTGCGACATCACAGTTCGGCCTTTTTAGATCAATGAAGGGGGGAGGCCTTTCATCGATCGAGCTCAAGGTAACGGAAGAACAAGTTACCGATAAGAAGAGCGGCATAGACTTTTTGAGGGGCATTGTCGCCACGGGAGCCGTGCTGGGCGGAGCTGTTGTGACAAGCACAACTGTCCATGCAGAAGAAGGTCAGGCTCTGGAAAAGGTCATTGATACGACAGACGTTTTAGCGACACGTGGTGAAACAGTTTTAGGAGAAGAAAGCAGTGCTGCTGAAGCAGCAACCGCAACGGCAAGCTCACATACTGAGTCAGAGTCTGTATCAGATACATCTTCAGCCAGTGCCAGTGCTTCTGTCAGTGCATCTATCTCAGCCAGCATCTCAGCTTCAGAGTCAATGTCACAGTTTAGCTCTGCCTCTATCAGTGCCAGCACATCTCAAGCAGTCTCAGATTCCTTAAGTGTATCTGAGTCTCTTTCAGTGTCTTCAAGTACATCTGATTCGGTTAGTGCATCTCAGCCAGCATCGACCTCAGCTAGCTCCAAGTCAGAATCCACTGTAAACAGTCAAAGCGCATCATCTGAAACCAATAGGTCTTCGACTAATAATGGCTCAACCAGTTCCTCAAGTGAAACGGCTCGTGTTCGGAAACGTCGAGCTACAGATACAACCCCGCCTACCATTACAGTTCCAAGTGATATTATTGCATATCGTGGTGAGGAGTTTGAATTTTATTTTGAAATCACAGATGATAGCGGTCAAGTTAAAAATATAGAATTAAGTACTTTTGGTAAACCACTGGGTTTAAATTGGTTAGAATATTCAGAGGATAATTTTAATGTACCGGGCAATGCCACTTCGGATAATCCCTTGCGAGTTAGAGTACATGGTACAGTTCCACTAAATGAGCCGATTCCCGCTGATAAAAACAGGGCTCAGTTTACTCGGACTATTAGGGCTTGGGATGCTGCTGGTAATGTCTCTTCAAATATTACTTTTGTAATTAAATATAGAGCACAGACCGATAAATATAATCCAGCTGATCCGACAATCACTTATGTAGATAGGCTTTCTAGTCTATCTCCAAGTGAGAAAAATGCGGTAGAGGCGGCAGTAAGAGCAGCCAATCCTCAGATTCCAGCAGCTGCGAGAATTACTGTTTCTGCAAACGGAACGGTTACCATTACTTATCCAGACTCATCTACAGATACTATTACTGCAAATAGAGTAGTAAAAGATCTTGCATCAAGTCGTTCGGCTTTAACATCAGCTTCTACATCCGCATCAACCAGTGCTTCAGTGTCTGCTAGTACCTCAGCGTCCCTTAGTGCATCGACCTCAGCGAGTCAGTCTATTGTAGATAGTAAATCGGCTTCTGTAAGTGCATCAACATCTGCAAGTACCTCGGCCTCCACGAGCGCGTCTGTATCCGCAAGCACGTCAGCGTCAACGAGCGCGTCAGTCAGCGCAAGCACCTCAGCATCGGCGAGTGCATCAGTAAGTGCAAGCACCTCAGCCTCCACGAGCGCATCCGTATCCGCAAGTACGTCCGCGTCAACGAGTGCGTCCGTAAGTGCCAGCACGTCTGCCTCAACGAGTGCATCCGTATCCGCAAGTACGTCTGCATCCACGAGTGCATCTGTATCAGCAAGTACGTCTGCCTCAACGAGTGCATCTGTATCAGCAAGTACGTCTGCCTCAACGAGTGCATCTGTATCAGCAAGTACGTCTGCCTCAACGAGTGCATCTGTATCAGCAAGTACGTCTGCCTCA

>c112_g1

GAAGCGGACGCTCCTTGATATAGCGTTCCCGAGCCCATGCTCCTGATAGCTGCAGCCAGTTATAATCAGCATCTGGCAGATCCAGCGACAGACTAGCTAGAGATTCCAAATAGCAAGCTTCCCTGCCTTGATTAATCACTTTGCTAGAGCGAGCGATAACCGGCCAATCAGCAAAAATCGTATAAAACAGCTGAACTTCTACTTCTGTCACCTCATCTCGTAGGGTTAGAATAAGAGTCTTAGCCTCCGTTTGAGTCTCTGTATAAGTAGCGGGCAGCCCCTCTAAAGCAGGCTTACCATCTACTATCTGATGACTGACATAGACAAAATCAGTAATACGCGAGCCATTTTCCTGCCGCAGACAGATAGCGGGATGACGAAAATCTGTCGTGCCGTATTCTGGAAATTCCTGCCGCACATGCTCCAAAGAATAGCGTAAATCTCCTTCTTTCCGATAGGTCGTCATGGGCCGATGCTGCAGCTCCACCAAATAAGAATAATCCCGCTCTGGTACCACCGCTCCGTAGTAAAGCTGCAAGAGTTTACCATCCTCATAAACCCGAAAGATATAAGAAATCTCACCGTTAGTCAGATGAAATTGCCTGCACTCTTCCTTAAACCAAATACTCGCCTTATTCATGGTCTTCTCCTTTCACAGTCTGCAATCAAACTTCCACTTTCATTATAAAAAAGGCCACCCCTTTTGGTAAGCCTTTCTTTCTAGTATTATTTATCCAAATGTGACTTTTTACAGCTCCTGTCCCAGATGAGAGCTAGCCTTGCGAAAGTTCAAGGGTGACTGGCCACGCGCCTTCTTGAAGGCTTTTGAAAAGCTCTGTGTTCCGGCAAAGCCTGTCTTATTGGCAATCTCTTCCACCGAAAGCTGGCTGCCCGTCAGCAAATCAGCTGCCATATTGAGTCGCAAATCATTGCTGTACTCCTTGATCCCCTTGCCTACTTCATCCTTGAAAAGCCGAGATAGGTAGGAAGGGTGCAGCGCCAATTTCTGCGCCAACTCCTGCACAGATAAACTTTCTGGCAGATGACTGCTCAGTAGTTCCAAGACCTGCTGGACATAAGGATTGACCCGACGGCTTTCCGTGATTAAGGATGTCTGCTTAAGACGTTGGGACAAGAGTTCTAGAAATCGATAAACTTGCCTTTGCAGAGCCAGTTCAGCAGTTATTCCACTGCCCTCATAGGCCAGACTTTCAAAGACCAAGGCCTTAAATTCCTGCAAAGAGGTCAGCTCAAAAGACCACTGGCCAGACTGTAAGCCCAGATCCTGCAGATAGCGAGGCAAGAGACGCCCCCCCAGTCCCATCCAGACATAAGACCAAGGCTCTTCCCTGTCAGCCTGATAAAAGACCGATTGACCTGGCGGCACCACAAAGCCCTGCCCTTCCTGCAAATCATAGCGCTGATTGCCCACGGAATAAGTCCCCCGCCCCTCCAGTACGATGTGAATGACATAAACATCCCGAACAGCCGGCCCAAAGCTATGATGGGCTTCCGTTTTTGAATAACCACAGAAGGCAAAAAAACAATCTTCTTGGCCAAACTGCATAGACTTAACCAAGGTCAGCGAAT

>c113_g1

CCTCTAGACAGTGCAAAGGGAGCATGACATAGATTATAGCGACAAATCTGCTTCCAGCATCTAGTGTAGTCCAATGTGTGATAAGATATAAGAGAATACCGATTCCCATAGCAAAGAGAATGGGAATCCAGATTTTTAACCAAGCAGAAATCAGTTTGAACATAACAACACCTCATTTATCAAAGTACCCACCACTGGTAAAGAGACCAGTAAAGGATATTAGCGACCACTGCCAAAGCAGATAGACTAGTCATGGCCGTCAGGAAGAGGCGGCCTTTGCTGAACTTTTCTCTGGATTTTCTGAAGAGCGGCAGCAGAGCAGCAGCGGTTAGCAGGAGCCCCAAGGCAGCAAAGAGCATGTAGCGCCAATGAGCTAGAGAATCGAGGTCATCTGACATCAAAGGTATCATAATCATTAGCAGGTTTAGGGGAACAGCCAGAATACCAAGAGAAGTCAGCAGATTCCAGACCTTCCAAGTGCGGTCTGTGGGCTCAACCGTTTTTCGGGAAATCAAGCGATAAACTCCTAATAAAAGGTTGGTGATGATCGTTCCAAAACTATAGACAATTCCAAGGATGCCTAATCCTAAGATGATATAGTCCTTATAGAAGTCAAAAGCAGAGATTTTCTCATAGTCCACCACAGCGACGGGAATTGTAATCTTTCCCTTGCTCTCATAGATGGTCCAAAAATTGGTACTCAAGATTCGGTTCTGAGCAGGATTGTCAATCTTCTCTATTGAGGGCATCATGAGTCTAAGAAAGGATAAGGGGCCATGGAGAAAGGTACGTGGAGAACGGTAGTAGCCTGGTGTAAACTGCTTTTGAGTCTCTTCGTCAGCTGTTTTACGTTTTCCGAAAACCAGTTCAGGCATTTGGTAGTTGTAATTCTCTTCCATGCTTTGATTGGTCATGACTATGTAGCCGATGCCACTTTCCAAGTCTAGCATAAGCCGGGAGCTGAAACCATCAGTATTGCCGCCGTGGCCAATGATGCTGACTCCGTATTCGTTAATCCAAAAACCGTGGGCATTGCGGGCGATGTCTGTACCTGGATAGGTCGAAGTAGGAGTGTAAAGAGTATTCCAAGTTTCTGGACGGTCAAAGAGAATCTTGCGCCCCAGCAGAGCCTGAGCAAATTTCTGTAAATCCTCCAAGGTACCGGCTGCTTGGCCAATTGAGTAGAGACCATATTCATAAAAAGCTGTCCCTAGTGAATTTCCCTTTGTATCATAGGCCTTAGATTCTTGACGTTTTTTCTGAACATAGGGATTATCAGATAAGTCCGGTAGGAGAGCTGTCTTGTCCATGCCAAGCGGCTGGAAGATATGCTCGTGGGTATAGTCAGCAAAGGTCTGGCCGGAAATCCGCTCGACAATCAGAGAAGCGAGCCCAGCACCATAGTTGGAATAAGAGGTTACTGTTCCAGGTTCAAAGGACTGGACAGGCTGGAGCATGCGTAGATTTTCCTCTATATTCTTATCACCTTTTGTGTAAGACAGGGATTCATCAAATCCCGTCTGATGATTCATGAGATCCAGCATCGTGATAGCTTTGTCGAAGTGCAGATTTTTAAGAAAATTCTCTGGCAGGTAGGTCTTGATATCTTCCTCTAGGTCAATCTTTCCTTGTTCCCAGAGTTGCATGACAGCTATCCAGACGGTTAGCTTGGTCACAGAGCCCCAATCAAAGACGCTGCTGTTGTCAGCTTTGATGCCTTTTTCTTTATCCATATAACCAAAGTTTCCTTGGTAAATGGTGCTATCCTTGTCAAAGACTGCTGTTTCCATAGCGGCAGTCGTCTTTTCATGCTCCTTGACATAGTCTTCGATTTTCTGGCCAATCTGGTTCCGTTCGATGCCAGATGGGAGTTTCTGTTCTTCAGCCAGTGCGGTAATTGGTCGAAAGAGTCCTAGAGTCAAAAGAGTTAAAAGGATTAGGGAAAATGTTTTTTTCATAGTTGGCTCCTTTCATATTAGAACACCCACCACTGATAGAGGGACCAATAGAGGATATTAGCGGCCACTGCTAAAGCAGATAGGCTGGTCACGGCTGTTAGGAAGAGGCGGCCTTTACTGAGCTTTTCTCTGGACTTTCTGAAGAGAGGTAACAGAGCGGATACGGTTAGCAGGAGT

>c117_g1

TGGTCAAGCGCCTCAATCAAGAATTAGGCATCACCATCATCATTTCCAGCCATATCCTGTCCGAGCTTTATCTAGTCGCAACTCGCTTTGGCTTTATCAATCAAGGGCATTTCATCAAGGAGCTGTCTAAGGAAGAATTTAACCGAGAAAGCGGCGACTATATCATCCTCAAAACAGATAATATCAAGCAAGCTGCTCATTTAGTGCAAGACAAGCTCGGCTACCAGCTCAGACCGACCAACAAGGAAGATGAGCTGCATATTTCCGGTAAGGTACAAGAAATTCGCAAGATTGCCAAGGAACTAGTCCTAGCCGACATCCCAATCGGGGAAATCTACTACGCCCACAAAGA

>c117_g2

TCCGCTCAAAAAAACTCAGCTAATTTAGTGATTAGCTGAGTTTTTATTTTACAAATCTCTCTTCTTCAGTGTAGCCAGTCCTGCTAAGCTAAAGACCGTGATCAGACTGAGAGCAAAGATAGCTGTTTTGATGATGACACCTGGTATTGAAGCGTCTCCGTAGACGAACTCCATCTTGATAGCCTTGAACCATTCATTATTGGCATCAACCATTTGACTAGGGAGGCCGAGTAAGATGTAGGTCATGATATAGGTGATAAAGACGACACTATAGTTTCGAGTCAAGTAGAGGACAAAGCTGGTAATGCAGTACCAAGCATAGGTACAGATGACCTGCATGACAAAGAGTATTGCGAATTGTCCCCAGAAATTAGCTGGCAGGGTTCCCATATTACCGCCGGTTCGGACAAATTCGATGACAATACCTAAAATGTAGAGCAGTATGAACTGCATAGGAAGTAAGCTAGCGATAACCAGTGCTTTAGAGAAGTAATACTGGGTCCGAGTCTTGCCTGTAGTCAGGTTATTGTGATAAAGTTTGTCATTGAGATCAACACCGAGAAGCAAGCTAGTCATGACATTGCAGGCAATAAAGAGCATGCCGCTGTTGTAGGAGACGACATCTATGGCAAAAAAGATACCGTTGTCGGAACTGACAACTTTGGAAGTGAAAATGGCGAGGAGAATTCCAAAGATGCAGAAAGCCTGCACTCCCCAGAATCCTACGGAGCGGAAGAGACGGTAGTAATCCGCCTTGATACTATTAAACATGAGTGTTTCCTCCTTGTTGGTTGATTAAGTCTGTAAAGTATTTTTCTAAGTCTTTGTGG

>c117_g3

CCACAAAGACTTAGAAAAATACTTTACAGACTTAATCAACCAACAAGGAGGAAACACTCATGTTTAATAGTATCAAGGCGGATTACTACCGTCTCTTCCGCTCCGTAGGATTCTGGGGAGTGCAGGCTTTCTGCATCTTTGGAATTCTCCTCGCCATTTTCACTTCCAAAGTTGTCAGTTCCGACAACGGTATCTTTTTTGCCATAGATGTCGTCTCCTACAACAGCGGCATGCTCTTTATTGCCTGCAATGTCATGACTAGCTTGCTTCTCGGTGTTGATCTCAATGACAAACTTTATCACAATAACCTGACTACAGGCAAGACTCGGACCCAGTATTACTTCTCTAAAGCACTGGTTATCGCTAGCTTACTTCCTATGCAGTTCATACTGCTCTACATTTTAGGTATTGTCATCGAATTTGTCCGAACCGGCGGTAATATGGGAACCCTGCCAGCTAATTTCTGGGGACAATTCGCAATACTCTTTGTCATGCAGGTCATCTGTACCTATGCTTGGTACTGCATTACCAGCTTTGTCCTCTACTTGACTCGAAACTATAGTGTCGTCTTTATCACCTATATCATGACCTACATCTTACTCGGCCTCCCTAGTCAAATGGTTGATGCCAATAATGAATGGTTCAAGGCT

>c11_g1

AAATCCGTAAGACATTGCGGGCATAGTTTCCCTTAATCCGGTCTGCAGAGCCTTGGGCAATAACCTGACCATGATCTATGATATAGATCTTATCCGCATCGTCTGCTTCATTAAGATAGTGGGTTGTCAGGACAATGGTCATCTGCTCTTCTTTCTGAATTTGCTTGAGTAGGCTCCAGATACTCTCCCGAGTCTGAATATCCAGCCCCGTTGTAGGCTCATCTAAAAAGAGCAGGTCGGGGCTATTAAGCAAGGCTCGTGCAATGTCCACCCGACGCTTTTGGCCGCCAGAAAGTGTCCCATAAGGCTGCTTGGCAAAGGCTGACAAGCCCAGCTGAGAGACCAAGCGCTCGATTCTACCTGTCGGCATCTCCTTGTACTGCTTGGCTCTGATGGTCAGATTTTCCAAAACTGTCAGCCGGGCGTCCAACACACTGTTTTGGAAAACCACACCCAACTTAAGCTTTTCAGCATAGCAAATCTGCCCGGATGTCGGCCGCAAGAGCCCTATCAGCATCTGGATAGTTGTCGATTTTCCTGCACCATTAGGGCCTAGAATAGCTGTAAAACTGCCTCTCTCAATCTGAATATTCAGATCATTAACTGCCACCTTATCGCCATAAACCTTGCTAAGATTTTTCGTTTCTACTAACATGATCTTTTCTCCTCTTCTTTACTTCTGAGACTATGATAGCAAGTCTTTTTTGAAAAATCTCAGCTTTTTGGACAAGCGGTAAAATGAGGGAGATGAGAGGTAAAAATCCATACACCAAAAAATCCCCGACCAGAGGTCAGGGATTTGCTCTTAACGGGCGACAATCTTGCGGTAGCTGCGTGAAGTCAGCAGAAAGACCAGAATATAGGTCAGTAGGAAGACTCCGCAAGTTGCAAGTGTTGTCTGAATCAAAAGAGGAAGATTGGTCACACCCAATAGAGCAACAATCAAGCGCAACATGTGGAAGACTGCTGCTACATGTAGAAAAGCAAAGATGAGAGGTAGGAAGAAAACAGTCAAAATCTGCTTACGAATGGTACTTCTAGTCTGCTTTTCATCTAAACCAACCTTTTGCAAAATGATAAAGCCATCTCGGTCTTCATAGCCTTCAGAGATTTGCTTATAGTAAATCACGAGAACAGCTCCTAGAAGGAAGATAACGGAGAGGAAGACACCGATGAAGAGCAAGGTTCCAGTAAGTTCTTGATAGCTCTTTTCTGCACTATAACGGTCACTAGCCATTGCAAAGCTACCATCTTGAGCTTGTTCCTGATCCAAAGTGTCCCTCAACCCCAGTTGGACTTGCTCGACAAACTTTTTACTGCCCTTAGTCTCTGAAGCAACTCCAATGTAGTAGTAATGTTCCACATCGAGATTAACCTCTTTGCTATCATTGACCACCATATAGAGCCCCTGTTCCATGGTTACATCGTTTGGATTAGGGATTTCCCCATGCGTGAAATTCGACGATAAAAGCTGCTTAATCTTCCAGTCCTTACCATTCACTCGGAGAGGCTTGTCCTTATTTAAGGAAATATTTTTCCCGTAAACTAAGGTTTCATCATCTGCTAAGTCTATCTTTTCCCCTGTCATTTTTTCATAATCCTGGCGATTGATGACCGTAATCGTACCAGCAGACTTAGTCAAGATACTTGTATCCGACTCCAGCTCTCTTTGCATTGGAACTGTCACATCATTACCAGCCAGTTTCGTAATGAAGGCTGATTGGTAGAGATAGGTAGTATAGCTTGGCTTCTTCAGACCTGTGTCCTGAGCGACTTGCTGGACCTTGTCTAACAGAGCCTCCTTCTGATCGGTCGATTTCGGCAAGACGACGCTGACATTGTAATCCTTTGGATGCAAGGTAGTAATATAATCCTGACCCCCAACATAGATATTGATTGTACCAACCAGAGTCACTAAGAGCATGGTAGATAGAATGGAGATAGTTGCCAATCCTGCTGCATTTTTACGCATCCGCGAGATGAGATTGGAAACAGAAATGAAATTCTGCGTTTTATAGTAGTAGCCTTTGCGCTTTTTGAGAAATTTCAATAGTGTAATCGAACCTGCATTAAAAAGCAGATAGGTCGCTAGAATAACCATGACCACAGCTATAAAGAAATTGCCGATAGCAGCGACAGGCTTGGTAACGGTCAAAGCCATATAATAAGCCACACCCATGAGCAGGAGCCCCAGCAAGGTTTGCAGCAAAAGGAAACGTCCTTTCTTCTCGCCTGCTTTCTTTTCTTTCATTAGATTAAGAGAGCTATAGCGTAGAAGACGCGTAGAGTTGAGCAACAAAATCACCGCAAAAGCGACACCCAGACTAGCTAGAGTCATCCAGACGTTCTTCCATTGGAAGGTCGAGGCAAGAACTGCCGGCATTCCCATCAATTTCAAAAGGACTGCGTAGAGCATCTTGTCTAAAGCTAGGCCAGACAAGATACCCAGACCGACTGTAACCAGATAAAAGACACAGAGTTCAAAGAAGGTCATGACTAGCAGATGTTTTTTCTCCATGCCCAGCAGACTATAGACTCCCAACTCTCGGGAGCGGTTTTTCATGACGAAGCTATTAGCATAGGTAATGAGGATAAGGACGGCAATCTGAATGACATGGATACCAAACTGCAAGGTCATGCGAGCTGCCGAACCACCATAAGAGGTCTCCATGTTGGGGCTATGAGCCAGTGAGACAAAACTATACAAAATCGCTGTTGCAAGTACCGTCGCCAAAGCAAATGGATAATACAAACTGCGATTTTTAACCAGATTGGACAAAGCCAGTTTACTCGTTAGTTTGAACATAATTGCCCACCTCACTTGCCATAACTGTCAG

>c122_g2

CGAGCTTTCTTAAGACCTGGTTTGGAAAGTATGGATTTCAGTTGGACTAAAAACAGCGTAATATCAAGGATTTTCAGAACGATATCTACTTTTTAATTTCATAAAATATGAATCAATAAATTATAAATAGGGGAATAGTTTAGGTCTGTAAGCATTAAAATAATACACCTAACTTTGGTCACCGTTTTTTAGTTATTCACTCTTTTTTCGGATAGTGTTTTTAAAGTTATGATGTAAAATGCCGCAATTAAAACACTATACATCATAATCGGAGGATAGAGAATTAAGGACAGAATCAATCCCACTCCTTTAATTATTAGGTCAGGTATCAATAACTTCCTATATTGTGCGGTAGCTTCAAGTAATTCTTTCTGATCTATATTGGGTTTATCAATTACTTTATGTAAAAACCAGTTTGCTACCGTTGAAACAATAACGATCAGTCCATAAAAGAGCTGTGCCGTATGATTCATGAAATGACTACTAACTATTCCAGTAGCATAGGGCATAAATGAAACAAAAAATAAAAGATACAAATTCTTTCAAATTTCTCCCTTTTCAATTTGCCTAACTTGACCTGCATTACTTAGGTCGCTTTGCTTTAAATTATACTTTTTTCTTAAACATTTAATACGAGTAGGTATTTCTTGTGAGTAATTTTCATCAAAAAATTTCATATATAATCTCCCTATAATCATGGAAAAGTAGTAAAAATAGTTGTTATCCTAGTTGATTAAAAAAATCTCTGATTTCCTCATCTTTTATAAAATAATATATAATTTTCCCCTCTCTTCTAGTGTCCAAGATGTTTTGATTGGCTAGTTTACGAAGATGGTGAGAGGCAGATGCCATACTGAGATTTAATAAACAGGCTATATCGCAGACACAGAGTTCTTCGACGGCAAGGAGATAAAAGATGATATTTATCTGTTTATTATCAGTAAATTTTGATAAAATGCGAAGTGATTTTTGGACTTTTTCCTTTTCAAGGTAGTTCGTTGCGGTTGTAACATTTTGTTGATTTATAACATCCACTTGACAGATACTATCTTTTTTCATAATTTTTTCTCCTAGCCTAACACAGCCCATAGCATGTCAAAGCTGTTGTTTTCAATCAGGATATACATCCCCAATCCTAAATAGACAACGGCAATAAACCATCTGCTATATTTTTCCAAAGTTTCTCCAACAGAAGGGACTTGTGCTAATTTTTGGGCTGAAAAAACCAAGAGATAAATCATGACTAGAAAAGTAAGTAAAGTCACTATCAAATTCGCTAAATTTAAGGTGGTAAAATATGGGACAAAGACACCAATATTGTCAGCGCCACAACTTGCAAAAGTAATCATAGCTACTAGAAAAATCAGGTTTTTATTGTCTTTTCGCAAACCATCTTTTGCAATAGCTTCTCCATCAGAATCTCCTAAAAGCAAAACTTTGAGGCCTAGGAAAATTGGAATCAAACCGAGTAAACCTAAAATCTCTTTACTAGGAATATAATTTAAGACAAATGCAAAAAGCAAACTTAGGAATATTAGACTAACAGAGCCTAGAAATTGTCCTAAATAGATGTTAATGATGTCCTTTCTGCTTTTTCTTTTGGCAAAAAATAACATTAGGATAATAAGTAAGTCTACGGCTGTCCCAGAATACAGGATTATTGAAGTAACAATATTTTGAATCATAAAACACCTCATTCAAATATATTTTTGAATGTATTCTAACATTAAACTTTGTAGATGTCAACTTCAGCTCCATCAAAATATAGATAAGAAGGTAGTGTACCAAATATTAAAAAGCCCTGCCATCGAAATTATAGCAGGGCTTAACTTCAATATCCAGATGATATATTTATCTAAAAAAGGTAGAGAAAAAGGTAGAGTTTGAATTGAAATACAGTGCAATATATCGCATTTGGCAAAAAGAAAAAACGCTTGAAATCAGCGTTTTTCTTCTGTATTGATTCGTATTGAATGCTTACTTCACTTCTGTAAACACAACGTGCTTGCGAAGTTTTGGTGAGTATTTCTTCAATTGAAGACGGTCTGGTGTGTTGCGTTTGTTTTTAGAAGTAAGGTACAAGCGTTCACCAGATTCTTTGTGTTCAAGTGTAATATTTACGCGCATGGTAGCTCCCTTCTATTATTCTGCTGCAGCAGCTTTGGCGATCTTACGGCCCTTGTAGTATCCTTTAAGGGATACACGGTGAGAGCGTGAGTAGTCACCAGTAGTTTCGTCAAAAGTTACAGTTGGAGCTGTCACTTTGTAGTGAGTGCGGCGTTTGTTTTTCTTCGCTTTGGAAGTGCGACGTGCAGGTACTGCCATTGTTTGTTTCTCCTTTTAGTTTG

>c123_g1

AAGACACTCTGAAACATATGTTTTCTTATATATTTTGTTATTCTCACTTTGAATCAACCTTTCTCTCCACTCCCAATCCATTCAGAACTTCTGGATACAACTTATACTCCGCCTCATGAATCCTAGTTTCAAAACTTTCAATCGTATCATCAGCTAGTCGAGGCACGCGCACTTGCTGGATGATCTTGCCAGTGTCCACACCCGAGTCCACCCAGTGAATGGTCACGCCGCTCTCAGAGACGCCAGCCTGCCAAGCATCCTCAATGCCATGAGCTCCTGGAAATTCAGGCAGATAAGCAGGATGAATGTTGATAATCCGGCCTTCATAAGCTGCCAATAAGGTTGGTCCAACGATTTTCATATAGCCTGCCAGGCAAACCAAGTCAATCTGGTGCTCTTCTAGCAGGTCTACAATGGCTTGCTCGTATGCCGCCTTACTGTCAAACTCTCTAAGCTCAAAGGCATAGCTTTTGACACCTAGCTTATCCGCCCGCTCGAGCACATAGGCATCGCGATGGTCTGAAAAGACAAATTCAACAGGGAACTGCTCAGCAATCACTTGGAAATTTGATCCATTACCCGAAGCAAAAACGGCAATTTTTTTCATTTGATAAGGACACTTTCATTTTCTTTTTTGACAATCCGGCCAACTTCATAAACAGGCTCGTCAAGAAGTTCTTTGACACGATCCACATTCTCAGGAGCTACAGCCAAAATCATCCCTAACCCCATATTAAAGATTTCAAACATTTCTTGATGCTTGATGTGACCATATTTTTCTAAGGCCTTAAAAATCGGCAAGACTGGAATCTTGTCTTCTTCAATCTCAGCAGCCAAGTCATCAGCAAACATGCGGGGCACATTTTCGATAAAACCGCCACCAGTAATATGGGCAATCCCATGAACCAACTTTTCCTTAATCAACGGCAGCAAAGCCTTGACATAGATACGAGTCGGCTCAAGCAGCACTTCCTTGAGTTTCTTACCTTCCAATTCTGGTAAGACTTCTTCACCGGTGTAGTCCGCAAAAACACGGCGCACAAGCGAATAACCATTTGAGTGAATACCGCTGGAAGTCAGCCCTAGAAGGACATCGCCCTCTGCGACTTTAGAGCCGTCAATAATCTCTGACTTTTCAGCCACACCAACAGCAAAACCAGCCAGATCATAATCATCTTCACCGTACATGCCAGGCATTTCAGCGGTTTCCCCACCAATCAAAGCAGCTCCAGACTGAACGCAGCCTTCAGCTACACCAGCCACCACTTGCTCTAGTTTTGCTGGTTCATTTTTCCCAGTGGCAATATAGTCTAGGAAGTAAAGGGGCTCAGCCCCTGCAGCGACGATGTCATTGACACACATAGCCACACAGTCCTGCCCAATGGTATCGTGCTTGTCATACTGAATGGCCAGCATGAGCTTGGTTCCGACCCCATCCGTACCAGAAATTAGCACCGGCTCCTTGACCCCAGTCTGAGACAGGTCGAACATTCCGCCAAAGCCACCCAGCGCCCCCATGACGCCAGCCCGCTCTGTACGTGCTACATGTTTTTTGATTCGCTCAACAACTTCGTAACCCGCTTCAACATCCACACCGGATTGAGCGTATGCATTTTTATTTGTCATAATTTTCTTCCTTACTCTTATTTTTGAAAAAGATTGCGACAACTACCGCTGACCTTGTTTTAATAAAAACTCGTCTTTTCCTTCAGGCTTTCCAGATAGCGCTCTTCATAATCATAGAGCGGAGTTGGGTACTGACCGTCAAAGTAAGCCACACAGAGTCCGCCATTCGGAGCATCCGTCTCAATACCGACTGATTCAATGAGACCTTCCAGAGACAGGTAAGTCAAGCTGTCAGCTCCTATAATCTCACAGACCTCATCAACCGTATGATTGGCCGAGATGAGCTCGCGTCGATTCTGAATATCAATGCCATAGAAACATGGATATTTAAGTTCTGGACTGCCGATAGCCACATGGACTTCAGCTGCACCTGCATCTCGTAGGAGCTGGACGATGCGTCGGCTGGTTGTTCCCCGTACGATAGAGTCATCCACCATGACCACGCGCTTGCCCTTGACAATGCTGGAAACGGCAGATAATTTCATACGAACTCCTTGCTCTCGCAACTCCTGCGTCGGCTGGATGAAGGTCCGTTGGATGTATTGATTTTTAATCAGGCCCATTTCATTTGGCAGACCAGACTCTTCGGAAAAGCCAGAGGCCGCTGACAGGGAAGAATTAGGCACACCGACAACGATATCTGCCTCATGTTGGAATTCCTGAGCCAGTCTGCGGCCCATCCGCTTGCGAGCTGCATGGACATTGACCCCGTGAATGACGCTGTCCGGCCGAGCGAAATAGACATATTCCATTGAACAAATAGCTAGCTGGGTATCTGTCGTATAGCTATCGTAGGTCACGCCTTCATCATCGATAATCACGATTTCTCCTGGCTCAAGGTCACGCACCCATTCGGCACCCACCACTTCAAAAGCACAGGTTTCACTGGAAACGACCCAGGCCCCATTTTTCATACGGCCGATGGACAGAGGACGAAAACCGTTAGGATCCAGCGCAGCGTAGAGCTTGTCCTCTCTCATGATGAGGTAGGCAAAGCCGCCCTGAACTCGTCTCAAGGATTCCTTGAGTTTGTCTAAGAAATTTTCCTGCTCGCTATGGCGAATCAAGTGCATGAGAATTTCCGTATCAGACGAGCTGGCAAAGATGGAACCCTTCTTTTCTAACTCCCGTCTCAGTGAATGGGCATTGGTCAGATTGCCATTATGCGCCAAGCCCATCTGCATATCATAGAAACTGAAGAAGAAAGGCTGGACATTATTGATGGAGGCTCCGCCAGACGTTGCATAACGGACATGGCCGATGGCTGCTTCTCCTGTCAGATTATCCAAATCTGCTGGGTTCTTAAAGACCTCTGCAACCAATCCCAAATCGCGGTGGCGTTTGAGCTTTCCATGGTCGTTTGACAGGATGCCTGCTCCTTCCTGACCGCGGTGCTGCAGACTATGAAGTCCGAAATAGGTGACCTGAGCCGCCTGTGGATGGCCCCAGATTCCAAAAATACCGCACTCTTCATTCAGTGACTTTACTTCGTATGTCATTGTTTTACCTAAATTCTTTTGTCTTGTAAAAGGGAGAAAACCTGGCCTTCCAGGCCAAGCAGACTCCTTTTTTGCTTGCTCTTGCAAGCCAACTTTATTCGTGACTCGCTTGGAAATAACGAACGGCGCTTTCAAACAATTTCTGGTCTTTCTGACCTGGAATATTTTGGAAAAGGCCGTCTTCATAACGTTCTGAGTGCCCCATCTTCCCGATGATTTGGCCGTTCTTGCTCATAATTCCTTCGATAGCGTAGAGAGAGCCGTTTGGATTATACTTGCTGTCCATGCTCGGCTGACCGTCAAAGTCTACGTACTGACTCCAAATCTGACCATTATCGCGCAGCTCAGCAAATTCCTCAGCCGTCACAACAAATTTCCCTTCACCATGTGAAACCGGAATGGCATGGATATCTCCCACTTGCACACCAGCTAGCCAAGGCGAGTTGGTATTGGCAATGCGGGTTTCAACCATCTTGGCCACATGCTGGTTGGCATCATTGTAAAAGAGAGTTGGGCTGTTAGCCCCTGCTTCTTCGAAATTTCCGTAAGGAAGAAGACCGGATTTTACTAAAGCCTGGAATCCATTACAGATACCAATGATGAGACCGCCGCGAGCGATGAAAGCATCGATGGCTTTCTTGACCTTCTCGTTTAGCAAGATGTTGACGATAAACTTAGCTGACCCATCTGGCTCATCTGCAGCAGAGAAACCGCCTGCAAAGAAGATGATATTCGCTTTGTCAATATTGTCAACCATGCTGTCAACGGACTTGACAATGGCTGCTTCATCCAAAGTAACGAATGGAACTAGATTGACCTTGGCTCCAGCTGCTTCAAAGGCTTTGGCTGAGTCGTATTCTGAGTTGGTTCCAGGGAAGACCGGAATGTAAACCAGCGGTTCCGCCACTGTCTCTTTGGCTTTGATAACTGTATCAGCGACAAGAGCCGGCACTTCTTCAATAACTGTTTCTTGTTTAAATTCTGTCGGGTAGATAGGCTCTAGTCGGCCTTCAAAGCTTGCCAAGAGCTCTGCTCCCTCAAGCTGGACACCATTGACAATCAGTGTAAAGTCTGGCTTGGTCTGACCGATCTTCACTGCGTCTGGAATATCCTGCTTCGGACTGGTAAAGACAAATCCGCCCAGCTGCGCCTGCAAAACGGAAGGCAGATCAGCAACATCCACTTGAGCACCAATACGATTACCAAAGCTCATCAAGGCCAGACTCTCTGCCAGACCACCGTATTTAACAGCGGCGGCGGCTGTAATCTCATACTTGTCCTGAATGGCCGCAAAATTTTCAAAGTTATTCTTGATTAAGTCAAAGTCAATATCCTGAGACAGGACCTGTCCTGGCAGATAGTAGATGGACTCTCCTGCTGCCTTAAACTCTGGAGACAGGATGCGCCCAGCAGTAGAAGTAGTCACGCCAAAAGCTACCAAGGTCGGCGGCACGGTCAATTCTTCAAAAGTTCCGCTCATGGAATCCTTACCACCAATAGACGGCAAACCGAGCTGAATCTGAGCTTCAATTGAACCAAGCAGAGCAGAGACCGGCTGTCCAAAACGCTCAGCCTGCTTGTCCATCCGCTCAAAGTACTCCTGATAAGAGAAGCGAGCCTTGGACCAGTCCGAACCAGCAGCAACCAAGCGAGCTGTCGCTTCAATCACTGCATAAGCTGCTCCATGATAAGGCGACCAAGCTGCCACATAAGGATTGTAGCCCTGCGCCATCACAGAGACCGTCTCAGTCTTGCCATGCTCAACTGGCAGTTTCTGCACAGAAGCCTCAGTCGGCGTGATTTGGTAGCGCCCGCCGATAGGATGATTAACAGTAGAGCGGCCAACAGAACTGTCAAAGATCGTCTGCAGCCCCTTTTGACTGGTATGATTGAGGTCGCTGAGTAAGCTCTTGAGGTCCTGCTCCAGACTTGCTTCAGAAGTCACAGCCTGCCCTGGCAGTACTTCTTGGGCATCCACAACCTTGGCATCCACAACCACGCGCACGCCATTGGTGTCCAGAAAACTCCTCTCAATATCAACGATGGTTTCCCCGTTCCAGTGCATAACCAGATTTGGCTTTTCTGTCACTTTAGCCACAACAACTGCCAGCAGATTTTCCTTAGCTGCTGCAGCGATAAATTGTTCCACATCTTCTGGACGAACCACCACTGCCATCCGCTCCTGCGATTCAGAAATCGCGATTTCTGTACCATTAAGTCCTTGGTATTTGAGCGGTACCTTGTCAAGGTCAATTTCCATCCCATCTGCCAATTCACCAATAGCTACGCAGACACCCCCGGCCCCAAAGTCATTGGATTTCTTGATGAGACGAGTCACATTGCCATCGCGGAAGAGACGCTGAATCTTGCGCTCCTCAATAGCATTTCCTTTTTGTACTTCAGCACCAGCAGTCTCCACAGACGCAGCTGTCTGAACCTTGGACGATCCAGTCGCTCCACCGACACCATCACGGCCCGTCTTGCCTCCCAGCAAGATAACCACATCACCTGCCGCTGGTTTTTCACGGACAACATTTTCCTTAGGAGCCGCACCAACAACTGCACCTAGCTCCATCCGCTTGGCTACAAAGCCTGGATGGAAATACTCACGAACATAGGTCGTTGCTAGACCAATTTGATTCCCGTAAGAAGAATAGCCATGCGCTGCAGTTTTTGAAATGACCTGCTGAGGAAGCTTGCCAGCCCGCGTTTCAGCAATCGGCTCAGTAATATCGCCTGCACCTGAAATCCGCATTGCTTGATAAACATAAGAACGACCAGAGAGTGGATCACGAATGGCTCCTCCGATACAGGTCGCAGCCCCTCCAAAAGGTTCAATTTCTGTTGGGTGGTTATGGGTTTCATTTTTGAACATCAGCAGCCAAGGCTCCTTGATACCATCCACATCCACTTCAATCTCCACTGAGCAGGCATTGATCTCATCAGACACTTCCATGTCATCCAGACGGCCATTGGCTCGCTCGTAGCGACCAAAAATAGTCGCCATATCCATAAGAGTCTGCGGTTTGTCCCCACGTCCCAGCTCATCGCGCATAGCCAAGTATTTATCATAAGTGGCCTGCAGTTGCTTTTCAAACTTAGAAGCTGAAAAATCAATCTTCTTCAACTCTGTCTCAAAGGTCGTATGTCGGCAGTGATCAGACCAGTAAGTATCTAAAACCTTAAGCTCAGTCTCTGTCGGAAAACGGCCAATCGACTTGAAATAGTCTTGAATGAAGAGCAAATCTGCCACTTCCATAGCCAGACCATGCTCCTGCTTGTAGGCTTTAAAGTCCGCTTCCGTATAGTCCTTAAAGAAGTCCAGAACCGGAATGGTCTTGTCAGACTCAGAGAACTGCTCCAGTTGGATCGGCTGCTCAATATCTTTAAAGCGTGAATCAACTGGATTCAGCAAGTATTTCTTGATTGCTTCCAACTCGCTGTCTGAGATGTCTTTATTGACCAAATAAAGCTGAGCTGTCCTCACCAGAACATCGGTCCCAGCACCGAGCAAAAAGAGAGCCTCCTGAGAGCTGGCTGCCCGCTGGTCAAACTGACCCGGCAACGCTTCGATGGCAAAAAACGTTGTCTCTGCAAGTGCATCCTCTACTTCCTCCTCCGTCAGCAATCTATCAGTCACCTGCTCAGAGAAAATATGCTTTTCAGCCCGAGCGACCAAATCTTCTGCCAGATGAAAGACATCATAAACTTGAATCATCCGAAGATCTGACAATGTTTTCAACTGAAGATTATGCGTCAGCTCTCTCACCAAGCTCTGTGACTTCACACCGAAGTTGCTCTTCTTCTCTACAAAAATACGCTTGTCCATGATTTTTCACACCCTGTCCTTACTTAATCGCCTGCAATTTTTCCCAAACAACCTGATAGACATCTGTCAGTTCACCAAGGCCACGCCGGAAAACATCCTTGTCCATGTGATGGCCTTCCGCATCCCAGAGGCGGCAATTGTCTGGTGAAAATTCATCCGCCAGAATAATCTTGCCATCTTTGTCAAAGCCGAACTCTAACTTGAAATCAATCAATTTCAGTCCGATTTGGCGGAACCAGTCTGACAAGAGCTTGTTGATTCGACGAGTCTCCTCTTTCAAAAAGGCAATTTCTTGATCGCTAGCAATCTTCAAGAATTTCACATGCTCGTCATTGATGAAAGGATCGTCCAAATCATCATTTTTGTAATAAAATTCCACAATTGGAGTTTCAAGCGCGATACCCTCTTCTACTCCAAAACGTTTGGAAAAAGAACCCGCTGTGTAGTTGCGCAGCACCACTTCCAAAGGAATAATCTCTACTTTTTTATTTAGCTGGTCCGTATCCGAAACCTTCCCAATAAAATGCGTCGCCACACCAGCTGCATTGAGTTTCTCAAAAATAAAAGATGAAATCTGGTTGTTGAGCACTCCTTTACCAGCAATCTGCTCCTTCTTGACTCCATTAAAAGCCGTTGCTTGGTCCTTGTAGCGTGCCAGAATGACTTGCTCATCATCTGTAGAGAAAATATCCTTAGCTTTTCCCGAATATAATAACTTATTAGACATTTTAATATTCGCCTTTCGTGGTTTTATACCCTCATTTTATCATCTTTGAATATATTTTACAAGAAATTCCGTATAAACTTTATATACCATATACCCAGTTAAAGAAATCGTTTCCTTTCCAAGATACAAAAAAAGACCGGAA

>c125_g1

GTCTAGAGGAATGGAGATTTCCTGGCGGTTTTCATTATAACTACAACATGCTTCGGCGCTCTCGAAAGCCCGATTGCTATCCTATGAATCAATTTTCTGATATGCTGACTATTATGTTAGCTGAATTAATTGGTATTGTCTGTCTTTTCTACGGTGTCAATCAGATCATTGTCATCTGGAATCTTATCTTTTGCTTTTTTGAAATGATTGGCCACCTGATTTTTGGGTTCAGTATGTACCGACGTTTTCGAACAGTAGGAAAAAGAACCATTTATAACCCAGGTTTTGCGACAGCGGTTGTCTTTACGCTTCATGCTCTCTACTATGTTCTGATTCAGTATCCTACAAATCTCCCCAGTTTGCCAATAATCATTTTAGCCATTATCAGTGGAATAGTTCTTGTAAGTAGTGTCGTTCTTATCCCTGAGCAACTGTTCAAATCAAAAGAGACCCCTTATCCTTTTGATAGCAACCGCTATTATGAAAAATATATTGCAAGGGAAAAAAACTGAATTCCCTTCCCTATTTCATAATCTTCTTCAAAAATTCTATGAGACGTCAAAGGCATAACTGAGCGACTAATACTCACCGAAAACCAAAAAGTTGCCTAGTTTTATTCTGCTATTTTAAGTTTGGCCTTGCGACTCAATCAGACATATCTAATGTTTGTTAGCGAACTACTGAAAGATTGCAGGGAGCAAGCATTAACTGGTGCTTCTATTGCGGGGTTGGGATGCATTCTTCTTATTAGTTACAGTAAATTGCCAATCAGTATGACAGCTCTTTTCTCACTTTACCTGATTAAACTTCTATTCGCTTTTAGTGTAAATCACCATACGACCGCTTGGAGTAAAGATTATAAATAAAAGTATGCGAGTCTAAAATATGATGAACAGAAAGGAATCCCTATGTTGAAAAGAATCTTTATATTTATCCTTAAAACAATCACTTGGCTGGTAGGACTCTTAGCTCTGGCCTTATTGGCTATCTTTCTCTATCACCGCTTTCAAATAGCTCAGGAAAGCAAGCTCATTGAGAAGCCGATTGGTCAGCTAGTCGAGGTAGAGGGTAAGAAGCTTAATGTCTACACTGCTGGCAAGGGCAAGAAGACTTTGGTTTTTCTAGCAGGACTGGGCACCAATGCTCCGGTGCTGGATTTCAAAGCTCTTTATTCCAAGCTGGAGGATGATTATCGTATCGTCGTGGTAGAACGTCTGGGCTATGGTTACAGCGATGATGGAAACGATGACCGCTCATTGGATAGACAAGTGGAGCAGACACGTACGGCCCTCAAAGCTGCTAAGATATCCGGTCCTTATGTCCTGGTACCACATTCGATTGCCGGTTTAGAAACGATTCACTGGGCCAATCATTACCCAGAAGAAGTAGAAGCTATTATCGGGCTGGATATGACCATGCCTCATACAGATGTGGCCGACCAGCAGAAATTTTATGGTTCTTATCAGACTATCCAGATGGGGAGAATGCTAGGCTTGGCTCGCCTGCCGATTTTCTTTGATGAAAATAGCAGCCCAGCCATAAAGTCTGGGGCTCTCAATGATCAGGAAAAAGCTGTCTTTAAAGCCTTGTTCCACCAGCGGTCAATCACCCAGGCGGTCATGAACGAAGTCAAGAATCGGAAGAAAAATGTTGAAACAATTAACAAAGAACCCGTGCCCCAGATTCCAACTCTTATCTTTGCGGCTGTCCAGAAAGGCGGAGAAACTCCTAAGTTGGAAGAAGAGTTTGTAGCGCAGAATGCCCAAGCCAAACTTGTGACATTAGAAAGCTCCCACTATATCCATGACGAAAAGCCAAAGGAAATTGCCCAGCAAATAAAGAATTCTTGAAATAATGAATCTTGAAAAGTATTTAAAATTCAGTTAGACTAAAATTCCTATAAAACATACATCCTTGAAAAAGGCTGAGACATTCAT

>c129_g1

TGTCGGAGTAACAGCTGTCACAGTTGCTTGGTAATTGGCCTTGGCTACTGTACCATTAACATCGGCACGGCTGAGTTCAACTCCTGGTGTCTTACCGACAAACGATTTGACTGGTGTGAAGGTAACATTACCATCCGCATCAACCTCGAACTTACCGACTTTGGCAATTTCTTTGACGTTCGTGCCAGTATCAAATACCGGTGTGCTGTCGGTTGGGAAGCCGACTTGCGGACTTCCTGCTTCAAAAGTAACCTTGCCTTTTTGAACTTGGCCTTGAAGCCCCTCTGTCTGAGCACCTGTCGCTCTTGGAGTCACTTTTTCAACTGTTGGCGTGTACTTAGCCGTCACTGGTGTACCATTCTTATCCACTCGCTTAACAACAACTGGATCTGGCGTCCCAACATATTGCTTATCTGGGGTAAAAGTAACAGAACCGTCTGGTGCCACTTCAAAGATACCGACATTTGGAACAACCTTGCGCTTGCTTCCGTCATCGAAAGTAGCCGGAGTATCATTATCCAGTTGTACTCCCGGAGTGCCGCTCACGAAGTTTGGCTTGCCTGTTTGTGGACGGCCTTGCTCACCAGTGCTGGTGATATTTGTTCCCGTTGGAACCACTTCTTTCACCACTGCTTGGTATTTGACGGTAACAGGAGTGCCATTCACATCTGCTCGAACCAATGTCAGCTCAGGAGTGATTCCCTTAAACTGCTTCTCTGGCGTGAAAGTAACCTTACCATTGGCATCCACTTCAAATTTACCAACGGTTGGAACCTCTTTAACAGTCAAACCATTGTCAAAGAGCGGAGTTGTTTCAGCTGGAAATGGTACAGAGGCATGACCAGGCGTGAAGGTGACATGACCTTCTTGAACCTGACCTTGAAATCCTTCTGTCTTATCACCAGTACCAGTTGGAGTCACCTTGGTAAACTCTGGACTGTAAGTTGCAGTAACCGGAGTGCCGTTCTTATCGACACGTTTTACAGTGATTGGATCT

>c129_g2

ATCTGCAAAGGTTGGCTCAACTGTTTCATCAATTGGAACTAGTGGGTCCGCAGCTTTGAAGATTGGAGTTCCTGTTTGCGGGATACCTTGTGGACCGGTGCTCGTAACGGTGGTAGCTGTTGGAGTTACTTCTTTCACTACCGCTTGGTATTTAACTGTTACTGGTGTTCCGTTGGCATCCACACGAATAATTCCTAATTCTGGTGTTTCACCCTTAAACTGTTTGTCTGGAGTGAAGGTCACAATGCCATCTGCATCCACTTCGAACTTACCGATATTTGGTACTTCTTTAATATTTTTTCCGTTACCAAATAGCGGAGTCGATCCTGCTGGGAATGGAACTAATTCATGGCCAGGCGTGAAGGTGACATGACCTTCTTGAACCTGACCTTGAAATCCTTCTGTCTTATCACCAGTACC

>c131_g2

TTCTCCTCTAAGTCTAACATAATTTCTCCAGTTAAGGAAAATTTGAGCCGCATTTGAGATATTTTAAATCGGCGAAATTGTAATATTTCTATAAAAGCCTTATCCTCTCTATGGAGAGGTTTATGGTCGTTTTGCACTTGTTTGTGAAATCTTTTATTAATTCAAATGTAAAATATACTTTGAGAATTTTCACAATCTTTGGTATAATATAATTTATGAATAAACTTACTGTTACTCAACAGGCAGAAGAAAAACTGAGAAAGGGCATTCTTTTATTAGATAAAAAGGATTTTGGTGCTCTTTCGCTTCAGAATCAATGTGTGGAGCTGCAAAACCGTCAGGGAAAGTTTTTAGGCACAGCTTATCTCTCTCCGCAGAATAAGGGAGTAGGCTGGCTGATTTCCCAGAAGAAGGTAGAGCTGAACTCGGATTTTTTTCAAGGTCTCTTTGAGCAGGCTAAGAGCAGACGTCCTACTTATTTCCATTCGTCTGACACGACAGCCTTTCGACTCTTTAATCAAGAGGGGGATGGCTTTGGTGGCTTCACGGTGGATTTCTACAATGACTTTGCGCTTTTTTCTTGGTACAATGACTTTGTCTTTGCTATCAAGGAGTGGATTCTGACAGCCTTCGCTAAGGTTTTCCCAGAGATTCAGGGAGCTTATGAAAAGATTCGTTTCAAGGGTTTGGACTATGAATCGGCCCATCTCTACGGAGAGGAAGCTCCAGAAACCTTCACAGTCTTGGAGAACGGTGTTCGCTATCAGGTTTTTCTGAATGACGGTCTCATGACCGGTATCTTTCTGGATCAGCACGATGTGCGTGCCAGTCTGGTAGATGGTCTGGCTGCTGGTAAAAGTTTACTCAATATGTTTTCCTATACAGCTGCTTTTTCAGTAGCAGCAGCCATGGGCGGTGCCAGTCAGACAGTATCAGTTGATTTGGCCAAGCGAAGCCGTGAGCTGTCAGAGGCTCATTTTCTGGCCAATGGTCTGACACTGGATCAGCATCGCTTTCAGGTGATGGATGTCTTTGATTATTTTAAATACGCTAAGCGCCACAATCTAAGCTTTGATATCATTGTGATAGATCCGCCTAGCTTTGCCAGAAATAAAAAGCGGACATTCTCAGTTGCTAAAGATTATCATCGTTTGGTTGCTCAGGCTTTGGAAATTCTGAATTCTCAGGGAATCATAATTTTAAGTACCAATGCAGCCAATCTTTCCAAAAGTAAGTTTAAACAAGAAATTGAAAAAGGCCTTGCCGGCAGAAAGCACCGCTATCTTGCGGAGTATGGCTTGCCAGCAGACTTTGCCTATAATAAAAAAGACGGGAGCAGTAATTACCTCAAGGTATTTACAATAAAGGTGGACCAATGAAATTAGTCGTTTCTGTAATGCCCAAGAGTTTAGAAGAAGCTCAAGAAATTGATGTATCACGCTATGAAGAAGCGGATATTATTGAATGGCGGGCAGATTTTCTGGCCAAGGATGACATTTTAAATGTTGCGCCAGCTATTTTTGAAAAATTTGCCGGACGTGAGTTGATTTTCACGCTACGAACCCGTCAGGAAGGCGGAGAAATCGAACTATCCGATGATGAATACGTAGCTCTCATCAAGGAAGTGGCTGGTTTTTACCAGCCGGATTATATCGACTTTGAATATTTCTCTCACAAGGGGAAATTTGAAGAAATGCTGGAGTTTCCTAATCTGGTGCTGAGCTACCATAACTTCGAGGAAACGCCTGAAAATATGATGGAAATCCTGTCTGAGCTGACTTCTCTGACTCCTAAGGTGGTCAAGGTTTCTGTCATGGCTCACAATGAGCAGGATGTGCTGGACTTGATGAACTATACTCGCGGTTTCAAGACTTTGAATCCTGAGCAGGATTTTGTCACCATTTCCATGGGAAAAGTTGGCAGGATTTCACGCATTGCGGCTGATTTGACAGGTTCCAGCTGGTCATTTGCCAGTCAGGATATGGCGTCAGCACCCGGTCAGATTTCTCTGAGCAATATGAAGAAAATCCAGGAGATTTTGAATGAGAATTAATGGCCACACCCGCATGGCTGCTGTGGTTGCCAAGCCAATTAAGCACAGTATTTCTCCTCTTATTCACAATATGGCTTTTGAAAAGACCGGTGTCAATGGTGTCTATCTAGCTTGGGAAGTAGAAGCAAAGGATCTGCAGGCCAGTATAGAAAATATCCGCAGATACGATATGTTTGGTGTCAATCTTTCCATGCCCTATAAGCAGGAAGTGATACCTTATCTGGACGAGCTGGATGTCAGCGCCCGCCTTATCGGAGCGGTCAATACAGTTGTGAATAAAAATGGAATTTTAGTTGGTTATAACACAGATGGCAAGGGATTTTTTAAGAGCTTGCCTTCTTTTGCTATTCGGGGCAAAAAAATGACGATTTTGGGGGCAGGTGGAGCAGCAACAGCTATTATTGCTCAAGCAGCTTTGTACAATGCAGAGGAAATTTTCGTTTTTACTCGGCAAGCTTCCTATGAGAAGATTGTCAGCAAAATGGCAGCTATCAGCCACCAAACCAAGAGTCGTATCCAGGTACTAACCTTGGAAGATGCGGATAGCTTGCAGGATAAAATCAATCAATCAGACCTTCTGGTCAATGGGACCAGTCTAGGCATGGATGGGGTCAGCATGCCCCTGCCTGAACAGCTTGAGCTGCCCAGCCAGATTTTAGTTGCAGATGTTATCTACCAACCTTTCGAAACTCCCTTTCTCAAATGGGCCAGAAATCAAAATGTCACAGCGGTCAATGGACTTGGTATGCTGCTCTATCAGGGAGCAGAAGCCTTTGAGCTTTGGACCGGCAAGCCTATGCCCAGTCAGGAAATTTGGCAATGTTTAGAAGAATTGTATAAATAAGGAGGCGCTTATGAAACTGAATGTGAATCTCCCGCATCATCCCTATGATATTCTCATCAAAAAGGGGTCTTTATCTCAGGCTGGAAGTTGGCTGAGTCAGCTTTGGCAGCCTCAGAGGGTAGTTATCGTCACGGACAATCGAGTGGCTCGACTCTATGCGGAAAAGGTCAAGCTGAGCTTGGAAGCGGCTGGGTTTGAGACTTTTGTCTTTGACTTTTTGGAGGGTGAAGCTAGCAAGAACTTAAAGACCGTCAACAAAGTCTATGAGTTTTTGGTCAAGGTTGGTCTGACCCGCAGTGACGGCATTGTAGCCTTGGGAGGCGGAGTCGTCGGTGATTTGGCGGGCTTTGCCGCTTCGACCTACATGCGGGGTGTGCATTTCGTGCAGATTCCGACCAGTCTGACAGCCCAAGTGGACTCCTCTATTGGTGGTAAGACAGGTGTCAATACGCCTTGGGCCAAGAATATGGTCGGAACTTTTACCCAGCCTGACGGAGTTCTGATTGACCCTGAAGTCTTGCATACTTTGGGTCAGCGGGAACTAATCGAAGGCATGGGCGAGGTGGTCAAGTACGGCTTGATTGAGGATAAGGAACTCTGGGATGAGCTGTCAGAAATGGATGGCAGTCCAGAGTCGATTTTGGAGCATGCGGAGAGCATCATCTACCATTCCTGCGATGTCAAGCGTAAGATTGTGGTCGAGGATGAGCTGGATAATGGCGTCCGCCTCTATCTGAATTTCGGCCATACTATTGGGCATGCTATCGAAGCGACAGCGGGCTATGGAAAAGTCATGCACGGTGAAGCCGTGGCGATTGGCATGGTACAGGTTTCCCGTGTCGCTGAAAAGAAAGGCCTCATGCCAGCCGGAATCACAGAAAACATCATCCATATGTGTCAGAAGTTTGGCTTGCCGGTGGATTACCAGCCTTGGAATGAAGCTGCTCTTTATCAGGCCTTGACCCATGATAAGAAGGCTCGAGGGAATTCTATTAAACTAGTACTTGTTCCTGAGTTGGGTTCGGCTAGTATTCACCAGATTCCGCTGGAAGAGATGAAAGAATTTCTGAAGAAATAAGTGCCAGATGGAGTTTAAGAAGATTGGATAGTTTGGATGAAAAATAGACAGAGTGTCCAGCTCTTTCCATCCCAGAAATAGGAGAAAATGTATGAGATACTTAACAGCAGGAGAATCGCACGGACCACGTCTGACAGCTATTATTGAGGGAGTGCCAGCTGGTCTTCCTTTGACCGCTGATTATATCAATGCTGAGCTCAAGCGTCGTCAGGGTGGGTACGGACGCGGTGCCCGTATGAAGATTGAAAGCGACCAAGTTGAGATTACGTCTGGTGTTCGGCATGGATTGACCATGGGCGGCCCGATTACCCTTAATGTCACGAATCTGGATCATCAGAAATGGCAGGAGATTATGAGTGCGGCTGATGTGGATGAAAAGAAAAAAGGGCTGCGCAAGATTACCAAACCACGCCCTGGCCATGCAGACTTGGTTGGTGGTATGAAATACCGCTTTGACGATTTGCGAAATTCACTTGAGCGTTCTTCTGCCCGCGAAACGACCATGCGTGTAGCAGTCGGAGCAGTAGCCAAGCGTTTGCTGGAAGAAATCGGTGTAGAGGTGGCTAGTCATATCGTAACCTTTGGTGGCATTGATATCAATGTACCGAACAATTTGACTGTAGGAGAAATCAAGGAAAGAGCTGCCCAGTCAGAGGTTTCCATTGTCAATCCCGATCGCGAAGAAGAAATCAAGGCCTACATTGACCAGATTAAGAAAGACGGGGATACCATAGGTGGTGTCATTGAGACTGTCGTTGGTGGTGTGCCGGTTGGTCTGGGCTCCTATGTCCAATGGGACAAGAAGCTGGATGCTAAGATTGCTCAGGGAGTCGTGTCCATCAATGCTTTCAAGGGAGTTGAATTTGGTGTGGGCTTTGAAGCCGGTCGTCTCAAGGGCAGTCAAGTCATGGATGAAATCCTCTGGTCTGAGGAAGATGGCTTCACTCGCAGGACCAATAATCTAGGCGGCTTTGAGGGCGGTATGACCAATGGTCAGCCAATCGTGGTTCGTGGTGTCATGAAGCCCATTCCAACCCTTTACAAGCCACTGATGAGTGTGGATATTGAGACCCACGAGCCTTATAAGGCGACAGTGGAGCGGAGCGATCCAACGGCTTTGCCAGCGGCGGGTGTTGTCATGGAAAGCGTGGTGGCAACAGTTTTAGCCACCGAAGTTCTGGAGAAGTTCTCTTCGGACAATCTGGAAGAACTAAAGGATGCGGTAGCCCGCCATCGGGAATTTGTCAAGAACTTTTAGAAGAAAGGAGTGGGCATGGAGAGAAAAACAGTCTATATCGCAGGTCTGGGACTGATTGGAGCTTCTTTGGCTCTGGGCATCAGGCGAGCTCATCCTGAGATAGAGATTCTTGGCTATAATCGAAGTGAGGAATCGCGCAGAGTTGCTCTGGAACGTGGAATGGTAGACCTGGTGACAGATGATTTTGCTGCCTTTGCTCCATTGGCTGATGTGATTATTCTGGCTGTGCCCATCAAGCAGACCATAGCATTTATCAAGGAGCTAGCAGATCTGGAACTGAAGGAAAATGTTATCATTTCTGATGCTGGATCAACCAAGGCTGAGATTGTGGCAGCTGCAGAAAAGTATCTGCAGAATAAGCCCGTCCGCTTTGTCGGAGCTCACCCGATGGCCGGAAGTCACAAGACCGGAGCCAAAGCGGCCCATGTCACTCTCTTTGAAAATGCCTATTATATTTTCACGCCCTCTAATCTGACCAAGCCTGGTACGCTTGAGGAAATGAAAGACTTGCTAAGTGGTCTTCATGCCCGCTTTATCCAGGTGGATGCAGCTGAGCACGACCGAGTGACCAGTCAAATCAGCCATTTTCCGCATATCCTAGCCTCCAGCCTGATGGAGCAAGCAGCAACTTATAGCCAAGAGCATGAACTGACTCAGTCTTTTGCAGCCGGTGGTTTTCGAGATATGACGCGGATTGCGGAGAGTGAGCCAGGGATGTGGACCTCTATCCTCTTGACCAATCCTCAAGCTATCTTGGAGCGGCTGGAAGATTTTAAAGACCAGTTAGACAAGGTGGCCGCAGCGATTGAGGCTAAGGACGAGACAGCTATTTGGGAATTTTTCGACCGAGGACGGCAGAGTCGCAAGCAGATGGAAATTCATAAGCGTGCCGGTGTGGATAGCTTTTATGACCTCTTTATTGAGGTGCCCGACGAAGAAGATGCGATTTTGGGTATTTTGGAGCTCCTGCGGGGAATTTCAGTCGTCAACATTCGTATCAACGAGGAAAACCGAGAAGATATCAATGGTATTCTGCAAATCACCTTTAAAAATCACCAAGATTTGGAAAAATCTGCTAAAATAGTAAGAGAAAATACGGATTACCTAGTGTCCGTAGACTAATATAGATTGGAGAACTCTATGTCAAATATTTATGATTTAGCCAATGAACTGAGCAGAAATCTGCGTGAGTTGCCTGAGTACAAGGCCGTCGCAGAAAGCAAGAAAGCAGTGGATGCTGATAGCGAAGCGAAGGCTATTTTTACAGATTATCTGGCTTTCCAGCAGGAGTTGCAACAGCTGGCTCAGACTGGTCAGGTACCGACTCAGGAGGTGCAGGACAAGATGACTTCCTTTGGCGAGAAGATTCAGGGCAATGCTGTTCTTTCTGAATTCTTCAATAAGCAGCAGCAATTGTCCATCTATCTGGCGGACATTGAGCGTATCATCTTTGATCCGGTGCAGGATTTGCTTAAGTAAGCTAATAAAAAGAAGAATCTGGGCTGAATACCCGGATTCTTTTTGCTTTAGCTAGGATTCCAGAAATTTTAAGAATTTCTGGTCTTTTTATGATAAAATAAGAAACAACAAAGGAAAGTACGAGGTCACCTATGAAATTATCAACCAATGTAAAAGGCCTGAAGGGCCGTATCCGCGTACCTGGAGATAAGTCTATCAGTCACCGTTCCATTATTTTTGGCAGTTTGGCTAAGGGAGTCACTACTGTTCGCGACATTCTGCGGGGAGAGGATGTGCTGTCTACCATGCAGGTTTTTCGTGACTTAGGCGTGCAGATTGAGGACGATGGAAATCTAGTCAAGATTCATGGCGTAGGATTTGAAGGGCTGCAAGCACCGAAAAATAAGCTGGATATGGGCAATTCTGGAACGTCTATCCGTTTGATTTCTGGTGTTTTAGCTGGTCAGGATTTCGAAGCGGAGATGTTTGGTGACGACAGTCTATCCAAGCGACCTATGGATCGGGTGACCATCCCTCTGCGGCAGATGGGAGTCGAGATTGCTGGTAGGACTGAGCGTGATTTACCACCGCTCAAGATGAAGGGCAGCAGGGAACTGCAGCCTATCCACTATCAGCTGCCAGTGGCTTCTGCTCAGGTTAAGTCGGCCTTGATTTTTGCTGCCCTGCAGGCTCAGGGAGAGTCGGTTATTATAGAAAAGGAAATCACCCGTAATCATACAGAAGATATGATTGCCCAGTTTGGCGGGCAAATTGAGGTTGAGGGTAAGGAAATTCGCATTCAGGGCGGTCAGGAATTCACTGCTCAGGAAGTGACGGTTCCAGGAGACATTTCCAGCGCGGCCTTCTGGCTGGTAGCTGGATTGATTGTGCCGGACTCTAAGATTGTTTTAGAGAATGTCGGCATCAATGAAACTCGTACAGGTATTCTAGAAGTGATAGAGGCTATGGGAGGACGGATGACGCTGTCAGATGTCGATCCAGTAGCCAAGTCTGCAACCATCACCGTTGAGACTTCTGAGCTTAAGGGTACGGAGATTGGCGGCGAGATTATCCCGCGCTTGATCGATGAGCTGCCGATTATTGCTCTTCTTGCGACGCAGGCTCAGGGACGGACTGTCATTCGTGATGCAGAGGAGCTCAAAGTCAAGGAAACCGACCGGATCCAGGTAGTAGCAGACGCTCTCAATAGCATGGGCGCAGCTATTACCCCGACGGAAGACGGCATGATTATCGAAGGGAAAACACCTCTTCATGGCGCCCAGGTCAACACTTTAGGTGACCATCGCATCGGGATGATGACGGCAATTGCTGCTTTGTTGGCTCAAAGCAGTCAGGTGGAACTTGAACGATCTGAAGCTATTAAGACTAGCTATCCAAACTTCTTCAACGACTTGGAGGGTTTGATGCATGGCTAAGATATTGCTGGGCTTCATGGGAGCCGGAAAATCCACAGTGGCCAGAGCCTTAGCCCAAGACTTTGTCGATATGGATGAACTGCTCTGCCAGCGGTTGGGCATGTCTATCAAGGACTATTTTGACCAGCATGGTGAAGCAGCCTTTCGGACTGCAGAAGCCCAGCTCTTGGCTGAGTTGATCGATACGGACTTGGTCGTCTCAACCGGCGGCGGTGTAGTCGTCAGCTCAGAAAACCGTAGATTACTGGCTCAGAATGCTGATAATATCTACCTGAAAGCTGATTTTGAAACGCTTTATCAGCGAATCCAGCAGGATGCTGAGCAGGAGCGGCCCTTGTTCTTAAACCAGAGCAAGTCAGACTTGCAGCAGATTTTTGAGCAGCGTCAGGCTTGGTATGAGGAAGTGGCGACCCAGATTGTCGATACGAGGCTTAAAAGCCCGCAGGAAATTATTGAGGAAATTCAATGAAAATAGCATTTTTAGGTCCCAGAGGCTCCTTTTCTCACCATGTAGCCCAAGCTGCCTTTCCCAGCCAGGACTTGGTGCCCTATCAGAATATCACCGAGGTCATGAAGGCTTATGAGGCTAGGGAAGTGGATTACTCGGTCGTTCCAGTGGAAAATTCCATTGAGGGCAGTGTCCACGAGACCTTGGACTATCTCTTTCATCAAGCAGACATTCAGGCAGTGGCAGAAATCGTTCAGCCCATTAAGCAGCAGCTCTTGGTGACGGATTTAGAAAAGCCAATCGAGAAAATATTTTCTCACCCGCAGGCTATTGCTCAAGGGAAAAAATACATCCGCCAGCATTATCCGCAGGCTGCCATTGAGGTGACAGCTAGTACAGCCTATGCAGCTCGTTTTGTGGCAGAGCATCCTGAAAAGAATTTTGCGGCTATTGCCCCGCGCACGGCTGCAGCAGAGTACGGGCTTAAAGTAGCAGCCAGCGACATTCAGGAAATGGAAGAAAATTATACCCGCTTCTGGATTCTAGGCCATGAGGTGCCTGAGCTTGAACTGGCTAAGACAGGAGACAAGCAGACATTGGCCTTGACCTTGCCGGACAATCTGCCTGGTGCCCTCTATAAGGCTTTGTCGACCTTTGCTTGGCGTGGGATTGACTTGACCAAGATTGAGAGCCGGCCTCTAAAAACGGCTTTGGGAGAGTATTTCTTCATTATTGACATTGACAACGAGCAGAAAAAATTGGCGGATTTTGCCTATCAAGAGTTGACAAGTCTTGGAATTACTTATAAAATTTTTGGTAGCTACAGTGTGTTTCTGATTCAGGACAAGTAGCAGTAGATTGGAGAAGAGATGAAGAAACCAGAAAATCTTAGCCATCATGAACAGCTCCGCTTAGATTATCTCTATAAAAATTATTATTATCTAAACGATAAAGAAAAGAAAGAATTTGACTATCTGCGTCAGAAGTCCAAGGGGATTGGCAGTTCAGCCAGTGCTCCTGACTATGAAGAGCAGCCGCATACAGTTAGTGACGCCTACGAGCAAGAGCCTGTGGAAATGGATTCTTCTGGCCTCTTGCCCAAGTACCCTGAGCGTTCTTCTCGCAGCAGAAAGCAAAAGAAGGAGCCTGAAGCTCTGGCAGAAGCTGGTCTTGGACAAGACAAGCCCAAGAAGCCTCGCAAAAAAATCCGCTTGAAACGGATTCTGCTTTGGGCAGGGATTTTCCTGCTGATGGTCTTGGGCGGCATGATTTTCATGTTTGTCAAAGGCTTGACCACAGCTCATAATGGCAATTCTAAGCCAGCAGAAACGGAATTTTTTGACGGCAAGGATACTAAGGACGGAGTGAATATCCTCATTCTTGGAACAGATGGCCGGGTTGGTGACGACTCGACTGAGACGCGGACTGATTCCATTATGGTCTTGAACGTCGGAAATAAGGACCACAAGGTGAAATTAGTCAGCTTTATGCGTGATACTCTCATTCACATCGATGGTGTCAGCAATGAGTACACCGATCCGTCGCAGGCAGATTACTATGACCAAAAGCTCAATTCCGCCTATACGATTGGGGAGCAAAACCACAACCGTGGTGCAGACTATGTCCGCCAGATGCTCAAAGATAATTTCGATTTGGACATCAAATATTATGCACTGGTGGATTTCCAAACCTTTGCGACAGCTATTGACACCCTTTTCCCAAATGGCGTCAGCATGAATGCTCAGTTCTCAACGATTGATGGGGAGAAGGTGACTGAAGTTGAAGTGCCAGACGACCTGAATATGAAAGACGGTGTGGTGCCTAACCAGACAATCAAGGTCGGTCAGCAGCAGATGGACGGTCGGACCTTGCTCAACTACGCTCGCTTCCGTAAGGATGATGAGGGCGACTTTGGCCGGACCCGCCGCCAGCAGGAAGTTTTGACGGCTGTCTTCCAACAGGTCAAGGATCCGACTAAGCTTTTTACTGGATCAGAGGCTCTTGGTAAGGTCTTTGCCTTGACTTCGACCAATGTGCCTTACAGCTTCCTTTTGACCAATGGTCTCTCTATGGCTGGAGATTCCCGCAACGGGGTTGAGCGCCTGACGATTCCAGAAAATGGCGACTGGGTTGATACCTATGACATGTACGGTGGCCAAGGGCTCTTGATTGACTTTGAAGCCTATAAAGAAAGACTGCAGCAAATGGGTCTCAG

>c132_g1

CCTCATGATGCAGGTGGAAAATGAATACGGGTCTTATGCCGAGGACAAGGCCTACATGCGCAGCATTGCACAGATGATGAAGGTACGTGGCGTTACGGTTCCCCTCTTTACCTCTGACGGGACATGGATAGAGGCTTTGGAGTCTGGAACTTTGATTGAGGATGATATTTTTGTGACGGGGAATTTTGGCTCCCAACCCAAAGAAAATACAGACAATCTACGAGCTTTCATGGAGCGCTATGGCAAGAAATGGCCTCTTATGTGTACTGAGTTTTGGGACGGCTGGTTCAGCCGCTGGAGCGAGGAAATCGTTCGGCGAGAGGCAGAAGATTTGGCCCAGGATGTCAAAGAAATGCTGCAGCTGGGCAGCATGAACCTTTTCCTATTGAGAGGTGGGACCAATTTTGGTTTTATCAGCGGCTGTTCAGCCCGCAAGACAAAGGATTTGCCACAGATTACCTCCTATGATTTTGACGCACCCATTACTGAGTGGGGGCAGCCAACCGAGAAATACTACGCTGTTCAGCGGGTGACACACGAGGTCTTTCCAGAGCTTGAGCAGATGGAGCCGATTAGCCGACAGGCCAAGGCCTATGGCAGTTTTCCGCTGCTTGGGACTGCAAATTTGCTGGATGTTGCAGCGGACATCACAGAGGAAATTCTGCTGGATTATCCGCAGCCTATGGAGCAGATTGGGCAGAATTATGGCTATATCCTCTACCGTTCCGATATCAAAAATCAATACCATGAGGAAAGGCTCAAGGCCTTGGAGACTCATGACCGCTGCCATTTCTATGTCAATCAGGAGCACCTAACTACCCAGTATCGGGAAGAAATCGGTGATGAGATGCTCTTCTCAGCTGACACCGAGCGAATTCAGATTGATGTGCTGGTGGAAAATATGGGGCGTGTAAACTATGGTTACAAGCTGGGCGCACCTAGTCAATCCAAGGGAGTCAAGGGGGGTATCATGATTAACCACCAGTTCCGCAAGGGCTGGAAGCATTATGCGCTTAAGTTTGACCAGGAAATGCTGGCCAAGTTAGACTGCTACTCAGATCCACCGGAAAAAGTCAAAGCACCGACCTTCTATCGCTTTGAAGCAGAGTTGGATGATATCGCTGATACCTTCATCGACTGCTCTAAGTATGGGAAGGGCTGTATTTCGGTCAACGGCTTCAACCTAGGCCGCTACTGGAATGAAGGGCCGATTCACTATCTCTATGTGCCGTCTAGCCTGCTTAAGGAAAAGAATGAATTTATCGTCTTTGAAACTGAAAATGTCAAGATTGAAGAGTTGACCTTGCTTGACCATCCAGTCTATAAGAAATGAGGTGTTGCGATGAAATATATGACAGTCGGAAATGAACTGGAAGGAGCCAGTCAGCTGGTTTTGGGCTGCATGCGACTGGCAGAGCATGATCCTAAAGAGGTCGTTTCCGTTCTTGAAACAGCATTAGAAGTGGGAATTAATTTCTTTGACCACTCAGATGTCTATGCTGGCGGCCAGTCTGAAGCCAAGTTTGCACAGGCTCTTCGCTCGGCTAAGATTCCCCGGGAACGAGTTCTCATCCAGTCCAAGTGCGGACTGCGGGATGTTCACACCAACTACCATTTTGACTTTTCTAAGGATTATATCATCAGCTCGGTTGAAGGCAGCTTGGAGCGCTTGCAGACGGACTATCTGGATGTGCTCCTCCTGCACCGGCCAGATGCTCTTGTGGAGCCAGAAGAGGTGGCTGAGGCTTTCTCACAGCTGCACCAAGCCGGGAAGGTCCGCTATTTCGGAGTCAGCAATCAAAACCCTTATCAAATGGAGCTGCTTCAGAAGAGTGTGGAGCAGCCCTTGATTGCCAACCAACTGCAGTTTGGTCCAGCCCATACTCCGATGCTGGACGCTGGGCTCAATGCCAATATGCTCAATCCTTTGGCAATCGTTCGGGATGGTAGTGTACTGGACTATTGTCGATTGCATCATATCACGATTCAGCCTTGGTCACCATTTCAGGTCGACTTGAACCAGGGACTTTTTATGGAGCATCCTAAATATGCTCAGCTTACAGAAACTCTGCATGCCTTTGCATCAGACTATAAAGTTTCTTTTGAAGCCTTAGTTCTCGCTTGGATTTTGCGTCATCCGGCACAAATGCAGCCGATTGTTGGCTCCATGAATCCACAGCGAATTCGCTCAATGGTGGCTGCTTTTGATATTGAACTCTCTAGGGCTGATTGGTATAAGATTTACAAGAGCGCGGGGAATCCTCTGCCCTAAGCTTGTTTTTGCAGGGAGATTATTTGTTATTCAGAATCAATGAAGGTTAAGAGCAGTGAATTAGTCTGCTTGCTTTATTGATTAAGCTCGACTCTGCACCTTATGAAAGGAGAATGATATGAATAATAAGTTTTCTTGGCAAGCCCTAGCAAAATCTACTTCTGCTGCTAAGTATGGGAGCAGCTTGCTGCTATGTGCTTCCCTCTTGTCCTTAGCAGCTTTGACAGCTGTCTCAAGAGTTGAAGCAGAAGATGTTGCGCCTGCCCCTGCCGCAGTAGCTGAAACGCCGGCTTCTCCAGCAGCGCAAGCAAGCCCCGAGGCTGAAAATCCAGCTGTAGCTGAACCCCAAGCAGCTCCTGCAAATCAGGCCGAATCAGCTGCAGCCGCTAGCGAGTTGCCAGCCAGTCAGGTCATGTCCGATGAGCAGAAGCAGCGCGCCATTGCTCAGATGGGAGCCAATCAAGGGGATGTCTGGGTTTGGTCGGAGGAAAATGTCGTTGAAGAAAATGGGGAGCGTCAAAAGCGCGGTCCTAATGGCGGTCCTGGCCAATTTAATGGCGGAGCCGTCGTGTTAGGCGGCCGTCCGACTTATGACCCAGCGGCTGATTGGTACTACCTTGCATATAAAGGCGAAGGGATGACTGACGAAGAAGCGAAGGCTGTCTTTGACAAGGAACGTGGCAAATGGATGAATGAATTTGATCCTTGGGGCGTTGTGATGGAAATGACGGACAATAAGGCCAGCAATGCTTGGGCGGATATTCGTAACATGCAGGCTTTCGTGCTCCGGAAGGACTCCAATGTCTGGGAGAAAATTGTGGATCACCCTCAAGGAGTTCAATGGGTTTCTCAATTCAAGGGAAATATGTCCAGCAACGTAGGAGATGCACGGCAGGAAGCCTTGGCTGGCGGGGGAACAGCTATTGAGGTATTACCTAAGCAGGATCGGGTTGCCCACTGGGGCTCTGATCAGGCCCGTGATATTCCAAATCCTCAAACCATTCGGGCTGTTTTAGTTTCCGTAGAAGCTCGTATTTCTAAAAAATCTGACCCAGATGCCAAGCTTGGTATTCAAGTTGGCGGTGACTGGAAATTTGCTGAAGAAGTGAGCAAGCCGCTCTGGTATCCAGGAGCTGGTCTGGCTGGTATTCAGCGACTGACCAAGGATTGGGCTCGTTACTATTTTGTCAGCCTCACTGGTATTCAAGATGCAGTCGAAGAAAGGGCCATTTCACAAGATGTATTTATGAAGAGTATCGTGCCGCTGGCCGATATGGAGCAGGTCGCCCAAAATCAAGCCCAGCAGCAAACTCCTGCAAGCCCTGATCCAAAGAGTAGCCAAGCAGAAAATCTGACCTACAAAACAAGCGGCCAGACGGATCCTCAAGAAGCGTCTCAAGAGTCAACTCTTCAACAAGCTTCTTCCAAACAACTGCCTAAGACCGGCAGCCAAAGCAGTCTATTGACCAATCTGCTTGGACTCGGGCTCTTAGGACTGGGAGCTGGCTTCCTTGGCTTGAAGAAAAAATCTTAGTTTTGCAAGCTAGAGAAAAAAATAATCTACAAAAAAGCAACAGCTAGAATCTGCTGTTGCTTTGTGTTTGATTTAGGTTTTGCACGACAGGCTGTCCAAAATTAGGTGCTTGCGCAGCGGGCGGACTGCAGACAGGCTCTTGGTATTTTTGAGTAAAGCGGCATCTTGGGAAGTTGGAGCAGCCCCAAAATTGCTGACCAGCCCTAGAACCCTTGGATGCTGTACGGAGCACCAATCTATGCTGGCAGCGAGGGCAGATCTTTTCTGCTTCTCTACGCTGTTTATTCTGGGCAATGCTGTTAATATGGCGCTCTTTGATGACCTGCTTTCGTTTGGTCAAAGGTAGCAGCATACGATAGAGGTCGTCAATCTGCTCAGGCGACAGGTAGACTGGACTTTGGCAGGCCTGCTGGTCAATTGCCCCAATCACTTGCCAGGTCTGAATAACCTTATGCCTGTCACTGTTTAGATGGATTTCTTTAAGCTGACAGTCATTGCCAAAGACGATATAGGAATATAGATTTGGGGTGTATTGGTTGAGATAGTAGCTGAGCCATTTGAGATGGACTTTATTTTGAATAATGGGATTGAAAAAAGAATGCTTGTAAGATTTTCCGCGGCCACCGGAAAGAACCTGAGTCCAGTATTGCTGATTTTCTGAGCCGAAGATCCAGCCCTTGTAGTTTTTTGACTCAAAAATGTAAATTCCAGATGGATGAATCAGCAGGGCATCAATCTCAGTGGTAGTGCCATCTTCTTTGAAGATGTAGAGATTGTACAAAATCTTTTTATAGCCATAGAGCTGGTTCAAGACCTGACCAAGCTGGTATTCTCCCTGTTTTCCCTTGTTATATTTGAGTTCCCAAGGGGCAGTCTGGGTTTGCTGGAAATAGTCAGGTTCTTGACTTTGGCTGGACGGCCAATCCGAGTCCGTATCTTCCCTGTCTTCAAGCAGCTTATAACTGATAAAAAAGACGATGCCAAAGACAGCCACAAATGCAACGAAAAAGAATCCAAACATTTCCTAATCTCCCTTAAAATCCCTAATTCGAGTATAGCACAAAATAAAGGACGGAAGATTTTTATAAGTGTAAAAACACAGCAATTGAACAATAGATTAGAATATGTCTAAAAATAGAAAGCTATCCTTTAAAAACTTATTTTCCAAAAACACCTCACAAACCTAAAAAATCATGTATAATAAAAAGACAGGAAAAGAATAGGAGTTATCATGTCTCAGGAATTAATCAGTGTTATCATCCCTGTTTACAATGTTGAGCAGTATTTAGCAGAATGTGTGAACAGCGTCTGTCGTCAGACCTATCAAAATCTGGAAATTATTCTAGTCAATGATGGTTCAACAGATGCTTCTGGCCAGATTTGCCAAGAGCTAGCTGACCAGGATGCGCGGATTCGTCTCATTCATCAGGACAATCAAGGCTTGTCCGGTGCCCGCAACACAGGGATTGAGCACTCTAGTGCAGATTATTTGATTTTTGTGGATTCGGATGACTGGCTGCCTGAGCAGCATGTTGCCCGCCTTTATGAAAAGCTCAAGGAGTACGATGCGGATATTGCCATTGGTCACCACTGTAGCTTCCGTGCAGAGGATTCAGCCTTCCTCTACTATTCGACAGAGCATTTTGAAACCCTCTATACGCGAGAAGAGATAATCGAGGAGTACCCTAGACGGCGCATGCTAGACGGTGTCTTTCTCTGTGCCTGGGCCAAGCTCTATAAGCGTGAGCTCTTTGATACAGTGCGCTATCCAGTCGGCCGAGTGGCCGAAGATGCCTTCACAACTTATAAGCTGTATCTTCAGTCAGAGAAGATTATCTACCTCAATGAACCGCTCTATTACTATCGCTTGCGGCCAAATAGCATCTCCATGACTTGGAATGAGCAGTGGTTCCGAGACTTGATTGTAGGTTTTGAGGAGCAGCTAGCCATTTTGGGTAAGCTGGGCTATGACCTGAGCTTCTACTACAAATACTACACTTTCCTGCTCCAGTATTGTCGCGACAGCGCTGCAGCTGTCGGTATGCAGTCCAGTCGGGTCTGTCAGGAAATTGAAAGTAAACTCCAACTATTTGGAGAATAAATAAAACAAGCCACCTTTCAACCATAGGGTTGACAATGTGGCTTGTTTTTTAGTGTTTGAAAATTAAAGACTAAAAATAACAATTAATGTCATGCTTCAAAAAGACATCTTGGTATTCAAATAAGTCTTCAGGGTGCAAGGCCTTGACATCTTCCATGGCATACTTTCGGCCGAGTAATTTGTATTTATTTTCGCCAAATTGATGGAAAGGCAGCAGCTGGACTTGATTGATGGATAGTTCATTAAACAAAGTCGCAAACCGCTCTGCATCTTCTAAGGAGTCGTTAAAATCTGGAATGACTGGAATGCGCAGAACAATCGTCTTTTGATGAGTAAAGGCGTAGTGGATATTCTGAACAATTAATTCGTTTTTTACTCCAGTCACCTTGCGGTGGTTGACAGAGTTATAATGTTTCAGGTCTGTATAGATGAAATCAACGTACTGGATGAGATCCACGAATTTTTCATGTTCTACAAAGGCAGTTGTTTCAATAGCGGTATGAATCCCTTTTTCCTTAGCCGCTTTGAGAATGGCCTTGGCAAATTCAAACTGGGCGAAAATCTCTCCGCCAGATAAGGTTAGGCCACCGCCAGATTCTTCATAAAAATCTCTGTCTTTTAGGACTTCGTTGATAATCTCCTCGACACTTCTTTCCTCCCCCATAGTGATGCTTTTTTTGGTAGTAGCATCCAGCATAGGCTCGGGTCTGAATTGCTGTGATTCTGGATTGGAGCACCAAGGGCAGCGTAGAGGACAGCCCTTGAGGAAAACCGTTGTACGAATCCCTGGTCCATCATGAATACTGAAATGTTGGATGTTGAAAATAATTCCTTTTGTTGTTTCCATACTTAGGCCTCCTTTGGATACAATCATTATAACTTATTTTAGAAAGAAAAACAATTACGAATGAAAGTTATTTTTATTTTATTTTTAAGACGTCTTTTGTTACAATAATAGCAGAGGTGATAGAATGGAACGTTTAGATGAAATTGTTAAATTAGTATCTGAATTTGAACGAATCGATGTCAATACTTTGTCTGACCGCTTAAAAGTGTCCAAAGTAACGATTCGCAAAGACTTGGATAAGCTGGAAACCAAAGGTCTGTTGCGCAGAGAGCATGGCTATGCTGTTTTAAATAGCGGCGATGATCTGAATGTCAGGCTTTCCTTTCATTATGATACCAAGCGCCGAATTGCCCAGGAAGCAGCAAAAATTGTTCAGGATAATGAAACCATTATGATCGAATCAGGCTCGACCTGCGCCTTGCTAGCTGAGGAAATCTGCCGAACGAAGAAGAATGTCAAGATTATTACCAATTCTTATTTTATTGCGGATTATATAAAACAAACTGATTCTTGTAAAATTATCTTACTGGGCGGAGAATTTCAGAAAGATTCTCAAGTGACAGTAGGGCCTCTTCTCAAGGAAATGATTCGGTTTTTCCACGTAGAACATGCTTTTGTCGGAACAGATGGCTATGATGAAAATCTAGGCTTTACAGGTAAGGACCTGATGCGGAGTGAGGTTGTGCAGTATATGTCAGAGGCATCTGACCAGATGATTGTCTTGACGGACTCAAGTAAGTTTACCAGAAAAGGAATAGTTAAGAGATTCGGCTTCAAGCAGATTGCTCAAGTGGTGACTGACAAGGCGATTCCTAAAGAGGCTGTTGAGCGCTTGAAAGCTGCCGATATCAAGCTGACTCTGATCTAGCAGAGAAGGAGATAAGATGAAAAATGAAAGAAAAAAACTATTGGCAAAAATTGCTTATCTCTACTATATCGAAGAAAGAAGCCAGTCTGACATTGCGGCTGAGACAGGGATTTATCGCACCACCATCAGCCGAATGCTGGCAGAAGCTAAAAAAGAAGGTATTGTAAAGATTGAGATTGAAGACTTTGATACCCGCTTATTTCATTTGGAAAACTATGTAAAAGAAAAATATGGGCTCAAGGGAATCGAAATTGTCAGTAACTTAGTAGATGAATCTCCAGCAGACTTGGAAGAGAGGTTGGCTCAAGCTGCTGCGGGTATGCTGCGCGGCCTGATTAAAGACAATGATAAAGTGGGATTTTCTTGGGGAAAGAGCTTAAGCCTTTTAGTAGAGCACTCCAGCAGCAAGCATTTGACAAATGTTCATTTCTTTCCTTTGGCAGGTGGTCCCAGTCATATCCATGCACGCTACCATGTGAACACCCTCATCTATAGCATGGCTGGAAAATACCATGGGGACTGCCGTTTTATGAATTCCACGATTATTCAGGAAGATGAGCAACTAGCAAATGGAATTTTAGATTCTAAATATTTTGAAGATCTAAAAAGCAGCTGGCAGGAGCTAGATGTGGCTGTGGTTGGTATTGGCGGGCAGGTTGACACGAGAAATCGTCAATGGCTGGACATGCTGACTTCAGAGGATTTTCTTGCTCTAGAGAGTCAGGATGCTGTCGGAGAAATCTGCTGTCGCTTCTTTAATAAAAAAGGGGATATGGTCTACCAGCATTTGCAAAATCGAACGATTGCCATCTCTTTGGAAAATTTGAAAAAGGTGCCGCTTAGTCTGGCCTTTGCCTATGGCAGTCAAAAATCTGCTGCTATCTTAGCGGTGTTGCGAGCTGGCTATGTCAATCATTTGGTGACAGATGAAGCGACGATTCTAAAAATGCTTGACTTGGATGGAGATAGGAGTTTCTTCACTTCTTGATAGTAGCGGCATCTCAGGTGCAAGAGATTTCCAAGACAGAATTTAGGGATTCTATTTTTTAATAGTGGATTAGCAGCTATGTATGGAGGTGGATTGTTGATTTGAAAAAGCCGATGAAACAAAAATTGACTAGTCTAGCCACAGGAGAGCTAGTCGCTGTGCTAGTATTTTGGATGAATTTTTTCCTGCTCAAAAAATGGATTCTTACTACTGGGGCTTTGATTTCTATTTCTTTCTCCTTGTTTGTACTGAGTTTTATTCTGATACAGGGATCTGTTTTCTGGTGGATTTTGATAAAAAGGATTTCCAATCCAAGATTTGCTGAAAGATATACAGGCAAGATTTACAGGGTACTAAAGATTTTAGACCTTATTTTACTGGGTGTGGGAACCCTGATAATCATTTTTAATTCCAGTGATTTTTCCACCTTCATAATCTCTGTAGCAATTTGGTTTTTTGCTGTCATCGAGTGGATTAATTATTTTAAATGGCAATTATCCTACAGTCTGAATCCTGCTGTTTTATTGAAATACAACCTCCAGAGGAAATTGCGGAAAAGTAAAATAGCAAAGGAAATTGAGAAGGCCGTATAAAAAGATAAGTTTCGGTTTCTTAAAATAGCATATATGAGGAAGTAGAAGCAAATAGCCGCTGCTTCTTTTTTGTGCCGAATCTGCTTATCGTTCTTATCTAATAGAGTGCCTTTGATTCGCAGTTGCATAAAGCTGTACATAACTGGCCTTTATGGGGGAGTTTTTATTTTTTGCACAAATGTGCGAAAAAATTTAAATAAGCTAATTTGTTCTATAATGTATTGACGTACTGAGATAAAGGTGATACATTCTAGTTACAAACAAAAATAAATCACTTTCAAACAAAAGAACAGGTTTTTGTTAATTATAAAGATAGGAGCGATTGTAACATATGGAAATGATTGTAGCAGATCAAATTATCATGGGTCTTATCTTAGACGCTGGTGATGCCAAGCAGCATATTTATCAAGCTTTATCACTAGCTAAAGATGGTAAGTTTTCAGAATGCGATGAGCAGATTGAGCTGGCAGATAAGGCGCTGTTAGAGGCCCATAACTTGCAGACCAAGTTCCTAGCTCAGGAAGCTGGAGGAACGAAAACAGAGATTACAGCGCTGTTTGTTCATTCTCAAGATCATTTGATGACTAGCATTACAGAGATTAACCTCATCAAAGAAATCATCGACTTGAGAAGAGAATTACAAGGGGAAAAATGAATCATCAGGAGGATGAAAAGATGGTTAAGATTGGTTTGTTTTGCGCGGCAGGTTTTTCAACAGGAATGTTGGTCAACAATATGAAAATAGCTGCAGCTGAAAAGGGGCTGGAAGCTGAGATTGAAGCTTACTCTCAGGCTAAGTTGGCTGATTATGCAGCGGATCTGGATGTCGCTTTATTGGGCCCGCAGGTAGCCTATACACTAGACAAATCTACAGCTATTTGCGACAGTTGCCATACTCCGATTGCAGTTATTCCGATGGCTGATTATGGTATGCTAGATGGCGAAAAAGTGTTAAATCTCGCTTTGAGTTTGTTGGATAAACATTAAAATCAAGGAGCTTCGTTATGTCTAAAGTGGATACTCAAAAAATCATTGCGCCGATTATGAAGTTTGTCAATATGCGCGGGATTATCGCCTTAAAAGATGGTATGTTGGCAATTTTGCCCCTTACAGTTGTGGGAAGTCTATTCTTGATTGTAGGGCAATTGCCGTTTGAAGGTCTAAATCAAGCCATTGCTGGTGTGTTTGGGGCAGACTGGACAGAACCGTTTATGCAGGTTTATTCAGGAACCTTTGCCATTATGGGGCTGATTTCTTGTTTTTCAATCGGCTATTCTTATGCCAAGAACAGCGGCGTAGAGCCTCTGCCAGCAGGGGTGCTGTCTCTTTCTTCTTTCTTTATCCTCTTAAAATCCTCCTACATACCAGAAAAGGGTGAGGCGATTGCAGATGCGATTGCCAAGGTCTGGTTTGGCGGACAAGGAATCATTGGGGCCATTCTCATTGGTCTGGCAGTTGGCAGTATCTATACGATTTTCATTCAAAAGCATATTGTTATCAAAATGCCAGAGCAGGTTCCTCAAGCGATTGCCAAGCAGTTTGAAGCCATGATTCCTGCTTTCGTTATTTTCTTGCTGTCAATGCTTGTTTATATTATTGCCAAAATAGCGACTGGTGGCGGTACCTTTATTGAGATGATTTACGATGTCATTCAGGTGCCTCTGCAAGGTCTGACGGGCTCTCTGTATGGAGCTATCGGTATTGCTTTCTTCATTTCTTTCTTATGGTGGTTTGGCGTCCATGGCCAGTCCGTTGTCAATGGTGTCGTAACAGCTTTGCTGCTGTCAAATCTGGATGCTAATAAGGCCCTGCTTGCTGCAGATAAGCTGTCTGTCAGCCAAGGTGCTCATATTGTGACCCAACAGTTCTTGGATAGTTTTCTTATCTTATCGGGATCCGGTATCACTTTTGGAGTGGTCGTAGCCATGCTCTTTGCTGCCAAGTCCAAGCAGTATAAGGCCCTGGGGAAAGTTGCTGCTTTCCCGGCAATCTTTAATGTCAATGAGCCAGTTGTCTTTGGTTTTCCGATTGTGATGAATCCGGTCATGTTCCTGCCCTTTGTTTTGGTACCGGTTCTAGCCGCTTTGATTGTCTACATGGCGATTGCGGTCGGCTTTATGCAGCCATTCGCAGGTGTGACTTTGCCTTGGAGTACGCCGGCTATCATTTCTGGCTTTATGGTCGCTGGCTGGCAAGGGGCAGTCATTCAAATTGTCATCTTAGCCATGTCCGCCTTTGCCTATTTCCCATTTGTGAAGTTTCAGGATAAGATTGCCTACAATAACGAATTGAAAAATGAAGAATAATCTGATTTTGATGGATAAAAACACAAAAGTTTCTTGGCTCGGATGGCACAAATGTGCAAAAAATCTTTAATTCAGTAATCGCCTATAAATGATTGAATTCTAGAATTTTGCATGTTACAATAGTTACGAAAGAAATAAATTAAATTTCATAAGAAAGGTCGTAACCAAATGATCCAAGTAAAAGAAGTAGAAAAAACAACAATAAAAACAGACTATTTTGGCAGCTTAACAGAGCGGATGGATAAGTATCGGGAAGATGTTTTAAATAAAAAGCCCTATATTGACGCAGAGCGCGCTGTCCTAGCTACCAAGGCTTATGATAAGCATAAGGAAAAACCAAATGTGCTGAAGCGTGCTTACATGCTCAAAGAGATTTTGGAGAATATGACGCTCTATATCGAAGATGAAACGATGATTGTCGGCAATCAGGCTTCATCTAATAAAGATGCGCCTATCTTCCCAGAGTATACTTTAGAGTTTGTACTCAATGAACTAGATCTTTTTGAGAAGCGGGACGGAGACGTTTTCTACATCACAGAGGAAACCAAAGAGCAGCTCCGTAGCATCGCTCCTTTCTGGGAAAACAATAACCTCCGTGCGAGAGCTGGAGCTCTACTTCCAGAAGAAGTTCAGGTTTATATGGAAACTGGCTTCTTTGGCATGGAAGGAAAGATGAACTCTGGGGATGCCCACTTGGCGGTCAACTATCAAAAACTCTTGGCCTATGGACTGAAAGGTTTTGAAGAAAAAGCTCGCGCAGCTAAGGAAGCCTTGGATTTGACCGATCCGGCTAGTATTGACAAATACCACTTCTATGATTCCATTTTCATCGTAGTAGATGCTGTGAAAGCTTATGCGGAGCGTTTTGTGGCCTTGGCCAATCAGATGGCTGAAAAAGCAGATCCTAAGCGCCGTCAGGAGCTCTTAGAAATCGCTAGAATCTGTTCTAAAGTGCCGTACGAGCCAGCATCAACCTTTGCTGAAGCCGTTCAATCTGTTTGGTTTATCCAGTGTATCCTTCAGATTGAGTCCAACGGACATTCACTCTCTTATGGACGCTTTGACCAGTACATGTACCCTTATGTCAAGGCAGATTTGGAAGCTGGACGTGAAACAGAGGCTAGTATTGTAGAACGCTTAACCAATCTCTGGATTAAGACGATCACCATTAATAAAGTGCGCAGTCAGGCTCACACATTCTCATCTGCGGGCAGTCCCCTTTATCAAAATGTGACAATCGGTGGCCAAACGAGAGATAAGAGAGATGCAGTCAACCCACTTTCCTACCTTGTCTTGAAATCTGTAGCTCAGACTCATCTTCCTCAGCCGAATCTGACTGTTCGCTATCATGCAGGCTTAGATGCTCGCTTTATGAATGAGTGTATCGAAGTCATGAAGCTTGGCTTCGGTATGCCGGCCTTCAATAATGATGAAATCATCATTCCGTCCTTTATTGCTAAAGGAGTTTTGGAAGAAGACGCATACGACTACAGTGCTATCGGCTGTGTGGAAACAGCTGTGCCTGGTAAATGGGGCTACCGTTGTACAGGTATGAGCTACATGAACTTCCCTAAGGTGCTCCTCATCACTATGAACGATGGAATTGATCCAGCATCTGGCAAGCGCTTTGCGCCAAGCTTCGGTCACTTTAAAGACATGAAGAGCTTTGCTGAGCTGCAAACGGCTTGGGACAAGACCTTGCGTCATTTGACCCGCATGAGTGTCATCGTGGAAAATTCTATTGACCTATCTCTTGAAAGAGAAGTGCCGGATATCCTCTGCTCAGCTTTGACAGATGACTGTATCGGACGCGGCAAGCATTTGAAAGAGGGCGGAGCTGTCTATGACTATATTTCCGGTCTTCAAGTCGGTATTGCCAATCTATCAGACTCACTGGCGGCTATCAAGAAGCTGGTCTTTGAGGAAGGCAGGCTGACTCCAGCTGAACTCTGGCATGCACTTGAAACGGACTATGCAGGTGAGCGTGGTAAAGAAATTCAAGAAATGCTGATTCATGATGCACCGAAATATGGTAATGATGATGATTACGCAGATAAGCTAGTGACAGATGCTTATGATATTTATGTGGATGAAATCGCTAAATATCCAAATACCCGTTATGGCCGTGGTCCAATTGGCGGTATCCGATACTCTGGAACATCTTCTATTTCAGCCAATGTAGGTCAAGGTCGCGGTACTTTGGCGACACCAGATGGCCGCAATGCCGGAACACCACTCGCTGAAGGTTGCTCTCCATCCCACAATATGGACAAGAACGGCCCGACCTCCGTATTGAAATCTGTTTCCAAATTACCGACAGATGAAATCGTTGGTGGCGTTCTGCTCAATCAAAAAGTTAATCCTCAGACCCTGTCTAAGGAAGAAGATAAAGTGAAACTTATTGCCTTGCTTCGTACATTCTTTAACCGTCTGCATGGCTATCATATCCAATACAATGTCGTTTCTCGGGAAACATTGATTGATGCTCAGAAGCATCCTGAAAAACACCGCGATTTGATTGTTCGTGTTGCAGGCTACTCTGCTTTCTTCAATGTGCTTTCCAAGGCGACACAAGATGATATTATTGGACGTACGGAGCACACATTGTAAAAGAGGTCCAATTTATGGAATTCATGCTTGATACCTTAAATTTAGAGGAGATAAAAAAATGGTCGGAAGTCCTCCCCTTGGCGGGAGTGACTTCTAATCCGACCATCGCAAAAAAAGAAGGAAAAATAAATTTCTTTGAACGGATTCGCGCCGTTCGGGAAATCATAGGAGAAGGTCCATCTATCCATGTGCAGGTTGTTGCCAAGGATTATGAGGGAATCTTGAAAGATGCTGCTGAAATTCGAAAAAAATGCGGCAATGCTGTCTACATTAAAGTTCCGGTCACACCGGCTGGGCTGGCAGCTATCAAAACTCTTAAACCAGAAGGTTACAAGATTACAGCGACAGCCATTTATACAACCTTTCAAGGGCTATTAGCCATTGAAGCAGGGGCTGATTACCTTGCTCCTTACTATAACCGCATGGAAAATCTCAATATTGATTCAGATGCTGTCATCAGCCAGTTGGCTCAGGCGATTGAGCGGGATCATTCCACCAGCAAAATATTGGCAGCTTCCTTTAAGAATGTCGGTCAAATCAATCGTGCTTTTGCTGATGGGGCTCAAGCCATTACAGCTGGACCAGATATCTTTGCAGCAGCCTTTGCCATGCCGTCTATCGCTAAGGCAGTAGATGACTTTGCTACTGACTGGTCAGCAATTCACAATCAGGAATACATTTAAAAGGTATATATAATGCTCTATCTATAAAAAAGAAAGAGGTGTTCTATGAGAATTTTTGCAAGCCCGTCTCGTTATATTCAGGGAGAAAATGCTCTGTTTGAAAATGCAAAACAGATTCTTCAGTTAGGAAGCCATCCAGTCTTGCTTTGTGATGATGTTGTTTACCAGATTGTTGGGGAGAAGTTCCATGACTATCTTACTCGTTATGGTTTTCATGTGCTGCATGTCGCTTTTAACGGAGAAGCTTCTGACGCTGAAATAGAACGGGTCGTTGCTCTGGCTGAAAAAGACGGAGCAGACTTAGTTATTGGTCTAGGCGGTGGGAAAACGATTGACAGTGCGAAGGCGATTGCAGACATACTAGGACGTCCTGTGGTCATTGCGCCAACCATTGCCTCTACAGATGCCCCAACTTCAGCACTTTCTGTTATCTATACAGAAGATGGCGCCTTTGAAAAGTACATTTTCTATAGTAAAAACCCCGAACTAGTCTTGGTGGATACGAAAGTGATTGCTGGAGCGCCAAAACGACTTCTGGCTTCTGGGATTGCAGATGGTTTAGCGACTTGGGTAGAGGCGCGTGCCGTCCAGCAAAAAAATGGCACTACCATGCTGGGACAAAGACAGAGCTTAGCTGGTGTAGCAATTGCGAAGAAGTGTGAAGAAACCCTGTTTGCAGATGGCCTGCAGGCTATTGCAGCCTGTGAAGCAAAAGTCGTGACCCCAGCTCTGGAAAATATCATTGAAGCTAATACCCTTCTCAGTGGCGTCGGCTTTGAGAGCGGCGGTTTAGCAGCAGCCCATGCTATCCACAACGGCTTTACAGCTCTTACAGGCGATATTCATCACTTGACCCACGGCGAAAAAGTAGCCTATGGAACCTTGACCCAGCTCTTCTTAGAAAACCGTTCTAAAGAAGAATTGGAAAAATATATTCGCTTCTATCAAAAGATTGGTATGCCGACAACTCTGAAAGAAATGCATTTAGAAAATGCCAGCTATGAAGATTTGCTCAAAGTTGGTCAGCAAGCGACGATTGAGGGCGAAACCATCCATCAGATGCCATTTGAGATCTCAGCTTCTGACATTGCAGGTGCCATCCTAGCGGTTGACCAATATGTCAGGGATTTGGATAAATAAAAGCAAAATATAAAACAAGCCACCTTCCAACCGCAGGGTTGACAGCGTGGCTTGTTTTTTGGTGAGATTACCAGATGTAAGGAAAAACTCGGTATCTAACCTTTTGGCAATAAGCTTCGTAATTTTTCCCAAAGTGATTGCCTAAAGCTTTTTCTTCTACTCTAAGTCGAATGCTATATCCTAACACGAGTAAAATAAGACTGATAATGATTGTAAGAGGATTTAATGAGGTAATCGATAGGCCAAGGATAGACAAGATACTGCCAGTATAGGCAGGATTTCTTACAATAGAATAAGGGCCATGGTCTACTAATTGTTGACTATCGGTTGTTTGAACGGCCAGTGTGAAGTTTTTTCCGAGATAATTGACTGCATAGACCCGCAATGCGAACCCTGCTAGAGAGATGAGGATTCCAAGATAGATAGTGAGGCTGGGTAAGTGGGGGACAAAGTCGAGAAATTGTCCGTTTATCAAAAATAAGCAGCTGATAACACTGCCGATAATGATGTAGCGGCTCCCTTTATCTGCAGAGTCGTTTACATTGTTTGACTTAGTTTTATTTTTAATCCACATTTCTGTGCTAATAAAAATAACAAAGAAAATATAAGTGAAAATAGTTTGTAAATTCATGATTATTCCTCCGGTCAGAATTATACAACTTTTAATTTTAGAGGTCAATCAGAGATTAACTCTTTAGAAGTGAGTTATAGTTTTTGTCAGTGAAAACTGGATTCCATCTTTCAAAATCCCTTGAAAATGGTATAATAGAAACAGTACAAAAAATGGAGAAAAGAATGGCTTATTATAATCACAAAGAAATCGAACCCAAGTGGCAGAAATATTGGGCAGAGCACCATACATTTAAGACAGGTACGGACAAGGACAAGCCTAACTTTTATGCGCTGGACATGTTTCCTTATCCATCAGGAGCGGGACTCCATGTAGGGCATCCGGAGGGCTACACGGCGACAGATATCCTTAGCCGCTACAAGCGAACTCAGGGCTATAATGTCCTGCATCCTATGGGCTGGGATGCCTTTGGTCTGCCAGCTGAGCAATATGCCATGGATACAGGCAATGACCCAGCTGATTTTACAGCGGAAAACATTGCCAACTTCAAGCGTCAAATCAACGCTCTAGGATTTTCGTATGACTGGGACCGAGAAATCAATACAACTGACCCTAACTACTATAAGTGGACCCAGTGGATTTTCACAAAGCTTTACGAAAAAGGTCTGGCTTATGAAGCTGAAGTGCCAGTAAACTGGGTTGAAGAGCTGGGAACGGCTATTGCCAATGAGGAAGTTCTGCCAGATGGCACATCTGAGCGCGGCGGTTATCCAGTTGTCCGCAAGCCTATGCGTCAGTGGATGCTGAAAATCACAGCCTATGCAGAACGTTTGCTCAATGACTTGGAGGAACTGGACTGGCCAGAGTCTATCAAGGATATGCAGCGCAACTGGATCGGCAAGTCAACCGGTGCCAATGTAACCTTCAAGATTAAGGACACTGACAAGGACTTCACCGTCTTTACGACTCGTCCGGACACCCTTTTTGGTGCGACCTATGCTGTTTTGGCTCCGGAGCATGCTCTCGTTGATGCCATTACAAGTGCCGAACAAGCCCAAGCAGTTGCAGACTACAAACATGCAGCCAGTCTCAAATCTGACCTTGCTCGGACTGATTTGGCTAAGGATAAGACTGGTGTCTGGACAGGCGCTTATGCCATCAATCCTGTCAACGGCAAGGAAATCCCCATCTGGATTGCCGACTATGTACTTGCAAGCTACGGAACAGGTGCTATCATGGCCGTTCCTGCGCATGACGAGCGCGACTGGGAGTTTGCTAAGCAGTTTGACTTGGAGATTATTCCGGTTCTAGAAGGTGGCAATGTTGCTGAAGCTGCTTATACAGAGGACGGTCCGCACATTAACTCGGGCTTCCTAGATGGCTTGGACAAGGCTGCCGCTATCGACAAGATGGTTGCTTGGCTGGAAGCAGAAGGTGTCGGAAATGAAAAAGTCACCTATCGCCTGCGCGACTGGCTCTTTAGTCGTCAACGCTACTGGGGTGAGCCGATTCCAATCATTCATTGGGAGGATGGCACCTCAACAGCTGTTCCTGAAAATGAATTGCCACTTGTCTTGCCTGTAACCAAGGATATCCGCCCTTCTGGTACTGGTGAAAGTCCTCTGGCCAATTTGACTGACTGGCTGGAAGTGACTAGGGAAGATGGTGTTAAAGGACGCCGCGAAACCAACACTATGCCTCAATGGGCTGGTTCCAGCTGGTATTACCTGCGTTACATTGACCCGCACAACAATGAGAAATTAGCAGACGAAGAACTGCTCAAGGCTTGGCTTCCAGTTGATATTTACATCGGAGGTGCGGAGCACGCCGTGCTCCATCTTCTCTACGCTCGCTTCTGGCACAAGTTCCTTTATGATATCGGAGTGGTTCCGACCAAAGAGCCTTTCCAAAAACTCTTTAACCAAGGGATGATTCTGGGGACTAGCTACCGTGATAGTCGCTGTGCTCTAGTGGCGACAGACAAGGTGGAAAAACGCGATGGTTCTTTCTTCAATATCGAAACTGGAGAAGAACTGGAGCAAGCGCCTGCTAAGATGTCTAAGTCTCTGAAGAATGTAGTCAACCCAGACGATGTAGTGGAGCAATATGGTGCCGATACGCTTCGTGTCTATGAAATGTTCATGGGGCCGCTGGATGCATCTATCGCTTGGAGCGAAGAAGGGTTAGAAGGCAGCCGTAAGTTCCTTGATCGGGTTTATCGTCTCTTTAACTCTAAGGAGCTGGTCACTGAAAATAGCGGAGCTTTGGACAAGGTCTACCATGAAACGGTCAAGTCTGTCACAGAGCAGATTGAGGAGCTCAAGTTTAACACAGCCATTGCCCAGCTCATGATCTTTGTCAATGCAGCCAACAAGGAAGAAAAGCTCTATGTCGAATATGCCAAAGGCTTTATCCAATTGCTGGCGCCTTTTGCACCGCACTTGGCTGAAGAACTCTGGCAGGCAGTCGCTCAAACTGGCGAGAGCATTTCCTATGTAACTTGGCCAACTTATGACGAAAGCAAACTGGTCGAAGCAGAGGTCGAAATCGTAGTTCAAATCAAGGGTAAAGTTCGTGCTAAGCTAGTCGTAGCTAAGGACTTGAGCCGTGAGGAACTGCAGGAAATCGCTCTGTCAGATGAAAAAATCAAGTCCGAAATCGCTGGCAAAGAAATCGTCAAAGTGATTAGTGTTCCAAATAAACTGGTTAATATTGTGGTGAAATAATTAGTTTGTTTATTGAATTTACAGGAGATTTCAATCAAGTCCGCATTAGACAATAAAATGCTATTGCAATCAAATACAAAGAAGACCTTGAGAAAATTTTCTCAAGGT

>c134_g1

AAGAAATTGACTTTGCGACCAATGAACTATATAAGGTCGGGGCTAATGTCAAGCGTAAGTATTCCTCAGCTGAGTACAATAATCTGGACATTTACCAGATTAACTCGACTTACTACTCAGCCTTGGGCGATGACGACAAGAAGTACTTCATTAGCCGTCTGATTCAGGCTTTTGCTCCAGGGATTCCACAGGTCTATTATGTTGGTTTCTTAGCTGGGAAAAATGACCTTGAACTGCTAGAAAACACTAAGGAAGGCCGCAATATCAACCGTCATTATTACAGTAATGAGGAGATTGCTGAAGAAGTCAAGCGTCCAGTTGTGCAAGCCTTGCTCAAGCTTTTCAATTACCGCAACCAGTCTGCTGCCTTTGACCTAGATGGCACTATTGAAGTGAATCTATTGGATGAAAATAGCCTCCGGATTGTCCGTAGCAATGCAAATAAGTCAGTCACTGCTCAAGTAGTTATAAACCTCAAAGAATTGACATATAGCGCTAGTGAAAATGGCCAAGCTATCACTTTTGAATAGAGATAAGAAAAGAGAAGAAGAATGGTAGAATTAAATCTGAAAAATATCTA

>c134_g2

GGTATGATGCGGTATTTCCCTCAAGTTTGGGCTAGTGACAATACCGATGCCATAGCTCGTTTGCCAATCCAATATGGGTCTTCTTATCTTTATCCGACTATTTCAATGGGTGCCCATGTGTCGGCAGTGCCTAATCATCAGATGGGACGAATTACTCCGCTGGCAACTCGGGGTCATGTAGCTATGATGGGAAATCTGGGGTATGAGCTTGATTTGACTAGTCTATCAGATGAAGAGAAAGCTGCGATTGCTGACCAGGTGAAGTTGTATAAAGAATTACGGCCAGTGGTCCAGCTAGGGCGTCAGTATAGGCTAATCAATCCAGATGTTGGCTCCAATGAAGCAGCAGTTCAATTTAACTATGAAGATCAAACGATTGTAACCTACGTCCGAGTCTTATCAGTTGTGGAAACGATGGAAACAACCTTGAAACTAAAAGACTTAGAGGAAGAAGGGCTTTATGAGCTGCAGGGAAATGGCGCAGTTTACTCAGGTGCAGAACTCATGTATGCTGGTATCACTATGAATCTTCCGCAAGGAGATTATCTTAGCAGACAGTTGCATTTTATTAAAAAATAAGGAGGCTAGACCATGAAATGGTATAAAAAGATGAGCTTAGCTGCTATTACAGGACTGTCCCTTTTGGGGCTGTCAGCTTGCAGCAGCCAAGGTGAGTCAACAGATGGTAAGGTAACGATTGAGTATTTCAATCAGAAGGGCGAAATGGTTGATACCCTTCGTGAGATTGCAAAAGACTTTGAAAAAGAAAATCCGAATGTGCATGTGAAAGTGGTGAATGTCCCTAACGCTGGGGAAGTACTCAAAACGCGCGTTCTGGCTGGAGATGTTCCTGATGTTGTCAATATCTATCCCCAGTCTATTGAATTGCAAGAATGGGCTAAAGCTGGGTACTTTGAAGATTTATCTAATAAAGATTATCTCAAGCGAGTTAAGAATCATTACGCAGATAAATATGCGATAGATGGAAAGATTTACAATATCCCTTATACTGCTAATGCTTATGGTATCTATTACAACAAGGATAAATTTAAAGAACTGGGATTGAAAGTTCCAGAGACTTGGGAGGAATTTGAGGAATTAGTTGATACGATTATAGCAAAAGGGGAAACACCTTTTGCCATTGCAGGAGCAGATACTTGGACCTTGAATGGTTATCATCAGTTGGCTCTTGCCACTTCTACAGGTGGCGGTAAGGAAGCGAATGATTACCTACGTTTTTCCAAACCAAATGCCATCAAGTCTTCAGATTCAGTGCTTAAAGATGATTTCAGACTGTTAGATTTATTCCGCAAAAAAGGTGCCATGCAAACCAACTGGCAGGGAGCGGGCTATACTGATGTTGTCGGTGCCTTCGCTAGAGGCGATGCCCTGATGACTCCAAATGGTTCTTGGGCTATCACAGCAATCAATGCGCAAGATCCTAAGTTTAATGTTGGAACGTTCCCTTTCCCTGGAAAGCAAAAAGGACAAAGCTTGACCATTGGAGCAGGAGACCTAGCTTGGTCTATCTCGTCAAGTAGTAAGCACAAGAAAGAAGCCAATGCTTTTGTTGAATATATGTCACGTCCAGAAGTTATGCAGAAATATTATGATGTGGACGGCTCACCAACAGCTATTGAAGGTGTCAAGGAAGCTGGGGCAGATGCACCGCTAGCCGGTCTTGCTGAATTAGCATTTACAGACCGACACCTCGTTTGGCTAGCCCAAGACTGGACCAGTGAAAGTGATTTTTATACCTTGACTGGGAACTATATTACAACTGGAAACAAAGAGGATATGGCGAAAGCACTAAATGCCTTCTTCAATCCGATGAAAGCAGACGTAGAGTAGGAGTAGATCCACTATGAAAAAATTGATTGAGAAATATTGGGGCTGGCTCTTTGTCCTTGTTCCCCTTGTACTACAGGCTGTATTCTTCTATGTGCCCATGTTTCAGGGTGCCTTCTACAGCCTGACAAACTGGACTGGTTTGACCTATAATTATAAGTTTGTCGGACTCAATAACTTTATGCTCCTCATGAGCGATCCTAAGTTTATGAACGCAATAGGCTTTACATTTATCATTACTATCTGTATGGTGGTTGGTGAGATTATGCTGGGAATCTGGATAGCGCGAGCTCTCAACTCGAAGATAAAGGGGAAAACTTTCTTCCGAGCTTGGTTTTTCTTCCCTGCTGTCCTATCTGGATTGACTGTGGCTCTTATCTTTAAGCAACTTTTCAACTACGGATTGCCAGCTATAGGGAATACCTTAGGAATTGAATTTCTTAAAACCAGCCTTCTAGGAACGAATGGCGGTGCAATCTTTGCAGCAATCTTCGTCTTGCTTTGGCAAGGGGTAGCCATGCCTATTATCATCTTCTTAGCTGGTTTTCAGTCTATCCCGACTGAGATTACAGAAGCAGCTCGAATTGATGGAGCTAGCAGCAAACAAATCTTTTGGAAGGTGGAACTTCCTTATCTCCTACCAAGCGTTTCCATGGTCTTTATCTTGGCTCTAAAAGGTGGTTTGACAGCGTTTGACCAGGTCTTTGCCATGACAGGCGGCGGTCCGAATAATGCGACCACCTCTCTAGGACTTCTGGTATACAACTATGCTTTTAAGAGCAATCAATTTGGTTATGCCAATGCCATTGCTGTTATCTTGTTTCTCTTGATTGCGGTTATTTCTATTATCCAGTTGAGAGTATCTAAGAAATTTGAAATCTAAGAGGAGAAGCCCGATGAAAAATGCAGAAAGAAAAGCTAATTTTAGTAAATATATCTTGCTAACACTGGGATCTATTATCATTTTAATTCCACTCTTAGCAACAATATTTAGTTCCTTTAAGTCTACCAAAGACATCGTTAATAATTTTTTCGGATTTCCGACTCAACCGACTTTGGAAAATTTCCAACGCCTTTTGGCTGATGGTATCGGAGGCTACTATTGGAATTCAATTGTAATCACGGTTCTATCTTTGGCTGTTGTCATGATTTTTATTCCCATGGCTGCTTACTCTATCGCTCGTAATATGTCTAAGAGAAAAGCTTTTGGTATTATGTATACGCTTTTGATTTTAGGGATTTTCGTTCCTTTTCAAGTTATCATGATTCCTATTACAGTTATGATGAGTAAGCTAGGACTAGCCAACACTTGGGGATTAATTATCCTCTATCTGGCTTACGCTGTTCCACAGACTCTCTTTCTCTATGTAGGCTACATTAAGATTTCCATTCCAGACAGTCTGGATGAGGCAGCAGAAATTGACGGTGCTGGAAAGTTTACAACCTATTTCAAGATTATCTTTCCTATGATGAAACCCATGCATGCGACAACCATGATTATCAATGCCCTCTGGTTCTGGAATGATTTCATGCTTCCTCTCTTGGTACTCAATAAAGACTCCAAGATGTGGACTCTGCCACTCTTCCAATATAACTACACAGGTCAATACTTTAATGATTATGGACCGAGCTTTGCTTCTTATGTTGTTGGGATTATTACCATTACTATCGTCTATCTCATCTTCCAGCGCAATATTATTGCTGGAATGAGCAACGGGGCAGTGAAGTAAGCTCAAGATACAAAGACTGTAAAAAGCTCACAGTGCATACAAAGGCAACCACATCCGTAAACTACTGAATTAGTAACGGCTGTGGAAAAATAGGGAATCTTAAAAAGAAGCTTCAGCTTCTTGGAAGATTCATCTTTTTCGCACAGAGCTTAGGGCGTTTTCCATTCAGCGCATGGGAAAGAGTATATAAGACAAGATTTTAGAGGAGAAGACTATGGCTATTCAAAATAAGACCATGCTGATTACTTACTCAGACAGCTTGGGGGAGAATTTAAAAGATTTATATGACAATTTAGAAAAGCATTTTGGGGATGCGGTTGGAGGAGTGCATTTGCTTCCTTTCTTCCCATCAACTGGTGACCGGGGATTCGCCCCAGTAGACTATGAAGAGGTTGATTCAGCCTTTGGTGACTGGGAGGATGTAGAAAAGCTGGGCGAGAAATATTATCTCATGTTTGACTTTATGATCAACCACATCTCACGCCAGTCTAAGTATTATATGGATTTTCAGGAAAAGAAGGATCAGAGTGAGTATCGTGACCTCTTTCTCAGCTGGGATAAGTTTTGGCCAGAAAATCGTCCGACGCAAGCTGATGTTGATTTGATTTATAAGCGTAAGGATCGGGCACCTAAGCAGGAAATCGTCTTTGCAGATGGAAGCACTGAGTATTTGTGGAACACCTTTGGTGAAGAGCAGATTGATCTAGATGTTCGCAGTCAGGTAACTAATGATTTCATCAAGAAAACATTGCGTCAACTGTCAGAGCATGGCTGTGATCTGATTCGTTTAGATGCATTTGCCTATGCGGTCAAAAAGTTAGATACCAATGATTTCTTTGTTGAGCCAGAAATCTGGGATCTCTTAGAAAAAGTCCAGCAGCAAGCGGCTGAGTTTGGGACGGATATTTTACCAGAAATTCATGAGCATTATTCTATCCAGTTCAAGATTGCAGAGCATGGCTACTTTGTCTATGATTTTGCGCTGCCGATGGTAACTCTTTATTCTCTATACAGTGGTAAAGCTGAGCGTCTAGTCAAGTGGCTTGAAATGAGTCCTATGAAGCAATTCACAACACTGGATACTCACGATGGTATTGGGGTTGTTGATGTTAAGGATATTCTGTCTGATGAA

>c136_g1

GGAACTCATAAAGAAATCATCGCTAAGCTGGATTATCCTGCTCCTAAATGCCCTTACTGCCAAGGACAAATGGCTAAATATGACTTCCAGAAAGAAGCAAAAATTCCCTATCTAGAGTGTGTGGGATACAAAACGCTGATTCGGTTAAAGAAACGACGTTTCCGCTGTAAAGTCTGTAGGAAAATGGCGGTCGCAGAGACTTCCTTAGTCAAGAAGAATCACTAAATCGCAGCCATCGTCAACCAGAAAATCGCTCAAAAACTAATCGAGAAAGTCCCTATGACAGCCTTCGCTGAAAGCTTAGCTGTCTCTACTTCCACCGTCATTCGTAAATTGAAAGAATTCAAGTTCAAGACTGACCTTAACTGCCTTCCAGAATACATGAGCTGGGATGTGGAAACAGTCAGGGAAGTGACTGTTTCAATCGGCAGATAGAGATGAACTTTATTGCACAGGATTTCGATTCCAGAAAGATTATAGCTATCTTAAGATGGGCGGACTCAAGCAACGATTCGCAATCACTTTCTGCGCTATTCCAGACAGGTCCGAAACCAAGTCAAAGTCATTACCATGGACATGTTTAGTCCCCACTACGATATTGCCAGAAAACTATTCCCAAACGCTAAAATCGTTCTGGATCGTTTTCACATTGTGCAACATCTCAGCCGTGCTATGAACCGCCTTCACATCCAAATCATGAATCAGTTGAATAAAAAATCGCACAAATATAAGGCACTCAAACGCTACTGGAAACTCATTCAACAGGATAGCCGTAAACTCAGCCATAAACGTTTTTATCGTCCAACTTTTCGCATGCACTTGACCAGCGGAGAGATTCTAGAAAAACTTCTCTCTTGCTCTCAAGAACTCCGAGAACACTATAAACTCTACCAACTCCTGCTTTTTCATTTTCAAGAGAAAAAAACTGAGCATTTCTTTGGACTCATTGAAGATACCATTTCTTATGTAAATCCTATTTTTCAAACTGTTTTTAAGACCTTCTTGAAAAACAAGGATAATATCCTGAACGCACTGGAACTGCCCTACTCAAACGCAAAACTAGAAGCTACTAACAACCTCATCAAAGTCATCAAGCGAAACGCTTTCGGCTTTCGGAACTTTGACAATTTTAAAACTAGAATCCTCATCGCTTTGAATATCAAAAAGGAGAGGACTGATTTAGTCCTCTCCAGGATATGACTTTTCATCAACCCACTACAGTT

>c142_g1

GGAAAGGTAAGTTTCTTTCCGTTTTTACATTATTTTTTCCCAGAATCATTCTTATTGATACCGCCGTTGTTTGAAGAACTGGACGGTTTATTATTGGTATTATTAGAAATCTGCGAGCCAGATGCTCCACCAATTCCCTGCTTAGCTTGATTCGGCTTATTCGCATTTGTCGCAGCATTTCCGACACCTGATGCTTCACTTTTAGTTCCTGCCTTCGAGTTGGCTCCAGAAGCTAAAGTATTAGAAACCGCTGTTCTTCCAGCATTATAGTTTTCTCTAGCAGCACTGCCAACATTTTTAACAGAATTCTTAACTCCTTCTGCCGTGCCTTTAACAGAGTTCTTGGCGCTATCATAAGCACTATCAATACCTGCTTTAGCATGAGAACCTAGATTGGCTGCTGCCTTACCAACCCCGCCAGCCTGCTTAATGCCTTCCATAGCACCCTTAGCGCCGCCAACAGTTCGAGCAGCTCCAGCTCCAATGCCTTTGACTGCTGCACCAGCACCTCGAGCCGCACCTTTAGCGCCGCCAACCGCTTTCCGAGCTGCCGTTCCAACGCCACGAGCAGACTTCTTAGCCACACCAGGGGCTGCTTTAGCCATTTTCATGCCAGAGCGGGAAGCTGCTGCACCCGCAGCCATACCAGCGCCGGCCATACCGGCCAGTTTGCTTCCTCCGCCTTGAGACGGAGAAACACCTAACCAACGCTCAACAGCACTATTACCACTAGTTAAAGCAAAATACACTCCTGCATAAATAATTATAGAAGCCATGATATTTTCCCAATAACCAAGCCCTGTACTAAAACCATCTTCAGATAGGCCAGAAATCCCTTTAACCAATATGGTAGGAAAGTCTCTTAAAATACTCATAGCAACTCGAAGTAGGATAACCTCAAAGAAAATACCTGTAATAGTGCCAAAAATGGTCTGAAGCAATTCCTTGAACTTATCTGAGTTTTCAACAGAAGTATAGCCAACGATTGGAGCAATCATACCAGCCACAATAACTTGAAAAACCGATTGAACAAATTTAATTGACATACTGATGAGCAAGGCTATTAAAATAATCTGTTGAACAATAAGTCCAATCCAGTTAACATTATAGCGAAGATAAACTGCAGAGAAAACATTCTCTAAATCGCTCCAAAAACCTGGCTTAACTTCCGCAATCCGAACATTATCTGGCTCATTGGATATTAGAACCGAACTCAGGAGGTGACCAACTCCTTTCAGTTCTGAGTTATTGTCAAATTCTTTCAAAACTTCTTGGTCCGTTCCTCCATACCAAGCAGCAAAATCAGTCTGTAATAAATTATCATCACTTAAATTATTTAATTTAGTCTCTGATGGATTCAAGAAACCTTTACTGTCCATCCCTAGCACATTGATATCAAATTTATTCTGGATGACCACGTATAAATCAACTACATTTTTTTCATAAGGCTTTAGGGATAAGGAGGTAAAACCAGATTTCCCATCGTCCGACATTGTTCTTACTGTTTGCGCATCACGAGCCAAGAATCCAGAAAACTGCTTAATAGCCTGGGGCAGTAATCCTACAACTGCTGTAACCAAAAGAAAGTGGCTAATAATATCTTTATATCTTGCTAGACCTGTAACAAAATTCGCTGTTACAAACATAATCAAAAGTAAGACAAAAACAACAATCCCAAGTTTTTGCAGGCCATCAAAAATCTGTCCGATAAAGGTATCATTCTTTTCAAGATAATCAAAGAAACCAAATAACTTAAATAAATTATTATATATCGTTTCTAAGGATAGACTAATAGAATACAGTGCTTTGGTAATCCAACCTGGAAGGTAGAGAAAATAAGCAAAAACCTGAGGAATAGATGATAAATAATTAGACCATTGTACATAAAAATTAGCTCCAGCTTGAGTCTCCGGGGCATTATAAGTCCATTTATCCCCTACACCACCAGCTCTTATAGGAGGAGCCTTACTAAATAGGTCTTGAAGGTTTTCGTAACGCGTACCCACTCATATCACTTCCCTTCTTCGGAAGAGGCTTGGCTGGATTCAGACGGCTGGGATTTATCTGAGCTGCCTTCTGTTTCAGGTTTTTTATCGCCTTCTTCCGACTGCTTCTCTGAATTTTCTTCATCAGCTTTTTTCTCACTGATGTAGATACTGGTGACAGGTTCAGAAGTAATCAAGAAACCATATTCAGCCTTGCTATCTTCTTTAATACCAAATGAAAGGCGTTTGATTTTATTGACTCCTTCAGCTGTCGAAAAAGTAATCACATAAGTCAAGGTAAAAGAACCATCTTTTTCCAATTTGACACTTTCCAGCAGATTACTGATAACCTGAGCCTGCTCTGGCTGAGTATATTTAGCATCTCCAGAAGAGAGGAAAGGATTGAGCGCTTCCTTATTTCCAGAATAGTAATTTGGAATAAAGAAACGTCCAAACACATCCGCTGCATGCTCTTTTGCTTGAAGTTTTTGGATGTTTTGCTGCTCTTGATAAAGATAAGCGGTCTGAGCCTCTAGCTCTGCTCGCTTGTTATTACCCATGATACCAATAACAACACCTACAAGGCCAATCAACAAACCTGCTATCCCAAGCCCCAGAGCAATCTTGCTCAAGAGCGGATTTTGTTCTCCCATTTCAGATACTCCTTTATTTCTAATCTTTTTAGAACTAGCTCTTTGCAGGGCTCCCTGCACATCTGACTCAATAGGGAAGTAAGGAAGATCCGCCTGAGATAAGCCCAGACTTTCCGCATATGTTCTTAAAATCCCATTTTCTTTATCCGTAAAATAAAGAGCCAAGTCATCATAAGTGTATTCTTTGAAACGGGGATCGCGCAAGGAAGCTTCCATCAGCTTGGTCATCAGATTATCATAAGTATGATCAATCAGAAAAGGGCTGATACTGGGTTCGTCTTCAATAGAGGGCTTGCCGCTTTCGTTATTCGCCAGAGAGACCTTAGCAAAAGTAAAATCCCCAAATTGATCCCGCAAGGCACGAAAAAGAACCTCCGCCTGAAGAAAAGTCAGATAAGGCAGACTGTCCTCCATGGTCCCAGTGTCTAGAAAATGCTCTTCGTCCAGATCTTCAATTAAATCCTTAATCGCTGCATTGTGCTTGGGAGAGTCCTTGCCTTCCCATTCAATATAAACAAATGAGGAGTCTTTGATAACTCCTCCTTTTCCTAAACCAAATCCTGCCATAGATTAACCACCAAACATTTGCTGAATAAATGGAACGGCTTGACTGGCAAGGACAATGACAACAACACCGATGATTACATAGACAATGTGGCCTTTAGCCCATTCACGCATCTTTTGTGATAACATCAGACCAATACCAGCTACAACCAACATAGCCACGGCAATAGTGAAACCAAGGGCAGTAAAATTTTGCGTCGCCTTATCTGCTCCCTGATTGACTGAATCAAAGGGGTTTTTCCCGCCTGCAAAAACTGGACTAGAAGTCAGAAATGCAGCGAGGGCAGTCATCACTGCTAGCCATTTTTGTTTAACTTTAAGATTTTTCAAGGTAGTATCTCCTTTTCTTATTTATCATGCTTTATTATAGCCTAGTTGCATATCGGTTTCGCTGTATCATAAAGAGAAATGAAGATTTTCCCAAAAGATTTATCTGATTCTCTTTTCGA

>c143_g1

AAGTACGTCCGCATCGACGAGTGCATCAGTTTCAGCAAGTACGTCTGCTTCAACGAGTGCATCCGTGAGCGCAAGTACGTCAGCCTCAACGAGTGCATCAGTTTCAGCAAGCACATCTGCGTCCACGAGTGCGTCAGTTTCAGCAAGTACATCCGCGTCGACCAGTGCGTCCGTAAGTGCAAGTACTTCAGCCTCAACGAGTGCATCCGTGAGCGCAAGTACGTCAGCCTCAACGAGTGCATCAGTTTCAGCAAGCACATCTGCGTCCACGAGTGCGTCAGTTTCAGCAAGTACATCCGCGTCGACCAGTGCGTCCGTAAGTGCAAGTACTTCAGCC

>c144_g1

AGTCAGTTTATTTGTGGCTGTTTGTCTAGGAGGAGGAATGTATATGGCCCACAAAAATCAAGAATTTCAGAATGAAATGACAAGAATTGTCCACAGTGAGGAAGTAAAGAAATTGATTGTGGAAGAATTAAAAGCAATTGATCCAAATGCTTTAACGGAGAAAGGGAAAATCCGTTCCTATAAAATTGATGATTCAACCATTCGTCATAATCCAATGGGAGGCATTATGTTTGATATTATCATCAATGATAGTATAAGCATGGTTGGGAAAATGGGAATTCAAAAAGATGGCGGAAGTGAGCAACTAAGTTCAGTAGGTATGGATGAATCAGTAGGTTTGCAAGCTTTGGTAGGGGAGTGATGATTAGTAATCCAATAACCTTGTTATAGCAAAATTTCTTATTTTAATTTGAAGTTGTAACGCTTATATTATATAATAGATTATAAGAGTAACAAGGGGTGTCTCATGAAAAAAAAACATAAAATTATACTAATTATCGTCAGTTTGTTTGTAGCTATTTGTCTGGGAGGGGGAATGTATATGGCTCACAAAAATCAAGAATTCCATAATGAGATGACAAGAATTGTCCATAGTGAGGAAGTGCGGAAGCTGCTTGAAGAAGATTTAAAAAGAAAGGACCCGAATGCGTTAACAGACAAAGGGAAGATTCGCTCTTATAAAATTGATGATAGCTCCATTAAGCATAATCCGATGGGAGGAATTATGTTTAATATTATCATTAATGATAGTATAAGTATGGTTGGGAAGACTGGTCTTCAAATGGATGGAGAGAATGGGAAAATTCGTACGGATGGTATGACTGAATCAGCGGGTTTGCGGGCATTATTAAGTGAGTGATATTAATGATTAATTATAATGAACAAGAAAGAAATGAAATTGCACGGCTAGAGTACAAAGATTTATCACATGGTGAGGGCGCCAAGATAAAATCAAGTGATGGTTCTGAGATTACTGTCGGCTATGTTTCTGATATTTTAGGCAAGAAGATTGAGGTGGGAGATTTATCTGTATTTCCAACACAGAAGAAAAGGGTCAAGGATAATGAAGTTGGTTTGGATGGCTATGTGTTGACAGATAGATGGATGTCGGAATCTGATTCCCCAGAAGATGTTAAGGAAATCACGGTGCTATTTGAGGGTTCACTTGTTGATCCGGAGCATAATATGACAGGAACTTTGAATGATTGGGGAAGAACAGATGCTCAAATGGCAGCGAAGATTTTGATGGGACAATGGGCAGGAATAAGAGGGGCGAAGCCTAAACAGTTAGCACTAGCTGGAGATAGGTTGAAAGAAATAATGGATAAATATCCTAATGCTCGGGTTAGTCTCTATGCTCACTCTCTGGGATCGATGGATGGCCAGGTCGCTTTGGCGAGTCTTGAAGACAGTTATTTACAGCGAATTGACGGTGCCTATCTCTATGAGGGACCAAATACTTATCCTATTTTAACTGATAAGGAGAGACGACAAGTTGATAAAATTAAGTATAAAATTTTTAATTATATAGATCGAAAAGATATAGTTACTATTGGTTACCCAGAAAAGGGAAGTGAAGGAGCTGTTGGAACTGTTGTTAATATTAATAGTAAAGATCGAAAAAATATAGGCACCCAACATATGTGGGGTGGTTATGAATATGATTCAGGCCATTTAAATGTGAGTGAGTCAGATCTTCAGGATTACCGCCTTGCCCGCGCCAAGCAAGCTATGGAGCAACTTGATATTAAGAAAAAAGCATTGAGTGAACGCTACCAGAAAATGGTTACTGCTGGTTATACTAGAACTGAAATGATTTACCTAGATAGTGAGCAGGCCACAACTTTTGCATCCAGTTTGCAAAACTTGGCAGTTATTTCGACTGAAGCAATTATGGCTTTTTGTGATTATGGTGTATCTAAAGTCAGTGGAAGGTGGGATGCTCTGTTAGCTCAAGCTCAGGCGATGCCGAATGTCTCTAGACTGTTAAGTGAAGCAGAGGTGATAGATGCTCTTTCTCAAGTAGGAGCTACAAAGGATACCGTTGAAACTAGCATCATAACTGAGCTGAAAGATATGCGGAACAAAGCCGTCAAGACTAAAGAAGAATTTGATGGACTAAGTTCTAAACTTCTTAATGGAATCCAAGAATTAGTGAAAAAGGATGAGGGGCTTGCTAGGGAGTACAAAAGATGGGGCAACATATAGAGAATAAGAAGGATAAGCTTTATGAACAGCTCAGGAGTACTCAAGAACGCTATAATGCGGAGTATGATAAATATTTAGATGAGAAGCGAAATTTCGAAGAAGTGATGACTGAAACGGATGACCTCTATCATTCTGCAAGGCAGCAAATACAAGATATGGAAGATTATACAGTTTCTTGCTTGAGACGTTCCACAGAAGGTGCTCAGCTTATCCATGAATTTTATGATAAAGTTTTTCAAGTACAAGATAATCTTGAGATAGAATACCGTAGAGAGCGCGAAGGGGTTGAAGAGGAATGTTCACTTTTGGATAAACGATTTCGTAAAAAGTCAGATAAATATGATGAAGAATTAGCTCATATAAGGAGAGATATCTATGGGGAAAAGTAAAATAGCTGCTAAACTTGATATAGACGCTTACCTTAAAACTTTGGCTTTAGGGACGATTAAGACATTAGGTAAGGCAGCATTGGCGGAAGTTCAAAAGGAAAGAGATGATGTCAAAAAAAAATGTCTCTACTGTTGCGACAGACTGGGATCGGTCTGTGGGTCAGATTATAGGTGGTTTAAATCAAGTTTTGGTCGGTCAATTTTCTAATCAGGTCAAAGAAAGTGCCCAGCAAAAGAGAAGTGGCATTCAGGAATTGCAGGATAATAAGTAAGGCTTTGTTTTTACCAGCCTCACACCGGATAACTCCCGCCCGTTTAAGGCGGGTTTTTGCTTTTGGTAAAATAGTTTATACTATATAATGAAACTGATAAAGATTGAGGAGTGTTTATATGTCTAAGAGAAGAAAAATGTTTATGGGGTTTATTGGTCTAAGTGTATTGATATTGTTAGGGGGAGGTCTCTACCTAGCTCATAAAAATCATGAATTTCAGAATGAGATGACAAGAATTGTCCACAGTAAGGAAGTGAAGAATCTAATAGAAAAGAAATTAAAAAAATTAGATCCTCATGCTTTAACGGATAAGGGGAAAATTCGTTCTTATAAAATAGATGATAAGAGCATTCGTCAC

>c153_g1

GAAACATTATGTCATATATAATTGACACCTTGTCCAGTGAAAAAGAAACCTTGCTTTTACAAGATTTCTAAGACCTATTTATACCCCCTGCAGGGAATTTTCTTATTAACCCAAATTTGTGACTCCTAAAATTTTCGTTATATAGTGCTGCTCTTAGATTTGTGATAAAAATAATATTAAGCAGTACCTAAAAATTCCTGGTAAAGATACATATTGGGTTTCGAAACCATAAAAATATCTATGTGTATATCACCCCAACTGTCTAAAAATAAGCTTAATGCTCCTAACTTCCTCTAAAGTAGGTTATTAAAAAATTATCTTTCGCTTGATGAACTAGAAAAATCTACTATAAATTATAGTGTTTTCTACAAAAAACGAATTAAAAGGACCGAATCCTGTTCAATATAGAATTCAATCCTTTCAATAATTATTGATCCAACGTTGAAATCTTAGTACAGAAACCTAGTTTTTCAATAAGATGTCAAAGAAGATTACTTTTCCTGCCTATTACTAGCTCCTTCGGAGCCCTCCTTTTGTCCCCCTCTCATTCCTTGAATTATTCCAAATAGCGAGGGAATGCGATCTTCTTTTTCTCCAGTAATGGGATTATAACCTGTCGTACCGATAACAACATCCAAAGGCTCTACATCTGTTTTAGATCCAACAATATTATTTGCAATAATAGCAATTCGACCAACAGATTCAGGCAGAGGAGAACTATCAGGAACGGCATCTGTTCCTGATAAAGTCTCTCCAGTAAGTCCAATGTAGATATTATTTGCTACATTATGGGTTAAATTATCAGCAATTATACCGATGTATCGTGCACCCGTTTCGCTCGTTCTAATCACCCCTGTTCCAAGAACCGGAGAAGTTCCTTGTGCTATATTATAATTTAAAGATCCGATAGGGGTTAGAACTTCTTCAGGCACTGGCTGAGGATCAGGAGTCACGTATGTTCCCAACTCAGTAAACGTCACTCCGGCCAATTTTCCTAACGTGCCATCAAATACATCATAAACTTCTTCTCCAACAATACCCAAAGCAGTCGTTGGAGTAGAAAAAGGCTTTCCAGTTTCTGTCCCCCACATATTAGGGTTCCTTCTTTCTTCTGCATTTGTTGTATTTGTTGCAGAGACAGTGTAATCAGCCTCCAAGGGAGAGTAATTACCCCAAGCATCGTTTCCCAAAACGTTAAAAGTTTCATTTGAATCACCTGTCAAACCGCTGCCAAGGTCAGCAGAAGATTCGGAGCCA

>c155_g2

GCCAGCCAATCTCTTCATTGCCGCCTCCAGCAAAGGTGACAAAGGTCCAGCCCAGGGGAAACATCAGAATGGCATTGAGCGGCGGTAATACAGGATTAGCGATAAAAAGGGTCAAAACCCGAACCAGACAGAAGACCAGCATGTAAATCCACCAGCCTTTGGCATGGGAGAAGATAAAGTCCAGCATCTTCTTGGGATGAGAAATTTTGAGGCTGATAAAAGTTCCCAAGGTCGGTCCGAAGCCCCCGATGAAATTGAGAGCAAAGCTCAAAGGCTCAGCCCCGCTTGTCAGCTTCAGGGCGGTTAAAATATACTGAAGCAGCCAGGCTCCCCAGGTAATGCCAAAAGTCCAGGCCAGAAAGGGCAGGATATGCCTTGTCTCTGGCTCTACATGTGTTTGTGTTTTCATATGATTACTCCTTTTTCTTTGGCAGAAGAGCGATTGCCAAGATTTTTAGCCTGCATCTTTCTGAACTCCTTTCCGATTGCCCTTCCAATACCACCAGCCGACCAGCAGGGCGGTCATGACCAGATTAGCAGCCTGGAAACTGAGATACCTTCCGCCATTTGTAGCACTGCCGACAATCGTCGCCTGAGCAAAGTTAATGCAGCCGTGAAAGACCATGCAGGCAAAGACAGAGGCCGTCTGCTTGTAGAGAACCGCCTGACAGAAACAGAGCAAGATACCAAAGGAGAGATAGAAGGGCAAAGAAACCTGACTTTGACTTGTTCCCGGAATCAGCCAGAGGGGCAGATGCCAGGCCACCCAGACCAGAGCCGTGATGACAGTTGCCAGAGGGAAGCAGAATTTTTTCTCCAGAGCCGGCTGCAGGAGGCCCCGCCAGCCAATCTCTTCATTGCCGCCTCCAGCAAAGGTGACAAAGGTCCAGCCCAGGGGAAACATCAGAATGGCATTGAGCGGCGGTAATACAGGATTAGCGATAAAAAGGGTCAAAACCCGAACCAGACAGAAGACCAGCATGTAAATCCACCAGCCTTTGGCATGGGAGAAGATAAAGTCCAGCATCTTCTTGGGATGAGAAATTTTGAGGCTGATAAAAGTTCCCAAGGTCGGTCCGAAGCCCCCGATGAAATTGAGAGCAAAGCTCAAAGGCTCAGCCCCGCTTGTCAGCTTCAGGGCGGTTAAAATATACTGAAGCAGCCAGGCTCCCCAGGTAATGCCAAAAGTCCAGGCCAGAAAGGGCAGGATATGCCTTGTCTCTGGCTCTACATGTGTTTGTGTTTTCATATGATTACTCCTTTTTCTTTGGCAGAAGAGCGATTGCCAAGATTTTTAGCCTGCATCTTTCTGAACTCCTTTCCGATTGCCCTTCCAATACCACCAGCCGACCAGCAGGGCGGTCATGACCAGATTAGCAGCCTGGAAACTGAGATACCTTCCGCCATTTGTAGCACTGCCGACAATCGTCGCCTGAGCAAAGTTAATGCAGCCGTGAAAGACCATGCAGGCAAAGACAGAGGCCGTCTGCTTGTAGAGAACCGCCTGACAGAAACAGAGCAAGATACCAAAGGAGAGATAGAAGGGCAAAGAAACCTGACTTTGACTTGTTCCCGGAATCAGCCAGAGGGGCAGATGCCAGGCCACCCAGACCAGAGCCGTGATGACAGTTGCCAGAGGGAAGCAGAATTTTTTCTCCAGAGCCGGCTGCAGGAGGCCCC

>c15_g1

GCTCTCTCTGCTTTTCATAGTCTGCTTCCTTAAAAGAATCCATCGAAAATAAAAATTCACCCTTGCTAGAAAAATAGAAGACTGGAATCCCATGGCGAGATAAAGATTTCAGTAATTGTGTTGATAACTGAGAATTACCAAATATAAGAATGTTATCAATTAGACCTAATGAAATAGCCTTGAGCATAGTGCGTTCTTGGTTCTTTATCATTAACTTACGATCACTAATTGATAAGCTGTATGAAGAGTTCTGAATATACAAATCGGCCATATTAAAACACTTTCTTTTTATTTAATATTTTAATATACCTATTTTAAGTTGTCAATAAAAACCCTTGCAAAATCTACAAGAGTCCTTTCTTT

>c168_g1

TACGCTCTCCTTTCCATCTGCTTATCTTTATTATAACAAGAAACTCCCGAGAAACAAGGCTTTTTCAGTAAGTGGTAGATTTTGGGCGGTAAGTGGTTTGCTGCTAACATTGACAAGAATCAAACATCAAAAAACCGGGACGAGACCCGGTTTTTTTGACATGATGGGAGAGATTATTTTTTGTAACTATTACTATGTCCTTCAGAGGCTCCCTTTTGACTTCCCTTTACTCCTTTGAATATTCCAAACAGCGAGGGAACTCGATCCTCTGCTTTTCCAGTAATCGGATTGTAGCCTGTCATACCAACAATAACATCCAGAGGTTCTACACCTTTTTTTGCTCCAACAGCATTATTTCCAATAATAGCAATTCGACCAACTGCTTCAGGCAGAGGAGAACTATCTGGAACAGCATCTTTCCCTGTTAATGTCTTTCCAGTAACTCCAATGTGAGCACTATCTATTATATTAGATGTAAATAAACTATCAACAACTTTAAGTCCATATCGCGCACCTGTTTCGCTTGTTTTGATTACTCCTGTTCCAATAACTGGCGGGGTTCCTATCATTGTATTATAATTTAAAGATCCAGTTGGTGTTAAAACTGAGTCAGGAATTGGCTGGGGATTTGGAGAGGTATATGTCCCCATTTCAGTAAGCGACACCCCTAACACTTTTCCTGCCAAGCCATCAATCTCATCATAAACCCCTTCTCCAACCTTACCTAAAAAAGTCGTTGGTTTAGAAAAAGGTTTCCCAGTATCTGTCCCCCACATATTAGGATTCTTAGTTTTTTCTTCATTTTTGGCCTTGGCAGCAGAAACCGTATTACTAGTGGCAGGAGCATGCCTAGCAGCTTCCAAAGCCTTCTGGACATCAGAATATCCAAACGTTTGCTGAGGGATATCTCCTATGGGAGTATGCACTACCGGCCCTGCAAACATCGCACCTGCTTGATAGTAGTCTTCTGGTGTTCGACTAAAAGTTTGAGATAAGCCTAAGTCAACCGTCGTTGGAAGAGTTTGGTTAGTGAGGACATCCTCTGTAGCCAAAGGATTGTTAAAAAGGGTTGAAGTAGGCTTACTTTCGCTAACTCGTTGCCAATCTCCGTCTAAAAATGAACCCGGCAATTGATTTTTTCCTGTTGCTGTCAGAGACGATGCATAGTCTAAAGCAAAGCCTGGGAGTCCTTCATTACTCGTGGTTGTCTGCTTGAAGCCTGCCCAATCAGCCAACGCCTCTGACATCGTTGGGGTGTTAACAGTTTTTGATTTCTCAGAGATATCAATCTGCTTCCAATCACCCTCGACTGGATTAAAGCCAGACCATTGATTTACTGATGATGTGCTAACAAAGTCCTCTGTAGGTTTTGTTGCAGAAAAAGTATATTCACCCTCCAATGGAGAGAGACCTGCTGTTCTTGACGCAGCATCCTGAACACTGGCCCAATCACCAAGTGTCAAATCACTTATAGACTTACTTTTGTTGGAAACCTGGCTTCCAAATGCATCGGTTACACGAGTCCAGTCTCCTGTTGTAAAATCATCTGTTGCCGTGATAGTTGACGAAGCCGAATTGTTTAAAGAATCATTGATTCTAGACCAATTATCTGCCCCCAAATCACTATAAGCAGCGCTTGTTGAAGTTTTATTGTCAGTAAAATCTGATACATGCGACCAGTCACCTGCTGTAAAGTCGTTCAGACCTAAGTCGCTTGTTGAAAATTTATCGCTTGATGAATCAGATACTCTAGACCAATCGCCTGAAGTAAAGTCACTCACTGTCTTGCTGGTTGACGAAGCATTATCAGCCAAGGTATCAGATGTTCGAGACCAATTACCTGTATCAAAATCACTAAAAGAGTCGGAAATCTCGGAAGATTTCTTAGCGGCTGAGTCGTTGGTGCTCGTCCAATTTCCTGCGCTCAAATCTGCAGAACTTGTATTGCTGTCAGAAATACTAGACCAACTTCCAGAACTATAATTGTTGCCCGATGAAATAGCTTCACCAAAGCTAGAAAAGTCGCTGTTATCCTTAGACGAACTAGTATCCCCAATACTGGACCAGCTTCCAGATCCATTTGACAAGTCGCTGTCGCCAATACTTGACCAATTCCCAGAACTATCTGACGATCCAAGGCTACTATTTGAAGTAGAGTTTCCAAGACTATCATTATCTGAAGTAGAATAGCCACCGAAACTGTTAGAATCTGAAGAGCTAAAACTTCCAGAATCTGAGAAACCTAGACTATCACTATTTGAAGCAGAGGAGCCATAACTACCAGAGTCTGAAGAACCTGAACCGAATCCACTAGAGTCCGAGGAGCTGGAACCGAAACTGTCGGAATCTGAAGAACTGGAGCCAAAGCTACCGGAGTCCGAAGAACCTGAACCGAAACTACCAGAGTCCGAGGAGCTGGAACCAAAACTGCCGGAGTCCGAAGAACCTGAACCGAAACTACCAGAGTCCGAGGAGCTGGAACCAAAACTG

>c171_g1

CTTTTCTAAATAGCTAGCTGGAACATCAATATGGTCTTTAATATTTTTGGTGACTGAGTCTTTGACTGATTCTTTTGAAACAAGGACATTTAAACTAACAGTTACATTTTTTGTTGCTACAAGATTAGTCGAATCTGGTTTACCATCTTTAGCACCATAGACTTTGACAGTCGCCTGGTAATCATGAGTGCCATTGATGCGAAGTTGGTCAAGAAGGGCTTGTCCGTCTTTACCTGTAGTATTGCCATTCAAATCCACTTCGTAGAAGTATTGACCTTTTGCCAAGCCTTCAAGCGGCAATAGAGCAGGATTGTTCGCTTTGCCATTGTCTGACCAAGGAGCCTTGTCAGAAGATTTGAGTAAGAGGCGCGTCAACATGCCGTCTCCACCGAAAGCTTCATACGGAATAACTTGATTAACACCTGCCAAGAATGGACCAGGAACGTTTGCTTTTTCTAAATAGCTGGCCGGAACGTCTACCGTGTCCTTAGTATTTTCTTCAACTCCTTTTCTAACTTCATCTGGAGTCGTTACCGATTGAGAAGTAGCCGCTTCACGATCTTGGTTTGAAACTTCAACAAGAGTTGCCACCGGTTTAGCTTCTTCTTTTTGATTGCTAACTTCTGCCATAGTCTGTTCTTCTTTTGGTTTTACCATTTCTTCCTTAGGAGCTGACTGATTTGCAGGTAGAGCCGAATCCATTTCTTCTTTTAAAACTGTCTCTGGCAACTTATTCTTTTCTAATAATGACGAATCTTCTTGAAGAACTTTAGTAGGAGTTGAGGTGAGATCATCTGCATGTACGGTATTTGGTTGACCAACAAGCAAAAAGAAACCACTGGAAACAACGACGGAAGCCACACCCACACTTAAACGACGAATTGACCAGCGGGTATATTTTTGATTTGAATTGAATTTCATAAGATTCTCCTTTAGGTTTGTTTTTTTATGACTTTAGTATAATCTCCTAAGATTAAAAATGGTTAAGAAAAAGTTAAAATTTTTCTTAAATCTATCTTATAAAAGGATTATATTGTCATATTGCTCATAAAAAAACAGCTTGAATTCCTTCGCCATAAATTCCCTACTATAATTGACAATGAGTCAAAAAAATCCACCACATTGTGGCGGATGTTATCTATTAGCTCTCCATCTCAAAACGCTCACTCTTCTGCTTGTTTGGCAAAAAGAGAGAAAGTAGGATTCCAACTAGAGCTGGCACCAACCAAGGCAGGGAAGCCCGAGCAAACGGAAGAGCATTTACCATATCATTTACCTTTGTAATATGGAACTGCTGCCCAAGAATAGCCGCAAAGGAAATCAGAGTCACTGCTGCCATCGTCAATTGCATACCGATTTTGGACAGTGGCAAGAATTTATTGACAATAACAATCAGCACGATAACAATGGTAATTGGATAGAGAATTTGCAAGACTGGAACAGAATATTGAATAATAGCATTGAGCCCAAGATTGGCAATCGCAAACCCAATCAAAGTAAAGACAGTTGCGTAAACTTTATAGGATACCTTAGGGAAAGTTTTATGGAAGAATTCCCCAGTTGATACAATAAGTCCCGCAGTTGTTGTAAAACAAGTCACAGTAACCATAGCTGCAAGGAAAATCTGTGCTGTAGGTCCAAAGATAGCCTGTGTCGCCTGCGACAGGACATAAACACCAGGATTGCCTTTGGCAATAATCTCAGCTGGAACTGGGAAATGATTGCCTAGAAAAGCCAAACCGATATACATGGCGCTAAAGCCCAACGCTACTATCAAACCAACAACCCAGATGGTTGAAACATACTCCTTTTTATTCTTGAAGCCTAGCTTATTTAGAGTCGTTACTGCAATTACGCTGAAAGCCACGGAAGCCAAGGCATCCAAAGTATTATAACCCTCCAAGAAGCCCTGTCCAAAAGCTGACGCTAAATAAGCTTCCGAAGCCTGCTGAGGAGCAGTTGAGCCGTATTTCAAAGCTCCTAGAATGACTAAGATAATAATCAAAACTGCAAAGACTGGCGTCAGAATACGTCCAATCCGATCCAAAATCTTAGATGGATTCAAAGAAATCAGGTAAGCAGCCGCAAAGTAAAGGGTCGTGAAAACAATCAATCCGATACCAGTCATATTTTTAGACAGCATAGGAGCAATACCAACTTCATAGGCCACCGTCGCTGTCCGCGGAATAGCAAAAAAGGGCCCAATTGACAAATACAAGGTTACAAGATAGACAATAGCAAACCAAGGCGCAATCTTCTGAGAAATCTCGTGAATATAGCCCTTGGGATTGAGCGTTCCGATAATCAAAGTCAGAACAGCAAGTCCAACCCCAGACAAGACAAATCCTGCAATAGCTGGGCAAAAATGCTGGCCAGATAATGTTCCAAGCGAAGGCGGGAAAATCAAATTCCCAGCACCGAAAAATATTCCAAATAAGAGCAAGCCTGTCAAGGCACCTTTTTTAATCACGGAAATCTCCTTTAAATTTTTTCATGCTTTATCATTATATACGTAAATAGCCCGCTCCGCAAGATTTTTAACTTTTCACAGTAGGTTGGTTTAAAATCAAAAACAATACATCGAATTAAATCGAATAATTTATCAATGGTTTTTAGCAAAATCATCACTTTTCCACAGATTTAAGGCTTATTTTAAATTATTTTAGAATAATACAAATAAATTTGTTTTAAATCGTATTTATATAACTTCACTATTACCTATCTTTTTAAAAGTTTCCTTACTTTAAATATATATGCTCTACTGTTTCTGAATGCGAGAAAAATACAATATTGTCCTTCCAGATCCAAAACAAAATAAAAGAGATTTTTCATAGTAAAAAGGTTGAGACATCCTATCTCAACCTTTTAAAAATGGCTATAAATCATTTAGCATTCTTTACACATCGCTAAATCTTCGGTACTTAATTTCGAATTCAAAGAAGTTCTTCTCTTGCAGCATATGGAAAATATCTCGATAAGCCTCCTCATCAAAATGATGTCCCAAATACAAGATTTCAAAGCTTAACCCTTCATATTCCAGAAAGGCATTGGTCGTTCGAAAGCCATTACGCTTATAGAAGTCCATACGTGCTTGACGTTGCTCTAAATTGTCACATTCTTCATCGACACGTTCTACCTCTAGAACCATAGTTTTTTGATAAAACTCTGTCAGTTTATGAATGATTTCTCCCCCATAGCCATGGCTGCGAAGGTGCGGCATAATCGCAAAGAAGCTGACATAGAACATTTTTTCGTTATAAACTGCAAAAGCAAAACCAACAAATTCTTCCTCATTGTAGAAGGCAAAGAAATGAGACCGTTCATCATTGGTATAGCGGAGAAATTCATTAATAGGAACTCTCTCTTCTTCAGGAAAGGCTTCTCGATTTAAGGCCTCCACCTTATCTAAATCAGGAAATTCTGCTGTAATAATTTGACTAGTTAAAGACATTGCTACCTCACTTTGCTTATATTCTAGCAGTTATCAAATGATTTAGCAAACCTTTTAAACTTCTTCTGCTTTTTTAGAAAATTGACTCTGGTAAAGGTCATAATAGAATCCTTTATCTGCCAAGAGGCTCTCATGATTACCTTGCTCGATAATCTGACCATCCTTGAGCACCAAAATCTTATCCGCTTCTTGAATGGTCGACAGTCGGTGGGCAATGACAAAACTGGTCCGACCCTGCATGAGTGTCTTCATAGCTTTCTGAATCAGGAGCTCCAGCCGTGTATCGACAGACGAGGTCGCTTCATCCAAAATCAGAATCTTAGGATTGGCCAAAAGAGCCCTCGCAATCGTAAGCAACTGCTTCTGACCCAGCGAGATATTGCTGGATTCTTGGTTCATTTCCATGTTGTAGCCGCCTGGCAAGGTACGGATAAAATGATCCACATTAGCAGCCTTGGCTGCCTCTACAATTTCTTCATCAGTGGCTTGCAGATTGCCAAAACGAAGGTTTTCCTTGATAGTCCCTTCGTAAAGCCAAGCATCCTGCAGCACCATACCAAACTGTTTGCGATAGTCCTGACGGGACAGATGTCGTATATCATGGCCATCCACCGTAATCGCGCCTTTGGTCACATCGTAGAAACGCATAAGAAGATTAATCAGCGTGGTCTTACCAGCACCAGTCGGACCAACAATAGCCACCATTTCACCTGGTTTGACTTCCAGATTGAAATTTCGAATGAGCGGTTTGGCTGCCACATACTGGAAGTCCACATCCTTGAAGCTAACCTGACCTGTCAGATCTTGATCCAGCTTTTCTGTCTCATCATTGACTTCGTCTGCTTCGTCCAGCACTTGGAAAATCCGGTCCAAAGAAGACTTGGCACTCTGCAGCTGACCAGCCAGCTGAGTCAGGTTTTGAATGGGTTGGTTAATCTGCCAGACATACTGAACAAAAGCCTGCATATTCCCGACTGTCAAGCGCCCAGCAATAACCTGAAGGCCGCCCAGCAGGGCCAACAAGAGGTAGGTCAGATCTGAAATAACATTCAAAACAGGCATCATTAAACCAGAGATAAAGCTAGCTTTAAAACCTACTTTCTGCAGTTTTTGCGTAATTTGTCGAAAATCCTCCTGCGAACTTTCTTCTCTGACATATAGCTTCAAAATATTAAATCCTGTCAGATTTTCCTGAACAAAGCCATTCATAGCCCCCAGTGCATCTGCCTGCTGTTTAAAGTAAGGCTGGGATTTCTTCACGATAAAGCGAGCACTGAGGTAGGTAATCGGAATCGAAACTACCACAATGATACCCAACTGCAGATTGAGCACCAATACCATGACGATAACCAAGATCAGAGTAAAGACTGCATTGATAACCTGCAGAAAGGACTGCTGCAAGGCGTTTGAAACAGCTTCGACATCGCTGGTAAAACGCCCCAGCAAATCACCAAACTGATGCTTATCAAAGTAAGAAACGGGGATACGATTAATCTTATGGCTAAGTTCATTGCGCAAGTCACGAATGGTAGCCTGCACTGCATTGGTCATAAAGTAATTAGAATAGTAAGAACCGATTTCATAAATAATCGCCCGTACAAAATACAAAATCATGACCCAGCCGACATAGGACACATTAATCTGGGCGCCAGCTACACCTTTAGCCATATCCAGCAGATTATTTGTCAATTCTGTAATGGCTAAACCTAATACAAAGGGCTCGACGACACTCATAACGACACTGAGAATTTTGAGGAAAATCGCAAAGGCAACTGCAAATTTATAAACTTTTAGGTAGCTCCACAGACGAGCAAAACTAGATGTGTTTTTCATGTCAGTCTCCTTTCTATTCCTCTGTCAAAGCTTGATTTTTCAGCTGCGAATCCGCAATTTCGCGATAAATATCATTGGTTTCCATCAGTTCTTCGTGCTTCCCGCGTCCGACGATTTCACCCTGATCCAAGACGATAATTTGGTCAGCGTCCATGATGGTTCCCACACGTTGGGCTACAATCAGCACCGTTGCCTGACCTGTCACTTCCTTGAGACGACGACGCAGCACCGCATCTGTCTTATAATCCAGAGCAGAAAAAGAGTCATCAAATATATAAATATCCGGTTTTTTAACCACTGCACGGGCGATAGAGAGTCGCTGTTTCTGTCCACCGGATAAATTGCTGCCTCCCTCAGCCAGATGAGTCTCAAAACGCTCTTCTCGACTCTCGATAAAGTCCTTGGCTTGAGCAACCTCAGCCGCCTGACTCAGCTCTTCCTGACTAGCCTCTTCCTTTCCGTAGCGCAGGTTCGCTGCGATAGTTCCGGTAAATAGCAAAGCCTTCTGCGGAATAAAGCCAATCTTCTGACGCAAAGCCTTCAGATTGTATTCACGGACGTCCACACCATCCACTAAAATCTTACCTAGTGTTACATCGTAAAAGCGCGGAATCAACTGTACCAGTGTAGACTTACCAGAACCAGTTGAGCCGATAAAAGCAATAGTCTCACCCGGCTTAGCCTTGAAGGAAATATTATGCAGTACAGGACTTTCCGTCTCACCTGGATAAGCGAAGGTTACATTGTCGAACTCCAGATACCCCTGTGTCTCTGTCTCGGTTACACCATCTTCATTAGGATTGATTGAGATGGGCATATCCATGACTTCCTTAAGCCTCTGGCTTGATACCGCAGTCCGCGGATACATGGTAAAGAGATTGGCCAAGAAGAGGAAGGACAGCAGGGCGTGAAAGCTGTATTCGATAAAGGCAACGAGATTCCCGATTTCAAGACTGCCGTCCCGCAAGGGGTCTAGGGCAAACCAGACAATGGCCACAATCATCGCAATAATAATCTGCACAAAGAGTGGCTCTGTCAGACCAGTCAGCTTAAAGAGCTTATTGGAGTTTTGCGCGTAGACCTCATTTTCGTCAGCAAAACGCTTCTCTTGAAATTCCTCTCTTGCAAAAGCTCGAATAACGCGTAGACCAGTCAGATTTTCCCTCACATACTGATTAATCTTATCAAGGGTCTTCTGCTGCTTTTCAGACAAGGGCTTGGTTTTAACCGCCACATAAATGACCACGACAGCCAAGAAAGGCACTGAAACAGCTACAATCCAGGCTAGAGATGGGCTGGTCAGAAAAATCATGAGAATACTGGACAGCATCATCATGGGAGTAATGACACCCATTTTCAGGGTTTGCTCTGCAAACTGCATAAGCACAAAAGCATCCGAAGTCAAGCGCGTGACCAATGAAGACACACCGATTTGCTCATACTCATGATGAGAATACTCTTGCAGCTTGGCATAGAGATCATTGCGCATGTCCTGCACCATGGTCGTCGTTAGCTTTCCAGCCGCATAAGCCAGAACGATTCGCCCCAGCACTCCTAAGATAATAACGCCAAACATCACCCAAGCCCAGAAAAAGAGCCGATCTGTCTGCTTAGGATTGATTCCCTCATCAATCATCCGAGCCAGCACCGTTGGCAACCCTAGATTGACAATCACAAAAAAGATTGCCGCAACAAAGTCCAAGACCAGCCACTTAGGATATCTCTTCAAATAAGACCAAATGTATAACATAACTCCTCCTTCACCATCTATTCAAAAATAACTCACTATAAAGCACCCAAGTGCTTTTAAAACACAGAAAAG

>c174_g1

TGATGCAGATGATTTAGGTCACTTTTCTACAGATTCTGCTGGAGATAATCTAACTACCTCGGATTCTTCTACTGACTTGGGCAGCGGTTTGACCGTTAATCCGGATAGTTTAGGAGACTTCGCTTCAGATATTCCAACTATTGATTCTTTGGATGCATCAGCAAATGATGGTGATAATTTGACTAATCTGGGGAATGTTTCTACCCCAGTTTCTACAGAATCAACCAATCCAACAACGAGCACAGATGAGATAATGGGGGGCACACTAGATGCAGCAGTAGCGACCGCTCCCGCACCCCTTACGGATGCAACTAATTCTGACACTACTATAGATGAAACAACGGCTTCCTCGACTCCTATAGATTCAGCTAATCCAACAACTGATTCAATAGACTCGGCAAGCTCTGACAAGCCTAATTCGATGGATTCGGCAGTTGAATCAAACACTGCAGATTCCGCAGGCAATGGTTTGACAGCTAATCCGGACAGCTTAAGCAGTTTCGTTTCAGATAAACCAGCCGATAGAACTGGCTCCGAATCTTCTGCTGACCTGGGCAGTGGTTTGACTGCAGATGCAGATAGTCTAGGTAGTTTTATCTCAGAATCAGATTCT

>c177_g2

GAATATCAACCTGTTGTCCATCGACTACGCCTTTCGGCCTGATCTTAGGCCCTGACTCACCCTCCGTGGACGAACCTTGCGGAGGAACCCTTGGGTTTTCGGGGCATTGGATTCTCACCAATGTTTGCGTTACTCAAGCCGACATTCTCGCTTCCGCTTCGTCCACCGCCGCTCGCGCGGTTGCTTCCCCCTAAGGCGGAACGCTCCCCTACCGATGCATTTTGACATCCCACAGCTTCGGCAGATCGCTTAGCCCCGTTCATCTTCGGCGCAAGAGCGCTCGATCAGTGAGCTATTACGCACTCTTTCAAGGGTGGCTGCTTCTAGGCAAACCTCCTGGCTGTCTCTGCACCCCTACCTCCTTTATCACTGAGCGGTCATTTAGGGGCCTTAGCTGGTGATCCGGGCTGTTTCCCTCTCGACGATGAAGCTTATCCCCCACCGTCTCACTGGCCGACCTTGACCCCAGTTATTTTGAGGTCATATCTAGTATTCAGAGTTTGCCTCGATTTGGTACCGCTCTCGCGGCCCGCACCGAAACAGTGCTTTACCCCTAGATGTCCAGTCAACTGCTGCGCCTCAACGCATTTCGGGGAGAACCAGCTAGCTCTGGGTTCGAGTGGCATTTCACCCCTAACCACAACTCATCCGCTGATTTTTCAACATCAGTCGGTTCGGACCTCCACTTAGTTTCACCCAAGCTTCATCCTGGTCATAGATAGATCACCCAGGTTCGGGTCCATAAGCAGTGACAATTGCCCTATGAAGACTCGCTTTCGCTACGGCTCCGGTGGGTTCCCTTAACCAAGCCACTGCCTATGAGTCGCCGGCTCATTCTTCAACAGGCACGCGGTCAGAGCCCAGGGCTCCTCCCACTGCTTGGGAGCTTACGGTTTCATGTTCTATTTCACTCCCCGATGGGGGTTCTTTTCACCCTTCCCTCACGGTACTACTTCGCTATCGGTCACCCAGGAGTATTTAGCCTTGCAAGGTGGTCCTTGCTGATTCACACGGGATTCCACGTGCCCCATGCTACTCGGGTCAGAGCATAAGCTAGTGATGCTTTCGGCTACTGGACTCTCGCCATCTAGGGTGCGGCACTCCACCGCTTCGCCTAGCAGCACGACGCTTTTTTTGCTCTCCCACAACCCCGTTTTCACGGTTTAGGCTGCTCCCATTTCGCTCGCCGCTACTACGGGAATCGCTTTTGCTTTCTTTTCCTCTGGCTACTAAGATGTTTCAGTTCGCCAGGTTGTCTCTTGCCTGCCCATGGATTCAGCAGCAGTTCAAAAGGTTAACCTATTCGGGAATCTCCGGATCTACGCTTATTTTCAACTCCCCGAAGCATTTCGTCGCTTACTACGCCCTTCCTCGTCTCTGGGTACCTAGGTATCCACCGCAAG

>c178_g1

ACAGCCTGCTAAGAACAGTCCCAGACCGGCAAAGAGTATGTAACGCCAAGATTGAACAATTGAGTAATCCTGATTCATGGCAGCCAGTAAAAGCAGAATGAGATTGCCTGCAAAAAGGATCATTCCTAGAGAGGTCAGGCAATTCCAAACCTTCCAAGAGCGAGGTACCGAACTTTTTGCTTTACGCAGGATGAGACGGAAACCGCCAATCAGAAAACTAATCAAGACATTTCCCAGAGCAAACACGAGCGCAAGAGCTCCGAGAAATACGACTAGATAATGCCTAAAGAGAACTGAATCGGAAATCCTTTCTGCATTAAGGACACCAAATTCGAGGCGGTCAGCACCTTGACTTCTGTCTATGGTCCAGAAAGTGCTCAGCAGATCCGGCTGATCTGAGACTTTGTTGATCTTTTGGATGGCCCCGGGAATCATGAGCATGAAGGACAGTGGCCCCCTATTATAGCTTCTCAGATAGTGGTAAGAGCCAGACTTGAATTGCTTTTTAGTAGCTTCGCTGACAGTCTGCATCTTGCCAAATATCAGCTCCGGCATTTGAACATTGTAAACTTGTTCATACAGTTGATTGGTTAAGATGACTTGGCCGATGCCATTTTTCAGATCCAAGTAGAGATAGCTAGAAAAGCCATCAGCATTACCACTGTGTCCCAGCAAGGTAACACCGTAGTGACTGGCCCAAAAGCCATGAGCATTACGAACAATATCCGTACCAGGGTGGGTAGAGGTTGTTGAGTAAAGCTCGGTCCAAGTTTCTGGACGATGAAAGAGCGTCTTACGTTCCAGCAAGGCCTGGGCGAATTTCTGCAGGTCTCCTAGAGTCCCCACAGCCCCTCCGACTGGGTACATCCAGAGCTTAAATGGTGTATCTCCCAAGAGCTTACCTTGGTCATCATAGCCCTTATCCTCCTTGCGCTTTTCCTGTACATAAGCGTTGTCCGATAAGTCAGGCAAGATAGCAGTCCGATCCATCTCCAGCGGCTCAAAAACATGCTCATGGACATAGTCTGCATACTTCTGCCCAGATATCCGCTCCACGATATAAGATACCAAAGCCGTGCTGAAGTTAGAGTATGATGTCGTCGTACCCGGCTCAAAGGACTGAATAGGCTGGTAATCACGGAACTGCTCTTCCAAGTCGCTCTTGTCTCCCTTTTTATAGAGTGGCATTTCATCAAAGCCAGCCTGATGGTTCATCAGATCCAGCATGGTGATAGGCTTATCATAGCGAAGATTGCGGAGAAAGCCTTCTGGCAGATAAGTTTTGATGTCTGCTTCCAGGTCGATTTTGCCCTCTTCCCAGAGCTGCATGGCCGACACCCAGACCGTCAGTTTAGTGATTGAAGCCCATTCAAAAACACTGTCATCGTCAACTTTGACTTTGTTTTCCTTGTCCACATAGCCAAAATTTCCCTTGTAGATGGTTCCGTTTTTATCAAAGACTGCTGTTGCCATGCCGGCCGTTGTCTTTTCATGTTCCTTGACATAGTCTTGGATTTTCTGGCCGATCTGGTCTCGTTCGATACCAGAGGGCAGCTTTTGAGCCTCCTCAGCCAGCACAGTAGCTGGCTGGAAGAGTCCTAGAGTTATAATCG

>c179_g1

CAACTTCTTGACCGTTAAAACTATAGACACGGATCTTGATAGTCCGCTCACCTTGAACTCCCGGAGTCGCAATTTGACGGGCATCGCGAACCAACTCGTCCGTCTCTTCTTCACGAATGGTAAAGTTGATTTTCTCTACCCGAGTTTCATCTGTATAAGTCAACGGGTATTCTGGAAGAGCGTCTGCTTTCGGCGCATCGTTCGGTACCATGTGTGGCTTAGCAGTTCCAACTTTGACAACTTGTGTTACTGGCGCTAGAGTCTCCTCGTTGGACAATTCTTGGCGGTCAACTTCTTGACCGTTA

>c179_g2

ATTATTTTTTTAATCAAACTCGGATGGATTATTATTGTTTTATTAGAAATAAATAGCAGAAAAAATACCTAGACTTTTTACAGTCTAGGCATTTTTAAATATATACGGAAAACAACTTATTGCTTACTTATTAAAGATTGACTGTCTGAAGTCGTCTGTTTCACGAAGATAAGCATTGTAAATCTTCTTCTTAAGCAGGTTGACCCAGCTGGTATCAACGCGGCTTGTAGCATTGGCAATGTTGCTCACATCCTCTGCTACTGCTGCGTCCATCAGCCTTTGCATGTCTGCATAAGAGCGAATGGTTACTTTCTTAGTACTGTTTGGATTTCTGAGTTCGTACTCGATGGTGATTGGCTTGAGCTTGGTCAGCTTATCAATCCGCTCCTTATACATAGCTTTCTTAAAGGCTACCCAGGAGTCATATTCACCCTTAAATACATTTTCCAAGACTTTCTGGTCTGTCACGAGACCGACATCTCTTCCAGACCATCCGTCCCACGTTTTCTTGCCTTCATCGAAGGCTTCCTGAGAGTACTTACCAGAAACATAAGGGATAAATCCTTCATGGTAACCCTTGGCTGCCAGCAACTCATAGGCCGTCCGGCGGAACATGACGTCACCCGGCGCTCCATTCGGATTGCTCAAAGCTGAGTAGATTGGTGAGAAGAGACTGAGGCTGAGGTAGCCATTTCGTCCATATCTACCACTGTCCTTGTTCTCCCGACGAGTGATGACATCATTTTCAATCAGGGAGTTAAAGGTTTTCAGCTGCTTGATTTCCTCAGCAGTAAAGCTTCGTGTCTGGTTTCCAGCATGGGTTTCCTTACCATACTTATCTGTAACGTAGTAATTCTCCATCTTTCTGAACCACTGGAGCTTAGCATCATCGCTCTGCTTCAGCATAGAGGTACCTTCTAGGTAATCTAGGGTGTAGATCATGTCAAACATACCATGGACATAGTGCTGCAGGTCTTCTGCATTTTGAACGCGGTCCTTGAAGTTATAAGTGTGCAGGCGCGTCTTAGAATCCTTGTCCACCTTGAAGAGGGTATTGAGCGTAATGGTTGCCTCATCCGCACTTGGAGTGGACTGCAGGAGTCCGCGAGCATAAAGCTCAGCTCCCAGACCTTCGCGGCGGCCATTGCCCTCAAAGTAAATGCCACCGTCAGAATTATGGGTCATTTCATGCGTGTAGACAGAGTTACCATAGTCTTCTAACAATTTAGCTGCATCAAAGTGGGTTACACTTCCTGTAGCATAGGCATTATAGCCCTTGCTAGGATACCACTTGCCGGCTGGTCCAAAGAATTCCTGCATAGCCAGTGATTTTTTGTCATTAGCTGGAGCCCAATATTTTTGACCATTCTTGTCAACCAATGAGAATCCATCGTACACCAGAACAGAACGGAAGAGCTTGTCCTTGCTTTCATCGCTTAGAATTTTATACCAGAAGTCATAGTGATTGCGCTGGTATTCAGCTGTTTTTCTCACTCGGTCTTCGACAAATTCAGCTAGCTCTGCATCTGTCCGCACCCTGCCATTGGCATCAAGACGATAGCGATCGTAAGCCCCCATAGAGATGGTAGACATATTAGAAATAGCATAGACGCCCTTGTCAGTCATGGTCAAGAGTGGCAAGAGCATGCCCTTGTGTTCCCAGTTATCAGCAGTAATCTTGTCATAAACGCCGACAGAATACTTACTCTTGTCCTCGGCTGCATCCTGAAGCTGTCTAGCTTCTGCTATATCCGACTTAGCCTCAACGATGTAGGCTTTTGTATTGGTCTTGAGCCATTCATTATTGGTCTTGTCTGGCAAGAAGAGCTGACGATAGCCTTCTAGATAGTTGAAGAGCCCTCGTTTACCAGTCGCTTCAGAAAGTGAGGCATCATAAGCCATATAGTTGTTCTTAGCTTTAAGATTGTTCATGCCTGACTTACCAAGTGAAATAATGGTATCTAAGGTTGAAGCATTATGATTTCCAAAGAAGTCAAATTTATAGGCTGACAAATCCTTGACATTGATATTGTCATAGTTGATATTATACCAACGGTTGAGATAGGTCAGACCAAGTAAGAAGGCTTCTTTATTGTCTTTAATTTGCTGAGCTATATAGTCTGCTACAACATTGCCTTCCGTGTTGATAGACTTGTCCATAGCCAGTACCTTGCGTAACTCTTCTGACAGCTTGGTCTTGACTTGATCAAAAGCCGTATCCAGATAGAGATCATCCAAAGAAGTATCAGCATTTACGCCTAAAACAGTTCGCATAGCCGGTGAGTCAAGGACAACCTTGTTCAACTCCGGCAGCACTTGATTGAGAACTCTGCTATAGTCTGATAGGAAGGCTTCTGGTGTATAAAGAAGGTCTAGGCCATTTACAGTATATTCTGCAATTGCCTTATATTTGAAATCTCCCTTATAGGTCAGATTCAGGTATTCAACTGTATTATCGTCAAAGTGCAACATTAAGCGGTTAATTCCGGTCTTATTGCTATTGATATCAGTGATAATCTGGTCGCCCTTCATCGGAACAACATCCAGCAAGTACTCTGTATTGAGCTTGTGATTATCTGGAAGTTTATTTCCATAGGCAATGATGGTCTCCTTATTGTAGAATGGCAGGAGTTTTTCCATGTTAGCATAAGCTATTAGACGGTCTTCTCGGGCATTCTTTTCTTGGGCATAGTTGACAGAATAAAGATTGAGATTCGTGTCTTCTAGCGTGGTTGTAATATCTAAGCTAGCCAGTTTCTCTTCTGCCTGGTCCAGAGTCAGTGTTGAAGTGACAAACTTATCATTTTCCAGTGTTTTATTTCCTTCAACGGCATATGCTTCTGTGATACGGTTGTTTTCATATCCCTGATCACCAGAAATACTGTAAACATTTGTACCGCTGATGTTGCTGATGACATTATCAATCAAGCCATTGAAGTAATTGGAGCCAACCACACCACCAATCTGACCATTCTTAGTAGAGTTTTGAATCTTTCCAGTTACATAAGAACGGCTGATGCGGGCATTGTTTTCTAAACGGCCAACCAGGCCGCCAAAGCGTTGGTTGTTGGCTCTAGCGCCTGCTAGAATGGTTACATCTGCCCTGCTTTGGCTAATCAAGGAGTCTCCTCCCTTAAGATTGGCAACCAAACCACCGACATTGTATTCCTTACCCTGTTTATCATTGGACACGATAGTTCCAGTAAAGGAGCTATTGGTAATTTGGGTATTGTTAGCAATAACAACCAAACCTGCCACCTGTGAAGCATTTTCTTTTACTTCTACTCTACCTTGGACTGAAACGTCTGTGATTCGAGCCCCTTCTGCATTGCGGGCCAGGGCTGCTGAATCATAGGTTCCTACAATATTAACCTCTTTCAGGTCCAGATTGGAAATAGAAGAACCACTCTTGAGGTTTTCAAATAAAGGCTTAGCTAGATTATAAATCGCGTACTGCTTGCCATTATGATTACCTGTCAGACTGCCTGTGAAGTTCCCTTTGAGGTAGACATAGTCCACAGGTGCCAGACTGACCTCGCTAGCATCCAAATCTGCTCCCAGAACATAGTTGCCAGCCATATTTTGCTTCATAGCGTCTACCAAACCAGCAAAGCTAGTGTAGACATTTTGTTGGCTAGGGACTGCCTTACTGATATAGAAGTCATAGCCAGACTTATAGGCTGTCTCACCTTCTTGGACCAGCTCAGGCAGAGAGACTGTTACTTTATAGACATCCCTGCCATCCTTATGGCTTTCCGCTATGCTGTGAACCGGCAGGAGCATTTCCTTAGATTCACTTGATTTGACTTTGACAAAATAGGTAGACAGATCCATAGGCATAGAGCTCATAGATACGACACGCTTAAACTGGTCGTTTTCTTTTCTGTAAAACTCGACCGAATCAATATCTCGGAAGGCAATCTTCTTGTATTCCAATTCAAAATTGCGGCTGTCCGATTCAAGGTCTGTGAAGCTGCCATTATCCAGTTCATAAGTCAGCTTGGTTTTCAAAGTATAGCCTGTATAGTAGTCTAAGTCGCCAATCTTCAGACTTCCTGCGAAATTGGTAATTGGAAGAGTACGAACAAGCTGATCTCCCTGGTACAATTCTGCAGTCGCTGAACGGAAGGACTGGGTCTGGTCTTCCAGTCTATATTGGACTGTAATCGACTTATCATCAGGATTTTCTGTCAGACTTAGAATGGAAACTGCCGGCTTGACTTTAGGTACTCCATTTAGAGCTTCTTTGGCTGCTGTCAGGCTAGCAAATGCTTGGTTGACCTGCACTTGTGTGGCAGTTTGATTGTTCAGAACTCCTTCTGCCTTGGCTAATTCGTCAGTATAAGCTGTCTGCTTATCTGAATCTGCATTCTTATATTTAGCATTTGTTTTAACTATTTGATTTAAATCGTATTCATTTTGAAGAGCTGTTTTGACAACCTCAAGACCACTTAACTGAGCTTTAGCTTGCTTGATTTGTTCCACCAGTTGGTTAACTTCTGCTTGGCTGGCTTGGGATTGGCTCAGAATCGCTTGACCAGATACCAAAGCAGTATCATAGCTAGATTGATGGCTCTGACTATCGTTGAAATAACGTGCCTGAGCTTTAATCTGATCCGCTTCCGCTAATAGATTGTGTAGTGGAATTAAATCGAACTCTTCTAAGACTGCTGCTTTCGGTGCTTCATTCGGTACCATATTTGGTTTAGCGGTACCGATTTTGACGACTTGCGTTACTGGAGCTAAGGTTTCTTCGTTAGACAATTCTTGGCGATCGATTTCTTGACCGTTGGAACTATAGACACGGGTCTTGATAGTCCGCTCACCTTGAACTCCCGGAGTCGCAATTTGACGGGCATCGCGAACAAGTTCATCCGTTTCTTCTTCACGAATCGTGAAGTTAATTTTCTCTACGCGGGTTTCGTCCGTGTAGGTCAGTGGATAATCTGGCAAAGCGTCTGCTTTTGGCGCATCACTCGGTATCATGGTTGGCTTAGCCGTACCGACTTTGACAACTTGCGTTACTGGAGCTAGAGTTTCCTCATTTGACAGTTCTTGACGGTCAACTTCTTGGCCATTTGAGCTATAGACACGAGTCTTGATGGTCCGTTCACCTTGGACACCTGGGGTTGCGATTTGGCGAGAGTCCTGTGGCAATTCATCTGTGTATTGTTCTTCAATATTGAAGGCGATTTTCTCTACGCGCGTTTCGTCGGTGTAAGTTAGCGGATATTCTGGTAGTTCTTCAACCTTTGGTGAGTCAGCCGGAATCAGACTTGACTTACTTGTTCCGACCTTAATAATTTGAGTGACTGCTGCAAGAATTTCTTCATCCGATAAAACTTGGCGGTCAACCTCTTTGCCATTCGAGGTATAGATACGAGTAGTAATAGCCCGCTCACCCTGTACTCCTGGTGTGACAATTTGGCGGCTGCCTTCTGGAATCTCATCAGTATACTGTTCTTCGACAGAAAAATCTATCTTTTCTCTTCTTGTTTCATCCGTCGCAGTCAGCTCTAATTCTGGGGCTTGATGCACGGGTGCTTCATCTGGTGCGGTACCATAAGTTGTTAGTTCCGGAACTTCTTGGACTGGAGCGGTGTCTGGTGCAGTGCCATAAGTCATTAGCTCAGGCACTTCTTGAACAGGTGCAGTATCTGGTGCAGTTCCATAGTTGGTGAGCTCCGGAACTTCTTGAACTGGCGCTGTATCGGGCGTCGTTCCATAGGTCGTCAATTCTGGGACTTCATTTACAGGTGCGGTATCTGGTGCAATGCCATAAGTCGTTAGCTCAGGCACTTCTTGGACTGGCGCTGTATCAGGCGTCGTTCCATAAGTTGTCAATTCTGGGACTTCTTGAACTGGTGCCTCTTTTGGCACAGATTTTACTGCAGCAGTTCCAACCAACACAATTTCCGAAACTGGCTCTGCAGTTACCTGGTCTGAAACCACTTCGCTTTTTACAATTGCTTTCCCTGCAATATAATTGCGTGTGACGACAGTGCGTGTACCAGCGACACCTGCGCGAATGACCTGAGATTGCCCCTCTGCGAGCTCATTTGAATATTGGTATTCTGTTTGATAAGGCAGGACTTGGGTTTGGCTGCTTTCTTGGCTTTTGAATTCCAGTTCTGGTACTTCAGTAACTGGAGAAAGCCGAGCAAATTCACGCTCTTTGGCAGCAATAATCTCTTTTTCTTGCTCTGTCAGTTTATCTGTTTCAGTAGACACAGGCTTGTTGGCAGTACCCTGATTCCCAGAAAGACTAGCAGATTTACCTGCCTGCTGATCGACTTCGATAGCTGACAAGTCCTTTTGTGCCGTAGGAAGCTGATGACTTCCTACTGCGTTCTCCTTGCCCTGTTCACCGCTTTTCAGATAGCCAACATATTCGAAACCAGCAATCTCTATAGGCTCAGGCAGCTTATCTCCTACTGTCAAATTCAGCCTTTGATTGTAGGCGGCTAATTCAATATTGGTCACTGCAAATACAGAAGGAGCTAGGAGAATAGAGCCTAGACCTGTCACCAGCAAAATAGAAGACAGGTATCGCTTACCATTTCTACCTCTTGCAATTACTAAAACTGCTAAAGTCAGTCCAGCAGCTGCAAAAGTTGCTTCCCACAGACTTGAGTAGCCCGTCTTTGGCAAACTTTTTGCCGAAAGTCCCTGGGTCTTTGGACGGTAAACCAGGTAATAAGCATCAGAGTTTTCTTCCACAAACTTTGGAAGCTCCTTAACAACTGCATTCTTCTCAGCTTCAGTCAGTTCAGACTCTACTACGTAGTGATAGTGAACTGGAACAGTTTTGCCTGCTGAGACTTCTGCAGCCTCAACCGTGCTTATATTTCCCCCTATTGCGGTACTCAAAAAGAAGCTTCCGATAGTGGCAGAAACCAATCCTACTGATAACTTTCTAAAAGAATAGCGCTGGAGCGTATCACCAGTGATTCTTTTTTCCATAATAATTCCCTTTTCTTCATATAATAATCTCTTAAAATTTTACAGAATTTCCTACAAAAATTCAATGCTTTTGCTTAAAAATATATCTTTGAAGGCTGAAATCATCATGTTTTTGAACAGCCTGAAAATACTGTTCATTTTTACTAAAAAAATAAAAAACAAATCTTCAGTTAGAA

>c179_g3

CGGGTACTCTGGAAGAGCGTCTGCTTTTGGTGCATCGTTTGGAACCATGGTTGGCTTAGCCGTTCCGACCTTAACAATTTGAGTAACAGGAGCTAAGGTTTCTTCATTGGACAACTCTTGGCGGTCGATCTCCTGACCATTGGAGCTGTAGACACGTATCTTGATAGTTCGCTCGCCTTGCACACCCGGAGTGGCAATTTGACGGGCATCCTGAGGCAATTCATCGGTGTATTGTTCCTCGATGTTAAAGGCAATTTTCTCAACACGTGTTTCATCTGTGTAAGTCAGTGGATACTCTGGCAAGGCGTCTGATTTTGGTGCATCATTTGGTACCATATTTGGCTTAGCCGTACCAACTTTGACAACTTGCGTTACCGGAGCTAGAGTTTCCTCGTTAGACAATTCTTGGCGATCGATTTCTTGACCATTAGAGCTGTAAACGCGAGTTTTGATAGTTCGTTCACCCTCAACACCTGGAGTCGTAATTTGACGGGCATCA

>c180_g1

ACTGAAAGTTGGCTTGCCGCTTTGTGGTTGACCTTGCAGGCCTGTTGACTCAGCATTGGTGCTGGTTGGTGTGACACGCGTCACGGTTGGTGTGTAAGTAGCCGTTACTTCTGTTCCATTTGTGTCTACGCGTTTAACTGTTACTGGAGATGGGGTCCCCACATATTGCTTGTCTGGCGTGAAAGTAATCGTACCATCTGACTCAATGACATAAGTCCCGACCTTGTCTACCTTCTTGGTTGTTTGATTATCCTCAAAGGTCATTGGTTGGGCCATATCAATCGGCACAGCCTTGTCGCCTGGGGTGAAGATCGGCTTGCCACTTTGTTGTTGTCCTTGCAAGCCGCTTGAAACGGCATTGGTACTTCTTGGCGTCACTTCCGTCACGGTTGGCGTATAGGTTGCTCTTATTTCGGTGCCATTTTCGTCAACTCGTTTGACCGTCACCGAGGCTGGGGTTCCAACATATTTCTTATCTGGAGTAAAGGTAATAGATCCATCTGAATTGATGGTGTATTCCCCTACGCCCGCAACCGTCTTCTTAGGTTGACCATCGTCAAAGGTCATTTGCTTGGTCATATCGATTGGGACCGCTGGATCGCCCTCCTTGAAGACTGGCGTTCCTGTTTGTGGAACACCTTGAGGACCTGATGAAGTAGCAGGTGTGCTGGTTGGTGTGACCTTGGTCACGTTCGGTGTATAGGTTGCAGTCACTTCTGTGCCGTTGGCATCGACACGTTTGACTGTAACCGGTGTTGCCTTGCCAGTGAATTGCTTAAGTGGCTTGAAGGTAATGGTATCATCTGAATTGATAGTATAAGTTCCTTCATTAGCTACTTCCAAGGTGTTCGTCCCATTTTCAAAAGTCATGGCTTTGCTCATGTCAATTGGGATTTGTTGGTCACCTGGGGTGAAGGTCGGCTTACCACTTTGTGCTTGACCTTGGAGGCCAGTTGATTCAACATTGGTGCTTCTTGGTGTTACTTGGGTTACCTTAGGTTGGTAGCGTGCTCGGTGTGCTTTACCATCGGCATCACTGACTTGAACTACCACTGGGTCTGGCGTACCATAAAAAGTTTTGATAGGTTTGAAGGTCACTTGGCCGGTATTTGGATCTAATTCATAGGTCCCGACTTGCTGGCCATTGGACATGGCTGGCACCGTGTTGCCTGCTACTGTCTGACCGTTAGCATCCACAAAGCTGGCAGGATGGGTAGCGTCTGGCGTAACAGGAGGCGTATTCGCATCATCATCGTTAAAGACTAGCGTTTTGTTTTGTTCCAGTCCCTGAAGACCACTTGATTCGTAGTTTGGAATCTTACGGACTGTCGGCACATAACGGCCATCCATTGTAGTCAATTGATCATTTTTATTAGGGTCAGAAGCATCCGTTGAAGTCCATCCAGTTGATGTACCATTGCTGTCAGTCCAACGGATGTTAATTCCATCAGCAATTCCAGAAAATTCCGCATTTGGTGTGAAGACTACTTGAACATCATTGCCATTGACGGTCAAGCGATAGGTCCCTTCTGGACGAATATAGTTGCCATCAACAGGCGTCAGAGCATTGCCTTGTTTATCCAGAACCTGAGGCGCTTGGGTACTCATAACTGCATTGACACTGTAATCTGAACGATCCGTTCCTTTAGGTGTGAAGTGAAGGACAGCAGTTTGAGTCTCACCTGTAAAGCCGTCCGTAGTCTGCTTTTCACCTTTAGGAGGATAAATGCGGTGTAAGAGCATATCTTCTACTTCACCACTAAAGGCAATACCGGTTGGTTGCTCAATATCACCTTTATTAGTCGCAATTCGGAAACGCATACCCAATTTGTCTACTTGACCGCCACTAATATTTGGGTTGGCATTGAAGGTCAAGGTATAGTCACCAGCAGCTGTAATCTCTTGTAGGTTGCTACCTTCGCTTTCATTGAAGACTCCATCATTGTTGAAGTCTATCCATGCCTTGACATAGGATTTAGCATTGCCATTTGGATTAGCTTTGATTTTAATTGTGTAAGTTCCATTTTTTGCTTTGTCTAAATCAAGCAAATCATTAGAATTACTTAATTGATCGTCCGCTAGTAATTGGCTGGCCCCCTCGTCCTTGTGCTCCTTTTTATCATCCAGAACCCAGTCATTGGATGACTCAACATCAATGTCTGCTGGAGTAGTCCCTAAGTAAGGCTGATTAATTAGCTTACCAGTCAGAGAATCACGACCTGAAATGGTATGGAAGGCTTCACCATAACTTTCCGGCGCATCGCTCCCATCTACCACGAGAAAACCCATCATCATGGCCTGTTGCCCAGATGATGCTACGTAAAATCCTACTTCAGAAGCTCCGCGTGTCATCACCACAGGAACCGTCCGTTCGTTCGAACGGTTTGGTCCAAAGACCTGAGTCCCCAAGCCACCTGTCACTGTATCCGGACTGAGGTACTTATACCAGTCTACGCTTGTATCTCTTAAGATTAAGAGACCTCTTCTCTTTACATTTTCATCATCCAGCATTTGCTTGGTAATAACGGTGTAGGCTTCTTTGGCAGTACTCTTATTCTTCCATTCACCGAGATATTCCCATCCTTCTCCGTTAGTAGTGAAGATAGCAAATTCACCTGGGTTGGCATCTTCACCATCGGCCATAACCACCGTAGGGGCCACGCGCTTACCGAGATAGGTTGCCTCAATATTGAATTTCACTCCCCAGTTGGTAGAGTTCTCTGGATAGACAAGCTGCGTCTTTCGTCCTTGCGTATTAAAACCTTGATCACGGATAGAAGTCCATTGGTTCTGGATTGCCCCCGTCACTGTAGGAGGTGTTTTCCCATATTGTGGAGCAGACTTTAAATATCCATTCTGAGCATTTGGGTCATAAGTATTTGCTGTCGGTGTTCCTTCAACCCGTTTTCTATAAGTTTCAGTCGAGTTAAAAGGTTTAAGCTCAGTGACCGTCAGGGTTACTTCATAGCCCGATGAAATTTCCTTCTTGAAGGTCGTTCCTACTTGGAGTCCGCCTCGTGAATCCAATCCTTTCCAACTTGCAGTATCCGAAAAATCAAGCCAACTAATCTGCTTAGCTAACTGAACACTGTCTTTCTTAACTGATTCCTCCAAGCTTGGTTTAGGTACTCGTGGCGTAGAGGTAGCATCATCTGCCCCAGCTGGTCCAGTACCTATCGGAACTCCTGTTGTTACGTTATTCTGTTGTCCAGTAGGCGCAGCATCCCGTCTCACACGACGAGACCGATTAGGCTGAGCTAGATCTGCGGTTGCTTCTGACTTTTCAGCCACGACAGTCGCGTTGGATATTTCTGGGCGAACAGTGATTGGTTGAGTATTCTCAACTGGTTTAGTTGTTGTAGATTCAGTAGATTCTTCAACTTTCTCAGACTCTGAGCTTCTTGGACTAGGATTATCATCTGATTGAGAAGGCTCACTAGCAAAAGCGAGTGCTGAACTTTCTTTTGCTTGTAAATGTGCAGAAGCCACAGTTTCAGCAGAATTCACTGATGATTCGGCTATTCCTGTTTGCGAAACTTCATTTTGGGCACCAGAATCACTAGCTTCGTCAGCACTAACTTGTGCCGCTGCTCCCAAAAGAAGCAGGGTTGACAAGAGCACAGAACAAACACCGACGTTTAGTTTCCGAATCGAAAACTTACGCAGGTGAGGATTAAACAAATCTTTTCCCATAATAATCTCCTAAATAATCATTTAAATTGCAGCTTTTATTATACACTATTCTATTTTAGTTATCCATATATATAACTATAATTGTAAGAAACTAACATTATAATTAGACACTTTGTTGAAAACAGTCTATTTTTAACAAAAATAGGAAAGATCCATAAACGAATCTTTCCTATTTTGATTTAGCGGTTAGAAG

>c180_g2

ACTGAAAGTTGGCTTGCCACTTTGCGGTTGACCTTGGATACCAGTTGATTCAGCATTGGTACTAGTAGGCGTTACCCGTGTTACCGTTGGCGTATAGGTGGCAGTAACTTCCGTGCCGTTCTTATCTACACGTTTAACAGTAACTGAAGCTGGGGTCCCCACATACTGTTTGTCTGGGGTAAAGGTGATAGTACCATCTGAGTTGATGGTATATTCTCCTAATCCTTGAACTGTCTTCTTAGGTTGACCATCTTCAAAGGTCATTGGAGAATCCATATCAATCGGAACAAGTGGATCACCACCTTGGAAGCTTGGACTACCTGTTTGCGGAACCCCCTGAGGACCTGTGCTAGTCGCGTCAGTTCCTCTTGGAACCACTTCTTTCACAACT

>c180_g3

TCGCGGTGCCACCTCAATTATGGAAAATAGCTAATATTCCCCATATCTCTGTCTTGTCATCAAACAAGCTGCACTGTAAGGTGTGCCCACCGAATTCTTATTGTTTCAAATTCATTTTATCCATTAGCCCAATTTCGTAAAGATTTGCTGCTCATTTCCACCAACCACAAGCTCGCTAAAAACAAATGCATCTACTACTTTTCTAATTTCCTATATTTTAAGTTTTTCCTGAAAAGATGTCAAGACCTTTTTGAAAATTTTTATAAAATGTTCGGATTTGATTTATAGAGGCTGTTATAGGCAAAAATTAAATTGCTTTTTGGCGAGACTTTAGAAGCTTTTAGCTTGGTTTTTTGTTATAATTTTCTGTAGCAACTTTCAAGGAGAAAAACATGTCCTTCGACGGATTTTTTTTACACCACATGACAGAGGAGCTCCGCCACGAATTGCTGGGCGGCCGCATTCAGAAGATTAACCAGCCCTTTGAACAGGAGCTGGTTTTACAAATTCGGAGCAACCGCCAAAGCCACAAGCTGCTCCTATCAGCTCATTCGGTTTTTGGGCGTGTCCAGCTGACAGATACCACGTTTGAAAATCCAGCTGTTCCCAATACCTTTATCATGGTCATGCGCAAATACCTGCAGGGCGCTGTTATTGAAGCAATCCAGCAGGTAGAGAATGACCGGATTTTGGAAATCAGTGTTTCGAATAAGAATGAAATCGGTGACAGCGTGGCTGTGACTCTGGTCATCGAAATTATGGGCAAGCACAGCAATATCATTCTCTTGGATAAGGCTAGCGGTAAGATTATTGAAGCCATCAAACATGTAGGATTTTCTCAGAATAGCTATCGAACGATCCTGCCTGGCTCAACCTATGTCGCACCGCCTCAGACAGGCAGTCTCAATCCTTTCGCTGTGGGTGATGAAAAGCTCTTTGAAATCTTACATACTGAAGACTTAGAGCCCAAACGCCTGCAGCAGATTTTTCAGGGATTGGGCCGAGATACGGCTACTGAGCTCAGTGGCCGTCTGACAGCTGACAAGCTCAAAACTTTCCGTGCATTTTTTGCCAGTCCAACTCAGCCAAGCCTGACCGAAAAATCCTTCTCTGCTCTGCTATTTTCCGACAGCAAGACCCAAATGTCCACGCTATCCGAGCTTTTGGACACTTTCTATAAGGACAAGGCTGAGCGAGATCGAGTCAACCAGCAGGCTAGCGAACTGATTCGTCGAGTAGAAAATGAGTTGGAAAAGAACCGAAAAAAGCTGGTCAAGCAAGAGGAAGAACTTCTAGCAACGGAGAATGCAGAGGAATTCCGTCAAAAAGGGGAGCTCTTAACTACCTTTCTCCATCAGGTACCTAACGATCAGGACCAGGTTGAGTTGGATAATTACTATACAGGTGAGAAGATTATTATCTCGCTTGACAAGGCCCTAACCCCCAACCAAAATGCCCAGCGCTATTTCAAACGTTACCAGAAGCTCAAGGAAGCCGTCAAACATCTGACCAGCTTGATTGAGGAGACTCGGGCTACGATTCTCTACCTAGAGAGCGTGGAAACTGCCCTTGCTCAGGCCAGCCTGACTGAAATTGCAGAAATTCGGGAGGAACTAATCCAGACTGGCTTTATCCGCCGACGCCAAAGAGAGAAAATCCAGAAAAGACAGAAACCGGAGAAATATTTGGCAACAGATGGCCAAACTATTATCCTAGTTGGACGCAATAATCTACAAAATGACGAGCTGACGTTTAAGATGGCCAAGAAGGACGAGCTTTGGTTTCACGCCAAGGATATTCCAGGTAGTCATGTGGTGATTACTGGCAACCTCCAACCTAGCGATGAAGTCAAGACAGACGCTGCCGAGTTAGCAGCCTATTTTTCCAAAGCCAGACTCTCCAATCTGGTCCAAGTAGATATGATTGAGACTAGAAAGCTCAACAAACCAACCGGTGGAAAGCCAGGATTTGTCACCTATACGGGCCAGAAAACCTTGCGCGTCACACCAGATGAAGAGAAAATTAAAAGCATGAAGATATAAAGTTACCCTCTTTAACTATCAAACTGACTTAGGAGCAGGACAGTCATTAGCTAGCTGAAGCTTTCTATTATCTCCATCACTTTCAAGAATTTTTTGATAACAAAACAGGTAGAACATAAGTTCTACCTGTTTTTGCGCCCTCCTGAAATTCTTCTAGTTCATTGGCGAAATTTCTTCAAGCCTATTTTAGTCTTCAGACTTTTTCTTTCTCTTCTCTAAGCCAATCAGTCCTAAACCTGATACTCCTGCAAGTAAGCTTGCTGCCAAGTATGAGGTTTCTTCTGTACCAGTCTTAGGAAGGGATTTTGCACCCTCACTCTTGGAAAGAGACGGTGGTGCAGGCGTAATCGTCCGCTCTGGCTGAACAGGATTTTCTGTCACAGTCGGAATGTAAACCGCTGAAATCGTCTGACCATTGCGGTCTTTGCGAATCACTGTCACACCGTCAGCTTGGCCGACAAAGCCAGTTTCCGGTACAAAGGTCACGGTGCCATCTGGCGCAATCGTGTAAGTACCTTGACCTGGAACAACCTTTTCAGTACTGCCATCCTCGAAAGTTGGAGGAACGGTTGGATCCACGTCGCCTTCAAAGATTGGTTTACCGGTCTGAGGCTGTCCTTTAAGACCCGTGCTGCTGGTATCTTCTGCGGCAGTACTTCCCAAGACAGTTGGGGTATAGGTAGCCGTCACAGCATTGCCATAAATATCTGAACGCTTCACGACAACTCCGCGAGCAGTTCCGACAAAGTCAGCTTCTGGTACAAAGGTCACTGCTCCTAGCATATTGAACCTGTAAGTTCCTTCACCTGGGACAACCATTTCGGTACTGCCGTCCACAAAGGTCGGTGCTACTGCCTGGTCAATCGCCCCTTCAAAGGTTGGCGTGCCCGTTTGAGCTTGGCCCTTGCGGCCACTGGATACACTGTCCTTGCTAGTTGATGGTGCAACTACGGTAGGAACATAACGAGCTGTAACTGTGGTGCCATTCTTATCGCGACGGACAAGGGTCACACCGCTTCCCTGACCAAGGAAATCATAATCTGGAGTGAAAGTGACTGTGCCATCTGGTGCGATAGTATAAGTTCCCTGACCCGGAATAGTTTTTTCCTTGCTTCCGTCTTCGAAACTTGGTGCAATCGTCTCATCAATTGGAACCAATGGATCACCTGCTTCGAAGATAGGTTTGCCAGTTTGAACATGGCCCTTGATATTTGTAGATGTAGCATCTTTACCAGTTGGAATTACCTTGGCGAACTCTGGACTGTATGTCGCAGTTACTGGAGTGCCATTTTTATCTACTCGTTTCACAGTGATTGGATCAGGTTTGCCTACAAACTGCTTATCTGGAGTGAAAGTCACTGCGCCATCTGGAGCAATCGTGTATGTTCCTTGACCTGGAATGGTCTTCTCTTTGCTTCCGTCGTTGAAGCTTGGCTCTACTGTGTCATCGATTGGAACTAGTTGGTCACCACCTGTTAAGGTTGGAGTACCCGTTTGAGGAACACCCTGAGGACCAGTGCTGGTTGCGTTGGTGCCTGTAGGAATAACTTCTTTCACTGCCGCTTGATATTTGACAGTTACTGGAGTTCCATTCGCATCAGTCCGAGTCAATTCGAGTTCCGGTGTTTCCCCCTTGAACTGTTTGTCTGGTGTGAAAGTCACCTTTCCGTCCGCATTCACTTCGAACTTGCCGACATTTGGAACTTCTTTCACTGTTGAATTGTTATCAAACAGCGGAGTTGAACCAGTTGGGAATGGAACAGAATCATGGCCTGGAGTGAAGGTCACTTTA

>c180_g4

CGTGAAGGTGACTTGGCCTTCTTGAACTTGGCCTTGCAAGCCTTCTGTCTTAGTGCCAGTACCAGTTGGAGTTACTTTCGTAAATTCTGGACTGTAGGTAGCTGTTACTGGAGTGCCATTTTTATCGACACGTTTTACAGTCACTGAATCTGGTTTGCCTACAAACTGCTTGTCTGGCGTGAAAGTCACTGCACCATCTGGAGCAATCGTGTAGGTTCCTTGACCTGGAATCTTTTTCTCCTTACTTCCATCTTCGAAAGTTGGCTCTACTGATTCATCAATTGGAACTAGTGTATCGCCACCTGTGAAGGTTGGGGTGCCAGTTTGAGGGACACCTTGAGGACCTGTGCTAGTCGCATTGGTGCTGGTTGGTATCACCTTGGTAACGGTCGGTGTATAAGTCGCAGTTATTTCTGTACCGTTAGCATCGACTCGTTTAACCGTAACAGGTGTCGCCTTGCCAGTAAATTGCTTAACTGGTTGGAAGGTAATAGAACCATCTGAATTAATGGTATAAGTTCCTTGATTAGCCACTACCAAAGTGTCAGTCCCATTTTCAAAGGTCATTGGCTTGGTCATATCGATTGGAACAGCTTGGTCACCTGG

>c180_g5

ACAAACTGCTTGTCTGGCGTGAAAGTCACTGCACCATCTGGAGCAATCGTGTAGGTTCCTTGACCTGGAATCTTTTTCTCCTTACTTCCATCTTCGAAAGTTGGTTCAACTGTTTCATCAATTGGAACCAATGGATCACCTGCTTCGAAGATAGGTTTGCCAGTTTGAACATGGCCCTTGATATTTGTAGATGTAGCATCTTTACCAGTTGGAATTACCTTGGCGAACTCTGGACTGTATGTCGCAGTTACTGGAGTGCCATTTTTATCTACTCGTTTCACAGTGATTGGATCAGGTTTGCCTACAAACTGCTTATCTGGAGTGAAAGTCACTGCGCCATCTGGAGCAATCGTGTATGTTCCTTGACCTGGAATGGTCTTCTTCTTGCTTCCATCTTCAAAGGTCGGCTCAACGGTTTCATCAATTGGAACTAGTGGATCACCACCCGCAAAGATCGGGGTGCCGGTTTGAGGAACACCTTGAGGGCCTGTGCTAATCGCATTGGTGCTGGTTGGGGTTACCTTGGTAAACTCTGGACTGTATGTCGCAGTCACTGGTGTGCCGTTCTTGTCTACTCGTTTAACAGTGATTGGATCTGGTTTTCCTACAAACTGCTTTTCTGGTGTGAAGGTCACTGCTCCATCTGGAGTAATAGTGTAAGTTCCCTGACCTGGAATTTTCTTCTCTTTACTTCCATCTGCAAAGCTTGGCTCTACTGTATCATCGATTGGAACTAGTGGATCACCACCTGCAAAGGTCGGTGTGCCGGTTTGCGGAACACCTTGAGGACCAGTGCTAGTCACATTGGTGCTAGTAGGAATCACTTCTTTCACTACCGCTTGGTACTTAACTGTAACAGAGGTACCATTGACATCAGTGCGAGTCAATTCAAGTTCTGGCGTTTCACCCTTGAATTGTTTATCTGGAGTGAAAGTAACCTTACCGTCTGCGTCCACTTCGAACTTACCAACATTTGGTACTTCTTTAACTGCGGTACCATTATCGAATAAT

>c182_g1

CAAACATGAAAAAAGAAACATTCTCAGACAAGATGATTAAACGATTTTACGGCATTACTGGACCTTTGGACGAGCAAAAGCGCCAACAGGCTGAGCATCTAGGAAATATAGGCTTTATTTGGCTTTTCTTTATTCTGATTTTTGGCAATGCCATCGCCTTTCCCCTAGCCTTTCGCTGGCCCAAAATTATCGCTGTTGCCTATCCTATCCTGCTGGAAATCCTTATCCTCGGCTTCTGTGTCTATATAGTCATTCAAGCACGGCGCAAAGGAATTGATGACCTAGAGCTAGGACTTCAGGATGAAAAAGAAGAAAGGGCCATGAAACATGCTGGCCTCAAAGCAGGTTTCTCCTATTGGCTACTCTTTTACCCTCTCTTTAGCATCATGATAGCTGTCATGGAAAAGAAAGATATCCTACAAGTTCTGCTTCAACCTAAACTCATTATAACTGGATTAGCTGCTGGTCTTGGATTTGGACTGATTATGCATTTCATTGCCAAAGGAAAGATTCGTCAAGCACAAAAAAAGGAGGAAGAAGACCTATGAGATTCAATCTCAAAGAAAAAATGGATTTAGGGATTCTGTCCCTCATTATTGCCATCGCTGCTTATTTCTTTTTCCGTATCACGGAGCCTTGGATTTTCTGGCTGGATGTAGCAATCATCGCTAGCTGCATCTATGTAGCTCTCCGTATTTTGAAAAAACGTAAACAAAAATAAAAGAACCCCTCGACAGGAA

>c182_g10

CTGACTTGAAAACCAATTAGACTTGTTTAGGTTTTTTCGGTTTTACTACTCTTGGCTTTCTGCCATCAAGCTTTCTCTTGGCTGCTCTGAGATTGGCAATGGCTTCTTTGACATCTTCCTGGCTGGCACCTGGATTATCCAAAACTTCCTTAGCCTGTGCATAAGCTTCTAGATAAGCCGCCTTAACCGCGTCAGTCGCATAGATAAACTTATTGCTGATGGCTTGATAATGCACCTCATCATCGACTAGCTTCTTGAGATTGGTAAAGTTAGTCGCCTTACCATCCAGCTTCTTCTCTGCTGTAGTCAGCTGAGCTAGGGCTGTATCTATTTGCTCCTGGGTCACCTTGTCCTGCACCAAGAGCAGAGCAACTGCTTGGAATGCCTTGTCATAAGCACGGCGAGTCCTATCCTTAGCATTGGCATAAGCCCCTGTCTTAGTAGTAGCTGCATAAGCCTCTACTGCCGCTTTAACCGCAGCAATATCAGAGTCCTTGCCGTCTAGATCAGCAGATGCTGTTTCTAGTTTTGCTCTTACGGCATCGACTTGCGTTTGGGTCAGTTTGCTTGTCAAAGCTTCCTTGGCTGCTTGATATGCTTGGTCGTAGGCTGTGCGCTTCGCTGCAGAAGCATTGTAATACTGAGCAGTGCCGACTAGACTAGACTGCTGATTAACAGCTGCCAGCAAGCCTGACTTGACGATAACTCCCAGCTTCAGCAGATTATTCTTACCTTCTTTGACCTCAACAGTCGGCTCATTTTCCCAGAGCTCATAGCCAGTCGGCAGGTTAACAAAGACTGAGTAGAATCCAGCTGCTACGGATCTTCCGAAAGCATGCTTGCCAAACAGTTCTGCTGGAAGCGTATAGGCATCGCCGTTATTATTCTTGAGAACCACTTGGGTATTCTTCGGAACAGCTTGATCAAAGGCAACGCTGACCGGTGCAAAGACCAGCTTCTTGACTAGAAACTCAATCGTCTTAAAGCTATCTTGGGCTGTCAGCTCAAACTCCTGAGTCAGATCGCTGTAGAAACGAAGCCCATCCTTATCATAAGTAAAGATCTCTGCTGTATAGCGGCCAAATGGCAGAGCGTTGATTTGCTTGCCCTTGTCCAGTTCGACATACTTGCCCTTGCTGTCCTTGATTCGATAGACAAAGGTTGAAGATACATCCTGATGCGTATTAGCATCAACCAGAGCAACACGGATGCGCCCTTCATTTTCAGCACTGACTAGGTCTGACAGGGCTATGGCATCACGGTTACCCGCATAGTCCTCTACCACATAGAAAATCTTAGACTTGTCCACTCCTTTTGGCAAGGTGTAGCTGCCATCCGCATTGGCTGAGATGTAAACTCGACGCTCGTAATCACGCTCAATGCCAAAATCATCCACGGCCTTATAGGTCTCTTTCCCTTTTTCATCTGCTTGCAGATAGAAGACCTGTTCTCTCAAAATACCGCCGTTGCCGACATCTTTTGGCTTACGAGCCGTAAAGGTTTCCGCACCATCCTTGGTACTTATATAGCCTGAACTAATCAAAGGTTTCTGCGTATCGATCTGCAGCTTAAAGCTAATCTGCTGCTCTTTAGCACCTGGTACATCTGGCGTATAGCGAACCACATAGGTATAGAGGCCATCTTCCAACTTCTCACCCTTGCTATCACGACCATCCCAGTATGTATCTTCTAGTAGGTAGGACTTAGGATTGTCTGACTGACCACTATAATAGTTCTTGCGGCCTTCTCTAATCGTGCTGCTCTGCCAGATTGGGAAGCGATAGTCCGTATCATCTGCCGTATAGACACTAGCTGTCAGATTATTGATATTGCGATAGAGAACTGTCCGATACTGGATGCTGTCTTGATTGCCATCACCATTAGGTGAGAAGGCCAAACGAATCTTGCCATCCGCTCCCAGCTGCAGCACATGCTTACCATCCGCATTTTCTGCAGTTCCTAGTACAATTGAGCTGCGAGCAGCTGTTCGACCGGTAGAGTACAGTGTATCATTTGACTCTGTCACAAGGGAAGAGACATTGTCATCAGACAGGATACTCTTGTCCTCTGGCACTGGGTAATAGAAGCCTGACTTACCTTCTTGGACCAGATTATAGATTGGTCTTTCTACAGCTGGAAGATCTTGGAATTGCCCACGGAAGCCCATAAATGGCAGACTAACCACATCGCCATCATCCGCTGGATCGACAAACCGGACAAAGCCTTCTAAGAAATAGCCGTTTGGCATCTGCTTGATCAACTCTTCCGCAAACTTAGCAGTATTTACCTTGACCGTAACTTTCTGGCTGCTGTGAGCCTTGACCGTAACTTCTGGCCAGACTGTTTCGGTTAGTTTGCGCGGTGTCAGGGTAAAGTAGCCATCCTTAACCGCATCCGTATTGGTATTGACAATCATCTTCAGCGTCCGGTCCTTATCAGTGATATTATGAACCGTAACATCAAAGGTGAAGCTGTCTTGGACATTACCCAAGGAAACGCTAGGATATTGGTTATCCCCTGTCACATAGAGGCCGGTAGAAATAGCCGCTGCCGTATCCACGATACCAGCACCCTGCTGACGCGGAGAGGTGTAGACACCAGTTTCTTTGTTGACGTGCAGCTTAGCTGTTGACATGATGAGGGCTTTGACCAAATCGGCATTCTGGGCAGGTGTTAAATCTGGATAGTGCTGAAGGAGATATTCCTTGACCAGAGCTGCTACACCAGCTACATGAGGCGTAGCCATACTGGTTCCCTTCATAGAGCCATAGGTATTGTCATTAAGCGAAGAGTAGATATCGCCACCTGGCGCTGTCACATCTGGCTTGAGCAAACCATCTGTCGTTACACCCCAACTAGAGAAGTCAGACAGACTACCTGCTCCTGGATGCGGCCGATTAACCTTAAGACCATTAAAGACCACCTTGTAGTTACCAGCTGCCAAAGCCTCTCCATATTCCTTACTGATAAAGGCTGACGGAACTTTTTTAGATTCACTGTCCAGGCTCATCGTTAGGTTAGCTCCGTCGACATTATTGTAGATCAGAGCACCAACAGCACCATGGCTGATGGCATTTCTAACCTTTTCAGCAAAGGTAAAGGAACCACGCTGGATAAGAGCCAGCTTCCCTGTCAGATCCTTACCAGCAAAGTCCTCTTCACGGCCAAGACCAACATGGACATACTCATATTCCTTGCCCTTTTCAAAGGTTGCATTTGTCTCACCCATGGAGTAGCTGAAATGACCGTTCAGGAGCTTGTCATTCTTCTCCAAGCCACGAACTTCCATGACTTCCTCTGTCAGAACAGTGTTATTGACAGAAGCGACTGAGATAGAGCTTTCTGCTGTTGAAGGACTGCCAACTAGACCATAGTCTGGATTTTCAACCAGCGGTTTAGAGTATTCACTTCCGAAGGTGTTATCATTACCCGCCGCAATGATCACACTCACTCCCTTAGCTCGAGCGCGCTCAATAGCCGCCTGCAGACTAGGGCTGACATCAACCGTTGAACCTGTTGCTGATCCTAGACTCATATTGATGGTATCAGCTCCCAAAGCCACCGCATCATCAATAGCTTTGATATAGATAGCGTCGCTAGTTGTGCGTTGACGGTCTGAGAATACCCGCATAAACATAACTTGTGCTTCTGGCGCAACACCATAGACATACTCATCATTAGGAGCTTTCTTGTTGGGATTTCCCGCTGTAATCCCTGTGACGTGCATACCATGTGAGTAACTGTTCTTTTCCTTGACATTGTCATCACCGTCGATATAGTTATAGGCATAGACAACCTTGTTGTTGTACCATTTCCCGTAGTCAATACCGGCCTTTTTCTTAGCCTCTTCCAAAGCCGCCTCTGTCTGATACTTGGCCTTTGACGGATCAGAAATCTTCAGAACCTCGTGATGGACATCCAGACCTGAGTCGATAATGGCTACCACACGGCCTTGACCTTTATAGCCTTGTGACCAGGTCTGAGGGACCTTGATAATCTCATTGGTACTGATGCTGCTTGGCTTAGCATCCTGAGAAGTTGGTGACTCTGCTACTGCATCAGGTTTAGATGCTGGCTTGTCTGAGTTTGCGGCTGCCGGAGACTCTACAGTCGGACTAGCCGGAGCTGTTGGCTTGGTCTCTGTGGCAGATGGCTGAGTTACTTTCTCAGCACTTGAAGAAGCCTCTTCTGATGAAGAAGTCGCTCCTTTTTCTACAGGAGCTGCAGATGCTTCCTTAGCCTCTGCCGTAGAAGGCTGGCTAGCCTGCTCTGTACTTGAAGCAGCCGATTCAGAAGAAGCTGGTGCTGCTTTCTCCACAGTAGCTGGAGTTGCCGGCTGACTCTCAGGTCTAGCTACTTGAGAAGCAGGCTCAGTCGCTGATACCACAGTGGCAGTCGGCTGGCCTTGCCCTGATGAACTAGCTGCCGCCTGCTCATCTGCCTGAGCCTGTCCAGCACCAAAAACCAGAGCAGTTCCCAGCAGAACAGAAGCCAAACCGAACTTGTATTTA

>c182_g11

GCTAGACTCTAACTGACTTTCTGTAAATATCCGTCCTAAACAATCTTGTAACTCTAACATACTTATTTATTCGTAATTTTTTGGCAAAATTCATCTTTTTTTTATAAAATATAGTTATGGAATTTAAGACAATTAAAGAAGATGGCATGGTTCAAGAGGAAATTAAAAAATCCCGCTTTATCTGCCATGTAAAACGAGTTTACTCCGAGGAAGAGGCTCGCGCTTTTATCGCTGCTATAAAAAAAGAACATTACAAGGCCACTCATAACTGCTCTGCTTTTATCATTGGGGAAAAGAGTGATATAAAGCGAACTAGCGATGATGGTGAGCCCAGCGGAACAGCCGGAGTTCCCATGCTGGGAGTCCTAGAAAAGCATGAATTGACCAATCTTTGCGTTGTGGTTACTCGCTATTTTGGCGGTATTAAACTAGGAGCCGGCGGTCTGATTCGAGCTTATGCCGGCAGTGTTGCTCTGGCTATTAAGGAGATTGGACTGGTTGAAATAAAAGAACAGGCTGGCCTGCACCTAAGACTGTCTTACAGCCAATATCAGGATTTTACGAATTTTCTTAAGGCGGAAAATCTTGCTGAATACGATACTGAATTTACAGATACTGTCTCCACTCTTCTTTTCGTTGACAAAGGTGAAAGAGAACTGCTTAAAGACAAACTGACTGAGTTTTTTAAGGGCAAATTGGAGATGATGGATCAAGGACTGCGCCAAGTAGAAGTCCCCTTGATGTTTTGACATAAAGAAAAGCTATGGCTCTCATAGCTTTTTTATATTTTACAAAGCTCTTATTTCATTTTATACTAATTCTA

>c182_g14

TCTCAAACTCATCCAAAAACTTCTCCAGTTCTTCCTGAGCTTCAGACGTTGTGCCTCTGAGCAGGCCCAGCATGTTTACAAGTTCAGCTGTTTTGCTGGCAATCTCACTATCGGTACAATTGATATTGGCGACAATCTCTGTTAGCGAAGTACATACGCCGAGCTCCAGAGAGCCTGATCTCCCCTAGGACGTTTGAGTTGTTTCTTGTGTCATAAATTTCTTTCTAGTTATTGGGTTTGAGAAACAGCAGAGCGGTTATCAATAGCCGATAACTTCTTTTTCAACTGCTGCTATGATTTCTTGGGGAATGTCTGAGATAGCCAAACGATAAACGATATTCTACCTGTGTACAGAAATTAATTAAGTCCCAATAACGGATTCCAAACCCACATAATCAACTTGATATAGTAAAACAAATTTAGGGTTGAATTCAGCTATTTCATTTCATTAGGATTGTTTCCAGCTTTTGTTAACTTTTTTAACCTAATCACTGGTATGCTAGATTTTTGAATTTATTCAATTTCATCATTCTCTTCTTCAAACAATGATATTATTCCTTGATTCTGTGAAATCACTTCAGTCTTATTTTTAGATTTTTCTTTGTATACTGCCTTTAGAAAATTTTCCTTACTAGATAATGCAAAATCCAATAAACCGCTTTTATACAAAAATATATAAATTGAGCACGCTTCTGCTTGACAATTTTTTGACTTTTCAGGATTAAACTCTATATCAGTAAATGCATCGTATTTTATTATTTGGTTTGCTAGAGATTTATGCTGATTAAGAGAATTTATATACAACCAATTATAAAAAAATGTTTGTGGTTCAAGCGGAAAATGTTGACCAAAACTTTCAAATTCAACTAGTTTATCAACCTGATGTAGCTTTCCTACAATTTTTTTCAACTCCTTAGATGTCATAGAATCTAGTAGTTTTAGTTGAGAACCATTTTTCTTAAACACTTTGCTGCCCTGAAAAATTTGTTCTACTGAATATACTTTACCTGATTTAAGTGTCACTTTTAAATTAAATGCACTAGCAGCTCTACCAACTTGATCTATTGACTTACTGGAAACCTCTAATATTTTTTTATTAGGAAACTGTCTTTTTATGTTTTTATGTAATGATTGAATTGATTTTTGTTTTTGAGAGGCTGAAAAACCTGAATAAAACTTAAAATCAATCATATACTTTTTATACCCAAAAGCGTCTCCATAAAATACTGGTCTATTTGCCATTCTTACTCCCTGTAATCATTAAAATTTATTGGAAAGTCATATGGAACTCTTCCGCAATTTAACCATTTAAGTGTTTTCTCACTGTCAAACAAATCTGTTGATTGTCTTATCTCAATATTTGCTTGATTCACTAATTTGAATATATCATCATCAACAAAATTTTCGTGAAAAATAACGCCTTTAATCTGGTTTATTGGAATAATCCCTTTAATTAATACTTCCGCTTGTAAATTTGATGGAACACTAGAAATCCATTTTGGATTTCTAGTCCGTCCTTCATTCAAAAATATAGACTCATAGGCTTCAACTCCTTCACTCACATATTTACCACATTCTTTGGCTGCATTGAGAGGACAAAATAGGCTTGTATCATCAATTATATAAGGATTAATTTTAAAGATAATCCATTCTTCTATATTTGTTTGAGTCTTCCGTACCATATTCTTCAAACAATATGAATTAGGTAATTCTACAGATGTACAAACATAATCTATGTAGCCATCAACTCTATCTGAATCATTCCATTGATCACGTGGTATACTCCTTGATGAAAAGATACCATTTTTTAGTATATTGCTCAAATTTGAAACATGTGTGAAATGACAAAGCCTATGGATTTGTCTAGATTTAGTAATTAAATCATATGGATTGTTATAAGGCATTTTTTATCCTCCATTTTATAAATTATTTTAGTATAAAATCACTTACCTAGCTTATATTTGACTTAATTTTGCAATCTCATAATTTGATAAGCTATTACTTAAAAGTTCTAAGTTTTCTTTCACATAGTTGATTGTTTCGACATCTCTCGGAATTACTATTTTCTTTAATGCAGAAGATGGTACACCAAAATCTAATAAGACACTCAAATTTTCATCAACTCCTTCATGTTCTAGTTTTGCAATATAAATTGAATAATCTGCTGTCTCATTTCTCAAAATATAATTTACAATACTTTGTAGAATAGCAAGCATTTTTGGTATTTCATACTTTGCTTGATTCTTAATGAAATAAAAAACATTTGATACTGCTTCATTAAGAAGTTCTTCCTTTTCCTTATAACAATTTTCCTTCTTAGAATTAAGGTATTCATACTCTAATTGTATTGCATTTGAAAGATTATTGTTGACAATCAGCTGACATTTAGTCGCAGTATATTTATATCCTTCTCCTTCTATTCCTTTTAATCCTTTATTTATAATTCTAAGCGTTTCTGATAAAATATTATAATCAGGCATAGGTGCGCGCCAGCTTAGTATTTCAAGCAACTCGCTATTTGAAATTAAGTAGTTGAATAACGATTTCTGCTTTTCCACATTATAGTAATTATTTTTAAATATCTCTATTAAATCTTCAGGAATTCCCTCTACCAGTTTTTGAAATTCTTCTTGTTTACTCTGTTTTAAATCGTTCTCATCAATATTTAATAAAATTTCACTTTGCAAATTATCATCTTGATCAACCACAGGGATATCTATAAATTCATCCTTTCTTTCAGGAGGATTATCAAAATAAAAAATATTCCCAGTATAATACTTCATCATTCGGCCTGCTCGACCTTTTATATTATTAAAATCAAAGTAAGTTATTTTACGAGTTCCTTTGAACATATCATAAATAACTACATTTTTTGCGATTGAATTTACACCTTCAATTAAAGAAGTAGTTGCAAAAATAATTTTTAAATCACCACTATTAAAGTAATCCAACTGACTATTGACGATATGGCGAGGGTACTGACCATTGTGTAGGCCAATCCCATTCTTTAAATATTTTTTAAGAATCCAATTTGAAGAAATATTTTCATCAATCCAATCAAAAACTGGAAAAGATTTCTCTTTCTCTAAAGAAAATTGTTCTATGTACTTTTTAGCAAGATCTTCGGACCTTTTTGGAGAACGAACATAAACTATCGTAGGTTCCGAAAAATCATTTAGCAATTGAAATAATTTTTCTTCCTTTTCGTCTTCTGATTCATAAGAGATCTGTTTTATTTTTTGATTTACTAAACTATATTCTGTTCTGTAAAATTGAATATCATATTTTCTTATAAAATCTTCTGAGATATTTTCAACTATAGGAGTAAGAAATAGTAATTGAGGTTTGAATTTCATGATTTTATAAAAAGCAATATTTAAATGACTCACTCTATCATCTTTTTTACTATTAGCTATTTTATAAAATTCATCAATAATAAACAAATCAATATCATTTATATTTGGGAAAATATCTAAAACTCGTTCACTTGTCAGTATAAACAAATTTTTTTCTCCAATCTTTTGATGAGTATTAACTACAATATTATAATAGTCAGAATAATGATTAAGTTTTCGTCTTGTTTCATCGATCAGTGCAAGCGTTGGTTGAATTATCAATATATTTTCATACTTTCTCCTTGCTACAAATTCCTCAATTAATAAGCTTTTACCAAAACTCGTTGGAGCAGAAACTACTAAATTTTGTTTTTTATTTATCTTTTCTTCTAAAAGCTTTTGTTCATAATGAAAAAGAACACTATTTCCTTGTTTGTTAACAAATTGGGATTTATGATATTCTCTACGAATTAATGCAGCAAGATCTAAATCTCCGCCCTCCTTTAAATAAGGGTAAAATCCAAAACCTTCAATTAAACTTGAAAAGACTGGCTGCACTTCAGAATGAACATACTCCCAATTTTCAATTACATCAATGAGTTTCTTTCTATCATTACTTACTGCAATCTCTTTTGCTAAATTAAAACAAAAGTCAAAATCTCTCAATTCCTCAATTATCATATAATGCTTGCTCTCTTTTTAACTTATTGTGTAGTGCCTGGATTAATTTAAATTTGCTATTTACTGGAAATAAAAATAAATGAATTTTTAACTTATCTTTATAAGGGTAAGTTGAAATTTTTTTATCAAAATCAAATTTAGTTTTTTCTACTGCTTTTTTCAATACATCTAAGAACTCTTGAGACGGATTATCTGTACCAGACTCATACGAAAAACTATTCACAATTTTACTATCAAATAATGCAAAGAAACATGCATTAATATTTATAATTTTAGATAATAATTTTGTTTCTGGATCAACTAAACTACTTATATCATCATCTTGAACACTCAAATCGCCAAATCTTCTCTTAATTATTGTAAACTCTTGATTAAAAAAATCAATATTGAAATGTTTGTTCAAATCGTCAGCTAAATCTCTTAAAGCACTATTTTTATCTTTATAAAATTTACTCTCTCCAATCCACAGCTCTTTTGAAATTTTATCAAAATGGACGGCGTCAAAGCCATGAACAACAGAATTATAACTATCTTTAAAATAAAGTTTAGATATTAATTGAGGCTTCCCCAATTTTTCTTGTAATAAGTAATATAAAATAAGTTCTCCAAATTCTCCTCTTTTTAAGTATTTATCATCAAACTTTAATTTAGGGTTGGTGAGATATTCTTTATTTGCCTCTCTTATTTCTTTGATATCATAGAGTAATTTTAATCCTTGTCTGACTGCCTCTCTTGGACTTTCTTCACAAATTTCCATAGCTTTATCCCGTCCTAAAGCAAAAAATGGAATTGAATTAAAAATTTCTTCAATCCAACCTTTTTCATTTTGAACAATATTATTAGACCCATCTAAATCAAAAACAATATGAAAACAATGAGATGATATTTCATCGTATTGATAGTAATCAGTATATTTTCCTTTCATATCTAAACTACCTCTTTCCTTCCCTACTTCCTCAATCAGTCG

>c182_g15

TTTGACTGCTTTTTTAGTTGCTTTTGCAAATACAAAACGCAATGCATAGTCTCCCTGAAACTTGTATTGACTGAAATAATTCTTCATTGCTTCATTATAGGTAAATAGTGACTGATGCTGAACTTGATTCCCCTCTCCCTCCCATTTTTGGCAATCTGTACATAAATCTGATTGCCTGTTGCGAAAACAGCGTGGGCAACGCTCTTCAGATATGCATTCAAAATTTTGATAACAAGTGGAGCAGCAGCTTGGTTCTTCTCCTTTTAATAGAAAAAGCTGTAAAAAACTGCCTTTCTCCTCAATTTGCCCCTTGCATAAAAGACACTCTGTCATAATCCTGCCTCCTTATTCATATCCCTCATTTCCCGAATTGCCTTTTCCATAGCAAGGGTAGTCCCATCATGAAAAAATAGCAACTGTCCAGTCGGTCTATTCATACTACG

>c182_g16

TTCACATTCCCTACCCAATGTCTATTAAACACCAAAAATCCCCTAGAATTTACAATTCTAAGGGATTATTTTTTATCCTGTTACTTCCGGTACTGGTACTGATTCAGTTTTAGTTTTCTTATTTGGGCTAGCTAGGCGGCGCTTGCTGTCTCGGATAGTATTGAGATCCTTGAGGAGTTTGGTAAAGTGGGCTTTCTCTGGGTAGGCTTCTGCAGAGGCTGCGGTATAGCGCATAAAGAGGTCATAAGTGCGGGAGATTTCCGTGCGCAGCTGCTTGGTCTTACCGACTTCCTTGCTGGACTTGGCTGCACGCGCTAGGGCTTCGGCCTTATCATAGTCTTCCTGAGCAGTCGTCACTGCGCTAATCACTGGTGAGAGCCCCAGCGTAGTTACTGCCGCATTGTAGGGCTCTTCCTTGAGAGTTTTGAGGAGACTCTTAATCTCAGCAGTGGCGACATCGTTATTATGCTTGGTGATATCCTTGTACTTAGTCAGGACTGGAGCCAGCGTCTCATGCGCTTCCTTGAACTTTGCTTCCTTTATCTTAGCAAATCCCCTATGCAGGGTAAAGAGCCCAACCAGAGCGCTGTCCCGCTCCTTATCTACCTCTGCCAGACTCGTGGTCTGCTTCTTTTCCACACTGGCTAGCTGTTCTTGAAACTCTTCTAGCTTGCTCGCAAATGGCTCCAAGTGCGTGCCATACATATCTTCGTTCTTATTTGCCTTGACAAAGTCCGTGATGATTTGACGAGAATCTGTCATCAAACTTTCAAACTCACGGTGAGTAAAGTTGCTATAGGTCAAGGGACGAATGG

>c182_g17

TTAAGACTCTGGGTCATCTAGACTACGGCCGTGAAGGCCTTTCTCGCGCTGAACTTGACGCAATTTTTCAGGCGTTACATCATTGCCATCCTCATCAACAATCTTGATGCCTTCGATGTGATGGCGAACAGAGCGACGGTAGCCTTCGATATACTCTTCACGAAGCTTGGCTTGTTCCACTTTTTCCTCAGCAGTTAAACCTTCCGTTTTTTTCTTTTTAGCCAGTTCATTAATACGGGCAATTTTTTTAGGATCCATTATTCTACCCCTCTCTTACCTTTTATTTGGTATTAATCTGATTAAACTGGGCCAGCTGTCCCGCTTTAGCCGTCAAAGACGCCAGGAGAGCTAGGCGATTGTTACGCACAGCCTCATCTTCAGCCATAACCATGGTATGGTCAAAGAAAGCATCAATAACTGGACTGAGTGCAAAGAGTTGGTCCATATTGGCAGCTAAGTCAGATGTCAGCTCCACTTTTTCAATAGCTGCAGCCAAGTCTTTTTCCTCTTGATTTTCAAAGAGTGCTGGATTTATAGCTGTCTGCCCTTGGGCCTTCTCAGCTAGATTAAAGACCCGAGACAGGCTTTCCACTGCTGATTTGAAATTGTCTTCCTGAGCTTTTTCTGCAAGAAGCGCAGCTGTTTCTACCAAATCGCGTACGACGAAATTAGTGCTTTGAAGAACAGCCGTCACGATGTCTTTTGGAGTGCTGCGATCCATCATCTTCTCAACGCGGGCACGGAAGAAGTCCAGCACAGCCTCTTGATTGTCATAGCTTAGACTATCAAACTTGAGGCCGTAAAGTCGACCAAGAAGCTGAGCCAAGTCAATGTTCCAGCCAAACTTGTCCAAGATACGCACGACACCCTGAGTCGCACGGCGTAGTGCATAAGGGTCATTGGAACCGCTCGGAATCAATCCAACTGAGAAGAAGGACAGAATGGTATCCAGCTTATCAGCCAGAGCCAAGACAGCACCGACCTTGGTGTCAGGCAATTCGCCATCAGCGGATGTCGGCATATAGTGCTCCCGAATGGCAGCTGCCACTGCAGCATTCTCACCAGCCAGAAGAGCATACTTCTCACCCATGATACCTTGCAGCTCATCAAACTCCCCAACCATGCCAGTCAGCAGGTCAAACTTATAAATAGCTGCTGCACGAGCCAAGTCGGCTGTCTCATTAGCATCCAATCCTGCCTCTTTTGCCAAGAGAGCAGCAATCTTGCCAGTCCGCTCCATGTGCTCTGCAAGAGAGCCAATCTTTTCGTGGAAGGTCACATTGCTCAGCTTTTCAACCAAGTCGGCAATAGCCAGTTTTTGGTCTTCCCGCCAGAAGAATTCCCCATCTTCCAGACGAGCCACCAAGACTTTCTCATTTCCCTTGATGACATTTTCTAAGTGCTCTGCATTTCCGTTGCGGACAGAGATAAAGTGTGGCAAAAGCTTGCCTTCAGCATCTCGCACAACAAAATAACGCTGGTGCTCTTTCATAGAAGTGACCAAGACTTCCTCAGGCACCTCTAAGTATTTAGCATCAAAATTACCCAAGAAAGCAGTTGGATATTCTACCAGATTCAACACTTCATTAAGCAGGTTTTCGTCAATCTCGATAGAAACTCCGTGCTCCTCTTCCAGTGCTCTAATCTGCTCAACAATCATATCTCCACGTTCAAGAGGACTAGCAATGACAAACTGAGCCCGTAGATCATCTTCATAGGAATCCGCTGAAGAAATCTCCGTTTCTTTCCCCAAGAAACGGTGTCCTCGGCTCGTACGGCCGCTCTTGATATCCAAGAAATCCAAATCAAAGGCCTGCTCATCTAGGAGCACAGTCAAGGTGTGGACTGGGCGGATGTATTCAAAGGTGTTGTTGGCCCAGTGCATGCTGACAGGGAAGGTCAGAGCTTGCAGAACTTCTGTCACTGCTGGGATGATTTCCTCTACCGGGCGGCCGATTTCTTCCTTGGTCACATAGACGTATTCTTCGCCCTTGATTTCACGGAAAGTAATGTCCTCAACCGTCAAGCCCTTGCCACGGACAAAGCCTTCGGCTGCCTTAGTAAAGTTGCCATCTGCGTCCAGAGCAATTTTCTTAGAAGGGCCTTTGAAATCTTCGGTCAGATCAGACTGCTTGTCCGCCAAGCCAACTACGCGCACCGCCAAACGACGAGGTGTGGAGAACATTTCAATTTTCTCAAAGGTCAGACGATGGTCTGTCAGGAAGGCTCCCATCTTGTCGCGCAGCTGCTTCATGCTCGGTGTCACGACATAGGCTGGCATTTCCTCCAAGCCTAATTCAACTAATAAATTTTTTACCATGATTATTCTCCCTCCTCTGCCAAGAGTTTCTCGCGAGTCTCTGCATCCAAAAGCGGATAACCCAGACGCTTGCGCTCAGCCACAAAGGTCTTGGCCACGACACGGGCCAGATTACGAATACGGGCGATATAACCAGCCCGCTCTGTCACAGATACAGCTCCACGTGCATCCAAGAGGTTAAAGGTATGCGAGCATTTGAGAACATAGTCATAGGCTGGGTGCACCAGATGCTCGTCCAGACAGCGCTTAGCTTCTGCTTCAAACCTCTCAAAATTCCCCAAGAGCAAGTCTTGGTCGCTGACCTCAAAACTGTACTTGGAATGCTCATACTCAGGATGAGTGAAAATTTCGCCGTACTTAACACCATCAGCCCACTCAATGTCGTAAACTGAGTCCACTTCTTGGATATAAGAAGCCAGACGCTCCAGACCATAAGTCACCTCAGCAGTCACCGGGTGAGTTGGCAGACCTCCGACCTGCTGGAAGTAAGTGAACTGCGTGATTTCCATTCCATCCAGCCAAACTTCCCAGCCCAGACCAGCAGAGCCAGTTGACGGATTTTCCCAGTTATCCTCAACAAAGCGGATGTCGTGCTCCAGCGGATTAATCCCCAAGAGCTCCAAAGACTGCAAGTAAAGTTCTTGGATATTGCTTGGAGATGGCTTCATAACCACCTGAAATTGATGGTGCTGATAAAGACGGTTGGGATTTTCCCCATAACGACCATCTGCTGGCCGACGAGACGGCTCTACATAAGCCGCATTCCACGGCTCTGGTCCAATAGCCCGCAGGAAGGTATAGGGACTCATCGTTCCTGCCCCCTTCTCATTATCATAAGCCTGCATGAGCATACAGCCCTGGTCATTCCAGAACTGCTGTAAAGTCAAAATTATTTCCTGAAAGGTAAGTTTCTTGGACATTAGTTACTCCTTTTGTTTGTTTATATCTCTTTGTCTATTATTTTCTGTTCTATGAACCAATCCAACAAGGGAAGTGTTTTATCTGTGTTTGCCCACCGATGATAAGAATCAAAGACCGTTTGCACGCTGCCTAGATTTTCTACTAGCTTGTCAACTGTCAAAAAACTGCGCCAGTATTCGTAAAAAATACTAGCATAATTCCCTTGATAAGTCGAAGTGTCGAAATCATTCAATGAGTGCCAACCGTGCTTTTTCTGAAAGAGCTTTACGAGAGCCTGATTACAAGCTTTTTCCACTCGAAATTCCTCATCTGTAAAGAAATACTTGCGACTAATATACTCAGCCATCCCTTCTTCAAACCAGATATAGGCACCGTATCCATCCAAGTCATCTAAAAAATGCTCTGACCAATGAGCCAACTCATGACCTACAATCTGTAAGAGAGAATTTTCAGACAAAGACTGATAGTGGCTTTCTATTGCTCGAGTTTGGTGAGAGATCTCGTAATTCTCCAACTGAAGGAGATATAAATCTTTCCAAACCGTCAATTCGGGAGTCATAACCATCCGCCTATCATTTGTATAGGCTGGTACAGGAATTTCACGAATGATCTGCGTAGCAGAATCAAAATCGGACCAAATGATGGCTTGCGGCAAGTCATAAACCGCAAACTCGTCTTTTAAAAATGTTAGATAATCCTGCAACTTTACGGCATTCTTTTCGACGAAATCTCGAAAAGCTGCTAATTGGTTCTCGTCTTTTACAAGATAAAGGTTCTCCACGAATCTCCTTTCTTTCGAACAATAGACTCAGTACGCAAAAGAACCACATCCGACAGTCCTAGGCTATTCACCTAGGGGCGTCAAGACGCGGTTCCACCCTAATTTATTACTTCATTACTTTTGAAATTATAACCGAAAGCGCCATCTGCACTGCTTCCCTATCCGGCT

>c182_g18

ACAGAACCTCCTCTATCAATAACCCTTGCAAGCACTTGGTAAATCATTTCTGTTTTTCCAGCGCCAGTAACAGCATGCACTAACGTATTCTGCCTTTTCTCTAGCGCATCAAGTAATCCTTTGGAAACCTTTCCTTGAAACTCTGTCAGTTGCCCCTGCCATTTAAGCACCTGATTCTTAGGAAAATCCTCTTGGGGAAAATAGTAGAGTTTTTGGTCACTCCGTACCTGACCCAAGATTAAGCATTCTCTGCAATAATAAGCTCCAACCGGCAGTTGATTTTCTCGTTCAATCAAGCTGTTGCAGCGTCCGCAGCAGAGCTTGCCTTTTTCTTCTCGGATACTGGGAATTTCTTTTGCTTG

>c182_g2

GTCAAATATAACTGACAAACTTGTTGAAAGTTATCAGTTATAGTATTAGAAAGGAAGGGTGTAATGAATCGTGTCAAAGAATTTCGAAAAGAAAAAAAGATGTCTCAGCTAGAGTTGGCCAAGTCTATCGGGGTATCGCGGCAAACTATCAATATGATTGAAAATAACAAATACAACCCCACCCTCGAGCTCTGTATCAATCTGGCTAGAGCGCTGGATACTGACCTCAACGCCCTCTTTTGGGAGCCCCAGCTGACAGATGAAGATTCAAATGACTAAGAATTCTTCCCTGCAGCAAATTTCATACCTAAAGAAAGGACTCAAACATGAAAAAAGAAACATTCTCAGACAAGATGATTAAACGATTTTACGGCATTACTGGACCTTTGGACGAGCAAAAGCGCCAACAGGCTGAGCATCTAGGAAATATAGGCTTTATCTGGCTTTTCTTGATTTTGCAAGTTGGTAATTTCCTAGCTTTCATGCTAGCCGATATCTATCCAGGCTTAGATGCTCGAATTTACCCCATTATTATAGAGCTTCTTACCTTTATAATAGCTGGTGTCATTTACTTCAGGTCTGAGAAAAAGCACCTGGCAGATTTAGATTTGGAACTCATGAGTGAAAAAGAAAGGCGCAAGCTTCAATATCCTGGTCTTAAGATTGCTCTCTTCGTTGGATTGACTTTCCATCCTATCTTTAGCCTTATAGAAGCAGTCACTCTCAAGCAAGACTTTTTCACTCTCTTTTTTCAAGCTGATCGAATCCTGAAAACAGCTTTGGTTGCTAGCATTTTAGGTGTTTTTATCAGCTTTTACTTCAAATCACGCAAACACCATACTGAACAGAGCGAATAATCCCTTGACCTAAGCTAGAAAGGACACAAACATGAAAAAAGAAACATTCTCAGACAAGATGATTAAACGATTTTACGGCATTACTGGACCTTTGGACGAGCAAAAGCGCCAACAGGCTGAGCATCTAGGAAATATAGGCTTTAT

>c182_g20

ATGAAGTACCGAAAGATGCTCCGGTTGAGGACAAGCCTTCTATCGAAATCACTCGCTTTGTAGATGTAGAAGGAAGACCTTTAGCAGCTGAGGAATTTGGTTTACTTGATGCAAAGGATTTTGAAAAATATGGTTTTGTTTCTGTAGCGGATGCAAATGGCGTTCGCACTTATGTTTACAAGCCAAAAACGCACACTCAAACACCTTTGGGAACAGAAACTTTTGAGCAGAAGAGTCCTGTAAGGGTTGAGAACTCTGAGACTACTCCTGTTCTGTCTCGAGTGTCCAAACATCAATTACC

>c182_g4

TTAGTATATCACCTATACTACCCAACTGTCAATACCTTTTTCCAATTTTTTTTGTTTTTTTTAAAGTTTATTTTTCTGTTCTAAGTATAAAGTTAATTTCTTTGTGAGCAGGAAGCTGGTTTATGTCACCTGAGTGTATGACTTTCTATAGCTAAAAAGGTTGAGAAACTTGCTCTCAACCCTCTTTTCTTATTTATTTGACTTCTAACAGGCCAATATCTCCATCCTCACGACGGTATATGACATTCGTACTGCTGTCCTCAGCATCGGAATAGATAAAGAAATCATGTCCCAATAAATCCATCTGCAGAATCGCTTCTTCCAAGTCCATCGGCTTCAAATCTATGTGTTTTGAACGGACAACTTTTGACGGAACTTCTTCTGCTTCTTCAGCAAAGGAATCTGTGAAAAGCTGACTTGTAGCAACTTTATTGCGATTCTTTCTTTCAATCTTGGTCTTGTTTTTGCGGATTTGGCGCTCAATCTTATCTGTGACCAAATCAATTGATCCATACATGTCCTGAGAGATATCTTCTGCACGAAGAGTGATGGAGCCTAGCGGAATTGTTACTTCCACTTTCGCAGTCTTTTCACGGTAAACCTTGAGGTTCACACGCGCATCAAGTTCTTGCTCTGCTTGGAAATACTTCTCAATCTTCTCAAGTTTAGAAACTACGTAGTCACGGAGAGCATCTGTTACTTCTAGGTTTTCACCACGGATACTATATTTAAGCATATAAGTACCTTCTTTCTAAACATAAATGTTTTATTTATAATA

>c182_g5

CCTCTCCAATAATTTGATGAACTAAGTATAGCACGTGACAATAAATCTTTTGCATGGCTGCCCTGAAATGTCATTTTTTATCCCCGAAATGACATTATACCAAAAAAATAGAAGAAAAAAACACGAAATATTGTAAAATATTTCGCATTTTTTACATTTCTGATCTCAGCAGGATTCGAACCTGCGACCGTTCGCTTAGAAGGCGAATGCTCTATCCAGCTGAGCTATGAGACCAATACTATCTCATTCTATCAGAAAATAGGATTGCCGTCAAGATTACTTATGATAAGGACTCCCCTGCTGAATCATAAAAGCTCGATAGATTTGCTCTATTAAAACAAGGCGCATCAGTTGATGAGGTAAGGTTAATTTGCCAAAACTCATTAGAAGATTTGCCCGTTTTTTTACTGCAGGATATAAACCTAAACTTCCCCCAATAACAAAAGTGATATCAGAAAAGCCATGCACTGTAGTATCCATCATGAGCTGACTAAACTTTTCTGACGGAAATTGGTTTCCTTCAATTGCCAAAGCAATAACAAATTCACGCTCATTGATTTTAGCCAGAATTTTATTTCCTTCTTTTTCAAGAATCTGCTGATTTTCTAAATCGCTAGCTTTATCAGGTGTTTTTTCATTCGCTAGCTCAATCATTTCCACCTTGCAGAATCGATTGAGACGCTTCATGTATTCAG

>c182_g6

TTCTATGTCACGTATTTATCAAAATATTACAGAACTAATTGGGCAAACTCCCATCGTCAAATTGAACAACTTGGTTCCAGAAGGAGCTGCTGAGGTTTATGTGAAGTTAGAGGCTTTCAATCCTGGCTCTTCTGTCAAAGACCGGATTGCCCTCAGCATGATTGAGGCGGCTGAGCGCGATGGCCTTATTAAGCCTGGCGATACCATCGTTGAAGCAACCAGCGGAAATACCGGTATTGGCCTTTCATGGGTTGGAGCAGCTAAAGGCTACAAGGTCGTCATTGTTATGCCAGAAACCATGAGTGTGGAACGCCGCAAGATTATCCAAGCTTATGGTGCTGAGTTAGTCTTGACTCCAGGTAGCGAAGGAATGAAAGGCGCTATTGCAAAAGCACAAGAAATCGCACAAGAACGAAATGGCTGGCTGCCACTGCAATTTAATAATCCGGCCAATCCAGAGGTTCACGAACGAACTACAGGAGCAGAAATTATCGCTGCTTTCGGTGAAACCGGACTGGATGCCTTTGTAGGCGGTGTCGGAACTGGCGGAACTATTTCCGGTGTATCTCACGCTCTCAAAAAAGTTAATCCGGACATCCAAATCTACGCAGTCGAAGCAGACGAATCTGCTATCCTTTCTGGTGAGAAGCCAGGCCCTCACAAAATCCAAGGACTTTCAGCTGGATTCATTCCAGAGACCTTAGATACAGAAGCCTACAACGGTATTGTCCGCGTAACTTCTGATCAAGCACTGGAATTCGGACGCTATATTGGCGGTCAGGAAGGCTTCTTAGTGGGGATTTCATCTGCTGCTGCTATTTTCGCAGCTATTGAAGTAGCGAAAAAACTAGGAGCTGGCAAGAAAGTCCTTGCTCTGGCTCCTGATAACGGCGAACGTTACCTGTCTACCGCTCTCTACGAATTTGATGCTTAGTATTTTTTGAAAACATGTCCATTTCAACTTAGTACTCAACTATGAATTGAATAGCAATTCTATCCAAGCAAAATATCCTGTTCTCTTATCGAACAGGATATTTTTTAAACTTTTTCTTCTTTTAAGAATTTTTTTGCCTCTTTGATCCAGATAGGCAACTGTCTTCCTAAAGGCTCAAAACCAATTTTACAGCGGTCATTTGAAAAGCGGTGGCGTCTAGGGAGGCGGTGCTTTTCTTCTTCCAGGGTTCGCATGGACAGACTGGACTTCCCAGAAAATTCATCATAGTCCACAACCTGTACCAATACTTGCTGGCCGATCTTTACACTATCATGGATATTTTCAATATAACCCGGACGAATTTCTGAGATATGAATCAGACCAATCGTTCCATTTTCCAGTTCAACAAAAGCCCCATAAGGCTGAATCCCTGTGATTTTGCCTTTTAATTTATCACCGATTTTCATCAGTCTTCTACCTCAATTGTCTCAATCACAACATCTTCCACCGGCTTATCCATAGCCCCCGTCTCAACAGCTGCAATCTTGTCCAAAACTTGGTAGGACGCTTCATCAGCCAGCTGACCAAAGACAGTGTGGCGCCGATCCAAATGCGGTGTTCCTCCCTGACTGGCATAGACTTCAGCGATAGCTTCAGGCCAGCCGCCGCGGCTCAACTCCTTAGCCGAATAAGGTAGATTTTTATTTTGAACAATGAAAAATTGACTGCCATTAGTATTTGGCCCAGCATTTGCCATGGAAAGAGCCCCACGAATATTGTACAATTCTGGTGAAAACTCATCTTCAAATTTCTCGCCATAAATAGACTGACCACCCATACCAGTACCAGTAGGATCGCCACCCTGAATCATAAAGTCCTGAATAATGCGGTGGAAAATCACACCGTCATAATAACCATCTTTAGCAAGGGCAATAAAGTTGGCTACTGTTTTTGGTGCCTGTTCTGGAAACAGCTGAACTTTCATGTCACCATGATTAGTCTTGATAGTCGCTTTTGGACCTTCTGCTGCTGCAAGTTCTACTTGTGGAAAATGTAATTCTTTTTCTACCATACCTAACTCCTCTAAGGCGGCAAAAATACCGTCTTCTTCTACTGTTTTTGTAATATAATCTGCTCTTTTTCGCAGTTCTTCATGGGAAACACCCATGGCAATGCCAATTCCGGCATAGTCAAACAATTCCAGATCATTGAGCCCGTCGCCAAAAACCATGACATTTTCAGGTTTCAAGCCCAGATGCTCGACAACCTTGGAAACTCCGGCTGCCTTGGAACCTTCTGTCGGCACGACATCTGACGAATGAGGATGCCAGCGAACCAAACGCAGATGCTCCGCTAACGCCTCTGGCAACTGCAGGCCATCTCCTCTATCTTCAAAGGTCCAAAGCTGATAAATATCCTTGTCTAAATGAAAATCTGGATCAACAGGCAAATCTGGATAAACCACATCAATGGCCTCTGAAATCAGAGAATTTCGATTGGACAAGGCTGCTCCATGGCTGCCGACCAGACCATAATCAATTCCTTCCTCCTGAGCCCAAGCAATATAGGATGTCACTCTATCGGACGGGATAACCGTCTGAGAAATCACCTTCCCTTTGCTGTCTTCGACATAAGCTCCATTCAGCGTAACGAAAAAGTCTGGTTGCAAAGCTCTCAGCTCCGGCACGACACCAAACATTCCTCGACCAGACGCAATACCCGTCAAAATCCCTTTGTCTTTCAACTGCTTGAAAACTGTCTGGATAGAATCTGGGATAAAGCCCGTATCCTTGACTCGTAGGGTATCATCTATATCGAAAAAGACAATTTTTATCTTTTTTGCCTTATACTTTAATTTTACGTCCATGTCTTCTTTTGTCCAATCTTAATCTTTTCTATTATACCATTAAAAGTCTTCTTGTGGGACAAGATGGTGCTGAAAAGCATAAACAACTGCCTGCGTGCGGTCGCTCACCTCCAATTTGGACAGGATATTGGAGACATGAGTCTTGACCGTTTTGAGGGAAATGAAGAGCTCATCTGCAATCCGTTGATTTTCATAACCCTTGGCCAAAAGCCCTAAAATATCACGCTCACGAGCTGTCAAATCCTCATGGAGTTCGATATGGTTACGATGGTACTCCACTTTCTTGCTGACCTCTGTTTCAATGGCAAATTCTCCCTTGGCCACTTTTTTGACGGCACGAAGAATTTCTTCAGCGCTGGAAGTCTTGAGCATGTATCCATGAGCGCCTGCATCCAGAACAGGATAAATTTTTTCATTATCCAAATAAGAAGTCAAAATCAGAATCTTTGCTTCCGGCCACTCCTTTAAAATGGCCAGTGTCGCTTCAATCCCATTCATCTCAGGCATGACGATATCCATGATAATCACATCCGGACGACTTGCCAAAGCCTTCTCCACACCTTCTTTGCCATTAACTGCTTCGGATACCTCTGCTATATCCTCTTGCAGTTCCAGATAACTTTTCAATCCCAATCTGACCATCTGATGGTCATCCACTAACAATATCTTCATGATCTTCTCCTTCTAGTAAAGGTATCGTGATTTCAATAGAAAGACCTTTTCTGGGGGCTGTCAAAAGCTTAAAAGTGCCCGCCATATCCCGTACACGGTCTTCCATATTTTTCAAGCCGTAGCTGAGTTCATCCTGAGCCAACGGATCAAAACCAACGCCATTATCAGATACCTTGATTTTAAGCTCATTAGGTGCCTGATAGAGGTAAATATCCAACCGACTGGCCTGGGCATGCCGCAGTGTGTTGTTGATAATTTCCTGCATAATCCTGAAGACATGCTCCTCCATCTGTTTGGGTAAATGCCCAACCTGATGATTGAAGTGAACATCAATGTCGCTTTTATCAGTCAGCTCCTTGATAATCACATCCATGCCTTCTACCAGAGTCTTATTTTCCAACTCTGTAGGTCGTAGGTGGAGCAGAAGAATCCGCAAATCCTTCTGGGCTGTGTCAAGAATATCGGCAATCCCCTTGAGCTGTTTTTGCAGCTTTTCTCCATCAAGCCGTTCTACATTTCCAGCCACTCCTGAAAGAATCATATTGGCTGCAAATAATTCCTGACTGACAGTATCGTGCAAATCGCGCGCAATCCGCCGCCGTTCTTTTTCGATAATTTTCTCTTCCGTCTGCAGGGTTTGGTTTTCAGACTTTTGGAGATTTTCTGTCAGACGGTGCAGCCTTCTCTCAAGCTGATGCAGAGAGCTATCCAGTTCAGGCTGATCTGTCGGCTCCAGCCTCCGTCCATCCAGCAAACGCCGAAGATTTTTTTGCACATGGGCTACCGACAGATATTGTATCAAGCGGGCTGCTATCACAATCAGAATGGTCAGTGATAAACCGATAATCACAACAAAGAAAACAAAAGACTGCAACAGCTCCAAATCATCAAAGAGCTCCTGCCAAGTAAAATCAAAAATATTGAAAATATAGTGAAAGATGATAAATAAGACAAAGAAAGTATAGAGGAGAATGAGAAAATAATTTGATTTCTTCACTTTCTGACAACCTCCACATTTCCTAAGAAGCTGACGAGGACAATCTTCACTGTCTTATTGGCTCGTTTATAGTCTGGTGTCGTCAGCGAAATGGTTTCGTTGCGTAGCTTACGAGATGGATGATGCAGAAAGTTCAGCTCCCCGTAAAGAGTATTGATTTGCAGCTGAATTTCCACATCCAGCGGCACAATAATCTTGGTATCACCAAAACCCTTTCGAATGACAATGACATTATCATGGTTGACCAAAATCACATCTTCCAAATGAATGGTGTCTTTCCCAACAATGCGCAAGAGATTGATGTCGTGAAATTGACAGCTGTCCTTAGAAAAATGCTGCAGGTCCCCCAACCAGCGATTCTTCTCACTCTGAATTTCCACATCTTCTTCATAAACGAGATGGGTTTCTTGATTTTCCTTATATATATAAGGATAGGCCACAATCATCCCATAAACCAGCGCAAATAAAAGGGCAGCAATGACATAGGGATTGAGCATAATAATGAAAAACAGCATAATCATGGAAGCGACCAGCAGAAAATTCCCTTTTTGCTTGCCGAAGTAATAATAGAGCAACAGCAAAAAAAGGACCAATATAATGACAACTCGTGAAAAATCAGCTGCCAACATGGTCACCAATGCCATGGATAATAAGACTGTTTCAAC

>c182_g7

CCCCTAACACTTAGCACTCATCGTTTACGGCGTGGACTACCAGGGTATCTAATCCTGTTCGCTCCCCACGCTTTCGCTCCTCAGCGTCAGTTACAGACCAGAGAGCCGCTTTCGCCACCGGTGTTCCTCCATATATCTACGCATTTCACCGCTACACATGGAATTCCACTCTCCCCTTCTGCACTCAAGTTAAACAGTTTCCAAAGCATACTATGGTTAAGCCACAGCCTTTAACTTCAGACTTATCTAACCGCCTGCGCTCGCTTTACGCCCAATAAATCCGGACAACGCTCGGGACCTACGTATTACCGCGGCTGCTGGCACGTAGTTAGCCGTCCCTTTCTGGTAAGA

>c182_g8

TTTCATTCCTTTTTTGCAAATTTTTTCTTATCTTGCGATTGAAAATGTCAAAATTTCTTTCACTCCTGCTTCCAGCAAGATTTGCTTAGCCAATTGCAAGGTTTTTCCAGTCGTATAGATATCATCAACCAGCAAAATTTTTTCTGGCAAATCAACTCCTTTCCTAATTTCAAAAGCTTGCTGCGTCAGCAGACGCTCCTCACGTGTCTTGCTCGACTGTGCTAAAGTGTCTTTTTTCTCTAATATATTTCTATATGACAAGTTTCCAGCATCTAAAATCCCCTGCACTTGATTAAAACCTCTCGCTTGAAATTTTTCGATACTGACTGGAATCGGAACAATGGTATGCTCT

>c182_g9

CAAAAGTTTAGGTGCTATTTTCTTCTTCTGAAGATACTTTTGAAAATCAGATAACCAAACTTTCTGAGGGATGGTCAAGGGATTACCATGAAATCTTCTGGGAAGACTCAGGCGTTTCAGCTCACCTTTTTGAACTCTTTTATCCAGTTCATCCGTGGAAGTGGCTGTCAGAAAAATAGTTGTAGCATCCTCTTTAACTGAACGCTCTACAGCGTGATACAGTATGGGATTATCCACATAAGGAAAGGCGTCTACCTCGTCCACAATCAACAAATCAAAAGCCCGATAAAATTTTAAGAGCTGGTGGG

>c183_g1

GGGCTATCATACCTGTGCTCAGAAGCGGATTGAAATTGCCGAGCACATGCGCTATCTGCCCATGGGTTACTTTAATCAAAATAGCTTGGGCAAGATTACCAGTGTTACGACTAATACGCTGGAAGGGTTGTCTGATGTAGCTACGCGAGTGGTTATGATGACTGTGCAGGGCTTTCTGACGACTGGTCTCATCACTATTTTGGTCTTTCTCTATGACTGGCGGGTTGGTCTGGTTCTCTTGGTGGGTCTTGTCCTCTTTCTCCTGCCCAATACCCTCATGCGTTGGCAAGTTGGCAAGGTTTCTGATGACAAGTATCAGGCGGATATGGACCTAGTAGCCGTTGTTTTGGAGTACAGCCAAGGAATTGCTGAAGTAAAGAACTATAATCTGGTCAACCGTTCTGCCAAGAAGCTGTCCAAAGCTATTGAAGGCAAGAGTCGGCTAGATACCAAAATGACACTCGTGACCTCACCTTTAATTGCCCTCCAAGGCATCGTGACCAAGCTG

>c183_g10

TGTTTTCGTAAGTCAGCTAGTATTTCCTGAGCAGTAGCGAGGTTGAAAGTCTTCTTGACTGTTAAACAACTGGCTACAGCAGCGATTTCTTCGCCTTGGGCTCCAGCTTGCAATGCTAAAGATTTGGCTTGCAATTTCATATGACCGGCTTGGATGCCAGTGCTGACTAGGGCTTTGAGGGCGGCGAAGTTTTGTGCCAATCCGACTGAAACGATGAGGGAAGCCAATTCCTTGGCCTGAGGCTGCCCCAGCAAGTCAAAGCTGGCTGCCACTGCGGGATTGAGGCCAATGGAACCTCCTTTAGTTGCAATGGGCATGGGCAGGGTCATCTGACCATGAAGCTGGCGTTTGTCTGGGTCTGCCGTCCAAGTAGAGAGGCCGCGGTAGCTTCCTTCTCGGCTGGCATAGGCATGGGCACCGGCTTCTACAGCCCTCCAGTCATTTCCAGTAGCCAGCACCAAAGCATCAATACCATTAAAGATACCTTTATTGTGGGTAGCTGCCCGGTAAGGGTCAACCTGGGCTAATTTGCTGGCCAGCTGCATTTTATCCGCAAGAAGTTCCGCCTCGGCCGTATCCCGGCTGAGAAAGCGATAGCCGATGACACAGCGGGCTGTTACGAGACTATCTGTTGCATAGTTTGACAGGATAGCCATCAGACTCTTACCGCCAGTCAGATCTTTCAGAGGAAGGGTGATGGCCTCCAGCATGGTGTTGAGCATATTGGCTCCCATAGCTTCCTGCGTATCCACAGATAGATAGAAAATGAGGAAGTCTTCCTTTGGCTCCAGCCAGAGATCTCTAGCTCCGCCCCCACGGGCAACGATCGATGGATAGGCTTGGTTGGCCTGCTCCAGCAACTCTTTCTTTTTCATGAGAATATCTTTAATGGCTCGGTCTGCATTATCAACCTGATAGAGCGCGATTTGCCCAATCATCTGGCGTTTGTGAACCTCGGTCTCAAATCCGCCTGAACGTTTGATAATCTTAGCGGCAAAGCTGGCTGCTGCTACCACAGATGGCTCTTCTGTCACAAATGGAACTTGATAGCTTTTGCCATCCACCAGAAAGTCTGGCACCAGAGAGTAGGGCAGGGCTAGAGTGGCCAGCACATTTTCACTCATCTGGTTGGCTGTTTCCAGAGGCAAAGTCTGCTGACTGTCTAGCAGCTGCCAATGTTCATCTTGTAAAAGTCCCTTTTCTTTCAGCATCTGCAGCCGTTCAGCAGGATTTTTTTTAGAAAATCCAGTCCAGTTTACTTTCATGATGATTTCTCAACTTTGATGTACTTACGTTGATGCTCTGCAATTTCTGCCAGAGCAAAGCTTTGTTCCTCATAGCCAGAGAAGCTGGCATTGCCTTCCGCATCCAGCTCAGCTTCTTCAAAAAAGATTCGCTCGTAATTCGGGATGGAAAGAGCCTGACGCTGGTCTAATTCCTCTAGGCGTGTTTGAGATAGATGCTTTTCATAGCCCTCTACCAAATTGGCACTAAAGATTTCAGAGACAGCTCCGCTGCCATAGCCAAAGAGAGCAATCCGATCGCCAGCCTTGAGGTTGTCAGAGTTTTCTAAAAGAGACAGAAGACCTAGGAAGAGGGAGCCTGTATAGATATTTCCAACTTTTTGGCTGTAGAGAATAGATGCTTCAAAGTTCTTTCTAAGCTGGTCTTGCTGCTCTTGGGGCAGGCTTTTATCCATGATTTTATTGAGGCCTTTAAGGGCCAGTTTTGGATAAGGCAGGTGGAAGCAGTAGGCCGCAAAATCTTTGAGAGCCAGCTTGTGGCGCTTCTGGTACTCCGCCCAAGTTGTCTTTAAGCTATCCAGATATTGCTGGGTTGAATAGAGGCCGTTGACATAAGGCGTTGTCGAGTAATTTGGGCGCCAAAAATCCATGACATCGCGTGTCTGGGCTACATTATCATCATTGAAAGCGAGGATACGGGGATTGCTGCTAATCAGCATAGAAATGGCTCCCGCTCCCTGCGTTGGCTCGCCAGGTGTGTTAATGCCATACTTGGCAATGTCGCTAGCTAGCACCAAAACTTTGCTGTCTGGATGTTTTTCAATATGGAGCTTGGCATAGTCCAGTGCAGCAGTAGCTCCATAGCAGGCTTCCTTAATTTCAAAGCTGCGGGCAAAAGGCTGGATACCCAAAAGGCCATGGACAAAAACAGCTGCCGCCTTGCTTTGGTCAATCCCTGACTCAGTAGCCACGATGACCATATCAATCTTTTCTTTGTCCTCTGCTGTCAGGATGGGGTCTGCTGCCGCCGCTCCCAAGGTAACAATATCCTCAGTCAGAGGAGCAATGCTTAGTTCCTTGAGTAAGAGCCCCTTGCTAAGCTTGTCTGGGTCAGTCCCACGGGCTGCCGCTAAATCATTTAATTTTAAGACATAATTACTGGTCGCAAAACCGATTTTGTCGATTCCGATTGTCATTATAAGACCTCTTTAAATCCTTTTATTCTAGTATATTCCGAAAACTATTTTACCATACTTAGGAATAAAACT

>c183_g100

TGATAATGGAACCCATCTTGACTGACTCGTCGCTGAAATAGCGGTAAAGCCGGCCATCTCCGTCTCCCATGTAGCTGATAGGAGAAAAACGGTCACTGCGGAAAGCGTCGTTCTCATCAATCAAGTCCTCATCTACCACGCGCCGAGTGACACGGGCGATGACATTGGTCAAGGCACCGCATTCGACCATATCCAGCACCTGACACTCAATGGACACAGGGCATTGGTCTACCAGCGGAGCATCCACGGTCTCGCCGATTGTGTAGCTAAGACCGGTCAAGGCAAACTTGTCCTTGCGGCTGTTGAAGCCAGCAATTTCAGACTCTTTGGTCAAATTCTTTTCTGGGACATTGACTGTGAATTGCTGGTGCTTGGTAATCTCTGTAATGGCATTTCCCTTGGTACGCATGGCAATAACCATCATGCTGCCCAAGGAATAAACCGAGCTTGAGGTTGAAATATTATAGCCGTGCACATCGTCCTTATAGCCTAAAAAGAAGACTGGAAAGCCGAAGTAAAGTTTTCTCGTTTCAAAAGTACGTTTCATATATGTTCCTTTATTTTTAATTTTAAAATAGATCAGCTAGTTAAGATATTCGTCAGCTGAAATGTCCCTTGTCGCCTATCAACTTTTTTGTCACGGGGTGACGAGCCTGGATAGGAAGCTCAAAGTCCTTTATCTCGTCAACGATTTCCCCGTCCGCCATGACCAAAATCCGATGACAGAGAGCATAGCTCAGCTTGAAGTCATGGGAGATGAAAAGATAGGTCAGCTGGTATTTCTCCCACAAATCATATAGGAGGCGTAATAGCCTTCCCTGAACAATGGGATCCAGACCGCTCAGAGCCTCATCAAAGATAAGAATATCTGGCTTTAAGAGCAGGCCGCGAGCGATGCAAATCCTCTGGAGCTGGCCGCCACTGAGCTGACGCGCTGGCTGATTCAGATAGCTTTTATCCAGACCGACGTCTTCTAGAATGGCTTCTATCTCTTCTCTAGCCACATCCTTTGGCAGGGCCTCTGTCAAGACCTGCTCGACTGTAAAGTGCGGATTGACCGAATGGAGCGAGTCTTGAAAAACCAGCAGAATTCTCACGCCAGACTTTTTGACTGAGTAGGGCTGGCCATCAAGTAAGATCTGGCCTTGACTAGGCTTTTCTAGGCCAATTAGCAATTTAGCCAGTGTACTCTTACCACTGCCGCTCTCACCCATCAGACCGATTATTTCACCCTTCTGGACGGAGAAGTTACAGTCTTTGACAACCATTTTGCCATCGAATTTTTTAAAAACATGTCTACATTCCAGCATCTTGTCTCACCAACCGTTCGTATGGATTTCCTAAGATAAGGTCTTGGCTATAGGTCTGTGAAGGGCTTTCGAGGAGCTTGGCCACCGGCCCTTCCTCCATGATAGTCCCTTGATACATGACAAGCATCTTGCCTCCCAGCTCCCGCGCCAAATGATAATCATGGGTCACTGTGATGAGGGTCTTGCCTTTAGCCAACTGCTCCTTTAAGATGAGGATAATCTGCTCGCGATTGTGGATGTCCAGCGCCGAAGTAGGCTCATCCAAAACGATGGTCTCTGGCTCTAGGCAAAGCAAGATAGCCAGCATGACCCTCTGCAACATACCGCCGCTGAGCTCAAAAG

>c183_g101

TAAAATTACCAACCTTGTTGCATTTGAAAACACCTATATCTTAGAGAACGATCAAGGCCTCCTAGTCGTCGATCCTGGCAGTGACTGGAAAAAGATTGAGCGCAAGCTGGAGGAACTCGCCAAGCCTGTGATTGCTGTACTCTTGACCCATACCCATTATGACCACATTATGAGTTTAGAAAAGGTCCGGGAGCACTATGCTGCCCCACCTGTCTATGTGGCTGAGAGTGAAAGCAGCTGGCTCTACACACCGACAGATAACCTATCTGGTCTGGCACGCCATGCTGATCTGGATGATATTATCTGTCGACCAGCAGAGGAGTTTTTCTCCTACGAGACAGACTACAACCTTGGAGGCTTTCATTTCTATGTGCTTGCAACACCTGGTCACTCCATCGGAGGTGTCTCTCTAGTCTTTCCTAATGACTGCCTTGTTTTGACGGGCGATGCCCTCTTTCGGGAAAGCATCGGACGAACGGATCTTCCTACAGGAAACATGGAGCAATTGCTGACATCCATTCGTGAGAAACTTCTGGTATTGCCTAAAGACTATGCTGCTTATCCTGGTCATGGTCATGATACCACTATTTCACATGAAAAAATCTTTAATCCATTTTTAGCTCAGTAGCCATTTAAGCAATTAACTATAACAAAAACAGAGTTTGAAGAATTTCAAACTCTGTTTTTTTATGATTTGTTAAATCTTCAAGAAGCGTCTCATAGAGATGATAGACCCCAATGATCCGATAAGAATTCCCAACACAAAGAGTGCTGCTATCATAATCGGACTAAAGAGTTTTGGATCAATCATAGAAAGTCCTTGACCTACCAAGCTCTTATTCATAGTCTGGTAGGCTATACCATATGTCAAATACACTACAAGTGACGGAACTGTTGCTCCTAACAAGCCAATCCATGCTCCTTCAAACAGGAATGGTCCGCGGATATAGCTATTCTTAGCACCAACCAAGCGCATGATTTGAATCTCGCGACTGCGTGAAATAATGGTAATCCGAATAGTATTGGAAATGAGAAGGATAGCAATGAAAATCAGAAGCGCTGTCCCAATCAATCCCCATGTTTGGATGAAATTAGAAAACGCAAAGAGATTTTTGGTATTGGTACCCCCATTTTGAACCTCTGATACACCATCAATCTTACGAGCATCTTTTGTAACAGATTCTACATACTTAGGTGCCTTGGTCTCAACAATATAAGCATCGTAAAGAGGATTATTTTCTTCATCAAAAACATCCCACTCACTACCCATGGTCTCTGTTAGCTTATTATACTGCTCTTGCTTGCTAGAAAAAGTAACCTTCTCTACATTTTTCATAGAAGTCAGAGCATCATAAACCTTGTGGTAGTCCTCATTCTGAACTGTTTGGCCTTCTTTTACAATCGTTTCGCTATTATCGAAAACATCCTTACGCATGTATACCATGACGCGGACATTATTGGAAATATCATTTGCCAACTTGGCTGTATTTAAAATTACTGAAGCAAAGACAGCCACTAAGCTCAAGGTAATCATAACCGAGCTGACAGCCGCTACAGTCATCCAACCATTCCGCGTAAGACTCTTAAGCGACTCAATCAAATGACGAAAAAATCTTCTAATCATCGTATCCGTATTCTCCTTCCGCTTCATCACGTACCACACGGCCATTTTCAATAGCAATAACGCGGTGACGCAGAGTATTTACAATCTGGCTGTTGTGGGTTGCCATCAGGACAGTTGTCCCCTGAAGGTTAATCCGCTCCAGCAGGTTCATAATCTCCCAAGAATTATCTGGGTCCAAGTTTCCTGTCGGCTCATCCGCAATCAGGACTTTAGGGTTGTTAGCAATAGCACGCGCAATAGCAATCCGCTGCTGCTCACCACCGGAAAGCTCGTTAGGGAATGAGCGAACCTTATGCTTAAGGCCAACTAGATCCAGCACTTCCATAACCCGCTTTTTAATATTGCGGCGGCGTTCACCAATAACTTCCATGGCGTAGGCAATATTCTCATACACTGTCTTTTTAGGAAGAAGTTTATAGTCCTGAAAGACGACTCCAACCTGACGGCGCAGCATTGGAACATCACGCTTCTTAATCTTAGCTAAGTCAAAATCAGCTACTTTCAGACTTCCCTTATCCAGCTTTATCTCCCGGTAAAGCAAACGAATAAAGGTTGACTTCCCCGCTCCTGAAGGACCTACAATATAGGCAAACTCCCCAGGTTCTACGTTAATAGACACACTACGCAAGGCAGTGGTCCCGTTGCCATACTTCTTGACAACATCTTTCATTTCAATTATTGACATCTATTGAAATTTCCTTTCAATCTAATTCTAATCTCTTACATTTCCTAGTTCAAACGCCATTTCAAATAAGCGTCGATGAAACCATCCAAATCTCCGTCCATCACCTTGTCCACCTGAGCTACTTCGTAACTAGTTCGATGGTCTTTGACCATGGTATAAGGCGTAAAGACATAGGAGCGGATTTGGCTGCCCCAGGAAATTTCCTTCTTATCTCCCTTGAGTGAGTCGACTTCGGCTGCTTGTTTCTCTTGCTCTAGCTGATAAAGCTTGGCCTGTAGCATCTTCATCGCCCGATCGCGGTTTCCATACTGAGTCCGGTCAACCGTAGACTGTACCACAATTCCTGTAGGAATATGGGTCAGCCGCACACCAGTAGAAACTTTATTGACGTTCTGCCCGCCGGCTCCTCCCGAGCGGAAAGTGTCCATCTTAATATCGTCATCGCGAATTTCCACCTCAATGGTGTCATCCAACTCCGGCATGACCTCTACTGAGGTAAAGGAAGTATGACGACGTTTAGCTGAGTCAAATGGCGAAATCCGGACCAAACGGTGGACTCCCATTTCTGACTTGAGCAGACCATAAGCGTGCGGTCCTTCAAATGAAAGGGTCACCGACTTAATGCCTGCTTCATCTCCAGCTTGATAGTCCAAGACCTCAACTTTGAAGCCCTTGGCATTGCCAAAGCGTGTATACATACGCAGCAGCATATCTCCCCAGTCCTGGGCCTCTGTACCACCAGAGCCCGGGTGAATTTCAAGGATAGCATTGTTATTGTCATAAGGCTCAGATAGGAGCAGGGTCATTTCATAGCTGGTCATCATTTTCTCGAGTTCGGCCAGCTTTTCTTCCAACTCACCCTGAACCAACTCATCCTCGGCCAGAAAATCAAGCAAGATTTCCGATTCATCAAAAAGCTCAGTCATCTGATGGAAATTTTCATAGGTTTGCTTGAGTTCATTCAGCTCTTGAGATGTCTTCTGGGCTGCAATGTTATCATCCCAAAAGTCGGGTTCTGTCATCTTATTTTCTAAGATGGCAATTTCTTCTTCCAGACCTTCTAAGTCAAAGAGACCCCCTGAAAGAAGCTAATTTTTCACGATTTGCGTCAATCTTTTGACGAATTTCTGAAATGTCCATAAATA

>c183_g102

TTTCAGAAGTCTTGACCGTTCTTGGCTGATTCGTCACTACATTTTCAGCTTTGCTTTCTTTGGGCTACTATTTGTTATGCTAAAAGGTTTCAGCACACCTCTTCCTGTCTGGCTTCTTTATCTGAGTTTCACACTCTTTTATCCTTTTGCCATGTTTGTCTATGAAAGCATCGTTGATTTGATTATAGGGGACAACATTTTTCTCATTCCTGTTCTTATCATGATCATTTGGAAAATCATTCGCTTCTTTATCATCTGGCTTTTTTCCATACCTATCGGCTTGATCGGACTAATTTATCTCTACTTTTCTGTCAATCGTAAGACTGACTAAGGCTGCTTCC

>c183_g103

TTTTTCACGAACACCCCTTCTTTCCTATGCCTCTCATTATAACAAATTTTGTCCCAAAAATCTGAATGAACCTAAAAAATCAGCCAATAAAAATCAGCTGATTTCAGATAGTTTCAGTCAAAAAATTCTTGTAATTCCTGCATAATGGCTGCCTCTGGCAGAATATCCAAAGAAGCCAATTGTGACAGATGATGCTGAATCTGCTTTCCATAGCGTTTACTAGAAATGCGATTATCCAAAAGAATAACAGCTGATTTCTGATGCTCATTGCGCCGAGTCCGGCCAATAGCCTGCTTAAGCCGTAAGATGGCAAGAGGCAGTTGATAATCATAAAAGGCATTCTTACCTTCCGCTTTCAGGCGGCTGTTAATCTTTTGCACAAAGAAATCCTTGGGATTGTCAAATGGAATCCGAGTAATCAGCTGAATAATCTGGTCTTGTTCAGCAAAATCAGCCCCCTCCCAGAAGCTGCCAGAGCCCAAAAGGATACCTGCCTCTCCTCTATCAAAACGGCGTTTGATATTGCCGGCATCGCCATTTTTATATTGGGCCAAATGCGGCAGAGCCAGCAAGTCCGAAACAGCCAAAAGCAAATTTTTAGAGGTGAAGAGGACGACGATAGGCAGACCAAGTTTTGCCACTTCTTGCAGGCAAGCAACAATCTCTTGGGCAAAATCCTCAGTGGACAAATCCACTACTGCTGGAAAACTTTTATCTAAAAAGAGCTTCTGCAGCGGCTTCTTCTTGCTTTTCAGCTTGTAAAAGTGATAACTTTCAAAGCCTAGCAACTGCGCCAGATTGACCTTAGAGCTGATTTCCAGAGTAGACGAAACCAAGATAACCTTAGCATTTTCAGGCAGGAAGTCAGTGAAATGCAGCAATTCTGAACGGCCAGCATGCAGAGTCATCAAGCGATGGTCTGCCAAATCTTCATCAACCAACCAATAATACTGGTATTTGCCACTGAACAAGTGCTGCAGCTCAGGCAATGCACTAGTTTTTAACTCGGACACATCCTGAAGCAGTCGGGCAATCTTTTCCGGACTTAGCTCAGATGCTTTCTTGCGATGCTTCTCTGCGGCATCAGCCAGCTCAAACTGAATGCTTTGCAAGAGCCTCTGCTGCAGAAGTTCCCCTTCTTCCTGAAGGAGATGATTTATCTGCTGCAGCGTTTTAGTTAGATTTATACTGGCCTGAGAGAAACTTTCCAAGGCGAAAAACATCTTCTGAGCCTCATCAACAACCAAAATACGATTGTCCAGCAAAGACTGGTCATCCTCTAAACGCGTCAGTAGATATGCATGATTGGTAACCACGACTCGACTGGTAGCTGACTTGACCTGTCCCAACCGCCAAAAATCTTCCTCATAAAAAAGAGAATGTTTGCTTAGCTTGCCATCATGACGGATTTCATTAAAGTAGCTTGAGAAGCGATGAGCCTGTCCGATTTCATTCAAATCACCCGTTTTGGTTTCTGTCAGCCAAACCAAGAGTTGCAGTTTGCAGCGATTAACCAAACGGTTGTCATATTCCCTGTCCAGTGTCTGATAGAAATGGTCCAGCTTGAGATAATTCTCCGGACTCTTGAGACTGTGAAAGGAAATACCAAATACTTCCTCCAACAATCGCCCTTCCTTTCGCAACAGCTGGTCCTGCAGAATCTTAGTCGGAACCGTCACTAGTACTTTTTCTTGACCTCTAGACAGAATAGGTAGCAAATAGCCAAAGGTCTTACCTAAGCCTGTCTGAGCCTCTAAAAAACTAGCCTCCTGCTGGTCCAAAGCTTCCTCCATAAAGCGAGCAAACTTGAGCTGTTCTGGCCGCTCTTCTAAGCCCAGCAGGTATAGATTGGTCAGGAAATCCTGCGACAGCTTCTTTTCCGAAGTCAGCTTTTGGGGACGTCGCAGGAAAATACCATGGCAGGATTGCAGTTCCTTATCTTGCTGATCCGGCATTGTCTGATAAACTTCCTCGATAGCCAGACGCGACTCATAGATAATGCTATCTGCCAAAGTTAGCATTTTCTCAACCAAGGCCTTGGGCAGGCTCTTGATTTTGTCTTGTATTTTTAAAAATAGCTGAGCCGTTGCTTGAGCATCTGCCAGCGCAGTATGGGCGTTTTCTAAAGAAATATCCAGCAAATTACAGAGATTGCCCAAGGAGTATTTATCAAAAGTCGGGTAGAAGACCTGAGCCAACTCTACCGTGTCCACACGCGGTGTCAGCAAGTCAAATCCTTCCCAGAAAAGGGCCTCAGCTAGTAGATTGGCATCAAACTTGACATTATGAGCGACAAAAATCGCATCCTGAATCAGCTCATATACCTCCGCTGCTACCTGAGAAAACTCCGGAGCTCGGCCAAGCCGTTCATCATCCAAACCTGTCAGCTCTTTGATATGCTCATCCAGATCCTCATGAGGATTGACATCTGTTTCATAAGTCTTGGTAATGATTCCATTCTCAATCAGAACAATGCCAATCTGGATAATCTTTGCACTGCTGCCCGTTCCTGTAGCCTCCAAGTCCACAACGGCATACTTATAATCATTTTGTGTCATACTGCTATGATTATATCATAAAAGCCATTCTGAAACATCTGAAAATTTTC

>c183_g104

GGCCGACTGCCCCCTGATAATGAGCTCGGAAACCACTCATGACCTCGTCCACAATAAAGAGAGAGCCGTATTCCTGAGTGAGGGAGCGAATAGTATTGATAAAGTCCGCATCCGCTGGAATCAAGCCCATATTGCCAGCAACTGCTTCTAAAATGACACAGGCAATGTCGTCTCCGTACTTTTCGAAGCAAGTCTTGAGAGCATCTGTATCATTGTAAGGCAAAGCCAGAGTTTCCCCAGCTACCTGAGCAATGACTCCGGCAGAATCAGCAATCCCAAAGCTGGCCAAGCCTGAACCAGCCTGAACCAAAAAGGAATCGCTGTGGCCGTGATAGCAGCCGATAAATTTGACAATTTTAGAGCGCTTGGTCACTCCACGCGCTACCCGAATGGCGCTCATGGTCGCTTCCGTTCCCGAATTGACCATCCGCATTCTTTCCATGAAAGGCAGACGCTCCTGCACCAGCTGGCCGAGGGCAATCTCTCTGGGACTAGGAGCACCAAAGCTGGTCCCCTCCCAAATAGCCTCCTCAACTGCCGGCAACACATCCTTAGGAGCATGCCCCAAAATCATAGGCCCCCAAGACAGGACGTAGTCAATATAGCGGTTGCCATCGACATCATGCAGATAGGCTCCGCTGGCTTTTTCGATAAAAAGCGGATGGCCTCCAACGGCCTTAAAAGCCCGGACAGGACTGTTGACCCCGCCTGGAAAGAGCTTTTGAGCCTCTGTGAAATATTGGTTGGAATGTTCTCTCTTCAATTTCGTCCTCCTTATCTAGAAAACTGCTGATTTTCCACAGTCTCTATCCTTCTTTCAGATAACCGGCCGCATCCTTGGCAAAATAAGTGATAATCAAGTCTGCACCTGCCCGCTTCATACTGGTCAAGGTTTCCATGACAATGGCTTTTTCATTGATCCAGCCCTGCTGGGCTGCTGCCTTGACCATAGCGTATTCTCCACTGACATTATAGGTCACCAGAGGCAGCTGGGTTTCCTGGCGCAGCTCTCTGAGGATATCCAGAAAGGCCAGAGCCGGCTTAACCATGAGAAAGTCTGCTCCCTGCTCCTCATCGCTCTTGGCCTCTCTTAGAGCCTCCAAGCGATTGGCCGGATCCATCTGATAGGTCTTGCGGTCGCCAAAGGCCGGAGCACTCTCACCTGCATCTCGGAAAGGTCCATAAAAGCTAGAGGCAAACTTAATCGCATAGGACATGATGGGAATGTCTTCAAAGCCAGCCGCATCAAGCCCCTGACGAATAGCCGCCACAAAACCGTCCATGGCATTGGAAGGCGCAATGACATCTGCTCCAGCCCTGGCTTGGCTGACAGCGACTTCTGTCAGTCTAGTCAGAGACAGATCATTGTCCACTTCCTCCCCTCGCAAAATACCGCAATGGCCGTGACTGGTAAACTCACAGAGACAGGTGTCTGCAATGACGACCGTCTCAGGGAAATGCTTCTTAATCAGACGGATAGCTTCCTGTACAATGCCATTTTCAGCTGAAGCCTGGCTGCCTATTTCGTCCTTTTCTGACGGAATGCCAAAGACGATAAAAGCCCGAATGCCCAAGTTCACACATTCCTGAACTTCTGGCAGCAAATCCTCGAGGGAGAACTGATAAACTCCCGGCATGGAGGCAACTTCTTGCTTTTCAGTCAAGCCTTCCTTGACAAACAAGGGTTGGATAAAATCATCCACAGACAGCTTGGTTTCTCTAACCAGCTGCCGCAGACCGCTGCTTCTCCTCAGTCTGCGATGTCTATAAAATTCCATGCTTCTTCTCTTCTTTCTTGATTATTTCCTTGACCATTTCCTCAACAGAAGGACTCTGGGGCTGGTAATCTACCCTATAGCCGGACTCCTCAACCGCCTGAGCTGTTGTCTGTCCAATCACTGCAATCTGATGACTGGATGCTAGATGGGGCTTAATAGCACAAAAACTCTGCCAAGCCGATGGACTGGCAAAGGTCCAAATCACATCCTCTTGGGACAGATAGGTCTCGAGCTGGTCCTGACCAGCAGGATTTGAGGTCGTTGCGTACATAGGCCAAGCCCAGACCTCATGACCTGCTTCTTTTAACTTCTCAGCCAGACTGGGATTGGACAAGCTACTCTGGGGCAGCAAGATCTTCTGCGTCTGCAGAGCCAAATCCAGCCACTCCTGAATAAAGTCAAGACCATAGTGACTGCTGGCCTGAAAATCACAGGCTCGGCCCAGCTTATCCAGCGCCTGACTGGTCTGCGGCCCAATGGTCGCAATCTGGTAGTCCGCTTTCAGATAGGGACTGAAAGCCTCTACTGCGACAGCACTGGTAA

>c183_g105

GGTTCGATTCCGTCTCGCGCCATAATTTTATAATCGGAAGGGTAGCGAAGAGGCTAAACGCGGCGGACTGTAAATCCGCTCCTTCGGGTTCGGGGGTTCGAATCCCTCCCCTTCCATAACCTTTACGGGCATAGTTTAAAGGTAGAACTAAGGTCTCCAAAACCTTCAGTGTGGGTTCAATTCCTACTGCCCGTGTTTAAATATGGCGGGTGTGGTGAAGTGGTTAACACACCAGATTGTGGCTCTGGCATGCGTGGGTTCGATCCCCATCACTCGCCTATTTAATATTATTGGGGTATCGCCAAGCGGTAAGGCAAGGGACTTTGACTCCCTCATGCGTTGGTTCGAATCCAGCTACCCCAGTTCTTAGGTAATAGATCTGATAAAATAATAAAATGTCTTTAGGTGTTTTATTTCTTTATAGGATGGAAATCGTTAACGATAGATCAATGTCATTTGCGGGTGCTTAGGAAAATAAATAAGTAGTATGTCAAGCGAAAGCTTGATTGTTGGAGGATTTTTTTAGATGAATGAATTTGAAGATTTGCTAAACAGTGTTAGCCAAGTTGAGCCAGGTGACGTCGTTACTGCTGAAGTATTGACTGTTGACGCTAACCAAGCTAATGTTGCAATCGCTGGAACTGGTGTTGAAGGTGTTTTGACTCTTCGTGAGTTGACAAACGACCGCGACGCTGATATCAACGACCTTGTAAAACCAGGTGAAACACTTGAATTGCTTGTTCTTCGTCAAGTAGTAGGTAAAGATACAGACACCGTAACTTACCTGGTATCTAAAAAACGTTTGGAAGCTCGCAAAGCTTGGGACAAATTGGTTGGTCGTGAAGAAGAAGTTGTCACTGTTAAGGGCACTCGTGCTGTTAAAGGTGGACTTTCAGTTGAGTTTGAAGGACTCCGTGGATTTATTCCTGCTTCAATGCTTGATACTCGTTTCGTTCGTAACACTGAGCGTTTTGTAGGTCAAGAGTTTGATGCTAAGATTAAAGAAGTAGATCCAAAAGAAAACCGCTTCATCCTTTCACGTCGTGAAGTTGTAGAAGCTGAAGCTGCAGCAGCACGTGCTGAAGTCTTTGGTAAATTGAATGTTGGTGATATTGTAACTGGTAAAGTTGCTCGCATCACTAGCTTCGGTGCTTTCATTGACCTTGGCGGAGTTGACGGACTGGTTCACTTGACTGAATTGTCACATGAACGCAATGTATCACCTAAGTCTGTTGTATCAGTTGGTGATGAAATCGAAGTGAAAGTTCTTGATTTGAACGAAGAAGAAGGACGCGTATCCCTCTCTCTTAAAGCAACTACACCTGGACCATGGGATGGCGTTGAGCAAAAACTTGCTGCAGGCGATGTGATTGAAGGAACTGTAAAACGTTTGACTGATTTCGGTGCTTTCGTTGAAGTATTGCCAGGTATCGATGGATTGGTTCACATCTCACAAATTTCACACAAACGTGTTGAAAGCCCTAAGGATGCCCTTAAGGTTGGTCAAGAAGTAACTGTTAAGGTTCTTGAAGTGAATGCTGCTGATGAGCGTGTATCACTTTCTATCAAGGCTCTTGAAGAACGTCCAGCTCAAGAAGAAGGACAAAAAGATGAGAAACGCCAATCACGTCCTCGTCGTCCAAAACGTCAAGAAAAACGTGACTTTGAACTTCCAGAAACTCAAACTGGATTCTCAATGGCTGATTTGTTTGGCGATATTGAATTGTAATTAGTTAAAAGAAGTTGAGAAATCTCAACTTCTTTTTCTGATTTTCACTTGTATTTTCAGAGAAAATCAGTTATAATGAATTAGTTGCCACCCTTAGTGTAATGGATATCACGCAAGATTCCGGTTCTTGAGATGGGAGTTCGATTCTCTCAGGGTGGATGACTTAAAATTGGACTCTTCGGAGTCTTTTTTTACTCTTTCTTGGAGGAGAATGCAGTGACAGTAAAATTAATCGCCCATACCTTGATTGAGAAAGACGGAAAGTATTTACTTATCAAACGCTCTAAAATAAAGCGCGGCCTTCCTAATGTCTATCCATCTTATTGGGATATTCCAGGTGGAAGTGTAGAAGAGAATGAACTGCCCAGAGAAGCAGCTCTACGTGAGGCTATGGAGGAGGTTAATCAAAAGATTCGGATTGATAAGATTATCCACGAAGATAGTCAGTTTGATGCTAGCCAAGAAACTGTTTTCACACGCTTAGTTTATACGGGTAGGATTATGGAGCAGCATGATATCATATTAGATCCAGAAGAGCATTCAGACTTTGTCTGGATTACTTCTCTGAAAGACCTAGAGGATGAGCTCATCGTACCTTATTTGATTGATATTTTTGCTGTTAAATCCATATAAAACAACTAAAAAACATCCCCTTTATTAGGAGATGTTTTTTTATAATTAGTTTTTAGATGCTTCTTGGAATTCAGGATTTTTCCATGCTTCATCAATAATTGCTTGCAATTCTTTAGCAGAAGCTTGCATTTTTTGAGTCTCAGCATCGTTCAGTGGGATGTTTACTGGACGTACGATACCGTGAGCACCAACGATAGCAGGTTGACCGATGAAGACATTTTCAACACCGTATTGTCCTTCTTGGAAGACTGACAATGGCAATACTGCATTTTCATCGTCAAGGATAGCTTTTGTGATACGAGCCAAAGCAACCGCGATACCGTAGTAAGTTGCTCCTTTTTTGTTGATGATTGAGTATGCTGCGTCACGGACGGAGATGAAAAGATCAACCAAGTCTTGTTCGTTCAAGTCGCGGTTAGCTTGCAACCATTGCTCCAATTTGACACCTGCAACGTTAGCGTGTGACCAAACGGCAAACTCAGAGTCACCGTGCTCACCCATGATGTAGGCATGGACAGAACGAGCATCGATACCAATCTTTTCAGCAAGTGCTTGACGGAAGCGAGCTGAGTCAAGTGAAGTACCTGAACCGATAACACGCTCTTTAGGGAAACCAGAGAATTTCCAAGTTGAGTAAGTCAAAACGTCAACTGGGTTAGCAGCAACGAGGAAGATACCGTTGAAGCCTGATTCTACGACTTGAGTAACGATAGATTTGTTGATAGCCAAGTTTTTACCAACAAGGTCAAGACGAGTTTCACCTGGTTTTTGAGGTGCACCTGCAGTGATAACAACGAGGTCAGCATCCGCACAGTCTGCATAAGTAGCTGCATAGATTTTTTTAGGTGAAGTGAAGGCAAGGGCGTGGCTAAGGTCTTCAGCATCACCGACAGCCTTTTCAAATAATTGAGGGATTTCAATGATACCAAGTTCTTGAGCAATACCTTGGTTTACAAGAGCAAATGCGTAAGATGAACCTACAGCACCGTCACCAACAAGGATGACTTTTTTATGTTGTTTAGTAGCAGTCATTTCTAAACATC

>c183_g106

GGAAAATCAAATGGTTGTAAAAACAGTTGTTGAAGCTCAAGATATTTTTGACAAAGCTTGGGAAGGCTTCAAAGGTGAAGACTGGAAAGAGAAAGCAAGTGTTTCTCGCTTCGTTCAAGCTAACTACACACCTTATGATGGAGATGAAAGCTTCTTGGCAGGTCCTACTGAGCGCTCACTCCACATCAAGAAAATCGTAGAAGAAACAAAAGCTCACTACGAAGAAACTCGTTTCCCAATGGACACTCGTCCAGCATCTATCGCTGATATTGACGCTGGTTACATTGACAAGGACAACGAACTGATTTACGGTATCCAAAACGACGAACTCTTCAAATTGAACTTCATGCCAAAAGGCGGTATCCGCATGGCTGAAACTACTTTGAAAGAAAATGGATACGAACCAGATCCTGCTGTTCATGAAATCTTTACTAAATATGTTACTACAGTAAATGATGGTATCTTCCGTGCTTACACTTCTAACATCCGCCGTGCTCGTCACGCCCACACTGTAACTGGTCTTCCAGATGCTTACTCACGTGGACGTATCATCGGGGTTTACGCACGTCTTGCTCTTTATGGAGCTGACTACCTCATGGCTGAAAAAGTTCGCGACTGGAATGGTTTGACTGATATTGACGAAGAAACAATCCGTCTGCGCGAAGAAATCAACCTGCAATACCAAGCACTTGGTGAAGTTGTAAAATTGGGTGACCTTTACGGAGTTGACGTTCGCCGTCCTGCCTTCGACGTTAAAGAAGCTATCCAATGGACTAACATCGCCTTCATGGCTGTCTGCCGTGTCATCAACGGTGCTGCTACTTCTCTCGGACGTGTGCCTATCGTTCTTGACATCTATGCTGAACGTGACTTGGCTCGCGGTACTTACACTGAATCAGAAATCCAAGAATTTGTTGATGATTTCGTTATGAAGCTTCGTACAGTGAAGTTTGCTCGTACGAAAGCTTACGACCAACTTTACTCTGGTGACCCAACCTTCATCACTACTTCTATGGCTGGTATGGGTAACGATGGCCGTCACCGTGTTACAAAGATGGACTATCGTTTCCTTAACACACTTGACAACATTGGTAACTCTCCAGAGCCAAACTTGACCGTTCTCTGGACTGATAAACTTCCATACTCATTCCGTCGCTACTGTATGCACATGAGCCACAAGCACTCTTCTATCCAATACGAAGGTGTAACAACTATGGCTAAAGACGGCTACGGTGAAATGAGCTGTATCTCTTGCTGTGTGTCTCCACTTGACCCTGAAAACGAAGAACAACGCCACAACATCCAATACTTTGGTGCTCGTGTAAACGTGCTGAAAGCCCTTCTGACAGGTCTCAACGGTGGTTACGACGATGTTCATAAAGACTACAAGGTATTTGACATCGAGCCTATCCGTGACGAAGTTCTTGAATTCGAATCAGTTAAAGCAAACTTTGAAAAATCACTTGACTGGTTGACTGACACTTACGTAGATGCCTTGAACATCATCCACTACATGACTGACAAGTACAACTACGAAGCTGTTCAAATGGCCTTCTTGCCAACTTACCAACGCGCTAACATGGGATTCGGTATCTGTGGCTTTGCCAACACTGTTGATACCTTATCAGCTATCAAGTACGCTACAGTAAAACCAATCCGTGACGAAAATGGCTACATCTACGATTACGAAACAATCGGTGAATACCCACGTTGGGGTGAAGACGATCCTCGTTCAAACGAATTGGCTGAATGGTTGGTTGAAGCTTACACAACTCGTCTACGCAGCCACAAACTTTACAAGAACGCTGAAGCAACAGTGTCACTCTTGACTATCACATCTAACGTTGCTTACTCTAAACAAACTGGTAACTCACCAGTTCACAAAGGAGTTTACCTCAACGAAGATGGCAGCGTGAACTTGTCTAAATTGGAATTCTTCTCACCAGGTGCTAACCCATCTAACAAGGCAAAAGGTGGCTGGTTGCAAAACCTTAACTCTCTTGCTAGCTTGGACTTCAGTTACGCAGCTGACGGTATCTCATTGACAACTCAAGTATCACCTCGTGCGCTTGGTAAGACTCACGACGAACAAGTAGACAACTTGGTTACTATCCTGGATGGCTACTTCGAAAACGGTGGTCAGCACGTTAACTTGAACGTCATGGACTTGAAGGACGTTTACGACAAGATTATGTCTGGTGAAGACGTTATCGTTCGTATCTCAGGTTACTGTGTAAACACTAAGTACTTGACTCCAGAGCAAAAAAACTGAATTGACTCAACGTGTCTTCCACGAAGTGCTCTCTATGGACGATGCTTTGAGCTAATAAACGACTAAGGACTTACAAACCATTTGAAAGAAGATTGGAAATCTCCAATCTTCTTTTTTGTATTTCAGACTTCGAGTGACTGCCAACTTG

>c183_g107

CTAAAATCCTGCTTACCCGAAACTGGCAGCCATCTGAGCTGAACAGCAAAAATCACAATCAGCATGTAGCCTAGCCAAAAGCTGGGCATGGAAACGCTAGAAAAGGATAAAAAGCGAGTGACCTTATCAAAGAGAGAACCCTTGTAGACTGCACTAAAAATTCCCAAAGGAATGGACGTAAGCAAAATGAGAGCAAAGGATGTCAAGCCCAGAGAAAGGGTCGACTGGAAACGCTCCAGCACCAAGGGCAGGACAGGAACCTTTAATAGATAAGACGTTCCAAAATCCCCACGAAGGGCCTTGGTCAGCCAACTTAGATACTGCTGGGGCCAAGGTTGATTGAGGCCCAGATATTCTCTAGCTTTTTCCAAGGATTGAGGAGTCACTTGAATCTTAGATACCCTTAGATAATTTTCTGCTTGATCAGCCGAGGATAGCTTGGCCAAGAGAAAGGTCAATACTGATATCATCAACAGGGCAGCTAAGAAAGAAACTATTTTTTTTAATAAATCTTTTTTCATAGTCCTTAAAAGAAAAGGGGCGGCTGATAAGCTTTTCAACTCAGCAGCACTACTCCCTTTATATTAAAATTTGCAGTTATGAAGTCAGTTTTTATT

>c183_g108

GACCTGTTTTAGCATTGGTGACACTGGACAGGATAAGGGCAAAGATAATGGAAACTGGCAAATATTTGAGGAAACGCTCTGCCATAGGCGGAAGACCCTTGTACTTGACTAGGATAAAGGGCAAAATCCGCGGAATCCAAGTGACCAGGGCTGACAAAAGAATGGCCAGCAAAATATATTTACTTATCATCCAAAAGCACCCCCACTGTACAGCCTAGCAAGGTTGCAAAAAGCACCGCCAGCGAATTTTGAAGTAGCATAGTTAGGACTAAGTAGGCAAGGCCCACAACACCCAGAACTAAAAAGAGCTTCTTTATCTTGACCCGTCGCAGCATGATTGTAAACTGAGAAGAGAAGATACCGATAAACATGGAGACTAGGGCAAAATCCAGGCCAAAGCTTTCAGGATTGGGCAGAAGGGCGCCGAGATCTGTCCCAAAAACAGTTCCCACAATCCAAGAAGCATAACTCATAAAATTGTTCCCCATCATCCAGCTAGCAGCAATCTCTTTTGTATGCACCTGCTCCCCCATCAAAACTCCATAAGATTCATCAGTCAAAAAAGATCCAATGACAATATTCTGCATCAAGTTAGACTTCCGAAAAAAAGTTGATGCATGCAGACAGAGCAGAAGATGCCGGATATTGATGAGAAAAACAGTCAGTGCGATGGCCAAAACCGGCGCCTGCTGGGCAAATAAACCAATCATAGCAAACTGGGCACTGCCGGCATAAACCAGAACACTCATCAACCCCATCTCCAAGGGATTCATATAAGGCGATGCCATGATGCCGCAGGCTAGACCAATCCCAATATAGCCCAGAGCCGTTGGCACCGCCGCTTGTGCCCCTTGTTTAAATGTCTGTCCGCGCATTCTTCTCCTTTACTCCATGGTTTCAAAAGCCAGACTTGGCTTAGCATTCAGGTCCAAACTGGCAAAATTCTCCTTGTTCCACTCACTGACACTGGCATAGGCAATCATACCAGCATTGTCACCGCAGAGGCGAAGCGGTGGAATAATCACCTTAACATCCTGAATCTCAGCTGCCAAGCGTTCTCTCAGTCCTTGATTGGCCGCAACACCGCCCGCCACGACCAGAGTCTTGACTGGATATTTTTCCAGCGCTTTCTTAGTCTTGGCCATGAGAATATCCATGACCGCCGCCTGAAAGCTTGCTGACAAGTCTTGATTAGATAAGACCTCTCCTTTTTGCTGGGCATTGTGGTGCAGGTTGATAAAGGCCGACTTGAGACCGGAAAAAGAAAATTCCAGATTGTCTTCCTTAATCATGGCCCGCGGAAAATCATAAATATCCTGTCCCTCATGAGCCAGCAGATCAATCTCCCGGCCTGCTGGATAGGTCAGCCCCATGACTCGACCGACCTTATCATAGGCTTCACCGACCGCATCATCGCGAGTTTCCCCCACAATCTTATAGTCACCAGCCTGACTAACATAGACCAGCTCGGTATGGCCGCCACTGACCAAGAGAGCCAGCAAGGGAAATTCCAAGGGCTCCACACTCTGGGCTGCCATCAGGTGTCCAGCCATGTGATTGACCGGAATCAAGGGAATATCATGAGCCCAAGCAAAAGACTTGGCCGCTGCCAAGCCGACCAGAAGCGCCCCAACCAGACCTGGACCGTAGGTAACAGCCACAGCTGTGACTTGCTCCTCGCTAATCCCTGCTTCTGCCAAGGCCTCCTCAATGCAAACCGTAATGACCTCCACATGATGGCGGCTGGCCACTTCTGGTACCACTCCGCCAAAACGCTTGTGGCTCTCAATCTGGCTGGCAATAACATTGCTCAAAAGCTCAGTCTCATTCTTCAGAACCGCCACACTGGTCTCGTCACAAGACGTCTCAAAAGCTAAAATATATCTATCTTTCATGGATCTCTCTTTTCATGACAATCGCATCTTCCACTGGCGCATGATAGTAGGCCTTCCGCCGCGCGATTTCTTCAAATTTTTCTTTTTTGTAAAATAGCAGAGCTGGCTTGTTGGACTCCCTCACTTCGAGGAAAATTTCTTTGTCTGCTGGCAAAAAGGCAAACAAGGCTGTCGCGATTTTCTGTCCCTGATAGCTAGGCAATACAGCAATCTGCAGAACCTCTGCTTCAAAGTCTGTCTCCTGCCAGACTAAAAAGGCAACAAGCCGATTCTCATCTTCTGCCAGCGCACAGCTGTTCACATCCGAGCGCAGAACCTCTTCCACTTGGCCTGCGGTCCAAGGACTGACCTCATAGACTGCAAGTAAGAGCTGTTCCAGCTCTTTTGCGAGAGTTGCTGCGTCCATATCTGAGCTATCGCTCCATTTTCTCATCGTAATCATAGGCGCTGAATGTAAGAATCACTGGATTCCTGATGTGTTTTGAGCCAATTTTCCTCAGCCTCTACCCGCTTGAGGTAGTTAGGAACAAAGTCATGAATGGACTGGGCTGGCAAGTCTAGACCGAGACGACCAACAGCTACGGCATCTGGCAACGTTGACTGAATGGCAGCCTGAGGAAGAGCCGCTTCAATCTGCTCCGCAAAAGCTGCTGTCTCTCCGGCAAAGGTGACCGGCTGGTTAGCAGCGTCAGCTATTTCCAACACCTCTGCCAAAGGCAGATGAGCTTCTGGTCGTACAGCCTGACCAGACTGGTAAAAGCCAGCATAGACATTATTGCGGCGGGCATCCATGACAGGAATGACCAAACCTTCCACCTGCTCTGGGACTAAAGCCAGCAAACTAGACACACCGACCAGCTCAATCTTGAGGGTATGAGCCAGAGTCTTGGCTGTCGCCACCGCCATTCGCAGACCTGTGTAGCTGCCCGGACCCTGAGCAACTACGATACGATCCAAATCCGTCGGCTTCATGTCCAGACTATTCATCAGAAAATCAATAGCCGGCATCAGGGTAATGCTGTGGTTTTTCTTTATATTTAAAGTCATCTGCGCCAGCAAGGTCTCGTCCTCTAAAATCGCGAGCGTCAGCGCCTTACTTGACGTATCAAAAGCTGCAATCTTCATGCTTATCCTCTATTTTTCCTCTTGCCGCCAAAGTGAGCGGCGTTTTAATTTGTCTTCGTAGAAACTATCTACCAAAAATTCCTCTAAAATCTGGCTAAAAGCCTCCAAATTAGGCACTTGCTGCGCCAAGTGTTTTCCAAAAATCGCTTCTTGCTCAGCAATCTTGGGCAGCAAATCCGCTCCAGCAGCAGTCAAGCTCAAGCGAGAACTGCGCTTGTCCTTGCTAGAAATTTCCTTCTTGACCAAGCCTTTTTTAATCAAAGCCTGCACCAAGCGACTAGGGCTTTTTTCCTCACAAATCAGGGATTCCCCCAAGCCCTTGAGAGACAGCGGAGCATGTTCGCCCAAAACGAGCAAGACCTCGCTTTGATTGGGCGTAATTCCCAAAGGCTCTAGCAACTTCCCATATTCTCTCGTCGCTAAGCTCTC

>c183_g109

GAGACTGGGACAAAATGTCTCAGTCTCGCTTTATCGTATTCACAAACTTTTGATTCCAAACTGTTTATAAAGAATCAAAAATTCGCGGTTCAGAACCTTATTTAATGTGTTTTTCTAAGTCTTCTTGGGCTTTTTTATTGGATGCTTCCTTTTCTTTTTCCCATTTCTTAAGAGCCGCTTCATAGTCTTTAGCTGTAACAATATCATTTTGGAGCTCCATGTTCTTAAAGACATAGGCATCTCCTTTAATTCCGACATAAGAGTAAGACTTGGTAAATGGCACAACCTTCTGAACGACTGGTGAACCACCACCAGAAACACTAGGAATAACAATGGAGCTATCCGTCAACCAAGCCTGAGCTGCTGCGTACTTGGTGTAACGAGCATCTGTATCTTGTTTTTCTTGATCAGCTTCATCCAGCAAAGCCTTGTATTCATTCAAGCCAACCTTATTTGCCACATCAGCATTTTTCCCTTTTTCTATACCAATATTATCTGCAGCATCACCTGTTTCAGGGTTAAAGATATTCAGATAAGTTGATGGATCTTGGTAGTCTGCTGACCAGCCAGACATGTTGAGATCGTAGTCTTTCTGAGCTGCTGTTTCTGCAAAGTAGGCAGAATTATCAAAGTCATCTGTTGACATTTGCTGAACATCAATAACGACATTTTCTTGGCCAAGCGCTGATTCGACAGACTGTTTGAAGGAGTTCGTCCGTTGTACCAAAACCTTGTCAGACTGATCGACAGGGACGTCAATATGGATTGGGAATTCTACACCTTCAGCTTGCAAGGTTTCTTTTGCCTTGGCAAATTCCGCCTTGGCTTTATCAGCATTGTAGATACTATCCTGAGCATCCGCCAGCTTAATGCCATTCCACTCATCTCCATAAGATGAAAGTTGGCTCTCTACTACATCAGAGAAGTCCTTACCGTCCACCTGCACAAAGCTAGGAGGAACAAGCAAGCTACGGATGAGTTTAGTCGCTCCATCTTTACCATTCATCTGAGCTCCAAAAGATGTCCGGTCAAAGGCAAAGTTAATCGCCTGACGGAAGTCTTTATTTTGAATAGCAGCTGTAGTTGATGCCTTTTGAGCATCTGATGTCTTAGACGAATGATTATAAGTCTTACGATTCAGATTGAAAGCAAAATAGAAACTAGTTGAATTCTGTGGTGTGTAAATAATATTGTCTTTGTACTGCTTTTCTACAGAAGCATAGTTAGAGCTCGTAGGGTAAAGCCGTGCTTGAGAATAAGCCCCGTCTGAGAAGTTGCGGATCAAGGATTCCTGATCCGAACCATCAAAATAAGTCAGTTTTATATTTTCAACCTTGACATTGTCCTTATCCCAATAGTTCTGGTTTTTAGCATATTCAATGACAGACTTAGAAGTGAATGCTTTCAGAACATAAGGTCCGTTATAAAGAATGGAAGAAGGTTTTACGGTACCAAAATCTTTGCCTTGTGACTTCAAGAAATCCTCATTGATGGGGAACAGAATCCCCATGGTCGTCTTAGAGTTCCAGTAGCTTTCTGGCTGGTTGAGCGTGTATTCGACCGTGTGGTCATCAACTGCTTTTACTCCAACTGTTGAGAAGTCTTTAGACTCTCCTTTGACATAGGCATCCAAGCCTTTGATAGAGCTTTGGACGATAGGAAGAGCTTCAGAATTTTCATCTGCCGCGTGCTTCAAACCTGCTACAAAGTCTTGTGCTTTAACCTCTGCATATTCTTCACCATCTGATGTGTACCACTTAGCATCTTTACGAAGCTTATAGGTATAGGTCAAACCATCCTTTGAAACAGTCCAGTCTTCCGCAATAGATGGAACCAAATTGCCGTATTTATCATTTTCTAAAAGGCCGTCCACCAAGTTTGTAATAACATCGGACGTAGTAGCTCGATTTGAATTGACATAGTCCAAAGTATCAGGGTCACTCAAATAGACATAAGAATAGGTACTGCTTGCACTGGAATTTCCGCAAGCGGCTAAAATCAGTGCTGACACGACTGTCAGTCCAGTAATGGCCAACCATTT

>c183_g11

GGCAACTTTAGATAAAAATCTTTTGTTAGAGATGTTCCGTAAGATGGAAGAAATCCGTCGCATGGACTTAAAAATTGCTCAACTTGTAAAAAAAGGAAAGGTTCCTGGGATGACTCACTTCTCAGTAGGTGAGGAAGCAGCCAATGTCGGTGCTATGCTGGCCTTAAATGAGGATGATTTGCTGACTTCTAATCACCGGGGACACGGTCAAGCTATCGCCAAAGGAATCAACCTCAACGAAATGATGGCTGAAATCCTTGGTAAATACACTGGAACCTGTAAAGGAAAAGGCGGCTCTATGCACATTGCTGACCTAGATGCTGGAAATCTTGGTGCTAATGGTATCGTAGGTGGTGGCATGGGAATTGCTGTCGGAGCAGCTCTGACCCAGCAAATGCAGAAGACAGGTAAGATAGTTGTCTGCTTTTTTGGTGACGGGGCTACCAACGAAGGTGTCTTCCACGAAGCAGTCAACATGGCTTCCATCTGGAATCTACCCGTAATTTTCTATTGTATCAATAACGGCTATGGTATTTCTGCTGACATCAAGAAAATGACCAATGTAGAGCACATCCACGAGCGCAGCGCAGCTTATGGCATTCCAGGAATGTTCATCGAAGATGGCAATAATGTTTTGGATGTCTATGAAGGCTTCCAAAAAGCTGTAGAGCATGTTCGCAGTGGTAAAGGACCTGTCTTGATTGAGAGCGTGACTTATCGTTGGTTGGGACACTCGTCTTCCGACCCAGGTAAATACCGTACTCGTGAAGAAGTAGAAGAATGGAAGAAAAAAGATCCAATCGAAAATCTTCGTAAATACTTGCTGGAAAACAAGATTGCAAGCGAGGAAGAGCTGGAAGCTATCCAAGCTAGAGTAAAAGAAGCAGTAGAAGCGTCTGTGAAGTTCGCAGAAGAAAGCCCGTTCCCGCCGCTTGAATCTGCCTTTGAAGATATTTACGCAGACTAAAGAGAGGAGAAAAAGAAAATGGAAACTAAAACTATGTCTTTTCGTGACACCATTATCCTCGCTATGTCTGAGGAAATGCGTCGCGATAAAAATGTATTCTTGATGGGAGAAGATGTCGGAGTCTTTGGTGGAGATTTCGGAACATCTGTTGGTATGCTGGAAGAGTTCGGTCCAGAGCGTGTACGTGACTGTCCGATTTCGGAAGCAGCGATTTCTGGAGCAGCAGCTGGAGCAGCTATGACAGGGCTGCGTCCAATCGTAGACATGACCTTTATGGACTTCTCTGTAATCGCCATGGATGCTATTGTTAACCAAGCTGCTAAAACGCGCTATATGTTTGGCGGAAAAGGGCAGGTGCCAATGACTGTCCGCTGTGCAGCTGGCAATGGAGTTGGCTCTGCAGCTCAGCACTCGCAGTCTTTGGAATCTTGGTTTACCCACATTCCAGGTCTTAAGGTAGTCGCTCCAGGAACTCCGGCTGATATGAAGGGGCTTCTCAAGGCTTCTATCCGAGACAACAACCCGGTCATTATCCTGGAATACAAGTCTGAATTTAACCAAAAAGGCGAAGTGCCACTGGATCCCGAGTATGTTATTCCGCTCGGTGTAGGTGAAATCAAAAAAGAAGGTACCGATGTAACAGTTGTTACCTACGGGAAAATGCTTCGCCGTGTCATGCAGGCAGCTGAAGAATTAGCAGAAGAAGGTATCTCTGTAGAAGTGGTTGACCCACGTACATTGGTGCCGCTTGATAAGGATATTATCATCAATTCTGTTAAGAAGACTGGAAAGGTCGTTTTGGTCAATGACGCCCACAAAACCAGCGGTTTTATTGGTGAAATTTCAGCGATTATTTCTGAGTCTGAAGCATTTGACTATCTAGATGCGCCAATCCGCCGCTGTGCAGGTGAAGATGTGCCAATGCCTTATGCGCAAAACTTGGAAAACGCGATGATTCCGACCGTTGAAAGCATCAAAGACGCTATTCGGAAGACATATCATAAAGAATAAGGTATAATGAAGAAAAGGCTTTCTTATGAATTATATTACATAAATTATTTTATGTAATATAATCTTGTGTAGAGGGTCAAAGTAGCTAAAATTTTACTAATTACAGACAATCGTTATTCGGATCGATTTTATCCGATGAGCGAATTAGTAAGCCTACAAGGTTGATTGCCGATAGCGGTAACCGTAGCAACTTGAGATAGGTTTGTATCCAAGTTACTGTAGAGTTGAACATGGGCTAAAGTCTGTGTGAAAAAGATAAACTTTTCTAGAAACGAAAATTTCTTCGTTAAGTTTTCTATTTTCACTTTGATTTTTAACGCCTTAGTATCATGTAATTGAATTCGGACGGAGGATTGCGAAAAAAAGATAGATTTCCTTGTGTTCATTGAACACAAGGAAATCTCCTATTTTTTCATTGTCCTCTGAGTCCTTAATATCTTATAATAGAATTGGAGAGGTCATGGCTGATGATAAGCTAAGAGCGACTCCTGCAGCTAGAAAGTTAGCGGATGATTTGGGAATCAACCTCTATGATGTTTCTGGCTCAGGCGCAAACGGTCGTGTCCACAAAGAAGACGTGGAAACTTATAAAGATACAAATGTGGTGCGCATTTCACCACTGGCAAAACGAATTGCCCAAGAACACAATATTGCTTGGCAAGAGATTCAAGGAACTGGCCATCGTGGCAAGATTATGAAGAAAGATGTTCTTGTTTTCTTGCCTGAGAATGTTGAGAGCGATACAATCAAATCTCCTGCTCAAATTGAAAAAGCGGAAGAAGTGCCTGATAATGTCACTCCTTATGGTGAGATTGAGCGTATCCCAATGACGCCGATGCGGAAGGTCATTTCTCAGCGGATGGTAGAATCTTACCTAACTGCGCCAACCTTCACCCTCAACTATGATGTTGATATGACAGAAATGCTGGCCTTGCGTAAAAAAGTGCTGGATCCAATCATGGAAGCAACTGGCAAGAAAGTAACTGTCACAGACCTGCTTTCGCTGGCTGTTGTGAAGACGTTGATGAAACATCCTTATCTCAATTCGACCTTGACAGAGAACGGCAAGACCATTATCACACACAACTATGTCAACCTTTCAATGGCAGTCGGTATGGATAATGGCCTGATGACACCAGTTGTCTACAATGCAGAAAAAATGAGCCTATCAGAGCTTGTGGTAGCCTTTAAAGATGTAATCGGACGTACCTTGGAAGGCAAATTAGCTCCAAGCGAGCTGCAAAATTCAACCTTCACAATCAGTAACTTAGGTATGTTTGGCGTTCAGTCTTTTGGGCCAATCATCAACCAGCCAAACTCTGCTATCTTGGGTGTGAGCTCAACGGTAGAGAAACCTGTCGTTGTTAATGGCGAAATTGTTATCCGTCCAATTATGAGTCTGGGCTTGACGATTGACCACCGTGTAGTTGACGGAATGGCTGGAGCTAAGTTTATGAAGGACTTGAAGGCTTTGATTGAAGACCCAATTTCAATGTTGGTATAAGATTTTCTTTGCTAGTTTAAAAAGTAAAGAAACGAAACACTCAGCAAGTGAAAAACTAAATTAGAAAAGGAAAGAAAAATGGCTTTAGAAGTAATTATGCCAAAAGCCGGCGTGGATATGACCGAAGGGCAAATTGTCCAATGGAATAAGAAAGTCGGCGAATTTGTCAAAGAAGGGGAAATCCTTCTGGAAATCATGACTGACAAGGTCAGCATGGAATTGGAAGCTGAAGAAGACGGCTACCTGATTGCTATTCTCAAAGGTGACGGCGAAACTGTTCCCGTGACGGAAGTCATCGGTTACTTGGGAGAAGAAGGAGAAAACATCCCAACCGCTGGCGGCTCTGCTCCTGCGGAAGCACCAGCTCCTGCAACAGCGGCAGTAAGTACAGATGATGATAAGAGTGATGATGCTTACGATATCGTTGTTATCGGTGGCGGACCTGCAGGTTATGTAGCGGCTATCAAAGCAGCCCAGCTGGGTGGCAAGATTGCCTTGGTTGAGAAGTCTGAGCTCGGCGGAACCTGCCTGAACCGTGGCTGTATCCCTACTAAGACTTACCTGCACAATGCTGAAATCATTGAAAGCCTTGGTCACGCTGCTAATCGCGGTATCATAATTGAAAATCCAAGCTTCTCAGTGGACATGGACAAGGTTCTGGAAACCAAAAACAAGGTTGTTAATACGCTTGTTGGCGGTGTTGCTGGCCTTCTTCGCAGCTATGGTGTAGATGTTCATAAGGGAATTGGTACCATTACTAAAGATAAGAATGTTCTTGTAAACGGTAGCGAGCTGCTAGAAACGAAGAAGATTATCTTAGCTGGTGGTTCCAAAGTCAGCAAGATCAATGTCCCAGGAATGGAATCATCTCTCGTTATGACCAGCGATGATATTCTGGAAATGAACGAAGTGCCAGAAAATCTGGTAATTATCGGCGGTGGTGTTGTCGGTATTGAGCTTGGCCAAGCCTTCATGACATTTGGTTCAAAAGTCACTGTTATCGAAATGATGGACCGTATCGTACCTGCTATGGATGCAGAAGTTTCTAAAAACCTTCGCCTCATCTTGGAGCGCAAGGGCATGACAATCCTGACTGAAACTAAGTTGGAAGAAATCATCGAAGAAAACGGAAAACTTCGTATCAAGGTTGAAGGAAAAGAAGATATTGTTGCAGATAAGGCTCTGCTTTCTATCGGACGCGTGCCAGACCTAGAAGGTATCGGTGAGGTTGAGTTCGAACTGGATCGAGGTCGTATTAAGGTCAATGAGTACATGGAAACTTCTGTTCCAGGTATTTACGCACCAGGTGATATCAATGGTACTAAGATGCTGGCTCATGCAGCCTTCCGTATGGGTGAAGTTGCTGCTGAAAATGCTCTGAAAGGTAACCACCATGTTGCTAAACTCAATCTGACGCCTGCAGCTATCTACACCCTGCCAGAAGTAGCAGCAGTTGGTTTGACAGAAGAGCAAGCTCGTGAGAAATACGATGTAGCAATCGGTAAGTTCAACTTCGCTGCTAACGGCCGTGCTATCGCTTCAGATGCGGCACAAGGCTTCGTTAAAGTCATTGCTGACAAGAAGTACGGTGAAGTGCTCGGTGTTCATATCATTGGTCCTGCAGCGGCAGAATTAATCAATGAAGCATCAACCATCATCGAAATGGAAATCACTGTAGAAGAAATGCTTAAAACTATTCATGGTCACCCAACCTTCTCAGAAGTTATGTACGAAGCTTTTGCAGACGTACTGGGGTTGGCAGTTCACTCACCTAAGAAAAAATAATTTAAAAGATAGATTAGACTGGACATAAATCATTGTTCCAGTCTCTATCGTATAAAGAGGTAGCTTATGAAATACATTGTTAACTACTCAAATGATACAGCTTTTAATATTGCTCTGGAAGAATATGCTTTCAAACACCTCTTAGATGAGGATGAAATTTTCCTGCTCTGGATTAACAAACCTTCTATCATCGTAGGGCGTCACCAAAACACTATCGAAGAAATCAACCGTGATTATGTTCGTGAGCATGGCATTGAAGTTGTGCGTCGTATCAGTGGTGGAGGAGCCGTTTATCATGATTTGAACAACCTCAACTACACCATTATCTCCAAGGAAAGTGAAGATCGTGCCTTTGACTTCAAGAGTTTTTCTACGCCGGTTATCAACACCTTGGCAGAGCTTGGGGTAAAGGCTGAATTCACCGGCCGTAACGACCTAGAGATTGATGGCAAGAAGTTCTGTGGCAATGCGCAGGCCTATATCAACGGTCGTATCATGCACCACGGCTGCCTACTCTTTGATGTTGATTTGTCTGTCTTGGCCAATGCGCTTAAGGTTTCTAAAGACAAGTTCGAATCAAAAGGAGTTAAATCCGTCCGTGCCCGTGTAACCAATATTGTCAATGAATTGCCAGAAAAGATTACCGTCGAAGAATTCCGAGACCTGCTTCTGGACTATATGAAGAAAGAATATCCAGAAATGACAGAGTATGTCTTCTCTGAGAAAGAACTGGAAGAAATCAAGCAAATCAGAGATAGCAAGTTTGGAACTTGGGACTGGAACTACGGTAAATCACCTGAATACAATGTGCGCCGTGGCACCAAGTTTACCAGCGGTAAGGTAGAAATCTTTGCCAATGTTGTTGAATCTAAGATTCAGGATATTAAAATCTATGGAGACTTCTTTGGTATTGAGGACGTAGCAGCAGTAGAAGATGTGCTGCGCGGTGTTAAATATGAACGCGAAGATGTCCTGAAAGCTCTTGAAACGATTGATATTAGCCGCTACTTTGCTGGAATCAGCAGAGAAGAAATTGCCGAAGCTATCGTCGGTTAAAATATGACATAAAGAAAACCAAGCTCTCGAAGAGTTTGGTTTTTCGTGTAAACGATATATTTCAAATCAACAACATTCTTACGTTTCCTTAACTAGCTAGTTTTCTCTAGGCTTTATCTGCTAGATATGATAAAATGATAGGAAATTAGAGAAAGGGGTGCCTTGATGCAGAAAGTCCCAGTAGAAAGCCTTGGTCTGTTTGAGCAGTTGGACCGCACAGTTGTAGCTTTTCTGAAGAAAAAACCATCTCCTAATCCAGACAATTTTTATGTTAGCATATCTCCAGAAGATTATGAGAAAAAGAAAGAAGAATTTGAAAAATCAGGCTATCAAGCTGTGAAATTACCGCTTGGCATGGCGTTGGATAATGTTATTCAGCAACCTTCTTTCCAAAACCTAGTCATTGGCGGGCTCTACATGGTAGATGTTTTCGTTCCCAAGGAAGACCTGATGTCCTTGAAGGATCTTGTAGACAGCTTTTGTATCATGTTTGCAGCAGCTAATAATCGAATAGAGAATATCAAGGCTTACGATCTGATGAAGAGTAAAACGGTCTATTTTATCGGAAAACTCTTCACGGATAATCCTCAGCCAGGCGATGAAGTTAGCTTCGAAGGGCTGAATAGAGAGACAGCAGAAGGCTCCTACGAAGCAGTCAAATGTTTCCTAACTAGAGAAAGCGCTGAAAAGTACAACTCCAAGAATCGACCTGTCACAGCAGCAAATCTAGAACATCTCAAGCACTTCTGGGGCAAACCGCTGATTGTTGAACCCCATCGTAATTATTGGATTGAG

>c183_g110

ATTACTCACTTTATTTACAGCCCTCCTGATCGCCTTGGGAACAGCCCAAGTTATCCAAGCCGATGACTACCTGAGAATCGGTATGGAGGCAGCCTACGCCCCCTTCAACTGGACTCAAGATGACGATTCCAATGGTGCTGTAAAAATCGAAGGCACCAACCAATATGCCAACGGATACGATGTGCAGATTGCCAAAAAAATTGCTCAAGAGATGGGCAAGGAACCTCTGGTTGTCAAGACTTCTTGGAATGGCTTAATTCCTGCTCTGACCTCCGGCAAAATTGACATGATTATTGCAGGCATGAGTCCAACTGCCGAGCGGAAAAAGGAAATCGCCTTTTCTAACAGTTACTACACCAGCGAGCCAGTTCTCCTAGTTCGTAAAGATGGAAAATATGCATCCGCTAAGACTCTAGAAGACTTCAAAGACGCTAAAATCACTTCCCAGCAAGGAGTTTACCTCTACAATCTCATTGACCAACTGCCTGGTGCCAAAAAAGAAACAGCTATGGGCGATTTTGCTCAGATGCGTCAAGCCTTGGAATCTGGAGTTATTGATGGCTATATCTCTGAGCGGCCAGAAGCCTTGACTGCTGAGACTGCCAACTCCAACTTCAAAATGATTCAGTTTGAGAAAGGCTTTGAAGTTGGCGAAGAGGATGCTTCTATTGCCATTGGTATGCGCAAGGATGACAGTCGTATCGAGCAAGCTAATGCAGCAATCGCTAAGATTTCTACGAATGATCAAGTCAAGCTGATGGATGAGATGATTCAGAAACAGCCTGTTGATACGGATAGTGAAACAACGAATGAATCATTCTTCAGTCAAGTGGCTAAGATCCTGGCTGAAAACTGGCCTCAATTCCTACGCGGAGCTGGTTTGACTCTACTCATTTCGATTACAGGGACCATCGCAGGTCTCATCATCGGACTGCTGATTGGCGTTTATCGGACGGCACCAGCTTCTAAGAATAAACTCCTAGCATCATTGCAAAAAATCTTCGGCTGGTTCTTAAATGTCTATATTGAGATTTTCCGCGGAACTCCAATGATTGTTCAGTCTATGGTTATCTATTACGGAACAGCCCAGGCTTTTGGAATTTCTATCGACCGGACTATCGCTGCCATTTTCATCGTCTCCATCAATACTGGAGCTTATATGAGCGAGATTGTCCGCGGTGGTATCTTTGCCGTTGATAAGGGGCAATTCGAGGCCGCAACAGCACTGGGTATGACCCATGGCCAGACCATGCGCAAGATTGTTCTGCCTCAGGTCGTCCGCAATATTCTGCCCGCAACTGGTAACGAATTTGTCATCAACATCAAGGATACATCCGTGTTGAACGTTATCTCGGTAGTTGAGCTTTATTTCTCTGGAAATACTATCGCTACCCAGACCTACCAATACTTCCAAACCTTTACCATTATCGCTGTAATCTACTTTGTCCTGACATTCAGCGTAACACGCATCCTGCGCTATGTTGAAAAACGCTTCGACACAGACAACTACACGACAGGTGCCAATCAAATGCAGACAGGAGAAGTGAAATCATGACAGAAACCATTTTAGAAATTAAAAACCTCAAAAAGTCCTACGGTGAAAACCAAGTCCTCAAGGACATCTCCCTCACCGTTCATAAAGGAGAAGTAATTTCCATCATCGGCAGCTCTGGCAGTGGAAAGTCAACCTTCCTGCGATCCATTAACCTTCTGGAAACACCAACTGGCGGACAGATTTTCTATCGCGGCAAGAATGTCCTGGACGAGAATTACGACTTGACCCATTACCGTGAAAAGCTGGGCATGGTCTTTCAGTCTTTCAATCTCTTCGAGAATCTTAATGTCCTAGAAAATACCATCGTCGCTCAGACAACTGTCCTGAAAAAAGAACGGTCAGAAGCCGAGAAAATTGCCAAAGAAAACCTCAACAAGGTTGGCATGGGCGAGCAATACTGGCAGGCCAAGCCTAAGCAACTCTCAGGCGGTCAGAAGCAGCGGGTCGCTATTGCCCGCGCCCTCTCCATGAATCCTGACGCCATCCTCTTTGATGAGCCAACTTCAGCCCTCGACCCTGAAATGGTTGGTGAAGTTCTGAAAATCATGAAAGAACTGGCTCAGGAAGGACTGACTATGATTGTCGTAACCCACGAGATGGAATTTGCCCGTGATGTTTCCAGCCGTGTTATCTTTATGGACAAGGGGGTCATCGCTGAAGCCGGCAGCCCGCAGGATATCTTCACCAATCCAAAAGAAGAAAGAACCAAAGAATTCCTGCAGCGCTTCCTCAGCTAAATTACTTCTATTTTCAAAAGCAAAAAGGAATCTGGAACTAAGTCCAGATTCCTTTTTTGTTTTAATGAGCAATATCAGTTGCACGATTTTGAAGTTTAGTCTTCAAATTTTTCATGGTTTTTGTCGTAGAAATCAATCAAGCCAAGAGCTGTTTCAAAAGAGATATTTTTCACTTTTGCACGTCCTTGAGCAAGAGCAATGATAGACATTTCACGTGCATTTGTTTCTTTACTAATACGGTAACCGGTGATCTTCTTATCACGTACCCAACTAACAACAGATTCTACTTTCTCGAAATTAGATTTAGCCATTTCTTACTCCTCTCTATTCTAGCTACATCAACTACTATAATTGTTTTTATACAGTTTGTCAACTTAAACAAACCAAATTCGAACAAATTAAATAATTCTGAATTTTCTTTACTTTGCTTTGAGAGCTGAAAGTATCTGAGTCCGAATACTTTCGACACCTTCTGGATTTGCCGGAAGGAAGATGGTATTATTACCCGAACTATCTGCAAAATTATTCAGCGTATCCAGGTACTGGTTGGTCAAAAGAATAGACATAATCTGCTCTTCTGTCAACTCAATATTGGCACCCTTCAATTCCTTGATAGAGTCTGCCAAACCATCCACAATGGCCTTCCGCTGCTCAGCGATACCAACCCCATGAAGACGGTCTTTCTCAGCTTCTGCAGAGGCGGCTGTCACAATCTTAATCTTATCCGCTTCAGCCAATTCCTGAGCAGCCACACGCTTACGCTGCGCCGCATTGATTTCGTTCATAGACTGTTTAACTTCAGCGTCTGGCTCAACTTTGGTAATCAAGGTTTTGACAATAATGTAACCATAGGTTGACATTTCTTCTGCAACTTGCTTTTGTACTTCTAGGGCAATTTCGTCCTTCTTTTCAAAGAGCTCATCCAAGGTCAATTTCGGAACGGAGGAACGAAGCGCATCTTCAATATAGGACTTAATCTGGGCTTCTGGCCGCATAAGTTTGTAATAAGCATCAATTACGTTGTTTTCATTTACACGATACTGGGTCGCAACATTCATAGTTACGAAAACATTATCCTGCGTCTTGGTCTCAACGACAATCTCGCTCTGCAGCAAACGCAGCTGGACACGAGCCGCTATCTTGTCAATCCCTAAAGGAAGACGGAAATTGATACCGCTGCTGCTGGTTTTGTGGTAGCGTCCAAAACGCTCAATGATTGCCACAGATTGCTGACGAACCACATAAACTGCACTAAGCAGCAAGAATATAAAGATTATGATCAATATGATAAAGAAAAATGGAATAAAAAACATGTTCAAGCCTCCTTGATTCACTATAGTATATTCTAACATATTTAATTTGCAGTTTCAAACGAAAGCATACAAATTAGGCTAAAATATATTTACAATTAGACAAGCATATTGTAGAGTGTTTCATTCCCGTTCAGATTGATAAAGGACGGATCGAACTGCTCAATACGGTTAATTAAAGAAGCATAATCATGCTTATCTGCCAGAGAAATACCAATCAAAACTGGTCCCGTTCCCTTGCTTGCCCGCTTGATATATTCAAAACGAGTAATATCGTCATTGGGCCCTAAAATATCATTCACAAATTCCCTCAGGGCACCCGGACGCTGCGGGAAGTTAACCACGAAATAGTGCTTGATACCATCATAAATAAGTGCCCGTTCTTCCATCTCTGGCATCCGGTTGATGTCATTATTACCACCTGAGATAATGCAACAGATAGTTTTCCCCTTGATATACTCGCTCAGTACTTCCAAAGCAGCAACACTTGCAGCACCAGCTGGCTCTGCGACAATCCCTTGCTTAGAATATAGGTCAATGATGGTCTCTGAAATAAGACCTTCATCTACACCAATAAGATTTTGAACATTCTTTTGTGTAACCTCATAGGTTGATGCACCGACCTTCTGTACTGCTATGCCGTCCGCAAATTTGTCGATTTCTTTAAGCTTGACCGGACCTCCAGCTTCAAAAGCTGCTTTCATACTGCGGGCACCATTGGCTTCCACACCAATAACTTCGATAGTTGGCTGACTTTCTTTGATATAGGTTGAGACTCCTGATATCAGGCCGCCGCCTCCAACTGGTACCAAAACTGCATCAAAATCGATAGCTTCTCTCTTAGCCTCGTCTAAGATTTCATAAGCCACTGTTCCTTGACCAGCTTGGACATCATCATTGTCAAAAGGATCGATAAAGGTGCGGTTTTCAGCCTTAGTATAGTCAAGCGCAGCCTTGGCCGAAGCATCAAAGGTGTCTCCAACCAGCTTGATATCCACAAATTCACCGCCAAAGAAGCGAACCTGGCCAATCTTCTGCTGAGGGGTTGTTATGGGCATAAAAATAGTTGCTGGAATTTTCATTTCCTTACAAGTATAGGCCACTCCTTGGGCGTGATTTCCCGCAGAAGCACAGACAACCCCACGCTGACGTTCCTCTTCATTAAGTTGAGAAATGGCATAATAAGCCCCGCGGAGTTTAAAAGAGCGTACCCGCTGCATATTTTCCTTCTTCAGGTAAATCTTGGCATGGTACTTCTCAGACAAATAGTGGTCGTAGTCAAGCGGAGTATTGACAACCACATCTTTTAACACTTTATGAGCATGAGTGATATCTTTTGCTCTGAGCATATTTTTTCCTTCCCTATTCTTCCAGTTTATTCTCTATCAAGCAAAACAAGGGGCTGGGAATTCGCATTCCAACCCCTAATGTCTTATCTGCTCTTTATCTTAATTATAAATCTTGAAAGCATCGTCGTCGTTTTTACCAACGAATGGCATTGCTTTACGCAATTCTGCACCGACCTTTTCAATCTCCAGATTAGCAGCTTGCTCGCGGTAAGCAGTGAGTTTTGGACGACCAGCCTTGTAATCATCAACGAAGTCGTTCGCAAATTTACCGTTTTGGATATCAGCCAAGACTGCCTTCATGTTTTCTTTGACTTGCTCAGTAATGACGCGCGGACCAGATACATAGTCACCATATTCAGCTGTATTTGAGATAGATTGGCGCATCTTCTTGAAGCCACCTTCATAGATCAAGTCTACAATCAGTTTCATTTCGTGCAGCACTTCAAAGTAAGCCAATTCTGGCGCATAACCTGCTTCAGTCAGAACTTCAAATCCTGCTTCAATCAGGGCAGTCAAGCCACCACAAAGCACAGCTTGTTCACCAAAGAGATCTTCTTCTGTTTCTTCTTTATAAGTTGTTTCAAGAAGTCCAACACGAGCTGAACCAACACCTTTACACCAGTCCATTGCGATGTCTTTGGCATTTCCAGTAGCGTCTTGGTAGACTGCATACAGAGCTGGTACACCAAAACCTTCTTCGAAAGTACGGCGAACCAAGTGACCAGGGCCTTTAGGAGCACACATAAAGACATCTACATCGGCAGGAACTTTGATAAACTCAAAATGGATGTTGAAACCATGAGCAAACCCAACTGCGTTTCCAGCTTCCAAGTTTGGAGCGATTTCTGCTTCATAGAGATCTTGTTGGATTTCATCTGGAGCCAAAATCATGATGACATCAGCTAATTTAGCTGCTTCTGCTACTGTATAAGTATCAAAGCCATCTTCTTTAGCCTTGTCAAATGACTTACCAGGACGAACACCGATAATCACATCGTGGCCTGTATCACGCAAGTTTTGTGCATGCGCATGTCCTTGTGAACCATAACCGATAACGGCGATTTTTTTACCGTCAAGCGCTGCTACTTTAACATCTTTTTCGTATTCCATTGTTACTGCCATAAGTTTTACTCTCTTTTCTATTTTTATTGCCTATTAGGCGGTTTTAACAAATTAAAGGTTTGATTTAATCTCGGGTAAAGCCTGTCGCTCCAGTCCGAGCAATATTTTTAATACCATATGGGCGAATGACTCGAAGCAGAGCCTCGCTCTTCTCCGCATCACCAGTCATCTGAACGGTAATAGAGCTAGGTGCTACATCCACAACTGTCGCCCGGAATGGCTGGATAATAGACAGAATCTCTGCTCGCTTATCAGCTGGAGCTGAGACCTTAACCAGAATCACTTCACGCTCTAAGTGAGGACGGTCCGTGATATCTCGAACCCGAATCACATCAATCTGACGATTGAGCTGCTTGATAATCTGCTCGACTTCTGCCAATGAAGCCACATCAATGATGATGGTGATACGGGATACTTCCGGATTTTCCGTCGTTCCGACTGAGATACTCTCAATATTAACCTGACGTCTGGAAAGGACACCCGTGAAGCGGTTTAGAACACCCGAACGGTTTTGGAGTTTAGCTGTCAACATTCTACGCATGGAACTTCACCCCCAACATCTCATGATTGCTCTTGCCAGCTGGCACCATTGGAAGAACGTGCTCCTTGCGAGAGATATCTACCTCGATAAACATCGGTATATCTTCCTTCAGCACTTCCAAATCCTGGACAATAGTCTCCGGATCGTCAAATTTATAGGATTTAACACCATAAGCCTGAGCCATGAGCTGGAAATCTGGCAAAGTTTCAAAGACAGACTCAGAGGTACGACCATCATAGAAAGCTTCCTGCCACTGGCGCACCATGCCTAGCGAATGATTGTTAAGCATGATGACCTTAATCGGAATCTTATAGATATTGAGAATAGCCATCTCTTGGTTCGTCATCTGATAGCCACCATCTCCAACAAAGAGCACTACTTCCTTATCCGGATTAGCAATTTTAGCTCCAATCGCTGCAGGTACACCGAAGCCCATAGTTCCTAGACCACCAGACGTTACCAGTTGACGCTCATTTTTATATGGATAATATTGGGCGGCCCACATTTGGTGCTGACCGACGTCTGTTACAACGATGGCATCACCCTTGGTCAGCTCACCCACGCGCTCAATGACAGCCTGAGGCTGTACGACACGTTCTTTCTTATCATATGAGCGAACCCGTTCCTTGTCCTGCGTTACTTTTTCAATCCACTTGCTGGTATTATTATGCACCTGCTCTTCATTTAGCAGCATCTGCAGGGCTTTCTTAGCATCCCCTACGATAGGAATGTCTACACTGATAATTTTCCCAATTTCTGCTGGATCGATATCAATATGTGCTACTTTAGCGTTCTTGGCAAAGGTTTTCGGATTACCAGTCAGACGGTCATCAAAGCGGCAGCCGATACTAATCATAAAGTCGGCCTCAGTCATAGCAATATTGGCCGCAAAAGAACCGTGCATTCCACCCATACCTAAGAACAAAGGATGGTCTGTCGCAATGGTCCCTTGCCCCAACAAGGTAGTGACTACTGGAATCTGATAACGCTCCGCAAAGGCAACCAATTCCGCTGCTGCCCCAGCATAACTGATACCGCCTCCCGAAAGAAGGACCGGCTTTTTAGCCTTAGAAATCTGCTTGATAATCTTCTTAATCTGCAATTCATTAGGCTCAATAGTCGGCTGATAGCTTGGTAAATGCAACTTGCTGTCATAGATAAAGTCCGTTTCCAGAGCGGAGACATCCTTAGGCAAATCAATAACGACTGGACCAGGACGACCTGTCGTCGCAATGTGAATAGCTTCTGTAATGATACGCGGAATTTCTGCCGTCTCCCGAACCTGATAATTGTACTTGGTAATAGGAGTCGTAATCCCAACAATATCCGCTTCCTGAAAAGCATCTTTACCGATCCCGGCCTTGGCTACCTGACCAGTAAAGACTAAAAGGGGGACGCTATCACTCATGGCATCAGCAATGCCGGTAATGGCATTAGTCGCTCCCGGACCGCTGGTCACAACTGCAACTCCGATTTTCCCAGTAGACTTAGCATAGCCTTCTGCTTCGTGAACACAGCCTTGCTCATGGCGGCCTAGAATATGACGGATGCCTTCAAAACTATAAATAGCATCATAAAGCGGCAGTACAGCGCCGCCGGGATAGCCAAAAATCGTATCAACACTAAGATTTTTCAAGGTTTCCAAAACAAGCTCTGAACCTGTCT

>c183_g111

AAAATAGGCACTATTTTTGGCCAAGTCATTGATATTCTGGAAAAGCCTGATTTGAAAAGGCCTCAGGAGCTGAGAGAACTTCCAAAAGGAAGGGATCTGGTTATGACGGATGTCAGCTTTGGCTATACAGATGAAGAGGAAGTACTCCATGGTATTTCGCTAGATATTCAGGCTGGCAGCATCAATGCTTTAGTTGGGCCATCAGGATCAGGTAAGTCTACAATTGCTAAGCTTCTGGCTTCTTTCTGGGATGTCACTTCTGGTCAGATTACCTATGGTGGCTTGGATATTCGTCAGCTTCCGCTAGATTATTACAGTCGGCAGATTGCTTACGTGACTCAGGATAACTATCTCTTTGATGAGACTATTATGGAAAATATCCGGATGGGAAATCCAGCAGCTTCTGATGAAGAGGTCATCGAAATTGCTCGTCGCTGTGGCTGTTATGACTTTATCATGAACTTGGAAGATGGCTTTGAAACTCAGGTAGGCTCTGGTGGCAGTCATCTATCTGGTGGGGAGCGTCAGCGGATTGCCATCGCCCGTGCCATGCTCAAGGATGCGCCGATTCTTATCTTGGATGAAGCGACTGCTTATACAGATCCGGAAAATGAAGCACGGATCCAGTCCAGTCTAGCTCGTCTGATTGAGGGTCGGACCTTGATTGTCATTGCTCACAGGCTGTCTACGATTATGAGTGCAGACCAGATAGTCTTGGTCAATGATGGTCGGATTGAAGCTAGAGGTCGGCATGAAGAATTGCTGACAGCAAGTCCGCTTTATGCTTCCATGTGGCAGGCCCATATTGCTACTAGGGATAGTGATGAGATGGAAGGAGGGTTGACTCATGCTTAATATACTGAAGAAATTCTTTGATTTCTGTACGGCAGAGGACCGTAGGAAATTTTATCAATCCATTTATCTGGGCGTCATCAAGTCCTTTATCATCGCTCTGCGTATCCCAGCGATCGGCTTAGTCGTTATGGGACTGATTGAGAAGAATCTCTCTATGCAGACTTTCTGGCTGGCTCTGGGCATCATGCTGGTATCCACAGTGCTAAACGTCTGGATTACCCTGAAAATCACCATGCTGCAGACG

>c183_g112

ATTTACCAAGCTGGCCTTCGATTTTATTTCGCTTTATCTGACAGTTGTTGGCTTGGTAAATATTGTTCTTCATGTTTTTACGCATAAGAAAGAAGGGGCAGTCTGGCATAGTCTGCTTCAGATTGCAGTCGCTATGGGTATCAGCTGGCTTAACCGAATCAGTGATGTGCCTGTCAATATTGTGATTATCAGTTTGGGTAGCTACCAGCTCTTGACTGCCGGTATTTACGGAGTGACCTATCTCCTTTATCGGCAAAATCATGTCAAGGGCGGCTTGCGCTATCTATTTGATACCGTTCTCTATGGTGGAATTGGGCTGACCAGTATTCTTTCGCCTGCGACTGACGGACACTTGCAGTTTTTGATTTTGGGAATCTATTTGATGATGCTGGGAATGTCCAATATCCGGGATGGCCTATTTTTTGATAATGATCGTGAAAAACATCGGCTGCGTAGGCAAATTCGTATTAACCTACCTATTATTTTTGCAGCCTTTATTCCGGTAGAAAATCTGGAACACTTTAATCGACTGATTCAGGGAGATGCAGCTTCTAATCGTAAAAATGTTTATAATCTTGTTAAGAGTGGAGATAAAAAGTCGGACTTAGAGGTC

>c183_g113

ATAGTCTTGAAATTAACCGCCGAACAATCCTTTAAAGAAGTTGACAATGGCATTCCAGATATTAGAAAAGAAATTACCGCCTTCTTGAAGGAGGCCGTTTGCATCAAAGTTGAGGTTAATATTGTTAAAGGTATCCCCTGCCTTTGAAACGATGCTGTCTTTCAAGTCGCTAAGCGTCTTTGTAAAGTCAGAATCTGTAATGACTCCACTGTTTGAAAGATTGATAGCGAAATTCACGATGAGGTTGATTTGGTCACTGGTCACTGACTGAGTCAGACCGTAGTTTTTCAAGGTTTCTTCAACAATCTTACGGACATCATCTGCTGTCAGATTGCTGTTGTTCTTCTTAGCATTGGCGATGGCTGCTTTGATATCAGCCAGAGCTACGTTGAGCTTGTCTGCGTCATAGCCTTCCTTACCTGTGTTTTCAGCATTGATGCCAGAAAGGGCAGCCAGTTCTTCCTGCGCTAGGTTTTTATTTTCCTGTGGAATGGTCGCACCGTTATCTTCTAGAGAGTAGTAAATCCCTGCCAAGGCACTCTCACCTGTTACCGGAATCGGTGCAGCAACGGTAATCTGCGCGTGCTGAACACCCAGTGTTACAGCCGCATTGCGATACATATCTTCTGTAACCTTAGTGATGTTTTGCGGAGTGACAATTTTGACCTCTAATGGCTTATTGCTGCCTAACTTCTGGATTTTTACAGAAGAATAAAGCTGCAGGCTAGGGTCGTTAGCAACATCCATGATTTTAGAGTAGACTTCTGGAGTCATGGTCTTAAGTGGCTTGTTGTCACTAGATGAATTGTATCCCAACATCTGCAAGGTCTGCTGTTTTTGGCTTTCATCTAAGGAATAACCCAGCACATACTCAGGCTGCACGTAAGTCTCATCAATGACTTTCTGAACATTTGAATCGGCAGCTGCGGTCGATACAGTAAACAGAGTAGCCAGCAAGGCTCCTGCTGTCATTAAAGTTTTTTTGAATTTCATATTTTTTCCTCTTTTCCTCCCGCTATAGTTTCCCAAATTCTATAGCTTTTTTCGTAGCAAACTTTCCATCTGATTGCCTCGAATTTCTGTTTCTTGAGCGTGTGAGTTCTGCTCCATTTGGTTTTCAGAGTGCTTCAGTTGAGTTGCTGCAATGATGGAGTTATTATTATCTCATCCATTGAGGGCTTGAGATAGTAGATACATTTTACAACATTTCTCAAAGAAGTTCAAAGAAAAAACAGTCACTGATTTTGCTTGCCAGTAACTGATCTTTTCTACTTTTTCCAAAGGAAATCAATTAATAACTCGTCTACTTCTGGATTCTCATGCAATTGGCTGTGCTGAGCCAACTGACCTGTGATTTTCTTTTCCTGGTAAGATTTAGCATTCTCAACTAGGAGATATTTGAGGCTGCGGGAAGAGGTATTGAGTACGGAGCCATCGGATTCGTTTTTAAAATCACCATAAATATTCAGAACATCGACTTGCTCTTGTGGATATACTTCTCGTAATCCCAGTAGTTTTTGATAAGTCTGGCTCATAGCACTAGGCTGGCCAGTTTGGTCGTCTAGGATTCTCAAATCTTCAGGCAAGTCCATTCCTTGCAGACCATTAACATGGTTGGCAATATTGACCTGCTTTTGCAGCTCGGGCAGCTTTTCATCCTGGCCGTTCTCCAGCATATAAAAGAGGATGGACATATTTCCCATCGAATGGCCTACCAGATTCATCTTATCAAAGCCATAAGTTTCTTGTAGCTTGCGAATCACAGCTGCTGCATACTTACCATCTTGAGCGTAGTCGGCATTGCGGTTATCTTCATAGTTTACCTTGATAATAGGATTGATAGCACCTTTCGGAATCTCTCCTAATAAGCTTACTTGACCGTTTTTACTGACATTTGCTGTGATAATTGTCTGAGTGACTCCAGCCTTTTTGGCTGCCTCTGTCATATGATTTTCAGCATTAGAGCTGGATCCAAAACCGTGGAAAAACAAGGTCGGTGTTGTTGACTGAACATATTGTTTTGTATTGAGTCCACCAGCTTCTTTATGGTTGTAAAAAGCAGTTAGACCGGCAATTGTCACTAAAACCAGAATAAGGAGCTTAAAACCAAATTTTTTGTTCATTTTTCATTCCTTTTAATGATAGATGTACAAAATTATATCATGTATCCATCGTTGTTACAAATATCTGGATTATTACTTCACCATCAAAAAAGAAAGAAGCAATCAAACTTCTTTCTAAAACCATTTTAAGAGGCGCATGATGTGCATGTTCATCTTGACCTGACGGGAATAATAATAATTTCCGCCGTTGATGTAGAGCTCTGGACCGTGGAAAATTGAAATCAGGTTATAGTAGCCGTAGGTCTTGCCAGTTACATTTCCTAGACTTGGCGCTACTACATCTTTTGAATATTTCTTGGCCAGCTCCAGCGTATTTTCGCCACCGTTTTTAGCGACGTAGTCAATATAGGCCTGACCGAAATTATAGGCTTGTACAGCTGTCCAAACATCCACCTTTTTCTCGCTGGCCAACTCTAGGTTATCAGACAAGGTCTGAACGCCCTGGCGAATACTTTCTTTGTTGTCCGTAATGGCATTAGTCTGACCAGTCGCGCTTTCACTGGACTGCATGACATCAGTGTCTTTTCCCTTTGTCTCTGTATAAATCATGGCCAGAACCAATTCTTCATTGGCCGCTGTGTCCTGCTCGTCCAGCACTTCTCTGACTAAGCTCTGATACTTCATGACTTGTTTGACATCATGATGAACCTGATAAATTTTATAGCCGGCAAATAGCACGAAAAGGACTAAAATCAGTCTTCTTAGAAATTTAAACATTATTTATTTTGGATATCCTCGATGTTCTTAATCAAAATAGAATAGGTTCCGTTGTCGTTCTGGATAAATTCTACAGACTCTGCGTCCTGATAGACATTATTAGGAACGATGAGCTCAATTCCATTTGAAAGGGAGAGTTTCTGATTTTCGAACTTTTTCTTCTGGCGGCTACTATCAATCTCATCAAATTTGACTGGCTCAGGAATAGCTTCCTTGACTTGGTCAATAAAGGTAAGTCGGGCAGTTAGATTGCTGTCAAAAAGATCATCGGCCAGTTTCTCAGGCGACAATTCGTCGTTTTCTTCCAGATTTTTGAAAATACTGGATTTAACCTTAGACTGAAACTGGAAATCGTCTTTGTTAAAACTTTCGGCAACTTTTTGAGCAGTCTTCTCAAGCGCTTTGATTGATTTCTTGGCTGAGATTGTCGGATTGACCTGCAAGAGATTGTCCGACATATAATTGAGAAAGGCACCGTTATACTTGATGCGTTTTTCAATCAGATGGTACTTGCGAGACTTGAGATTGATGACCAAAGCCTCGTCTGCACCTGTACCAAATCCAGGTAAATTGTTCTGCGTCAGCTTGATAGGATTGTCCACCTCACCACCCAAGTGGGTCAGAGTTTCCCGCAGAGCAATCCGCAGAAAAGCGAAGTGTTCCACACCTTCTTTGTCAAACTGGACAAAAATCAAATCATTGGTTTTCTGGTTTTCACTGACAGAAAACTCCTCCTGCCAGAGCTTGGCCACTGTCACTGATGTTTCTAGTAGGTCGTCTGAAAGATGGGCTAAAAAGACGTTTTCGGGATCGAAAACACCTGTTTTAGCATCGTCAGAATAGACCCGCTCAATCTTCTTGCGCAGGTATTCTTCAATCTTAGGAGTAATGTTGAGAAACTTATCCGCCAGCAGCAGTTCGGTATCAGCTGGGCTGAATTGATGAATAATCGCTTTTTTTACGTAAATATCCATCTTAGTTCAAGCCCTCATAGAGTGGAAAAGCGTCTGTCAATTCACGAACTTCCGCCCGCACCTGATTCAGCACCGCTTCATTTTCAGCGTTTTCCAGAGCCTTGATAATAAGCTCTGCCACCTTGATGCTTTCTGTTACACCAAAGCCTCGAGCTGCAATAGCTGCAGTACCGATACGAATTCCGCTAGTCTTAAATGGTGACAAAGTCTCGTAAGGGATGGAGTTTTTATTGAGGGTAATATTAACCTCATCCAAGAGATTTTGCGCTACTTTCCCATTTTCTACAACCTTAGTAACATCCACCAAGAAAAGGTGATTTTCAGTACCGTCAGAAATCACACGGAACTTGTCATGCTGACGGAAGACTTGCGCCATAGCTTGGGCATTATCCAAAATCTGCTGTGCATAAACCTTGAAAGCTGGATCCAGCACTTCTTTAAAGGCAACTGCTTTGGCTGCAACGACATGCTCCAATGGTCCACCCTGAATGCCAGGAAAGATAGCTGAGTTAATTTTCTTAGCCAATTCTTCATCATTTGTCAAAATCAATCCACCGCGAGGTCCGCGTAAGGTCTTGTGGGTTGTAGTTGTTGTGATATCCGCATAAGGAACAGGGCTAGGATGGAGACCTGCAGCAACCAAGCCAGCGATATGAGCCATATCTACCATAAGCTTGGCACCTACAGCATCGGCTATTTCGCGGAATTTTGAAAAATCAATGATATGAGAGTAGGCTGAAGCCCCCGCCACAATCAGCTTAGGCTGTACTTCTTTGGCTTGCTGGAGGATAGCATCAAAGTCCAGCAATTCTGTTTCTGGATCAACGCTATAAGATACAAAATTGTAGGTTTGCCCAGAGAAGCTGACTGATGCACCGTGTGTCAAGTGACCACCTGCAGACAAATCCATACCCATAACTGTATCACCTGGCTCAATCAAAGCCATATAAGCTGCACAATTAGCCTGGCTGCCTGAGTGAGGCTGAACATTAGCAAACTTGGCACCAAAGATTTCCTTGGCGCGTTCAATTGCCAGACCCTCAATCACATCCACTACATCTGTCCCACCATAGTAACGGCGACCTGGGTAACCTTCAGCGTACTTATTGGTCAAAATAGAACCTTGAGCGGCCATGACAGCTTTAGAAACGACATTTTCTGAAGCAATCAGCTCAATATTATGCTGCTGACGTTCTTCTTCTTTAGCAACTGCTTCCCAAATCTCTGGGTCAAATGCTTTGTAGTCTTCTTGGTCAAAAATCATAAGGTGTCTCCCTTCTTTTGCGAAAATAGGACCTTAGGTCCTAAAGTTAAAAATTTATATCATTTACTTGGGCGAGAATATACTCGCGCGTGATTGCCCCTTGGCGTAAAATGAGTGCCTTTTCTCCAGACAAGTCCAGAATAGTTGAATCCTGACCTGTCAAAGATGCATCATCTTCTACACCTGAGATCTCTTCTTGAAAATCCATCATAATCTGCTGGAAAGAAGTTCCACTAGCTTTTCCAGAAAGATTGGCGGATGGACCAATCAAGGGACCGTACTTGCGAATCAAGTCAAGCGTAACCGGATGCTTAGGAATCCGAAAGCCCACAGTTTCCATACCTGAGTTGATCCAAGTCGGCACTCGGTCATTGGCTTGGAGGATAATAGTCAGCGGCCCAGGCAGAAAGGCTTGATAGAGTTGCTTCAGATAACTGGGCTGGTTCTTAGAAAAAGCATAAATCTCTTCTATGCTAGCCACATTGAGATTAAGAGCCTTGTCACGAGGTCTTCGTTTTAATTCGTAGACTCGCTCAACTGCCTCTTCATTTAAAGCCTGAGCAAAGAGGCCATAAACAGTTTCTGTCGGCAGGACAACCGCTCCACCTGCTACCAGAGTCTTTTCTATCTTATCCATTGTCCATCGCCACCATTCTATCTTTGCCAAATTGATCTTTCAGGACTCGGATTCGTTTTTGAGGGAAGTTTTTTTCCAGCAACTCCCTGATGCCATCTCCTTGCTTGTAACCAATTTCCAGATAGATTTTTCCTTTTTTTGTCAGATAGTCTCCTGCTTGCTCGGCTATTTTCCGATAGACGGCATAGCCATCTTCCTCAGCAAAGAGGGCCATGTGAGGCTCTGAGGTCAAAACATTGAGTCCAACTTCGTCTTTATCTGCTTCTGAAATATAGGGCGGGTTGGAGACGATAATGTCAAATTTTCCTTGAATCGAATCCAAGCAATCGGATTGGACAAAAGCAAGGTTAAGTTCGCAGGACTGAGCGTTTTCTGCTGCCAAGGCAAGAGCATCATCCGAGAGATCAGAAGCAGTAATCTGCCAGTTTGGACGGCTGTTTGCCAGTGCCAGAGCAATGGCTCCGCTACCCGTTCCGATATCCAAGACAGACAGAGAACTTTCGGGATTTTCTGACAAAATCAGCTCCACTAGCTCCTCCGTCTCAGGCCTTGGAATCAGAACCCGCTCATCCACCTTGAGAGTCAGGCCGTGAAAGTCACTGCTGCCAATGATATACTGGGCTGGCTTGTGAACAAGCAGCTGCTCTTGAATAGCTTTTAGCTGGTCACGGTCTTTTTGACTTACTTCTGCCCTAAGTTTGAGAACAAAGTCGGTAAAGGAGAGCTTATTAAGAGCCCTATAAACGAAAGACAGGCTTTCTGCTTCTTCTCCAGCTGCTACTAGCTCTTGCTCTAGCTGCAGCAAATATTGAGCTAATGTCATTATTTATTCAGCTCTTCTAGTTTTTGCGTTTGGTCATAGAGAACCAGAGCGTCAACGACTTCATCCAGTTTGCCAGCTAAAATCGTGTCCAGCTTTTGCAAGGTCAAGCCGATACGGTGATCTGTCACTCGATTTTGCGGGAAATTATAGGTGCGAATCCGCTCTGAGCGATCACCTGTACCAATCGTAGACTTGCGCTCTGCATCTTGCTCATCTTGAGCGATTTGAGCAAAGTGGTCTGCTACACGAGCTCGGATGATTTTCATAGCCTTATCCCGGTTCTTCTGCTGGGTCCGCTCTTCCTGCATTTCGACCTTGATATTGGTTGGTAGATGGACAATACGAACCGCGGTCGCAACCTTATTGACGTTCTGTCCGCCAGCACCTGAAGCATGGTAGATGTCCACTCGCAGATCTTTAGGATCAATGTCGTATTCGACTTCTTCGATTTCTGGCATCACCAAGACAGTTGCTGTTGAGGTGTGAACGCGACCTTGGCTCTCGGTTACAGGAACCCGTTGTACGCGGTGAGCACCAGATTCATACTTAAGCTTTGAGTAGACCGACTGGCCTGAGACCATAGCGACCACTTCCTTTATCCCACCTACACCATTATAAGAAGCCTCCATGACTTCGAAGCGCCAGCCTTGGCTTTCAGCATATTTTTGATACATCTGCAAGAGGTCTCCTGCAAACAACTGCGCTTCGTCTCCTCCTGCAGCCCCGCGGATTTCAAGGATAATGTTCTTATCATCGTTTGGATCCTTAGGTAAAAGAAGGATTTTTAGTTTTTCCTCGTATTCTTCTTTTTCAGCCTTAGCATCCTTGAGCTCCTGCTTGGCCATTTCTTCTAAGTCAGCATCACCAGAGGCGTCTTTTATCATTTCTTCAGCGTCTGTGATGTTTTGCAGGACTTTTTTATACTCGCGGTATGCCGTCACTGTATCTCGGGTGCTGGCTTCTTCCTTAGAAAGGTCCATAAAACGCTTGGTATCGCTGACTACATCAGGGTCGCTTAGGAGTTCACCAAGCTCTTCGTAGCGATCTTCAACCGCTTGTAACTGTTCATAGATGTTCATAGATTTTTCATCATCTCCTTTTTTTCTATCGTTGGATTACTTAATATCGGGGTTAAAATAGTGCTTGCGGCAGACTGAGATATAGGTCTCGTGGCCGCCAATTTTAATCTGCTCTCCGTCATAAACCGGCTTTCCGTTATCAGTCCGTAAAACCATGGTGGCCTTACGAGAGCAATATTGGCAAATGGTTTTAATCTCCTCAATCTTGTCAGCCAAGAGCAAGAGGTGCTTGGAACCCTCAAAAAGTTCATTGCGAAAGTCATTTTTCAGACCAAAAGCCATAACAGGAACATCCAGCTCATCCACCACTCTAGCCAAATCATAGACATGATGGCGTTTCAAGAACTGAGCCTCATCGATCAAAACGCAATAAGGTTTTTCAGGCAACTGCTGGATATAGCCAAAAATATCCGTCTGGTCTTCAATAGCTATAGCATTCCGCTTCATGCCGATGCGACTAGAAACAACACCAAAGCCGTCGCGGGTGTCAACAGCCGAGGTCATGATAACCACGCTCTTGCCTTGTTCTTCATAATTGTGGGCCACTTTCAAAATTTCAATAGTCTTACCCGAATTCATCGTAC

>c183_g114

ATATGTCTGGTCACAGTCAGTCTGGTCCTGGGGTGGCTTATACCGCTTCAAAATATGGACTTCTAGAGAATAGAGCAATCAAAGTTACGAACTTTATGGACTTTGAAGCCCATGATGCGGTAGCGAGTGGTGCAATCTCAAAAGAACAGGTAAACTATCTTAACAAAAACGCTACTATCTATAGAGATAGTCGAGGTGATGTCGTTTTCTTAGATGGCAATAATGGCGATGTTCCCTATGGAAAAACAATGAGATTTGAAGTTCTTGAAGGAGAAGACGGACATTCACCAAGAACTCCAGTAATGAAGGGGAACAAGTTAGATATAGATTATTATTATAAAAGAAATCTATTTGCAACTGGCATGACAGAAAAACAGGTCCGAAAGATTGCTGAATATAAAGCAAAAACGTACAAAGTAAATGTAGCAATCGCAAATTATGGATTAGAGGATGACAAGCAAAATCCTGAGTATTATGTTAGTGAGTATTTAAAGGAATATGGAGACTTTGCTCCTGAGCCTTCTAAACAGGATTTAATCGCCATAAATAGACAACACATTGATGAACTTCATGCTTCTTTAAGAACCAGCTCTGGCGACAAAAAAATTAGTTTAAGAGAGGAACTTGTTCGTACAAGTGCGCAAACAGCTCAGCTTCAGGCTGAAGTATATGAGCAAGAAATTAAAGATAAGATAAAAAGTGCTAAATCTAGTGTTGAAAATCATATCAAAGAACTGAGAGATGCAGCTTATACTCTAGCCCACAATCTTTCTGCCGATGAGATAGAAGGATTACTATCTGAGTTGAGCATATCAACTGCTTGGAATGATGGAAAAGAAGCAACTACCCTTGCCTCAGCAAGTGCATATACGACTAAAATGACGGAAATCGCTGGTAATTTAAACAAGGCCGCTGATAATATCGTAGCGATTGATCAAAAAGGCGCCCAGATTTTCACAAAAAAATAGGAGGAAACGCATGATTGAACCAAATACAGAAGATCGAGCTGAAGCCGAACGCATAAAAAAAGAATATTTAAAGATTCAGGAACGAATTGCCATTCGCGGCTTGATTTCAGCTAAAAGAGCTGTCCTACTTGAAGAAAGTCAAGCATTGCAGAGTTGGCTAGATAGTCAAGCTGAGGCTATGAAAACTTTTGCCTCTACTCAAGTACCTGCTGACTTATCTGCGGCATTCACAGGTGGAGCAGCAGATTCAATCAAGGAAGTACTAGGCGCTGTTCCTAAGCCTAGTTTGACCAGCCCTATCTTATAGGAGGCTTTATGGAAATTCAAAAAAAGATTGATCGTCTTGATGATGATCACATAGCCTTTCGTAAGAAAGTTAGTGAGTATGAGTGGGATTACCAAGATATGAGACGAGAGGCTAAAAATGTTTCAGAGCAAATGAGCGAATGGATATTATCTTTTTGTCGTAATAGTCCTGATACCGTGCCCTCATATGAATTAAGACAAATAGAAGAAAATAGAGAAATTTTTGAGAGAAAAATTCAGCGTTATGAAGAACGGCTAAACAAAACTTATCACGAAGAAAATCGAATCTATAATAAAAAATTAGAGGAACTTGAGAAAGAAAAGAAGAACTCTTGACCTTTCTTCCATTTTCATAAACCTCATGGACAGGTACTACATCTGTCTTTTTGTTTACATTTATTCTGCAACGAAATCTTTTAAATATGATACAATAATAACAGATTATAAACGGACAAGGGCGGCAGAGTAATCTTGCCATGTTGTTCGCTAGATGAGGAGAAGCTGATGCAAGCAGTTAAACATTTTATTGAAACTTTTGTTCCTGAGCACTATGATCTCTTTTTGGATCTGAATCGTGCTGACAAGACTTTTTCAGGCAAGGTGACCATTACTGGAGAAGCTAAGACGAGTAAGATTTCCCTACATCAAAAAGATTTGACAGTTGAAGCTGTAGAGGTGGCTGGCCAAGCTCGTCCTTTCGCTCTGGATAAGGACAATGAAGCTCTTTATATTGAGCTGGAAGCAGCTGGTCCTGTAGTTGTGACAATAACTTATTCTGGTAAGATTACGGACAATATGACGGGGATTTACCCATCTTACTACACGGTAGATGGTGTGAAAAAAGAGATTATTTCTACGCAGTTTGAGAGCCATTTTGCCCGCGAAGCCTTTCCGAGTGTAGATGAGCCAGAGGCTAAGGCAACTTTTGATTTAGCTTTGAAATTTGACCAAGCGGAGGGTGAGTTGGCCCTGTCTAATATGCCGGAAATTGATGTTGAAAACCGCAAAACAACTGGCGTTTGGAAGTTTGAAACGACTCCACGTATGTCTTCCTATCTCTTGGCTTTTGCTGCTGGTGATATGCAAGGAATTACTGCTAAAACTAAGAATGGAACCTTGGTAGGAGTCTATGCGACCAAGGCCCACCCAGCCAGCAATCTAGAGTTTGCTTTGGACATTGCGGTTCGCTGTATTGAATTTTACGAAGAATACTATGGAGTCAAGTATCCTATTCCTCAGTCCCTTCATGTGGCCCTGCCAGACTTTTCTGCTGGGGCTATGGAAAACTGGGGCTTGGTGACCTATCGGGAAATTTATCTACTGGTGGATGAAAATTCAACAGCCCTGAGCCGCCAGACAGTTGCCTTGGTTGTTGCACACGAACTAGCCCACCAATGGTTCGGAAATCTAGTGACCATGAAGTGGTGGGACGATCTTTGGCTCAATGAAAGCTTTGCCAATATGATGGAGTATGTTTCAGTTGATGCAATTGAGCCTAGCTGGAAGATTTTTGAAGATTTCCAGACCAGCGGAGCTCCTTATGCCCTCAAGCGTGACGCGACTGATGGCGTTCAGTCGGTCCATGTAGAGGTCAAGCACCCAGATGAAATCAATACTCTTTTTGATGGAGCGATTGTTTATGCTAAGGGTAGCCGTCTCATGCACATGCTGCGCCGTTGGCTGGGAGACGATGCTTTCCGTAAGGGACTAGGCGCCTACTTTGAGAAGCACCAGTATGGAAATACCATCGGTCGTGATCTTTGGGATGCTCTTTCTCAAGCTTCTGGCCGTGATGTAGCTGCCTTTATGGATGCTTGGTTGGAACAACCGGGTTATCCAGTTGTCACTGCTAAGGTTGAAAATGACTGCTTGATTTTGACCCAGAAGCAGTTCTTCATTGGAGAGCACGAAGACAAAGGCCGCCTCTGGCCAGTACCGCTCAACAGTAACTGGCAAGGACTTCCTGATACTCTGACGACAGAAAGACTGGAAATTCCTAATTATGCAGCCCTGGCAGCGCAAAATGAAGGAGCCCTGCGTCTCAATACAGAAAACACTGCTCACTATATTACAGATTATCAGGGTGAGTTGCTGGATGCTCTTCTGAATAATCTCAGCTCTTTGGACAATATCAGTAAACTGCAAATCGTGCAGGAGCGCAGACTTCTGGCAGAGTCAGGTAAGATTTCCTATGCGGACCTGCTGCCAGTTATCAGCAAGCTGGCAAACGAAACTTCTTATATGGTGGTTTCAGCTGTTTCCCAAGTGCTAGAAGGATTGAACCGCTTTGTGGATGAGGGTAGTCAGACTGAAGAAGATTACAAAGCACTGCTGAAAATTCTCAGTCAAAGTAATTTCGACCGTCTTGGTTTTGAAAAACAGGAAGGTGAAAGCGATGAGGATGAGATGGTTCGTCAGCTAATTGTGGGCAATATGATTAAGGCTGACGATGAAACGGCTAAAGCTCAAGCTAGCCAGATTTTTGATAGTTATCGTGATAATTTGGAAAAACTCCCTGCCGCTATTCGTTTGCAGGTATTGGTCAACCAAATCAAGCATCATGAAAGCAAGGAGCTGACTAAGCTTTATCTGGACTTATATGTCGCTTCAAATGATGGAAGCTTCAAAAATGCTCTTTCTACAGCACTTTCTTATACTAAGAACAAGGAAACCTTGGATGAACTGTTAGCGACTTGGAAGGATAAATTTACTGTGAAACCGCAGGATTTGTCAGCTTGGTATGCCCGTTTCCTCAGCCGAGATTTTACTCAAGAAGCAGTGTGGACTTGGGCGCGTGAGAATTGGGACTGGATCAAGGCAGCACTAGGTGGCGATATGAGCTTTGATAGCTTTGTCATTAGCCCGGCAGTGGTCTTCAAGACAGAAGAGAGATTGGCAGAGTACAAAGCCTTCTTTGAACCGCAGTTGGACGATATGGCGATTAGCCGAAACATCAGCATGGGAATCAAAGAAATCGCTGCGCGTGTTGAACTGGTGAAGCGTGAGAAAGAAGCAGTAGAAAAAGCAATTCGAGCTGCTATC

>c183_g115

ACCACCGAAGGCAAACACTTTAGGTCCTTCAATCTTGGCTGCTATCTCAACAGCCGCCATAGCTCTGGTAATCTGCTTTTCCTTGGCCAGCGCAACCACTTCTTCATCGCTAATCAAACAGCGATAGCTCACACCCAGCTGGTCCAGCACCCGCTTGTTAATCCCATTGAGAGAGATATTGGAGTCCAGCAGGATAGTCCCCTTAGCTCTGATTACTTCTTCTATTTTTTTAAGGACATCGTGCTCAAAGCGCATGGTATAGAGATAATCAAAATCTCCCGTCGTATGGATTGCCCTCTTGATGATGGATTCTTCCATTTCCGAATGGAAGACATAATCAGGATCTTTTTCAGTAATCATAGACTGAATAATTTGAAAGCTCTTTTCCTCAATAGAGGAAGGATTTTGGATATATGTCATAGTATACTCCTAGAAATATGCTTGCATTACTAAATAAAAAAGAGTGAACAAAATTAAACCTGTCATTGTGCTTGTATAAAGCAGAGAGATGGCTGTCTGAATATCATCCGCCTCAACCGGTCTGGAATCCTCTCCAATGGTCGGCTTCTCAATCAGCTCTCCGTGATAGACGTGCGGTCCGCCCAGCTGAATGCCCAAAGCCCCAGCCACAACTGCCTCCGAAAAAGCACTGTTGGGACTGGCATGCTGATAGCGATCCCGCCAGCCGATCCGCAGGGCTCCTTTGACATCCAGCAGTAAAATCTGACTGCTGAGAATCAAGAACAGCCAAGTCAAACGCGCAGGAATCAGATTAGCCAGATCATCCATCTTGGCGGATATCAAGCCGATTTTGCGGTATTTTTCCGTCTTATAGCCTACCATGGAGTCCAAGGTATTGATGGCCTTATAGGTCATGGCTAGAATGGGGCCACCTAGAAAAAGGCAGAGCAGAGGGCCGATAACCCCGTCACTAGTATTTTCAGCCACCGTCTCAATCGTCGCCTTGCTAATTTCCTCTGGTGTCAGCTGGGAAGTTTCACGGCCGACAATCATACCAACTTGCTTTCTAGCTTCTTCCAAAGTGCCAAACTTGAGCGTATGGTAAACCTTTTGGGCCTCAAAAGCTAGACTCTTAGCCGCCAGACTAGCATATGCCAGATAGATCCAGACGAGCCAGTAGAGGACGGGATGAGCCAGACCAGCCAACCAAAGCAGCCCGCAGCTCACTCCCAGCGCCAGTCCAACTGTTGTCAGCCAGAGGAAAAAGCCAAATAGATAGGGCGAAAACTGCTTTTTCTCCTGCAAGCGCATGCATAGATAAATATAGGAACCCATCCACTTGACTGGATGAGGCCAGCTATAGGGGTCGCCAATCAGCCAGTCCAGTAAGACGGCCAAAAAAATTGCAATGAGTGTCATATCAGTTCGCCTCTTTGATATAGTCCAGCCAGTTGGCTAGCAGCTGCTCATCCTGATAAAAATGAACATGCAGGTAGCTAGCAAAGGTCCGGCCCTTTTGATAACCGCCTGTCCAGGCAGCTACGACCTGACCGTCTCTGACCTTCTCTAGCTTGAGGACCGTATCTTCCTCCGTCTCAAAGACTGAATGGTGAAACTCATGTCCTCTGACCGCCGTACCTTTAGGACCAAAAAGACTGTCTACCTGCGTTTCTGCTTGGCAGTAGCCAAAGCTTTTCAGGCCGGGGGTCATCAGGCTCTTGCCCTTAAAAATACCGACCATTTCATAGACCTGACCCTCTACTTCCAGCAGCTCTCCCAGATACATGAGGCCACCACATTCTGCATAGATTGGCCGGCCCTGCTCGTGAGCTTTCTTAACCGAAGCCCGAAAATCAGCATTTGCCATCAGCTCCTGAGCATAGACTTCTGGAAAACCGCCGCCAAAATAATAAGCATCTGCAGCTGGCAGTTCCTTATCTTTCAGGGGGCTGAAAGGCACTAGCTGGACATTGAGCTCTCTTAGAAAGTCTAGATTGTCTTCGTAGTAAAAGTGAAAAGCATCATCCAAAGCATAAGCCAAAGTCAAGGGCTGGTCATTGCTGATACGAAAAGGATTTGTCATCCGCTTATCGGGAAGTTCTGCCTTTTCCAGCAGTCTGTCTAGGTTAATATGTTTGGCTGTTGCTGCTCCCAAGTCCTCAAATCGACGGTCCAAATCATCCATTTCTACGTCAGGGATTAGACCCAGGTGACGCGATGGCAGCTCTGCTGTTGCATTCTTAGGCAGATAGCCCAGCACTTCCACATCTGTGTAGCGCTCAATGGCGCCCTTGATGAGTTCAAAGTGAGTCTGCGAAGCCACCCGATTGATAATGACCCCAGCAATATCCAAATCCGGATCAAAAGTCGCAAAGCCATGGACCATAGCAGCTGCTGAGGTGGATGTTGCCTTCCCATCAATAATCAAGACAACCGGAATGCCCAACTTTTTAGCAACAGAAGCAGAGGACGCACAGTCCTTGTCTGTTCCCAGACCATCAAAGAGCCCCATGACTCCTTCGACCACTGCCACATCAGCATCTCCATGCCATTTGTAGTAGGACCAGGCCAGGCTCTGATCGTCCGGTATCATAAAGCTGTCCACATTCCGCGACGGCCGCTTGGTAATCCGACTATGATAAGCTGTATCAATATAATCAGGTCCTATCTTATAAGGCTGGACCTGATAACCTCTGTCTGCCAGAGCTTTCAAAATCCCTAGGGTAACTGTAGTCTTTCCAACACCGCTGGAAACACCAGCCAGCATAAATTGCTTCATCGCTTTACTCCAGATTCACTGATAAAAAAAGCACTACAAAAAAGAAGTTTCATTTTTTGTAGCACCGTTGCTCACTTATATG

>c183_g116

TTAGATTTTTTGTGTTTAACTTTTGGGATTAGTGCAGAACATTCTCACCAGTTTTTATTATGCATTTGTCCGAACGTAAACTGCAATCACATCCGCTGTGTACTGGGCAGTGCCTGGTCCAAATTGATCGATATTTTTCTTGAATTCTGGATTGTGGACATAGCCCTGACCGATGTGGGCAAAGACTTGCAGTGAGCAGTCAAAACCATAAGTGCGAATGGCTTGTAAGAGACGCGCTGCCTGCTCTTGATTTTCTGCTGCTTCAATCGGCAAGCCTTGCTGCATATTCTCTGCCAGACTTTGAAAAACTTGATTGAAGGCTGCGGCAGATTCTTCTTCCCGTCCATTTTGACGAACCAATGCTTCCGACATGACTTCTTGACCGTATTCTTCGACGGCTTCTTGATGATATTTGTGATTGTCTTCATAGGTAAAACCTGCGAATTTTTCTTTCATTGTCATTTTTCTTTCTCCCTTTTCATCTTGGATGGTTTTTTGCAAGGTGGAAATCAAGGTATCCAAGCGGTCTCTTTCCTGCTGCAAATACTCTAACTGCCTAACCAGATGTGGCAACAAAGCCTGTTCATCCTGACTCAGCAACTCTGCTATTTTCTCCAAAGAAAAACCCAGATACTTATAGTAAAGGATGACCTGCAGCCTCTCTAAATCAGCTTGGCTGTATGTCCGATAGCCATTTTCTGATTTGGCCGGGACTAACAGTCCGATTTTATCATAATGATGCAGGGTTTTGACAGAGACTCCTGAAAGCTGCGCAGCTTCTTTGATATGGTACATGATAGACCTCCTTACCTAACTAGTATATACCATGACCTAAGGGGAGGGTCAAGTTTTTTAGAAATTTTTAAAATTTCCTTACAGTTTCCAAAGCGACTAGAAGTTATTTACGAGAAACCATGGCAATAAAGCCCAGTTTTTCTTGATTCTTTTGGAACATCTTAAACATCTTCATAAACTGGCCGTAGTTCTCTTTTTTAAGGGCATTAAAGCAGATTTTCAGAGTTCCTCCAAGTCCTTCATCATAAATCATACCTCGAAGACTCATCAAGGTCATTTCGCCGACAAAGGTGTCCACACGGTCAAAAGCATGAGAACGTGCCAGCTGAATCCAAGATCCCTCGGTTAGAGGCCCCACATTGACATTAATCGCACGAGATAGCTCTTCTCGGATATTCTCATCTCTTTGTTTGAGCATGACATCATGGGTGAGGAGCACTCCGCCTGGCTTCAACACCCGGTAGTACTCATCCATACATTTGGCCTTCCTCTTATCTGTCTGCATGGTCAGCATGGCTTCATTGATAACGATATCAAACGAATTATCATCATAAGGCAGCTTCATGGCATTAGCCTGCTCAAATGTGACGAGTTCACTAACACCAGCTTTGTCTCCATTAAGCTTAGCCTGAGCCAGCGCAGCCTTATCCAAATCAACTGCAGTAATCTGGCAGCCATATTTTTTAGCCAGCTCGATTGTTGTGGTCCCCATATTGCAGGCAACTTCTAGAACTTTCTTATCGCTGGAAAACTGCCCCTGCTCAATCAGCCAATCCGTTGCCAGCTTACCACCGGGACGAAGGCGTTTCTTTCCTAATTTTGCTAAAAATTTATGACCTGCTTCTGCCATTTCTGAACCTCCATGATTTTGATTGCT

>c183_g117

AAGGAAAGAAAGAATGTTAAAGGATCTTAAAGAATTCTTGCTGCGCGGGAACGTGATTGATTTGGCAGTCGGTGTGATCATCGCCAACGCCTTTGGAGCAATCGTCACTTCATTGATTACTGACGTAATCACTCCCCTCTTCCTCAACCCAATTTTGAAAGCTGCAAACTTGGAGCAAATTTCTCAATTGAAATGGAACGGGATCGCTTATGGTAACTTCTTGAGTGCTGTAATTAACTTCTTGGTAATCGGTACTGTTCTTTTCTTCATCGTCAAGTCTGCTGAAAAAGCACAAAGCCTTGCTAAGAAGAAGGAAGAAGTTGAGGAAGCGCCAGCTGGACCAACTGAATTGGAAGTCCTGCAAGAAATCAAAGCTTTGTTGGCTGAGAAAAAATAAAGAGAATTCACTGATGAAAATCAGTGTTTTTTTCTTGTCCATTGCACACAATCTTCCTCTTCTCTCCAAAGAGTGAAAACAAAAAACAGACCGGAGTCTGCTTTTTTGTTCAATTAGAATTTTTTACGTTTACGAGCTGCTTCTGATTTGCGTTTGCGTTTTACAGAAGGTTTTTCATAGAATTCACGTTTGCGTGTTTCTTGAAGAGTACCAGCTTTAGTAACCGCACGTTTGAAACGACGAAGTGCATCATCAAGTGATTCATTCTTGCGTACTACTGTTTTTGACATTTTTTTCACTCCCTTCAAGTCCAAAATCTATATAATCATACCACATATAGAAAAGAGTGTCAAGTATTTTCTGTAAGTATAATGTAGCTCCTTTTTATTATACAAACAAGAAGACCAACTGCTCTGCTGCAGCTGGTC

>c183_g118

ATTTTTCTTGCCTTATGCTATACTAGATTTATGTTAGATTTGAAAGAATACGGAATTGAGATGTGGGAGGCAAATAAAATCGCCTCTTTTCGCGAGAAATTATTAACTTGGTATGATGAAAACAAACGTGACCTGCCTTGGCGCAGAACAAATAATCCTTATCATATCTGGGTTTCTGAGATTATGCTGCAGCAGACTCGGGTGGATACGGTGATTCCTTACTATGAGCGCTTTCTTGACTGGTTTCCGACTGTAGCTGATTTAGCTCAGGCGCCAGAGGATAGACTGCTCAAGGCTTGGGAAGGGTTGGGCTATTATTCGCGGGTGCGAAATATGCAAAAAGCGGCCCAGCAGATAATGACGGATTTCGCTGGAAAATTTCCTGATAGCTATGAAGGAATTGCTAGCCTCAAAGGGATTGGACCTTATACAGCTGGTGCCATTGCTAGTATTGCCTTTGGCCTAGCTGAGCCTGCGGTAGATGGCAATGTCATGCGGGTGCTGAGTCGATTGTTTGAAGTTGATTTAGATATTGGTCAACCTAGCAATCGTAAGGTCTTTCAGGCTATGATGGAGATTTTGATTGATCCAGACAGACCTGGAGATTTCAATCAAGCGCTGATGGATTTGGGGTCAGATATCGAAGCTCCTGTTAATCCTCATCCGGAAGACAATCCTGTAAAAGAGTTCAGCGCAGCCTATTTACACGGAACAATGGATAAATATCCAATCAAAGCACCTAAAAAGAAGCCAGTTCCGGTCTATCTGCAAGGCTTGATTATTGAAAACGAACAGGGACAGTTCTTACTTGAAAAGAATGAGGCTGATGGCTTGTTATCAGGTTTTTGGCATTTTCCCTTGATTGAAATCGAGGAATTTCAAACTGAAAATCAGATGTCCCTCTTTGAGGTGGCAGAAAATCAGTCAAGTCTGGATCTGTCTCCTCAAGAAAGCTTTGAGCAGGATTATGATCTAATTGTTAATTGGCAGCAGCAGTCCTTTCCAAAGGTGCAACATGTCTTCAGTCATCGTAAGTGGCATATTCAGCTAGCTTATGGACGAGTCAAAAATAGCCAACACGCAGCTGATGGAGAGGTCTTGTGGCTTCATCCAGAAGACTTTGGCAATTACCCCTTTGCCAAACCCCAGCAGAAGATGTGGGAGGCTTTTCAGGAAGCTAGAAATGAATAAAAAATCTGAGACTAATTTGGTCTCAGATTTTTTTGAATCAATTTTACAAGTTTGCAAAAGCTTTAATCTCATCTGCAGTCATAGAAGAATCACAACGAACAGATACTTGGCCGTCTTGAACATGAACAAAACCAGGAACCGTCGGAATACTATATTCAGAGCGGAAGGCTTGCAGTTTCTCTAATTCGCTGGCTTCTTCGCTATTGATAAAGAAAATATGCGCCTTGGTATCAGCGACAACGCCAGCAAGAGTAGCAGCGAATTTACGACAATAAGGGCAAGTTTTACGACCGACGAAGAAAGTTGCAGTCTCTTTGTTAGCAATCGCTTGACGGGCACGGTCAACAGTTGTGACTTCCAAGTCTTTAATATTTTGAGCAAATTGTTCCATAAATAATCCTCATTTCTTTTGATAAGACTAGTATGCCATAAAAAGCAGAAAAATGCTTGATTTGGAAATGAAAAACCAGAGAATATGAAATTCATCCTCTGGTTGATTGGTTGATAGTGTTGTTTTACTTGATAAAGTCATTAATCTCTGCTTCAATAGCAGCAATTTTTGCTTGAGCATCTTCATTGCTGTCACCGACAACGGCAATGTAGAACTTGATTTTTGGTTCTGTTCCGGATGGCCGGACAGCAATCCATGAGCCATCAGCCAAGGTGTATTTGAGCACATCGCTTGGAGGAGTTGTCAGAGCTGTAACAGTACCGTCTGCAGCGGTAGAAGTCTGTGCCTTGAAGTCTTCAGTAACAGAAATGGCAGTAGCGTTGAATTCTTTCGGAGCATTGTCACGGAACTTAGCCATGATAGCCTTTATTTGCTCTGCTCCATCAACACCAGACAAGGTCACAGAGATAGTCTTTTCAGCAAAGTAACCGTACTCTTTGTAGATTTCTTCAATACCGTCAGCCAGAGTCAAGCCGCGTGAACGGTAGTAGGCAGCTAGCTCAGCTACAACCAAAACAGCTTGGATAGCGTCTTTATCACGTACAAAAGGTTTAATCAGATAGCCGAAGCTTTCTTCAAATCCCATCATGTAGGTGTGGTTGTGTTTTTCTTCGAATTCTTGAATCTTTTCAGCGATAAATTTGAAACCAGTCAAGACATTGAACATGGTTGCACCATAGCTTTCAGCAATCTTAGTCACCAAGTCAGTAGAAACTATAGACTTGCAGAGAGCAGCGTTAGCTGGCAGAGTTCCGGCACTCTTATGAGCTTCCAAGATGTACTTAGCCATGATGGCTCCGATTTGGTTACCTGAAAGATTGAGATAGCTGCCGTCTTTTTGCAGGACTTCCACACCGACACGGTCAGCATCTGGGTCAGTTGCAACCAAGACATCTGCACCAACCTTGCGACCTAATTCCTCTGCCAAGGCAAAGGCTGCTTGGCTTTCAGGGTTTGGAGACTTGACAGTAGAGAAGTCAGGATCAGCTACAGCTTGAGCTTCAACAACTTCAACAGAATCAAATCCTGCTTGAGCCAAAGCGCGACGAGCCAGCATTTCACCAGTACCGTGCAGAGGAGTATAGACAATCTTCATGTCCTTACCGTATTCATCAATCAGTTTTTCGTTGATATTGACATCTTTAACTTCCTTGAGGTATTCAGCATCAATAGCATCGCCAATCACTTCAATCAAGCCAGAGGCTTTTTCGGCTTCAACATCAGCTACTTCGATAGCGAAAGGATTTTCAATCGCACGGATGTAGTCAGTCAATGCATCTGCATCATGCGGAGGCATTTGTCCGCCGTCTTCACCATATACCTTGTAGCCGTTAAATGGAGCAGGGTTGTGGCTGGCTGTAATCATGATACCAGCAAAAGTCCCCAGATGGCGCACTGCAAATGAAAGTTCTGGAGTTGGGCGCAGGCTTTCAAAAACATAGGACTTGATGCCGTGTTTTGCTAAAACAGCAGCAGATTCAAAGGCAAATTCTGGTGAGAAATGACGGGAGTCGTAAGCAATCGCAACACCGCGTTTTTTGAACTCATCACCTTTTTCTTCAATCAAACGAGCCAAACCTTCAGTTGCCTGACGGACAACATAAATATTAATACGGTTGGTACCAGCGCCAATTAAACCACGCATACCAGCGGTACCAAACTCAAGATTGGTGTAAAAAGCATCTTCCTTAGTTTTTTCGTCCATACTGTTCAAGTCTTGGCGAAGATAGTCAGGAAGTTCAGCATAATCAAGCCATTTTTTGAAGTTATCTTGATAAGTCATAGGTGGAGTCTCCTTTATTTTTGAAATGAAGAGTTGGAAATTTTAGTAAAAAATCCTGCTCTTAAATTTTCTTAATCGCTTACATTGTAGCATATTTTCATTAATTTTGAAAGAGGTTACCTAGAAAACGATGAAATACTTTTCAAGGCATTGTTTCAAGGATTTCAAGATGGTAAAGTTGAAATGCGGGATTAAAAAAAGACCAAAGCAAGGCTTGGTCTGGAAAGCTAATTTATCATCATTTTTTGGCTTTTTCAAGCGCAGGAATGATAGTTGCTGTCAAAATCGCAGAAATGATTAACTCGGCAATGGAATTTGCTGATAGAATCACAGCCAGAAGCGCTTTGATATCACCTTGGTATACATTTGCAAAGAGAAAGAAGATTCCGCCCAAGACAAAAATGGTATTGGTGGCAGAACCAACGGAACCAGCCACAAGCAAGCCGACCTTATTTTTCATTGCTTTATATACGAAATAAGGAGTGATACCGATGAGAATGCGAGGTATCATGGCAACAACAAGTGAAGAAAGGCTTCCATTTGGCACAAAAGGGCTAAAGAGATAGCTGGTTGGCAGCAGCACCAAGGTATTGGTAATAACGCTGATACATCCCATGAGGCCGCCTAGGACAGCCCCGACTTTAGGGCCATAGAGGATACTGGCGATAATCACTGGGATGTGAATAATTGTAGGCTTAATCGGTACTGGAAGCAGGTTAAAAATGACTGAACTGAGCAGGTGCAGTACCAACATGATGGCAAAGAAAATAGCAATTTGAGCAATTTTAGATTGTTTTTTCATAAATAAGTTCCTTTACTGTATCAATAATTGTATCAATTTCAGCTAGAGCACCTGTCCCCTGATCGCCACAGGCTAGAACAGCTTCGCGCGGCTGGATAATCTTCCATCCAAAACGCTGCAGACGCTCCAAATTGTGTTGGGTTAGTGGATTTTCAAACATCTTAGTGTTCATAGCTGGAGCCAAAACTTTCTTAGTATGAGTCGGCAGAGCCAGGGCAGTAGCTGTCACCATATTGTCAGCTAAGCCGTTGGCTAGTTTAGCGATGGTATTGGCTGTAGCGGGGGCCAGCAAAAAGAGGTCAGACTTTTTAGCGATATCAATATGATTGACCTTGCTCGGGTCAGGTTCTTGCATAATATCAATTGCAACTGGCTTTTGCGACAAGACTTGCAGGGTCAGCGGTGTGATAAATTCTGTCGCAGCATTGGTCATGAGGACAGCTACTTCAAAGTTCTCTTTCTTTAGACGGCTAGTGATGTCAGCAGCCTTATAGGCTGATATACTTCCGCTGACAGCTAAGGTAATCTGAGTCATCTTAATCCTCCTTATGTATTGTGTATATGCAGGCAGAGGAGCTGAGCAATTTCTTCTTTGGATTGAGCTTGAGTTACAGTATCTTTTCCGACGAGATAGGCTCTGTGCCCATGGTTAGAAATCTCTGTCAAATCATTGGCGACGATAATTTCTGCTTGATTTTTTATGAGACTGTCTCGTGCGGTCTTGAGTAACTCTTCCTTAGTAACATTGACCAAGAGTTTGAATCCAATCAAGCGAATGGCTGGATTCCATTTTTTTACCAGGCTGATGACTTTTGGAGTCTTTTTGAGAAAAAGGACTTGAACATCATCCTGAGAAGAAATTTTGCTTTCTGAGTTAGTCTTATTCAAAAATTCAGTCATGTCAGGGCTAGCCGCTACAGCTTCCAAACCAGTCATGTAGACGGGAGTATAGTCAGAAACAGCCATTGCATGAATCAGTACATCATGTGTGTGAACGAGCGGCTTCAAGCTTTCAAGCAGTTCTGCAACATTTTCAATCTGGACAATGGTAAGATTGGGATGAGCTGCCGGTCGAACAGCCTTGGGAGTCGTCACCAGAGTCACCTGATCGCCCTTGTCTAGGAAAGTTTCAGCTATGATTTTCCCAAGCCGGCCTGTCGAATGATTGGTAATAGAGCGGAC

>c183_g119

CTACTATAAAGGCTAAATTTTTGTCAAACCTCTTAGAAATCTTCTTCTTGCCACTTTTCCAAAAAATGATATAATTTGAAGAGTGAAGATTACCAAGGGCTTAGCCCTTGTGAGAGAAAGGAAAGAAAATCTTATGATGAACATGCAAAGCATGATGAAGCAAGCACAAAAGCTTCAAAAACAAATGGAAAAAGGACAGGCAGAACTAGCTGCAACAGAATTTACCGGCAAATCAGCTCAAGACTTAGTCGTTGCTAAACTGACTGGCGATAAAAAGGTAGTCAGCATTGACTTCAATCCAGCAGTTGTGGATCCTGAAGATTTGGAAACTCTGTCAGAGATGACTGCGCAGGCCCTGAATCACGCGCTGGCTCAGATTGATGATGCTACTCAGAAGAAAATGGGCGCTTTCGCAGGCAAATTGCCTTTTTAAGCAAGATAAATAACCTTACCTCTTGACTAAACATAAGTCAAGCAATACTCGAACCAGCAAAAAAGACTGAGATTCTTCAGTCTTTTTTGATTTATAGGCGTTCCCTCACCAGTCCACTACACAAAATTAAGCAAATCTGCTGCCTTACTCATCAAAAGCACGGCATAGTCGGGATTGTTTTGCAGCTCCTTGATAGAGCTCTGCACCAGCTCTAAATCATCGGTAATCATCCCATCAACGCCCAGACGGAAGGATTTGCCAATGCTGTCTGCATCATTGATGGTCCAGTCGTAGAGCTGCTTATCTGTCGTCCATAGCTTATCGACAAAGTTTTCATCAAGGGTCGAGTACTCCATCGTATAGCCGGAAGCCTGCGTCCGTGGGAAAATAGTATTGTAGGGTAGGATAAAAAAAGTCGGTATGCTCTCATCATACTGGACAGTCTTGTCAATAACCTGATAGTCCAAAGACTGAATCTGATGGCCATAAACCTTGATATTAGCTCCATATTGGCTCAGGAAACGGTCCATCATATCGTCTGAGTCCAAGCTGCTGGTCTTGATTTCTATCAAGAGTCGCTGCCCCATCTGATTGGCTCGTTTGAGATAAGCATCAAAGCTGGAAATTTTGGCAGTGTGTCCATTTTCTGAAATATCTAAAGCCGTCAGCTCTTGCAGAGTCAGCTCTTGCGGTCTAGCATCGATGCCGGCCAAAGCTTCTAGATTGGCATCGTGCATCATGACAAACTGGCCATCCTTGGTCTCCTGAACATCCATTTCGATATAGTCTGGCTTGAGCAGAGCTGTCTTCTCAAGCGACTCAACTGTATTCTGGACACCATTTCCCTGCGAAACTCCACGATGCGAGATGGTCAGAGGGACGTTTTCCAAGGGCAGATTGAGATAGGCAAAGCCTTCAAGGGCAAAGACAGAGCTAGTGACCAAAAGGATAAACCAGCGCATGAGAGGCAGGCCTTTTCTGTAGCGGTATTCAGACAGCTTACTGTCTGTCAGAAAAGCCACAAACTTAATCAGAAAGTAGGCTACCATAAAGTAATAAGCCAGCTTGATGAGGCAGTAATTGACAACCGCTGCTGTCAAGGCTATCTGATTGGACTGGCCATCCGCATACTGCTGCAACACTAAAATCGGAATGCTGCTTAAAAGAAAGAAGAGAAAGCTCTTGGCCAATATCCAAAAGAGCTGCCAAGTATAGCGAATCAAGTGCCCCTTGGTCTTATCCAAACTATACCTAATCGCATCTCGTAGGCGGAAATGCTCAAAAAAGAGCTTGGGCAGGGCAAACATTAGGCGGACAGCTATCAAAAAAAGCAGAAAACCTAAAGCATAGATTGATAATTTCATCCATAAGGCCGTCTTGAGATAGGTCACGATAAATTCTGGAATCAGAATTTTATTCAAGTAGTAGATTTTAAGAATTTGCCGCAAAAAGGGAAAGATAAAGCCCATATACAGTGCAATGAAGAGGACCTTGCTGGGTCGGGCGTAGCGAATCAGCGAGCCGCTGTCTTTGAAACTTTTCTTGATAAACTGAAAAGCGCTTCGCTCTTCCTCATCTAAAAGATTGCGGAGCCCCATAAATATCAGTCCAATCTGAAAATAAGCGACAAAGAGATTGGCTGCAAAAAGCGCTAGAAAGGCCAAGCCAACCCAGACATTTGAAGTCAGAACCTTCAGGGCATTCGTATAAGATAAGAAGAGGTAGCCTGTCTGCTTGAGCAGAGTCTCTGCTGCAAAGGAGTTGAAAGGCACCCAAGCCAGCTCCATGAGCATAAAGACCGTGAAAAAGAGCAACAGAATTTTATCAAGATTGCGATAGAGACGTAAAAGTCCCAGTCTTTGTGATTTCATTTTCCTCCTTTATTTGTGAACGATACAAAACTTGCTAAAAAGCGATTCTTATTTAAAACAAGGTTGAGACAGATGTCCCAACCTTATTTTTATTTTCCAAATAAACGTGCCCAGAAGCCCTTGCTTTCCTGCTCCTGCACCTTTTCCTTGGCTTCATCCAACTCCAACAGCAAGGTTTCACGCTCATTCATAGCCTTAGCTGTCAGCTGCTGCTGCTGGTCCAGCTGCTTGTCCTTTTCAGCAATCTGGACATCCTTGATGCGCAACTGCTCATCTTTCTTAGCCAGCTGGCTATCCTTGGCTTTAAGCTGTTCATAGAGACGCACAATCTCAGCATTTTTTTCGTCCACCAAGATTTCCATCAGCTCGCGTTGCTTCACATCTTCGCTAACAGGTTCATCTTCAAAAATAGTTTTCTTATAAATCTCTTCCAGCTTAATCAAGCCGCTGCGGGTCACCACCGTGACTCCTTTTTCATTCTTTTCCGTATCTTCTGGCGGCAGTGCCTTGACACGATTGTTAATCGCCTGGCGGCTAACTCCAAGAATCTCGGCTAACTCGCTGACTGTCTTTTCAATTGCCATAATTTCCTCAAAAACTTTTTCTAGTTTGTAGATACCTAAATCTTATCATATCAAGCCTTAAC

>c183_g12

GTTTCCTATTACAAACGTGCATTCAATTCTTTGCTCAATTCTTCAAATCCTGGTTTACCAAGAAGGGCAAACATATTTCTCTTGTAGGCTTCAACCCCTGGTTGGTCAAATGGGTTAATTGCATTCAAGTAACCTGAAAGGGCGATGGCCAATTCGAAGAAGTAGATGATGTAGCCAAGAGTGAAGGCATCTTGCTCTGGAAGAGTCACATACATATTTGGTACGTCACCATCTGTGTGGGCAAGAAGAACACCGTCAGTTGCTTTTTTGTTTACAAAGTCAACGTCTTTTCCTTGAAGGTAACCAAGTCCGTCAAGGTCTTCTTCCAAGCTAGGAATAATCACGTTCTTACGTGGTTTGTCAATACGGACAACTGTTTCAAACATGATGCGAGTTCCTTCTTGGATAAATTGACCCAATGAGTGCAAGTCAGTTGAGAAGTTTGCTGAAGTTGGGTAGATACCTTTTTGATCTTTCCCTTCTGATTCACCAGCCAATTGTTTCCACCATTCTGAGAAGTATTGAAGTGATGGCTCGTAGTTTACCAAGATTTCAGTAGCGTAGCCTTTGCGGTAAAGGATGTTACGAACGGCTGCGTATTGGTAGGCTTCATTTTCTGAAAGTTTGTCTGAAGTGTAATCTTTACGAGCTGCATTCGCACCTTCCATAAGGGCTTTGATGTCCGCACCTGATGCAGCGATTGGAAGCAAACCAACTGCGGTCAATACTGAGAAACGTCCACCGATGTCATCTGGAACCACAAATGTTTCCCAACCATTCGCATCTGCTTCAACCTTAACAGCACCTTTTTGGCGGTCAGTTGTTGCATAGATACGTTTGTTGGCTTCTTCTTGACCATATTTCTTAACCAAGAGTTCTTTGAAAACACGGAAAGCAATCGCTGGTTCAGTTGTTGTACCTGATTTAGAAATTACGTTTACTGAGAAATCTTTGTCAGCTACATACTCTACCAAGTCAGCAAGGTAAGTAGATGAGATTGAGTTTCCAGCGTAAAGAATTTGTGGGGCTTTGCGTTCTTCTTTTGTTTGCAAGTTTGAAAAGTGGTGGTTCAAGAAGTCGATGGCTGCTTTGGCACCAAGGTAAGATCCACCGATACCGATAACAACCAAGACATCGCTGTCTGATTTGATTTGCTCAGCAGCTTTCAAGATGCGGTCAAATTCTTCGCGATCATAATTTTCAGGAAGGTCCAACCAACCCAAAAAGTCGCTGCCAGCACCAGTTCCTTTGCGGATCAATTCATCTGCAGCTGTTACTTGTGATTGCATGTACTCCACTTCATGTGGGGCAACAAATTTATCTAAAACTTTTGAATAATCAAATTTAATATGTGACATGATATTCCTCCATTTTTTATTCCACTCTATCATATCGCTTTCATACTTATTTAGCAAGTAATTTATTGTTTTAAAACGCTTCCAATTTAATTTTTTATAAATGAGCTAATCCTAGTAGCCATCTGAAAGCAATCGTTTGCGTAAGTCGGAAAATTTTAGCCCTACTAATAAGAATATTTTTGATTAACAAGAAAGCCAATATGCTTCTTAAGATTGGATGTCAACTGAATTCATTTCAGATTCGTTCGAACTAGATTCACTGCTCCAATATTCATCTGCGCCTTTCAGGCTTGATTCATTCAACATTGGAACGTAGATATCGCTTCGCTCAAACTGCATCAATGTACCTAAGTTCTTTTGTGTCCTTCGGACTTAATTCACTC

>c183_g120

CTGAGATTAGATGTTTTTGTAAGAGTTTTATTCCTAAGTATGGTAAAATAGTTTTCGGAATATACTAGAATAAAAGGATTTAAAGAGGTCTTATAATGACAATCGGAATCGACAAAATCGGTTTTGCGACCAGTAATTATGTCTTAAAATTAAATGATTTAGCGGCAGCCCGTGGGACTGACCCAGACAAGCTTAGCAAGGGGCTCTTACTCAAGGAACTAAGCATTGCTCCTCTGACTGAGGATATTGTTACCTTGGGAGCGGCGGCAGCAGACCCCATCCTGACAGCAGAGGACAAAGAAAAGATTGATATGGTCATCGTGGCTACTGAGTCAGGGATTGACCAAAGCAAGGCGGCAGCTGTTTTTGTCCATGGCCTTTTGGGTATCCAGCCTTTTGCCCGCAGCTTTGAAATTAAGGAAGCCTGCTATGGAGCTACTGCTGCACTGGACTATGCCAAGCTCCATATTGAAAAACATCCAGACAGCAAAGTTTTGGTGCTAGCTAGCGACATTGCCAAGTATGGCATTAACACACCTGGCGAGCCAACGCAGGGAGCGGGAGCCATTTCTATGCTGATTAGCAGCAATCCCCGTATCCTCGCTTTCAATGATGATAATGTAGCCCAGACACGCGATGTCATGGATTTTTGGCGCCCAAATTACTCGACAACGCCTTATGTCAACGGCCTCTATTCAACCCAGCAATATCTGGATAGCTTAAAGACAACTTGGGCGGAGTACCAGAAGCGCCACAAGCTGGCTCTCAAAGATTTTGCGGCCTACTGCTTCCACCTGCCTTATCCAAAACTGGCCCTTAAAGGCCTCAATAAAATCATGGATAAAAGCCTGCCCCAAGAGCAGCAAGACCAGCTTAGAAAGAACTTTGAAGCATCTATTCTCTACAGCCAAAAAGTTGGAAATATCTATACAGGCTCCCTCTTCCTAGGTCTTCTGTCTCTTTTAGAAAACTCTGACAACCTCAAGGCTGGCGATCGGATTGCTCTCTTTGGCTATGGCAGCGGAGCTGTCTCTGAAATCTTTAGTGCCAATTTGGTAGAGGGCTATGAAAAGCATCTATCTCAAACACGCCTAGAGGAATTAGACCAGCGTCAGGCTCTTTCCATCCCGAATTACGAGCGAATCTTTTTTGAAGAAGCTGAGCTGGATGCGGAAGGCAATGCCAGCTTCTCTGGCTATGAGGAACAAAGCTTTGCTCTGGCAGAAATTGCAGAGCATCAACGTAAGTACATCAAAGTTGAGAAATCATCATGAAAGTAAACTGGACTGGATTTTCTAAAAAAAATCCTGCTGAACGGCTGCAGATGCTGAAAGAAAAGGGACTTTTACAAGATGAACATTGGCAGCTGCTAGACAGTCAGCAGACTTTGCCTCTGGAAACAGCCAACCAGATGAGTGAAAATGTGCTGGCCACTCTAGCCCTGCCCTACTCTCTGGTGCCAGACTTTCTGGTGGATGGCAAAAGCTATCAAGTTCCATTTGTGACAGAAGAGCCATCTGTGGTAGCAGCAGCCAGCTTTGCCGCTAAGATTATCAAACGTTCAGGCGGATTTGAGACCGAGGTTCACAAACGCCAGATGATTGGGCAAATCGCGCTCTATCAGGTTGATAATGCAGACCGAGCCATTAAAGATATTCTCATGAAAAAGAAAGAGTTGCTGGAGCAGGCCAACCAAGCCTATCCATCGATCGTTGCCCGTGGGGGCGGAGCTAGAGATCTCTGGCTGGAGCCAAAGGAAGACTTCCTCATTTTCTATCTATCTGTGGATACGCAGGAAGCTATGGGAGCCAATATGCTCAACACCATGCTGGAGGCCATCACCCTTCCTCTGAAAGATCTGACTGGCGGTAAGAGTCTGATGGCTATCCTGTCAAACTATGCAACAGATAGTCTCGTAACAGCCCGCTGTGTCATCGGCTATCGCTTTCTCAGCCGGGATACGGCCGAGGCGGAACTTCTTGCGGATAAAATGCAGCTGGCCAGCAAATTAGCCCAGGTTGACCCTTACCGGGCAGCTACCCACAATAAAGGTATCTTTAATGGTATTGATGCTTTGGTGCTGGCTACTGGAAATGACTGGAGGGCTGTAGAAGCCGGTGCCCATGCCTATGCCAGCCGAGAAGGAAGCTACCGCGGCCTCTCTACTTGGACGGCAGACCCAGACAAACGCCAGCTTCATGGTCAGATGACCCTGCCCATGCCCATTGCAACTAAAGGAGGTTCCATTGGCCTCAATCCCGCAGTGGCAGCCAGCTTTGACTTGCTGGGGCAGCCTCAGGCCAAGGAATTGGCTTCCCTCATCGTTTCAGTCGGATTGGCACAAAACTTCGCCGCCCTCAAAGCCCTAGTCAGCACTGGCATCCAAGCCGGTCATATGAAATTGCAAGCCAAATCTTTAGCATTGCAAGCTGGAGCCCAAGGCGAAGAAATCGCTGCTGTAGCCAGTTGTTTAACAGTCAAGAAGACTTTCAACCTCGCTACTGCTCAGGAA

>c183_g121

AAAGGAGAAAGCGATATGACTATTACAAAAAAAGCCTTTATTGGAATTCTATCTCTGACAGCAGCAGTCCTATTGGCTGCCTGCTCAGGCAATAATAACCAAGGAGGGAATACTAGTTCATCACAGAATACACAAGGCCAAACATCACAATCTGCACAAAATCAGACTTCGCAGTCTTCAGCCGCATCATCCTCTAACCAGCCTTCATCATCAAATGCAGGGCAGGCTACGAATCTAGATGGGCGCTACCAAGCAACCGACCATGATGGCGATCAACATGTCCTAGAAATCAATGGTACGACTGGTACTTGGACTGAGACTGAGGTTGATGGTGATAAGGAGATCAAGCAGGTACAAGTAGATGCTGCTAACCAAAGACTGATAGTTGGTGATGATGCTAAAAGCTATCGCCAAAATGGCAATCAGCTGATTGTGGACGAATTGGATGATGATCCAGATACTCTGACCTTTACCAAGCAGTAGAGCGCTTCTCATTCTGCTCAGGACAACAGGATGAGCAGTGAGGGAATCAAACTAGGAAACTCTGAGAATGGAGTTTCCTTTT

>c183_g122

AGTGATACTCCTTAAATATTTTTTAGACAAAGTCTATTTTATCAAGTATAATGGATAATGTAAAGGTCATTTTTAATAAAAATAAGAAAAAGAGGGAAGAATATGCGTTTTAATCAGTATAGTTATGCAAAAACGAAACGGGAAAATATGTTGATAGAATTAGCTGAATTAGGCTTTTTTTATGACAGCAATCGCTCTGATAAGGAAAATCTTGAAGATTTTCTACGCACCAGCTTTTTCACTTATAAAAATACAGATTATCCCTTGAAGTCCTGGGCTGCTGACAGCCAAACAGACCTGCTGAGCTTTTTCCAGTCTGACAGAGAACTGACGGCGTCTGTTTTTTACACTGTGGCCTTTCAGCTGCTTGAATTCTCGCCCTTTATTGATTTTACAGATGTTGAGGCCTTTCGCCAAGAAACAGACTTTCCTATTACATTTGGAGACTTATTGGAAAATCTCTACCAGCTGCTCAACACTCGGACAAAAAATGGTAATTTGTTGATTGATAAGCTAGTCAGCGAGGGTTTGATTCCTGAGGACAATACCCACCACTGCTTCAACGGCAAGAGCCTAGCAACCTTTTCCAGCCATGATGCTATCCGAGAGGTAGTTTACGTGGAATCACGTGTGGATACTGATCAAGACGGTCGTCCGGACCTTATCAAAGTCAGCATCATCCGGCCCCGTTATCAAGGACCAGTTCCTGCTGTCATGACTGCCTCTCCTTATCATCAAGGAACCAATGATCCCGCCAGCGACAAGGCTCTCCATGATATGAATGTGGACTTAGCAAAAAAAGAACCCCATCAAATCACGGTACAAGATCCTGAACTTAAATTGCTCCAGCTGGATTCACCAGTTCCTGCCCAAGAAGTATCTGAAACCGAGGAAAAATTAGGTCATATCGGCACCTACACGCTCAATGATTACTTGCTGCCCCGCGGCTTTGCCAATCTCTATGTGTCTGGCGTAGGAACTAAAGACTCTGAAGGGCTGATGACCAGTGGCGACTATCAGCAGATCGAGGCCTATAAGAACGTTATTGATTGGCTCAATGGCCGTTGCCGAGCCTTCACCGACCACACACGCCAGCGGGAAATCAAGGCCACTTGGTCCAACGGAAAAGTGGCTACGACCGGTATCTCCTATCTGGGCACCATGTCCAACGGACTGGCAACGACTGGTGTAGATGGACTGGAGGTCATCATCGCTGAAGCAGGGATTTCTTCTTGGTACAACTACTATCGCGAAAACGGCCTTGTCACAAGTCCTGGCGGCTATCCAGGAGAGGATTTTGAATCCCTGACCGAACTGACCTACTCCCGCAACCTGAGGGCTGGTGACTACCTGCGCAACAACGACGCTTATCAGCAAAACCTAGAGCAACAGCGCAAAGACCTAGACCGGCAAACCGGAGATTACAATCAATTTTGGCATGACCGCAACTACCTGCTCCACGCAGATAAGGTCAAGGCCGAAGTCGTCTTCACCCATGGCTCTCAAGACTGGAACGTTAAGCCTCTCCATGTCTATAATATGTTTCGGGCCTTACCGCCCCACATCAAAAAGCATCTTTTCTTCCATAACGGTGCCCATGTTTACATGAACAACTGGCAGTCCATCGACTTCCGTGAGTCTATCAATGCTCTGCTGAGCAAGAAACTGCTGGGTTGTGAATCAGACTTTGTCCTGCCAGCAGTCATCTGGCAGGACAATAGCCAAGCTCAAAGCTGGCTGACCTTGGAAGACTTCGGTGGACAAGAGCAGAATCTTCATCTACAACTAGGCCAAGACTGCCAATCTATCCAAAATCAATATTCAGAAGAAGACTATAATCGTTTTGCTAAAAACTACCAAAGCTTTAAGACTGAGCTCTTTGACGGTAAGGTCAACCAGATTACTCTGGACTGGACCTTGGAAAAGGACCTCTTTCTCAACGGAGCGACCCAGCTCAATCTTCGACTCAAATCCAGCACCGATAAGGGATTGATTTCTGCTCAATTGCTGGACTTCGGACTAGCTAAACGACACACACCAATTCCTACGCCTATTGAACCTAGAGTCATGGACAACGGCCGCTACTATATGCTGGACAATCTGGTTGAACTGCCCTTTGCCGAAACGCCCCATCGCGTCATCACCAAAGGTTTTCTCAATTTGCAAAATCGAACCAATCTACTGAGCGTCGAAGAAGTCACTCCTGACCAATGGCTGGAATTTTCCTTTGAACTGCAACCAACCATCTATAAGATGAAAAAAGGAGACCAACTGCGCCTCGTTCTCTACACTACAGACTTTGAGCATACTGTCCGTGATAAGATCGACTATCAGCTGACGGTAGATTTGGAGCAATCTAGTTTGGATTTGCCAACAATGACATCACATTAATAAAAGAAAAAGACGCTGAAATTCAGCGCTTCAGATTGAAGACAAAGTCCTAAGAACAGAAGTTTCTAAGGGCTTTTCTTTTATTGTAGATTGAGATATTCCTCTGCAT

>c183_g123

GGCCGGTATAGCTGGCTGGATTAGCAGCGACTTCTTCCGGTGTACCCGTCGCAATAATCGTACCGCCACCGACACCGCCTTCTGGTCCTAAGTCGATGATATGGTCAGCTGTCTTGATAACATCCAGATTGTGCTCGATAACGAGTACTGTATTGCCATCGTCCACAAAGCGTGATAGCACTTGAAGGAGCTTGGCAATGTCTTCAGAATGCAGACCCGTGGTCGGCTCATCCAAGATATAGAAGGACTTACCAGTCGAGCGCTTGTGAAGTTCGCTGGCCAGCTTCATCCGCTGGGCTTCACCACCTGACAAGGTCGTGGCTGGCTGTCCCAGAGTCACATAGCCTAGCCCCACGTCCTTGATAGTCTGGAGTTTGCGGGCAATCTTGGGAATATGCTGGAAGAATTCTACCGCATCATTAACCGTCATGTCCAGAACCTGAGCGATATTCTTCTCCTTGTAGTGGACTTCTAAAGTCTCGCTATTATAGCGGGTGCCGTGGCAGACCTCGCAGGCCACATAGACGTCAGGCAGGAAGTGCATCTCAATCTTGATAATCCCATCACCCGAGCAGGCCTCACAGCGACCGCCCTTGACGTTAAAGCTGAAACGGCCCTTCTTGTAGCCGCGAATCTTGGCTTCATTGGTCTTGGCAAAAAGATCGCGAATATCGTCAAAAACTCCAGTATAAGTAGCTGGATTAGAGCGCGGTGTCCGGCCAATCGGACTTTGGTCAATATCAATCAGTCGATCCACATGCTCAATGCCGCTGATTTTCTTGAACTTACCTGGCTTAGCAGAATTGCGGTTGAGTTTTTGAGCAATGGCCTTTTTAAGGATAGAGTTGACCAGCGTTGACTTACCGGATCCAGAAACTCCCGTCACAGCGATGAATTTACCCAAGGGGAATCTGGCCGTGATATTCTGCAGGTTATTTTCCTGAGCCCCTGTCACTTCGATAAAGCGTCCATTGCCTACACGGCGCTCCAGTGGTACTGGGATCTCGCGCTTACCTGACAAGTATTGGCCTGTAATAGATTTCTTACTCTTAGCTACTTGCGCAGGAGTACCAGCTGCGACGATCTCACCGCCAAAAACACCGGCTCCTGGTCCCACATCAATCAGCCAGTCAGCCTCCCGCATAGTGTCCTCATCATGCTCAACCACAATGAGGGTATTGCCCAGGTCGCGCATCTTCTTGAGGCTGGCAATCAGGCGGTCATTGTCACGCTGGTGTAAGCCAATAGAGGGCTCGTCCAGGATATAGAGGACGCCACTCAAGTTAGAACCAATCTGAGTCGCCAAGCGAATCCGCTGACTCTCACCTCCTGATAGAGTCCCAGCCGAGCGAGACAAAGTCAGGTAATTGAGGCCAACGTTATTAAGGAAGGTCAGGCGGTCATGAATTTCTTTAAGAATAGGCCGCGCAATCGTAGCTTCGTTGTCAGTCAAGCTAAGCTTGTCCAACTGGACCAAATGATCTGCAATGGACAAGTCTGAAATCTCACCGATATGCAAGCCGTTTTCGCCGCCAACCTTGACAGACAAGGCCTGAGGACTGAGTCGATAACCATGACAAGCCGCACAAGTCAGCTCATTCATATAAGCCCGCATCTGCGTCCGAGTAAAATCACTATTGGTCTCATGGTAGCGGCGGTTGATATTGGTGACAACCCCCTCAAAAGGAATGTCAATATCGCGCACACCACCAAATTCATTTTCATAGTGAAAATGGAACTCCCGCCCATCTGAGCCAAAGAAAATCAGCTGCTTTTCTTCCTCAGTCAGCTCCTCAAAAGGCTTATCCATATCAATGCCAAAGGCAGCCATGGCCTGCTCCAGCATCTGCGGATAGTAGTTGGAGGAGATGGGATTCCAAGGTGCTAGCGCTCCTTCGCGTAAGGTCTTGCCAGCTTCTGGCACAACCACATCTAGATCCACCTCCAGCTTAACCCCCAGACCATCACAGTCCGGACAGGAGCCAAAAGGCGCATTAAAAGAAAAGAGGCGCGGCTCTAGTTCTGGCACCGTAAAGCCACAGACTGGGCAGGCATAGTGCTCAGAAAAAAGAAGCTCTTGACCGTCCATGGTATCAATAACCACATAGCCCTCAGCAATCCGCAGAGCAGCTTCGACTGAGTCAAAGAGGCGGCTGCGCACCCCTTCCTTGAGAACAATTCTGTCTACCACAACCTCAATATCATGCTGCTTGCTCTTAGAAAGCTCTGGCACTTCCGTCACATCGTAAATATCGCCATCCACGCGCACACGGACATAACCGTCTTTCTGAATCTTATCAAAAACGGTCTTATGCTGGCCCTTTTTCTTGCGAATGACCTGCGCTAATATCTGCAGCCGCTGACGCTCTGGCAATTCCAAGACCTTGTCAACAATCTGTTCAACAGACGAAGCCGTAATAGCTCCATGACCGTTAATACAGTAAGGAGTTCCAACACGAGCATAGAGAAGGCGCAGGTAGTCATTGATTTCAGTAGCTGTTCCCACTGTTGAGCGGGGATTTTTACTGGTCGTCTTCTGGTCGATGGAAATAGCTGGGCTGAGACCATCAATCGAGTCCACATCCGGCTTTTCCATATTGCCCAAAAACTGCCGAGCATAGGCCGACAGACTCTCCACATAGCGGCGCTGCCCCTCAGCATAGAGGGTGTCAAAAGCCAGACTGGACTTGCCCGAACCGGACAGACCGGTCACCACGACCAGCTTGTCCCGCGGAATTTCTACATCAA

>c183_g124

AAAAACTATAGAGATTGCCAAAGAGAATCAGCTCATACATAGTCCAGTCTTCCACTTGGAAAAGATAATCAGCCACCTTATCTAAATCCTCTTGCCTCATACGAAACTGGCTGTCTCGCTGACAAATCAAGCCCTGCATCAAAATCCAATTCAGCTCATAGTAAAGAGGAGCGCTACTGGCCTTGGCCTTTTCCAACTGCTCCGCTTGCAAGTCCTGAAATCCCTTGATGTCATTTGAGTAGTAAAGAGGGATAATCTTACCCATCATGGCCACATGCTCATGCTGCTGGAAATTCCGTGCCTTGTCCATAAAATTTTCAAGGGTCACATGAATATTGTCCAAGAGATCTAAAAATTTTGAGAGGGTCATGTCCGACTCACCCAGCTCAAAACGAGACAGCTG

>c183_g125

TTTAGAGCTATTCTTATTTACTCCTCTTTTGACGCCATTTCCGCCGAACTTCCTCACGGTAGGAGGAGTTCCCAACAAAGCGGAGAATATTGAGCAAAGGAGTACGATTAGGCCCCAAAACTCCGACCAACTCAGCAAAGAGCTCAACACCCAGAAGTAAGCCAATCATCAAGAGCACGCCACCAATTCGACTGGAAACATTCAACAAGAGAGAGATCATGGCAAAGACCAGTGAAATCCCATAGATAACCAGCACTGTCCCTCGGTGGGTCAATCCCAAGGACAAGAGCCTATGGTGAAGATGGTGCTTGTCAGGTGTGTAGAATTTCTGACCAGACAGAGTACGGCGGATAATCGCCAGAAAAGTATCCGTAATCGGCACTCCTAAGATAATCATAGGAGTCACCACCGCAACAGCCGTTGCATTTTTTAGCCCTTGCAAGGACAGAACAGCAATCATAAAGCCGATGAAAAGGGCCCCCGTATCACCAAGATAGATGATAGCCGGGTGGTAATTGTAAGGGAAAAAACCTGCAATTGACAAAACCAAGACGAAAATTGTCAGAGTCAAAAAGAGATTGTGCTGGGGCAAAAAGAAGTAAGAAACAATCCCCATCGTCACCAAGGAGATGATGGACACCCCACTCACCAGACCATCCAAACCGTCAATCAAATTAACCGCATTAGTGATAGAAATAATCCACACCACTGTCAAAATATAGGACAGCCAAGGCTCAAAATGCAGGAAAGGTCCGCCAAAAGGAAGCTTAAAATCATCCAATCGGAAATCAGTCAGCCACCAGATTAGACTGGCTGCTAGAACAATCCCCCCCATTTTCAGCATAGGTGACAGCTCTTTTATATCATCGACCAGCCCTGTAAAGGCAATAATCAAACCACCTAGTACAACCGGCCAAACATAATCAAAATAGGTCTGTCCAAAAAAATCTACAGCAACAATTTGCGGCATCAAGAGCAGAGTAGAGATAGAAAAGGCCGCTACAATCGCCAACCCGCCACTGCTAGGCATGGGCTTTTTATTGATGCGGCGAGCATTGGGATAATCTACCGCACCAATCTTAAAGGCTAAGAGCCGAACTAAGGGCGTCAGAATCACCCCGATAAAAAAAGTTCCCAGCAAAACCAAGATAAACTTTAACGGAAAAGTAATCATAGGCATTCAACCTTCTGCAAGCCGGAGACGGCATCTGTCACCAAAAGCAGATGTCCGTGCTCCTGCAGCACTGCCCGAGTGATATCTGAATCATCTGCGAATTCACGCATCTTAGCCAAGAGCCAAGCTGGATAGCGTTCTGGGAAGCCCTCAACATCTACTAAAATCGTCAAATAATAAGCTGAATCATACTTATAAAGTTCTGATAAGTCAATCTTATAGTCCACCGTCTGAGCAAAGGCCGCAGCAGCCTTAATATCTTCAAAGCGCAGGATATAGTATATATAGCGCTCTGCATTTTCTGCCGACTGCTCATCCGCCGCCTCCTGAGAAAGCTGCTCTCTTTCCTCTGCTTCGGCTGTTTCCAAAGATTTGACTGCTTCTATATCTTCCTTGCTCTTTTCAAAAATGCTCTTTTCCAGTGTCTTGAGGAATTCATCTGGAGACATATGAGACAGCTCGTCCATGTCTGGCAAATCTGCCAAATCCTCAAAACTCAGATTCTTATCCAACTTAGACTTGGTAACAAAAACATCCACCTTATCTGGCTTAGGCGTCACACGGAAGCTCAGCATACCGCTGTCCAAAAAATTATCCGGCATTTCCAACTCATCTAAGATGGTATAGAAAAATTCTTCCGTCTTTTCTTGGGGGACCAAGAAATCGGCCATTTCCATGCCGCGCTTTTCCAAATCTTCCAGCTGAATCGTGATTTT

>c183_g126

TGACCACTCGCACACCACGAAGATTTTCCTTAGCAATGGCATTGATCTTATCCAAAAGAGTTTGGAATTTCTTAAAACGAGGGCCCATCATCCCCATCATGACAGCCGTTAGACCAGCTATGAGCACAATCAGCAAGAAAATAATCCACCAGAGAGAGGGCATGGTCACAATGGCCAAGATGACTGATCCGATAAAAAGGATGGGCAAGCGAAGAAGAATTTGAAAGAGCATCATGGTCACATTTAGAATCTGATTGATGTCATTCGTCATCCGCACGACTAGATTTCCAGCGTTGAATTCCTCGATATTGGCATAAGAAAAGGTCTGGATCTTGCGGAAAGTCTCCTCACGGAGATCTGATGAGACAGCCTGGGCAATGTAAGCCGCCAAGGTCACATTAATCCCTCCTGCCACCAGTCCGACAAAACCGATGCCAATCAGCCAACCGCCCATACTGTAGATGGTCTGGCTATTTTGCTCCTGCAGAGCATCTAGGATGTGTTTTAAAAACCAGGGCAGTAAAAGAGAGCTGGCAATCATCAGCGAGGTCATCAAAAACGAGCTCAGGGCATACCATTTATAGCGTAATAGACTTTTAAAAAACATGCTTCCTCCTACTTTTTTCATATTTGACTTAGTTTCCT

>c183_g127

AAAGCTTATTTTTTAGTTCTCTTCGTCCGGTGTAAGCACCATCGGAATAATAATGGGTTCGCGCTCGGTACTTTCGTAGAGGAAAGGACGCAAGGCGTTGACGATAGCGCCGCTGACAGATTGAACGCTGGCATCTTTATTCTTGAGTGCAATGCGAATAGCATTGAAGAGGATGCGCTGGCTTTGGCGAATGAGGTCACCTGATTCGCGCATGTAGATAAAGCCGCGGCTGAGAATATCTGGACCGGCTAAAATCATCTTGGACTCAAAATCAACCGTCGCAACAGCCAGAACCACACCGTCCTCTGACAGGTCCTTACGGTCGCGCAGGACTGCAGCTCCGATTTCACCAATGCGGTTCCCATCAACATAAATATCCTGAGCGTTGAAGCTGCCGGCAATACGAGCTGAGTTAGCTGTCAGAGCCAGCACATCACCATTGCTCATGATGAAGATATTGTCTTTTGGTACTCCAGTATCAACGGCTAGACCTGCATGGACTTTTTGCATACGGTATTCACCATGGACTGGCATGAAATATTTAGGCTTGATTAAGCGGAGCATGAGTTTCTGCTCTTGTTGGCCACCGTGTCCAGAGGTGTGGATGTTGTTAATCTTACCGTGAATCACTTCTACACCAGCCTCGGAAATGATGTTAATCAGCTTGTTAACGCTGGTCGTATTGCCAGGGATAGGACTGGATGAGAAGATGACTGTATCTCCAGGTTGCAGCTGAACCTGACGGTGAGTACCATTGGCAATCCGAGACAGGGCTGCCATCGGCTCACCCTGACTTCCGGTACACATAATCAGTACTTCGCCAGCTGGATAGTCCTTGAGCTCATTTGGCTCGATGAAGGTATCTTTGGGAACCTTGATATAGCCCAGCTCAATCCCGTTGACAATAGCCTTTTCCATGGATCGGCCAAAGACCGCAATCTTACGGCCGGTCTTAACAGCAGCATCTGCTGCCTGCTGGAGCCGGAAGATGTTTGAAGCGAAGGAGGCGAAAATAATCCGTCCGTGGATGCCTTCAATAATCTTCATAATAGACTGTCCAACCACTTTTTCGGAATTGGTAAAGGTCGGCACTTCAGCATTGGTCGAGTCAGATAGGAGACAGAGAACGCCTTCCTCACCCAGAGCAGCCATACGGTGCAAGTCTGCCGGCTCACCGACTGGTGTAAAGTCAAACTTGAAGTCACCGGTACAGACAATCTTACCCTGCGGCGTATGGATGACAATTCCCAAAGGCTCTGGAATAGAGTGGGTTGTCCGGAAGAAAGTAGCTTTCAGATGCTTGAACTGCAGTTCTGTGTGTTGATTGATTTCATAGAGCTTGGCATCGCGCAAAAGTCCATGCTCTTCTAGCTTGCCGCGAATGAGGGCCAGAGCCAGAGGACCCGCATAGATAGGAACATTGGCTTGCTTGAGCAGGAAAGGAATACCGCCGATATGGTCCTCGTGCCCGTGGGTGATGAGGACAGCTTTTACGCGGTCAATATTTTCCACGATATAAGAGTAGTCAGGAATGACATAGTCAATTCCTAGCAGGTCGTCTTCTGGGAATTTGATACCGGCATCCACGATGATGATTTCATTTTGGTATTCAATCCCGTAGGTGTTTTTACCGATTTCGCCTAACCCACCAATCGCAAAAACGCCAACTTCTTTGGGTTTTAAGGTATATGCCATGGGTTAGAACTCCGTCAATTCAAAAGTGCCTGACTCTTTTTCGTATTCGAGGTGTTTGTCAGACAAGAGCTCGATAAATTCAATATTGTAAGGGGTATTTTCTTCGATGAGCTTACGAGCTTGAATGCGGCCTTCCAATTCATTAGCCGCTTCAATCTCTAAATAAAGGGCACGTGTCTTTTCACGGCGCGGGCTGCGCTCTTTTGTTTCCTGATAAAAAACTTTGTAAATCATAAGATTCCTTTCTGTGATAAGGTCTAAAAAATAAAAGAAGGCAGGCGTTAGCGCATTTCTTCACATGATAAATTAAAAATGATAAATGGAAAGTGAGCGATTGCTGTGACTTCTGATAGGACTTACTCAAATGAAGAGGCTAAAATGCTGTTTTAGCCTTGTATCTT

>c183_g128

CTTGTCTTTGAAGTATGGACCAAGACCTCTAAGTCCGACTTTTTATCTCCACTCTTAACAAGATTATAAACATTTTTACGATTAGAAGCTGCATCTCCCTGAATCAGTCGATTAAAGTGTTCCAGATTTTCTACCGGAATAAAGGCTGCAAAAATAATAGGTAGGTTAATACGAATTTGCCTACGCAGCCGATGTTTTTCACGATCATTATCAAAAAATAGGCCATCCCGGATATTGGACATTCCCAGCATCATCAAATAGATTCCCAAAATCAAAAACTGCAAGTGTCCGTCAGTCGCAGGCGAAAGAATACTGGTCAGCCCAATTCCACCATAGAGAACGGTATCAAATAGATAGCGCAAGCCGCCCTTGACATGATTTTGCCGATAAAGGAGATAGGTCACTCCGTAAATACCGGCAGTCAAGAGCTGGTAGCTACCCAAACTGATAATCACAATATTGACAGGCACATCACTGATTCGGTTAAGCCAGCTGATACCCATAGCGACTGCAATCTGAAGCAGACTATGCCAGACTGCCCCTTCTTTCTTATGCGTAAAAACATGAAGAACAATATTTACCAAGCCAACAACTGTCAGATAAAGCGAAATAA

>c183_g129

TCAACTTGCATCTTCCTCTACAAATTGACTATTATAGAGGTCCGCATAGAAGCCATTCTGATCCATGAGTTCATCATGGTTGCCCTGCTCGATGATATTTCCATCGCGCATGACCAAGATAAGATCCGCATTACGGATAGTAGACAGGCGGTGGGCAATGACAAAGGAAGTCCGGCCCTCCATGAGTTTGTCCATAGCCTTTTGAATCAGCTCCTCCGTCCGCGTATCGACTGACGAAGTTGCCTCATCCAAGATCAAGAGCGGCGCATCTTTCAACAGAGCACGAGCGATGGTCAACAGCTGCTTCTGACCAACCGATAAGTTGACCGTGTCATCCAGCACTGTATCATAGCCCTGCGGCAGAGTCATGATAAAGTGATGAACACCGACCGCCTTCGCTGCAGCAACAACTGCTTCATCCGTGACATGCTCCTGATTGTAAATCAAGTTCTCTCGGATAGTTCCTTCAAAGAGCCAGGTATCCTGCAAGACCATTGAAAAGGCATCGTGAACCTCAGAGCGTTTCATATCCTTAATATCTATACCATCAATGCTGATACGCCCTTTATTAATTTCATAGAACTTCATAAGGAGATTAACGATGGTCGTCTTACCAGCACCCGTCGGGCCGACAATGGCAATCTTCTGTCCGGCTTTGGCCTGGGCTGAGAAATCATGAATGATTGTCTGGTCTGGCTTGTAACCGAAGAAGACTTCTTCAAAGGCAACATCTCCCTTGACAGCAGCAAGCTGCTGCTCTTTATGCTGGTCGTCTTCCATCTCCGGCTCAGCCAGAAAGTCAAGGACACGAACCAGTGCTGCACCAGCCTGCTGCAAGACAGTGATACCTTGGGCAATCTGTGACAATGGCTGAGAGAAGGTCCGCACATAAACCATAAAGGCGACGATAATTCCGATGCTAATAGAGCCATTAATCGCCAGAGCAGCACCTACCAAAATAACCAGCACATAGGCGAAATTCCCCGTAAAGAACATGAGCGGCATCATCATGCCAGAGATAAATTGAGACTTCCAAATACTGTTATAGAGATTGTTATTGAGAACTGCAAAGCGCTCCTTAGACTGCTCAATAGCATTATAGCTGGTAACGACATTATGACCGGAGTACATCTCCTCTACATAACCGTTGACAGCCGCCAGATCGTTTTGCTGGGCGGTGAAATAACCCTGAGACTTGCCCATGATGAGCCCGACAAAGACAAAACCGAGTATAGTAGCCAAGATAGTTACCAAGGCCAAGATCCAGTTCATAAAGAACATGGTCAAGACCACAGCCACCAAGAGCAGGCTAGCAGAGATAACATTTCCCAGACTCTGGTTGAGGGATTGGCCGACCGTATCCACGTCATTGGTCACGCGCGACAGAGTATCCCCCTGAGAATGACCATCGAAATAGCCCAGCGGCAGCTTATTAATCTTCTCTGCAATGGCCGTCCGCAGTCGCTTAGAGAAATACTGGATAACCGTACTGATGATAAAGGACTGACCATAGTTGAGCAGGGCTCCGACTGCATAGAGCACGGTCAGCAGAAGGGCAATCTGAGACACAGCTTTCAAATCAATGCTGGTAGCCAGACCATCAGAAATGAGATTGGTAATTTCTTTTAATTTAATTGGCCCATAGACCGTGATAATGTTTGACAAGACAGCTCCCACAAATGCCAAAGCAAGAGGAAGCTGGAAGCCTTTGAGATAAGGCCGTATCTGGCTAAATAATGAAGGCTTCTTATTTTCCATTTTCTAATTCCTCCTTGGATAGTTGTGAGTATGCAATTTCTTGATAGACTTCGTTGCTGGCCAAAAGCTCTCGGTGAGTACCTTGACCGACAACCTTACCAGCATCCAAGACCAAGATTTGATCCGCATCCATGATAGTAGAAATCCGCTGCGCCACGATTAGCTTGGTCATATCCTGCGTCCGCTCAGCCAGTTCCTTACGCAAAATGCGGTCTGTCTTATAGTCTAGGGCTGAGAAGGAGTCGTCAAAGATAAGGATTTCTGGCTTACGAGCCAAAGCTCTGGCAATAGCCAAACGTTGACGCTGACCACCAGAGAAGTTGGTCCCGCTTTGAGCCACTTCTGACTTGAGCCCTTTTTCCTTCTCTTGGACAAAGGGCTTAGCCTGTGCTAGATCAAGAGCATCCCACATTTTCTGCTCATCCAGCGGGCTTTCTTTGCTTTGACCGAAGTCCAGATTGCTCTCAATATCGCCAGAGAAAAGCACAGCCTTTTGCGGGATGTAGCCGACCTTATTATGCAGATCTTCCAGCTGGTAATCCTGAACATTGACACCATCTACCAGAATCTCTCCTTCAGTCACATCGTAGAAACGAGGGATGAGATTGACAAGTGTGGATTTCCCTGAACCAGTTGAGCCGATAAAGGCCACAGTATCACCAGCCTGCGCTGTAAAGCTGACATGCTCAATGACCGCTTCAGAATTCTTAGCATAGCGGAAGGACACATCTCGAAAGACCACTTCTCCCTTGCTATCCGATGCTGCCTTAGGATGTTCTGGACTGGTGATAGAAGAATGCAGGTCCAGTACTTCATTAATCCGGCCAGCCGATACAATGGTCCGAGGCAGGACGATGAAGAGGGCACCCATGAGCAAGAACCCAATCACAATCTGCATAGCATAGGACATAAAGACGACCATATCACTGAAGAGTGGGAGACGCTCCTGCAGTCCAGCCTCCTGAATCAAATAAGCACCAATCCAGTAAATAGCCAGTGTCAATCCGCTGGAAATCCCCATCATAACTGGATTCATCATAGCCATCAGACGACCGATGAAAAGATTGAGCCGGGTTACTTCATCATTGGCTGCCGCAAATTTCTCATCCTGATAATCCTCAGCATTGTAAGCCCGTACCACGCGAATCCCAGTCAGACTCTCTCTAGTGACGCTGTTTAGCTTATCGACCAAGCGCTGCGCAACTGACTGCTTGGGAAAGGCCAATGTCACCAAAACTACCGTCAGCAGGATATTAACAAGAACGGCAATGAGAACAGCTGTCAGCCAGTTCTCAGACTTACCGATAATCTTGGTCATGGCCCAAATCGCCATAATCGGTCCTCTGGTCACCACCTGCAGTCCCATGGTAATTAAAGTTTGCACCTGAGTAATGTCATTCGTTGTCCGAGTCAGCAAACTAGGGATTGAAAATTTCTTAATCTCTGTCTGTGAATAGTCTAGCACACGGTTGAAAATATCGCTCCGCAAACTCTGGGTAAAGCTGGCTGCTATACGAGCGGCAAAAAAGCCAACCACAATAGCGGACAGCAAGCTAGCAAAAGACAGGCCGATCATTTTCAGACCTGGCTCCCACAAATCTGCCAAACCAGTCCCCGGCGTTTGCAATAGCTCTGTAATCGTCGAAATATAGGTTGGAACTTCTAATTCCAGATATACCGTTAGGAAGGTGAAGAGCACACTGAGAACAATCATGCTCACTTCCCTTACCGTCAGCCGTTTAAATAACTTAAACATCTTTCCCTCCATTTTCTAAACTTTCGATATTCTGATAAAACTTGGCCATGACTTGAGAAAAAATCAGCAGTTCCTGCTCAGAAACACCATTCAAAATAGAAAGGTTCATTTCATCAAAAAAGTCACGGATTTTTTTCATTCTTTCCTGCGACTGCGGAGTGAGACGGATATATTTAGCCCGCTTGTCTGTCTTGCCCATTTCCAGCTTGATAAAGCCATTCTTCTCCATCCTTTTCATTAAGTTAGAGGCCACCGATTTGGAAATATCCAACTCCTGCTCAATATCCTTGATAAGGGTATCCTTCCCCTCTTCCATACGACAGGCGACAATATGCAAGACCTGCCCCTGAGGCCCAGCCATGGACTCAATCCCTTGCTCCTTGGCAAGCTTTTCCGCTATGAGATGAATCTTTCGTCCAAAACGCTTCA

>c183_g13

ATCCTTTTCTTTTTTTTGAAAGCGATTTATGTTATAATAACAGTTGTGTTTATTTTATACAAAAAATAATGATATGACAAGGTAAGTGTATGACAATAGAATGGGAAGAGTTTTTAGACCCCTATATTCAAGCGGTTGGAGAGCTGAAAATTAAGTTGCGTGGAGTGCGAAAGCAATACCGCAAGCAGCAACGTCATTCTCCGATTGAGTTTGTGACCGGACGTGTCAAGCCTATTGAGAGCATTAAAGAAAAGATGATTTTGCGAGGCATTCGCGAGGAGAATATTGAGCAAGAGATGCAGGATATCGCTGGCTTACGGGTCATGGTCCAGTTTGTCGATGATGTTGATGAAGTTTTAGAAGTCCTGCGAAACCGGACTGATATGCGTATTGTCCAGGAACGGGATTACATCAAAAACAAAAAAGCCAGTGGCTATCGGAGTTACCATGTGATTGTAGAATATCCAGTTGATACGATTAATGGTCATAGACTTATTCTGGCTGAGATTCAGATTCGCACCCTTTCGATGAATTTTTGGGCTACGATTGAGCATTCTTTGAATTACAAGTATAAAGGAGAATTTCCCGAGGAAATCAAATGCCGCTTGGAAACGACAGCTAATCTTGCCTATCAGCTGGATGAGGAGATGGGAGAAATCCGTGATGCTATCCAGGAGGCGCAGGCTCTCTTTGATCCTCTTCACCGCAAGTTGAATGACGGTGTGGGAAATAGTGATGATACAGATGAAGAATACAGATAAAAAAATAGCGATTATCCGCAATCGGAAAAGACAGAGCGAACAAGTTTATCAAGATTTGAAGCAGAAGCTGAAACAGAATGGCTTTATTTTAACTCCTAAGAATCCCGACATCGTGATTTCGGTGGGTGGTGATGGCATGCTGCTATCAGCTTTTCATATGTATGAAGAACAGCTGGATCGGGTCCGCTTTGTCGGTGTGCATACAGGGCATCTCGGCTTCTATACGGATTACCGTGATTTTGAATTAGATAAACTGGTAGAAAATCTCAAATTAGACACTGGTGCTCAGGTTTCTTATCCAATTTTAAATGTCAAAATTACTTTTGAAAATGGTGATACACGCACCATTCGTGCTTTGAACGAAGCAACAATCAAGCGTTCAGATCGGACTATGGTAGCTGATGTCATTATCAATCGGGTTCATTTCGAGCGTTTCCGAGGAGATGGGATTTCCGTTTCTACTCCGACAGGCAGTACAGCCTATAACAAATCTCTAGGTGGAGCGGTATTGCATCCGACAATCGAAGCCTTGCAGGTGACAGAGATTGCCAGTCTAAACAATCGGGTTTATCGGACGTTGGGCTCGTCGGTCATTGTTCCTAAAAAGGATAAGATTGAGTTAGTGCCGACCCGCAGTGATTATCACACTATTGCGGTCGATAATCAGACTTTCTCTTTCAAAAACATTGTCCGCATTGAGTATCAGATTGACAATCACAAGATACATTTTGTGGCTTCACCAAGCCATACCAGCTTTTGGAATCGTGTCAGAGACTCCTTTATTGGAGAGTGCAAGGAATGAGGTTTGAGTTTATTGCTGATGAGCATGTCAAGGTAAAGACCTTTTTGAAGCGACACGAAGTTTCCAAAGGCCTCTTGGCAAAGATAAAATTTTCCGGCGGAAACATTCTTGTCAACCATCAGCCTCAGAACGCTATTTATCTCTTGGATATTGGCGATAAAGTGACGATTGATATCCCTTCAGAAAAAGGATTTGAGAGTCTGAAAGCTGTTGATAAGGACTTGTCTGTCGTCTACGAGGATGAGCATTTCTTGGTTTTGGATAAGCCGGCAGGAGTGGCCAGTATTCCCAGTGTTAATCATTCCAATACTATGGCAAACTTTGTTAAGGCTTACTATATTCGTCATGCCTATGAAAACCAGCAGGTGCATATCGTGACACGTCTGGATAAGGATACAAGTGGGCTCATGCTCTTTGCCAAGCATGGCTATGCTCATGCCAGATTGGACAAACAGTTGCAGAAGAAGCTGATAGAAAAGCGCTATTACGCTCTAGTTCGGGGGACTGGTGACTTAGAGGAGCAAGGTGAGATTATTGCTCCGATTGGCCGCAACCCTGAGAGTATCATTACGAGGCGCGTGACAGAGGATGGTAAGTACGCCCATACCAGTTACAAAGTCATAGAGCGATTTGGAGATGTCTATCTGGTGGATATTCATCTGCATACTGGGCGGACTCATCAAATCCGCGTTCATTTTTCACACATTGGCTTTCCGCTTTTGGGTGACGACCTTTATGAAGGAAGTTTGGAACACGGAATAGAGCGTCAGGCTTTACATTGTCATTCTTTGAAATTTTATAATCCTTTTAGTGGTCAGGAAGTTGAGCGTGCCAGTCCTTTGCCAGAAGATTTTAAACAAGTTATTGAGAAATTAAAAGAATAAGAGATAAAGGAGTTTTACGAAACATGAAAATTTTTGACTCAATTCGCGAAGCCTTGAAAGATAAGGAAGTAAAAATTGTCTTGCCAGAGGGTGAAGAGCCACGGATTTTGCAAGCAACTAAACGTTTGGTGAAAGAAACCGATATTACACCAGTTCTTTTGGGGAATCCTGATAAAATCCGTATCTATCTAGAAATTGAGGGAGTAAAAGAAGGATATCAAGTTATTGATCCTTCAAACTGTTCCTGCTTTGAGGAGTTGGTAGAAGCATTCGTTGAGCGCCGTAAAGGGAAAATCACTGCAGATGAAGCACGTCAGTTGCTCAAGGAAGATGTCAACTACTTTGGGGTCATGTTGGTTTATCTTGGTAAGGTTCAAGGGATGGTGTCTGGCGCGATTCACTCCACAGCAGCGACTGTTCGGCCGGCTCTTCAAATCATCAAGACCCTGCCTTGGGTTTCTCGTACATCAGGAGCCTTCCTCATGGTGCGTGATGACGAACGCTACATTTTCAGTGACTGTGCCATTAATATCGATCCAGACGCCAATATTTTGGCGGAGATTGCTGTTAACTCAGCTTTAACAGCACAAATTTTCGGTATCGATCCAAAAGTTGCCATGCTCAGCTATTCGACCAAGGGCTCTGGATTCGGTGAAAAGGTTGATAAGGTAGTGGAAGCGACTAAGCTGGCTCGGGAAATGCGTCCAGACTTGGTTATCGATGGTGAATTGCAGTTTGATGCAGCTTTCGTCCCTGCAACAGCAGAGCTGAAAGCACCTGGCAGCCCTGTGGCCGGTCAAGCAACTGTCTTTGTCTTCCCAAGTATCGAAGCTGGGAATATCAGTTACAAAATGGCAGAGCGTTTAGGTGGCTTTTCAGCGGTAGGACCTATCCTGCAGGGACTTAACCACCCAGTTAATGACCTTTCCCGAGGCTGTAATGCGGATGATGTTTATAAGCTGACTTTGATTACAGCTAGT

>c183_g130

TTTTAATGCAAGACAGAAACTTAGTAAATGTTAATCTGACTAATGAAATGAAGACTAGTTTCATTGATTACGCGATGAGCGTTATCGTGGCTCGGGCACTTCCAGATGTTCGTGATGGTCTAAAGCCAGTTCACCGTCGGATTCTCTATGGTATGAATGAACTGGGCGTGACGCCTGAAAAACCTCACAAGAAATCAGCTCGTATCACAGGGGATGTCATGGGTAAGTATCACCCGCATGGTGACTCCTCTATTTATGAAGCGATGGTGCGGATGGCTCAATGGTGGAGCTATCGCTATATGCTCGTAGATGGCCATGGAAACTTCGGTTCTATGGACGGAGACGGAGCTGCTGCTCAACGTTATACAGAAGCACGTATGAGCAAGATTGCTCTGGAGATGCTTCGCGATATTAATAAAAACACTGTTGATTACATTGACAACTATGATGCCAGCGAGAGAGAGCCTGTAGTTCTTCCTGCTCGCTTCCCTAACTTGCTTGTAAACGGTGCGACAGGGATTGCAGTTGGGATGGCGACAAATATTCCACCGCATAATCTTGGTGAGTCTATTGATGCAGTTAAGCTTGTCATGGACAATCCGGATGCAACAACTCGCGATATCATGGAAGTCCTTCCTGGACCAGATTTTCCTACAGGTGCACTGGTTATGGGTAAGTCTGGTATTCACCGGGCTTATGAAACAGGGAAGGGTTCAATTGTTCTTCGTTCTCGCACTGAAATAGAAGAGATGAAAAATGGCCGTGAGCGAATTGTCGTAACCGAGTTTCCATACATGGTTAATAAGACTAAGGTCCATGAGCATATTGTTCGTCTGGTGCAGGAAAAACGCATTGACGGTATCACTGCTGTTCGTGATGAGTCCAACCGTGAAGGGGTCCGTTTTGTCATTGAGGTTCGCCGTGATGCCTCTGCTCATGTCATTTTAAATAACCTTTTCAAGCTGACTCAGATGCAGACCAATTTCAGCTTCAATATGCTGGCTATTCAAAATGGTGTGCCGAAGATTCTATCTCTGCGTGAGATTTTGTTAGCCTACATTGAGCATCAGAAAGAAGTGGTGACTCGACGGACGGTCTTCGATAAAGAAAAGGCAGAAGCGCGAGCTCATATCTTGGCTGGTTTGCTAATTGCATTGGATCATATTGATGAAGTGATTCGGATTATCCGTAATAGCGAAACGGACGCAGAAGCACAGGCTGAATTGATGGCTAAGTTTGAGCTATCTGAGCGCCAGAGTCAGGCTATCCTCGATATGCGTCTGCGCCGTCTGACTGGCTTGGAACGTGATAAGATTCAGTCAGAATACGATGAGCTGATTGCCTTGATTGCAGACCTGGCTGATATTTTGGCTAAGCCAGAGCGCGTTATCGCTATTATCAAGGAAGAGTTAGATGAGGTCAAGCGTAAATTTGCGGATGACCGCCGTACTGAGCTGATGGTGGGAGAAGTTCTTTCTCTTGAAGATGAAGATTTGATTGAGGAAGCGGATGTTTTGATTACCCTGTCCAATAAAGGCTATATCAAGCGTCTGAATCAGGCTGAATTTACCGCTCAGAAACGTGGTGGTCGAGGTGTTCAAGGAACTGGTGTCAAAGATGATGACTTCGTCAAAGAGCTGGTTTCAACAAGTACCCACGATAGACTGCTCTTCTTTACAAATAAAGGCCGTGTTTATCGTCTCAAAGGATATGAAATCCCTGAGTATGGTCGGACAGCTAAGGGCTTGCCAGCGGTCAATCTCTTGAAGCTTGATGAAGGAGAGACTATTCAGACTATTATTAATGTCCAGCAAGACCGCAGTGATGATTCCTACCTCTTCTTTACTACTCGTCATGGGGTGGTCAAACGGACCAGTGTAACAGAATTTGCTAATATTCGTCAGAATGGTCTCAAAGCTTTGAATTTGAAGGATGAAGACGAGTTAATCAATGTCTTTCTGACGGACGGCGCTGCAGATGTTATCATTGGTACTAAGTTTGGTTATTCTGTCCGCTTCAATGAGACAGCTGTCCGGAGCATGGGCCGTATAGCGACTGGTGTTCGCGGAGTTAATCTTCGAGATGGTGACCAGGTCGTTGGAGCTGGTGTGATTGCTGAGGGAGACGAAGTGCTTGTCATCACCGAAAAAGGCTATGGTAAGCGAACTCTTGCTAGTGAGTATCCAACCAAGGGCCGTGGTGGTAAAGGGATTAAAACAGCCAATATCACTGATAAGAATGGACCTCTCGCTGGTCTGATGACTGTTACTGGAGAGGAAGATTTGATGATTATCACTAATACAGGCGTCATCATTCGGACCAGTGTGGCTAATATTTCTCAGACAGGCCGCTCAACTATGGGTGTTAAGGTCATGCGTCTGGACCAAAATGCACAAATTGTCACCTTTACAAGCGTCGAAGCAGACGATAAAGAAGATGTAGCAGAGGAAGAAAACGAATCGTAAAAGAGGACCTCTATATGGTACAAGGAAAAAGAAGTCGAAAAAGAAAACAAAAAAGCAAAAGAAATATTTTTATCAATATTGTTGCGACATTATTAATTTTTGTGGCCCTAGGCTTAATCTTTAATGCGCAAATCCGAAACATGATTATGGTTTGGCATACCAACCAATATCAGGTCAGCAAGGTTTCCAAAGACTCTATTAATAAAAATAAAAATGCTGAGACTAGCTTTGATTTTAATAAGGTAGAGTCGCTTTCTACAGAAGCAGTTATCAATGCTCAGTGGAAAGCCCAGAAGCTGCCTGTTATCGGAGGTATCTCAATTCCAGAAGTATCCATGAATTTGCCGATCTTCAAAGGGCTGGATAATGCTGGATTGTATTATGGTGCTGGTACGATGAAAGAGACTCAGCAGATGGGACAGGGAAATTACGCCTTGGCTAGCCACCACGTCTTTGGCATCACAGGGGCTAGTAACATGCTCTTTTCCCCACTGGATCGTGCTAAGGCTGGTATGAAAATCTATATCACCGATAAAGAGCAAGTCTATACTTATGTAATCACCAGTGTTGAAACGGTAACTCCAGATCGAACGGATCTGATTGAGGATACTGAAGGCGTTACAGAGATTACTTTAGTTACTTGTGAGGATGCTGCGGCAACCAATCGAACGATTGTCAAGGGAACCTTAGAAGGCTCTGTCGAGTATGACAAGGCTCCTAAGGATGTTCTTGAATCTTTCAGTAAGTCCTATAATCAAATGCA

>c183_g131

AAATTCAGACTCGGAATTTCATCATAAGGAGAAGATATGGATAAACAACGAGTTGCTGTGATTGGTCCTGGTTCTTGGGGAACGGCCTTATCACAAGTGCTAAATGACAACGGACATGAGGTTCGTATTTGGGGAAATATCGCTGAACAAATCAACGAAATCAACGACGAACACACCAATAAACGCTATTTCAAAGATATTGTACTGGATGAAAAGATCAAGGCCTATCATGACCTAGAAGAAGCTTTAAAAGATGCAGATGCCGTGCTTTTTGTCGTGCCGACCAAGGTAACCCGTCTGGTAGCCAAACAAGTAGCTCAAGCTCTGGACCACAAGGTGAAAATCATGCACGCCTCCAAGGGACTGGAGCCCAATACCCACGAAAGGATTTCAACTATCCTAGAGGAAGAAATCCCTGCAGAGCTACGCAGCGAGATTGTCGTTGTCTCAGGTCCCAGCCATGCTGAGGAAACCATTGTACGCGATATTACCCTGATTACCGCTGCTTCCAAGGACCTGGAAACAGCTAAGTATGTGCAGGAGCTCTTCAGCAATCACTACTTCCGCCTCTACACCAACACCGATGTGATCGGAGTGGAGACTGCAGGCGCTCTGAAAAACATCATCGCTGTCGGAGCCGGTGCTCTCCACGGATTAGGCTATGGTGACAATGCCAAGGCTGCCATCATTACACGCGGTCTGGCTGAAATCACGCGTCTAGGTGTCAAGTTAGGAGCAAATCCTCTGACTTACAGCGGCTTATCTGGTGTCGGTGACCTGATTGTCACAGGTACCTCTGTTCACTCCCGTAACTGGCGGGCCGGCAATGCTCTAGGACGCGGCGAGAAATTAGCTGATATTGAAGCCAATATGGGCATGGTTATTGAAGGAATTTCTACCACCAAGGCTGCCTATGAACTGGCCCAAGAGCTGGATGTTTATATGCCGATCACCCAGGCTATTTATAAGGTTATTTATCAAAACTGTAATATCAAAGAAGCCATTTATGAAATCATGAACAATGAATTTAAGGCTGAGAACGAATGGACTTCGTTCTAATAGAAAGGAAATAATTATGTCAAAAGTTAAGAAAGCCGTCATCCCTGCAGCTGGTCTCGGAACCCGCTTCCTGCCAGCTACCAAAGCACTAGCCAAAGAAATGTTGCCGATCGTTGACAAGCCAACCATCCAGTTTATCGTCGAGGAAGCTCTCAAATCTGGAATTGAAGATATTCTAGTTGTCACAGGTAAGTCAAAACGCTCCATCGAAGACCACTTTGACTCAAACTTTGAATTAGAATACAATCTCAAAGAAAAGGGTAAAGACGATTTGCTCAAATTAGTTGATGAAACCACTGGTATCGGTCTGCACTTCATCCGTCAAAGCCATCCGCGCGGACTGGGAGATGCTGTCCTTCAGGCCAAGGCTTTCGTAGGAAACGAGCCTTTCGTTGTCATGCTGGGCGATGATCTCATGGACATCACCAATGATAAGGCTGTGCCGCTGACCAAGCAATTGATTGATGATTACGAGGCTACTCACGCCTCTACCATCGCTGTCATGCAGGTCCCTCATGATGAAGTTTCCTCTTATGGTGTGATTGCCCCTCAAGGCGAAGGTGTCAAAGGTCTTTACAGCGTGGAAACCTTCGTCGAAAAACCAAAACCTGAGGATGCACCGAGTGATTTGGCTATTATCGGCCGCTACCTGCTGACACCAGAAATTTTTGAAATCTTGGAAAAGCAAGAGCCAGGTGCTGGAAATGAGATTCAGCTGACCGATGCAATTGATACTCTCAACAAGACGCAGCGCGTCTTCGCTCGCGAATTCAAGGGCGATCGCTATGATGTCGGTGACAAGTTCGGCTTCATGAAGACTTCCATCGACTACGCCCTCAAGCATCCGCAGGTCAAAGACTCCTTGAAGCAGTATATCATTGACTTAGGTCATAAACTAGAGAAAAAACAAGAGAAAAAATAATGGCTTCAAACAACTAACTTTGAAAATAAAATTTTAGCTATAAAAAA

>c183_g132

ATAATATTTTGTGCTATAATGGAAGCAATCAAAATCATGGAGGTTCAGAAATGGCAGAAGCAGGTCATAAATTTTTAGCAAAATTAGGAAAGAAACGCCTTCGTCCCGGTGGTAAGCTGGCAACGGATTGGCTGATTGAGCAGGGGCAGTTTTCCAGCGATAAGAAAGTTCTAGAAGTTGCCTGCAATATGGGGACCACAACAATCGAGCTGGCTAAAAAATATGGCTGCCAGATTACTGCAGTTGATTTGGATAAGGCTGCGCTGGCTCAGGCTAAGCTTAATGGAGACAAAGCTGGTGTTAGTGAACTCGTCACATTTGAGCAGGCTAATGCCATGAAGCTGCCTTATGATGATAATTCGTTTGATATCGTTATCAATGAAGCCATGCTGACCATGCAGACAGATAAGAGGAAGGCCAAATGTATGGATGAGTACTACCGGGTGTTGAAGCCAGGCGGAGTGCTCCTCACCCATGATGTCATGCTCAAACAAAGAGATGAGAATATCCGAGAAGAGCTATCTCGTGCGATTAATGTCAATGTGGGGCCTCTAACCGAGGGATCTTGGATTCAGCTGGCACGTTCTCATGCTTTTGACCGTGTGGACACCTTTGTCGGCGAAATGACCTTGATGAGTCTTCGAGGTATGATTTATGATGAAGGACTTGGAGGAACTCTGAAAATCTGCTTTAATGCCCTTAAAAAAGAGAACTACGGCCAGTTTATGAAGATGTTTAAGATGTTCCAAAAGAATCAAGAAAAACTGGGCTTTATTGCCATGGTTTCTCGTAAATAACTTCTAGTCGCTTTGGAAACTGTAAGGAAATTTTAAAAATTTCTAAAAAACTTGACCCTCCCCTTAGGTCATGGTATATACTAGTTAGGTAAGGAGGTCTATCATGTACCATATCAAAGAAGCTGCGCAGCTTTCAGGAGTCTCTGTCAAAACCCTGCATCATTATGATAAAATCGGACTGTTAGTCCCGGCCAAATCAGAAAATGGCTATCGGACATACAGCCAAGCTGATTTAGAGAGGCTGCAGGTCATCCTTTACTATAAGTATCTGGGTTTTTCTTTGGAGAAAATAGCAGAGTTGCTGAGTCAGGATGAACAGGCTTTGTTGCCACATCTGGTTAGGCAGTTAGAGTATTTGCAGCAGGAAAGAGACCGCTTGGATACCTTGATTTCCACCTTGCAAAAAACCATCCAAGATGAAAAGGGAGAAAGAAAAATGACAATGAAAGAAAAATTCGCAGGTTTTACCTATGAAGACAATCACAAATATCATCAAGAAGCCGTCGAAGAATACGGTCAAGAAGTCATGTCGGAAGCATTGGTTCGTCAAAATGGACGGGAAGAAGAATCTGCCGCAGCCTTCAATCAAGTTTTTCAAAGTCTGGCAGAGAATATGCAGCAAGGCTTGCCGATTGAAGCAGCAGAAAATCAAGAGCAGGCAGCGCGTCTCTTACAAGCCATTCGCACTTATGGTTTTGACTGCTCACTGCAAGTCTTTGCCCACATCGGTCAGGGCTATGTCCACAATCCAGAATTCAAGAAAAATATCGATCAATTTGGACCAGGCACTGCCCAGTACACAGCGGATGTGATTGCAGTTTACGTTCGGACAAATGCATAATAAAAACTGGTGAGAATGTTCTGCACTAATCCCA

>c183_g133

CGCTTCTTAGCCGCTTTGGAAAGGGAAATGTCCTGTACCTTATCATAGAACAGGAACTGCTGAATGAGCTCTCTGTCGCTCATGTCAGGATTCAAGTCAACAACATAGTCCAGCTTGATAGACTTCTTTTCACCCGACTTGAGAGATGTCAATTCAGCCGATTTACAAGTCGTTTCAAAGATAGCATCAAAGAGTACTTCGTAGTCAAAGTCAGCTGGAACCTGAACCTGGACATAACGTCTGGTCGGACTGACTGAAGTAAAGCTCATCTTTTCAAAGAGCATCCAGATGCCTGAAATAGCTAGAGTAAAAACAATTCCCAGTGCTACAAATCCCATCCCTGTTGTAATCCCGATAGCCGTCGCCATAAAGATGGCTAGAAGCTCCTTAGAGCCACCTGCAGCTGACCGGAAGCGAATCAAGCTGAAAGTTCCTGCTACGGCGACACTGGTACCCAGATTTCCATTAACCAAGAAGATGATAATGGAAATAATGGCTGGCAAAAGGGAGAGGGTGACGACAAATTCCTTAGTATAAATGGTTTGGCGCTTATAGACCTTGGCCAAGATAATTCCTAAAACCACACTAGTTGCTAGTGAGAAAATCAATGCTAAAGGATTTATCTTGACTTCCGTAGAGGAGTAGATACTGTTAAATAACTGATTGAGCATAGACTACCTCCTTAGGCCTTGACTGTTTGAGTCAGCCTTTCTTTAGTTTTCAGGTAGGCATTGCCGTATTTTGAGAAGGACTGGTCTTCAAGACCGTATTTGTTCAGAATACCAGCCAGCCACTGAGGATATTGACCAGGAACCTTGATTTCCATAATTACCTTGTCGTCATCCAGCAAGGGCAGTCCATAGCGGCCTGAAGCCAAGTCTGCATCATAATCTCGGTAGCGGATGTTGGAATCAACTGTCACACGTACCTTTTTATCTTCCAATCCTCTCATAGAGTAGCGGTCATAGCTAATCACCATTTTGGGCTTCAAGTCTATATAGCGCTCCTGCAACTGCTCCACTTCTGCCTTGACCCGGTCATCGGAAATAGTATGGTCTGCCACACCATTTACAATATAGTTGGTAACAGAGAGAGGATTCGAAACCAAACGGTATTTGAAGCCTACTTCATCCCGTTTCTTCTTGATTTCTAAGAAAACTTGACTATCATCATTCGGCTGCTCAGCATAAGTCCGCATCCGCATTTTTTCGCGACCACCTTTTCTGGCAATGGAATCCTGGATCATCTGGAACTCATCATTATCAAAATACACATTAGAAATGGTTGATGTCGCATAATCATCTGCGGTTAGGTAAGGTCTCATGTCAGCTTCAAGACGAGCTAGCATTGCTCGATCAAGAATATACTTCGTTTCGATTCGTTGGAAGTTTGTTTGGATTTGCTTTTGTTTCATTTGAATTCTCCTTTGATTCTACAAATGTAGTTTGTGCTTTTAAAAGAAATAGAGCTTCCGCTCTGCATCCTACAAGCGTCTGGATAAGCTTTGCTTTCTTCTCCCTTCCAAGCAGTATTTTTCAAAAACATTCTACAAATGTAGTTTATAAGTATATTCTACAATTGTCGTTTGTGAATGTCAAGAAAAAAGATAAATAATCTTTCAGTTTGTTTTTGGTATTAGGAAACGAAGTACAAACTGTAAGCAAAATCGACCTAAAATAAAGATAAACTGATGAC

>c183_g134

CTACATTTATTTTGTAACCTTTTATTATCCTTGATTTATAAAAGAAAAGAGAATTTTATGATACAATGGTTATATCTTGTTTTAGGGGAAAGAAATGAAACGATTATTCCTATGTTTACTAGTTTTGGTCGGAATGACCGCCAGCAAGGTTGCAGCAGATGATTTCAACATTGCCGCTAAGAGTGCAATGGCTGTGGACGCTACTTCTGGAAAGATTCTCTACGAAAAAGATGCTAATACTCCAATCGAGGTCGGCTCTATCACCAATCTTTTGACCGTCTATCTGGTCTATGAAGCCATTGACAGAGGAGACCTGACTGCCGATACTTATGTTGATATTTCAGACTACGCCTACAATTTGACAGCTAATCCAAATATCAGTAATGTCCCTTTGGAAGCCAAACGTTACAAAGTCAAAGACTTGATTGCCGCTTCCTTGATGTCCAGCTCCAACAGTGCCACGATTGCCTTGGCTGAGAAGGTTGGGGGCAGCGAAGAAAATTTCGTCCAAATGATGAAAGCCAAGCTCAAAGAATGGGGTATCAAAGATGCTACTATTGTGAACTCCACAGGGCTGAATACTCTTCTTCTGGAATACGCAACTGAAGAAACCGACTATACTAGCACAGCTAAAAAAAGCAAGGACACTGAAAATAAATTCAGCGCCTACGACTTGGCTGTTATCAGCAGACATCTGATTATGGACTTCCCTCAAGTGACAGACATCACGTCCAAGTCTACAGCCAAAATTGCGGGGACGAGTCTTGAAAATTATAACTTCATGCTGGAAAACCAGTCCAATTTCCGCTCAGGTGTAGACGGACTCAAAGCCGGCAGTTCGGATAAGGGAGGATCTTCCTTTGTTGCGACGACTACTGAAAATGGTATCCGCATGATTACTGTTGTGCTGGACGTTGAGCAAACCGACGGCGACCCCTACGCACGCTTTGTAGCCACAGCCTCTTTGATGAACTATGTTTCGCAAAACTTCACCCAGACGACTATTGTGGCTGAGGGGGAAGCCTACAATAAAAGCAAGTCAACCGTTATTGATGGCAAGCAAAAGACCGTTCCAGCCGTTGCCAGCAAGGATTTCACCATCATTGAGCGCATCGCCAATCAGGCTGAGCACAAGGTAGAATTTTCAACCAACGAAAAAGGCTTCCAAGCTCCGCTGAAAAAAAATACTGAACTTGGTACCCTGACCTATACCGACCCTGAACCGATCGGCCAAGGTTATCTGGAAAACAAAGCGCCTTCTGTCACGATGGTGGCAGGCCAAGAGGTAGAAAAAAGTATCTTCTTCAAGGTCTGGTGGAATG

>c183_g135

TATCTTAAATGATGTCAAATATTTGACTTGAAGGGTGCTTTAAGTTGTATAATAGTTGCAATAAAGATATCAAAGGAGAAGAAACGATGAAATTGGCAGTTTATACAAAAGCGGGCCAAGTTGGGCTCGCTGACCTTGACCGTCCGCAAATCATTGAGGCAGATGATGCCATTATTCGGATTGTTCGAACCTGTGTCTGTGGATCTGACTTATGGCGCTACCGCAGTCCAGATATTGAAGCAGGCCATCAAAATAGCGGGCATGAAGCGATTGGAATTGTCGAAGAAATCGGAGATACAGTAACGACTGTTAAACCTGGTGACTTCGTTATCGCACCTTTCACTCATGGATGCGGGCAATGTGATGCCTGTCGGGCAGGATTCGACGGCACTTGTGATTCTCATATTGGGAATAACTGGTCAGATGGGGTGCAAGCAGAATATATGCGCTTCGAATTTGCTAACTGGGCACTTATCAAAATTCCGGGCCAGCCCTCTGATTATACAGAAGGTATGCTCAAATCCCTCTTGACACTGGCTGATGTCATGCCAACTGGCTATCATGCTGCACGAGTGGCAGATGTTAAGCCTGGTGATAAGGTGGTCGTTATTGGCGACGGAGCTGTTGGGCAATGCGCTGTGATTGCTGCTAAAATGCGTGGTGCTTCCCAAATCGTTCTCATGAGCCGACACGAAGATCGACAAAAAATGGCCTTGGAATCAGGTGCGACAGCTGTTGTTGCAGAACGTGGCGAAGAAGGAATTGCCAAGGTTCGTGAGATTCTAGGTGGCGGGGCAGATGCAGCTCTAGAATGCGTCGGAACAGAAGCAGCTGTTGATCAAGCTCTAGGAGTCCTGCACAACGGCGGCCGCTTGGGCTTTGTCGGTGTGCCTCACTATAATAACCGAGCTCTTGGTTCTACCTTTGCTCAAAATATTACAGTGGCAGGAGGAGCAGCCTCTGTCACGACTTACGACAAGCAAATCTTGCTCAAGGCAGTTCTTGATGGTGATATTAATCCAGGTCGCGTCTTTACTTCAAGCTACAAACTAGAAGATATTGATCAAGCTTATAGAGACATGGATGAGCGCAAGACCATTAAGTCCATGATTGTCTTTGATTAGGTGTAGAAGAAAA

>c183_g136

GTTTTAGAGCTCGGATTGGAAACTAGCCCTGCAGCTCCTAGTCCTATGAGTAACATAAAAAACGCCCACATAAGTTTCCAACCCATAGAACTATCAGATGAAGACTCCGAATTTTCCTTCCAAAAAGCCTCATTCTCTTCTTTTTTAGCGAGCTCCTCAGCACTAAATCTCGGACGAAGCTCATACTCTTCTGCTTCAGATACAAGTTCTGAGTCTGGACGATTAGTTTCATCCTTAAAATAGAGTGATTCTTTCTTCTTAGATTTCAAGGATTCTAAAGCAAAAATAAGGCGAGATAACTCTCTCTCCCTCAGGTGTAAGAA

>c183_g137

TCTCAAGCGATGATTGACGCCGCTCTTAGTCAGTGGCCGGCTCAGACTATCAGCCAACTGCTGGATGGAATAGTCCGGATGCTGGATACGAAGTTGGGCTACCTCCTGCAAGTCTACAGGTAAACTCTCAATGCCAATATTATCGCTGATTTTGGCAATATTGTTGATGGTTTTCATACTGGCTGTGACCGTGCGAGCGATGTTCGCTGTCTCCGCATTATTGGCACGATTGAGGTCGTTGCGCGCTTCCCGCATGAGCTTAAGAGACTCAAACTCAGCCATGGCTTCCATAGCCCCGATGACAATGAGAAAATCCATGATATCTTCAGCCCGCTGCAGATAAGTAACAGCCCCCTTCTTGCGCTCAATGGTCTTGGCATCCAGCAGAAAACGGCGCATTAAGGCAGCCAAGTCCTCAGCATGATCCAGATAAACGGACAAGATTTCCAGCTGGTACTTGCCTGAGTCTGGCTCTCGCATGCTGCCATTTGAGAGAAAGGCTCCCCGTAGATAGGCTCGACTGGCTTCGTCATCAGTCAAAATAGCCTGGTCAATCCCTGCCTCGATACCAAAAAAGGAGTCAGCCAAGTGCAGGTCAGAAAGGATTTCTTCCACTTTCTGATCTAGAAATACTGTATAAACACGGTTCTTGCGCAAGTTGGTCTTCTGATGGTGGCGGATTTCTGACTTGACCTGATAGAGGTCCGACAGCAACTCATAGAGATGGCGGGCAATTTTGGCATTTTCTGTTGTGACAGACAGGGTCAAACCACTGCTGGCCAAGCCCAAACTGCCAGACATCTTGATCATGGCTGACAGCTCATTTTTATCTCTGCTCGCCAGACTCAGCAGTTCTTCTTTTACTTTTACTGTAAAGCTCATTTACGTACCTGTATAATCTGCATCAATTCATCAACAACCAACTCACCATCATGGAAAGCTCCGCCATTTTCCAAACGCAGAAAATTCGAAGAAATAACGCGCGGAACCTGCTCTTGCAACCCCTGAAAATCATGCTCCACCTGTACTAGGTATTCGTCAAACTGATTACTGTCCATGTACTCGTGAGGAACAGGTTCGATATTAACCAAGACTGTATCTACAAACTTCCTGCCTAAATGACGGTGCAGGACTTGGACGTGGTCGCTGTCCGAAAAATGCTCTGTTTCGCCGCGTTGGGTCATGATATTGCAGACATAGGCCACTTCTGCCTTGGTGTCTAGAAGAGCTTGGCCAATTTCCTCGATAACAAGATTAGGCAAGATAGAGGTAAATAGTGAACCAGGCCCCAGAACCACCATATCGCTCTCCAATATGCTCTCGACCACCTTTTTACTGGCAGCAGGCTTCTTGTCATCATAGGTATTGGTCACATAGACCCGCTCAATCATTCCACTCTTGCTGGTCAGATTGCTTTCACCGACAACTTCCGTCCCATCTGCAAACACAGCATGCAGAGTTAGGGGGGTGTCGCTAGAAGGGTAAATTTTTCCTGTCGTATGGAAGAACTTGGTCAAAAGCTGCATGGCATTATAAGTTGAGCCCTGCATTTCGGAAATGCCGGCAATAATCAGATTGCCCAAAGGATGCCCCGCTAGAACGCCGTCTCCTTCCGCAAAGCGATACTGGAAGACCTTCTCATAAAACTTTGGCATGTCGGACATAGCTACCAGGACATTTCGAAGGTCACCTGGCGGAGTCAGCTGCTGGATGTTTTTTCTCAGCTCACCAGAACTGCCTCCATCATCTGCTACTGTAACAATGGCTGTAATCTCTACGTCCTTTTTACGCAGGCTGTCTAAAATAACTGAAATTCCTGTTCCGCCGCCGATGACTGTAATTCGTGGCTTTCTCATGAGCGGTTCACCGTTTCTTTCCGACGGTTTTTGTCGCGGTGACTGGAATTGACCGGCCAATTTTTAGCCAAATCATCTGCTAAGCGCTGAGCAAAGGCTACACTGCGGTGCTGACCACCCGTACAGCCCACAGCAATAGTCAGGATAGACTTGCCTTCCTTCTGATAGCCCGGCAAGATTGGCTCGATCAAGCCTAGCAGGTTCTTGTAAAATTCTTCTGACTCGGCGTGGTTCATGACATAGTCAAAGACATCCTTGTCCAAGCCTGTCTGATTGCGCAGTTCAGGTTTATAGTAGGGATTGGGCAGAAAGCGCACATCAAAAACCAAGTCAGCATCCAAAGGCAGACCGTATTTGAAACCAAAACTCATGACCTCAATACGGAAACTATGCATGTCAGCCTGATTTGAAAATTGTTCAGAGATGGTCTTTCTGAGCTCCCGTGGTGTCAAATCTGTTGTGTCCACGACATTTTGGCTGAGATTCTTCAGTGGGGCTAAGAGTTCGCGCTCCAATTTAATCCCATCTAAAATCCGTCCATCAGCTGCCAGCGGATGGCTGCGACGCGTTTCCTTGTAACGAGCCACTAACTCCTTATCAGCCGCGTCTAGGAAAAGAATCTTGAAATCAATGTTCTCATTCTGCTCTAATTCATCCAAAACGTTCTGAATTTGCAAAAAGAAGGAACGGCTGCGCATATCAACAACCAGAGCCAATTTATCATTGTCAGTAGTCCCTTCGACCAGCTGCAAGAACTTCGGCACCAGAGTTGGCGGCATGTTATCAATGGTAAAATAACCTAAGTCTTCAAAGGACTGGATGGCTACGGTTTTCCCCGCCCCGCTCATTCCAGTCACAATGACAAGCTGAATTTTCTTCTCAGACATAATAATCCTTTCATCAGATAGAATCTGTCAAAGAGTGGTTTTCCCCCGCTCTTTTTTAGAGAATCTCGGCAATAACCTCAATCTCAATCTTCACATCCTTAGGCAGGCGAGCCACTTCGACTGCTGAACGAGCTGGAAATGCTTCTGTAAAGGCCGTTTTGTAAACTTCATTAAAGGCCACAAAATCATTGATATCACTCAAGAAGCAAGTAGCCTTGACTACATGGTCAAAATCTGTTCCAGCGGCTTCCAAAATGGCTGAAACATTTTTCAGCACCTGCTGGGTCTGCTCTTCAATAGTCGTCCCAATGATTTCTCCAGTCTCAGGAGACAGGGGAATTTGCCCGCTCGCGAACAAAAGATTGCCGACAATTTTTCCTTGAACATAAGGTCCAATCGCTGCTGGTGC

>c183_g138

CAACGATTCAATGGTTTCCGGGGCATATGTCCAAGGCACGGAGACAGGTACAGGAGAATATTAAGTTTGTTGATTTTGTGACGATACTGGTTGATGCCAGACTTCCTTTATCTAGTCAGAATCCTATGCTGACTAAGATTGTGGGGGATAAGCCCAAGCTTTTGATTTTAAACAAGGCTGATTTGGCAGACCCTGTTCGTATCAAGGAATGGCAGAGCTATTTTGAAAGCCAGGAGATTCCGACTTTATCTATTAATTCCAAAGAGCAATCTGCTGTAAAAAAAGTGACAGATGCAGCTAAAAAGCTTATGGCTGACAAATTGGCGCGTCAGAAAGAGAGAGGAATTCGCATCGAAACCCTGCGTACCATGATAATCGGTATTCCCAATGCTGGCAAATCAACCCTCATGAATCGCTTAGCTGGTAAGAAAATCGCTGTTGTAGGGAATAAGCCAGGTGTGACCAAGGGTCAGCAATGGCTCAAATCAAATAAAGACTTGGAAATCTTGGATACACCAGGGATTCTCTGGCCAAAGTTTGAAGATGAGACGGTTGCTCTCAAGCTTGCTCTGACTGGTGCTATTAAGGATAATCTGTTGCCTATGGATGAGGTGACGATTTTTGGTCTCAATTACTTTAAGAAACATTATCCTGAGGAGCTGATAGCACGCTTCAAGCAGCTGGATCTAAGTCAGGAAGCACCTGATATGATTATGGATATGACTCAAAAACTCGGTTTCCGCGATGACTATGACCGTTTTTACAGCCTTTTTGTCAAAGATGTCCGCGATGGCAAGTTAGGTCGTTATTGCTTGGACACGGTTGGAGAACTAGATGGCAACGATTAAAGAAATCCAGCAACGTTTAGAGTTAGTGACTGATTTGGCTGATCCTTTTCTGGCAGAAGCGGCTAATGACCAGCGGAGCGGAGTTCAAAAGGCGATTGAAAAACGTAAAAGAGCCATTCAGGCGGAGTTAGACGAGGATTTACGTCTGGAGCAGATGTTACGGTATGAAAAAGAGCTTTATAAAGCTGACTACCAGGCAATTGCTGGGATTGATGAAGTCGGTCGTGGGCCTCTAGCTGGACCGGTTGTCGCTGCAGCTGTCATCTTACCACCAGAATGTAAAATTAAGGGGCTCAACGATAGTAAAAAAATTCCCAAGAAAAAACATCAAGAAATCTATCAGGCAGTTCTTGATAAAGCTTTGGCAGTCGGTGTTGGCCTGATGAACAATGAGATTATTGATCAAGTCAATATCTACGAAGCGACCAAGCTTGCTATGAAAGAGGCTTTGTCTAAGCTTTCTCTCAAGCCCGATTATCTGCTGATTGATGCCATGAAACTGGATGTTGATATCCCGCAAGAGTCCATCATCAAAGGCGATGCCAATTCTCTGTCTATCGCAGCGGCCAGTATTGTTGCTAAGGTTACTCGGGATAAGCTAATGGCAGACTACGACAAGAAATTTCCTGGCTATGATTTTGCGAAAAATGCTGGTTATGGGACCAGAAGTCATTTGCAGGGCTTGGAACGAAGTGGCGTAACTCCTATTCATCGCAAGACATTTGAACCAATAAAATCCATGTATGAATAGGCACGAAGAGGTGAGGCATGCTTATCAGTAAAATCTTAAACAATAATGTGGTGATTTCTGAAGAAAACCAAGAAGAAGTTATTCTCATGGGACGAGGACTGGCCTTTGGTCGAAAAGTTGGCCAGGAGATTCCAGATGAGCTGATTGAGAAAAAGTATATCTTGTCAGAAAATAGACGCCAGCTTCTGATGGAGTTGCCAGCAGAGGTCATGGAAATGTCGGATAAGATTGTTTCTTTCGCAAGGGAGAAACTGCAGAAGAAGCTCAAAGACAGTGCTTTTCTGGCGATGGCAGATCATATCCACGGAGTCTTGCTGCGCTTGGAAGATGATATTTATCTCAAGAATTTCCTCATGTGGGATATTAAACGCTTTTTTCCTATCGAGTTTGAGGTTGGTCAGTATGCCAAACAGCTTTTGAGCGCTTATGTCAGCAAGGAACTTCCAGATGATGAAGCAGCATTTATGGCGCTGACCTTGGTCAATGCAGAGTTGGAAAATGGCGATGGAACTGCGCGTGATTTGACTATGATGATGGAGGAAATCATGACCATTGTCAAGTACAGTTTGGAAATTTCTTTAGATGAAGAAGATATCTACCTAGAGCGCTTCATGACCCATCTGAAATTTTTCTGTGAGCGAGTCCTGACTGATAATGGCCACCGTGACTTGGAGGACAATGAAATGTTTGACTTACTTAAATGTAAGTATCCCTTGGCTTATGAGACAACTAGGAAGATTGCTGAATTTTTAAAGCAAACCAGAAATTACCAAACATCAGAGGATGAACAACTGTACCTGACTATCCATTTGTCACGCATGAAAAGGAGGATGATATGCAAAGCGAATACGAAAAAATGATTGCTGGAGAGATTTACCGACCACAAGACAGTGAATTAAAAGAATTGGCAGCAAGGTCAAAGGAATTTCAATATCGTTTTAACCAAGAACAAGACAGTGTCAAGCGCGCGGCTATTATCAAAGAATGGTTTGGCAGCACAGGGGAGAATCTTGCTATGAAGCCTGACTTGGTCTGTGATTATGGAATCAATATTCATCTAGGGGAGAATTTTTATTCTAACTGGAATCTGGCTATGTTGGACGTTTGCCCGATTCGCATCGGAGATAATGCTTTGCTTGGTCCAAATTGCCAGCTTCTGACACCTCTTCATCCGCTTGATCCAGTGGAAAGAAATTCCGGTATTGAGTACGGAGCACCAATCACTATTGGTGATAATTTCTGGGCTGGCGGCGGTGTGACGATTCTGCCAGGCGTGACACTAGGAGATAATGTGGTAGTCGGAGCAGGAGCGGTTGTGACCAAGTCTTTTGGTGATAATGTAGTCTTGGCTGGCAATCCTGCTAAAATTATTAAGGAAATTCCTGTTCATAGTGAATAAAAGGTGTGTTAGGCAAGGA

>c183_g139

CGAAATGGATTTATTTTCTTATATTTATGTTATAATATCATTGTGCTTTATTAAATAATATTATATAGTTTAGGAGTAAAACAAATGACGAACTCACAAATAAATAAATGGTTCAACGATATCAGTTCTTTCATCATTGGATATTATGATGAAACGGGAAGTAATTTTACAATTAATAATGAAATTAAAGAAGAATTACAAAATTTAATTGAAATCTGGGACAATGACAATAGAATTTTATATAGAGGCTTCCCTTCTAGATCTGTAAACACAAATTTTTGGAAATATATTTTTACTGTTGGTGAAAAAGGTGCGTACTTTAGGAATAAAGCAAATATTGATGTATCTAAAAAATCTACTAATTATTTTTCCAATCAAACAGATTCTTCTCAAAAAAATTACGAAGACTTACTTTCTGAACTTCAAAGTGAAATTATTCCAAAATATAAGCACAAAGAAATGTTAGGCACTAAAGAATTAAACTTATATAATGAATTTGAGAAATATAGAAAAAATAATGAACTAGATTATACGTTGATGTATTATCTAATTCTTACATGGCTTCATAATATCGGTAAGGCAACCGGTTTAAAGAACGATAGTCCTTTTATTTCGACAACTACTTCTTTAGATGTTGCGCTTGAATTCCAAGATAAGGAATCTGACTCAAAATATGTTTTAGTTATTTTGCTAGTTGAAAATAAAATCAGTGATTATTTTGATACTAACAACTTAAATAAAATATTAAAACAATTAGGTATAAATTGGCATGAAAATATTAATAAAGAGATTATGTTTAAGGACTCGATATTTCCACATTCAATATTGGGTATAATAGAAAAAAAGAAAAATGAAACAAAGCTAATATTGAATCCAAATTTAATAAAATTTTTAAAAGAATCGAAGTGTTGTTATAAAACTAAAGCATCAACCTTGCTAAAGAAAGGAATTCCTATTGATGATAAGAACTTTAATGAAGGTTTAGAATCACTAGGTTATAAAAAAATGACCGAGCAATTGCCTTCTAAAACTGAAAGAACAATTTTTGATGAAGATGGCAAGAAATACGATGTATCTCCTGTAAATCCGCTTAATTCTGTACCCAAACCCTAAATTTTTAAGTTCCAGTTTTAGTGTATTTTCTATTGTCAAAATCAATCTTCCCCTTCGGATATACAAAAAATTATTTTGCAGTTCAATATTTATTAACTACCTCTTTTTACGTAAATTCACATAAACAAAAATCCTTTAATCAAATCAACTACCTGTTCATTTTGCGGCAAGGAAGAATGGTGAGCATCCTTTCCTACGATGACGACCTCTCTGTGTGAAGCAATATTTCCTTTATAAATCAAGCTCCCGGCCTCTACACTGCTTTTATGAACAATTCCATCACCATCTCGGAAAAAGATTCCCAAAATTGACAAATGCTGCAGCTTCTTAGGAAGTTTTCCCTGGTTGGCTACAAAGTCATCTAGCATCGCAACTCGATGATTGAGATTTTTCTTGTTCAGATTATAAGGACTGCCAATAGTCAGGAGCTTTTCCATCTTCAACCCAGAATGGTTTGCCAGGTATTGCTGCAAGAATACTGTATAAATTAGGCCACCGTTAGAATGCCCTAGCGCCTTAAAACTGTTAAAAGAGTACTTTTCTCGCAAAACAGTCAAGGCAGCATCAAACATTGCCGCCTGCTGCTTGATGTTTTCATAACCATCGCGATTATTTTGAAAGCCAACCACAAAAATGGGATTTCTATCCTTCCGCTTCAAATGCCCTCGGTAAGTTATATGCCCGTCATTCCAGACTTTAATACGAACCAGACTATGATGTGGATGCTGATTGCGATTGAGTTTTTTAACCATTCGATTAAAACGATTCTCAGTCGCCGAACTACCTGGAATCATGATGATCGGGCTGATTTTTAACTTGCTGGCAGGTAGATGAGAAATAGGGTGGGAAGGCAGCTTATTCAATAATTTTTGAAAGGTTTTAGCAAGTCGATGAATCAATTGAGTCAAAAAAGGCCTCATTCTAGTCTCCATATCTGTATCTGATCTTTCCAAAAACAAGCCACATAACGGATTTATGTAGCTTGTTATTTTCTCTTTGAAAGAAATATGCTAAATTACTTTATTTCTTAATTACATTATTTAACTTAAATTGCTTTCTATCTGAAACATTTTTTCTTTAGGTTTATCTTCCCCTAATAATTGATTATTAAAAAAATTAGCCATTGGAAGAAAAAAGTCCAAATCTAAATGCTTTCCATTAACGACTGGTATTTTTAACTCAATATTTCCAATTTTAATAGAAGAGTTTTTTCCTTGAAGGGCTTTTATAGTTACTATTTCATCTAAATTAATTTCTGGTATATTCTTAATAGTTGAAAATACCATTGATTGACGAACTATGTCGTAAACATTTACTTTACGCATACTCTTTAGAAGATGAGTTCGAGCATTATAAGCGTATCCTGAAACAAGTAAAGCAGTAAAAAATTCAAATGGATTCTCTTGGTAACCTTTTTCTAATACATCAATTGCTGCTTCAACAGCTAATTTTGAATGATAAAGTTGCCATCCTAACGCATCTGAATAACCTGCGTTCCCTGCAAATTCGTTTTTTATTTGAGCTATTTCCTTTTTTACTCGTTGAATATAGTCTTCGCTATTTTTCATATAAGCAGAAATAGCTCGTTTGTTACCAAAGTTTCCTGCTCTTCTAAATCCGTAGCGTCTAACTTTTAGAGAAGTTTCTGCATTATCATTATTTTCTTGATATATCACTGAAAAATCTGTAAAAACACTTAACGAACTATTTATATCAATTAAACTATAAAGCTTTTCAGTAGGGACTAATAATCCAAAAGAAGCTAAAGTAAGGTCTTGAAACCATTTCTCATTTTGGTAACCAGCTTGTACAGCAATTGAATTCCAGTAATGAACATCACGATGCGTATGTCCACTAATTAACCAATATCTGTTATCTGGCTCAGGCTTATAAATACTAGGATAAGGTGTTTCTTCTTTTGGGAATAAAGCAAGAGTCTGCCACCAAGAATCTATTGCGTTTTCAGCGAGTGAATCCATCGGCCAGTGTGTTACTATGAATAGTGATTCATTTTTATTTAATCTAGTAATTCTCTTATTTGCAAAATCACACCACTCTTTATTAAGTTTTTTAAGCGTTTCTATATCGAAATTTTTAACTTGAGTGCAATCTGGTACGCGGTTTTCTATGTCTTTGATTGTTGCAGTTAATAACCAATTTGAATCATTTTCGTCAATGTATTCAAATAAGTCATAAACATTTGCTATGCGATTAAGGTCTAGGACTGTATAATTTAAATTAGTGAAGCCAGTATCTCCGATAAAAACCAAATTACCTATTTTGTACTCTCTACCAGTAAGGAGAAGGCGACAGAATTTATTTCTCTCTGTTGCCATTGAAGCTTGCTCAATACATTCAATCCACGTTCTTTTTTTATCTGAATAAGTCCAATAATCGTGATTTCCTAAAACTACAAAAGATGTAATCTGAGCTTTTTCGAGAATTTGTATAAAATCAAGCGTCTCAAAGAAATCGTCAAAAAAATCTCCAGCTAAAACAAAAATATTTCCTTCTCTATTTGAATATAGTAAATCAATAAAAAATTTTATATTTTGTTTAATTTTATTTTTAGAATATTGATAATCTCCACTTTTCAATACTTCTTCCATATATTTGAAATGAGCAATAGGATCATTCTCTAATTTTATTAATTTCTGTTTTACATACCCTGGAAGTGCTTTTTTATCAAAATGTAAATCACTCAATACATAAATATTAGGTTTTTCGTTCATAATAATCTCCTAAATTTATAAAACTAAATCCTAACTAAGTTAATAAAGAAATTCTTCTCCTACTTCCGCTTCTTCTTGTTTTTCTTCATTTGCTTGGCCATTTTGTTCATGCTGCGACGCATTAGAAATTCTCCAGCCTTCCCTTTGAGGCCGCCACCAAACATTTGGCTCATATCCGGCATGCCGGATCCTCCTAAGCCTGACATATCTGGTATACCGCCTTGGCCCATCATGCCTTCAAGAGCTGACATGTCGGGCATTCCTCCGCCACCAGGCATGTTCTTAGGCATATTGTTTGGATTGAGCCCCATTTGCTTCATCATCTTGCTCATATCGCCTGACAGAACGCCCTGCATCATCTGCTTAGCTTGATTGAAGTCTTTGATAAATTTATTGACATCGACAAAGCTGTTACCTGAACCATTGGCAATCCGACGACGACGGCTAGGGCTCAGCAAGTCAGGATTTTCCCGCTCAGCTGGTGTCATAGAGGATACAATGGCACGCTTGCGGGCAATTTCTTTCTCGTCCACTTTGATATTCTTGAGGGCAGGATTATTGGCCATACCTGGAATCAGCTTGAGCAAATCCTCCATAGGCCCCATGCCTTGTACCTGATCCAACTGATCAATAAAATCATTGAAATCAAAGGTGTTTTCCCGCATCTTTTCAGCCATTTCAAGCGATTTTTTCTCATCGTATTCCTGAGAGGCTTTTTCGATTAGAGTCAGCATGTCCCCCATGCCCAGAATCCGGCTGGACATGCGGTCTGGGTGGAAGGTTTCGATATCAGTAATCTTTTCACCGGTACCAGTAAATTTAATAGGCTTACCAGTAATCTGACGGACAGACAGTGCCGCACCGCCACGGGTATCACCATCAATCTTAGTCAGGATAACCCCGGTCACTTCCAGTTGGCTGTTAAATTCTCGAGCGACATTAGCCGCTTCCTGACCAATCATGGCATCGACAACCAAGAGGATTTCATTGGGATTAGCCAGCGCTTTGACATCAGACAGCTCCTGCATGAGCTTTTCGTCAATTTGAAGACGCCCAGCTGTATCAATCAGGACATAGTCATTGTGGTTAGCTTTTGCCTGCTCCAAACCTTGACGGACAATCTCAACAGCCGGAACTTCAGTGCCTAAAGCAAAGACAGGTACATCAATTTGCTGTCCAAGTGTTTTCAGCTGATCAATTGCCGCTGGACGGTAAATATCCGCCGCAATCATGAGCGGACGAGCATTTTCTTCTTTCTTGAGCTTATTAGCAAGCTTACCAGCAAAGGTAGTCTTACCAGCCCCTTGCAGACCAACCATCATGATAATAGTCGGAATCTTTGGTGACTTGATAATCTCAGCAGTATCAGAACCCAAGATAGTTGTCAGCTCTTCGTCAACAATCTTGATAATTTGTTGCGCTGGATTGAGGGTGTCGATAACCTCATGACCGACAGCACGCTCACGGACTTTCTTGATAAAGTCTTTGACAACCGGCAGGGCAACATCGGCTTCTAAAAGGGCCAAACGAATCTCTTTGGTTGCTTCCTGGACATCGCTCTCTGAGATTTTTCCTTTTCTGCGTAGATTTTTAAAGACGTTCTGCAAACGTTCTGTTAAACTTTCAAATGCCATTTTTTATTTCTCCTAGTTTCTATTTCAATCAATTCAGTGGAAATTTTCCAAAACGTGTTAAACAAGCGTATTCTTCAGCAGAGACTTCTTTCATAATCTCATTTGGGTAGCCCCAGCAGCTGAAGCCTTCTTGCATTGCCTTAGAAAGATCGTCAGGGGAGATGATTTGTACCCTGGCAGGAAAAGATTCTTCCAACTCTAACAACCAGCAAGACATTCCCTTGTAGATAACTTGTTTAGCCATGTTCACTCTCGGTTGTCAATACCAGACAGAATCCCAATCTGCTGCTGCAGATAGCTGTCGTCAGGGTATTTCTCTGTAATCTGGTCAAATATCTGACTTCTGACAATATAGTCCGAGTACATGTGCAGCTTTTTCTCATAGTCTTCCAGAATCTTCTCTGTCCGTTTGATATTATCATAAACGGCCTGACGGCTAACACCAAACTCCTCGGCAATCTCCGCCAGACTGTAATCATCTGCATAGTAGAGTTCAATATAGTTCATCTGCTTGTCAGTCAAAAGCGCCGCATAAAACTCAAAGAGAGCATTCATTCGGTTTGTTTTTTCAATTTCCATAAGAAACATTATACCAGAAAAGCATGATAAACTCTAGTCTGACAAGGGAAATAAACACTCGAAATATGAGATTGCAAAACTTCAATCAGCTTTTTCTTCTGT

>c183_g14

CAACGATATTATTTAATTCTTTCGTTCAAAATGTTCCCGCTGTTAGCATCAATGGTTATTTTAATCTTTTGCGTACCGTTCAGAGCTTCCAGCTTGTGAGATAAGCACCCCTATTCATTTATTCTATTTAGAAAGATTACTCAATAGATACTGTCCGTTTTCCTGACGGATAAAAGTCAAAGTTATCTCCTTATTTGAAGAGGTTTCATAGTTTACAAGCATCTTCGGCTCTGATTCACCATCCCCATAAACTGAGATATGTTGATATTCTGGGTATTCCTTGATTACTTCTTTAAAGGAAGAACCGCCCTCGCCTGTTTCCGCATCACCCGTCTTTAAGCTCTCTGTAATCTGGCTAGATACCTTTTTATTATAGCTGTCACTGCCGGGCATAATACTCATACCCTGAAGATGGTAGCTACCATCCTCGTGTTTCATAAAGGTCAGAGTGGTATAACTAGAGTCAGGTAACCAAGACAATGAGAGCCCTTCTTCCTGAAACGTCACACTGCTGGCCAAGCCAAAATCTTTGACTACTTGTTCAGGGGTCAATCCTTCATCATCCGCACTAACAATGCTCAGCTTTTCAAGGTCTTTCACTTCCCACTCATACACATCCTCTTTTGACGGAGCCACAAAAGAAGCAACATCGCTTTGATGTCCCTTTCGAGACAGACTTGGCACACTTCCCCCACCCATAAAATCATTTAGGAAATAGCCGCCAAGACCTCCTAGCAATAGTCCCGCTGCCAAACCTATTCCTAAAAATCCTATCAGCTTGCCCGTACTATTTTTCTTTGGCTCTGGACTTGGCTGGGCAGCGAAGTTTGCAGCTTGCTGTGGTCCGGCAAAGTTCTGACCAGCTGGATTAAATCCCTGAGGAATTGCTTCTTGAAAAGCAGGATTTCCCCAATTATCCTGCTGAGGCATCTGCTGCTGAGGAGCATCAAAAATTGGTTGCTGAACACCAAAATCTTGATTAGGATTCGGCATTTCAGGACTGCCCCCTTCTACTGGGAGAGGCTGCCCCTCTGTTGAGTTAGCTACAAATTGTCCTGAGTCTGATGACTGGACTTGCTGCGGGTACGGAGCCAATCCTTCTAAAGATTGTTCCTGCGGTTTATTTTGTTCTGACATCGTTTCTCCTTTTATTATTTGAGTTCTGTTTTATTTATTGGCAGTAGTGGACAAAAGATAGTTTCCGTCCTCCTGCTGAACGAAGAACAACCTATAAGTACCATCATTAGGAGCGTCATAAGCCGCCTCCATAATGGTTTGACTAGTATCCTCACTAAAATCTTCATCTACACGAATAGAGATACTCCTCGGACTGCCATATTCCTTAAAAACTTCCTTATAGGAAATGCCATCTTTACCAGTCTTTGCATCGCCTTTTTTCAATTTTTCAAAGTAATCAGCCGCCATCGCTTCGTCCTCAGCATTATGTTTGCTTCCTTCAAAGCGAATATTATAGATATGAAGGCTATTCAGATAGAAACCATCTTTTTTCTTCTCAAAAGCCAGAGAAGCGGTTTGATCTGTATAATAAACTGGCCAGTCCTCCTCATCATCAGAATCTTGAAGCGTTCCCCACTCTAAATCAAGGCTGTCACGAGAGATTTCTTCTTTTAAAGCCTTGCCATATTTATCAAGAATATCTTCCACAGAAGTACCATTCGTATCTGTCTGAATAGTATTGAACCGTAACTCTGACAAACTATCAATGTCCCACTTGAACTCAGTAGTATCAGGGTCAATGTACTGATCTGATTGATCTCTCTTTGATTCCCTAGCATCTGCCAAAGGATGAAGCACAGGATATTCTCCTCTGATGACCTCCGCTACTCCCACTCTATATCCCCATATTCCCCCTCCGATTAGTCCAAGACCAAGAGAGAGCAAGGCTGCACCTATCAGGATTCCTTTCCAGGATTTATTCTTGGTTAAAGGTGGGGTTGGTCCCTGCGGACTTGAAACATGGCCCTGCTGAGGTTGAAAATGATGCGGGAATTCCTCTTGCGACTGAGCATTCCTTGAAGGCTCAAAAGAATGCTGTGTTTGCTGTTCTGTCAATCTTCTTTCCTCTTTCTTGTTTATTTCCAATAGTCTTATCAGTTCTCTTTTAATAACGAAATGCTTTGAAAGCAGTCAGTCACAAAAGATAAACTAAATAACTAAAAACTTACATTTCCTCTGACTTTATATATAAGAAATTTCCATCTGGCTGGCCTACAAACAATAGGTATTCATAACCATCTTCTGTTTTATAAGTCACCTCAGCAATCAGACGGTTGGAGATAATCTTATTATCTACGTCACGCTCGCTTGTGATGACAATTTCTGACGGAACAGGATGCCTCTTCAGAACCTCTGATAAAGCTGTTCCTCCTTCACCCGTTTCCTTGTCTCCTTTTTTCAAACGTTCAAGGTCTTTGGGAGATAATACGCTCCAATCTGTCGTTTCCTTCCCTTTTCTATATTCTTTAAAAGAATCTCCAACTACCACTTGCCTCAGATAGTAGTTCTGGTCAACTTTATCAAATTGAAAAGAAATATTTTGCTCCTTGTCCCTGACAGAAGGGCCCCATCGGAGTAACAGGCGATTACGATCATATTCTGCTGAACTAGCCAAGCCATAAGTTTCAACGATTTGATCAGCCGTCAGACCTAATCCTTCATCATTTGTATAGCTCAACCGTCCCAAGTCTTTCAATGTCCAATTAAACTGAACCGACTGCCCATCTGTAGTAAAGCTGTCACTCTTGTCTTTGGCTTCTTCCTGAGCCTTACTCAGACGCTTAATCCTTTGTATATCCTTATTTGTCTGCGCCTGCTTGCCGATAAAATAAGCTGAACCGCTGCCAATCAAAAGACCAATTATGAGAGCAAAAATTGAAAGTCCTATGACTTTTCCGATATTGCCTTTCTTATCAGGTAATTCTTGCGGCTGAATCTGCTGGAAATTCCCTTGCATTGGCTGGCTAGTTTGATTTTCTTGCCAAGCCCCTTGCACTTGACCTTGACCATCTTTATCTGAC

>c183_g140

TTGATTATAACATTTTCCTACATTTTGAACAAAGGAAAACTGAAGCATCAAAAAAGAAGAGACTGCCTGAGTCTCTCCTAGAAATTTTCAAGAATTGTCAATATTCAGGTGTGGGCGAAGAACATTTGCAAAGGTTTCAATATTGAGACTTTGGATGTAAATTTCCCCAGTTCCATAGAAAGTATTGACCACTCCTTCACCAGTCCCCATGGACTGCCAGAAACCATTTTCTAAATGAATATCATATTCCAACTCTCGACTCCATGCTACTACGTGGGCATTGTCAATCGTTATCCTATCATTATGCAGCTCGACCTTTTGAATCGAGCCAAAGGAATTTACCAAGAGTGTTCCCTGACCTTGGGTACTCATCACATAAAAGCCTCCTTGGCCACCAAAGAGAGCTCGACCTACCGACTGCCTCTCCAAAGTATAAGAGGCAGAGCCATCCATAGCCAGAAAAGCTCCATCATTAAGGCGGTATTGCTTTTCGCCTAGTTCCAGAGCAACAATCTGACCAGGTACATTAGGCGCTAAGGCTAGACGACCGTTATTTGATTGAGCAGTTGCTTCAGTGATAAACACTCCTTCACCAGAAGCCATAGAACGGCCCACAGCTTGGATTGCCATGCCAAAGATTGATTGAGACTCTGCATTCACTTTAGCATTCAAGACAACGTTGGGAGTGTGGTATATCATGCTCCCGGTCTGAATACGAACAGACTCTGATGCATCTAAGGCAATCTCCGCCAAGGGAAATTGAGCATTGTTGGTAATAGAAACATTCATACGATGATCTACCATGGTTGATTATCCTCCTAAAAAATCTGGAAAAACAACTTATAGGAATTTCCAGACAAATCCTATTTTTCTAGCAATATTATAGCACGCACAACAACTTTTCGCAATACTACTTTAATCAAGAAAATACCTTCTTTAAAACTTCCCCAATCGTCGTCACACCGATGACTTGGATATTGTCTGGAACTTTGAGGCCGTTCAGAGAGTTTTTAGGAGCGTAGACTTTGGTGAAGCCCAGCTTTGCTGCTTCGTTGATGCGCTGCTCGATGCGATTGACTCGACGGATTTCACCAGTCAGGCCAATTTCACCGATAAAGCACTCTTGAGGATTGGTAGGCAGATCCTTGTAGCTGGAAGCAATGGCGACCGCTACTGCCAAATCAATGGCTGGCTCATCTAGCTTGACACCACCAGCTGATTTGAGGTAGGCGTCTTGATTTTGCAGGAGGAGCCCTGCCCGTTTTTCCAGAACCGCCATGATGAGGCTGGCTCGGTTAAAGTCCAGTCCAGTTGTGGTCCGCTTAGCATTGCCGAACATGGTTGGTGTCACCAAAGCCTGCACCTCTGCCAAAATCGGCCGCGTCCCTTCCATGGTCACGACAATAGACGAACCTGTCGCGCCATCCAGACGCTCTTCTAGAAAGACTTCACTTGGATTGACAACCTCAACTAGCCCACCAGACTGCATTTCAAAAATGCCAATCTCATTGGTTGAGCCAAAGCGGTTCTTGACCGCTCTCAAGATACGGAAAGTATGCTGACGTTCACCCTCAAAATAAAGCACGGTGTCCACCATGTGCTCCAAAGTTCGAGGACCAGCTAAGGTTCCTTCCTTGGTCATGTGACCAACGATAAAGGTCGCAATATTATTGGTCTTGGCCAGCTGCATGAGCTCAGCTGTCACCTCTCGAACCTGAGAAACAGAGCCTTGAACACTGGAAATTTCCGGCGACATCACTGTCTGAATGGAGTCAATAATCAGAAAATCTGGCTTAATCTTCTCAATCTCTGTGCGGATATTTTGCATATTGGTCTCGGCGTAGAGATAAAATTCACTGTCAATATCACCGAGGCGTTCCGCCCGCAGCTTAATCTGCTCAGCCGATTCCTCCCCGCTGACATAAAGGACGGTGCCCTGATGAGAAAGTTGAGTGGATACCTGCAGAAGCAGGGTGGACTTCCCGATGCCGGGATCCCCGCCAATCAGGACCAAACTGCCAGGCACGACGCCACCACCCAGCACACGGTTGAACTCATCCATTTCTGTCTTGGTCCGGTTGACATCAATCGAAGTGACCTCAGCCAGCTTCATGGGCCGGGTCTTTTCGCCTGTCAAGGAAACACGGGCATGCTTGACTTCTGCAGCTTCTACTTCCTCAACGAAAGAAGACCAAGAGCCGCAGTTAGGACAACGGCCTAGATACTTGGGCGAATGATATTCACAATTTTGACAGACAAAGGTCGTTTTTTTCTTAGCGATGGTGATTCTCCTTATTTTCTAATAATCCAAACAAAAGTAGATAAGACCTGACACACTTGCTCTATATTGGTAAAACCAGCCTCTTCTAGCAAGCCTGCAACTTCTTGAGGCGAGAGGGAATTCATCAAACTAGCTTGATTCTCATAAGTACGCTGAACTTGTTGCTGATTAGCTCCTAGCTCTGAAGCAAGTGTTTTCCAATAGCCCAGTTGCTGGTTGGAAAAAAAGCTAAGAATCAGAAGGCCATCTTCACTTAAAGAATCATAAATTTTTCGAAGAAATAAGCTGGGATTTTCAACAAACTGCAATACTAGCAAGCAAGAACAAATCTGATAAGCAGAAGGCAGCACTGCTTCTTCAAACCGACTATTAAGATACTCCGCTTGAGGCAGATGAGCCTGTCTTTTAAGCTCTTGTAGCATCGCCTCACTTGGCTCTACTACCGTCAATCCGGCCTTAGGAAATAAGGATGCCAAACCATTCAGCTCATCCGTCTGGCTTGCCAAAGCTAACACCTTATCGACTTTTAAACTGGGTGCTAACCTAAGCAATACTCCTCGGAAAATCACATCTAGCATCACATCATAGCCTGGAATTTTCTGACGAATTTCTGTTGCATAGTCCTTGTTCTTAAATTCTTTCATATTTTCTTCTATCTCAAAATCCTTTTATTAAGATTCTTTTTACAACGAAACATTAAAACTTCAGTTCACATTCAACAAACTGGCAAAAATCAATCTGCTGATCAGCTACAAACTGGCGGATGAGCATGCGATGGGCTACAAGAGCTACCGTCTCGTAGTCTCTGTATTTGCTTAAGGCACTGAAAAAGCGGGCTTTCATCTGCTCAGCCGTTTCATACCGGACAGGACTTCCTGCAGGAAGAGAACCATTATTCTTAAAAAATAACTTCCGAGCCTGCTCAAACTTTTCTGTCCCACTCTCATATACCTGCCATTCATGCAGAAAAGGTTCCACAAAAAGCGGTAGTTGGCGGTCTCGAATCAGATAGGAGGCCGTCTCCAAAGCCCGCGTCACAGAAGAGGAAATCAGAATTTCCGCCTGTTCCAGCAAAGGATTAGCAGCGGCCTCTGTCGCCATCCGACGACCAGCAGCCGACAAGGGTGCCAAATCTAGTCTAAATCCAGTATAGCCGGCCTCTTCCAAAAGTGAATAATCTGGCTCTCCGTGACGGATAAAAATGATTTTCATACTAATGTCCTGTCGACCCAAATCCACCGGTCCGCACACCTTCCGCTTCATCTCCATCAGCAATCAGAAATGGTGCAAAGACAGCCTGCACTACACGTTCGCCAACTTCAAGGACGACTTCCTGATCCGTGATATTCTTCATTTGGGCAAAGATATGGCCTTCGTTTCCAGGATTGCTATAGTAGTCGCCATCAATAACCCCAACGGAGTTAATCAAGACTAGGCCTTTTTTACGAGGATTGGACGAGCGATCGTAAAGATAAAGCACCTCGCTCGGCTGCATATAGGCCTTGACACCAGTCGGAACCAGCTTAATCTCCCCTGGCGCAATGAGGGTGCGCTCTGCCACTTTCAAGTCGTAGCCAGCTGCGTGAGCTGTCTCTCTCTTTGGCAATAAATCTTCATTTGTAAAGCTGGAAACCAGCTCGAATCCGCGGATTTTCATGTATTTCTCTTTTCTATTTTTGCTGTCCCTATTATACTAAATTCGCAAAAAACAAGCAAAAAAACCGCCGATTGGCGATTCTCCCTAGTATAGATCTCTGATGATCTCTCCATTATCCAGATAGGTAAACTGCTCCGTCAAATCTGATGTGAAAACTTGATACATTTCCTTAAAGCTCACAGCCAGCGAGTAGCTGTCAAAATCTTCACGGTTAATCCAGAACACCTCGCCTTCGTCGGATGAAACTAGCTCTCCTTCAAAACGATTGGTCCTGTAAAGAAAAACGATGTAGCGCTCATCTTTTTCAGTATAAAACTGCTTGACACCACACAAGCGAGGCTCATAGATAGTCAAACCCGTTTCTTCCTTGATTTCCCGTATCACTGATTTGACAAAGGATTCACGATTTTCAACATGGCCACCTGGAAAGCAAAGACCGGTCCAATCGTCATTTACTTTATTTTGCACTAAAATGCGATCTCCATCGTAAACCATACACATATTTGTTAAAATAACTCGCTCCGCTCTTGACATCTCTCTTCCTCCATCTACCGACCGATTCGCTCGGCCTACTTGTGACTCATTATAACATAAAAGACATCAAATTGTTGAAAATCCAGATTTCTCAGCCTTTCCAGCAAAAAAACCGCCAATTGGGCGGTTCTTATAGGGAGA

>c183_g141

TGTTGGACTAGGACTAGCAAACATCTTCTAATATTAACTCGCAATGCGGTTCTTCAAGCAGTAATTTAAAGTTTCGCTTAGACAAAGCTTCACCCAACTGATCCTTCCATTTCATAGCTGATCAAGCGGTTAAGCTCAACAGCGTATTCCATAGGTAATTCTTTGGTGAAAGGTTCAACAAAGCCCATGACAATCATTTCGGTAGCTTCAGATTCGGACAAGCCACGGCTCATGAGATAGTAGAGCTGCTCTTCGGAAATCTTAGAAACCTTGGCCTCATGCTCCAGAGCCACCTGAGAGTTATGAATTTCATTAAAGGGAATGGTATCTGATGCCGAAATATCGTCCATGATAATCGTATCACACTCAATATGGCTGACGGATTTCTGTGAATTCTTTCCAAAGGTCACTTGACCGCGGTAGTCTACTTTACCTCCGCCCTTAGCAATAGACTTAGACACGATAGAAGAGCTGGTATGCGGAGCATTGTGAATCATCTTAGCCCCTGTATCTTGGTGCTGATTGGTATTGGCAAAGGCAATAGACAACATTGTTCCGCGCGCTCCAGGTCCATCCAGATAAACAGACGGATACTTCATAGTTGTTTTTGCACCAAGGTTGCCGTCAATCCACTCAACCGTCGCATCTTTCATGGCCCGCGCCCGCTTAGTCACGAGGTTGTAGACATTATCAGACCAGTTTTGAATAGTGGTGTAGCGCATATAGGCACCATCAAGGGCAAAAATTTCGACAATTGCTGCATGTAGACTATTACTAGAGTACGTCGGAGCTGTACATCCCTCAACATAATGGACGCTAGCGCCCTCATCAACGATAATCAAAGTCCGCTCAAACTGACCCGAATTTTCATTGTTAATCCGGAAATAAGTCTGCAAGGGAACGTCAACCTTGACACCTTTTGGAACATAGATGAAGGTACCACCTGACCAGACTGCGGAGTTGAGAGCTGCTAGCTTGTTATCTGTCGGAGGAACCAGCTTAGCAAAATACTGCTTGAAAAGCTCTGGGTATTCCTTGAGAGCTGAGTCGGTATCCGTAAAGACGATCCCAAGCTTTTGGAACTCTTCCTTCATATTGTGGTAGACCACTTCTGATTCATATTGGGCTGCTGCACCAGCCAGGTAGGCTCTCTCCGCTTCTGGGATTCCAATCTTCTCAAAGGTTTCCTTGATCTTTTCAGGCACTTCATCCCAACTTCTAGCAGGCTTATCAGAGGCCTTTTGATAATAAATCAAGTCATCAAAATTAATTTCTGACAAATCCGGCCCCCAAGTCTGCATCGGCATCTTCTTGAAAGCCTCGTAGGATTTGAGGCGGAAATCCAGCATCCACTCTGGTTCATCCTTGGCTGCAGAAAGCTCACGAATGACCGCTTCATTCAGCCCTTTCCCTGTCGAGAGAACAGGTTCAACATCGTCATGGAAACCAAACTTGTATTCACCGAGATCAATCGGTTTTGGTTCTACTCTTTCTTCTGACATAATTTCCTTTCACATTCTTACTTTTTATCTTCTTCAATTGCTCGCTTGAGGGCATTCCAACCCAAGGTCGCACACTTAATTCGCTGCGGGAATTTGGCAACGCCTGCTAAAAAGGCGCCATCTCCCAAGTCTTTCTGGCGACTGTCTTCCTGGCCCTGAACCATCTGCGAGAAGACTTCTGCTAATTCCAGCGCCTGCTCTTTTGTCTTGCCCAGAACCGCATCTGTCATCATGCTGGCTGAAGCCGTTGAAATGGTACAGCCGGAATTCACAAAAGCAATATCCTCAATCTTATCTTCCGCATTAAACTTCACAGACAGGCTGATAACATCGCCACAGGTCGGATTATTGAGAACTACTTGCTCCACATCTTCCAGTTTCCCATGATGATGGGGGTGAGCCGAGTGGTCGGTCACAACCGCCTTGTAAAGACTGTCTAACTTAGAAAGCGCCATTGAAAAACTCCTTTGTCTTTTCTAAAGCATCTACCAGCTTGTCACAATCTGCATAGGTATTATAGATATAAAAGCTAGCACGCACAGTCGCTGGCACCTGTAGATAGGTGAGCAAAGGCTGGGCACAATGGTGACCCGCCCGAACAGCCACTCCTTCGTAGTCCAGAGCTGTCGCGACATCATGCGGATGGAGACCATCCAGGTTAAAGGCAATCACGCCCGAACGCTGGGCCAAATCCTGAGAGCCATAAATGGTCAAGCCTTCCACAGCCTGCAACTTAGGAAATACATAGGCAATCAGGTCCTGCTCATGCTGAGCAATGGCATCCATGCCCAATTCTTCCAAATATTCAATGGCCGCCGCAAGACCGATTGCTCCTGCCATATTTGGAGTGCCGGCCTCAAACTTCCACGGAAGCTCCTTCCAGGTCGCTTCCTGCTCATAGACAAAATCAATCATTTCGCCGCCGAACTCGACTGGCGACATCTGCTCTAGCAGCTCTTCCTTGCCATAGAGAACCCCGATACCAGTCGGGCCTGCCATCTTATGCCCCGAAAAGGCGAAGAAGTCTACATCCAAATCCTGCACATCAATCCTCATGTGCGGAATGGATTGAGCCCCGTCCACCACCAAAAATGCTCCTTGCTGATGAACCAGCTGGGCAATCTCCTTGATGGGATTGATGACACCGAGGACATTAGAAGCATGAGCCAGAGAGACCAACTTGGTCCGCTCATTGAGCTTGGCACGGAAATCCTCCATATCCAGAGCACCATCCTTGAGATAGACATAGACTAACTTGGCTCCGGTCTTCCTACAAGCCTCCTGCCAGGGAATGACATTGGAATGGTGCTCCATGATAGAAATCATCACTTCATCACCAGGCTGCAGCCTTTCAGCTGCAAACTGAGCCACCCAATTGAGACTCGTCGTGGTTCCTCGTGTAAAGAGAACCTCTCTGCTAGAAGCCGCATTGATAAAAGAACGAACTCTTTCCCTGGCTGCTTCATAAGCTGCTGTCGCCCGCTCAGCTAGCGTATGCACACCGCGGTGGACATTGGCATTGTCTCTAAAATAGTAGTCTTCGATAGCCGTCAGCACCTGCTTGGGTTTCTGAGTTGTCGCCGCATTGTCCAAATAAACCAAAGGCTCATCATTAACAATTTGGTCCAAAATGGGAAAATCTTGCTTGATTGCTTCTGCATTAAATCCAGACATGAAGACTCCTATCTAACTAATACGGGACAAGGCCCAGCTTTTTATCTTTTTGCGAGAATAATATCGATATTTTCAATCATTTCATCACGGACTTCTTTAACAGGGATTTCCACAATCACTGATCCTAAGAAGCCGCGCACGACCAAGCGTTCAGCCGTCGCCTTATCAAGACCCCGACTCATGAGATAATACATATCTTCTGGATCCACCTGACCGATAGAAGCCGCGTGACCAGCAGTCACATCGTTTTCATCAATCAAGAGGATGGGATTAGCATCTGAGCGCGCTTGATCAGACAGCATGAGAACCCGGCTTTCCTGCTGGGCATCTGCTCCCTTAGCCCCTTTGATAATATGTCCAATACCATTGAAGGTCAGGGTTCCTTTTTCCAGGATAACCCCGTGCTGCAGGATATTTCCGATAGAGTTGCAGCCATAGTTAGTTACGCGGGTATCAATCCCCTGAACCTGCTTACCGCTTGAGAGAGCCACCACCTTCATATCCGCATGGCTACCCTTGCCATAGAGGTCGCTATCAAAGTCAGCCACAACATTGCCTTCGTTCATAACGCCGATGGCCCAGTCAATCATGGCATCATTATCCAGCTTGCCACGACGACTGATATAAGCTGTGACATTTTCGCCCAAGCGATCAATAGCTGAAAACTTAATCTGAGCTCCAGCCTGAGCAATAACTTCGACAGTGATATTGGCTGTTACTGGAACAGAGCCCTCACCATAAGTCTCCAAGCGTTCCAAGTAGTTAACCTTGGAATGCTTGCCTGCGATAATCAAAATATGCTTGTTAAAAGGAACATCGCTTTCGCTGTCCTGATAAAAAATTCCTTCAATTGGCTGGTCAATCTCAACATTGTCCGGCACATAAAGAACGGCACCGCTGTTGAAATAGGCAGTGTGGTAAGCTGCCAATTTGTCCTCGTCGTACTTGACCGCAGACATAAAATGCTTCTCTACCAGCTCTGGAATCTCTTCCAAAGCTGTGTGGAAATCTGTGAAGATCACACCTTGTGCTGCCAAATCAGCCGGCAATTGCTCCAGAACAGTATGAGTTCCCACCTGAACAAGTTTGAGATTGTCATCAAGTGCGGTAAAGTCTGGAACACTTGTCAGCGGCTCGCTCTCTGAAATACGGCCGTCTCCCAGATTCCAGCGGTGAAATTTAACCCGCTCAATACGTGGCAATTCTAACTGGTCAATTTTATCAAAGGCCTGCTGGCGCAGTTGAAAGAGCCAATCTGGCTCTGCATGTAGCTGTGAAAATTCTTGAATCAATTCTTTAGTCATGTCATTCTCCTATCTTTTATCAAAGTCAAAAGAATGTTTGACTAGGCTTCTTCGGTATAGGTGAAGCCCAACTCTTCAGCCAATTTAGCATAGCCTTCTTTCTCCAAGCGGACTGCCAATTCTGGACCGCCAGAAAGAACGACTTTACCATCCATCATGACATGAACCACATCTGGAGTGATATAGTTGAGCAGGCGTTGGTAGTGGGTGATAATCATAGCACCAAAGCCTTCACCGCGCATAGCATTAACCCCTTTGGACACAACCTTAAGAGCATCGATATCCAGACCTGAGTCAATCTCATCCAAGAGTGCAAAAGTCGGCTCAAGCATGAGCAACTGCAGGATTTCATTGCGCTTCTTTTCACCACCGGAGAAACCTTCATTAAGGTAACGCTCAGCCATTTCTTCTTTCATGTTGAGCAGTTCCATCTTTTCATCCAGCTTCATGATAAAGTCGCGGACAGAGATTTTTTCCTCTTCTTCCTTGCCAGCATTCATAGCTGCACGTAGAAACTCTGCATTAGTAATACCAGGAATTTCACTAGGGTACTGCATAGCCAGGAAGAGTCCCATGCGAGCACGCTCATCCACTTCCAATTCTAAGATATTGACTCCATCAAAAAGCACCTCTCCTTGAGTCACTTCGTAGTTAGGATTGCCCATGATGGCTGCAGACAAGGTGGACTTCCCTGTTCCATTTGGGCCCATAATAGCTGCAACCTCTCCCGTTTTCAGAGTGAGATTCACCCCTTTGAGAATTTTTTTCCCTTCGATTTCAACATGAAGATCTTTGATTTCTAAGACAGACATTTCTTTTTCCTTTCTTTGCTTAGCTGATTTACACACTTAAGTATACC

>c183_g142

AGAAAATCAAATATTCTTCTTGCCGCAGGGCTCACTCTGCTCTCTGTCGGCTTACTTACAGCTTGCTCTGGAGGCGGAAGTTCCCAATCCAGCAAAAAAATCTACAGCTATGTCTTTACGTCTGATCCAACGACTTTGGACTACATCCAGTCAGCAAAGGTGTCTACCCACGAGCTGACTACAAATGGTGTAGACGGCTTGCTGGAAAATGATAAATATGGAAACTTAGCTCCGTCTATCGCAGAAGATTGGACAGTCTCTCCAGATGGTCTCGTCTATACCTATAAGCTGCGCAAAGATGCCAAGTGGTATACCGCTGATGGCGAAGAATACGCAGATGTCACGGCTCAGGACTTTGTGGCAGGTATCAAACATGCTGCTGATGTCAAATCAGATGCCCTCCCTCTTATCCAAGATTCCATCAAGGGACTGAGTGAATATGCAGCTGGTACCAACAAGGACTTTTCAGCTGTTGGAGTTAAGGCACTGGACGATCATACGGTTCAATATACACTCAACAAGCCTGAAACCTACTGGAACTCTAAAACAACATCAGGTGTTATGATGCCGGTCAACGAGGCCTTTCTAGAAAAGCAAGGCAAGGAATTCGGTCAGGCAACCAAAGCCGACTCCATTCTGTATAACGGTCCATTTATCATGAAATCCATTACTTCTAAATCCTCTGTCGAATTTGAGAAGAACCCTAACTACTGGGATAAGGACAAGGTCAAGATTGATGGTGTCAAACTGTCTTACTACGATGGTTCTGACCAAGATTCATTAGCGCGGACCTTCGGAGATGGCGGCTACAGTTTGGCAAGACTCTACCCAGCAACTTCCAGTTATTCCTCAATCGCTGAGAAATACAAAGATAATATCTTCATGACAGAAGCAGGAGCGGGGGTCGGTCTAATCAGCTTTAACATTGACCGTCAAAGTTATAACCATACTTCTAAAACCAGCGATGAACAAAAAGAAGCTACCAAAAAAGCTCTGCTGAACAAAGACTTCCGCCAAGCTCTAGCCTTTGCCCTTAACCGCGAAAGCTATTCGGCTCAGGTCAACGGAGAAGATGCAGCCAAGCCAGCTGTCCGTAATCTCTTTGTTCCGCCAACCTTCGTACAAGCCAATGGCAAGGAATTTGGCACTCTTGTCGAAGAATCACTGGCTTCCTATGGTGACGAGTGGAAAGGTATCAAGCTAGATGACGGTCAAGACGGTCTCCACAATACTGACAAAGCCAAGGCGGAATTTGCCAAAGCCAAGCAGGCTCTTGCAAATGAAGGCGTACAATTCCCAATTCACCTAGATGTTCCTGTCACTCAGAATTCTACTAACTTCGTCAACCGTATGCAATCTCTGAAACAGTCATTAGAAGAGGCTTTAGGAAAGGACAATGTCTCTGTAGATCTACAGATGCTGGCTGAGGACGAAGCACTAAACATCACCTTCAATGCTGAAGCTGCTAGCCAGGAAGACTGGGATATCAACGGTCTCGTTGGCTGGGAGCCAGACTATCAAGATCCATCTACTTACCTAGACATTTTAGTCCCAGGTAACAGCACTCAGACCAGAACCTACCTTGGATTTGAAGACAAAGACAATGCTGCTGCTAAAGCTGTAGGACTGGATGAGTATAGCAAACTGATTGAAGAGGCCGGAAACGAAACACAAGATGTTGACAAGCGTTATGAAAAATATGCTGCAGCTCAAGCATGGCTGACAGATAGCGCCCTCGTTGTGCCAACTATGAGCAGCAGAGGAGCAGCACCGTTCATTTCTCGGATTGTGCCATTTACCAACTCTTATGCTCAGACAGGTACAAAAGACGCCAACTATCATAAATACGTGGAAATCAGTGATGAGATTGTCACGACTAAAGACTATCAAAAGGCCCAAGAAAAGTGGAAAAAAGAAAAAGAGGAATCCAATAAAAAGGCCCAAGAAGATCTGGCTAAACATGTGA

>c183_g143

AAAAGATTGGCTTGGACGACTGAAAAACGCTACAAACGCTATGAAGACTGGACTCAAGAAGAGGTACAGCACATCAAAGAAAACATCGCTAAATCTCCTTGGCGGGCTAACTACCATGTGGAGCCTCAGACTGGTCTGCTCAATGACCCCAATGGCTTTTCTTATTTCGACGGCAAGTGGGTGGTTTTCTACCAAAACTTTCCTTTCGGAGCAGCTCACGGTCTCAAGTGCTGGGTCCAGATGGAAAGCGATGACCTAGTTCACTTCACGGAAACTGGCCTTCGGGTGCTGCCAGATACTCCTCTAGATAGCCACGGCGCCTATTCTGGCTCTGCCATGCAGTTTGACGACAAGCTCTTTCTCTTCTATACTGGCAATGTTCGTGATGAAAACTGGGTGCGTCATCCTTATCAAATCGGTGCCTTGTTAGACAAATCAGGCAAGCTGGAAAAAATTGACAAGGTCTTGATTGAACAACCTGCTGAAGCGACTGACCACTTCCGCGATCCGCAGATTTTCAACTACAAAGGACAATTCTATGCTATCGTCGGAGGACAAAATCTAGACAAGCAAGGCTATGTTAAGCTTTACAAGGCTGTTGATAATGACTATACCAACTGGGAAGTCATTGGCGATTTGGACTTCGCCAATGACAAGACAGCCTATATGATGGAGTGTCCTAATCTAGTCTTTATCAATGACCAACCAGTCCTGCTCTATTGCCCTCAAGGTTTGTCTAAGGATGTACAGGACTACGGCAACATTTATCCAAACATGTATAAAATAGGTCAATCGTTTGACACAAACAAAGCTGCTATGATTAATCCTAGTCCTATCCAAAATCTGGACTACGGTTTCGACTGCTACGCGACCCAAGCTTTTAACGCACCTGACGGTCGCGTACTGGCTGTCAGCTGGCTAGGCTTGCCTGATGTGGAATACCCATCTGACCGCTTCGACCATCAAGGTGCCTTCTCCCTCGTCAAGGAATTAAGTCTGAAAGACGGCAAACTTTACCAATACCCAGTGCCAGCTATTAAGGATTTGCGAGCAGAGGAACAGCCTTTCGCTGCTTTGACAGAAAGCAAGAACAGCTACGAACTAGAGCTGAATCTCGCTGCGGACACCGAGCACGAAATCGTCCTCTTTGCAGATAAGGATGGCAAGGGGTTACGCATCAATTTTGACCTCAAAGCAGGTCTAGTAACGGTTGATCGTAGTCTGGCAGGTGAGCCATTCGCTCTGGACTTTGGAACCAGCCGAAGCTGTAATATTGACAAAGAAGCAACGAGCGCTACTATCTTCATCGATAAATCAATTTTTGAAATTTTCATCAATAAAGGAGAGAAAGTATTTTCCGGCCGTGTCTTCCCGAGAGAAGACCAGACAGGCATTGCTATTACTAAGGGCAATCCAACCGGTACTTACTATGAATTAGATTATGGTCGCAAAGCTAACTGATGTAGCAAAACTAGCAGGAGTCAGCCCCACAACTGTTTCCCGCGTCATCAACAGAAAAGGCTACTTATCCGACAAAACGATCTCTAAGGTTGAGGCAGCCATGCGAGAATTGGCCTACAAGCCCAACAATCTGGCACGCAGTCTCCAAGGAAAATCAGCCAAGCTAATTGGACTCATTTTCCCTAATATCAGCAATGTCTTTTATGCAGAATTGATTGACAAGCTGGAGCATGAGCTCTTTAAACAAGGCTATAAAACCATCATCTGCAATAGTGAGCATGACTCTGATAAGGAACGCGAATACCTTGAGATGCTGGAAGCCAACCAGGTGGACGGCATTATTTCTGGCAGCCACAATCTAGGCATCGAGGACTACAATCGCGTGACTGCGCCTATCATTGCCTTTGACCGGAATCTCTCGCCGGATATCCCAGTTGTTTCCTCAGACAACTATGGTGGCGGAGTGCTGGCAGCTCAGACCTTGGTCAAGGCTGGAGCTCAAAACATCATCATGATAACCGGCAATGACAATTCCAACTCACCAACAGGCCTGCGCCATGCTGGCTTTGCTTCCGTCCTGCCAGACGCACCGATTATCAATGTATCCAGTGATTTTTCTCCCGTCCGAAAGGAAATGGAAATCAAGCAAATCCTCAGTCAAACTAAGCCCGATGCTATTTTTGCTTCAGATGATTTGACAGCCATCTTAATTATGAAAGTCGCTCAAGAGCTGGACATCCAGATTCCTAAAGATTTGAAAATCATTGGCTACGATGGTACCTACTTTGTAGAGAACTACTACCCGCAGCTAGCAACGATCAAGCAGCCACTGAAAGATATCGCCCGTCT

>c183_g144

GGGACCCAGTTCATAATTATGTTCATGTGGACCATCAGGTTATCTATGATTTAATCAATACTAAAGAATTTCAACGGCTGCGCCGCATCAAACAGCTGGGTACTTCCGGCTATACTTTCCACGGCGGGGAGCACAGCCGTTTTTCGCATTGTCTGGGAGCTTATGAGATTGCTCGGCGCATCACCAAGATTTTCAACGAAAAATACCAAGCCAACTGGGACAGCCATGAAAGCCTACTGACCATGACAGCCGCTCTCCTGCATGACTTGGGACACGGAGCTTATTCACACACTTTCGAGCGCCTTTTTGATACTAATCACGAGGACATTACGCGGCAAATCATCACCAGTCCTGAAACAGAGATTCATCAGGTACTGGTGCAGGTTTCACCAGATTTTCCAGAAAAGGTAGCTAGCGTTATTAACCATACCTATCCCAATAAGCAGGTGGTCCAGCTGATTTCCAGCCAGATTGATGTGGACCGGATGGATTATCTCTTGCGCGATTCCTTCTTTACTGGCGCTTCCTATGGGCAATTTGACTTAACCAGAATTTTACGAGTGATTTGTCCAGTAGAAAACGGCATCGCCTTCAAGCGCAATGGCATGCATGCGGTGGAAGACTACGTAGTCAGCCGCTACCAGATGTACATGCAGGTCTATTTCCACCCGGCCAGCCGAGCGATGGAAGTTCTGCTGCAAAATCTGCTCAAGCGAGCTAAGTTCCTCTATCCGGCCCAGAAAGATTATTTTGCGCTGTCATCGCCCAATCTCATTCCATTCTTTGAAAACAGAGTGACCCTGCAGGATTATCTGGCCTTGGATGATGGTGTTATGAATACCTATTTCCAAGTCTGGATGACTAGTCCAGACAAGATTTTATCTGACTTGGCTCAGCGTTTTATCAACCGCAAGGTCTTTAAGTCTATCGTCTTCTCTCAGGAAAACGAAGGGCATTTGGATATCATGCGAGACCTAGTTGGACAGGTTGGTTTTGACCCTGATTACTATACTGCTATCCATCGTAATTTTGATTTACCTTACGATTTCTACCGGCCTGACGTTGAAAAACCCCGGACTCAGATTGAAATCCTACAAAAAGATGGCAGCTTAGCAGAACTGTCCAGCCTGTCTCCTATCGTCCATTCGCTAGCCGGGACCAGACAGGGTGATAATCGCTTCTACTTCCCCAAGGAAATGCTAGCAGAGACCGGACTTTTCAGCGAAAAAAACCAGACCTTTATGCACTATATCAAAAACGACCAATTTACCTACGGAGAATAATATGTCTATCAAATTAGTTGCCGTTGACATTGACGGTACCCTTTTAAATAACCAAAAAGAAATCACTCCTGAAGTCTTCAGTGCTGTTCAGGATGCCAAAGCTGCTGGTGTCAAAATCGTCATTGCAACCGGCCGTCCTATTGCAGGCGTTCAAAAGCTTCTCGAGGATCTAGAGCTTAATCAGCCAGACAACTATGTCGTTACTTTCAACGGCGGACTGGTGCAGGATACTGTCACAAGTCAAGAATTAATCAAAGAAACACTGACCTATGACGACTATCTAGATATCGAACTGCTCGGGCGAAAATTAGGTGTTCACATGCATGCCATCACCAAGGACGGCATTTATACAGCCAATCGCAACATCGGCAAGTACACTGTTTACGAGTCCAATCTGGTCAGCATGCCTATCTTCTACCGCACACCTGAAGAAATGGTCAATAAAGAAATCGTTAAGTGCATGTATATTGATGAGCCAGAAATTCTGGATGCGGCCATTGCTAAGCTACCACCGGAATTAGCCGAAAAATATACACTGGTTAAATCAGCTCCTTTCTATCTGGAAATTGTCAAAAAGACTGTCAATAAAGGAGCTGCTATCCTTCATCTGGCTGAAAAGCTTGGCTTGAGCAAGGAGCAGACCATGGCTATCGGTGATGAAGAAAACGACCGCGCCATGCTGGAAGCTGTTGGCTCTCCTGTTGTCATGGAAAACGGCAAGGAAGAACTCAAGAAAATCGCCAAATATATCACCAAATCGAACGACGAATCTGGCGTAGCGCACGCTATTAGAGAGTGGGTTTTAAAATAATGTTTAGTTACAAAATTGGAATTTCAGCTCAGGAACACGACGATTTTGTCACTGCTCATCCGCAGGCCAATCTTCTGCAGAGTTCAGCCTGGGCTCAAATCAAGGACAACTGGGAAAACGAACGCTTGGGCTTCTATAAAGACGACCACTTGGTTGCTGCAGCCAGTGTCTTGATCAAACCACTTCCCTTGGGGATGACCATGCTTTATATTCCGCGCGGTCCCATAATGGACTATGGTGACAAGGAGCTGCTGACCTTTGTTTTGGCCTCGCTCAAGAAATTTGCCAAGGAGAAAAAAGCGCTCTTTATCAAGTTTGACCCTAGCCTCTTTCTGGCAGAAAGCAAGATGGGCGGTGAATTGCAAGACAAGGCAGAAACGATTGAGCTGATTCAACAGTTACAGGAAGCAGGCGCTGTCTGGGTCGGACGGACTGAGTCTCTGGACGAAACCATTCAGCCTCGCTTGCAGGCCAATATTCATAAAGAAGATTTCAGCGAAGAGTTGCTTTCTAAAAGTACTCGCCAGGCTATTCGTACAGCCCGTAACAAAGGCATTCAAATCCAATTTGGTGGAGCAGAATTACTGGACGACTTTTCAGGCCTGATGAAGAAGACAGAAAACAGAAAAAACATCCACCTACGCGGCAAAGACTATTACCAAAAACTCTTGGAAACTTATCCTGAGCACTCCTATGTCACCTTGTCCACTATTGATCTAAAGGCACGATTGGAAGACTTGCAGGCTCAGCTGACTAAGACACTCAAGGAAGCAGAGAAATTTACCGAGAAAACCAAGCCTGGCAAGATTGAGAATAACCAGCAAGAACAGAAACGACTTCAGGAAGAGATTGACTTTTTACAGGCCAAAATCAGCCAAGGGGCTGCTATTGTTCCCCTGTCTGGCACCTTGGTCTTAGAATACGGCAAGACCTCTGAAAATATCTATGCTGGAATGGACGAGGAATACCGTCGTTATCAGCCTGCTATCATCACTTGGTACGAAACCGCCAAACATGCTTTTGAGCGCGGAGCAGATTGGCAAAATATGGGTGGAATCGAAAACGACCTCAAGGGCGGTCTCTACAGCTTTAAATCCAAGTTCAATCCTACCATTGAGGAATTCGCTGGTGAGTTTAACCTGCCAACCAATCCTCTTTACCACCTCTCCAATCTGGCCTACACTATCAGAAAGAAACTGCGTAGCAAGCATTAAAAGAAAGGAAGCCTATGACCTTTAAACTTCTCAGTCAAGAAGAATTCATCCAGCATACCTCAGCTAGCTCCCAACGCTCCTTTATGCAGACCGTGGAAATGACAGAGCTGCTGAGCAAGAGGGGCTTCAATACCCAGTATGTCGGCTACACTGACCCACAGGGACAGGTAGTGGTGTCAGCTGTCCTCTACAGCATGCCTATGACTGGCGGCCTTCATATGGAAATCAACTGCGGTCCTGTCTCTACCGATGCTCAATACCTGACCCCCTTCTATCAAGCTTTGCAAGCCTATGCTAAAAAAGAAGGCGCCCTAGAGCTCATTATCAAGCCCTATGAGACTTATCAGACTTTTGACAGCAATGGCCAACCCACATCTGAAGAAAAAGGCGAGCTTATCCAGCAGCTGACTGACTTAGGCTTTGCCTTTGATGGCCTACAGACAGGCTATCCAGGCGGGGAGCCTGATTGGCATTATGTCAAAGACCTAGCTGATCTGACGGAGAAAGACTTGCTCAAGTCCTTCAGTAAAAACGGCAAGGCGACCGTCAAAAAAGCTAACACCTTTGGTATTAAGCTAAAAAGGCTGGAACGTGACCAACTCAGCATTTTTAAAGACATCACAGCTGCCACTTCTGACCGGCGGGAATATGATGACAAGCCACTAGACTACTATCAAGATTTTTATGATAGCTTTGGTCAGCAGGCAGACTTTATGACTGCTAGTCTCAACTTCAAGGACTATCTTCAGAACTTACAAAAGGATCAAGAGAAACTGGGCCAAAAAATCCAGAAACTGCAGGCGGACTTGGAAAAGAATCCTCAATCTGAAAAGAAACAAAATCAGCTACGAGAGCTTTCCAGCCAGTTTGACAGCTTTGAAACCCGCAAAGCTGAAGCCCAAGAACTGATTGATAAGTACGGCGACCAAGACCAGATCCTGGCTGCCAGCCTCTTTATCTACACCCCCCAGGAAGCAACCTATCTCTTCAGCGGCTCCTATCCTGAGTTTAACAAATTCTACGCCCCTGCCCTGCTGCAGGAATATGTTATGACCGAAAGCATCAAGAGAGGCATTCCATTTTATAATTTCCTCGGCATTATGGGAATTTTCGACGGTTCTGACGGTGTTCTGCGCTTCAAACAAAACTTCAACGGCTTCATCGTTCGAAAAATGGGAACTTTCCGTTATTATCCTAACCCTCTCAAGTTTAAGCTGCTTCAGCTCATAAAGAAAAT

>c183_g145

TTTATCTATAACGAGGTGTCGCAAAACCACGGATATTTCCATGACCGATGTGGTAAGTATTGCGTTTAACTACGTTATTGCTGTTACCTTCAATCGTGTGAATGATCCCATTTTCGACTTTTTCGACAATACCGATATGATCAGCCCAGCCATCGTTTTGCTGGGTATCTTTGTCCCAGTTAAAGGTGATGATATCACCTGCGCTAGGAGTCGAATTGCCATCTTCATTCCAGATACCTAGTCTTTGGAAAATATGAATATGGCGCTCTACACCGCACTCACGGCCGATAAGATCGCTCAAGCCTTCTCTTTGGAAAATAACCGTCGTGAAAATGTCGCACCAGTCATCTGTGTTTTTCACAGCATAGCCAACTGGCAGCGGTCTGACACTGTTATAATCGTTGACCAAGCGCTGATGCTCAGCACTTCCTCCCCTGACACCTACCATAGCAGCTGCCGCCGCAAGAACTCGATCTCGCTGACTAGCTGCCTGAGGACGGTTCACAGAGATAGAGGTCTTTTCTCCACCAGCTCCTTGCAGCTGATTTTGATTATTTAAGTAATACAAGTGAACATTGTATTCGCCTGCATTATTCCTATGGTCACGCGCATATACATGCTTACGATAAGTTCCATCAGCCTGACGATCCGCTGTGTACCATTGGATATCATCTTGACCATTCGCTTCTGACCAAGTCGGCAGGTAGACTGTCTTGAGGCCTTCTGGTGCCACAATGCCTGATACTACAATGTCAAACTCTCCAGTATCGTTATTCTTATTTTGAATACTGATTTTACCCTGCGGCTTACTGATAGAAACGTTGGTCTTGATGCCACCTGCTCCTACCAGCCTGCCATCATTTTGCACATAGTAGAGATGGACATTATATTCGCCCTGAACGTTGTTATGGTCGCTCGCATGAACTCGTTTCCGATAGGTACCATCCGGCTGACGTTCTGCGTTGTACCACTTGATATCATCCTGACCGTTGGCCTCTGACCAAGTTGGTAGGGAAACATTCTTGAGACCGCCTGGCGATACGATATCTGAAACCACAATATCAAAATCG

>c183_g146

TGTCGGCAAGGCACATAGTAAAATTATTTTAATGGGAGAGCATTCGGTGGTCTACGGCTATCCGGCCATTTCCCTGCCCCTTAATCGTATTGAAGTGACCTGTCAGGTCTTTCCGTCTGAGCGGGCCTGGACCCTCTACGCCGAGGATACGCTGTCTATGGCTGTTTTTGCCTGTCTAGAACATCTAGGCCAGCAAGGAGCTAAGATACGCTGTCAGGTGGAGTCCATGGTCCCTGAGAAGAGGGGTATGGGGTCTTCAGCCGCGGTCAGTATTGCGGCTATTCGGGCTGTCTTTGACTACTTTGAGGAAGAACTGGATGACCAAACACTGGAGATTCTGGCCAATCGAGCAGAGATGATTGCCCATATGAATCCCAGTGGCCTTGATGCCAAGACCTGCCTCAGTGATGTAGCTATTAAGTTTATCCGCAATTTTGGTTTCAGTGAAATTGAGCTGGATTTGGATGCCTTTTTAGTCATTGCAGATACGGGCATTCACGGTCATACCCGCGAAGCCATCCGTGCTGTAGAAAGTCAGGGCCAAAAGGCTCTGCCTTTGCTGCAGGAATTGGGGAATTTAACGAAAATCCTTGAGAAAGCTATTTTTATCAAGGATCTGATGACAATGGGGCAAGCTATGACCAAGGCTCATGAGAAGCTAGCCAAGCTAGGAGTGTCTTGCCAGAAAGCGGATGAGTTGGTAGCGGCAGCTCTTGAAAATGGGGCTTTGGGTGCTAAAATGAGCGGTGGTGGTCTGGGCGGCTGTGTCATTGCTCTTGTAGGAGAAAAAAGTCAGGCGGAGGCCTTAGCCGCCTTATTGAGAGAGAAAGGGGCCATTAACACATGGATCGAAAGCCTGTAAGTGTCAAATCCTATGCCAATATTGCAATTGTCAAATATTGGGGGAAGAAAGATGCAGAAAAGATGATTCCGTCTACCAGCAGTATCTCGCTGACACTGGAAAATATGTATACCGAGACGCAGCTGAGTCCTTTGCCGGATACAGCGACTGGAGATGAGTTTTATATTGACAGCCAGCTACAAAGCCCGGCAGAACATGCCAAAATCAGTAAGATTATTGACCGCTTCCGCTCTCCAGAAGATGGTTTTGTCCGCGTTGATACCAGCAATAATATGCCAACAGCAGCGGGTCTGTCTTCCAGCTCCAGTGGTCTGTCTGCCCTAGTCAAGGCCTGCAATGCTTATTTTCAGACGGGTTATCAGACGCAGGAGCTGGCTCAGCTGGCTAAGTTTGCTTCAGGATCGTCTGCTCGCTCTTTCTTTGGTCCGCTAGCGGCCTGGGATAAGGACAGCGGGGCCATTTATCCAGTCAAGACGGATTTGAAACTAGCCATGATTATGTTGGTTCTGCACGATGAGAAAAAGCCCATTTCCAGCCGCGACGGTATGGAGCTTTGTGCTAAAACTTCCACCATTTTCCCAGATTGGATTGCCCAGTCTGCCTTGGATTATAAAGCTATGTTAAGCTATTTGCAGGACAATGATTTTGCTAAGGTAGGTCAGCTGACAGAAGAAAATGCTCTCCGGATGCATGCCACGACAGAAAAAGCTTATCCGCCGTTTTCTTATCTGACAGAGGAGTCTTACCAGGCTATGGATGCTGTCAGAAAGCTGCGGGAGCAGGGCGAGCGTTGCTACTTTACCATGGACGCAGGGCCAAATGTCAAGGTGCTCTGCTTGGAGGAAGACCTAGACCATCTGGTTGCTATATTTGAGAAGGATTACCGGCTTATCGTCTCCAAAACAAAGGACTTGTCAGATGAAGACTAGTGCAAGAGTCCAAACCTGTGGCAAGCTCTATCTGGCGGGTGAATATGCAGTGCTGACGACCGGTCAGCCGGCCATTATCAAGGCCATTCCTATCTATATGACGGCGGAAATCCAGGCAGCCTCTGCCTATCGCTTGACATCAGATATGTTTGAACACAGCGCTAGCCTAGAGCCAGACCCTGATTATGCCTTGATTCAGGAGACAGTAGCAGTTATGAATGATTATTTGCAGGTCTTAGGCTACCAACTTCAGCCTTTTTCTCTGAAGATTAGTGGCAAGATGGAAAGGGACGGCAAGAAGTTCGGGATTGGCTCCAGCGGTAGTGTTGTTATTCTGACCATTAAGGCCATGGCAGCACTTTATGAGCTAGACTTGGAGCCAAAGCTACTCTTTAAGCTGGCTTCTTACGTTCTCCTCAAGCGCGGAGACAATGGCTCCATGGGAGATCTAGCCTGCATTGCTTTTGAAGACCTGATTTACTACCGGTCTTTTGATAGAGAGCTGGTCCGCAAGCGTATGGGCAAAGTTTATTTACAGCAATTACTAGCAGAGGATTGGGGTTTTGAAATTCGTAGTATTAAGCCTTGCTTAGCCATGGATTTTCTAGTTGGCTGGACCAAGCAGCCGGCTATCTCTAAGGACTTAGTCAATCAGGTCAAGTCAGCTATTTCAGAGTCCTTTTTGACAGGCAGTAGGACGCAGGTTGATGCCTTGGAGAAGGCTTTATTAGCAGGAGATAAGCTTGCTATTCAGTCTAGTATGGAAAAGGCTAGTCAGCTCTTAGAAACACTCAGTCCGGCTATCTATACAGACAGGCTGAAAGTCTTAAAAGAAGCGGCAGAGGGACTGAACTGTGTGGCTAAGAGCAGTGGTGCCGGTGGTGGCGACTGCGGGATTGCTCTCAGCTTTGATGTCGCATCTAGCAACCAACTGATTCAAGCCTGGCAAGAAGCTGGCATTGAGCTATTATACAGAGAAAGGATGGGCCATGATGAGCCAGAATCGTAAGGATGACCATATCAAATACGCTTTAGAGCAGCGTCCGGGTTACAACAGTTTTGATGAAATGGAACTGGTTCACCGTTCTCTGCCCAAGTATGATTTGGCAGAGATAGACCTATCTACTCACTTTGCTGGCCGTGACTGGGAGTTTCCTTTTTACATCAATGCCATGACTGGCGGTAGCCAAAAAGGTGGTCAAATCAATGAAAAACTGGCTCAAGTAGCTGAAAGTTGTGGCCTTCTTTTTGTGACTGGCTCTTATAGTGCAGCCTTGAAAAATCCTTCTGATCCTTCCTATCGGGTGGCGACTGGTCGGCCTAATTTATTGCTGGCTACCAATATCGGCTTGGACAAGCCTTTTCAAGCCGCCCAGCAGGCAGTAGCTGATTTGCATCCCCTCTTTTTGCAGGTCCATGTCAATCTCATGCAGGAATTGCTGATGCCAGAGGGCGAGCGAGAGTTTCGTTCTTGGCGCCAGCACTTGGCAGATTATAGTCAGCGGCTGGATCTTCCTCTAATTCTTAAAGAAGTTGGCTTTGGCATAGATCGCTCTACTGTTGAAGAAGCGCGCTCCTTGGGGATTCAGACTTTTGATATTTCCGGCCGTGGTGGCACTAGCTTTGCCTATATTGAAAATCAGCGGGGTGGCAATCGTGACTACCTTAATGATTGGGGTCAATCTACTCTGCAAAGTCTGCTAGCTCTTCAGCCATTGCGTGATGAAGTCGAACTCTTGGCTAGTGGAGGTGTCCGCCATCCTTTAGACATAATCAAAGCTCTGGTCTTAGGAGCCAAGTCGGTTGGCCTTTCTCGCGCCATGTTGGACTTGGTGGAAAATCACTCAGTGGAAGAGGTTATTGATATTGTAGAAGGCTGGAAATCGGATTTGCGGCTTATCATGTGCGCCCTATCCTGCCGAAACTTGCAAGAATTAAAGAGTGTACCTTATCTACTCT

>c183_g147

TTTCAAAATCAGAAGTCTTATTCCTCTTCTGCTTTCGCTAATGCTTCTCTTTCCAGACGCAGCTTGCGTTTGGGGTTGAGTTTATTAAAGAGCTCTTCTAGTTTTTCTGCATTCCAGACGTCGGCTGCAGAGACAAAATTTCCATTTTCATCGCGGAAGGATATTGGTTTATCACCCATTGCAAGCCTCCTTAGTCAACAAATTCAAACTCAAACTTGCCAATACGAACCAGATCTCCATCCTTGGCTCCGCGATCACGAAGGGCTTCATCAACTCCCATACCGCGAAGTTGGCGGGCAAATTTCATGACTGCTTCATCGCGGTCAAAGTTGGTCATACTAAAGAGTTTCTCAAGCTTGTCACCAGACAGTACCCAAGTCGCGTCATCATCACGAGAAATTTCAAAGGCTGGTGCTTCTTCGTCAAAGCCATAGTAAGCTTCTTCTTCCATTTCAGACTCATCATAAAGCAAGAACTCTGGTGTCTTATCCAGTAACTCTGCAGTCGCATCCAAGAGAGTCGCAAGTCCTTGCTTAGTCAGACTGGAAATCGGGAAGATTTGCGGTAGTTCCGCAAACTCATCATAATTAGCTGCTAACTTCTCTTTGAATATCTTAAGATTTTCAGCGCTATCTGGCATATCCATCTTGTTGGCAACAATAATCTGCGGGCGCTCCATGAGACGAAGATTGTAGGATTCCAACTCTTTATTGATAGCCAGATAGTCCTCATAAGGGTCACGTCCCTCACTAGCTGACATATCGATGACATGCAGGATGACCCGTGTCCGCTCAATATGACGGAGAAACTGGGTTCCCAGTCCCACTCCTTGGCTAGCCCCCTCAATCAAACCCGGCAGATCTGCCACCGCAAAGGACTCTCCAGAATGAGTGCGAACCATGCCTAGATTGGGCACGATTGTCGTGAAATGATAGGCACCAATCTTAGGCTTGGCTGCTGTAATAACACTAAGCAAAGTGGATTTCCCGACAGATGGGAATCCGACCAGACCAACGTCTGCTAGGACCTTGAGCTCCAGCAGAAGCTCTCGCTCTTGACCCGGTTCTCCGTTCTCAGAAATCTCTGGAGCAGGATTTTTAGGCGTTGCAAAGCGAATATTTCCACGACCGCCTCGGCCACCACGCGCTACGATAAATTCTTGACCATTTTCTACCAAGTCCGTCAGCACCTTGCCCGTCTCCGCATCACGAACTGTCGTCCCCTGAGGCACACGAACAATCAAGTCCTCTGCCCCTCGGCCATGCATCCCTTTGGTCATGCCCTTTTCACCAGACTGAGCCTTGAAGTGGCGATTGTAACGAAAGTCCATCAGGGTACGCAGGCCCTCGTCTACAACAAAAACAACATTGCCGCCACGGCCACCATCGCCACCCCAAGGGCCGCCGTTAGGAACATATTTTTCACGGCGAAAGGCCACCATGCCATCACCACCATTGCCAGCCTTAACTTGAATCTTGGCCGTATCTAAAAACATACTCATTTTTTCTATTCTCTTTTTCTAGTCTAAAAAAACGCCCTGAAGCGTTTGGATTAAAAGATTGCGCTGAGAGCAGAAGCAAAAATCGCTCCGACTGTCACGATAAGCATGATAATCACGACCAGCATCGTCAATTTTTCA

>c183_g148

GTTTATGATACTGTGTTAAAATACTAAACAATAAGAAACCATCTCAAAAAGGAGTTAGTCAAACATGAAGTCAACCCAATCTTCAAACTTTGCTTTGTTGTTGCGTTACTCGGGCTAGTGCAAAAGCATTAGTCCTGTTTGGCTTACCAAGCGGGAGTAAATCAACATCTCGCTTGTTCCTCAAGTGAGATGTTTTTTATTTCACCTATCTTACCTCAAGCTCTGATGAGGAAGAAATGGTTTCTAAAGGAGCGACGGGAGTGGGGCTACATCTTGATTTTTCTGAAATCGATTTGTCTCAGACCGTCTCAGTCATTGAACACGACCTAGAAGTTTGGAAAGAATTTTTCCTAATTGGTCTATGTATCTTAGAAAAGGAGTTGTCTATGACACGCAAAGTTGAGTTTTTTGATACCAGCCTCCGGGATGGCGAGCAGACACCAGGAGTGAATTTCTCTATCAAGGAGAAGGTGGCAATTGCCAAGCAGTTAGAGAAGTGGGGGATTTCAGTCATTGAAGCTGGTTTTCCTGCTGCGAGTCCAGATTCTTTTGCTGCGGTGCAGGAGATTGCTAAAGTCATCACAAAAGCTTCTGTTACTGGGCTGGCGCGTTCGGTCAAGTCCGATATTGATGCCTGCTATGAAGCGCTCAAGGATGCTAAGCATCCACAGATTCATGTCTTCATTGCGACCAGTCCTATTCACAGAGAGTTTAAGCTGAAAAAGTCTAAGGAAGAGATTTTAGAAGCTGTGAAAGAGCATGTCTCCTACGCGCGCTCCAAGTTCGATATAGTCGAGTTTTCACCAGAGGATGCGACACGAACAGAGCTGGATTTCCTTTTTCAGGTGGTACAAACAGCAGTTGATGCAGGTGCGACTTATATCAATATTCCAGATACAGTCGGCTTTACTACTCCGGAAGAGTTTGCAAATATTTTCGACTACCTAGTGGCAAATGTGACTTCTGATCATAAGGTGGTATTTGGTGTTCATTGCCATGATGATCTTGGTATGGCAACGGCTAATAGTTTGACAGCTATCAAGCACGGTGCTGGTCGTGTTCAGGGAACTATCAATGGAATCGGTGAACGGGCTGGAAATGTAGCATTGGAAGAAGTAGCAGTTGCCTTAGAGATTCGTCAGGACTATTACCAAGTTGAGACAGACATTGTCCTTAATGAAACCATCAACACTTCTGAGCTGGTATCCCGCTTCTCTGGTATTCCAGTGCCTAAGAACAAGGCGGTCGTTGGTGGCAATGCCTTCTCTCATGAGTCCGGTATCCATCAGGATGGTGTTCTTAAGAATCCACTGACCTATGAAATCATCACACCTGAGCTGGTTGGAGTCAAGAGCAATTCACTGCCGCTTGGCAAACTGTCTGGCCGTCATGCTTTTGTCGAAAAACTCAAAGAATTGGCTCTGGATTTTGCGGAATCTGAAATCAATGATCTCTTTGCTAAGTTTAAGGTCTTGGCTGATAAGAAAAATGAAGTCACGGATGCTGATATTCGGGCTCTGATTGCTGGAACGACGGTCGAAAATCCTGAAGGCTTCCACTTTGATGATCTGCAGTTGACGACCAATGACGACCATACCATCACAGCGGATGTGCAGCTGGTTAATGGTGACGGCGAGACGGTCAGCTGTGTGGCAGAAGGTAAAGGAAGTGTTGAAGCTATCTTTAATGCCATTGACCAATTCTTTAACCAGTCTGTTCAGCTTTTGTCTTATAACATCGAAGCGGTAACGGATGGTATTGACTCTCAGGCTCGCGTTTTGGTAGCTGTTGAAAATACGGATACAGATACAATTTTCAACTCCTCTGGTATTGACTTTGATGTTCTTAAGGCCAGCGCCATTGCCTATATCCATGCCAATACCTTTGTGCAAAAGGAAAATGCTGGTGAAATCGGCCATCAGGTATCTTACCGCGATCTGCCTGCAAATAATTAGAAAGAAGCGACTATGACAAAAGAAATTGTAGCTCTGGCGGGTGATGGTATCGGTCCAGAAATCATGGAGGCTGGTCTCCAAGTGCTGGCTGCTGTTGCGGGCAAGTTCGGCTTTACTTATCATATCACGGAGAAGGCTTTTGGGGGTGCTGGTATTGATGCGGAAGGTCATCCTCTGCCTCAGTCTACTCTAGAAGCGGCCAAGAAGGCGGATGCTATTCTCCTAGCAGCTATCGGCAGTCCCCAGTATGATAATGCCCCAGTTCGACCAGAGCAAGGCTTGCTCGCTCTGCGGAAGGAGCTTGAACTCTATGCCAATATCCGCCCAGTTAAGATTTTCGATGCTCTTAAGCATTTGTCGCCTTTGAAAGCTGAAAGAATTGCTGGTGTGGATTTCGTGGTCGTGCGTGAGCTGACTGGTGGGATTTACTTTGGTGAGCATATTTTAGAGGATAAATCTGCGCGGGATATCAACGACTACAGCTATGAAGAGGTGGAGCGGATTGTCCGTAAGGCCTTTGACATTGCACGTGGTCGCAGAAAGCGCGTGACTAGTATTGACAAGCAAAATGTGCTAGCGACATCCAAACTCTGGCGTAGAGTAGCAGATGAAGTGGCCAAGGATTATCCAGATGTGACCTTGGAGCACCAACTGGTGGACAGCGCTGCGATGCTCATGATTACCAATCCTGCTAAGTTTGACGTTGTGGTAACGGAAAATCTCTTCGGCGATATTCTCTCAGACGAGTCCAGCGTCTTGTCAGGAACGCTGGGAGTTATGCCATCGGCCAGTCATTCGGCTGCAGGACCCAGTCTTTACGAGCCTATCCATGGTTCGGCTCCAGATATTGCAGGGCAAGGTATTGCCAATCCTATCAGCATGATACTTTCTGTGGCCATGATGCTGCGGGATAGTTTTGCAGAGCTTGAGGCTGCAGAGGCGATTGAAGCAGCGGTTGAGAAGACTTTGGCTCAGGGTATTTTAACCCGAGATCTTGGCGGTCAGGCAGGGACAGCTCAAATGACGGAGGCAATTATTAATAACTTATGAAATGGATATTAACAGGTATTTGCCTGCTATGGAACCTTGTCGTTTTCCTGCTTTATGGCTGGGATAAGCGTAAGGCTAAGAAAAATCACTACCGCATTCCAGAGAAAACCTTGCTCTTGTCAGCTTTGGCAGCCGCAGGTTTGGGCGCTTTATTAGGCGGTCGCCTCTTTCATCACAAGACCAGAAAATGGTATTTCTGGCTGGCTTGGATTTGCGGAATAATAGTGGGAATCGGAATTTTATATTACATATGGAGAAGCTAGTATGGCT

>c183_g149

CTCTTGGCAGGCAAGTCTTCCCACTTTGTTCTCAGCAAAATGGGACGCGGTTCGACTCTGCCGGGGAAAGTTGCCCTGACATTTGATAAAAATATTTTGCAAAACCTAGCAAAGAACTATGAGGTCGTGGTTATTACCGGAACCAATGGTAAAACTCTGACTACAGCTCTGACAGTGGGTATTCTCAAGGAAGCCTTTGGAGAAGTGGTGACCAATCCTAGCGGTGCCAACATGATTACGGGGATTACGACAACCTTCCTGACCGCTAAGAAAGGCAAATCTGGCAAGAATATCGCTGTGCTGGAAATAGATGAAGCCAGCCTATCTCGGATTTGTGATTATATCAAGCCTAGCCTCTTCGTCTTTACCAATATTTTCCGCGACCAGATGGACCGCTATGGTGAGATTTATACGACCTACCAGATGATTCTGGATGCTGCAGCTAAAGTGCCTGAAGCGACTGTACTGATGAATGGTGACAGCCCCCTCTTTAACTCAGTCAGTCTGAAGAATCCTGTCCGCTACTATGGATTTGATACCGAGAAAGATCAGGCTCAGCTGGCTCACTACAATACAGAGGGCATCCTCTGTCCCAAGTGCGAGCATATCCTCAAGTACGAGCTCAACACTTATGCTAATCTGGGGGCTTATATCTGTGAGGACTGCGGCTTCAAGCGTCCTAAGCTTGACTACAGCCTGACCGCTCTCAAAACACTTGAGCATAACCGCTCGGCCTTTACCATTGATGGTCAAGACTATCAAATAAATATCGGCGGTCTCTACAATATCTATAACGCTCTAGCAGCGGTATCAGTAGCTCAGTTCTTTGGTGTCGAGCCAGCTACTATCAAGGCTGGCTTTGACAAGAGCCGGGCTGTCTTTGGCCGTCAGGAAACCTTCAAAATTGGCGATAAGGAATGTACCTTAGTCTTGATTAAAAATCCGGTCGGAGCCACCCAAGCACTGGATATGATTGGGCTTGCGCCTTTTGACTTTAGCCTATCCGTTCTGCTCAATGCCAACTATGCAGACGGCATTGATACCAGCTGGATCTGGGATGCTGATTTTGAGAAAGTTCTAGAGATGGAGATTCCTCATGTCATTGCAGGCGGTGTGCGCCACTCTGAGATTGCTCGTCGACTGCGGGTAACGGGCTATCCAGCAGATCAGATTACTGAAGTCAAAGATTTGGAAGCAGTCTTTAAGACCATTGAGCAGCAAGAAACCAAGCATGCCTATATCTTAGCAACTTATACCGCTATGCTGGAATTTCGCGAATTACTGGCAGAACGACAAGTGGTCAGAAAGGAGATGAACTAATGGTATACAGTTCCCTCACTTCTCCTGAAAATCAGGACTATTGCTATGATATAAAGATTGCTCATCTCTATGGCAATCTCATGAATACCTACGGCGACAACGGCAATGTCCTCATGCTCAAGTATGTGGCTGAAAAGCTAGGTGCTAGGGTTGAAGTCGATATCGTCTCTCTAGAAGATGACTTTGACAAGGACAGCTACGACATCGTCTTCTTTGGCGGAGGTCAAGACTATGAGCAAACGATCGTGGCTCGTGACCTGCAAGCTAAAAAAGAAGCTTTGGAAAGCTTTATCAATGAAAACGGCGTAGTGCTAGCTATCTGCGGTGGGTTCCAACTCTTAGGCCAATACTATATCGAAGCTTCTGGTCGACGAATCGAAGGCCTGGGCATCATGGGTCACTATACCCTCAACCAGACCAAAAACCGCTATATCGGTGACATCAAGATTCATAACGAAGAATTCAATGAGACCTACTACGGCTTTGAAAATCACCAAGGACGGACTTTCCTCTCGGACGATGAAAAACCTCTGGGCAAAGTCGTCTATGGAAATGGCAACAACCAAGAGGATGGTTGCGAGGGTGTTCATTATAAAAATGTCTTTGGCTCCTACTTCCATGGCCCTATCTTGTCCCGCAATGCGAACTTGGCCTACCGTCTGGTGACCACCGCTCTGAAAAATAAGTATGGATCAGATATTAAACTAGCAGCTTATGAAGATATCCTAGCCCAAGAAATCCCAGAAGAATATGGAGATATCAAGAGCAAGGCCGAGTTTGAATAGATAAACAGGAGAATCACCATGAAAGAAAAATTGCATTATATTCTGCTTTTAATTACCATTCTATTGGTTGCTGGTATTTCCTTAGCCAATATGCAGAGCGTCAATGTTAGCTTTATCTTGTTCAGCTTCAAACTTCCCTTAATCATATTGATTTTGGTCTCAGTGCTCCTAGGCTCTGTCACGACCTTTCTCATCAGCATGTTAAAAAATTTCTCGCTGAAAAAAGAACTTAAAA

>c183_g15

GTCCACTCTGGGAATCAGCTTAGCTGTTTTGGCTCTTTTGGGAACCCAGCTAGTCTTGCCGGCTTCTATGCCTTTTTACAGTGACTGGAGAGCTTATGCTGGATTGATTGTCCTGATTGTTTTCATGTCACTGGCTGGCGGACTCTTGTCCATTCACCGGGTTCTGAAAATTGACCCAATTACAGCGATTGGAGGTGAGTAAATGTCAATCATAGAACTAAAAGGAGTTACAAAAGAATACGGTCAGGGGCATACGCTGGTCCAAGCTCTGAAACCAACTGATTTCCAACTGGAAGCGGGGCAGTTTGTAGCTATTATTGGGCCGTCAGGCTCGGGTAAAACAACCCTTCTGACCTTGCTGGGGCATTTGCAAACTCCCTCAAAAGGACAGATTCTGCTGCATGGGAAAGATACTTCCCAGCTCAAGGAAAAGGAACGGGCTGCTCTACGCTTCAATGATTTCGGCTTTATTTTGCAGGCTTCCAATCTCATACCTTTTTTGAAAATCGAAGACCAGTTCCAGCTGATTGACCGCTTATCTAAAAAGGAAAGGACGGATCTAGACAGTCTGATTGAGTTGCTGGATCTTAAAGGAACTCTCAAGCAGTATCCGAAAGAATTGTCTGGCGGAGAACGGCAGCGGGCTGCAATCGCCCGAGCACTTTACAACAGCCCTGATATTATCTTGGCTGATGAGCCGACAGCTAGTCTGGATACGGAGCGGGCTAAGCGCGTGGTCCATCTGCTCAAAGAGGTGACCCAGAAATTCCATAAGAGCGTGGTCATGATTACTCACGATACCCGCTTGCTGGACGAGGTTGACAAGGTCTATGAGATGCAAGACGGAGTGCTGACTCAAGTTCGATAAAGTTTTAGAAGTGATTTGCAATCAAACAGAGAAAAAACAAGCAGAGGACCATGATTCATTATAAAATAAATCCGCAGCTAGATTTTGCGGCAGTGCTAGACCTCTATGATTCGGTTGGTTGGAGCAATTACACCAATCGTCCTCAGCAGTTAGAGCAAGCCTTCCATCAGTCCTTGTTTGTGATGGCGGCCTATGACGATGAGGAGTTGGTTGGTTTGATCCGAGCGGTCGGAGATGGACTTACCATTGTCTTTATTCAGGATTTGCTGGTGTATCCACACTACCAGCGTCAGAGGATTGGCCGAAGCCTTCTTCAGCAGACGTTGGAAAGATTTAAGGATGTTTACCAAATCCAGCTGGCAACCGAGCAATCAGATAAAAATCTAGCTTTTTATCAAGAGCTCGGCTTTCGCCGACAGGAAGATTTCGACTGTA

>c183_g150

CTATGATTTAATTGTCATCGGCTTTGGGAAGGCCGGTAAAACATTGGCAGCCAAAATGGCGTCCCAAGGAAAAAAAGTTGCTTTGATTGAGCGAAGCAAGGCTATGTACGGGGGAACCTGTATCAATATTGCCTGCATCCCAACCAAGACCCTGCTTGTCGCAGCTGAAAAAGGCCTGGCTTTCGACCAAGTCATGGCTGAAAAGAATGCTGTAACCAGCCGTCTCAACGGGAAGAACTACGCAGCAATCAGTGGCGCTGGTGTTGACATCATCGATGCGGAAGCTCATTTCCTTTCCAATAAAGTCATCGAAATCACTGCCGGCGATGAGAAGCAGGAACTGACTGCTGAAACTATTGTCATCAATACTGGTGCTGTTTCCAATGTCCTGCCAATTCCTGGACTGACTGAAACCGAACATGTCTATGACTCAACTGGCATCCAAAATCTGAAGGAACTTCCTAAACGCTTGGGAGTTCTGGGTGGCGGTAACATCGGCCTAGAATTTGCTGGACTCTACAACAAATTGGGCAGTCAGGTGACTGTGCTGGATGCTGCTCCTGTCTTTCTCCCTCGAGTAGAGCCTTCTATCGCTGCTCTAGCTAAGCAATACATGGAAGAAGACGGAATCCAACTCTTACAAAATGTACGTACCACACAGGTCAAAAATGATGGTGACGAAGTTGTAGTTGTGACAGAAGATGGAGAATTCCGCTTTGATGCCCTTCTCTATGCTACAGGCCGTAAGCCGAATATTGAACCACTGCAGTTGGAAAATACAGATATCGAGCTAACAGAGCGCGGAGCGATTAAGGTCAATAAGCACTTGGAAACATCTGTACCTGGCGTATTTGCAGCGGGCGATGTCAATGGCGGTCTGCAGTTCACTTATATCTCATTGGATGACTTCCGTATCCTTTATAGCTATCTGGCTGGCGATGGCAGCTACACACTGGAAGACCGTAAGAACGTCCCTACCAGCATGTTCATCACACCACCTTTGGCTCAAATCGGATTGACTGAAAAGGAAGCCAAAGAGCAAGGCCTACCGATTGCAGTTAAGGAGATTCCAGTCGCAGCAATGCCTCGTGGTCATGTCAATGCTGACTTGCGTGGTGCCTTCAAGGCCGTTGTCAACACTGAAACCAAGGAAATTGTCGGGGCAACTATCTTCTCAGCGGGAGCTCAGGAAATTATTAATATCCTGACAGTAGCCATGGATAATAAAATTCCTTACACTTACTTTAGCAAGCAAATTTTCACCCACCCAACGTTGGCTGAAAACCTTAATGATTTATTTGCTATCTAATTTTAAGCCCC

>c183_g151

TCTCGGCTTTGTTACAAAAAATAGGACAGATAAATCCAGCATCTAGCCTGATCTTCTGTCCTAGTTTTATTTCTTATGAGCTTCTTGGAATAAACTTGCTGAGTGAGCCTGCAAAGGTTTGAAGATTGAGACTTTGCACATAGACTTCACCAGTACCTTGGAAGGTGTTGACCACTCCTTCTCCCGTTCCAATAGACTGCCAGAAACCATTTTCCAGATGGATATTATAATTCAAAGACTGGCTCCAAGCTACTACATGGGCATTGTCAATCGTCACTTCTTGGTTATGAAGCTCAATTTTCTTAATAGATCCAAAGGCATTAGCCAACAGAGTGCCCTGACCTTGAGTTGTCATAACGAAGAGACCCCCTTGCCCTCCAAAGATAGCCTTGCCGACAGACTGACGCTCCATCGTATAGTAAGCTGTGCCATCAAGAGCCAGGAATGCGCCGTCATTAAGTCGATATTGCTTTTCGCCTAGCTGAAGAGGAATGACTTGGCCAGGAGAATCTGGCGCTAAAGCAAGGTAGCCATTATCAGACTGGGCAACAGCCTGGGTAATGAAGGTACTTTCACCAGAAACCATAGAACGCCCCACAGCCTTGACAAAACGTCCCAAGCCAGAACCGCTTGCATTGAGCTGGGTATTCAGGGTCACATTCGGCGTGTGGTAGACCATACTGCCACGCTGGATAAAGACGGTTTCTCCCTGATTGAGCGACAACTCAACCAAAGGAAACTGCATATTACTGTCCATAGAAAATCGCATTGA

>c183_g152

CAACAATAGCTGGTAAAAGCGGAACGAAAACATCACCAAACGTACGGATGGCGCGTTGGAACCAATTCCCTTGCTTAGCGGCTTCTACTTTCATATCATCTTTTGATGAAGTTGGTAATCCTAAGGCTACAACTTCGTCATAGATTTTATTAACCGTACCGGTACCAAAGATAATCTGGTATTGGCCTGAGTTAAAGAAGGCACCCTGAACTTTTTCCAGATTTTCGACAGTATTTTTGTCGATTTTACCTTCATCTTTAACCATCACGCGTAAGCGAGTCGCACAGTGAGCCACACTGTTAACATTCTCACGTCCGCCAAGGGCTTCGATGAC

>c183_g153

ATCGCATGGGATATACAGTTGCTGTAGTAGGTGCCACTGGTGCCGTTGGATCTCAAATGATTAAAATGCTGGAAGAATCTAGCCTTCCAATTGATAAAATACGCTATCTGGCGTCTGCTCGTTCAGCTGGAAAAGTTCTCCAGTTCAAAGGACAAGATATTACTATCGAAGAGACGACTGAGACGGTTTTTGAAGGTGTAGATATTGCTCTCTTCTCAGCTGGAGGTTCTACCTCAGCAAAATTTGCTCCTTACGCAGTCAAAGCTGGAGCAGTAGTGGTCGATAACACCTCTTATTTCCGTCAAAATCCTGATGTTCCTCTGGTTGTTCCAGAAGTCAACGCCCATGCCTTAGATCAGCACAAGGGAATTATCGCTTGCCCTAACTGCTCGACAATTCAGATGATGGTAGCTTTAGAGCCTGTTCGTCAAAAATGGGGTCTGGAGCGCATCATTGTGTCTACCTATCAAGCGGTTTCTGGTGCAGGCATGGGAGCCATTCTTGAAACCCAAGCACAATTACGCTCTGTTCTCAATGATGGTGTAAATCCTAAGGATGCAGAAGCCAATATCCTGCCGTGTAGCGGTGATAAGAAACATTATCCTATTGCCTTTAATGCCTTGCCTCAGATTGATGTCTTCACAGATAACGACTACACTTATGAAGAGATGAAGATGACCAAGGAAACTAAGAAAATCATGGAAGATGATTCTATTGCTGTTTCTGCGACATGTGTCCGTCTTCCGATCTTGTCTGCTCACTCAGAGTCTGTTTACATCGAGACTAAGGAAGTCGCACCTATTGACCAGGTAAAAGCAGCAATCTCAGCTTTCCCAGGTGCTGTTCTGGAGGATGATGTAGCTCATCAGGTTTATCCGCAAGCAATCAATGCGGTTGGTAAGAAAGAAACCTTTGTTGGCCGAATTCGTAAGGATCTTGATGCGGAAAAAGGAATCCACATGTGGGTTGTTTCAGACAATCTTCTTAAGGGGGCGGCTTGGAATTCTGTTCAAATCGCAGAGACACTGCATGAGCGTGGTTTAGTTCGTCCAACAGCAGAAGTTGTTTTTGAATTAAAATAAAGTGACCAAGGAAACTTGACAGTACAGGGTTGAATAATCGTTCTTTCAACCCTCTTTTCATATAATAAAGAGAGGTAAGTTTATGGCGTATGCGGATTTGAAAAACTGTAAAATCATCACAGCTTTTATTACTCCTTTTCATGAAGATGGTTCCATCAATTTTGAAGCTATCCCAGACTTGATTGAGCACCTTTTGGCGCATCATACGGATGGGATTTTGTTAGCTGGAACAACTGCTGAAAGTCCAACCCTGACTCACGATGAAGAGTTGGAATTATTTGCGGCAGTTCAAAAAGTTGTCAAGGGGCGTGTCCCTTTGATTGCGGGTGTTGGTACCAATGAAACGCGCGACTCTATCGAATTCGTCAAAGAAGTAGATGAATTTGGTGGCTTTGCTGCTGGCCTTGCCATTGTTCCCTATTACAACAAACCTTCTCAGGAAGGGATGTATCAGCACTTTAAAGCTATTGCGGATGCTTCAAACCTGCCTATCATTATCTATAATATACCTGGACGGGTAGTTGTTGAGATGACACCTGAGACTATGCTAAGGTTGGCAGAACATCCAAATATTATCGGCGTTAAAGAATGTACCAGTCTGGCGAATATGGCCTATCTGATTGAGCATCGGCCAGAGGAGTTTCTGATTTACACTGGTGAGGATGGCGATGCCTTTCATGCTATGAATCTGGGGGCTGATGGAGTTATTTCTGTTGCTTCTCACACAAACGGTGATGAAATGTTTGAAATGCTGGATGCTATTGAGCACAATGACATCAAAAAAGCAGCAGCTATCCAACGGAAATTCATCCCTAAAGTCAATGCCCTTTTCTCTTACCCAAGCCCTGCCCCTGTCAAGGCAGTCCTTAATTACTTAGGATTTGCAGCTGGTCCAACCCGCTTACCGCTTGTGCCAGCGCCTGAAGAAGATGCCAAACGAATTATCAAGGTTGTGGTTGATGGTGACTATCAAGCGACCAAAGAGACGGTTAAAGGCGTTCTGAGACCGGATTATTGATTGATAGCAGCAGTTTTAGGATTGACATAGATAAAAATAATAAAGAAAGAATTGAAATTTGTGACAGAAAAACATAGTGAACGAATTGAGGAGTATAAGGCTAAACATAGACTGGAAAAACTTGAGGGCAAAAAAGTTTATGATAAGCCAGTTATCCGAACTGGTGATAAGGCCGGTTGCTATGGTTCAGGATTGATTTTACTTGGATCTGTTTTAGGAATTATTTATTTACTTCTTATACTGATAACGGATTGGGATTTGAAACTTTTCTCTTTCATCTGGGAAACACTTGCTGCGTTAGGGATAGTTTTTAGTCTTTTTGGAGCTTTTAAAATTGGTCAGTTGGAGCTGCACAGTTTGTCGATGGTGGCTGACAAAGAGAAAAATTTTGAAATCGATAAAGCTTTCAATCTTTATAACGTCATTCACAAACCAACAAAGAAAATTTTACTTCAATATGATAGTGAGGATGATAGTTTAATTTCTCTGAATGCTGGAGGAGAGTTATTAGTTGAGCTCTTAAAGAGAGTTGAAGAAGAAAAATTGTCCTTTAAGCCTATTAACAAAACATTGAGTTTAGAAGAATTGTCTGAAAATCACTGGAAAATGCACTATCGTTATAAAAGACAGGACATAACATTAGAGAAATCCGATACATAATGTTTGGCTTGTTATTTTAGTTGTATTGACTTAAACTGAAAAGCGAATTCAGTATCTGATAGTTTATGAAGAACATTGGAAAAAGGATTGTCCAGAGCAGTCCTTTTTTATATGGACTTTTTAAGCAAAAGAAAAAAGGAAGCTTCCTTTGAAAAATATTGGAAAAAATTATATAATGTTAAAGTATGGTCTGACTTTGAGAATTCTTTTTGAGCAGACTGTAGACAACTGGATTGGCAGCAGTCATGATAGAGACATACATTAGATTAGAAATATATAATAAAAAAGGAGTTTTTTATGCAAGTTATTAAACGGAGCGGTGAAGTCGTAGAATTCGACCCAGATAAAATTTATCAAGCAGTTTTAAAGGCCGCGCAGACTGTCTATGTGTTGACAGATGATTTGCGTCAGAATTTGGCGCAGGTTACAAAGAAAGTCGTTATGGATTTGGAAGAAGCTAAGGTTGAACGTGCGACGATTAGCATGATCCAGTCCATGGTTGAAAGCCGCCTCTTGGGTGCAGGTTATATTACTATTGCCGAGCATTATATTTCCTATCGCTTACAGCGCGATTTAGAGCGCAATGGCTATGGAGATCATATAGCTGTGCATCTGCATTTTGAACAAGTAAGATAAAGTTAGAAAGCCCGCTAGGGCTTTTTTTGCTCTTGAGGTAG

>c183_g154

ATGACCACTAAAAGAAGAAAAGCAACCGGCGTGCTAGCTACCGTTATCCAGGCCAATCTAGCATTTTGAACAAACATGATAATCACGAGGCCCAGATAGAGTAAGGCATTGCTCATAACCTGGATCATACTCTCGTTTAGAGCCTGCAGGATATTGTCCAAGTCGCTGGTAAAGCGCGACAGAATGTCTCCATCCTGATGGCGGTCAAAGAAAGCAACTGTCATCTTGGACAGCTTGCCAAACAGGCCCTTGCGCATCTCATTGGTCGAATGGGCAATAACTCGGGTCATCAAAACCATGTAAATCAAGCTGGAAACAGACAGGCAGACAAAGGACACCAATACACTGCCCATGACGCTGCCAAAAGTTGACAGAATCTGATCAGTCTCACCCTTCTCCGCAAAAGCCCGTCCCAGCTCAACCAAATGCTGAAC

>c183_g155

GTCGGATTAACCGAGCAGAGGTTCTGCAGAAAGAGCAGATTATGGGCAGTCAAAAATTAGCCCGGAAGATGCAGCAGGAATTGGGAGATATGAGGCAGGAGTGCTTGGTTGCTATTTATCTTAATAGTCAAAATCAAATTCTGCACCAGCAGACTATTTTTATGGGGACTGTCAGCAGAAGCATTGCTGAGCCGAGAGAAATTCTCCACTACGCTCTTAAGCATCTGGCGACATCTATCATACTGGTACACAATCATCCATCAGGCTCAGTCGTTCCTAGTAGAAACGACGATGAGGTGACCCAGCATATGAAAGAAGCATGTGAGATGATGGGTTTGGTCCTTTTGGATCATTTGATTGTGTCCAAGTCCAACTACTATAGCTATCGAGAGGAGACAGATATGATATAATTTGGTTTACTTGAATATAAAAAAACTTCTATTCGTGTAGAACAGAAGTTTATCTAGGAATAGAGGTTTTTAAAGAATATTGACAACATAATCAAACAGAGCTAAGTCTCCTTCTCTGGAGCCAAACAAGAACTCTGGGTGCCATTGGACGCCAAGGAAAGGACTGCCGTCAGTAGAAGTGATGGCTTCAATAATCTTATCGCGAGGATCATAGGCGATGACTTCTAGGCCAGCAGCTAAATCCTTGATACTTTGGTGGTGGAAGGAGTTGATTTCAGATGCTGGTCCATAGATTTCATGCAGAATAGAGCCGTTCTTGGTCACCATGCTTTGAGTTGTGTACTCAGCAGAGCTATCTTGCCAATGATCTTCAATATCTTGATGAAGAGTACCGCCTAGGGCTACATTATAAAGTTGGGTTCCGCGGCAGACAGTGAAGATTGGTTTATTTTGGCGGCGAGCTTCTTTTATTAAAGCTAATTCAAAAATATCTCGTTTGAGTAGATAATCATCGCTGTCAATGGTTTTCTCTTCACCATAGAATTGAGGGCATACATTCTGACCGCCTGTGATGATTAATTTATCAACAATGGAAACATACTGTTTAGCCATTTCTTCATCACCGATTGGCAAAATCATTGGAATTCCGCCAACTTCTTTGACACCCTCAACGAATCCTGTCGCTGTATAGCTCATATGGATGAAGTGATCGTCTGGGATTTCTCTTTCATTTCCGGTTATTCCAATAATAGGTTTACTCATAGAGTGTTTAATACCTCTTTCAAATTAATGTTCATTTTTTCTCATGAAGTAGAGCAAAGTCTGCAGTTCACTAGTCAGGTCAACATACTGGACAACCACGTCCTTGGGCAAGGATAGGTTCACTGGTGAGAAGCAAAGAATGCCCTTGACACCTGCTTGAACTAAGATAGATGCGACTTCTTGAGCTTTCACGCTAGGTACAGTTAGGATGGCAGTTTGCACATTTCCTGACTGTATTTTTTCTTTTATTTGGGAAATTCCATAGATAGGAATCCCATCGCTGGTTGTGTTGCCAACTTCTGGATGGTCATCTATGTCAAAAGCCATTACAACCTTCATTTTATTGCGCTCATGAAAACGATAGTGCAGCAGAGCCCGCCCCATATTACCAACACCAACAATCATGACATTGGTAATAGCATTGTCGTTCAAAAGATCAGCAAAAAAGTTCATCAATTTCTTGACATCATAACCAAATCCTCTGCGGCCAAGTTCACCGAAATAAGAAAAATCTCGTCTGACAGTAGCAGAGTCAATACCAATGGCTTCTGCAATTTGCTTGGAGTTTGCTTTTTCAATCTTCTCAGCATTAAAACGTTTAAAAATACGATAGTAGAGCGATAGCCTTTTAGCAGTAGCTCGAGGAATTGTAGTATTTTTTTCAGTTTTCACAAAATCACAACCTTTCTAATTCTATTTTATAGGAAGTTTGTGAAAAAATCAACTAATTAAGAGTGTTTGATTCACTAAAAAAGAAAAGAGTGGAAGTTTTGGTTATGTTGTAACTGCGAAGGGATTAGTCCTGGTAAGAAGTCAAGTCTAACTGGTACATCTGGCGGTACAAGTCACCGATTAACCCTGTAAAAGGCAGCTGGTGTCCATTATAAGTGATGGAAAGTACTTTGAGCGAGTCAGGAACTGTGACGCAGCCAGAGAAGGCGAGAAGAAATTCGTCGAATTCTTCATAGGTAAGTGTCCGTTTCACTTTATATGAGTCCAAATAGGTCAGTTCAACCATTGTTAATCCTTTCTTTGGGTTCGGTTGGTCTTTTCTTCAAGTGTTCTAAGTAGAAGTCAAGCTCGTTTAAATTTTGAAGTGTCAAAACCAAAAAAGCAAAATTGCGAGAATCTTGCTTATTGTGGTGTCTTTTCAAAAATATTTCTTTCAACCAGGCTATCCATCAAGAAATAAAATTTTTGCTGGATAATGTGATGGTCTTCTGATTTGAAAATATTGACCAAGCGCAGGCCTGATTTGTCAGTGATATTGATTTTGAAACCATTCAAATCTTTATTGACGATGATTTTCAGCAGAAAACCATTTCCGCTGTTGGGAACAGATTCCAGCAGACGAGAGAGTTCATAGTTTCCAACCTTGCTTTCAGCGAAAGTATTGTCGCGCAAGGTAAATTTCTTGACATTTGGGTGAAGTGAATAGGTATATTCACAGTTTGCTAAGCTTACAGATGTTTGAAAGGCCATCTTATCCTCCTAAAATTTCTTTTAATTTCTTTGTAAATGATTGAATCTCTTGCAGAGTCGTCTGGTCCGAAGTACTGATTCGAATAGACTCATGCAAGCGGTTGGAATCCTTGCCATACATAGCCTGCAAGACATGACTGGGTTGTATAGCTCCGGCTGTGCAGGCTGAGCCTGTTGAAATGGAAAAGCCATTTAAATCCATTTGAAGTAGAAGCAGGTCATTTTTTTGATTTGGAAATCCCAAATTGATGACATATGGCAAACTTGGCTTGCTTTCATTTAAGTAAAAGTCAATGTCTGATAAGTCAGCAAGTAAGCTGTCTTTTAAGTCTTGCGCATGCTCTAGATTTACCTCTAGATGTTCCGTGCTATCTGACAGAGCCGCTGCCATACCTGTGATAGAGATTAGATTTTCAGTTCCGGCCCGGTGTTTTTCTTCCTGGTCGCCACCATGCATGAAATTGTCAAAATCCATCTTCTTGGCATAAAGGAAGCCAACGCCTTTGGGACCATGAAACTTATGAGCTGAAGCAGATAAAAAGTCAATCCTTAGCTCGTCAGGGTAGATGGGAAGTTTACCAATTGCTTGGACAGCATCAACGTGAAAGGCAGCAGGATGTTCTTGAAGTAATTCACCAATTTCCTTAATGGGAAGGATAGCACCCGTCTCGTTATTAACTGCCATGACAGATACAAGAATAGTGTCAGGTCTAAGAGCCTTCTCAATATCCTCTGCTCGAATCTGGCCATCAACCGGCTGAACAAAAGTAGCTTCGAAGCCAAATTTATCTACTAGGTATTCAACCACTTCTAAGACAGCATGGTGCTCAATAGCAGTGGTAACAATATGTTTTCCTCGGTTCTGGTGACGAAAGGCATAGCCTTTGATAGTAGTATTATTGCTTTCAGTGCCGCCTGAAGTAAAAAGAATCTTATTGCTTTGAGTATGCAAGGCTTGAGCAATATCCTCACGCGCCTGTCTGAGCAATTTACTAGCTTCTCGGCCATGGCTATGCGTACTGGACGGATTTCCAAAGACAGTCATCGTCTTGGTCATTGCTTGGATTGCAGCGAGCGACAAAGCAGTTGTAGCGGCATTATCTAAATAAATCACTCTGGTTACCTTATTTCTTTTGATAGGCAAAGAGTGGGCTGACTGGTTTTCTCTCTTGAATGCGGACAATGGCATCACCAATCAGTTCACTAGCTGTAATGTAGTGGAGATTCTTAGGTGTTTTTTCTTTGGTATCAACAGAGTCTGTCACAAGAATTTCCCTGATAGGAGAATTATCTAGTAATTCTGCAGCTTTATCTGCAAAGAGTCCATGACTAGATACGGCATAAATTTCTACTGCGCCTTCGCGACAGACAATTTTAGATGCTTCAGAGAAAGTCCGACCGGTATTTAAAATGTCGTCAATCAGAATTGCTTTCTTGCCTTCGACATCTCCAATGATATAGCCCTCGGAGCGACCTGCCTCATCTTCGCCATAATCAATAATCGCAATCGGGGAATCCAAATACTCAGCAAGATTGCGGGCGCGTTTGACGCCAGAGTTTTTAGGACTGACGACAACGACATCTTCACCAGTTAATCCCTTATTGCAGTAGTGTTTGGCAAAGAGCGGAATAGTGAAAAGGTTGTCGACCGGGATATCAAAGAAACCTTGGACCTGCACTGCATGAAGATCCAGAGAAATCACGCGATCTACTCCAGCACCAACCAGCATATTGGCAACCAACTTAGCTGTGATTGGCTCGCGAGGAGCAGCAGTCCGATCCTGACGGGCGTAACCAAAATAAGGCATGACAACATTAATCGTATTGGCACTGGCACGCTTGCAGGCATCCACCATAATCAAGAGTTCCATCAAGTGATTATTGACAGGGAAGCTAGTGGACTGAATGATGTATATATCATAGCCACGGACACTTTCTTCGATATTAATCTGAATCTCGCCGTCCGAGAATTGGCGGGAGGAAAGTTTCCCCAATGGAACCCCAGCCGCCTCAGCAATTTTTTCAGCTATGGAATGATTGGAGTTGAGGGAAAAAAGCTTCATATTTTTTTTATCTGACATGAGTTGGACCGTCCTCTTTTTGTGTCATTTCTATAATCTAATGCTCCATGAAATTTATATTTCAATAGGCTTTAGATTGCTACCTTTTATTTTACCAAAAAAATTAGATTATTTCAGCTATTTTGTGGTCGTTGATTTACAAGTTTTCAGAAAATCTTGCTATCTTGCTCTTGCCGGGTTTGTAGTCGATCTTGTGCTGTTTTAGGAAGTTGAGGAAATTTTCTTTTCCTTTTTCGAAATCTTCAACTTCGACTTCGAGTTCGAAGTCAGTTTTGCCCAAGTAGTGGCTCTCATCCAGAGCAAATAGACCGATTTCATTTTCTTTTTCATAGCGGATAGTCTCTAGAGAGCCTAGAATCTTCAAGTCTTGGATGGGAATTTCTTTCTGAAGCAGCGTTTCTAAAATCTCACCAGCGGGAAAGATATTGTTCTGCAAAATATTTTCAGTTTCTTCTGGTGTCAAGTTCTGATTGAGTTCCAAGGCTCCGACTTCTTGAGGAATCTTGAGCGTCAGCTCTGCTTCGCGATGGTTAAAAGTCCGTACTCGAAAAGCCATGTGAGCGTGTCGGATACTCTGCTGATCTGAATCGATGTATTGGTTGGTTTGGCTAATAGGCGAAATATCAACAAATAACTGAAGCAGACGGTCGTGCTCTTCTTTGGTCAGCATGGTTTTGAATTCAATTTCTA

>c183_g156

CGCCGACATACTGACTTTTTACATACTCGAAAGCTGTACCATCATCGAAGTAAGAAATCTGAGTCAGTCCTAATATAGCATGGCCTCGAGGAATATCGAGGTAATTGGCGATTTTTTCCTTAGCCAGCCGAGCATAAATAGTCTGCTGAGACTTGCCGATTTTATAGCCATGTTCCTGCAAGGTCTGGAAGAAATGACTAGTCACCTCTTCTTTTTTGAAATTCTTAATGAACTTCTCAGGAATTGAAGCCACCTCGTAGACCACTGGCAAATCGTCAGCGTAGCGAACCCGCTCCATCCGAATGACATTTTCCGTCTTGTGTATACCTAGTTTCTCCACTTCCTGCTCACTCGGCAGGGTACGTCGATAAGAAATGAGCTGACTGGATGGAGTTTTGCCTTGCGCCTTCATGATTTCCGTGAAGCTGGTGGTGCCACGCATTTTCTCTTGAACGCGTGTGCTGGCAACGAAGGTACCGCTGCCGACCCGACGCTCCAGCACCCCCTCATCAACTAAGAGCGTAATGGCCTGACGCAGGGTCATACGGCTGACCTTAAAGGTTTCGGCTAGATCTCGCTCGCTGGGCAGGCGCTCTCCAATCTCCCAAACGGCATCGTCAATGTCCTTCTTAATCTGATCATGAATTTTTATATATGCGGGTAACATGCTGGCTCTTCTTTCTTATTTGGTACCTTCCTTTATTTTAGTACCAAATACTTCAAATGTCAAACTAAATGATAAAGATCAGCTCTCAAATGCTTGCTTTTCTCACGATTCTAGCTATTTTGTCAAATAAAGACTCATATTCGTTTTTTCAGCACTCTTGCTCCTTATTACCTTGGAAAAATTCCTTTTTTGTGATAAAATGAGGAGAAAAGATTACTTCTTTTAAGAAAGGGAACAGATGACTACTAGAACTGAATTGCAAGATGTCGAAAAGATCATCGTGCTTGATTACGGCAGCCAGTACAACCAGCTGATTTCACGTCGCATTCGTGAAATCGGTGTTTTTTCTGAGCTCAAGAGCCACAAGATTACTGCAGACGAGGTCCGTGCTATCCAGCCTGTCGGGATTATCCTTTCCGGTGGTCCGAATTCTGTATATGAAGACGGCTCTTTTGATATTGATCCGGAAATTTTCGAGTTGGGAATCCCGATTTTGGGGATTTGCTATGGTATGCAGCTGTTAACTCACAAGCTGGGCGGCAAGGTTGTTCCTGCTGGTCATGCTGGAAATCGTGAATACGGTCAGTCAAATCTGTCACATACTGCTAATTCCAGCCTTTTCAACGGTACGCCTGAAAGCCAGTTAGTTCTTATGAGCCACGGAGATGCAGTAACAGAAATTCCCACTGACTTTGTCCGTACTGGTACTTCTGCTGACTGTCCTTTTGCAGCTATTGAAAATCCAACTAAGAAAATCTACGGCATTCAGTTCCACCCTGAGGTTCGTCATTCAGAATTTGGTTATGATATCCTGCGTAACTTTGCTCTGAATATTTGTGGAGCTAAAGGCGACTGGTCTATGGATAACTTCATTGACATGGAAATTCAAAAAATCCGCCAAACTGTTGGTGACAAGAAGGTGCTGTTGGGACTTTCTGGCGGTGTAGACTCTTCTGTTGTCGGTGTTCTCTTACAGAAAGCAATCGGCGATCAACTAATCTGTATCTTTGTGGATCATGGCCTTTTGCGTAAAGGCGAAGCAGATCAGGTAATGGATATGCTGGGTGGCAAATTTGGTTTGAATATCGTCAAGGCTGACGCAGCTAAACGTTTCCTTGATAAACTAGCTGGTGTCTCAGATCCAGAGCAAAAACGTAAAATCATCGGAAACGAGTTTGTCTATGTCTTTGACGACGAAGCCAGCAAATTGAAAGATGTGAAATTCTTGGCTCAAGGGACTCTCTATACAGACGTTATCGAGTCCGGAACTGACACAGCTCAGACTATTAAATCTCACCATAACGTTGGTGGTCTTCCGGAAGACATGCAGTTTGAGCTGATTGAGCCACTGAATACGCTCTACAAGGATGAAGTGCGTGCTTTGGGTACAGAGCTTGGTATGCCTGATGAAATTGTCTGGCGCCAGCCATTCCCAGGACCAGGACTTGCTATTCGCGTTATGGGAGAAATCACTGAGGAAAAATTGGAAACGGTTCGCGAATCTGATGCTATTCTCCGCGAAGAAATTGCAAAAGCTGGTCTTGACCGCGACATCTGGCAATACTTTACTGTTAATACTGGCGTCCGATCTGTTGGGGTCATGGGAGACGGCCGGACTTACGACTACACAATTGCGATTCGCGCTATCACTTCTATCGATGGTATGACAGCTGACTTTGCCAAGATTCCTTGGGATGTTCTGCAAAAAATCTCTGTACGTATCGTTAATGAAGTAGACCATGTCAACCGCATTGTCTATGATATCACCAGCAAACCACCTGCAACTGTAGAGTGGGAGTAAAAATATAAAAACATATTATTCTTACTTCAAAAAGGGCGAAAAGCCCTTTTTGATTGCATTGACTTCGCACACCATTTTTTATATAATGACTAGTAAAATATTGATGAAGGAGTTAGAGATGAAAAAATCGCTGGATGCTTTTCATTTGAAATTGATTGCTATTGCTGCTATGCTTATCAACCATTTGGGACATACGCTAGAGTTGGAAAACCAGAATATTTACTTGTATTTCTTGACTGAAACAATTGGACGACTGACTTTTCCAATCATGGCTTATTTACTGGTTGAGGGCTTTCAGTATACACGAAGCAGAGGGAAATACGCACTTCGCCTAACTCTTTTTTGGTTACTATCCATTCTACCATTCTATTATCTTTTTGAAAGCCACAAACCATTGACGGTTAATAATAACATTCTCTACACTTTGCTCTTAGGGCTTTTATTGCTAGTATTTTTGGAAAAGGTTCAACATTCTTTTCTGCGCTTGCTCTTGATTTTAACATTCTCTTTTCTGACTTGGCAATCGGACTGGGGCTTTTTGGGAATCTTGACGATCGTTGGTTTCTACGAAAAACGAGAGGAATCAGACGGCTTTGTCACACCAATCCTCACCCTTATGGTAGTGTCTATTCTGATAAATCTTTGGGCCTTTTATACAGTTCCCAATCCTGTCCTGCTCTGCGATGCGGCTGCTATGTTGGGTCTACTCCTGACTCTGCCGCTTCTCAAGGCCTATAACGGCCAGCGCGGTTATTCGCCTGCCTGGGTAAAGTGGGGTTTTTATGCTTTTTATCCGCTTCACCTTTGTCTACTGCTGCTCTTCCGTATTT

>c183_g157

GGAAAAGCCAGATTATAAAAAATATGCAGAGGAATATCTCAATCTACAATAAAAGAAAAGCCCTTAGAAACTTCTGTTCTTAGGACTTTGTCTTCAATCTGAAGCGCTGAATTTCAGCGTCTTTTTCTTTTATTAATGTGATGTCATTGTTGGCAAATCCAAACTAGATTGCTCCAAATCTACCGTCAGCTGATAGTCGATCTTATCACGGACAGTATGCTCAAAGTCTGTAGTGTAGAGAACGAGGCGCAGTTGGTCTCCTTTTTTCATCTTATAGATGGTTGGTTGCAGTTCAAAGGAAAATTCCAGCCATTGGTCAGGAGTGACTTCTTCGACGCTCAGTAGATTGGTTCGATTTTGCAAATTGAGAAAACCTTTGGTGATGACGCGATGGGGCGTTTCGGCAAAGGGCAGTTCAACCAGATTGTCCAGCATATAGTAGCGGCCGTTGTCCATGACTCTAGGTTCAATAGGCGTAGGAATTGGTGTGTGTCGTTTAGCTAGTCCGAAGTCCAGCAATTGAGCAGAAATCAATCCCTTATCGGTGCTGGATTTGAGTCGAAGATTGAGCTGGGTCGCTCCGTTGAGAAAGAGGTCCTTTTCCAAGGTCCAGTCCAGAGTAATCTGGTTGACCTTACCGTCAAAGAGCTCAGTCTTAAAGCTTTGGTAGTTTTTAGCAAAACGATTATAGTCTTCTTCTGAATATTGATTTTGGATAGATTGGCAGTCTTGGCCTAGTTGTAGATGAAGATTCTGCTCTTGTCCACCGAAGTCTTCCAAGGTCAGCCAGCTTTGAGCTTGGCTATTGTCCTGCCAGATGACTGCTGGCAGGACAAAGTCTGATTCACAACCCAGCAGTTTCTTGCTCAGCAGAGCATTGATAGACTCACGGAAGTCGATGGACTGCCAGTTGTTCATGTAAACATGGGCACCGTTATGGAAGAAAAGATGCTTTTTGATGTGGGGCGGTAAGGCCCGAAACATATTATAGACATGGAGAGGCTTAACGTTCCAGTCTTGAGAGCCATGGGTGAAGACGACTTCGGCCTTGACCTTATCTGCGTGGAGCAGGTAGTTGCGGTCATGCCAAAATTGATTGTAATCTCCGGTTTGCCGGTCTAGGTCTTTGCGCTGTTGCTCTAGGTTTTGCTGATAAGCGTCGTTGTTGCGCAGGTAGTCACCAGCCCTCAGGTTGCGGGAGTAGGTCAGTTCGGTCAGGGATTCAAAATCCTCTCCTGGATAGCCGCCAGGACTTGTGACAAGGCCGTTTTCGCGATAGTAGTTGTACCAAGAAGAAATCCCTGCTTCAGCGATGATGACCTCCAGTCCATCTACACCAGTCGTTGCCAGTCCGTTGGACATGGTGCCCAGATAGGAGATACCGGTCGTAGCCACTTTTCCGTTGGACCAAGTGGCCTTGATTTCCCGCTGGCGTGTGTGGTCGGTGAAGGCTCGGCAACGGCCATTGAGCCAATCAATAACGTTCTTATAGGCCTCGATCTGCTGATAGTCGCCACTGGTCATCAGCCCTTCAGAGTCTTTAGTTCCTACGCCAGACACATAGAGATTGGCAAAGCCGCGGGGCAGCAAGTAATCATTGAGCGTGTAGGTGCCGATATGACCTAATTTTTCCTCGGTTTCAGATACTTCTTGGGCAGGAACTGGTGAATCCAGCTGGAGCAATTTAAGTTCAGGATCTTGTACCGTGATTTGATGGGGTTCTTTTTTTGCTAAGTCCACATTCATATCATGGAGAGCCTTGTCGCTGGCGGGATCATTGGTTCCTTGATGATAAGGAGAGGCAGTCATGACAGCAGGAACTGGTCCTTGATAACGGGGCCGGATGATGCTGACTTTGATAAGGTCCGGACGACCGTCTTGATCAGTATCCACACGTGATTCCACGTAAACTACCTCTCGGATAGCATCATGGCTGGAAAAGGTTGCTAGGCTCTTGCCGTTGAAGCAGTGGTGGGTATTGTCCTCAGGAATCAAACCCTCGCTGACTAGCTTATCAATCAACAAATTACCATTTTTTGTCCGAGTGTTGAGCAGCTGGTAGAGATTTTCCAATAAGTCTCCAAATGTAATAGGAAAGTCTGTTTCTTGGCGAAAGGCCTCAACATCTGTAAAATCAATAAAGGGCGAGAATTCAAGCAGCTGAAAGGCCACAGTGTAAAAAACAGACGCCGTCAGTTCTCTGTCAGACTGGAAAAAGCTCAGCAGGTCTGTTTGGCTGTCAGCAGCCCAGGACTTCAAGGGATAATCTGTATTTTTATAAGTGAAAAAGCTGGTGCGTAGAAAATCTTCAAGATTTTCCTTATCAGAGCGATTGCTGTCATAAAAAAAGCCTAATTCAGCTAATTCTATCAACATATTTTCCCGTTTCGTTTTTGCATAACTATACTGATTAAAACGCATATTCTTCCCTCTTTTTCTTATTTTTATTAAAAATGACCTTTACATTATCCATTATACTTGATAAAATAGACTTTGTCTA

>c183_g16

CCAGATGCTGGCATTGCACCCTTTACTTTTATCCATCCTAACCCTTATAAAGACAAAAGTCAGGATATGAGTACCTGGTATATGGGCGAAATTGAAATATCGGAAGACTCTTCTTGGGATTGGAAGCGAGAGCAGGATGAGGCAAAGGAAGCCGTGTATAATTTTTCGAATGCTCTTGCTGACTCAGGAGAGAATATCGTGTATCGAGTTCAAAAAGAAAGGGCGACTCGTTTCTTTAATGAATGGCTGCAAGTACATCAGGAAAATTTCAAATCAGCGATTCAATCAGAACTGTATAGGGAACTTCCAGAACTCGAACAATCATTGGGAAAGATTCAAAGTATTCGTCTGAGTGAACATCAGTCCTATTTTCCATCTTCTTCTAGAGAACTTAGTTTCGATATATCCTTTGAAAAATATCCTGAAGAAGTTGCAACCATAAAAGGAGTGGTTCGTTCTCAGAGTGAACAATCTATCTTTCAAGATTCTTCAGCGTCCGCATCTATAAGTTTTGACAATGGACGTTTTGTCATTGATTCTGAGAATGATTCAAAGCTATATTCCATTTTTAGTAAGAGTAGACTAGGAAGCAGTGCAGGGGACATATCTTATTACCTCCCA

>c183_g17

CGAGAGTGGTTTTGAGCTTGCTTTTATTGATAGCAAGCATGGGACAGTGCCACAAGCCTACCAGTCTAAGGCGAGTGGCCTAATCATCAATATCGAAGTTGACAATGTGGATTGTTTTTATGAAGAATTGCGTCAGCAAGAAGAAATGGAGTTCCTTCTTCCCATTAAGAGTGAAGATTTTGGTCAGCGCCATTTCATTGTGGAAGCGCCGGGTTCTGTTTTGGTTGATGTAATTCAAGTCATTCCACCTAACGCTGAGTTTGCAGCAAATTATTTGGAGAGTGAAGATGAATAAAAAACAGCAGGCTTCCTTAGAGACAAGCAAGAAAATATTAGCTATCGCCCGGAAACATTTTTCTTTAAAGGGTTTTTCAGAAACTTCCTTGGAAGAAATTGTAGATGAGTTGGGAATGACAAGAGGTGCACTTTATCACCATTTCGGGAATAAGAAGACTTTATTTACCGCCGTACTAGCACAAATTCAATCCGAACTGGGATCTTATGTAGAAAAAAACGCCCTGGAAGCACAGGATTCTTGGGAGCAGTTGGTGGAAGGCTGTGTTGCTTTTGTTCGTTTTGCGACCTTGACGGAAAATAAACGTATTCTTCTGCTTGATGGTCCCAATGTCGTTGAGTGGAAAGAGTGGCGTCGTCAGGATGAGGCTAATTCTTTCTTTCATTTGAGAGAGCAATTAGACATTTTATCTAAAGAAGGAAGACTTATCTCTATTGATTTGGATATGGCGGCTCATATGATTTCAGGTGCCTTGAATGAGTTGTCTCTTTTTCTAGCTGAGAAAGAGGAAGAAGCAGAAGTCAGA

>c183_g18

CGAAGGCCAGCTTGGTAAATCTGAAGCCATTGACAATGATAATCAGGCCAAAAACAAGGGCTAAAAAACCAAAGAGTAAGGTTCTGCGGCCACTGATCTTATGATATTTTGATACTTGCATCTCCATCTTTACCTTCTCTTTCGTTCGAAAAAAGAGTTGAGGAAATCCCCCAACTCTGATCTTATGTGCTTATCCGTAGATTGTTTTAAACAAGAGGACTGCTGTGATACCTGCAAGAATCGGTGCCACAACTGGAACCCAAGAATACCACCATTTTGAATCACCCTTGTGTTTACCCAAAACAGATTCTGGCAGGATGAAGTGGAGGATACGAGGTCCGAGGTCACGAGCTGGGTTCAGCCCAGGTCCAGTAGGGCCTCCCAGAGAGGTTACCAAAGCCATTACCAAGAAACCAAGTGCCAAGTGAGCGACTGAAAGGCTACCAACTTGGAATGGTGCAACTTGATCTTTAGCCTGAGATATTGCAGCAGCAATCTGTTCTTTAGGAACTGAAGTCCCTTGTGCTGCCATCTGAGCAGCTTGCGAATTGATAGTTGCTTCAGCTTTGCTCAACAATTCTGCTCCAAAGAAGTTCTTGGTCAAGCCCATAGCAGCAAAGAAAAGAACAAAAGAACCAACAAATTCATTGATAAAACCATTGAACAAGGCTGCTTTACGTGATTCAGGCGTGCCATGGTCTAGACTAGAGATGGTAGAGAAAGTACCCAAAATATTATTAGGATTTTCTGTTCCCAAATAGTAAGGGCGATGAGTCGCAACGACCAAAGCCTGTCCAAAGATAGCTCCCAAAATCTGCGCTGCAATGTATGGCGCAACCTGTGCCCAAGGGAAATAACCGCTGACAGCAAGTCCCAAAGTAAAGGCAGGGTTGATATGGTTACCGGACACATTACCAAACATCAAAGCTGGAATCATAACCCCCATACCGTATCCGACAGCGATGACCAACCAACCGCTCTGATGTCCCTTAGTTCCTTTTAATTCAACGTTGGCAACAGCTCCGTTCCCCAAGACAATGAGGATAGCTGTTCCTAAAAATTCTGTG

>c183_g19

AGCTATGATGTTTTTTCTGAGCGATGCAAGGGCATGATTGGAGATGGTGTTCTTTTCAAGGTTCCTAAAGATGTCTATATTGAGCTTTGCAAAAAAGAAAGCAAGAAAACACTCTTTGGTTATTCTCTGGCTTTGACAGATAAGGAGAAAGAGGCGGTTGAGAAGCGCCTGGCTGAGATTGATCAGCTGCTGGTAGAATGGGAGCCGCCAGCAGAACTGAAAAATGGCCAGCCAACATACTCCTACAAGTTGAAGCATGAACTAGGAGCGCAGCTATATAAGTTTAAAACCAGTCGTTTCAAGACCTATTTTGTCCTGTCGACCAACTGTTGCCTGCTAGCAGATTCGATTATTGGGCAGGCTGGGACAGATATTCTGGATATTCGTGGTATTATCGCACCTGGTACCTACCAGTCCTATCTGCAGTACGAGTTTGAGTCCGCTAGGGGTTTGGTCATTGCTCAGGCCGTTTACCAATAAATCCAAGGCCTTCTTGTTTTAAAAATTGTTTATTTGCGCATAATTTCTTGTCAAAAAGATGAAAATCCATTACAATAAAATGGTTTGGAGGAATTATGAGCGCGAATCATATTATTAGAATTATCCCTGTATTAAAGATCAATAATCGTCATTTAAATCAAGAATTTTTTGTAAATCAGCTGGGCATGAAGGCCCTTTTAGAGGAAGCTGCTTTTCTGTCTTTAGGAGATCAGACTAAGACTGAGAAACTGCAGTTGGAAGAATCACCCAGTATGCGGTCGCGGCGAGTAAAAGGTCCTAAAAAATTAGCCAGAATCGTTGTCAAGGTTGCAGATGCTAAAGAAATCGAGTCTCTATTGGCCCAAAAGCCTGCTTGGACTAAGCTGTATCAAGGAGAGAAAGGCTATGCTTTTGAAGCTCTGTCACCAGAGGGTGACCTAGTTCTCCTGCATGCAGAAGAGAATAGAACAAATCTTCAAGAAGTGGCGGAGGCGCCTGAATTTGAAAAGCAAGAGGACTTCATCGGTCTTAGTCAATTTGAGATAGAGACGGTAGAAATTCGTGTACCTAATGCCAATGAAGCTCAAGAATTTTATAGCAAGATTGAAAATGCGCTGGATTTCCTGACCTTTACAGAAGCAGAGGGACAAGACTTGCAGGCAGACAATGCCCTAACTTGGGACTTGACTATGCTCAAAGCTCAGGTCAATCGTTTAGAAACCGCTGCGCTGCGTCCTATCTTTGAAGGACACGAGGTCTTTGTACCTAAGTCAGATAAGTTCCTGCTGAGTCAGGATTTTAGTAAGATTGAATTGTGGTTTGAAGCATGACTCTAGAAAACATCATCAGTAAAATCAAAGAACAGCTAAAAGACGGTATCTATCCCGGTGCTAGTCTGGCCTTGTATCAAGCTGGCCAGTGGCAGGAATTCTACTTTGGTCTAGCTGATCCGCAAGAAGGAAAGGCCACTCAAGCGGGTTTGGTCTATGACCTGGCTAGCGTCAGCAAGGTGGTCGGAGTTGGTACTCTGGCAGCCTTTTTGTATGAGCAGGGCAAGCTGGAACTGGATTTGCCCTTACAGCATTATTATCCGGCCTTTCATCAAGAAGATGTGACCTTGCGTCAGCTCTTGACCCACACATCAGGACTAGATCCCTTTATTCCTAATCGTGAACAGCTGACAGCTCCCAAGTTGAAAGAAGCACTCAATCATTTGACAGTGCTTGAAGATAAGACCTTTCGCTATACGGATGTGAATTTTCTGCTCTTGGGTTTTATGCTGGAAGAGATCTTCGGTCAGGCCTTGGATCAGATTTTTCAAAGTCAGATTTTCCAGCCTTGGGGTTTGACTGAGACATGTTTTGGGCCAGTTCCAGGAGCTGTGCCGACCGTTCGCGGTGTCAAGGATGGTCAGGTTCATGATCCCAAGGCGCGTGTTCTAGGCATCCATTCTGGTAGTGCTGGCTTATTTTCAACCCTCAGGGATTTGGAAATATTTCTAGAGCATTATCTGCAGGATGACTTTGCGGCCAATCTGACCCAGAATTTTTCCAAAGAGCCTGGCAAAAGACGCAGTCTAGCTTGGAATCTGGAGGGCAGCTGGCTGGATCACACTGGCTACACAGGAACCTTTATCATGTATAATCGTAAGGAGCAAAAGGCGGCTATTTTCCTATCCAACCGGACCTATGAAAAAGACGAGCGGGCTCAATGGATCTTAGACCGTAACCAGCT

>c183_g2

AAACAGATGAGTTAAACGAAAAAATCTTGGAAAGCGCAAGGAGTGAGTTCTTGGCTTATGGCTATCAGGATGCTTCACTTCGGAGAATTTGTCGCGCTGCTGGCTTGACGACTGGGGCTCTTTATAAGCGCTATGAGAGTAAGGACAGTCTCTTTGCTGCCCTGCTTGAGCCTACTCTGACAGCCTTAGACCAGTATGGACAAGAGCAGAAGCGACGTGACTATGCTTTCCTAGAAGAGGGACACCTGTCTGATATGTGGGCCCATCGCTTGGAGGATCTCCAGTCCCTGATGCGGATTCTCTATGAACATAAGGATATTATGCAGCTCTTGCTCTTTAAATCTCAGGGATCTTCGCAAGCGGACTTTAGGATGCGTCTGCCTCATTTGGCTGCAGACGAGACTTATCGCTATTTGGAGCTGGCATACAAAGAGGGTAAGATCAATCATTTGGTCAAACACGAGTTTCTGCGATCGTGCATGACAGCTTATTACACTGCTGTATTTGAGCCCTTGGCTCAAGACTGGCCCCAAGAGCAGGCGCTGGAATTTTGCCATTCCATCATGGACTTGTTTGACTGGGGAGGTTTGCTAGGTTTTTGATAGAAAGAAGGAAATTCTATGAAGAAAAAATCTGTCCTTGCTTGGATCTGGGACTTTGTTTCCCTTCATAAGATATATTTTGTGCTCAGTCTGATCTTTGCCTTTGCCTCTGTGATTTCAGGATTTCTGCCTTATTTCTTTATTGGTGGAATGATTAATCAGCTCTTGGCTGGCAATAAAAACTGGGATTTTTACCTGCAGCAGTCAGCGTGGGCAGGTCTGGCCTGGATTGGCTACTGGGGATTCCATGGTATTTCTACCATGCTGTCGCATACAGCTACTTTTAAGATTCTAGCGGAAATGCGGCACCGCCTAACAGATAAGCTGGCTAGACTGCCTTTAGGCACAGTGCTTAGTCAGTCATCAGGCAGTTATAAAAACATTATCGTTGAACGGGTAGATGCGACCGAAACGACCTTGGCTCACCTGATTCCAGAGTTTACCGCTGGGATTTTTGGTCCGATTATTGTTCTCATTGCCATGCTGGTTATCGATTGGCGGTTGACCTTACTGTCTCTCCTGACTATTCCTATTGTAGTATTGGCCTATGTTCGGATGGCTGTCAATAGCGAAGCGGACTATCAAAATACTCTGGTCAAGACCAAAAAACTCAATGATACAGCCGTGGAATACATCAATGGGATTGAGGTTATCAAGGTTTTTGGCAAGGAAAAATTTTCTTACGATAAGTTTGTGACGGCTGCTAGAGAAGGAGCAGACTGCTTTATTGAGTGGATGCGCAAGTTCAATCTAGAGATGGGCATTGTGACTGCTTTTCTGCCATCAGGCTTGCTCTTTCTGCTGCCGGCTGGCTGTTATTTTTATCTGCAAG

>c183_g20

GAAACACTCGCTTCATTGCGATCAAATCAGGCTTCCTTGCCATTTTCTATACTGTTATTATATTATAAACTATCTGATTGACTTTGTCAATGAAGCGCTTTCAGATTTATAAAAAAATAAAGGCAGGGAAAAATTATTTCTCTACCTTACTGATTAAAATGCATATACTCTAATCTTCTCAATCAATGTGTTAGAGGAAACCTAACGTATTCAAGACTAGACAGACAATCCCCTTCAATTCTACCATTTTTCAGGCACTTGATTTCTCCAGCCTGTGAAAACTTGTGCATAGCCCAATAGATACAAGGGCGTCAATGCGATGCTCAAGCCTACCAAGGCAATAATCATATAAAGTCCTGACAGCATAAACAAGCGAGTGATGACTGCTACTGCAGCTGACAAGCAGCACAGGACGATGGCGTGAATCTGCAAAATATTGATCTTTTGTTTCTGTTCAAAATATCTCAATTTTGAAACGATCACCATCTGATAGAGCAAATTAATCAACTGATAAGACAAGCTGAGTATGATAAAAATCATAAAAAATGCTAAGTGGTTCTCACCAAAAAAGATGGAGCCCCAAAAAAGCAATTCAAAATGCAGAAATAGGTAAGAGCTGGTCGGAATGAGCTGCAAGACTTTGGTATCAAAAAACTTTTGGAAGAGTAAAAAGGCCAGGAACAAAATAAGCGCGTGAATGATAGTAAAATTCATGACTATCATGCTCAGATGATGCATCTCACCGCGTAAGAAAATAGAAACGATAAATACCAGTGAGATAAAAAATAACTCAATTAAACCAGCCTTATAAAAATCTAAGTTTCTAATTGGATTACAATCCGTAACAAGAACATTCAAAAATTCATAATATCTCTTTTTCATTTTGTAAACTCCCATTGTTTAACTTTATAAAATTTCCCCTAAACTTTGCGAAATTTTCTATAAAAAAATTCCACATTTCATTA

>c183_g21

ACTCAGTTGATGAGGAAGGAAATTCTAAAGAAGATTCTTCTGTGCTCAAAATCACCTTGGGTAAGTATGAGATTGACCATGTGGGTTCTTCCTTGATTGCAGAAGAAAAAGGCTTGAAAGATGTCTTTGCTCAGTATCAAAAGACTGCAGATGTTGAGGAGAATAGCCATTCTGTCCCTGTTTTAAATCGCATGATTTCAGCCTTTAAAAACGACTTTACTGGAAAGAAAAAGGTTATTCTGATTCGCTCTCAAAACGGCACTCCGTTGGCTGCCTATGCTGGAGACCGTGTTTCTCTAGATAAATCCGATGCCCCTAAAACTTCTGAGTTGCTGATTGACGGCAAGCGTCTCGTGATTTACCGCTGTGACTACACTATTTATGATCGTGAATTGCTGGAGTGAGCTATGAATGTAGAGGAATTGGTAAAAGAATTAAAAGCTGTAGCTAACCCTGATGATGCAGTGGCCATGAAAGCCTATATGAAAAATAAATTTGAGTTTTTAGGAGTCAAGACTCCAGCCAGGCGGAAACTCGCAAAGGCTTTTTTCAAACAGCAGACTGACTCCGTCATTGACTGGAATTTTATAAATGAAGCTTGGAAAAATCCCTATCGAGAATTACAGTATGCCGCGCTAGATTATCTGGAAAGTCGCAAGAAGCTATTGACTCCATCTGACTTGCCACGTTTGAAAAAGCTGGCCCAAACAAAATCCTGGTGGGATACCATTGACTTTCTAGACCGCTTGGTCGGATCCATTATTGCCCGATTTCCAGAAACCAAAGCTACTATTTTATCCTGGAGTTGCGATGAGGATATTTGGTTACGGCGCTTGGCCATTGATCATCAGCTGCTCCGAAAAGAAGAAACAGACACAGAGCTTTTGGAGAAAATCTTGGCGAATAATCTAGGCCAAACAGAATTCTTTATTAACAAGGCCATCGGCTGGGCGCTGAGAGATTATTCCAAAACCAATCCAGATTGGGTCAAGGATTTTATAGAACGGTATCGGGTGGAAATGGCTGCGCTTAGTATTCGAGAGGGAAGTAAGTATTTATGAAACATATTTATTTAAAATATCTGGAAGAGAAGGATTTCTCGGTTTGGCTGGAAGGATTTTCCAATAGACTACCTTCCCAATCGCCATTTGATGATGGGCTTTTAGATATGACAATTTGTACAGAAAGTTGGTTTGCGGATTTGGTGGCTAAGCACCGTGATTTTCG

>c183_g22

TCATCCATGAATAAACGAAACAGATAATTGCCATAATTTATTAGGTATTCAGATAAAAGGGAGACTCTCCATAGGTGCTTAAGCATTTCCAAGTTTTGAAAAATATGCTAGACTAAAACCATGACAAGAATCGGATTTATGAGCGACCTCCATCTAGATTCCAACCAGTTTGGAGATTTTGAGCAGCAAACTCTTCGCCAGCTTTTAAAAGAGGAGCAAATTGACCACCTACACATTGCTGGAGATTTATCCAACGACCTGACCAAGATTAGTTTGCCCTTCATTGAAACTTTGAAGCAAGAGATTCCTCTCTCTTTTAATCTGGGAAATCACGATATGCTAGGACTTTCTGAGCAAGAAATTTCAAACTATGATTTTCAGGTTCAACAGTTCGGCCAGACCAAGCTCGTCAGCTTTTCTGGCTGGTATGACTACAGCTTTGTTCCAGAAAAAAGCAAGGAAGAGCATCTGAGAACCAAGACCAATTTCTGGTTTGACCGCAGATTGGAGCGTCAACTCGACGACCCTAGCATTACAGCTCAGATACTGCAAGAGTTAGAAAAACTGCTGGCGACTTTAGATGGTCCCATTATTGTTGCTCTGCATTTTGTCCCCCATCAAGACTTTCTATACGACCATCCCTACTTCCAGCGCTTCAATGCCTTCCTAGGAAGCCAAGCTTTTCATCAGCTTTTTGTTAAATACAGGGTAAAAGAGGTGGTTTTTGGTCATCTCCATCACCGCCACCAAAGCCGTGTCATCGAAGGGGTTCGCTATCATATGCGTCCTCTAGGCTACATTCGTGAATGGGAACTGACTCGGAACTTTTTTAATGATTTTCCTCAGTATAAGATTCCCCAGATGTACCGCCTGCACAAGCGTTATAATGCTGTTAAAGATTTAGCCGAATTTCGAGACTATAAGAAAAAACACCTGGCAGCTGAACTGCGAGATGCTTTAACAGTGATTGAAGTTCAGTAATGGAAATGTTGAATAGCAGTTCTCAGCTCATCTGAGAGCTGTTTT

>c183_g23

CGCCGCTTCCTTCAAAGAATAATGTCCATTTAAACGAAAATCACGAAAAACTTTGCCTAAATCTTCCATTTGTTTATCACTTAAATTCCTTTAATTGCTGTTTCGCTCCCCTTGTCGCTCTTTCAAAACTACCTTCACCGCCCCTTGTTCGGATTTTATTTTCAGACGAAGCTCTTTTCGTCAAAATCTTATCGGCTGAAAATCGGAAAAGGCAAATTCTGCTAAAAGAGCTCGTCAGAAAATAGAAAGTTCGGGATTTGCTCTCAGAGCAATCGTCAGAAAATCTGACAGAGTCATTTCGCTCCAAAATAGCTTTTCAGAAAATCTGACGGACTGACTTGT

>c183_g24

GATAGTTTGAAAAAAATGGGAGTTGATGATGATAGGATTAAAGCGATTAGCGCAAAACTAGAATACTATGTCAATCCTTTTGATATTGTTAGTATGCTCAATCGTGAAAATACAATCTATAATTTAGAGAAACCTGGAGAAAAGCCAACTCGTAAGGAATTGGGAACAGCTCATATAGTTGTGCCTCTTCATTATACTCGCTTTGACTTTATGTCTGATAGTGCTCATGATTTTGGTGTTTTTCAGGCAGACGGCAAGGGTGGATTTTTAGTCGCTTCAGAAGATTTTCATCCAGAGCTTCTGAGAGCTGGAGAGAAGCTTGCGCGATTAGAAGCGAAATTTTTAGATTTGCTTCGTATGCAAGGTTTGACTGATGATGAGGCGATAAAATTCATGAATGCTGTTGTTGACGTTTCTTCTTTTGGAACAGCAAATTTTGCGCGTGCAGGTATTTCTACATCTGCTTTCTATGAATTTAAGCGGGATTATCAAGCAATCATTGATGAAGCGCGTAGAGAGTCGATTAAGTGGGACAAGAAGATGATTCCAAGCTATCAGAAACAGCTTGGAAATGGCAATCTTACAGGTGAAGAGAGAATTTTAGTTCGTGCTCGTCTCCTTCAGACTGCAGCTCAGTTGGCTATCTTTGAAATAGAAGATAAAGTTAAGCATGTCAAAACCTTATTGTCGGATGCCAAGGAATCTGTGCAAAAGATAATTAATGACGCAAGAACAGAGGCATTCGGTCTTGCTACTTATTTGAGCGACTCAGAAGTGGAGAGTTTATTGATTGATTTCGATATAAAGCACTACTGGGACGATTCGGTTGAAACAAATACAAACACATCCGCAAAAGGATTCCTGACAGAGATTGAACAGTTGGGAACGACTTTGGTCAGAGCAAGTGGAGATTTTGCAGCAGTCGATACTCAGCAAGCTGAGGATTTCAATAATCTCTTAGCAGATGTGAAAGGATCATGGAGGGTTATTGAAAATGCTTATACCAAATGATGATGCGCTCGTAACTAGAGTGGAGTTAGAGAGACAGTTTAAAGAAAAGATGAAGGAGCAGGAGAGGCAGGCTATCAAGGCTTTGATAGTGGTCAAAGAGCTGCTTATCCTTGCAAAAGGATTAGATCTGGCAGCTCAGCTACAGGCGGCGGCTTTGGATATGAAAAATTATGCTTCTACTAACTATGTCAATGATATTAAGGGTGGATTTGAAGGGAAGGCAGCGCAAGCTGCTGAAACTCATCTAACTCAGACGATGCAGATGCCTTCTTTGGATAGCCCGATTAAGGGATAGGAGGTGAGGCAATGCTGGATAAAAAGAAGCTTCAGGAACTGGAAGATGAACATGCTCTAAAAATGCGTGAGTTTGAGCGAGTAGAGACAGACTTAGATACATACTACTATAAGTTTGACAGAGAGACGAACAAACTCTTAGAGGCAATTTCTTATGCATGTAGAGAGGTTCCTTTGACAGCGGCTCAACCTTATATTTTTCAAATAGAGGATAATCTGGATCAATACCATCAGCAATACAAAAAGCGCATAGACGATGTTCTAGAAGCTCGCTATCAGGAGAACAGACGCTTTCAGAATAAGCTGGA

>c183_g25

CCATTCCTCTCTATGACAGGAGACTGCCTGCTATCTGCTTAGCTGGGCTTCCTTACTATCTAGTTTGATAGTTTCCCTGCCGTTGAGCAGTACACCTTTATTTTCATCGCGGCCTTGCAGGTAGTAAAGGGCATCTTCCTGACCGTAAGTTTCAATTGCCTTAGTTTGCAAAAGACCGATTCCCTTGCTAGGATCTCTCAAAAGATTGAAGATAATACCCATTTGCAGTTTAGGGAAGTCCTTGAGCATAGCATAGTCGCCATTACTGTCACCGGCAATCAGGATTGGTTCTTGATTGTCATGATTGACAGCAATCAGCTTCTTGATGGTCTCTGTCTTGCCCTCACCTTGAGTCTGAGCATAGTTGGTATCATATTCTGGCTGAATTACTCCCTTGTCATCCTTTTTCAGGCGCATAGCAGTAACATTTTCTTTAGGAATATTGTAGCCATACTTAGAATTGCTAGCATAAGGGATAATCACATCGATATAAGACGCTGAGCAGATATAGACATCAATGCCGTTAGCCATCAGAGTCTTATAGAGATTTTGCATTTCCTTGACTGAGCGAACACCACGCTTGAAGGTCACAGTGATTTGGCCTGACTCACCTTTCAAGCCTTCTGGACTTTCCCAAGTTTCAGAAGTTAGCTTGTCTTGAAGAGCCTGGTCAATAGACTTTTCAGATAGGGCTTGAACTTCTTCAGAAGTCATACCTGCATAGAGGTAGGTCACCCATGGATAGCTGATGTCAGAGCTGAAGGTGTCACCAATCGCTTCGTAAAGATAGCGGAGTTTAGCAGCAAAGTCTTGGTATTCATCGGTCTTTTTGACTTCTTCCAGAGACTTGTCTCCTTGCATTCCCTTATAGCTATTGTAAATAGCCGTGTAGTCAGAAAGCAAGTCAGCTGCAATCTTGTCAATGTTCACTGGTTCACCATCTTTGTTATGGAAATCCTCAACGAAATCGTCGCTTGGTATATTGGTCCGCACTGCTTGGTCAAATTCTTCCGGCGTCATCTTAAAGGCCAGATTTTCAATTTGATAGGTAAAGGTTGCTTCGCCAATGTCGTTAATGACTGTCGTATTGTCCCAGTCAAAGACGGCATAAGGCTTTTTATTTTTATCGTAGCTAGAGCTGGTGTTCCCATTTTCCTTGATGAGCTTGGTCAGACGGGCATAAAGCTTGTCTTCCCAGACGCCCTTATCCAAAGTCTTAGCCTTGTCCTTATCTGTTGTCTCCGTCTTAGCCGAGGATGACTCCGTCGTCGTCTTATTATTCCCCGCTTGACAAGCTCCCAGAAGCAAAGCCAAACTACAAAGCAGCACAATTCCTTTTGTCCGCATAGAAACAATCCTCCATAAAAAATAATAGTGCGCAAAAAAATGGCATAGAAAACCCCTTTGATT

>c183_g26

CTAGTTGTATGTCTGTTGCAATTTAGATAGAAACTGGAATTAAAGTAGCCTAATCTAAAAAATTTTTCAATAAACTAGCTACTCAATTTGTAAGATGAGGTGACCAACGCTTTTTAAACTTTAGCTCTGCACCTCTTCTTTGAGACCATTGAGCTTCAGACTCAGAATGAACATCTCAGGTGTAATATCCTTAATGTCCTTCAGTCCAAAACCTTGGAAGAAACTGTTCTTTTCCACCTCTTCGACATTGACCTTTTGCAAATCAATTTCAATCAAGGTCTGCAATTTCTTGTCTGCTGTAACTGAATAATCAATATTAACTCCTTCCAGACTCTTAGCTGCTACATAGTTTGAATCAGACTGCATTCCTTCACGAATGATAGTCGTCATTTCCTCTGGAGTCAAAGTAGCTGATGCCGCACTAGCTTCTTCTGGCAAGGAAGTGAGCAATTCCATCCGAAGATTCAAGATTTTCTTGCCCTGATAGGTAATTGTATCACGATGATGGATTTCTTCAATGTCTCCGACTAAAGTTTTAGAGACAATCTCTGTACTTTCAGAAGAAGAACTGGATGACTTGCTAGAGCTGGAGCTAGAGCTGGATTTAGTCCGCCTAGAAGAAGCTGCTGATTCAGAAGAATGTCTAGGACGATGCTTGCTGAAGTTAGGAAACACACAGCCCGTCAAGAGTACCGAAGCAGAAATAACAAGAATGAAAAATTTTTTCATAGTTCACCTTTTTTTACTTTTTATTTGTTACAGTATAACATAATACCAGTTTCTTGTCTAAGTATTTTCTACAAAATGACAAAAGGCAACCTTTCGATTGCCTTTTTATACTATTCAACACTAAAAATATATGGATAAACCGGCTGACTGCCCTGATGAATTTCTACTTCGACATCCTCGAATTGTTCCATCAAATCTTGAGCAAGACTGCTAGCCAATTCTTCGCTGCCGTCTTCACCGATATAGATAGAGACAATCTCGCTGTCTTCATTGAGCATCTTGCTGAACGTTTCTTTCAAAGTTGCCAGCATATCTGGATTAGAAACAACAATCTTACCATCCACCATACCGAGGTTGTCATTTTCATGAATTTCCAAGCCATCAATCGTCGTATCACGAACCGCAGTCGTCACGCTGCCGCTGACTACTTCTGCCAAGGCTGCAGTCATACGCTCTTGGTTTTCCTCAATAGTCTTGCCGCCATCAAAAGCCAGGAGACTGGTCAAGCCTTGAGGAATCGTACGAGTCTCAATAACAGCTGCAGGCTGTTCAATCACTTCGGCTGCTGATTGGGCTGCCATGAAGATGTTTTTATTGTTTGGCAAGATAATAATATTGCGAGCATTGACTTGCTCCACAGCCTTGATAAAATCTTCTGTTGATGGGTTCATAGTTTGCCCGCCAGAGATGATGTAGTCAACTCCCTGAGCCTTAAAGATTTCAGCCAGACCATCACCAGCCACTACAGCGATAATCGCAAACTCTTTTTCTTGAGCCGGCTTAGCTGAGCGCTCTTCCTTTTCAACCTGCGCTTCGTGCTGGTTGCGCATATTGTCAACCTTGACCTTGACCAAACTACCATACTTGAGACCTTCCTGCATAACCAGTCCTGGATCTTCAGTATGGACATGGACTTTGACGATTTCGTCATCGTTGACTACCAACAGGGAATCTCCCAAATCGTTGAGGTAGTTGCGGAACTCATCATAATCAAAATCTTTTACATAGGTTGGTCCTGTTTTAAGAGCCACCATGATTTCTGTACAGTAGCCGAAAGTGATGTCTTCTGTCGCCACATGGCCAGCTACAGACTTATGATGCTCAGCATTGATCATCTCAGACATGGTCGCAGGTGTCGCCACAAAGTCCTCAGATGCGATATATTCGCCTGTAAGTGCAGACAAGAATCCTTCGTAGATAAAGACAAGCCCTTGACCGCCAGAGTCCACAACACCGACTTCTTTCAGAACCGGCAGCATATCCGGTGTCTTAGCCAAAGCAGCCTTTGCTCCTTCAAGAGCTGCTTTCATAACTTCTACAGCATCATTAGTGACTTCAGCTTTCTTCTTGGCTCCGATAGCCGCACCGCGGGAAACAGTCAAAATCGTTCCCTCAACCGGCTTCATAACTGCCTTGTAAGCCACTTCTACACCAGACTGAAAGGCCAGAGCTAGATCTTCACCAGTCAGCTCTTCATGCTCTTTCACACTTTGAGAAAAACCGCGGAAAAGCTGGGAAGTGATAACTCCTGAGTTACCACGCGCGCCCATCAAAAGGCCTTTCGCAAAGATACCCGCCGCTTCGCCAACTGTTGAAGCAGACTTGTCTGCCACTTCCTTGGCACCATTTTCAATGGTCATACCCATGTTGGTACCTGTATCGCCATCTGGAACCGGGAAGACATTCAAAGAGTTCACATATTCAGCCTGTTTATTTAAGCGAGTAGATGCCGCCTGCACCATTTCTTGAAATAAACTAGTAGTAATATTTGCCACGATTATTCTCCTACGACTTTAATATTTTGGATATAAACATTCACTGCATGGGCAGTAATTCCGAGCTTGTTTTCCAAACTGAATTTAACACGCTCTTGGATGTTTTTGGAAACTTCGCTGATTTTGACTCCATAGCTTAACACAGTGTAGACATCAACTGCGATATCACCTTCATCAGTCGCTTTCACAACCACGCCCTTAGCATAGTTTTCTTTTCCTAAAAGGGCTTGAAAATTATCCTTGATTGCATTTTTGCTGGCCATGCCAACTACACCAAAAATTTCAGTCGCTGCACCGCCGACGATTGTCGCAATCACATCATCAGTCAGCTCAATTTGACCATCTTTTGTATTAATTTTCACAGTCATAATTTGTACCTCAAAAGTATTTTATAGTTTATTCTACCATATTTTATACAGGGTGTAAAAGAGGAAGGGTCTAAATCAACTGTCTAGGCGGAAATTTCCAAGCAAAAAAGGACGGAAAATTTCCATCCTTTTTAAATAAAGATTAAACGCGTTCAACTTTACCTGATTTAAGGGCACGAGCTGAAGCCCAAACTTTTTTAGGTTTACCGTCAATCAAAACAGTAACTTTTTGAAGATTTGGTTTAACAGCGCGTTTTGTTTGGTTCATCGCGTGAGAGCGGTTGTTTCCTGATACAGTCTTACGACCAGTAAAGTAACATACTTTAGCCATTATCGTATTTCCTCCTATTAGATCTAATATTACGGATGTGCTAGCACCACATACTTTCCTATATTACCAAAAAACTTGACGTTTGACAAGTACATTTGTCCAAACTTTCGAAATTAATCCGTTTTCTTAAACATAGCTAATCTTCAAAGAACCGAAAGCTACGTTGCCTTTGACATAGAGAGTTTTTCTCTTTTCATTGACATTACGAGGATTGTAGACAGAGCCAAAGGCATTATCTACATCCAGCTCTACACGCCAGTCACTGGGCACATAAAGCATCACACTGCCAAAAGACAGATCCACTTCAAAAGTTGCTGAGTCTCCTTCGATAGTCGCATTATCAAAATAAACCGATGCACTGCCAAAAGCACAGTCTAACTTTTCATAGGTGAAGTTATCTGAGTTGATATAGCGCGTGCCACTGCCAAAAGCAATATCATCTTTTGAGGAAGAAGAGGCAGCCGAGAAGATACGCCCCTTAAAAATTCGCTGGGGCTTGAAAATCATATTGAGACCAATACAAGCCAGAATACCACCCAAAACCAAAGTTCCTGTTGAAATAGCTAAAAAGTGGTAAACGGCATTGGCAATAATCAGCGCAATAACTGCCATGATCAGCCCAGCGCCAAAGTCTCTTTCCAAGAAATTTTCCAAGGCAAAATAGGCGAACATTGCAACAACTAGCATGGGCCAAATATTAAAATTGAAGGCAGGAATTCCAAAATTCCCCTGCAACAGGACCCAAGCTGCCAAGACTAGAAAGCCGATTCCAAAAATTGTTTTTTTCATGATAATTACCTCACTTCACTTAACTTTTCTTTTACTAACTGATAATAATGCCTAGACACATGCACCTGTTTATGGGTATTGTAAAACTGGACGGTACTTGTTCCTGAAAAGGACTTGTCCAAAGCATAGATAGCCTTGATATTGGCAATAGTGGACTTTGAAATCCGACAGAAATAACGGGGCAGAATCTCTTCTAACTCATAGAGCTTTAGCTTGACCTCATAAGCATCATTGCGGGCATGGGCAAAAATCTTGCTGCCGTCCGTCTCGAAAAAGAGAATATCCGCTAAATCAAGAAAGTACTCACTGCTTTCCTTATAGAAAAGAATGGACGGAGTCGCCGTTTCTTCCAGTGCTTCTTGGATCCGCTGAATCCGCTCATCCAGCCGCGAAACTTTTATCACCATTTCTGTTTCAT

>c183_g27

GAAATTATTAACTTAGCAAAAGACATTGGGATTTCTAAGATTGGCTTTACGACAGCGGATGATTTTGACTATCTGGAGAAGTCGCTGCGCTTGGCTGTAGAAGAAGGACGAAATTCAGGATTTGAACATAAAAACATCGAGGAACGGATCAAACCCAAGCTGAGTCTGGCTTCGGCTAAGACCATCATCTCCATCGCAGTCGCCTACCCCCACAAGCTCAAGCAGCAACCTCAGAAAACGGCCTACAAGCGGGGCAAATTCACCCCTAACAGCTGGGGGCTGGACTACCACTATGTCCTGCAGGACAAGCTGGACCGGCTTGCCAAGGGAATCGAAGAGCTGACCGCTGACTTTGAATACAAGGGCATGGTGGACACAGGTGCCTTGGTTGATACCGCCGTAGCTCAAAGAGCAGGAATCGGCTTTATCGGCAAGAACGGCTTGGTCATCTCCAAGGAATTTGGTTCTTATATGTTTTTAGGAGAGCTCATTACCAATCTGGACATCGAGCCTGATCAGCCTGTAGACTACGGCTGCGGAGACTGCAACCGCTGCGTGACAGCCTGTCCTACTTCCTGCTTGATTGGTGACGGCAGCATGAATGCCAAGCGTTGTCTATCCTTTCAGACTCAGGACAAGGGCGTCATGGATCTGGAATTTCGCAAGAAGATTAAAACCGTCATCTATGGCTGTGATATCTGCCAAATCTGCTGCCCTTACAATAAAGGTTTGGACAATCCTCTGGCGACAGAGATTGACCCCGACCTTTCTCATCCAGAACTCCTGCCTTTCTTGGAGCTTTCCAACGGTCAGTTTAAGGAGAAATTCGGTCATGTGGCCGGCAGCTGGCGGGGGAAAAATATCCTGCAGCGCAATGCCATTATCGCTCTGGCAAATGCCAATGACCGCTCTGCCATTCCTAAAATGCTGGAAATTATCGACAAGGGGCAAAATCCCATTCATGTCGCCACAGCTATCTGGGCTCTGAGTCAGCTGGTGCGTGAGGTCCATCCGGAAATGATAGAACTGGTCATGAACGTCAAAAATCCAACTCCGCAAATCCAAGAAGAGCAAGGCCGTTTCCTAGAGAAATTTGG

>c183_g28

AAAAATGTTACATACTTGTTTACGTGTTGAGAATTTGGAAGCGTCTATCGCTTTTTATGCAGAGGCTTTTGGCTTTAAGGAGCTGCGCCGCAAGGATTTTCCAGATTATCAGTTTACTATCGTTTATCTAGGTTTGGAAGGTGATGATTATGAGCTGGAACTGACCTATAACTATGATCATGGTCCTTATGTGATTGGGGACGGCTTTGCTCATGTGGCACTTAGCACGCCAGACTTGGAAGGTCTGCACGCTGAGCATAAGGCCAAAGGCTATGAAGTGACAGATCCAAAGGGGCTTCCTGGCAACCCGCCTAATTATTATTTTGTAAAAGATCCTGATGGCTACAAGGTGGAAGTCATTCGTGAGAAGAGTCTCTAACATCTAGCTGCAGTTAATTTTGCAAGATAAATGATGATTTTTATTCATTTAGGACAGGTGTATGAAAAGACAAAATAACAAGAAACACGGTAAGAAAACACTCATCGTTCTCAGCTTGCTAGGCTTAGCTTTTGTTGCTGCTCTATTTCTGGGTGCTGTAAAGATTTACGCTATTTTCCAAGAAAAGGAACTGCAAGAGAAGGTTAGTGTGCTGATTTCTCAAGAAGAGACTTTCCACGCTGAAAAAACTGAAAAGCAGAGCAAGATGATTGGCAGCCACTATGTAGAGGCCTTTTATCCTCTTCTGGATGGTCAGGTTATGGCAAGTGTCAAGGAGCAGATGGACGCTGATAGCCAGACGATTAAGGATAATCAGAAGAAGGGCGATAAGATTGAGGAATTGACTTTCTACTATGCTGAGGAAAAAGAAACGAGCCTGAAGGATGTCAAAGAAGTACTGGTGCATCGTAAGGACTATCATGTCAAAGAGATGAAAATCAGTAAAGGAGAAGAGCGGGAAGTTGCTGACAGCTACCTCGGTGCAGACGGCAGCCCTTTCACTCTGGATAAGCTCTTTCAGGATCCCGATGCAGCTAAGGAAATCTTCATCAACGAAATATCCAGCCAGCTGACCTTTAGGCAGGCCGATGAGGCGGTGCAGACGGAAATCCTCAACACCCTCAATGGAACGGAGTTGGGTCAGTGGTCCTTCCGTTATGAGTACAGCCATTTTTCTATTAAGCTGAGCAAGGAAGTGCAAGGCTTAACCAGCATTGACGTTCCGTTATCCAGCTTTTATGACCAGATCAATGCGGACTATCTGACCGGAGATGACTTGGCGGCTTATCAGAGTTTCGAGGCTAAGAAGCATGTGAAGATGGTTGCTCTGACCTTTGATGACGGTCCAGATCCTAAGACAACACCGCAGGCTCTGGATATCCTCAAGAAATACGGTGCCAAAGCGACCTTCTTTATGGTTGGGCAAAACATTGCTGGTAATGAAGCGATCGTCAAGCGCGTACACAATGAAGGTCATCAGATTGGGATTCATACATGGGACCATCCAGTCTTAACCAAACTTCCTCTGGAGACAGCCCAAAAAGAAATCCTTGATACACAGACCGCTATTAACAATGTCATAGGCATCAAGCCAACGATTACGCGACCGCCTTATGGAGCCATTAATGCTACAATCCAAAATTCGGTTGACCAGTCCTTTATTATGTGGAATGTGGACAGTCTAGACTGGAAGACACGCAATACAAAAGCTATTATGCAAGAGATTGCTAAAACTCAGCCAGGCTCGATTATCCTCATGCATGATATTCACCAGACCAGTATTGACGCTCTGCCAAGTGTCCTCGAGTATCTGAAGAGTAACGGCTATACTTTGGTAACGGTCGATGAGCTGCTGGAAGGCCAGATAGAGCCGCATCGTATTTATTACGGCAGAGACTAAGACGGTCCTTGGGGTTAGAAGTTTGAAATCGTTGTTGGCAATGAGTAAAAGCGAAAAGCTTTGGAAATCTAATGTTTCCAAAGCTTTTTTAGTTTAACTCCTTACATAAAGTTCTTGATTTTTTAGTACAATTTCGCGAACAGAAGCGTATTTTTTTAATGTCTTCAGTTCTTCTGGATGACTGATAAAGGTAATGACATAGCCTTCCTTGCCCATGCGGCCGGTTCGGCCAGAGCGGTGGGTATAGGTTTCTAAATCGCGCGGAAGCTCATAATTGACGACGCATTCTAGCGAGTCAATATCAATGCCGCGAGCAACCAAGTCAGTGGCTAGCAAGAGAGTAATCTGATGTTCTTTGAACTTATCTAGAATCACCTTGCGGAACTTAACATTGACATCGCTGGCTAGAGAAACCGCGTTGGCTTCACGATACTGGAGTTTTTCCTCGGCGCTTCCTAGGTCTGACAGAGCGTTGAAGAAGACCAGTCCACGGAAATCTTCAACATTGGATAATTTGCGGAGCAACTCCACCTTATCACGTTTTTCGACTTGCATGTAAAAATGCTGGATGTTATCCAAAACTTGGTCGTCAATGCTGATTTCTAGAGTATTTTCAGCAATTTTATCGTGGTCGAACTTAGCAGTGGCGCTCATATATACCAGCTGGTGGTCACGAGGCGCATAGTGGGTGATTTTATCTACAAAATGATACTGAGAATCGCTGAGCAGCTGGTCAAATTCATCTAAAATGATGGTTTCAACATTCATCATCTTGATTTTCTTGAGCTTGACCAACTCAAAAATCCGTCCAGGCGTTCCGATGATAATCTCTGGACCTTTCTTAAGGCGCTCAATCTGTCGCTTCTGGCTGGAGCCAGACAGCAGGAGCAGGGCTGTCAGCCCTAAAGGCTCCGCCCAAGTCTTGCAGACCTCAAAGATTTGTCCAGCCAGCTCAGTATTAGGAGCTAAAATCAAGAGTTGTTGGGCTTTTTTAGGTGTTAGTCTAAGCAGACTTGGAAAGAGATAGGCCAGAGTTTTACCAGTACCAGTCGGACTGATTCCCAGCACGGTATCGCCTGCAGAAATAGGCTCAAACATCTTCTCTTGAATAGCTGTCAAATTTTCAAAGCCTAGCTGAGCCAGCTGATTTTGCCAGCTCGGGGGAAATTGTTCCTTAATCATCATCAACCTCAAATCTAATGCCAGCATCCTTGCGCATGGCAAATAAGCTTTCGTGAACAGCTGCTGCCGCATCTAACCATTTCTCGTAAGTCGCATCCTGTCCACCCTTGAGCACTCGCGCGAAAGCCTGAGCTTCTTCCAGCATGGTGTGGGAAGCGCGCTGGATAGGCAGCACCTCCTCTTGACCGTCCAGACCTTGGAAAATAGCAGAGCTGATAAATTCAATCCCGTCTAAAGTCAGAGTTCCCTGATCTGTGTAAATCTCTGCAGGCAGATTGCTGTTTATATTTTTACCGGCCTGAATCTGAACTTGAAAATCTGGATAGATGAGACAGCCGGCTCCATTTAAATCAATCGTATTGGCTAGCTGCTGGGCGCTGTAGCGAGCGAAGCGCGGATGGCCGAAAAGTCTGATAGCCGCATAGACTGGATAGACACCCAGATCCATCAAAGCGCCGCCAGAAAATTTAGCTGAGAAAACGTTAGGCTGCTCTCCCGCAAGCAGAGCCTGCATTTTTGAAGAATACTTGGCATAGGTGAAGTTGGCTCCTAAGACAGTCTTGTCCTTGAGGAAATCACTAATCGTATCAAATGCCTGCTCATGATAGTTGCGGGCAGCTTCAAAGAGATAGACTTGATGCTCGTCAGCCAGCTTGACCAATTCCCGCCATTCTTTGGGACGAGAAACCGCAGGCTTTTCGACAATGACATGTTTACGAGCCAGAATGGCTACCTTGGCATGCTTGAAATGCAGGCTGTTGGGACTGGCGATATAAACGACATCAATATCGGACGACAGAAATTCCAACATTTCAGTATAGACGGCTGTATTTTCATAGTTTTGCACAAAGCGCTCAGCAGAAGCCAGAGTCCGAGAATAGACAGCTGCCAGCTGATAGTCGCTTGTTGAGTGAGCGGCCTTGATGAACTCATGAGAAATAGAACCTGTTCCGATAATTCCAAGTT

>c183_g29

TTTCCGCTTTCATTTATCCCTCTATTATAACATAAGAGCCTTTTTGCTGAGAATGAAAAAAAGTTCCCGAAGGAACTTCTTTTAAAAGCGAATATTCTCGCGTGTTTTTTCGTAGGATGTCACAAAACGCTCTGTCACGCCTGGCTCGACAAGATCCAAAGCTTTTGAAATGATTTCCAAGGATTGAGCGTAGTCGTAGTCATGTTCAAAGACATACAAGGACTTGTTGAAAGCCGCCTGTACGTTATCATCAAAGGAACGATAGCGATTTGAATACTGCAGCAGCTGCTCAGTCAAGGTAGCATCTTGGACGATACGATAGGTTTCTTCTTCCAATTGCTCCATATCATTTCCTAGAATTTCCAGCCAACGGTTAACTGATTCGATATTGACTCGTGTAGCTTCCAATTCCTTCACCAACTCTTCAATATTATTGCTGGTTGAAAAGAAAATTTCTAAGAAAGAGTCTGGAATACCTGGCAAATTGCGTTTTTCCATATATCTCTTAATGGTATGCAATTTGTTGGCATAGATATTGACTTTTTGGCGAGCATTAGCATCATCTTTTTCAATCTCTGCCAGTGCTTCACCAAGAGAAATCTGCTCATCTTCGATTTCTTTCAAACGCTCTTGGATAGCTTCCAACTCTTCCTGAACCACTGAATAAGCTTGCTTGGTTTCAGAAGAATCTTCTACCGCGCTAAGAACCACATCTTCCTGGGCAGAAAGTTCAGCCTGCAACTCCTTGACATGAGAAGTTTCCGTATCAGAGATAAGGAAGGTTTGAGATAAACGTTCTATCTCTTTTTGGAGTTGCTGGTTGTTTTCCTTTGTATGAGCCAGATAGCTAGGTAAGTTTTTAATCAACTTCTCCACTACTTTGTGGGCTTCAATTTCCCGCGTAAAAATTTCATAGAGAGCATTAATCTCTTCTTGCGCCTGCTCATTTTCATACTCAGCATTATCCAGCTCTAGGGCTGAAATATTGGCTTCGTTGCGTTTGAGGCTTGCATGGAGCTGCTGGAAGCGAGACTCTATATCTGTTTCGATAAAGTGATAACCAGATTCCAAGAGCTTACGGTGACCAGATTCTAAGTCTTCCAACTGATCTGGCAATTTCACAGTCAACTCTTCGACAATGGCAGGAACTTTCTCAACGATATGTGTCAGAGCAAGGATATGATCCTCAGCTTTATCCAATATTTCAGCTGCTTCAACCGGGTCACCTGAAGAATTAAGAGTCACAAACTGAGAAAACTCTGACTGGATATTTTCCAGCTGTTTTTCAATCTCAGCTAAGGCTTGTCCATACGAGTCTGCATTCTCCGCAACCTGGGTTTGCAGTTTTTCAAACAAATCCAAGGCATGAAGCACACGGCCGCTGTTCTTAGACTCTTGCTCTTTAAGATCTTCCAAGGCTGCACGAATCATCTTGATATCTTCTTCAATCAGATCAATCTGACTCTCAATATTGCCGATAGCATGCTTGGCTTTTATGAAACGGAAAGAATTATTGTAACCTTCTGCTTCAAATAGATTATTTTCGATATCAGCAAATGAATTTAAGGACAAGTCTACCCATTTTTGATTCCATTCACGGAAGGCTACCTGACTCTGCCCAATCAAATGCATATTTTTTACTTCTTCAACCTCATCATTCACAGGGAGATTATAGAGTGCTTCTTTTCTCTCCTCCAAATTTTGAAGTAAAGCTTCATTTCTCTTTCTCATCAGAACTGCAGTACCATAGCCGACAACCAAAAGCAGAGCTACCACAGCAACGAGAATAACTAGTCCAATAGACATATAGAACTCCTTCACATTGTTACTATAAAGCAAACATAATGATTATATCATATTTTTGCCCATAATGGTTGATTTTTCTGATTTTTTTAGACGTCGAGCGTACTGTATACAGCATTTTCTTCGATAAATTCACGACGAGGTTCAACGCGATCCCCCATCAGCATGTCAAAAATCTTGTCAGCTTCCGCTGCATCATCCACTGAAACCCGTGCCATCAAGCGATGTTCAGGATTCATAGTTGTCTCCCACAGCTGATGGTCATCCATCTCTCCGAGACCTTTATAGCGTTGAATGGTTGGCTTGGAGCGACCTTCACTGTAACGAGCCAAAGCAGCCTGAAGTTCTTCTTCCTGATTAGCACCTGGCTGGATGTATTCTTTGACTTCACTTCCGACCTTGACACCATAGATTGGTGGCTGAGCGATATAGACAAATCCAGCTTCCAGAACTGGCTTCATATAGCGGTAAATCAAAGTCAAGAGCAGGGTCCGGATGTGAGCACCGTCTACATCTGCATCGGTCATGATGACTAATTTTTGATAGCGGGCCTTGCTGACATCAAAGTCAGCACCGAAGCCAGTTCCCATAGCAGTAAAGAGACTGCGGATTTCTTCATTGGCCAGAATCTTGTCCATGCTGGCTTTTTCAACGTTGAGAATCTTACCACGAATTGGCAGAATGGCCTGAAATTCACGGTTACGACCGGACTTAGCTGAACCACCTGCAGAATCCCCCTCCACGATGAAAAGTTCTGTTTCCTGTGGGTCGTTGGACGAGCAGTCAGCCAATTTACCGGGCAGATTGGAAATTTCCAAGCCAGACTTCTTGCGGGTTACTTCCCGAGCTCGCTTGGCAGCAATTCTAGCCTTAGAAGCCAAAATTCCTTTTTCAACGATTTTTCGAGCTACCGCAGGATTTTCCAAAAGAAAATCTGAGAAGGCTTCGCTAAAGAGACGATTAGTAATCTTGACCACTTCGCTATTGCCTAGCTTGGTCTTAGTCTGACCTTCAAACTGAGGGTTGGGGTGCTTGACAGAGATGACCGCTGTCAGTCCCTCACGAACATCTTCCCCAGTCAGATTGTCTTCGTTTTCTTTGAGAAGCTTGTTTTTCTTGGCATAGTCATTGATAACCCTTGTCAGGGCTGTCCGGAAACCTTGCTCATGCGTTCCACCCTCGTGGGTATGGATATTATTGGCAAAGCTCATGACTGTCTCGTGGTAGCCAGTCGTATACTGCATAGCTACTTCAACCGTAATGTCGTCCATTTCGCCATCAGTATAAATCGGCGTTTCAAAGATTACATCCTTGTTTTCATTGATGTATTGGACATAGCTGGCAATCCCACCCTCATAGTGGTAATCCTTGACCTGCTCCATTCCATCTCGCTTATCAGTGATGGAAATTCTGAGACCACGATTGAGGAAGGCCAGTTCTTGTACCCGCTTATTGAGCTTTTCAAAGTCAAATTCTACTGTTTCAGTGAAAATCTCTGGATCTGGTGTAAAGTGAACTGTCGTACCAGTACGGTCTGTCTCGCCGATGATTTCTAAATCAGCGACTACATGACCGCGACGGTATTCTTGGTAATGAATCTGACCGTTCTTATAAACACGGACATCCAGCTGAGTGGATAGGGCATTTACAACGGAAGAACCTACACCGTGCAGACCTCCCGATACCTTGTATCCGCCACCGCCGAATTTTCCTCCGGCATGGAGCACGGTAAAGACGGTTTCCACGGCCGGACGGCCTGTTTTCTCCTGAATATCAACCGGAATTCCCCGACCGTTATCCACTACTGTGATGGAATTGTCTTTTTCGATAAAGACTTGAATGTGACTGGCAAAACCAGCCAGCGCCTCATCAATTGAGTTGTCAACAATTTCCCATACTAAATGGTGAAGACCTTCCTTGGAGGTCGAACCGATATACATCCCCGGACGCATACGAACGGCTTCTAAGCCTTCTAAGACCTGAATCTGACTGGCATCATATTCCTGGGCCTGTATATCTTGCTGCTTTTCTTCTGTCATAGTGTTCCTTTTCTAATCAATATCTATAAATTCTTCAAGATGCTGCATATTATCAAAGAGATAGGTAACAAAGCCAGCTGCCTGCCCTGCTTCAATATCCAGTGGACGGTCACCAATGACCAAACCAGAGACAATCTGATACTTGTCTTTTAGATAGAGCATGGAGTCTGGTGAGGGTTTCCTTGGAAAACCATTCGCCGATGTCACCACTTCTGTAAATGCTGATGCAATCGCAGTTTTCTCCAAGATTTCCAGCACTTGATTGTCCCGGTGGGAGACCAGAAAGTTCCGACCACCCTTGTCCACAATCCGCCTAAGCAAGTCTGCCGCTCCGTCAAATAAAACAGGGTGACTCAACTCTTCTGCTTCATTGGCCTTATAAAACTTCAAAAAGTCTTTTTCTTGGGGAGCAAACTGCTGGACTGCATAGTCCGTAGACACCTTGAGGGCCTTATAAACTTCGTCGTGACCAGCTTGCAGACCAAACTCTTTCAAGGTTTGAACAAAAGCTGCAGTCGAAGTTTCATAATTATCCAAGAGTGTTCCACCTAAATCCCAGATGTAATCTTGATAATTCATACCTTTCATTATACCATAATTTTCATGAAAAAGGGGCTAATATTTGCCCGAATCCAGCACTTTTCCGACTTTTTTCAAGCCTTTTTCTAAACAAAATATCTCAAAGAATCCAAAATTTAAA

>c183_g3

ACCCTCCTTGCTTGCTTTTAATTTCTTCGTGCGCGGCAGCAGTTTGGGATTTTCCTGAGCCAGTCGGACGCGAGGATTGAGCCAAATCGTAAGCAGCTGAAAGACTAGATTTACAGTCAAGACTCCCGCTGTGATAAAGAGGACCGCACCCTGAATGAGGGGATAATCCCGCTTGGCAACTACGCCAATCAGCATCTTACCGATGCCCGGCCAGGAAAAGAGGTGCTCGATAATAGCCACCCCTCCTAGGAGCGAGCCCAGAGAGAGGCTGACTAAGGTGAGAAGAAAGGGCAGGACATTGTAAATCACGTCATGCAACAGGATATGTCCCTCTCGAATCCCCCGACCACGCGCACCTTCGACTTCTGGCGAATGCAGCACTTCTATCAAAGCGGTCCGCAGCTGCGGGATATAACGAGTGGACATAATCATGGCCAGTGTTATGACTGGCAAGACTAGACCCAGCTCATTAGCCGTTGCCTGAATGGGAAACCAGTGCAGCTGAACAGAAAAGATCAGAATCAGGATAATCCCCATAACAAAGCTAGGAATGGCATTCAGAAAAGCCAGACCTCCCATCAGGAAGCGATCAACTGGCTTTCCTGCATGGTAGGATGTGTAGAGAGCTGTCGGCAAGGAAATTCCCAGAGTTGCTAGCAGGGTATAAAAAGCTAGGTAGACTGTATTGGGAAAATAGAAAACCAGCTGCTCCCAGACCGAAGCTCCGGAATTATAGGTCACACCAAAGTCGCCATGGACAATCCTGCCCAGCCAGGCCAGATATTGCTCCATAAAGCTGCCATTCAGGCCCATCTCAGCCCGCTTGATTTCCAGCAATTCCTTGGTAAAAGGAATACCCTGAGCATTGAGGATACTCTCAGCCGGATCTCCCGGTGCTAGATAAACCAGCAAAAAGGAGATAAAGCTGACGCAGAGCAGGATGAGGACGAATTGCAGAATAGTTTTGATAATAAATTTAATCATGCGCCTCCCTTTCAGATGTCTGGTAGCTTTCAGGCTTCTTGCTGGCTACGGCCAGCAGCACTTCGCCTTGGTGGACGACCAAGGTCGCTTTCTGGTAGAGGACCAGACCGTCTTCCTTGGCAGTCACCTTTTCTAAAATATTGCCATAAATATCACAGATTTCGCCAATGACCTGACCAGCCTGTACTGTTTGATTGGGTCGGACAAAGCACATCCAGCAGCCCGACCTTTGACAGGTCAGATAGTAACTGTCGTCAAAGATCAGCGGCGCCCGCTTATAATAGGGCATGCTCCCTTCAAAAAGGTATCGGGCGACTTGTTTGATCGACTCCTTCATAGCCAGCACATCCTCTGGCAGACAGGTGCCATTTTCTCCCTGCTCCAGCAGGATGCTAGGCAAACCATAGTCCACCGAGGCAGCGTGATAAAGATTGCCACGGTCCTGAGATCGATAGATAATCGGTGTCCCAGAAGCCTGCAGCATCTGATAGGACTTTTCCATCACCTCTGGATTTGCCCGCAGGGAATAGTAACCATGAGGGGTCAGCAGTTCTTCCCGATTGCCACTATGCAGGTCAATGAGGAAGTCACTGACACTAAAGACCTGACTCTCAATCACAGACCGAATCTGATAGCTGAGGCTGGAAACTGGCTCTTGATCCTGAAAAATCCTGTTCAGATTGAGGCCGTCCTCAGGTACAAGTGTCGTCTCCCGAGCCCAGAAGCCGCTGACATTGACTGCATGCAGGAGGAGGACAGAGCCTTGAAAGTTAAAGTCCCACTCATGCGCTAAATCCATTAGCGCCTTGACACCGACATACTCGCAGCCATGGACTGCAGCACTGATGGTCAGCAAGGGCTGGGAGGCCTGTCCTTTGAGCAGAGTGTACTGGATAGCCAGCTCATCCGTGATGGGCAGACTTCCTTGATAGCTGCTGCATGGAGCCATAGAGTCTAGTTCTTGCTTAATCCTCATCTTTATCCTCCCTGTACTGACTGAGCAAGAGCTTGGTATAGTCACTCTTGGGAGCAGAGACGACCTCTCGCATGCTGCCCTTCTCCTGCACGATTCCGTCCTTGAGGACGACCAAATCTTCCGCAAAATTGCTGACCAGAGCGATGTCATGAGAGATGAAGAGAAAGGAGGTCTGCGAGCGTTCCTTGATATCTGCCAGCAGCTGCATGACTTCATACTGGACGGTCACATCCAGAGCACTGGTGATTTCATCGCAGATGAGCACATCAGGATTAATCAGCAGGGCACGGGCAATGGCCGCCCGCTGACATTCTCCTCCGCTCAGCTGATGCGGCAGCCGCTGAGCAAGCTCAACTGTCAGACCGACCGAGGTCAGCAAGTCATGGATGGCCTGCTTTCTATCTGACTGCTGCTGATAGAGAGAGAAATTTCGACAGACCTCTTCTAGGCTCTGCTGAATGCTGCGCTTGGGATGAAAAGTCGACTGGGGCTGCTGGGCAATATACTGGACCCGAGCATAGTAGTCTCTATCTCTGTAGGCAGAGACTGGTTTGCCCAGTAGGGTCAGCTCTCCTTGGTCCGGCTTTAAAAAGCGGCAGATGAGTTTGGCAATGGTACTCTTACCAGAGCCTGATTCACCGACCAGACCCAAAGCCTGTCCCTTTTCCAAGCTAATGTCAATATCAAAGACACCGCTTCTCCGCTCGCCTATCTGACTGTACTCGTAGGTCAGCTGGCGACCGATCAATACTTGATTCATAGCTGCTCCCCTTCTCTAAACTCTGCCAGCTTGGGTACGGCCTTCAGCAATTTCTGGGTATAGGCCTGCTCAGGATGGCAGAGAATTTGATGGGCTGGACCACTTTCGACAATCTGCCCTTCCTTCATGACAACTACTTTTTGCGCCAGATGCTCAGCTACGCTGATATCATGTGTGACAAAGAGAATGGCCATGTTTTCCTCCTCATGAAGCTTGCTCAAGAGACGGAGCAGCTTCATTTTCGACAGGACATCCAGAGCGCTGGTCGGCTCATCAGCCATCAGCAGTTTGGGCCTGCTAGCTAGAGCCAGCGCCACTCCAACCAACTGCATCATACCGCCACTAAGCTCGAAAGGATACTTATTAACGACTTTTTCAGGCGGCAGCCCCACCATCTCTAGGCATTCTTCCAAGGGACGGAGATTTACTGCTTGCTGACCTTGATTTTTCACCAGGTCCTGATAATGCTTTTTGATCTTACGGCGGTTATTGAAGCTGAGACTGGCATGCTGACTAATCCAGGCTACTTCTCGGCCAACAAAAGGCAGATAAATACGGTGGTCTTGGGGCTTGATTTCCTGCCCCTCAAAGGTAATACTGCCCTCTGTTACTGTCAGCCCCCTGAGAGGGAGACCCAGCAGCATCTTGAGCAGGGTGGACTTGCCACTGCCACTCTCGCCCACAATCGAAAGGGACTCCCCCTCAGCAAGAGAGAGGGAGACCCGATTGAGAATGACTTTATCTGCTGACTGCACCAGAAGATCTTTTATTTCCAGCATGTTTTATTCCTTTGATAGTTTGTTGCTGACGTGATAGTAGTCTGTCGGATGGGATTCCAAACCAGAGACGGACTTGTCCATCACCAGAGCGACCTTGAAGAAGCCGATAATGGTAAAGCCGTAGTCCTTATCCATGATTTCTTGGATTTCCTTGCTGAGCTGATTGCGCTTAGCTGGATTAGTTTCAGTCGCCAGCTCTTCGATTTTCTTGTCTGCTTCTGGATTGCTGTAGTGACCGATATTAGCTGCACCGTTTGTCTTAACAGCACTGTTAAAGAAAGCATATGGATCTCCGATAGGAGCTGCCACCACCGCATAAGGTGTGAAGGCATAGTCCTTTTCACTGGCAACGGCACTGACTTCAACTTTCTTAATGGTCGCTTCGATTCCAACTTCCTTGAGCTGTTGTTGGCTTGCTTCTACAGCCAGCGGCATTTCAGGCAGACGGGTAAAGGACAACAGCTCAATGCTGACTTTTTGACCGTCTTTTTCACGGTAGCCATCGCCGTCAGTATCCTTGTAGCCAGCTTCATCCAAGAGTTTCTTAGCTTTTTCTACGTTAAATTCATGGACGCTCTTTTGCAAGGCAAAGGCGAAGCCAGTAGGGAAAGGTCCAACGGCAGGCACAGCCGATCCTTTAAAGAGGGATTCTGAGTAGGTTTTCTTGTCTACCAAGGTATCCAAAGCCTGACGGAACTTATCATCTGCTACATAAGGATTTTCAAAGTTATAGTAATAAGCTAGGTAGCGGGAACCTTCTACCTCAGAGATTTTGTAGTTCTTATCGGAAGCATAGGTACTTAGATTTGCATAAGGCAGACCATAGACAGCATCCACTTCCTTAGACTTGAGAGCTGCAGAAATAGCAGTCGGATCAGAGAAGTATTTTATCTTCAGATTAGCAACTTTTGGCTTGCCATCCCAGTAGCTCTCATAAGCCTTGAGCTCTGCTCCGCTCTCAGGAGTGTACTTGGTCACCATGTAAGGGCCTGTTCCGACAGGAGCCTTGTCAGATGCGCTCTTACCAGTCGTATCGACAATGGCGGAGTAGGGCTCTGCCAAGAGATTCGCCATGATAGGCTGCACAGCCTTGGTCTTGATGGTCACAGTCTGACCCTCCGCAGAAATGCTGTCAATGCCCAAATCGGCTGCTGCCCGCTCACTCTTCTCAACCAGGCGTTCCAGAGAGGCTTTGACTTTTTCACCGGTCATTTTTTCACCGTTTTGGAAGACAACCTTATCCTTGAGGGTGATTTTCCACTCTAGATCTGATACAGCTTCGATTTTCTCAGCCAACCAAGGTTTTACTTCAAGTTTGTCGTCCATCTTAAAGAGGGTTTCTCCCACTCCGTAACGGACCGTGAACCAGCCATTGTATTCCGCTGCTGGGTCTACATTTGCTGGAAATTGAGTGTAGCCAATGGTTACAGTCTTACTGCCACTATCGCCAGTAGGCTCACTGGTTTTACTAGGGTTTACACAGGAGGCTAGGGCTAGGCCTGCCAAGGCTAGAGCCATAATTTTTTTAAAATGTTTCATAAAATAATCAAATTCCTTTTCTATTTTGTAAACTAATTTAAGACGGTCTGTCCTAGTCAAATCTAAATACGCTTCGCTCTCTGTCTAAGGTGGTTCTATGTCCTGAAGATGAGAGAGCAGACGAGTGAAAAACAAGTGATTGCTCATTATTTGTCCTCCTTCTGGCTTGGTATGTCTTAATCTTTTCCTTCCAGATAGCTTCGAGCATCCTCGACAAAGGCAGTTGGCAAAGCCAGGTCAGCCTGCAACTCATCGCGATAAGCTCGGATGACTTCTTCCTCTCGGCCGGAGACAACCTTTTCAGCCCAGTCTGGGTCTAGGAGCAGGGCCTTGCCGAGGGCAAAGAGCGGAATCCCCGCATCCAATACTCGCTGGGCATCCTGCTTGGTCTTTATCTGACCGACACCAATAAGGGGTACTCTACCATTAATTTTTTTGATAATTTTCTGAATCACTGGCTCTGAGTCCTGAGAATCTCTGATAGACGAGCGCCAGACATCAGAGGTCGAAATATGCAGATAGTCCACCTGATGATAGATAAGCTGCTCCAGCAACTGCAGGGTATCATAAAGCTGGATTCCTGGCTCTTCAATCTCTTCCGGAGAAAAGCGGTAGCCAATCAGGAAGGGGCGGTCAGCCTCTTCTTTGACTAGTTGCTTGGCTCTTTTCAGCAAAGTCTTTGGGAAGCGCAGGCGGTTGTTAAGGCTGCCGCCCCACTTGTCCTGACGGACATTTGAATGGGGAGAGACAAACTGCTGGATCAGATAGGTATTGGCGCCATGAAGCTCGACTCCGTCAAAGCCAGCCTGAATCGCCCGTCTGATAGCTGACAGAAAGTCCTCTATCACCTGCTCCACTTCAGCGTTCTTGAGCGCCCTCGGCTCTGCCAAATAATCGCGCGGAGCCTTGACAGCGCTGGGTGCTACTGGCTGACCGTCAATCAAATCAGGCAGAACCATACGACCACCGTGATAGAGCTGGACAATAGCCAGAGCCCCTTGGTCCTTGATGGCCTTGGCCAGACGGCTAAGCCCCTCAATCTTATCGTCCTCGGCGCCGCTGAAGCTCTCCGCAAAGGACTTTCCCAGAGGATGGACATAGGTACTGCCAGTAATGACCATACCAACCGACCTAGACCGGCGGGCAAAAAAATCAATATCTGCCTGAGATACATAGCCACCCGGCTCACTAGCGCAGATAGTCATAGGAGCCAATACAACGCGATTGCGCATGGTCTGACCATTTTTAAAAGTAAATTCATCCTGAATCTGTGTATTCATCTGGTCACCTCTTCCCCAAGATAAATCATGTTGTTTGTTTTAAATAATAAGCCCTTAGAGTAAGGTCGCCTGCCAAGTCTGGTCCCGCTCTTCCAAGAGCAGCACTCGTCCTTGGGGGACATCAATGTTCCAAAAAACAGCCTCTCCATCAACCAAATGCTGGTAAATCAAGCGAAGGACTCCCAAATGAGCCACTAGAGCCATTGACTCATCAGCACTATCTAGCAGGCAGTCTGTTGCTGTCCAGACTCTGGCCTGAAAGTCTGAGAAAACTTCTGCCTCAGGAGGCGTGACCTCAAAGGGGGCTCCCAGCCAAGCTTGCCAGACTTCTGGAAAGGCTGCCTGAATCTCATCAGCTGTCAAGCCCTCCCATTGTCCAAAGCCCCGCTCGTCAAAGTCGCCTATGGACTGAATTTGCCTGTCTGGATAAGCTAGCTGGGCTGTTTCCTGCGTCCGTTTGAGACAACTGGTGTAAATCACATCGACTGGGTACTCCTGCATCAAAAGTTGTAGCTGCTTGGCATCTTTTTGGCCTTGCTCGTTGATAGAGACATCATGGCTGCCGTAGAAACAGCGCCTGCGGTTGTAGTCTGTCTGACCATGCCGCATCAGATACCATTTTTTCATCCTAGTACCACCAATCCTAAGAGATAGAGAAGCTGGGCAATTTCGACATAGGCCCCTATCGTATCGCCTGTGTGACCGTCAATTTTATTATAAACGAACCAGCGATAGCCGATTGCTCCCAGAAATACCAAGCCGTAAGCCAGAAGGCCTCTCCAACTGAAAACTAGCAGAGACAGGAGCAAGGGGAGAAGCTGGGCAAGCAGGATATGACTGGTCTTGCTGCCACTGAAGAAATTCCCCGAGCCGCCGCCTTCTCTGGCATAGGTCATCCGGTAAAGCTGTAGACTCAGACCTGCCTTGCCAATCATGGTCAGACTAGCCACGATAAACCAGCGAGGCTCTGGCAGATAAGGGTAGAGAACCAGCATCAGAGCATAGTAGAGAATGAGCGCCAGAACGCCATTGCTGCCAATCCGACTGTCCTTCATAATCTCTAGCATCCGCTCCTTCTTCCGCGAGGAGAAGAGACCGTCTGCCGTATCCGCTAAAGCGTCTAAGTGAAAACCACCTGTCAGCAGAACATCGAAAGCAAGGGTCAGAACCCAAGCGACCATCCCTGGCAGGACTAGGCTCGTCAGGAAATAAAATCCACCTGAAATCAAACCCAGCAAGAGGCCAAAGAGGGTCAGAAAAGGAAGTCCCCGTCGCAAATAGGAAATATCCACTGCCTTTGGAATCACAATCCGGCTAAAAAATTGGGTATAGATAATCAATGCCTTTATCATTTCAACTGCTGAGCAAGGCCGCAGATGACTAGATAAGCCTCGCTCGCCTCCTTTGCAATCAGTTGGTTAATCTTGCCCTGCACATCACGGAAAAAGCGTCCTAGTCTGGTCTCTGGGACAATACCCAGTCCAACTTCGTCCGTTACAATCCAGCACTCAGCATCAGTCTGATGGATTGCAGATAGGAGCTCTTGCCATTCTTCTTCCAAGAGCTGCAGCAGGAAAGACTGCTCCTGCCGACTGAGAAAGTGTTCCTCGGTCAGCTCCAGCTTGTCTGGAAAATGCTGGGCAATTAAATCAAAGAGGCGATTGCTGGTCAGAAGGGTGGCACAATCCAGCAGGTAAACTGGATGCGACTGTTCTCGCAGCCAGTCCGCCAGCCCTGCATACTGTTCCTGAGTTGTCCAGGAAGCCGGCCGGCGTTCTTGGTGCAGCCGAATCCGCTCCTGCCATTCTGGATCCTCTCCTCGAGGCAGGCCAGTCGCAATATAGCAAACTCGCTCTCGGTCAGCCAACTGCTCCTCTGCAAATGCCGACTTGCCACTTCTGGCACCGCCTGTCACTAACACAATCTTAGCCATTTGCTTCTTCTTTCATTTCTTGAAATGCCTGCTCAAAGGCTGCCAAGGTCTGGTCTAGATCCGCCCTAGTATGGGCACTGGACATAAAATTGGTTTCATACTGAGATGGCGCCAGATAAATGCCCTCTTCCAGCAAGAGACCGTGCAGTCGTGCAAACTGTGCATGGTCAGCCGCCTTAGAATCCTCAAAATTGCGGACTGGCTTCTGGCTGAAGAAGAAGCCAAACATGGTACCCTTGGACACGACTTGCAAACTAATACTGTACTTGTCTGCTAGACTTCGCAATCCATCACAGAGGTAGCTTGTTTTCTCTTCAATCTCAGCAAAGAGCTCAGGTGTTAGCTGTTTCAGCGTTTCGTAACCAGCCGTCATGGCGACTGGATTGCCCGACAGAGTACCAGCCTGATAGATGCTGCCTAGAGGCGCTACCTGATCCATATAGACGGCCTTGCCGCCAAAGGCTGCGACTGGGAAGCCGCCACCGATAACCTTGCCCAAGCAGACCA

>c183_g30

GTTTCCTTGTTAGAATCTAGTATACCATATTCGGCTTTTTCGTCCTTAGTCTGCCTCCAGATAAGCCTGGAGACGGTCAATTCCTTCGTTTTCCGTCGCTGATATTTCAAAAATTTCCTTAGCTCCAGCCGCTTTCAGCTGTCGCCGTGCTTTTTCAATCTGCTCTTCCTTATCAGCCATATCAATCTTGGTGACAATGCCAATCACTTCTTTATTGAAGAGGGAAGAAAATCCTTGAGGAAAGGTCTGCATCTGATTGGAAGCCGCCACCAAGAGACCAATCACATCAGCCTCCGTTGCTGTCACGTTCAGAGCATTATAATACTTACGATGCTGGAGAAATTCTCCAGGCGTGTCAATGATGGCATCATAGAACTCAATAGCCTGAGTCTTAAAGTAGCTTAGCTCCAAGCCTTTCAGTCGCTGGGTCAGAGTAGTCTTACCCACTCCGACTGGACCGACAAACATGATTTTTTTCATATTTT

>c183_g31

AGACCCAGTCGGCTTCTGGCAGAATTGCTTGGACAGCTTCAGGCAAAGGTAAAGACTGACAGCGAATGAGAGGAACATGATGCGTCTCAAATCCAGCCTGTCTGATCTTTTCTAGCCAGGCAGAGTCTGGCGCCTGCTCTCTGGTAAAGATTATTTTTTTAACCATGGCATTCCTACTGCTCCCTTATCAGCTAACTGGCGCACAGCCTGCTCTGCCAATTGCTGGCCATCCTGCCCTTGAAGGCTGACAAAGATGCATTGGCCGTCCTCCTTGGCCAGCATGGCCTCTAGCTGATAGCCCTGACCGTTTTTCTGGGCAAAAGCAGCGATAGGGAAGGTGCAGTCCGCATTCATCTGAGCCAAAACTGCCCGCTCAACTGCCACTTCAGCTGCTGTCTTTTCGTCCTGAACTGCCGCTAGTAAGCTCAGCAGTTCCTCATCCTCTTCCCGGCATTCCACTGCCAAAGCTCCCTGAGAAATAGCTGGCAGGCAAAGACTGGTCTCTAAAGGCTGAATGTGAAGTCGGCTTTGGTCCAGCCAGCCCAAGCGTTTAAGACCGGCCATAGCCAAGACAATCGCGTCGTACTCGCCTTCTTCCAGCTTCTTGATGCGTGTATCAATGTTGCCCCGAAGTGGCTTGAAAGCCAAATCCGGTCTCTGAGCCTGCAGCTGAACCTGTCGGCGAATGCTGCTGGTTCCTATAAGAGAGCCCTTAGGCAGGTCAGCCAATGTCTGGCCAGCTTGACGGAAAATCAAGCAATCTCGAACATCTTCTCGCTGACTAATCGCACCGAGTGCACAGCCCTCAGCTAGCTTAGCCGGCATGTCCTTGAGACTGTGGACCGCCATATTAATCTCACCAGCCAAGAGGGCTCGCTCGATTTCCTTGACAAAGACTCCCTTGCCGCCAA

>c183_g32

AAAATTTAGCCTTTATAGTAGAATGGTCATGAATCTATATACCAAGGAGAAGACAACTTGAAAAACAAGAAGATAAAAGCTTTGCTGTTAGTAGTGCTCTCATCCCTCTTCATCCTAATTGGCTGCAGTGGCAGTCCCAAAATTCAGGGAAAATGGAATGTACAGGATGCTAGCGGTGAACAAAAGACCATTGAAATAAAAGACAAGACCATTATTGTCAACGAAGAAGAGTATGAGTATACTCAAAATGCAGTTGGTTTTAAGAATGGAGTGAGTTATTACGGCCTGACTCGGAAAGATAATGGCGGAACATTTTCTATCGTCTTTCCTGAGAAAGATAAAAACATTGCTATTATGCTAATTCCTGACTCAAACGATGACTATCTCACAGGCAGTATGCTCTTTGC

>c183_g33

GAAGAATCTTCCTTTTTGCCACAAAATCAGAAATTCCACCTGTAAAAATTCGCTTTTTGTTCTTTTGGGACTGATTTCACATGTGAAATTAAGTATTTTGAGCTCTGGGAAGCTCATTTCTCACAGAAACTAGGCATTCTTACACAGGTTCTTTTTCCTTCTCACACCTTCTCCGCCAGTTCTTCCCATTCTAACATGGCTTCTTCTTGGGCGGCGGTCAGCTGGTCGATTTGCTGCTGGCTCTCCATGAGTTCGGCGGCGTCATTAGTGGCCTCCATGGCTTCCTGAAGCTGACTGATCTGGCTGTCCAAGTCCTCGATTTGTGTTTCCAGTTGTTCTAGGCGGCGGGCCAGCCGGCGCTGTTCTTTCTGGTTTTCCTTTTGAAGCTGATAGTCATTGGCAGGTGCTGGCTTGTCAGGACTGGACTGCTCCGTCTCCTCACGCAAGGCTTCCTGCTCTGCCTTTTTCTCCAGATAGTAGTCATAATCACCCAGATAAAGGGTGGAGCCTGTCTCAGAGAGCTCAACAATCTGTGTGGCCACGCGATTGATAAAGTAACGGTCGTGGCTGACAAAGAGCAGGGTGCCGTCAAAGTCAATCAGGGCATTTTCTAGCACTTCCTTGCTGTCAATGTCCAGATGGTTGGTCGGCTCGTCTAGGATGAGGAAGTTGTTGTTTTCCATAGACAGCTTTGCTAGAAGCAGACGCGCCCGCTCTCCACCTGATAGCATGCCAACAGACTTCTTGACATCGTCTCCCGAGAAGAGGAAAGCACCTAAGCGATTGCGAATTTCGACCTCTGGCGTCAGCTTGAAGTCATTCCATAGCTCGTCAAGTACCGTATTGCTGGCAGTCAGTTTGCTTTGGGTCTGGTCATAGTAGCCCACTTCCACATTAGCTCCCAGCTGCTCCTTGCCTCTGATAAAGGGCACCTGACCGATTAGTGACTTGATCAAGGTCGTCTTTCCAATCCCATTTGGCCCGACAATAGCTACCGCATTAAACTTGCGAATGTCAAGGTTAATCGGCTCCGACAGGACTTCCTCAC

>c183_g34

GAGGTCTGTTACCTGAGTGCTAGCATGCCCTAGCTGGTGGCTGACGAGAACTTGAGATTTGGTTGCATCGTATAAACGTGTAGCAAGCGTGTGGCGGAGCTTGTGAGGCGTCACTCGCACCTTGAAGTCCTCAGAATACTTGGCTACCATTTTTTCGACACTGGAAGCATCGATTCGATTAGGAATGCCGCGGTATTCAGTCAAAAAGAAGGCTGTATCAGTCTTTTCGGCCTTGTATCGCTTGATCCGAATGCTCAGATACTCTTCTAAATAGGGCTTGGCAAAAGCGGCAACATTGACAGAGTCCCTTTTTCCGCCTTTTCTTGTTACTTCAATGACCATCATTTTGAGATTAATGTCTTTCAGGTCTAGATTAACAGCTTCAGACAGACGAACACCAGAGGCTAACAGCAAGGCAATGATGGCTAAATCTCGTTCCTTGTTCTTGTTAAAAGAGGACAGGGCGCGATTAGAGAGTTTCTTAGGATACTCTGTATCGATATATTGGAGAAATTCTTCCGTCTCATCCCCTAAAAAGAGCTTCTGCTTAATATTCTCTGCACGAGCGGCCAGAGTTTCCTTTTTCTTTTTAGTAGCAACCTTTTTCATCACATTTCGATAGAAGTAGGGCTCACCCTGCTCGTTTTCGACTTCCTCAGTCAAATATTTGTAGAGGCTGGACAAAGCTGACAGAGTTCGGTTTATGGTTGTCTGGGAAACACCGTTTTGAGTCGTATTGGCATTGAGAAGCGGCCGCTCTCGCAGATACAGAATGAAGGCCTCCATGTCTTTCTTGCTCATATTTTCCAAGACTGAAAGAGGAATCTCAGCAATATGAGAAGCATCCGTGATACCAGATTCTAGCACCCAGTTAAAAAAGCGATCATATTCTTTGAGATATTCGTATAAGGTTGTAAAACTATAAGGCACTGCCAGCTTAGACTGGTAATATTCCAAAATGTACCAGGGCATGGTAGCCTTAAGTTTATCAATTCTTTCTAATAACAGTTCACGTCTCATTATATTTCTCCGTTTTTCTATAATAGTAG

>c183_g35

GTACGTTAATTTTCTAAAGATGAGGTTTCACTTGTCTTTCTGCAAAGGAGCAGAGTCAATAATTGGAAAGCTAGTGCTAATCAACGAAGCTTAGCATAAAGATACAATGATGTATAGGCGGCTTTCTTTTTTTAGAAAATCAAAATAAGTTCTGTCCTTCCAAGACTTGGTAAAGTCAATTTGGAAAGACAGAACTTTTTCTGTTTCTTTTTGTCACTGGCGTTTGCTTGCCCAACGCTCAACATCTGCCTTGGTGATATTGTGGAAGACCTTAGCACCTCTTTCATAGGCGATGTCTTCTTGATTTTTAGTAAAGTTGATTACTCGAGCCAAGGCAAAGAAGTAATCTGATAGACGGTTGACAAAAATCAGGACATTATTGTTAATTTCTGTAGTCCACATAGCCCCTACGATATGACGCTCTGCCCGTCTTGCAATGGTCCGAGCCACATGAATCATGGAAGCAATCTCGTCACCGCCCGGCAGGATAAACCTTTCCAGAGCCGATGGAATTTCTGCGTAAGTATCAATCCTCTCCTCAATCCAGTCAATCAATTGCTGATCAACCTTATAGGGATAAACTCCCTGAGGTGTAGAGAGGTCTGAACCACAGTCAAAGAGATAATGCTGCAGCTGGAGCAGCTCATCTCTCAGCTTATCATCCTTGTCCAGCTTGGTAATAGTATAACCAATCCAAGAGTTAAGCTCATCAATGGTTCCGTAAGCATTAACTCGTTCTGCATCCTTGGCGACACTTTGGCCACCAACTAGCTTGGTCAAGCCCTTGTCACCTGTTTTTGTATAGAGTTGCATATTTTTCCTCCTTTAAGTCACATTTTTGCGAATATCAACATAATCTTCTTCTTTCAAGCTGCCTTCAGCAAAGGTTGGCATGTGCTCCATGGTATAGACAGCATTTTCCAGTAGAAAGAAAGCCAAAGGACAGCCCGAGCCTTCACCCAGTCGCATGTCCAGCAAAAGCATGGGCTCAAGTCCTAGAAAGTCACTGACCAGTCTGTAAGCAGGCTCCGTCGAAGCATGGGAAGCAAAGGTATAGTCCAAGACATGAGGAGTCAGCTGATGGGCAATCAGAAGCCCCGTCAGAGAGATAAGGCCATCCACTACACATGGCAGCTGATAGCGGGCACAGGCAAGATGAGTGCCTGCCATAGCCAGCATATCCAGCCCGCCAAGCTTAGCTGTCAGATCCAGAATATCTCCATAAGGAGCGTGACAATCCAAACACTGCTGGATAACAGCTGTTTTATGAGCCTTCATATCCTGAGTCAGACCGGCTCCATAGCCTGTGACATCTTCTGCTTTAAGACCGAGAACTGCCGCAATGACAGCAGCCGAGGTGGTGGTGTTGCCAATCCCCATTTCTCCAGTTCCAAAGAGACGATAGCCGTCTTTGATCAAGGCCTCTGTCTTCTTGTAGCCGACTAGAATTGCCGCTATAGCCTGCTCGCGAGTCATGGCCTGCTCCTTGAGCATGTTACGCGTCCCATGACAGACCTTGTCCTTATTTTCCTCAAAGATATCCTTCTTGCAGCCAATGTCTACCAGGCAGATGTCTGAACCTACATGTTTGGAAATAGCGCACAGGCCTGACTTCCCTGCTAGAATATTGCGAGCAACAGTATAGGTCGTTTCTTGAGGATTGGCAGAAACGCCTTCAGCCACAATGCCATTATCTGCCACGTAGACGAGAACAATCTTCTTCTCTAAATCAATGGGACCAGAAAACATGGCATGCAAGCGAGCATAGATGGTCTCGAGCTTCCCTAAGGAGCCTGGCGGCTTTCCTAGCTGATCACAGTAGCGCCGGCCTTCTTGAAGCTTGTCTTTGTCTATCGGAAGAATCTGCTCAATAATTTTTTCTAAGTCTTTCAAACTAGTCTGTCTCCTCCCTGACCGGCTGCACCCCTTCAAAAATAATCCTGCTCAAGGTCCGAGTCTGATAATAAGCCCCACTGGCTGAGCGAGCTAGTTCAGCCTCCAATTGAGGCAAGTTTTCTAGCTGTTCCCTGCTCAGCAGCAGACCAACCAATGTGCCACTATGGGCAACATTGAGCCCTAAGCATTGGTATTTTTTTACCAAATTCAGTAATTCTTCCAGATAGGGCTTGGGCAGCCGTTGGTTATTCAACAAAGCACTATAACTCGCTAAATGGCCAATCTTTTCCAGACTCTTCTCCTGACAAGCCTCCTGAAAGAGAGGAAGCAAGCGTTTGGACTCCTCAGCCGGATAGCTAGGACTGTCCTTCATCCGAACCAAGTCAAGGGTAGTCACCATCTCCACAGGCTCCAAGATATAAACATAGAGCTCCGGTTGCCAGTCTGTCTGCCAGACCACTTGACCAGTCAGGGGATTAATGACCGTCCAGTCCTCAAAAGCCACCGAGTCAGTAGGCTCAATCTTGGCACAGAGACGAGTTAAATCTGCTGCTCTTAGAGGTTGCTTCTGCCCCAGAGCAGCTGCCTGCAGGCAGCTAACCATATCTGCTGTGCTGCTAGAATAGCCCTTGCTGATAGGCAGGTCGGACTCCTGAACAAAAGAGAAAGCATTGGACTCAGGCAGGAGCTCCAAAGCTCGCCTTACCTTTTCACCTTGGTCTTGCCTTGCGAGAGACGAAGCTCCGTCCGATCTCACTCGACTGCTCTTCTCAATCCCATAGGAGAGCAGGACTTCCTGCTCTCCAACTAAACCTTGAAATAATTCACCGCAGGAACCTGGACAGGAAACCATTACCTTAGTCATAAGGAGCCTCTTTCGCTAAGACTTCTGTCAGACAAGCCAGAAGCTGTTCATTTTCCTGATGGCTGCGGATGGCTATGCGGTAATGCCGTTCTGACAGATCATGATAATTTTGACAGGAACGGATAAAAATCTTTCTCTGCCGAAGCTCCTGACGCAGGTCCAGCCGACCTAGATACTCAAAGAAGATATAATTGACACTAGGCTTGACCGGCCGAATCTGTGAAAATGCAGTTAGTCCTTGAAAAAGGAAATCTCTTTCTACTCGAAGCCACTGCTTGGTAGCTTGCTGATAGGCCTGATCTTCCAAAAGGACAGGCAGGGCGTGATCCGCCATAGCATTGACCGACCAAGGAGCGCGGCTCCCCTCTATCTCATCAAAGCAAGTTGGATGACAGCTGAGAGCATAGCCCAGTCTCAGACCGGGAATAGCATAAAACTTGGTCAGAGACCGCACCACTACTGCATTTGGATAGGTGGCCAGACGTGAGACGAAGCTATAGTCCTCCTCATCGTCCAGAAAGTCCATAAAAGCCTCATCCAGTATCAAAAAGATTTGCCGCTCCTGCAGGTCCTCAGCTAGCTTTTCTAGCTCAGTACGCCGAATCAAGGTACCCGTCGGATTATTAGGATTGCAGATGAGTACAGCGTCTCCAGCAGTTAGTGAATCCAAGGCCGGCATCATATCAGCTAGATTCCACTCATAGGATGGAGAAGGGAGGCTAAAGCGCTCCACCTTAGCCTGCACTTGCGAAAAGGCTTTTTCATATTCCATGAAAGTAGGGCTCAAAGTCAGGACAGTTTTTGGACGCAGGAAGCGGGCCAGCTCATAAAAGACTTCTACCGCACCATTTGCCAACAGGACCTTGTCCTTTTCTAGTCCATGATGATGTGCCAAAAGCTCTCTGGAGCGACTGTAGCTGATGTCTGGATAATGAACCAGCCAATCAATGGACTCGGTCAGACAAGCCCTCAGCCGCGGAGAAATCCCCAAAGGGTTGATATTGGCACTGAAATCCAGACAGTCTTCTAGAGAAAAACCAAATTCCTCAGCCAAAGCTGCCGCATTTCCACCGTGCTCTACTTTCATAAA

>c183_g36

AAGAGAGTTACAAATAATAAATGATAGCGATATAATCTATCAACTTGGAAAAAAAGAATAAAATGTTTAATTTTAGATGAAGAGGTACCCCATGAACGAAATCAAATGCCCCAACTGCGGGGAGGTTTTTACTGTTAATGAGAGCCAGTACAGTGAGCTTTTGTCGCAGGTTCGGACAGCAGAGTTTGACAAGGAAATTCATGCTAGGATTGAGCAGGAATTGGCTTTAGCAGAGCAAAAATCCCAGAATGCCCAACAAGCTCTGCTATCGCAGAAAGAGCAGGAAATTAGCAACCTTCAAAGTCAAATTGCTCAGTTTGAGACCCAACAGGAATTGGCAAAGAAAGAGGCGGAGCAGGTAGCTAGTCTTCAATTGCAGGAGAAGGATAAGGAAGTTCAGCAATTGGAAAGTCAACTGACAACTCTGCGCTTGGAGCATGAAAATCAACTGCAAAAGACCTTGTCTGCGCTGGAAAAAGAGCGAGACGAGGTTAAGAACCAGCTAGTCTTGCAAGAAAAGGAAGCAGCGCTGGCTCAGACCTCGCTCAAAGAGCGCTATGAGGTAGAGCTGCGGCAGAAAGATGAGACCATAGAGTTCTACAAGGATTTTAAAGCCAAGCAGTCCACTAAGATGATTGGTGAGAGTTTGGAACAGCACTGCGAGTACGAGTTTAACAAGAATCGTATGGCTATGTTTCCACGAGCGGAGTTTGGCAAGGACAATGATGCCAGAACCGGCAGCAAGGGCGACTACATTTATCGAGAGCTGGATGAAAATGGTGTAGAAATCCTCTCCATCATGTTTGAGATGAAAAATGAAGGCGATGAGACAGCGACCAAGAAGAAAAACGAACATTTCTTCAAAGAGTTGGATAAGGATCGGCGGGAGAAAGGCTGTGAGTATGCTATTCTGGTGACACTTCTTGAGACTGACAGCGAGCTCTACAATTCGGGTATTGTCGATGTATCTTATGCCTATGAGAAAATGTATGTTATCCGGCCTCAGTTCTTCCTCCCCATGATTACCCTCCTGCGTAATGCTGCGCTCAACTCGCTACAGTACAAGCAGGAGCTGGCCTTGGTGCGGGAGCAGAATATTGATATTACGCATTTTGAGGAAGATTTGGATGCCTTTAAAGTAGCTTTTGCCAAGAACTATCAGTCTGCTTCGACCAATTTTGGCAAGGCCATCGAGGAGATTGACAAGGCCATCCGCCGTATGGAAGAAATCAAGAAGTTCCTGACGACCTCTGAAAATCAGCTGCGCTTGGCAAACAACAAACTGGACGATGTCTCTGTCAAAAAACTGACACGCAAAAATCCTACCATGAAAGCTAAGTTTGAAGCTTTGAAGGGAGACTAGGAGGATCCTATGCAGATACGAAAAGCAACCATGAAAGATGCTGAAGCCTTACTGTCTTTATACGAAGACTTGGGCTATCCAACGACCGCTTCTAAGCTGGCTCGACGTTTAGAAACGATTCTTTCTCAGCCGCATTATGGCTGTCTTTTAGCTGAAAGAAACGGAGAAATTTTAGGTTTCTTAGGTTATGCAAAGCTCTTCTTTTTTGAAGCAGATGGATCTTACTATCGTATTTTGGCTTTGTCAGTTGCAAAAGAAGCAAGACGACAAGGAATTGCTAGTAGGCTAATCGATGAATTGAAAAAACAAGCGGTAAAAGAAGGAGTTAAGGCACTGACTCTAAATAGTGGATTAACTTCTGAACGAAATGCTGCACATCAGTTTTATCAGGCGGTTGGATTTGAAAAAGTGACTGCCGGCTTTGCACTGCATTTAAAAAGTCAACATGAATAAACATGATAAAATAGTAATGGAAACGAAGTAATGTCTTCAACAAAAGGGAAAACCTTATATTTTAACGATAAGAGCATGGACTATGTGACCTTTGGAAAAGGGAAGAAGCCTCTGCTCATCATTCCTGGTCTAGGGGATGGTTTGGCGACTGTTAAGGGAATGGCGCAAATGCTTGCCCTACCTTATAGGAAATTCGCAACAGCTTACCAAGTGTATGTTTTTAGCAGAATCAATGAGTTGCCAGAAAATTATACAACACGAGATATGGCGACTGATATAGCTGAAGCTATGGATGTTTTAGGTCTGAAAACGGTAGCTGTCATAGGAATTTCTCAAGGCGGTATGGTTGCCCAATGGTTAGCTGTAGATTTTCCAGAAAAAGTTGAAAAATTGATTTTGACAGTTACTACTGCGAAACTGAATAATCTTGGTAGGGAGCGGATTACTCGCTGGCTTGAGTTGAGTCAGACTGGAGCCTATAAGGAACTGATGTTGGATATTGCAAGTCACTCTTATACACCTAAATCCTTTGGAAAGTTTAAATACCTTTATCGAATAATGGGAAACTTTGGTCGTATTAAAGATAAACAGCGGATTGCTATTCAGACCATATCTTGCTTAAGACATGATAGTTTAGCAGTTCTGGAAAAGATCAACTGTCCTACGCTAGTCATCGGAGCAGAAGAAGATGACGTTCTAGGTGTAGAAGCTTCGCTCGAATTGCATCACCATATCAAAGATAGCCAGTTCACTATTTTGCCAGACTGTGGCCACGCACTCTATGAACAGCATAAGGATTTCCAAAAGAGAGTTTTACTATTTTTAGAAAGTTAACCAATGAACGGAATTATCAACCTAAGAAAAGAAGCGGGCATGACCTCGCATGATGCGGTATTTAAGCTGCGAAAGATTTTAAAAACCAAGAAAATTGGTCATGGGGGAACCTTGGATCCAGATGTAGTAGGGGTGCTTCCGATTGCTGTGGGCAAAGCGACGCGCTTGGTCGAGTTTATGCAGGAGGAAGGCAAGGTCTATGAGGGGGAGATTACTCTAGGCTGCTCAACTACGACAGAGGATGCCAGCGGAGACATCCTTGATCGGACACCAGTGACAGAGCTTTTAGAAGAAGCTCTCATTGATGAAGCGATGGAGTCCATGACTGGTGAGATTCGCCAGATTCCGCCCATGTACTCTGCAGTTAAGGTCAATGGCCGCAAGCTTTATGAGTATGCAAGGGCGGGTCAAGAAGTGGAGCGGCCAGAGCGGCAGGTGACCATCTATAGTTTCAAGCGCACCAGTCCGATTTCCTATGAAGATGAACAAGCCCGCTTTCGTTTTCGAGTGAAGTGCAGTAAGGGGACCTATGTCCGAACCCTGTCTGTTGATTTGGGAGCTAAGCTTGGCTTTGCCAGTCACATGTCCCAGCTGACACGGACTTTCTCAGCAGGTATGAGCTTAGATGATGCCTTGACCTTGGATGAAATAGCAGAGCGAGTAGCAGTAGATGATTTCTCCTTCCTCCAACCACTCGAATTAGGAATCGGAGATTTAGTGAGAGTTGAGCTCTCAGACGAGCAGGTTGAGGACGTGAGAAACGGTCGTTTTATCAGCTTGATGTCAGAAGAAGCTGAGCTAGCTGGCTTTTATAAGGAGAAGTTAATAGCCATTTTGGAAAAGCGGGAAGAAGCCTACAAGCCCCGCAAAGTCTTCCTTTAATATCCGCAGTAGAATTGTCAAAAGATTGTTACTCTT

>c183_g37

TTTTGAAACCTTAATTAGAGAAGGAAAGTTAAAAGGTATTGAAAAATCAGATAAAGGTAGTCCAGATGCAGAAATTGTTTATAACCTGCAACTAAAAAAGGGGGTGCTTCCGGATGACATTGAATGATAAGGAAATTCAAGAATTGCAAGGGATGGTTAAGAAGGAACTGGGAGCGACTGAAGGTACGGTATTCCAAATACCTAATCAAGATAAGTACTACCAAATTGTCCATAGCGTTGATATGACCACACAAGCAATGGCTGTTGTTCCAGTTGATGATAGGGAGGGACATAATCCCAATTTTCAAGAAACTACAATTGTAGTTGGAGGTACACAAGTTCCTTTTGAACATATGAAATATTGGGATAATCAGCCCTATTTTTGGAAGAAGGATTTTGAGATTACAGCATCTACAACAAATGCTTTTCTTACCAGTGGATGGTTTGGAATGGTACAGGGGGGATTGACACCTCAAACTGCT

>c183_g38

CAGGGAAGAAGCTAAAGATTTCAATCTGGCGGTAGATGTCATTATGCTGGCTGGGACACTGCTGCTACAGAGCGGATCCGAGACTTATCGGGTAGAAGACACCATGATTCGCATCGCACATTCACAAGGGATCATCGACTGCAATGCCTTAGCAATGCCAGTGGCTATTTTCTTTTCGATTGAAAACACAAATGTCTCACGGATGAAGCGTAATCTCAAGACCAACTACAACATCGAGAAGGTCTGCGACGTCAATCAGGTCTCCCGTCAGCTAGTAACAGGAGAAATCAGCCTTCAGGAGGCTTTTGATGAGCTGAATCGTCTCAAAGTTAAGGAGCTGCCCTATAACAACAAGCAGCTGATCGCTGCCGCAACGCTCAGCGCTCCCTTCTTCTCCATAATGTTTGGCGGAAATTTCTACGATGCTTTGGGGGCTGCCATTGCTACCTTCTTTGGCTTTGCTTTTTCTTTGTACGTTGACAAATATATCCGCATTCCTTTTGTGACAGCTTTTGCCGGTGCCTTTGTCTTCGGACTGCTGGCTCATATCTGGACGCGCTATTCCGGTTTTAACTCCACAGATGATTTGATTATTGCGGGCTCTGTCATGCCTTTTGTGCCCGGTATTGCTCTGACCAACTCTGTGCGTGACATCATGACCAACCATATTAACTCTGGGATGAGCAAGCTCTTTGAATCGCTCCTCATCACTCTTGCTCTCGGTGCAGGTACCTCTGTCGCCCTCCTTATTATGAAATAAGCCTATGACAATCTTGAATTTCTTACTGCAAGCCGTTGCCAGCCTGCTGGCCATCATTACCTTTTTAATCGTTCTAAACGTCCAGCGTAGTATGCTGATTCCCGGCGGAGTGCTGGGCATGGCTATTTGGCTGCTCTATCTCCTGCTCAAAGGACCGACCAACGTCATCATAGCAACCTTTGTGGCTGCTATCCTTGGTTCCTGCATCAGCCAAATCCTCAGCATCATCTACAAAACGCCTGCTGTTGTTTTTATTTTGGCTATTCTAGCCCCCTTGGTCCCAGGCTATATTTCCTATCGGACGACTGCCTTCTTTGTCACTGGTGACTATAGCCAGGCCATGATTCACGCCACTCTGGTAGTCATTCTAGCCTTGGTCATCTCAATTGGCATGGCCAGCGGCACCGTCGTACTCAAGCTCTATCACTACTTAAA

>c183_g39

GGCGGAATCTATATATACAACAAACTAACCAAACCTAACTTTAGCCCCAAAACAACCAAGCTCTATCAGCGAGGCTTTCGACTCTTAGAAGAACAATATGGAACCTATTTCAAAGAACACTATAAAGGTATTGAGAAGATTAAATTTTCTCCGATTTATATTGAAGGGGATAATGGTGGTTCTATGCTAAATGCCTATGTACGTCCGACGATTTATGATAAATATGGGAATAAAGCAACATTAGGAACAACAATAGAGAACTACACACCTAATAGCTATGGTTTAGTTACTCATATATTTCTTGATTTTGATGGTGCAGGAAATGATGTTATAGAATTAATGGACTCACACGGTAATGACATTGATGTTTCAAATGCA

>c183_g4

CAAGATCCGCGTGAACAACAGCTAGCTTATCTTAAAAAGCATGAAGATGAGTTGGCTAACTTTGTTAAAGCTTTGAATCCTAAGGTTGAAAGTGTTCAGTTTAATTGGGACAGTATGAAAGTCGAAGACATTGGGAATGGCACCCCTCAAGGTGGAGGATATATTCTGACTTTGGATGGTGGAATAAATAATAATAAAGACACTGAATTTACATTGGGAATTCCTTTAAACCACAATTCAAATGAAGTTCCTGATAAGCTAGTTATTTATGAGATGCAGCCGATAAGAATTTTACGAGATGGAGGTTGGTTCTTATATGAGTAACAGTAATTTAAAAAACTTTGATAATTTCTACGCAAATTTAGCACAGTCAGCTTACAATGGACGTCCCAATAACTTTCCACCCAAAAACAACTCAAAAAAATTCGCAGAATTTAATTTTTCCAACGATGGTTATATGAGAGATAAGGATGGGAAGATAACAGAAATCACCCCTGGAGGTAAGAATCTCGATAATGATGGCAAAGTTTACCTGCAGCCAGATCCAGATTTGCATGTTGAAAAGGGATTAGATTTACCCTTTGTGGATGATAATGATAAGAAAATTGCTGATTATAAAGAGAAAGCCGGTTTAGGACCTTACCAGAAAGGTCGTTTAACTAATGAGAGAGTTGGCTTTAATGCATATTTTCTAACAGATACACCGACTTTGGGCAAGGATACAAAGCATACTTATATGGCTATTCGAGGCAGTGATGGTTTTAATGCTGAGAGAGTTAAGAAAGAAGGAATTAAGCCTTTAAATCTGAATGACTGGCTTGTTAATGATGCTAATTTTGCTTTATTTGATTCGCATATACCTCAGGCTAGACTAGCAACGGAGGGAATGAAAGCCACAATTGCAGAAATGAGCGAGAAAGCTCCCCAAGCTACGATGGATCTTACAGCCCACTCGCTGGGGACGATGGTGACAGTTCAAGGAATAGCTAATCTGAGCCAACAAGAATTTGATAAAA

>c183_g40

CTGGCTTTTCCTTGCGGTTCATAGCAAAGAGCATACTGCCTGTGAGATAGTCATCGTTTGAGTCAGGAATTAGCATAATAGCAATGTTTTTATCTTTCTCAGGAAAGACGATAGAAAATGTTCCGCCATTATCTTTCCGAGTCAGGCCGTAATAACTCACTCCATTCTTAAAACCAACTGCATTTTGAGTATACTCATACTCTTCTTCGTTGACAATAATGGTCTTGTCTTTTATTTCAATGGTCTTTTGTTCACCGCTAGCATCCTGTACATTCCATTTTCCCTGAATTTTGGGACTGCCACTGCAGCCAATTAGGATGAAGAGGGATGAGAGCACTACTAACAGCAAAGCTTTTATCTTCTTGTTTTTCAAGTTGTCTTCTCCTTGGTATATAGATTCATGACCA

>c183_g41

TCTGCTTCAGTCAGCGTTTCTGAGCCACTTGAGTAAAGCGAACTGCTCAGTTGGTTACCACGAATTGTGCTCAGTCGGTACTTGCCGTCAATTTTTTTGAACAAAAGACTAGCTGTTTGTTGTCCACTCGATTCACTATAAAAAAGATAAAGTGTATCGTCGCTACCGTTGTTACTCTCTCCTCCTGAAGTTGCCTTCCCAAACCGTTCGATAATTTCTTCGGCAGTTGGAGCGTCTTGTCCACTTCCTTGCTGTTCAGATGAATAGACTTGAAGAGCTAAGATATCATCAAATGTCCAAGCGAAGTTAGCTGCTTTCTTATCAACATAAGCTGTCGCTTCTTGAGAAGTCTCATCCTCAGGAACC

>c183_g42

AGCATTTTCTTCTGGAGCAAAGCGCATGCCCTCGATTTCTCCCTTGCGGTAAACGGAAACAACGGATTGGTAAGTCAGAGGGATATAGATAGCTTCATCATGGAGCATGGTGAGGACTTTCTTGTAGCCTTCATCGACTTTTGCCTCGTCTGGTTCGACCAGAGTCGCCTTGATAATTTTATCAATTTCTGGCTTAGATGGCAGAGCTTCTAGGGAAACATTTTCTGGGTGGCCATGGTCAGCTGGTGAAGTCAGGGCAGTCATCCAAGCATGTGGATCCCACGGTGCTCCCCAAGAATACGTCAACATCAGGTCAAAGTTTCCTGTCTTGGCATTTTCCCAGTAGTCATCTTCTTCCATAGCTTTAAGCTGAACGTCAATTCCGATTTTCTTCCATTCGCCTTGGAAGTACTCAACCAAGTCCTTGTCTGTCGCCTTAGAAGAGATATAAGGAACATTCAGGCTTAATTTCTTGCCATCTTTTTCGCGGATACCGTCAGAACCTTTCTTCCAGCCTGCTTGATCCAGCATCTTTTCAGCCTGCTTGATGTCGTAGTCATAGGGAGTCAGATTGGCGTCAGAGTGCGGAGTAGACTTAGAGAAGATGGTATCAGCTGGTGTTTCTGTTCCGCGGAAAATGTCTTTCGCAATAGACTTCTTATCAACCGCATGGTTCATAGCCTGACGGACAGTCTTGTCTTTGAAGATTTCTTGCTTGGCATTCAAGAGCATGAGGCGGCTGGACATTGGCTGAGATACGTCTGTCGTATACTTGTCATCCTTGGCATACTTGGCAAAGTTATCCAGACCGATAACACCATTTCCATAGATTAAGTCAACATTACCAGACTCAAACTCAAGAGCACGGGTTTGTGGGTCTGGAATAATCTTGACTGTTACTTCTTTCAGTTTAGGCTTTTCACCCCAGTAATTTTCGTTACGGGTAAAGGTGATGTACTCGTTTTGCTTCTTGTCTTTGACAACCCATTGACCGGTTCCGATTGGCTTTTTGAGGTTGTCCTTGGTCGTGTCGTCTCCGTCAGGGAAGGCAGCATCAGCTATGAAGCGGATTGGACGAATCATTGATAAATCATAGAGGGTCGCACTATAGGCTTGTTTTAGCTTAATCTCAAAGGTATGCTCATCTACAACTCGATAGCTTTCCAGCTGGTTGGTGAAGTCAAACCAAGTATGGTTTTTCTTATTTTCTTCTGAAAAGACGGTATCGAAATTCCGTTTGACATTTTCTGCATTAAAGTCAGAGTCGTCTGAGAACTTTGCCTTTCTCAGCTTGAAGGTATAGGTTTTACCATCTTCGCTGATATCCCAGCTTTCAGCCAGGGCTGGCTCTATTTTCCCATTGTCACCGTAGCGGACTAGGCCTTCATAGACCATATCCTGAATGACAAATTGGTCTGGATTGTAGCGGTGAGGATTGACATCCCCAAAATCCTCTCCCCAGACCATAGTGAGTTTGTCTTTCTCATTGGCTTGT

>c183_g43

CAGATTTGAAGGTCATATTAGCCGATCGACTGCCGGTCTCCGGCCTGTCCAAACGCTCCATCTTTTCCAGCTGCTTACGACGGGACTGAGCCCGCTTGGTAGTGGAAGCCCGAACTAGATTGCGATTGACAAAGTCCTCCAACGCAGCGATTTCTTTCTGCTGCTTTTCATAGTTCTTGGCTTCAGTAGCCAGCTTCTGCTCCTTTTGGACCACAAAGCTAGAGTAATTGCCCACATAGCGATCCAAGGAATGCTTGGTCAAGTCCAGCGTGATGGTCGCAACCTTGTCTAGGAAATAGCGGTCGTGGCTCACTGTAATAACTGGACCGGCAAAGCCTTGCAGAAAGTTTTCCAAGACCGTCAGAGTTGCAATATCCAAATCATTAGTCGGCTCGTCAAGAAGCAAGACATTGGGCTTTTCCAAGAGAAGCTTGAGCAGATAAAGACGCTTTTTCTCTCCGCCAGAAAGCTTTTCGATTAAAGTCCCATGAGTAGAACGAGGAAAAAGAAACTGCTCCAAGAGTTCTGCAATGGAAGTCGTTCCACTACCTATCTTGACCTCCTCAGCCACTTCTTGCAGGTAATTGATAACCCGCTTAGACTCATCCAAGCCCTCAATCTGCTGAGAGAAATAGGCCACTCGGACTGTCTCTCCAATAATAAGCTGACCAGCCTGAGGCTGAAGTTTACCGGCAATCAGATTGAGCAAGGTTGACTTGCCGACACCGTTATCTCCAACGATACCTATGCGGTCCTTGTTCTGGATTAAAAGACTAAATTGAGAAAGAATAGGCTTCTGATCATAAGCAAAGTCCACATTTTGAAACTCAATGACTTTCTTACCGATACGGCTGGTTTCAAAGTTCATTTCCAGATTACTGTCAATCGTCTGGTCTGCCAAATCTTTCTTTAGATCATGGAAACGATTGATACGAGCCTGTTGCTTGGTTACCCGAGCCTGAGGCTGCCGTCGCATCCAGGACAACTCCTGCTTGTATAACTGCTGCTTCTTATGAAGCAAGGCCGCATCCCGTTCATCTTGCTCCGCCTTAAGACGAACATAGTCTTGATAATTTCCTTGGTATTCAATCAAGCAACCGCCATCCAGCTCAAAAATTCGTGTCGAAATATTATCCAAGAAATAGCGGTCGTGTGTGATGAAAAGCACGGTTTTCTTGGAATTTTTCAAGAAATGGGTCAGCCATTCAATGGTATCAATATCTAGATGGTTGGTCGGCTCGTCCAGCAAGAGCAAATCGGCATCACCTAAGAGAACCTGTGCCAGCTGCACTCGGCGGCGAAGACCTCCTGATAACTGACCAACCTTAAGTGACAGATTAGTCAGACCAAGCTTGGACAGGACCGTCTTGACCTGACTTTCGATTTCCCAAGCATTCAGCGAGTCCATCTCGGCCATAACCTCTTCTAGTCTAGACTGTTGGCTTTCATCATAAGCCGTCAGGAGCAGCTCATACTCTCGAATCAACTGCATTTCCCGTAAATCACTTGATAGAACAGTGTCAAGCACCGTCTGCTCTTCATCGAAATCAGGCTCCTGAGTCAGATAAGTAATTTTATAGCCCGTCTTAGCAGAAAAAGGGCTGACATCTCCGTCAAAGCCCGACTTGCCTGACAAGACATCCAGCAGCGTTGTCTTTCCTGTTCCATTAACACCAATCAGACCAATCCGGTCTAGCTCATGAATGATAAAGGAAATATCCCGAAAAACAGTCTTATCTCCTACTGATTTGGTTAGCTTGTCAACGATAAAATCGCTCATTTCTTCTCCTCGATATAAGCATAAACGGCCTCTAAGTCATTAGGTAAGCAGCCGTCTACAATAGCGTGTTCCAACTCTTTCAGAATTTGACCCAGTTTTGGTCCAGGCTCAAAGCCGAACTCCTGCATCAGCATACCACCATTAACCACCATTTCCTGTTTGTTATGAATAGTTAGACTAGCATCAAGATTCTGAATCGCAGAGAAATCCACTGGTAAACCTTGCGCCTGCCGCAGTTCTTCTGCTTGTAGCAATAAGTCAATATCATAGTCATAGCAGTCGCGCTTGGTCAAGTCTCGTTCTGAGCGGATGGCCGCAATCTCAACCAAGTCTTCTGCTTTCTTGGCAAATTCACGAGAAGTCTTCCATTTTTTCAGAAAACCTTTGACATTTTGCACATCTAAAGCCAGTAAAAGGGCTGCCCAAGCCTGTTCAGATGCTGAAAAGCGAAAATCTGAAGCCAAGTCAAAGAGCCGTTCTAACTTAGCTCTGCTTCCCTTCAAATCAGGCAAAAATTCTATGGCACCGCTTGTCAAGAGTGCCTCTAAACCCTTGCGCCAAAACGGAGCCAGCAAGAGCTTATCAAACTCGATAAAAATTCGTTCAACCGAAATCTTTTCAAGCAAGGGAGCACAATCCTTCATAGCTGCAAAAGTATCTTGCTCCAAGTCAAAATCCAAGGCGGCCTGAAAACGAAAGCCCCGCATGATACGGAGAGCATCTTCATTAAAGCGCTCTGCAGCAGTTCCAACTGCCCGCAAAATCTGATTTTCCAAGTCATCCAAGCCTTGGAAAAGATCAATAACTTGGCCGTTTTCATCCAATGCTAGAGCATTGATGGTGAAATCGCGCCGCTTGAGATCTTCTTCCAACGAGCGCACAAAAGAAACCTTGCTGGGCCTGCGGTAGTCCACATAGACATCCTCGGTCCGGAAAGTTGTCACTTCATATTCACGGTTATTTTCCAGAACCAAGACAGTTCCATGCTCAATCCCCACATCAACTGTCCGGTCAAAGATACGCTTGGTTTCCTCAGGATAGCTGGAGCTAGCGATATCCACATCGTGAATAGGTCGCTGCAAAAGGGCATCTCGAACAGAGCCCCCAACAAAATAAGCTTCAAAGCCAGCCGCTTTTATCTTCTCTAATACTGGCAAAGCCTCCTGAAATTCAGAAGGCAGAGTTTCTAATCTCATAGTAAATGTTCCAATCCATAGACTAATTCTGAGCGTTTGACAACTTCTTTAATGGCAAGATTAACCCCTGTCATAAAGGAAGCACGGTCATAGGAATCATGTCGCATGGTCAATCCCTCTCCCTGGCTGCCGAAGATTACTTCTTGATGGGCAACCAAACCTGGCAAGCGGACCGAATGAATACGCATGCCATCGAAATCAGCTCCGCGGGCGCCAACTATAGACTCTTCCTCATCAGCAGCTCCCTGCTGCTTGCTTGGTCTGACTTGACTGATGAGCTCGGCTGTCTTGATAGCCGTCCCACTCGGAGCATCCTTTTTCTGATCATGATGCAACTCGATAATTTCTACATTTGCAAAATACTTAGCTGCCTGAGCTGCAAACTGCATTAGTAAAACAGCCCCCAAAGCAAAGTTTGGAGCAATCAATCCACCCAGCTTCTTTTCCCTAGACAGTGTGATTAACTCTTCCAACTGCTCAGGAGTAAAACCTGTAGTTCCGACCACTGGGCAAAATCCCTGTTCTAGGGCGAAGCGAGTATTGTCATAAGCTACTTTTGGGGTTGTAAAATCAACCCAGACATGGGCTTCCAGTCCAGCCAATTCTTCCTTGGCATTGAAGACGGGAACACCAGCCACTTCTTTTTCATCCGTAAAGGGATCCAGCAGTCCAACTAATTCTAGTTCAGAATCTTCTGTTACCATTTTATAGGCTGCTTGGCCCATTTTCCCTTTAAAACCAGCAATAATAACTTTGATACTCATGTTTTCCTCCTTACAGAGACAAGCTCAGATATTCAGACAATCAAATCACAGGAATATAAGCAAGTGCAACGCTCCCCTTGCCTAGATGCGTTCCAATGACACTGCCAAAGGTTGCGATAGGAACTTCACTAGCAATACCGCTGTCTAGCAGATTTTTCTGAAGCTCGGCTGCTTTTTTTGGCGCATTGCCATGGATAACCATAATCTGATAAACACCGCCCGCTGTTCTTTCTTGGACAAGTTCCAGCATACGCTTGGTAGCCTTCTTCTCCGTCCGAATCTTTTCAAAAACTTCTATCACACCATGATTGTTAAAATACAGAATAGGCTTGATGCTGAGCAGATTTCCCAGAATAGCTGCGCCGTTAGACAGGCGTCCTCCTTTGACCAAGTGGTCCAAGTCGTCCACCATGATAAAGGCGCTGGTGCCATCAATCTGCTTCTGGACATTAGCTAAAATTGTTTCAAAATCTAGTCCTTCTTCTGCCCACTCTAGAGCAGATTGAACCATAATACCGAGCGGTGCACTGGTAATCTTAGAATCTGGAAAGGCAATTTGCAAACCATGAAATTCATCTTTCAGATACTGAATGTTTTGATAAAAACCAGAAATTCCGCTAGATAGAAAGAGTCCCAGAACATGGGTATAGTCTTTGCCGTCTAGAATGGTAAGAATCTCTTCTAGGTCCGCAATGCTAGGCTGACTGGTCTTAGGCAATTCATCTGACTGAGCCATTTTTTCATAAAATTCACTAGCTGTTAAGTTTCTTCCTTCGACATAAGACTCACCGTCAATCACGACCGGAATATCCAAGACGAAAAGATTTTCGCTCTCTAGCAGCGAAGCCGGCAGATAGGCCGATGAATCTGTGATAACCGCTAACTTCATGATTAAAACTCCAAATTAATGCCTGGCAAATCTAGAGCAATTTCTGTCACTTCATAAGTCAAGCGATTGAGCATGGTTAAGCAAGGCCGAGCCAGCTCTTCTACTTCATCGTGGTCAAATTCACTCGGTTCCTCAATAAGACGACCGTGAATGTGGTTAGCTTGCGAAATAGTTCCGCTGATAACAAAATTCTCAAAAACAATCATAAAAGTCAAAATCACAATCAATGAAGTTGTATGGGCTTCGACATCTCTTTTGACCAATTGGAAATTCACATCAACCTTGGTTTCAGGTGTTCCATTTTCTTTTTCCCACTCAAAATTTCGAGCGTCAAAATGGTACTGACTAA

>c183_g44

CTAGCCAAACAACTAAAAAATAAGTATGATGAGGAGGAAAAGATAATGTTAGGAAAACAATTGAAGTTTATACGGGAGCAAAGAGGATATAGCCAAGCTCAGATTGCAGAATCATTAGGAACGACTCGGCAGACCATCTCGAACTGGGAAAATGATAAGACGATTCTAGATAGTGCAAGTCTTATTCGTCTAGCCGATTTTTACCAGATTTCATTAGATGAGCTATGTGGTCGAAAAGCTTTATCTATCTCCAAAGATTCGAGCATAAAATCAATGATTTTGACAAATGCCTGTACACTTTGGACTTGCTTTGTTTCATCTCTCGGATAGCTCTATTGATTTTCCTGCTAACTTTTTAGTTATTTAGAATATTAGGAGGAAAACAATGTTTTACTATTTTTATTTATCTC

>c183_g45

CCAATGATGACCAGAGACTGACCCGCCTCCACTTCAAAGCTGATGTCCTGCAGTATGGTCTTTCCCTCAATCTGGGCTGTCAGTTGCTTTACTGCTATTTTTTCCATATCTGTTTCCAGCCTTTCTCTTCAAAGCATTCAGCAAATAGATTAAAGGCGACCACAGTCAGAAAGATAGCCAGTCCGGGCGCCAGCATCATCCAAGTTGCCGTTTGAAAATGACTTCTAGCATCATGCAGCATCATGCCCCACTCGGTAATGTTGGGCTGGACACCGATTCCCAGAAAGGAAAAGCCAGAAATCATGAGAATGATATTCCCGATATTCATCAGCACAATAATCAAAATGGGCTTGTAGATAAAGAGCAGGATATGAGTTTTGAGAATATGAATCTGGGAGAGTCCCATGGTCTGGGCAGAAATGACATAGGCTTCCTCTTTGGCACTCTTGACCAGATTGGCCGTCACCCGAGCATAATAGACCCACTCTATGATGACAATAGCTAAAATCATATTGGTCATTCCCTGTCCCAATATCCCAACAGTAGCTAGCGATAGCAGGAAGCTAGGAAAGGCAGAAATAACATTTGCAAACCATAAGAAGGTGGCTTCAGCCTTTCCCTGATACCATCCAATCAGCAAACCGACTGTCACACCA

>c183_g46

GTTCTCTTCTTATCAGGTCCATCAGCTGGTTACGGTCTAAGATCCATTGAGCCCGCTCGTCTTTTTCATAGGTCCGGTTGGATAGGAAAATAGCCGCCTTTTGCTCCTTACGATTATACATGATAAAGGTTCCTGTGTAGCCAGTGTGATCCAGCCAGCTGCCCTCCAGATTCCAAGCTAGACTGCGTCTTTTGCCAGGCTCTTTGGAAAAATTCTGGGTCAGATTGGCCGCAAAGTCATCCTGCAGATAATGCTCTAGAAATATTTCCAAATCCCTGAGGGTTGAAAATAAGCCAGCACTACCAGAATGGATGCCTAGAACACGCGCCTTGGGATCATGAACCTGACCATCCTTGACACCGCGAACGGTCGGCACAGCTCCTGGAACTGGCCCAAAACATGTCTCAGTCAAACCCCAAGGCTGGAAAATCTGACTTTGAAAAATCTGATCCAAGGCCTGACCGAAGATCTCTTCCAGCATAAAACCCAAGAGCAGAAAATTCACATCCGTATAGCGAAAGGTCTTATCTTCAAGCACTGTCAAATGATTGAGTGCTTCTTTCAACTTGGGAGCTGTCAGCTGTTCACGATTAGGAATAAAGGGATCTAGTCCTGATGTGTGGGTCAAGAGCTGACGCAAGGTCACATCTTCTTGATGAAAGGCCGGATAATAATGCTGTAAGGGCAAATCCAGTTCCAGCTTGCCCTGCTCATACAAAAAGGCTGCCAGAGTACCAACTCCGACCACCTTGCTGACGCTAGCCAGGTCATAGACCAAACCCGCTTGAGTGGCCTTTCCTTCTTGCGGATCAGCTAGACCAAAGTAGAATTCCTGCCACTGGCCAGCTTGATACAAGGCCAGACTAGCACCGGGATAGATACCGTCTTTTAGCTGTTCTTTGATTTTACTGATGATGTTTTCTAGAGTCATGCTTCAAACCACAATTCAATCTTACTAAAATCCTGACTCAGCAGGAACTTATCTGACTTAGGTACAAAGACCTCGTGTCCTTCAAAGATAGGACGCAGCGCAGCGGTTTCTAAACGATTGACCTGAGCTTTGAGCATAGTCAAGTCCCAAGTTAGGGCATTGTCTGCCTGCAAGTCTTGTCCCTCTGCTTCTGTAAAGGTCAGGAAATCCAGCGCATTTTCAATCTTGCTATAAAATTCTTGAGCTTCATTGGCATTAGGTACACGAATTTCTACCGTCTCTATCTCAAATTGACTAAGACCGATGAAGTCCTCTTGCTTTTCAAATTCAGGCGCCTCCGCCACTTCTTGAAGATTTGTTCTATTCTCTTCTGCATGCAGGAGAACTAGGTCACCCTCTGGTGACAGAGCTTCAAAAGCATAGCCTTTCTCTCCTTGATACAGCTTAGTCCAAGCAGGCTTTTGGGCCAATAGAGACTCGATTTCTTTAGCATCTGCAACCTTGACAACGATTCTGGCTAATTTTTTAGGACCTTTTACTCGCCGCGACCGCATACTGGGTGATTCTTCCAACTGCAGTTTCTCAGTCTTAGTCTGATCTCCTAAAGACAGAAAAGCAGCTTCCTCTAAAAGGGCCTTCATGCCCAGCTGATTTACAAAAAATTCTTGATTTAAATGACGATTATTGATCTTTAATACAGGGATAATTCTAATAATATGATTCGCGCTCATAATTCCTCCAAACCATTTTATTGTAATGGATTTTCATCTTTTTGACAAGAAATTATGCGCAAATAAACAATTTTTAAAACAAGAAGGCCTTGGATTTATTGGTAAACGGCCTGAGCAATGACCAAACCCCTAGCGGACTCAAACTCGTACTGCAGATAGGACTGGTAGGTACCAGGTGCGATAATACCACGAATATCCAGAATATCTGTCCCAGCCTGCCCAATAATCGAATCTGCTAGCAGGCAACAGTTGGTCGACAGGACAAAATAGGTCTTGAAACGACTGGTTTTAAACTTATATAGCTGCGCTCCTAGTTCATGCTTCAACTTGTAGGAGTATGTTGGCTGGCCATTTTTCAGTTCTGCTGGCGGCTCCCATTCTACCAGCAGCTGATCAATCTCAGCCAGGCGCTTCTCAACCGCCTCTTTCTCCTTATCTGTCAAAGCCAGAGAATAACCAAAGAGTGTTTTCTTGCTTTCTTTTTTGCAAAGCTCAATATAGACATCTTTAGGAACCTTGAAAAGAACACCATCTCCAATCATGCCCTTGCAT

>c183_g47

GGAAAAAGGACCTGAAAAACAGTCGCTGTTACCATCATCAAGAGCACAATGACAAAGGATAATTTGTAACGCTTAAAATATTGCCAGAAAAATGCAATCGTCCGCATCTTAGTCCTCCTTTCCTTTCTGAGTTTCGTAGATTTCACGGTAAACATCGTTAGTTGCGACCAATTCCGTATGCGTCCCTTGGCCAATCAGCCGACCCTGATCCAAGACCAGAATCTTATCTGCATGAACAACGGAGCTAATCTTTTGAGCAATGATAATTGTCGTCGTTCCTTGCAGCTCTTTATTCAAGGCTTCTTGAACCAACTTTTCTGACTTGGCATCCAAGGCTGAGGTCGAATCATCTAAAATCAAAATATTGGGATTGTTGACAACACCTCTGGCAATGGACATCCGCTGCTTCTGACCGCCAGAGAAGTTGCTGCCACGCTCCTCCACTGCACTGTCAAAACTCTCTTCCATACGGCTGATAAATTCACTGGCTTGGGCAATACGAGCCGCCCGCTCCATCTCAGGCAGTGAAGCATTGAGCTTGCCCTGACGGAGATTATCCGCAATGGTCCCGCTAAATAGAATCGCCCGCTGGAGCACGATGGAAACATTTTTACGAAGAGTTCCCTGACTAAGCTCTCGCAGATCCCGGCCGCCAATCTTGACAGAACCTTCCTGCGGGTCAAAAAGACGGGGAATCAGCTGGGCCAAGGTAGACTTACCAGCGCCCGTCGCACCGACAACTCCAATCATTTGCCCCGGCTCAACCTCAAAGCTGATATCCTTTAGCATTGGCTCGTCATCGTTGGGATAAGAAAAAGTCACATGCTCAAATCTGAGGCTGCCTTCTAGTTCTTCATCTGGCGAGGCTGGGAAAGTCATAGCTGGCTCCGTCGCCAATACTTCCTTGATCCGACGGATAGAAATGATCCCCCTCGTCACACCATTTCCCAAGAAGCCAACCATAATGATAGTGAAAATAATCTGGCTCAGGTAACCGATAAAAGAAGCGATGGAGCTGACCAGTCCTGGCTCCGCCTGAATCATGCCAGACAGAGTCCAGATAGCCAAGTAAACAGAACTGTAGCCGACCAACATCATCATCGGCTCAATGATTGAAAA

>c183_g48

TTTAAAACATTTGCTTACATTTCAACCCCCTTTTGTGGTACAATAATGCTAGTTTATTTTAAAAAAGATTGAGGAAATTTATGTCTGAACTGTATGATATTACGATTGTTGGTGGTGGTCCAGTTGGCCTATTCGCAGCTTTTTACGCTCATTTGCGCCAAGCCAAGGTTAAAATCATTGATTCCCTGCCTCAGCTGGGTGGTCAGCCGGCCATTCTCTATCCAGAGAAGAAGATTCTGGATGTGCCGGGCTTTACCAATCTAAGTGGCGAAGAGTTGACACAGCGTCTGATTGAGCAGCTGGAAACTTTCCAGACTGAGATTTGCCTCAATGAAACAGTGCTGGATATCGTTAAATCTGATGATGGCTTCACCATCACGACTTCTCAAGCCCAGCATCAGACCAAAACCATTATCATCGCCATGGGAGGCGGCGCTTTCAAACCGAGAGCTTTGGAGCTAGACGCTGCTGAAAGCTACAGCAACCTTCACTATCACGTTTCAAACATCAGCCAGTACGCTGGCAAAAAGGTCGTTGTTCTTGGCGGTGGCGACTCGGCTGTTGACTGGGCGCTAGCTTTTGAAAAGATTGCAGAAACCAGTCTGGTTCATCGTCGGGACAACTTCCGGGCTTTAGAGCATAGTGTGGAAGAACTCAAGGCTTCTAGTGTTGAGATTAAGACACCATTTGTACCGAGTCGATTGGTCGGCGAAAACGGCAAGATAACCCACTTGGAAATCAGCCAAGTCAAGGGGGAGGAAAGTCAGCTTCTGCCTCTAGATCATCTCTTTGTCAACTACGGTTTTAAATCCTCTGTCGGCAATCTCAAAGATTGGGGCTTGGAGCTCAACCGTCACAAAATTTTGGTCAATAGCAAGCAAGAAACTTCCGTGCCAGGTATCTATGCAGCTGGTGACTGCTGCAGCTACGAGGGAAAGATTGACTTGATTGCGACTGGGTTAGGTGAGGCACCTACTGCTGTCAACAATGCCATTAACCATATCTATCCTGATCAA

>c183_g49

CATCATCCTATATCAATGTTATCGGTGCTGGTCTTGCGGGCAGTGAAGCAGCTTACCAGATTGCCAAGCGTGGAATCCCAGTCAAACTCTATGAAATGCGGGGTGTTAAGTCAACTCCTCAACACAAAACTGCAGACTTTGCAGAACTGGTCTGCTCTAACTCTCTGCGAGGAGATGCGCTGACCAATGCAGTTGGTCTGCTCAAGGAAGAAATGCGGCGGTTGGACTCGGTCATCTTAAAGTCAGCAGAAGCGACTCGGGTGCCAGCAGGTGGTGCTCTAGCGGTTGATAGAGAAGGTTTTTCTCAGATGGTAACAGAGCTAGTGACCAATCATCCACTGATCGAAGTCATTCGCGAAGAAATCACTGAAATTCCTGAAGATGCCATCACAGTCATTGCGACAGGGCCCTTGACCAGTGATGCTTTAGCAGAAAAAATCCACGCGCTCAATGGAGGAGACGGTTTTTATTTCTACGACGCAGCAGCGCCAATCATCGATGTCAATACCATTGATATGACCAAGGTCTATCTCAAATCCCGCTATGATAAGGGGGAAGCAGCCTACCTTAATGCTCCCATGACCAAGCAAGAATTTATGGATTTCCATGATGCTTTGGTCAATGCCGAAGAAGCACCGCTTAATTCCTTTGAAAAAGAAAAATACTTTGAAGGCTGTATGCCTATTGAAGTAATGGCCAAGCGAGGCATTAAAACCATGCTTTATGGTCCGATGAAGCCGGTAGGTTTAGAATATCCAGATGACTACCAAGGGCCTCGAGATGGTGAATATAAGACACCATACGCTGTGGTGCAGCTTCGTCAAGACAATGCGGCAGGCAGTCTTTACAATATCGTTGGCTTCCAAACTCATCTCAAGTGGGGCGAGCAAAAGCGAGTGTTCCAGATGATTCCTGGCCTTGAGAATGCAGAATTTGTCCGCTATGGAGTTATGCATCGAAACTCCTATATGGACTCTCCAAATCTTCTAGAACAGACTTTCCGCTCCAAGAAACTGCCAAATCTTTTCTTTGCAGGTCAAATGACTGGAGTTGAAGGCTATGTCGAGTCAGCAGCTTCAGGCTTAGTTGCTGGTATTAACGCTGCTCGACTCTTCAAGGGAGAAGAAGCACTTGTCTTTCCAGAAACAACAGCTATCGGCAGCCTGCCTCATTATGTTACCCATGCTGACAGCAAGCATTTCCAACCTATGAATGTCAATTTTGGCATTATCAAAGAGCTGGACGGCCCTCGGATTCGTGACAAGAAAGAGCGTTATGAGA

>c183_g5

ATTGCGCTCCACTGCCACCACTTCCAGTTCTGGATAGGTTCGGGCTGCCTGAATCGTGACACTGCCAGTGCCAGAACCGACATCCAGCATGCGCTTGGCTCGATGCAGCTCCAACTTATCCAAACTGATGGCTCTGACTTCTTCCTTAGTCATAGGGACTTTAGCCCTGATAAATTCCGAATCCCTCATCTAACACCACCACCACATTCATTTCATATTCTCTATCTTCTACCTCACTCGCAGGCAGAATGGTAATACGCTCGTCTTCATAACTCAGCTGCTCACCAATGACCAGCACCTTATCTAAGCCTCTTTTAACAGCCTCTTGAGCAATTTCGTAGGGGCCAACTTTCTGATCGGTCACCATAAAGACTTTTGACAAACTCATAATCAAGTCAAAGTTAGGCACCTTAGCATGACTGCTCGTCAGGTAAGCATCGTTCATAGGCAGGGCAATTCTGCTGGCCAGATACTGCATGGCGCTGATACCTGGCGAGATGACCACGCGCCCCTTGAACTTCTCTGACAGCCATTTGCCCAAGCCGTAAATCAGCGGGTCACCTGAGGCCAGATATAAAATTTCTTCATCCTCAACAAAGCTTTCCAGCAATTCTGCTAACGCAGCCAATTTTTTAGGAGGGACCAGGCCCTTGTCCCGATAAGCCGGCGGTAAAATTTCCAGATGGCGTTCACTGCCCAAAATGCGGTCAGCCTTTTCAAAATAGGCTTCCTGCCCCAGAAGACCATAGCTCGGATCTCCCGGACCAATTCCGATAACATAAATCATGTCCATTCCTCCTTTATCTGCTCTAAAGGCTGAGTTGATGCCAGATAGCCTTCCTCAGTCCCAAAGACGACCACTTCAATATTGACTTCTGGCTTACGGTAGCGAAGCAGACGCTCACTTCTAGCCTTAATCTTATCCGCCAATACCTGATAGACACCCTCATAAGAAAATTCTTTAATGGCTGTCCCAGCTGCCTCGGTCGTCAAGCACTTATCAACCTTTTTCAGCAACTCCATGGGCGCCCCCATCAGGGCTAGATTAGCCACCAGCGTTTCCATACGGGCATCCGAGTCCTTACTGTGGGTAGAAAAAATCCCAGCCGCCACTTTGACAAATTTGCCCATGTGTCCTATCATCAAAACCTTGGTAAAGCCGATGCGCTGCACTTCTTTGAGGACGTGTCCGACAAAGTTACTCATATTGACAATGCGCCCAGCTGGAATCCCCACAGTATTAGTAGCAAAATCTTCACCGTAATTCCCTGGCACCAAGACGACTGAGCGGTGCCCTTGATTGTAGAGCATGGTCAGCTCGATTGTGATAGCTGCTTTCCAGCTTTCCTCAGACATAGGATTGACAATCCCTGTCGTCCCCAAGATTGAAATACCGCCGATAATACCTAGTCTAGAATTGTAGGTCAACTTGGCTGTTTCTTCTCCTCCGGGAACGGAAATGATGACCCGCGCCCCACAGTTGGGACCGATAATCTTACGGACATGCTGCTCAATCATCCGTCTAGGAGTCGGATTGATGGCTGCCATGCCAACATCGCAGGCCAGTCCTTTCTCGGTCACACGCCCGACGCCCTGTCCACCGTCAATCTCGATTTCCGTCTGATCTGGCAGCAGCGTCACAGTCGAATAAATCAAGAGACCGTGCGTCGCATCCGCATCATCTCCTCCGTCTTTTTCAATGGCTGCGGTCGCCTCTTCTGGTCCAAAGGAAGGATTGTGAACATCCATGGTCACTTCCACACCTGAAGCCGTATGCACAGTAACCTTTTCTTCCCTTTCCTGATTTAAAATCATGCTCAAGGCTGCAACAGTCGCTGCCGTTGCGCAAGTACCAGTTGTAAAACCTTTTCTTAATTTCTTTCCATTGACATAAGCGTATTCATCCATCATCAGCCCTCTCCTTTCTCCGGAGCCTGCTTTTTATCTGAAACACTGTAACGCTCATAGTCCGTAGTAGCAAGCTTGAGCTGGTAGAGAATGGCATTGACCACAGCTGCTGCGATGGTACTGCCTCCCTTTTGACCCAGTGCCGCGATAGAAGGCACCGAACTTTCATGCAGTTTGAGCTTGGATTCTGCCGCTCCGACAAAGCCGACTGGAACCCCGACAACTGCATCCGGCTGCAAGCTTCCCTCTTCGACCATATCCAAAACCTTGAAGATAGACGTTGGAGCATTCCCGAAGACAAACAGTTTTGGACCATCCACTTTAGCTGCATATTCTACAGCAGCCATGGAGCGAGTAATTCCTTTTTCCTTAGCTTCCTGAAAGACTGCTGGCTCATTGACCAGACAGCGATAAGAAACACCTAGATCATCCAGCAATTTCTTATTAAAACCACCCAAGACCATGGTCGTATCCGTAAAAATCGTGCCCTTGTTTCGGAGGACGTAGAGGATTTTCTCAATAACCTGATTGGAAAACTTGATATTGTAAAGGTAGTCAAAGTCTGCACTGGTAAAGACAACCCGCTTGAGCACTGCCTCTTCAAAAGGATCCGAAAAATGAATATCCGGATGCTCTTGGTCAATAATTTCCTGAATCTTGCTGAAACTAGCTTCTTCAATCTTGTGCGCTGCTTTTAGATAGGTCATCTAGTTCTTTCCTCCTTCTTTACTGGATGGTGTAGAGTAGCGGACATAGTCATCCGTCACCACTTCTCTCAGCTGGTAGATAATGGCATTGACAATGGCTACTACAATCGTGCTGCCGCCTTTTCTGCCGAGATTGACAAGGGCAGGAAGACCTGACTGAAGCAGTTCTTCTTTGGATTCTTCGACATTGATAAAGCCGACTGGAACCCCAATCACTGCATCCGCTTCTACCAGTCCCTGCTCTGCCAGCTCAATCAGATAAGACAGAGCCGTAGGAGC

>c183_g50

ATAAAGCATAGCATCAGCCAACTGGTCAGCGTCTCCGCCCAGCTGACGAACCAGCTCATAGGCAAAAGCCAATGCAGTTGATGGACCACGGCTAGTAATCAGCTTCCCATCAACAAGAACTGTTTCTTTACGGTAAGTTCCATTTTTAATATTGTCTTGCGCACCATCATAGCAAGTGAAATACTTACCAGTCAAGATACCTGCGCGGTCAAGAGCAATGGGAGCGGCACAAATGGCAGCGATGAATTTATCCGACTGCTGAAAGTCCTGCAAAACCTGCATCAGGCTGTCATCATCACGAAGATTGGCCGATCCTGGCATCCCACCTGGCAAAATGACCATATCGTAGTCATCCAGTCGCCCCTTCCAGACCTGATCCGCTTTAACAGTGATGGCGTGAGAGCCAGTCACTGATTCATCAAAACCAATCATATGACAGACAAGACCTGCACGGCGCAAGACATCCACAACTGTCAAGGCCTCGATTTCTTCAAAGGCGGGAGCTAAGAGTAAAGCTGCTTTTTTCATAAAAATATCCTTTCTGAGATGAGTCAGTCACAAAATCCCATCTGATTTGTGAGTCAAAAACTTGCTTTATATTTATTTTAATTTCTTCAGTACAACCTTTTTTCGGACAGCAGGAATTCGCTCCTTCTTTTCATCCGTCAGGCTGTTCTGATAGGAGACGGACAGTAGGAGACCAACACCGATAAGATTACTGATGATGGAGGAGCCTCCCTGTGAAATAAAGGGCAGCGGAATCCCTGTCAGAGGCAGAATTCCTGTCACCGCTCCAATATTTTCAAAGATATGGAAGAGCAGCATCATGATAAAGCCAGTTGAGATATAAGTATAAAACTGGTTGTTGGATTTAATCGTAATCTTGAGCATGCGATAAATCAGCAGTAAATAGAGCATGATAACCAAGGTTGAACCTAAGAAACCAAAATCTTCTGCAATAACGGTGAAAATCATATCACTTTCCCGAACAGGCACCAAGAGATTGGAAACATTAAAGCCTTGGCCAGTCAGACCACCGCTTCCGACAGCGATTTGTCCCTGTGCTTGCTGGAAGGTGGTCGTCTGAGCGTAGTCAAAGGGATGAAGCCAAGCCAAAATCCGATTGATTTGGTAGGTTGGCATGCCTAGATTATGCAGAAAGGCACGACCGCCATCAGAAATAAAGATAAAGAGAAATCCTCCCAACAGCAGGACTCCCGTCAGAAAGACCGGCAGGATAATCTTCCAAGAAACACCTGACAGTAGAACGATGCCGCCGTATATAGCAACAAAAACCAGAGCCGTCCCCAAATCACTCTGAAGGGTTAACAGAACCAGAACAGGCACTGTGTAGAGCCCAAGCTTCAGAATCAGAAAAAAGTCTAAAGCCAAGGTTCGCTCATCCTGCTTGTGCTGCTGGAGAAAATGAACCACCAGCCGCGACAGCATGAGAATATATGAAATCTTCATGAACTCCGAGGGCTGAAAGAGGGTCACACCACGAATAGCAATCCAGTTTTTGGCACCAGTGGAGGCTACCAGAGATTCACTGTAAAAAATCAGAGGCAAGACCATGAGGCCAAGACCAAAAACATAGAGATAAGGCGTAATTTTCCAGAGAAACTTGGTGTTAAAAAACATGAGGATAAAGCTGAGCAAGAAGCCCACCGCAATCCAGGCAATCTGCTGACCAACCATGGGCCAGGCATTGTCAGGATAATCATGACTAACAGCAATGTATATAGCCACAACTCCGATTGAAAGCAGCATCAAAACGGGCAAAATTAAACTATAATCAATCCGAGACTCAAACGTCCGTCTTTGATAGGGCATAGTCCCTCCTTTTTAGAAAAAATACTGGATAATCAAGCTGGAAACAGCGACGCAAAACCAAGCGAAAGCACCAGTCAGCAAAGGCGCTGCTCCTGCTTGCTTAAACTGCTTAAAGGAAACCTTTGCGCCAATAGCTGCCAAGGCCATAGCCATCAGCCACTGAGAAATGAATTTTGTATAGGGGATGACAGAGGCCGGCAGAAATCCAAGACTGCTGATAAGTGAAGCTAGGACAAACCAAGCTATAAACCAAGGAAAAATCTGTTTCAAGTTTGTCTTTTGTGATGACTGCTTAGACTTAATATAGCGATAAGCTGCGAAGATCAAGCAGGCCGGTACAATCATCAGAGCCCGACTAAGCTTGACAATAGTCGCCAAGTCCCCAGCTGACGGGCTATATGTATAGCCTGCTGCCACTACTGACGAAGTATCATTGATGGCTGTTCCAGCCCAAGTCCCGAAAAAGGTATCCGACATCTGCAGCAAATGCCCTAAAAAAGGGAAAATGAATACTGCTAAAATATTGAAAAAGAAAATAGTGGAGATGGACAGGGCAATTTCCTCTTCATCAGCGTCTAAGATTGGTGAAGCAGCCGCAATGGCAGAACCTCCGCAGATGGCTGTTCCAAAACCGATTAAAATAGTCAAGACACGGTTCATCTTAAAGAAACGGCCAGCCAGATAAGCCGCCAGAAAGGCTATCAGAATGGTGATAAGACTGATACGAAGAGAAGAAATCCCTGTCTCAGAAACCTGACCAATGGACATGGAAAATCCCAAAAAGATGATGGAATACTGCAACAATTTCTTCCCCGAATAGCTGAGTCCTTCCTGAAAAACAGCCGGCAGCTTCATGCTGTTGTTCAAAACAATCCCAAAGACAATAGCCAAAACACTAGAGCCTATCAAGGGAAGCAAGCCGCCTAAAAAGATAGAAACAGCCGC

>c183_g51

CTATTTTTACCTTGAAATGAAAACACATGAGTTAGTCGTTCCTTATACCAAACAGAAACGTCGTGTTCGCGTCTTGCTTCCAAAAAACTATAGTCAGGACACAAATAAGACGTATCCAGTCGTTTATTTTCACGACGGCCAAAACGTTCTCTATAGCAAGGAGTCTTTCAGCGGCCATTCGTGGAAGGTTATTCCGACAATCAAGCGTAATCCAGATATTGAAAAAATGATTGTCGTGGCTATTGACAACGATGGCCAACGGCGGATGAATGAATATGCAGCTTGGAAGTTCCAAGAGTCCAATATTCCCGGCATTCAGTTTGGCGGCAAAGGAACGGAGTATGCGGAGTTTGTCATGGAAGTTGTCAAGCCTTTCATTGACCAGCATTATCGGACCAAGTCGGATCGGCTGCATACGGCTATGATTGGCTCCTCTCTTGGGGGAAATATCAGCCAGTTTATCGGCGTTGAATACCAAGATCAGATTGGCTGTCTGGGGATCTTTTCATCAGCTAATTGGCTGCATCAGGAAGCCTTTGACCGTTATATTGAGAGAAAAAAACTTCAGGCTGACCAGCGAGTTTTCATTTATGTTGGGACGGAAGAGGCTGATGATACAGACAAGACATTGATGGCTGGTAACATCAAGCAGGCCTATATTGATTCATCGCTCAGCTATTTCCGACAGTTACTTGTATCTGGGGTAAATTTGGCAAATATTCAGATTAAGATCCAGTCTGGAGCTATTCACAATGAAATCGCTTGGGCGGAGCACTTGCCAGACTGTTTTCGTTTTATCAGCGAAAAATGGTAAGCTAGTCAAACAGTGAGAAAGAGGTAGAAACTATGCATGTAGAATTTTTAAGTCATTGGAGCGGCAATCTAGGCCGTGAAATGTATATCAATCGCTATGGCCATGCTGGACTCCCGATTATAGTCTTCGCATCATCAGGCGGTAGCCATAATGAATATGCTGACTTCGGTATGATTGAGGCCTGCGCAGGTTTTATTGAGGCTGGTAAGGTCCAATTTTTCACTCTCAGCAGCGTTGATAGCGAGAGCTGGTTGGCGGACTGGAAGCACCCACACGACCGAGCAGAAATGCACCGTGCCTATGAGCGTTATGTCATTGAAGAAGCGATTCCATTTGTCAAACATAAGACGGGCTGGTTTGACCCTATGATGACGACAGGCTGCTCCATGGGTGCTTATCATGCCGTTAATTTCTTCCTGCAGCATCCGGATGTTTTCAATAAAGTGATTGCCCTCAGTGGTGTCTACGATGCACGTTTCTTTGTTGGTGACAATTTGAACGATGAAGTCATTTATCAAAACTCGCCAGCTGACTACATCTGGAATCAAAATGATGGCTGGTTTATTGACCGCTATCGACAGGCCGATATTATCGTTTGTACTGGCTTGGGCGACTGGGAGCAGGATGGCCTTCCTTCCTTCTACACGCTGAAAGAAGCTTTTGAACATAAGAATATTCCAGCTTGGTTTGCTGAATGGGGCCATGATGTTTCCCATGACTGGATTTGGTGGCGTAAGCAAATGCCTTATTTCCTTAATGAACTCAATCTTTAAAGGAGGTTTCCTATGAATTATATTGTTATTTCTCCCTACTATCCGCAAAATTTTCAGCAGTTTACCGTGGAGCTGGCTAACAAAGGAATCACTGTTCTGGGAATCGGTCAGGAGCCTTATGACCAGCTAGATCAGCCTTTGAAGGATTCATTGACCGAATATTTCAGAGTAGAAAATCTTGAAAATCTTGACGAAGTAAAAAGAGCTGTTGCCTTTCTTTTTTACAAACACGGGCCTATAGATCGGATTGAGTCTCATAACGAATACTGGCTTGAACTGGACGCTGAGTTGCGTGAGCAATTTAATGTCTTTGGAGCAAAACCAAAAGACTTGAAAAAGACCAAGTTCAAATCAGAGATGAAGAAACTCTTTAAAAAAGCAGGTGTTCCAGTCGTTCCAGGTCAGATTGTCAACACAGAAGCTGGGGCAGATTTGGCTGTCAAGAAACTTGGTCTGCCTTTGATTGCCAAACCTGATAATGGAGTAGGAGCAGCTGCGACCTTTAAGCTGGAAACAGCAGAAGATGTCGAACGATTCAAGCAAGAATGGGATCATCAGACAGCTTATTTCTTTGAAAAGTTTGTCCAATCTGGCGAGATCTGTACCTTTGATGGTTTGGTAGATAAGGATGGTCAGATCGTCTTTGCAACCACCTTTGACTATGCTCATACACCGCTGGATTTGATGATCTATAAGATGGACAATTCCTACTACGTTCTTAAGGATATGGATCCAAAACTGCGTGCTTATGGAGAAGCGATTGTCAAAACCTTCGGCATGAAAGAACGCTTTTTCCATATTGAGTTCTTCCGCGAAGGTGATGATTATGTAGCCATTGAGTACAATAACCGTCCAGCTGGCGGCTTTACCATTGATGTCTATAATTTCGCCCACTCAATCGATCTTTACAGAGGCTATGCAGCGATTGTAGCAGGAGAGCCTTTCCCAGCTGCTCACATGGAGCCACTGTATTGTTTGGCAACCTCGCGCCGTGCATCGACAAACTATGCTTACCCTGAGGCTGACTTGCTGGAGAAATACAGAGATAATTTCAAAGTTAAGAAAGACATGCCAGCCGCTTTTGCAGAGCTGCAAGGTGACTACCTCTATATGCTAACAACACCAAGCCGTGAGCAGATGGAGCAGATGATTGCAGATTTTGCTAAACAGGCGGACTAATCAAAAGCATGCTCTGCTATTATTTTCGTATAACAGTTTCAAAATAGTAAAAACTACTCTA

>c183_g52

GTCATCATGGTCATCATCATCGTCCGAATCATCAGTGTCTGTCGTATGAGAGGAACTGTTCTGAGACGGAGTTTGAACTGTCGCTGTATTAGCCCCTGTAGAGCCCGCTGCTCCCGTATTTCCTGTACCTGCAGTAGAGCTGTTATCAGCTGGAGTTGATGCAGCAGACGAGGATGGACTGACTTCTTTTTCGACCGTTACTGTCTTTTCGACAATGGTATGCTGCTTGGATTGGATTAGTTTTCCATCCTTAGCATTGACAATGGTTTCATGAGCTGTTTGCCCGTGCGAGAAGCGGATTTTATAGATGTCTTTCTCATGCGAAATTTGTAAATCTTTAATATCTTTTTCAGAAAGAGACAGCTCTTTTAAGACGGACGCTAGAGCCTGACGCTGACTGAGTCCTTGAGACTGTTTTTGTCCTGCCACAGGTCTGCTTTGTTGAGCAGCTGGACTTTCTAGAGCAAAGACGGAACTAGTCAAGAGCAAAAGGAGAGGAACGGCTACTACAAAGACAGCAGATAGAAACCTCTTAGGCAGGCGGTTATCCATAATACCGACAATCCGCCGCTTGAGATTAAATTTGTCAGAGTAAAAACAAGTCGTCAGAGCAATCGGGGTCTTCTTACTGCGGTCTATCATGGTCAAAATCGTTTCACCGTAGAAAGTCCGATACTCTGTATCTCTACAGCTCAGAACATCGTAGTCACAGTACATTTCTCCTGCTTCCTGAGTTTCACGGCAAGCAAAGCGGACAACCGGGTTAAACCAGTGCAGACTCTTGGCAAAAATTCCCAAAAGATTAACTAAAACATCGCGGTGCTTATAGTGAGTCAGCTCATGCTTGAAAATCAGCTGCAGCTCTTCTTCTGTGTAGTCCAACTCTGGCAGCACGATTAAAATATCTCTGAAACCTAGCAGCATAGGACTTTGGGACATAGGATAGTGGAGCAGACGAATCCGGCCCTTAACTCCCATCTCCTGCTGAACAGCCCGCAGCTGAGCCATCGCTTCTTCGTCCTGAAACTCCGTGCCCCAGCGCCTCAGCATCTTTCTGAAGCGAATATAAGAATAAGCATATCTTCCAATACTAAAGACAAAGCCAATCAGCCAGATGGCAAAGAGAATTTCAAACCAAGGTAGGCCCAGAAAAGCCTCCCATAGATTTGTCTGGGCAGCATTTTCAACTGCTTGAGAAGCACCCTGAGCTCCACCTGTCTGGGTGGCAGTCACTGCTATCTGAACTACCGATCCTGTATTCAGCCGAATCAGGCCTGAGCCAAATTGGGGACGGAACGGAAACAGAAAACTCAGCAATATCAGAAACCAAATAAAATACTTCACTCTGACCGAAATCTTAGTCTTAAAGGCCGTAAAAAGCAGACTAAGCAAAAGCACCAAAATGGATGTCGTCAAACTGGTCAGAAGAAAAGAAAGAAGGAACTGTTTCATACTTATCCCTCCATCCTACTTTCCTTGGTTGAGCAGACTGCGCAGCTCATCCAATTCATTTTCCGAAAAGGAGTTAGAAGAAAAGAGTGTTTTGACAAAACCGCCCATAGAACGACCACTGTGACGCTTCAAGAAATCCGAAGCCTCAACTTCCAGATACTCATCTTCTGAGATCAAGGCTGTATACTGCCTTTCACGCCCCTTGCGGACACTTTCTAAAAAGCCCTTTTCTGTTAAGCGAGCCAAGACTGTCAGCAAAGTCTGAGGCTTCCAGTGATTGTCGGGTCCTAACTTTTCCATGATACGAGCAGAGGTCGTCGGGGCAGGTAGCTGCCAAATTACCTTTAAAATAGTAAATTCCCCGTCTGGCAAACGTTTAATAGATCTTTTCATCGTTCCACGCGCTTTCTAAATTAATGCTATTTCTATTTTTATTCTACACTTGTCTAACAAAAAAGTCAATCATTATATAAAAAAGAGCGAGAAATCAATTTTCCTCACTCTATTTAGAAAAATAAACTAAGCAGGGTCAGCATCGCCAGCCCTCCTTGCTTAAGCAGGATTTTGGGGTCACTGGTCACTGCACCGTAAGCTGCTACCAAGATGATATAAACCATAAAAATTCCCAGCCAAACCGGACTAGGCTGCACAAAGGCCGCAATCAGCACTAAAACAGCTATCAATCCGTTATAGACTCCTTGGTTTTTGAAAAGAGTATTGACCGACTGCTGCCCCAACTCTTCTTGCGACATACCGAAAACCCGAGCTGTCGCTGCAGAAGTCGTCGCGAAGGTCTCCAAGTACAGGATATAGAAAAATTCCAAGGCAACCAAGCTTGCCAGAA

>c183_g53

AGCCCATTCACTTCGTCAAAATACAAAGAAACAGTCATATTGTCTGTGGTTGAGTAACTTAGACTAATTGAATCAACTTGCCCCTTAACTGATTTAGAAGCATAAATAGTTTTAGGCATCCCCTGTTCTGCTATGGCCTGTTTTACTTCCACTCCTTCTTGCTTATCTTGAGGAATAAGAGTTCGGTAATCATCGACCGATTTCGTAACTGGCCCCCAAGCATTACGGAATAGTCTAACGTATACTAATGAGAGCTCTGTTCTACCCTCAACATCCCTAAACCTTAAGGTAATATCTCCTTTCTCTCCTATTAATTTATATGTCAGTTCAACTAATTGGCTGCCTTTCACGCTATTATCTATGCTACCGGAACTAGCTTTTCCATACTTCTCGACAACATCCTGTGGAAGCACATTAGGTTCTTTCATCGTTTTTACCATCTGTAGTATATCTTCTATTTGGAAGTCTATTTGTCGATTCTCTTCTATAAGGTCAGAATAATCTTCAGTCAGCTTCTTTATGTTATCTTCTGATTCATTCTGAACTGTACTAGTAGACTGCGCTGATGACTGATTTCGTAATTGCTTGGATCTTGGCCACATATATGATACAAGTATTGCAAGACAAGTAATTAGCACTAGACCTGTAGAAACTACTAACAAAGAGGCGTATCGAGACTGACCTAAACTTGATATAAGTTTAGGTTTTCGACTACCTGCCAACTTTTTTTGCTGTTTTTCTTTAGCTGTAGCCTCAAAAACTTCCCCCTGAGGACTGCTATCAGAGAACACACTAGTTGCTTGCTTGTTGAATCCTGGC

>c183_g54

TTGCAACACCATCAGACGCTGCGACCTTGCTTGCTATTTACGCGCCTTATGTAGAGAATACAGCCATCACTTTTGAGTATGAAGTTCCAACTATCGAAGATTTCACAAATAGGATTGCGAAAACATTGGAAAAATATCCTTACCTGGTGGCGGAAGAAGACGGTGTAGTCGTAGGTTATGCTTATGCTTCGACTTATTATGCGCGTGCCGCCTATGATTGGGCGGCGGAATTGTCCGTCTATGTCAGTCAAGATGCTCGAGGAAAGGGAGTCGGCAGTAAGCTTTATGATGAACTTGAAGACCTGTTAGATCAGATGGGATATATGCATTTTCTAGCCTGTATTTCTCTGCCTAACGAAGCTAGCCTGGCTTTGCATCGAAAAAGAGGCTATCAGCAGGTGGCGCATTTCCCTAAAATGGGCTATAAATTCGAGCGTTGGCATGACATTGTTTGGCTGCAAAAGTCCTTGGACAAGCAAGCTGGCCCCATCAAACTTTTAAAAGAAATGGAGTGGAAATGATGAAAAAGAAACATTTATTAATAACTCTTCTAAGCATTGCCCTTTTGACTTTGAGCGGCTGTCAGGCCGTTGAAAATTGGTTCAAAAATGCCAAGGAGGAATGGTTGGGGTTGGAGATGACGGTTCGCACCTATGATGAAAATTCTCAGCTTATTG

>c183_g55

ATTTAGTTTTCCATTAGAATAAGTAACAGGAACACTCTCTTTATTGTCCCCAATTGTTATTGTATAGTCACCTTTAATGTCAGATTGGGTAGTATCTAAGTATATTTCATATTCCTTAACTTCGCCTCCGTCTTTAATATAGACACTACTTGTATAAAGCTTTAATCCAGTAAAATCCTTTTTTTCTTCTTCCAAAAATTTAAACATAGGATCAAATGTTTTCTCAAATTCGTACTGTTCTTTTGTTCTATTTTTGGGGATAGTGTAATCATCCAAACGCCAGCCATTTATCAATGTTATTGATAAAACGGCTACACCTCCAAGAATAATGAAAGTAATATATTTTTTAATCTTTTTCATTTCTT

>c183_g56

GAAGAGTTTTTCTCACTCAGATACTTCATGCTCTCTGAGCGGATTTTGATGATTTGATAAGGATAAGCTATTTGCGATTAAAACAACAATTAATAAGCAAAAAGGCCAAGACACTTGTCTCGACCTTCATTATTATTCAACGATAAATTGACTCAAAATCCCATTAATGAACTTACTGGACTTTTCGTCAGAGAATTGCTTGGAAAGCTCAATGGCTTCGTTGACTGCTACCAGCTGTGGGGTATCAAACTCTGTAATCTCAAAAATACCCAGGCGCAGGATATTTTTTTCGACTAGCGTCAGACGATCCACTGTCCAGCCCTTTTTGAGGTGCTGGGCAATTTTCTTATCCAAGTCGTCCTTGGACTGGACCACACCGGAGACCAGATTGAGCAAGAAGGCTGGAATATCAGCTTCCGCTGCTTTGTCAGCCCCTTCGTCCTTATCATAAGAATAGGCAAAGCGACAAGCTTCTACAAGATCCCCTTCATACTCTAGGCTCATCAGGGCCTGAAAAGCCCGCTGGCGCAGACCTCTTCTGGATTCCAACAGTATATCAGTCATTGAGGAAGTCCTCATCAAATAGATCTTTCAAGTCTGGCTTCGGTGCCTTTTCTGGAACAATGCCTGCCACATGGATATTGACCGCAGACAGCTCTACCTCAGCCATATCAAAAACAGCGCTTTTTACAGCCTTCTGGATTGCTACTGCAACAGTTGGAACGCTGACACCGTATTCCAAATACAGGTAGATGTCTACAGTCACATCACCTGTCTCATCTGTATGGAGCGATACTCCACGACCGAGTGAACGCATAGAAAGACTGTCTGACATACTCTTATTTGCGAAGGAATAAACACCTTCTACCTTTGCCGTTGCAATGGCAATGATTTTTTCCAAAACACGTGGCGCAATGACGATTTCACCTAATTGTTCAGCTGCCATAGTTATTACCTCTTCTATATTATGCACGAGAAACGTAAGTTCCTTCTGCAGTGTTGATGATTAATTTTTGTCCAACTTCGATGAAGTCTGGTACGTTGACAACAAGACCTGTCTCAAGTGTTGCTGGCTTACCAGATCCGGTAACAGTAGCTCCCTTGATAGATGGCTGAGTATCTGTCACGACCAATTCAACTGTTGTTGGTACAGTCACACCAATCACTTCTGTTCCGTAGAACTGGATTTTCACATCAGAGTTTTCAAGGATAAATTTCAATTCTTCTTCTACATTGACTACTGGAATTTCATATTGATCGTAAGTCTCAGTATTCATGAAATAAGCAGTGTCATCCATTTGGTACAAGTATTGAGCTGGAACAGTTTCAATAATGGCTTGTTCAAATTTTTCTTCTGGGCGGTAGCTTGTGTCAAATGTAGAACCAGTACGAACGTCACGCAATTTCATACGCATAATCGTATTTCCCTTACCTGGTTTGTGGTGGCTCGCTTCCAAAACGCGGATGAGTTTTCCATCAGCTGTTTCAAAGGTCATTCCAGCTTTCAGCTTACTTGCTTCAATCATTCTATATTACCTCTTTTTAAAATTATATTTCTACTTATTCTATCATAAAGCCTGATAATTCTCAAATAACAATCAACTCTTTCGGCGCAAGAGTTAGGACTTCGCAGCCTGTTTCTGTGATGAGGAGATCGTCTTCAATACGGACGCCGTATTTGCCGTCCAGATAAATACCCGGCTCATCAGTCAGGACCATGCCGGCTTCAATCGGCTCTTCTGACTTGCCAAAGTAAGGAATTTCATGTATATCCAGGCCGATACCGTGGCCAATCCCATGGCTAAAGTAAGGACCGTAGCCAGCATCGTTAATAATCTGGCGCGGAATCCGGTCAAAATCAATCCGGCTAAGACCAGCCTTAGCTGCTTCTATCAGGGCTTGATTGCTGCGCAGGACAATATCATAGATTTCCCGCTCTTCATCTGTCACTTGCCCCACATGAACTGTCCGCGTCATATCACTAACATAGTGATTGTAGTAGCAGCCAAAGTCCATGGTCAGGGTTTCCCCCTTTTGAATGACCTTGTCACTAGCCACACCATGCGGCATAGCAGAGCGATAGCCCGAAGCGATGATGAAGTCAAAAGAGGCACCTGAAGCACCTAGCTGGCGCATACGGGCATCTAAAAAGTTCATAACAGCCAGCTCTGTCGTCTCACCAGGCTTGATAAAGTCCAGTACATCTAGGAAGGCTTGGTCCGAAATCTGACAGGCCTTGCGAATAGTCGCGATTTCGTGCTCATCTTTAATCATGCGCAGATTTTCAATGAAGGCTGTCATGGGAACCAGTTCGTAGGCCGAGAAGACACTTTCCAGCATTTTGAAGTAGGCATAGGAAATCTCATCCTCAAAACCGATTTTTTGCAGCTTGTCATCCGCAATAATCTTGACAATCTCTCTAATCGCGTCGCGCGTTTCGACAATATCAAAATCTTGGACAACTCCCTTGGCAATCAGCGTATAGCGCGCATCTGTCAGGAAAATTCGACGAGTCTTACTGATAAAAACTGTCGCTTCTGTCCCGCTGAAACCAGTCAGATAGTAAATATTTTTCAGATTGGTTACTAAAACAGCGTCGCATTCTGTCTGAGCCAATGC

>c183_g57

GCTGATTATACAGCTGCAAAACCAACCATCTACTCAAAGACAAACTGGTTGTGATAAAGTTCTGAATAGAAACCGCCGAGTTTAAGGAGCTGATGATGATCGCCCTGTTCGATAACCTCTCCGTCTTTGAGGACGATAATCTGGTCGGCATTGAGAATTGTCTTGAGGCGGTGAGCAATGACAAAGCTAGTTCGGCCAGCCACAACGGCTTCCATGGCTTGCTGAATCTTGCTCTCCGTCACCGTATCGACATTGGAAGTCGCTTCGTCCAAAATCAAGACTTGCGGATCAGTCAGCAAAGTTCTAGCGATGGAAATCAGCTGTTTCTGCCCAGTTGAGAAAATATTCTGGTCATCATCTACCAGAGTATCGTATTTGTCTGGCAGACTTTCGATATAGTCATGGATATGAGTCGCACGCGCAGCCGTTTCTACCATCTCCTGACTGGCATCCGGCACACCGAAGCGGATATTGTCCCGAATCGTACCACTAAAGAGCACCGAATCCTGCAAGACAATGCCCACATGGCTCCGTAAGCTATCCAGCTCATAGTCCCGAATGTCGCGACCGTCAAACTCAATGCTGCCACTATCCACATCATAAAAACGATTGAGCAGGTTCATAATGGTCGTCTTACCTGAACCAGTCGGTCCAACAACAGCCGTCATCTTGCCCTTAGGGGCTAAAATAGATACGTCTTTGAGAATAGGCTTGCCTTCTACATAAGAGAAGTCCACATGCTTGATCTCCACGGAGTCTGTAAGCTCTATGAAAGCAGGTGCATTCTCTGGTCGGACTTCCTCAGGCGCATCAAACATCTCCTGAATCCGGTCAGCCCCTGTAAAGGCCAACTGCAGACTGCCCCAGCTGGCTGCAATCTGGATGATGGGCTGATAATACTGCTGGGAAAACTGAGTAAACGTGGTAATCAGGCCAACTGCAACA

>c183_g58

GCACGACTCCGATAACCGGCACCTGGGTCAGCTCCTCAATCATATCAATACCGGACTGCAGCAGGGCCACATCGCCACGGAATTTATTGATAATAACACCCTTGATCCGCTTGCGGTCCTCAGGCGGCATGAGCTCAATCGTTCCGTAGATAGAGGCAAAGACGCCTCCCTTATCAATATCCGCCACTAAAATCACTGGCGCATCAACCAGCTTGGCCATGCCCATATTGACAATGTCTCGGTCATTGAGATTGATTTCAGCTGGACTGCCAGCGCCCTCAATGACGATGATATCATTCTCAGCACCTAGCTCATCATAGACTTCCTTAATCTTAGGCAGGAGCTGCTGCTTGTATTCATGGTATTCGACAGCATCCATATCCCGCAGTACCCGGCCTAGAAAAACAACCTGGGACTTGCGGTCAGAGGTGGGCTTGAGCAGGACTGGATTCATGCGGACATCCGGCTCTTTGCCAGCTGCCTCAGCCTGAACGACCTGAGCCCGGCCCATTTCATCTCCTTTTTTGGTGATAAAGGAATTAAGCGCCATATTCTGGGACTTAAAAGGCACCACTTCTAGGCCATCCTGCTTAAAAATCCGGCAGAGCCCCGCCGCAATAATGCTCTTCCCAGCATCTGAGGCAGTCCCCTGTACCATCAATGCTTTGACCATGTTACATTTCCTCCTAGCTGCTCAAAGAAAGCAGCTTCATTTTCTGCCAGTGGCGTCTGGATAGTCTGATGAAGCTTGACAATCCAAGGCACAGCCAAGCCTGCCTCTACTAGCAGATTTCCCTGCTGGAAGAAATCAAACTTGCTGCCCTCCGCAATCAAATGCCCTTTCTGCAGCAGGTAGGCATAGTCGCAGCAGTCATACATCAGATCCATGTCATGGCTGGATACGATGATGTTTGTCCCAGCCTGAACCAGCTTTTTCATGGTCTCGGCCATCTGATCCCGCCCTTTGGGATCCAATCCTGCCGTAGGCTCATCAAGCAGCAGATACTTGGGCTGCAGGGCCAGCATGCCAGCAATAGCCACCCGCTTTTTCTGGCCATAGCTCAGATACTGCAGCGGTCTGTCCTTCAGATGAGAAATGTCCATCATCTCTAAAGCCGTCGCCACTCTGCTGGTTATCTCGTCTTCTTCATAGCCTAAGTTTTCCAAGGCCATGCCAATATCGTCCTTCACAATGGTATAAAAGAGCTGCTGCTCTGGATTTTGAAAGACCATATTGACATGCTGCCGATACTGGAACAAGGCCTTCTTAGAGTCGCCTACAGGCTGACCATCATATAAAACACTGCCCTTCTGCGGTTGGAGCAGTCGGGTGATGATTTTCATGATGGTAGACTTACCTGAACCGTTGACTCCCAGAATGCCAGTAATCCGGCCCTCTTCGAAGTCCATAGAAATATCGTGCAGCGTTGGATTTTCGTCATACGAAAAAGAAATATTCTTTACTTGTAACATCTATGTTTCCTATCTACTTTCCATTATAGTATAAAAGCAGTCTTATTGTAAATGCGCTTTCATTATTCACTGTGCTCTGCATCAAATCTTAGCGTCAGTACTTCATTTAAATGCTGATTATCATCTAGCAATTTAACAAAAAGAGTATTGGCTAATCTGCCCCAAGCTTGGTGTTGCTTCCTTTTATTGTAAAAGGAGAATTTCAAGTCCAAGGTATCCCGAATGGTCACAAACTCATAGAAGACCAGGAAAATAAAGCGATACATGAGGACAATCAAGTCTAGCAGTACCCGTGGAACATGCATAGCTTTAAAAAGCCGCAGCATCTGAGCAAAAGGCACCGTCAGGACAAAGAAATAAGTCGAAACCAAGGATGAATAGATGCGCAAGAGGATAAAAATGGTCTGCTGGACAGAGGATTGGCTGATGCCCAGATAGCCCTGACCCAAAGGCAAGGCCAGCAAGAGCTGCTCCTGACTATTCTGATAGGTCAAGACAAAGGTCAAGAGACTAATCAGGATAAAGATACTGGCATGCAGATACCATTTCAGATAGCGCAGCCACGGAAGCCGAGCCACATAGCAAGTCAGAGGCGCCATCAAGACGATAAGTCCTAGCTGCAGACTCCTGACACCAGAAAAGCTGACAGCTAACAAGAGCAGATAGATGACAAATTTATAAGCAACCGGCAGATTTTTCAGCCGATTTTGATAGGCATATTTATCAATCGCAAACAAGTGGAGGTACTCCTTTCTAGCTTTGTTTTTACCACGCCCCCAGTACAGAGCAGCTCCGGTCCCAAACAAGCTGAATCGCTATCAAGAACCAGAGCTCTCTCATACTGTTGAAGGCTTTTATTTATGTTCAGAGTTTTCCTTTTGTTGGCCTTGCTTTTTCCCTTTGTGGTAGCCAATGACATAGAAGATGATACCCGCTCCGATACTGCCTTGCAGGGTGAAGAGCAGACTTTCTACTTCCGGACCTGCTGGCTCAAAAATTGGTGAAAACCAAGGCTCATAGTCCTTTTGGATAGAGCTAATGGTTTTTTCAGCAGCGTCATCTGTTCCACTGTATTCCACACCCTTAGGTGCAAAGATAAAGGCAGCTAGAGTGACTGCTACTGCAGCCAAGATTAAGATGATATTCTTGTATTTTTTCATTTGAACAGACCTGCTCTTTCTTTGACATTTTCTGAGATTAAGTTATAGAGGACAACTGTCAGCAATCCTTCTACGATAGCGATTGGGATTTGAGTAGTCAAGAAGACACCCATGAATTTCAGTGCAGAACCAACAAAGCCACTGTTAGCATCAGGGAAGACCAAACCGAGCTGGATAGAAGTTGTCGCATAAGTCGCCAAGTCCGCAATCACAGCACAGATAAAGATTGAAACCGGTGTAGAAAGCTTGATGGATTTAGCAAACTTGTAAACGAAATAACCAACAAATGGACCAACCACTGCCATTGAGAATGCATTGGCACCCAGAGTTGTCAAGCCGCCGTGAGCCAAGAGAAGGGCTTGGAAGAGCAAGCAAATGGTTCCCAAAACGCTGATAACGGACGGGCCAAACATAGCTGTCCCCAGACCAACCCCAGTCGGATGCGAACTAGATCCTGTAACAGACGGAATCTTCAAAGAAGACAGGATGAAGATAAAGGCACCGGACAAGGCCAACATAGTCTTGGAATTTGGATCCTCTGCAACGATTTTCTTGATGCGCATCAATCCTACTACAAAGAAAGGCAGGAATACGGCGAACCAGAAGATACACCAAAAGAGTGGAAGATAGCCTTCCATGATATGCATAGCTGACACAGACTGGGTACTCAGTACCGCCAAAATAGCCAGCAAGGCAACAAAAGTTACTTTTTTATTTTTTAATAGTTTCATGTTTTTTTCCTTTTCTAAGAGCCTGAGCTCACAAGAATGAATCTTCATCCTTGAACATAGACTCTGATTTTAAAAAATCTTCTTTTTCACGAGGAAGGTAGTAAAATAAGGCAACTTGGTCTCTGGTGTGATTCCTTCGAGACCCAGCAGAGTCTGCTGCTTATCCGTCGAAGAGTTGCTAACCATAAAAGTCTGCTGCAAGAGACCGAGCTTTTCAAGCAGAGGCAGGACTGTATCCAGGTGATTGGCCACCTTCATGATCACAATCGAGTCATGCAGCTCAAGCGCCGCCTCGATCTTCTCAGCAGAAGCTGTAGCCGGCATGACCGCCAGAGACTCCTCATCCATGATCAGGGGCTGACCTAGCTCAGAAGCCATGCTGCAGAAAGAGGTGATACCAGGGATAGTCTGACAGTCGATTTCCTTTTCCAGCAGGGCCAGCAGGTAACTGTAAGTGCTATAGACCATGGGATCCCCCAGTGTGATAAAACCGACATTCTTGCCAGACCTCACATCCTCTGCGATTTCAGCAGAGATAGCCTGCCACTGGACTTCCTTGGTCGAATTGGAAAGGACCATAGGGAAATGCCGCTGCTTGATTTCCAGATGCTCTTTCAGATAGGGTTCTGCAATGCCTAATGCAAAGCTCTTGCTTCCCTTCTTAGCCTCTGGTGTATAGAGAATATCTAAGTCTTCCAAAAGCCGACTGGCCTTGACCGTGACTAGCTCACTATCACCCGGTCCCACACCAATTCCGTAAAATTTCGCCATTCCTTCCTCCTATTCGATGATTTCTTCTAAATGTTGGATGTAGAGCTGCTGAACTTCAGGATATTCGCCCAGACCAACTAGATGGGCCTTAACCTCATATCCCTGCTCCTTAAAGAAGCGATACCAAGAACCTTCCTCATCTGAGCTCATATCATTGGTCGCATGGTCGCCTGCTACCAGCATGAACGGTGCCAGATGCACTTCTCGGACGCCATCTTTCTTCAGTTCCTGCTCAATCAGCTCAACCGGAGGATAGCTCTCTACACAGCCGACATAGACTGAGCTTCCCTTCAGCATATGATCCAAGGCTGCATAAGCTGTAAAAGCATAATGCTGGCTGCCGTGGCCCATCAGAACTGTTGCTGCATCCTGACCCTCATGGCCATACCGTTCCAGCAGAATATCCTTGACAGCCTCATAGTCTTCCTGACTATTGAGCAGAGGCTTGGCAACGACTAGCTTTTTAAAAGCTGATTCAAACTCTTTGGCTTGGGTAAGAATCTTCTCATACTCGCTGCCCAAAATGACATGGAGAGGCTGGATATAGACCTCTTCAGTACCCGCTTCCAAGAGCTGATCCAAAACCTCTTTGACCGTAGGGATATCCAGCCCTTCCTGCTTCTTAATCCGCCGTCTAACGACATTGGATGTAAAAGCTCGGTAGACCGGATAGGCTGGAAAACGCTCCTGAATAGCCTGCTCACAGGCCTCGATTGTTTTGCGTCTGGTCTCGGGATAGGTCGTTCCGAAACTGACTACTACTATAGCTTTACTCATGATTCACCTCCTTTCAGAGAATTTCCCGACATTTGTCAACGATGGAAGCAATAGTAGCTTCATCCGTCTCAACAATCTTGCTAAAGCCTGCTTCTGCTAAAACAGCCCGCGTGCTAGCACCCATGACAACGATTGGAACATCCTTCCAGTCAAATCCAGCTTCTTGACTAGCTGCTACAAAGTTCATGGCTGCAGCAGAGTTCGGGAGACAGACTACTTCAATCTCTAACCAATTGGTACTGTTAATCTGACTGCCAAAGGCCAGCCTGTGGCTAGCAATAATCGGCATCGAATAGAGTTCTGCCAGACTGGCTTTTTTATGCTCAGCTGTCAGAATATACCAGTTACCGCCTTCTTTTTCAAGAGCAGTTGCAAAATCCGTATCCGACTGCTGTGGCGTCATCCGATCCAGCACAATGCCGCTCGCTTCGATAGATTTAGCCGTATGATGACCAATCGCTGCAAAGCGGATGTGCGGCAGACTGCGCAAGTCTTTGCCTGCCTTGCGGAGGGCTTTCAAGAAGAGCGGCCAGCTCTGCATATCTGCAAAGAGGAGACCGTCTACCTGCTCCAAGTCCGGCAGCTGCAATTCTAACTCTTCAACCAAGTCTCGAGCTGGGAAAGTGGTCAGCGCAGCTCCAGCATCCTTAAGCAAGCGAGGAAGTCTGCCAGTCTCTGACTGTTGGATAAGGATTTTACGACCGAAAAGTGGCAGATTCTCATGGAAATTCAGCTGCTGTCTATAGGCCACGACATCACCTACCACGATGACACTCGGTGCCTTAAAGTCTTCAGCTTCTGCTAGTTCGACTATGTTCCCCAAGGTTCCGTCCACTGAGCGCTGCTGAGGATGGGTTCCCCATTCAACGATGGCGGCTGGCTTGTCCTTGCCATAGCCTCTAGCCGTCAGCTCTTGGACGATGGTAGGCAGATTTTTCATGCCCATGAGGAAGACCAAGGTTCCTTTGAGCTTAGAAATTGCTTCCCAGTTGAGCATTTCTGTCTCATCCTTGAGATGGGCTGTAAAGACATGGAAGCTAGTCGCTACATCCCGATAAGTCATAGGAATTCCTGCATAGGTCAGACCAGCCACAGCAGAAGTGATGCCTGGCACCACTTCAAAGTCCACACCAGCTTCCACCAGCTTGACTCCTTCTTCTCCGCCTCGGCCAAAGATATAGGGATCACCGCTCTTGAGGCGGACTACCCGCTTGCCCTGACGGGCTTTTTCGATGAGAATTTTTTCAATTTCTTCCTGTCGGATGCAGGGCTGGCCTGGCTTTTTACCCACATCTATCTTTTCGCAGTCAGACTTTAGATGCTGAAAGAGCTCTTGATTGACCAAGCGGTCAAAGACCAAGACATCTGCTTCTTGAAGCCGTCTCTTGCCTTTGAGACTCAACAACTCCGCATCGCCTGGACCTGCTCCCAGTAATGTTACTAAACCGGACATTTTTTCTCCTTTTTTTAGCTATTTTGTCAGATAAGCTTCTAGCTCTTCTAAACTAGAAACCATCTGCGGATAGGACACAATTGGCCGAGAGATGATAATGCAGTCCATCCCCAGCTCTTGGCAGCCGTCAATCTTTTCACGAATACCGCCAACAGTACCGCTTTCCTTGGAAACAAAGACGTCTGCTCCCGAGCGCAGCAAGAGCTCTTTATTGCACTCTTTGGAAAATGGAGCCTTGATCGCATCAATCTGATCCGCTACCAGTCCCAGCTGCTCACAAGCCAGAAGCACTTCCGAAGTCGGCAGAACGCGAACAACAATCCGCTGCTCTGGCAGACCATTGACAAAGAGCGGCAAGGTCTTGCTGCCCGTTCCCAGATAGACTGTCTTGTAGCCGCGCTTGGCAATCTCATCAATCGCTTCTTGGGTCGAATGCACCACGATGGCCCCGCTCAAATCCAAGGTCGCCTGACGCTCAAAGCGCAGGTAAGATACACCGGCCATCTCTGCCGCCCGAATGGCTTCCTTGGAAACGATGTCCGCAAAAGGATGGGTCGCATCAATGATTTCATCCACATCATTCTCTTTGATAAAGGCAACCATGTCCTCGGCTGTCAGCCGGCCCTGAATGACCGGCTGGCCGTATTTGGAAGCCAAGTGCCGGCCGTAGTCTGTGACAACAGAACTGGTCACATCAATCTTCAAGCGGTCAAGCACTTCTAAAATAGCGGTGCTGTCAGATGTCCCTCCCAGCAGTAATTTCATCATAAAGTATATCCTCGAGGTGTGATCATGCGTCCGTTTTTAACATAAGTTTCTTTGTTACCAACGATAACGATTGTCGTCATATCTACCAAAGTCTCATCCAAGTCTTTGATAGTCGTCAAGATGATTTCCTCATCCTTACGGCCAATGTCCTTACCGATACCGACAATGGTATCCTCTGACTTGTACTCAGACATGATAGAAAGTGCTTTTGTCAGATGGTCTGGACGTCCCTTACTCCGAGGGTTGTAGAGGCAGACTACAAAGTCACCTTGAGCTGCGGCATGCAGACGCTTCTCAATCATTTCCCAAGGCGTCATCAAGTCACTCAGACTGATATGACAAAAGTCGTTCATGAGGGGAGCTCCCATAACTGCAGCAGCACCCAGACTAGCTGTAATACCTGGAACCACCTTAACTTCTACATCACTGGCATCTCCCAGCAATTCTAAAATCAAGCCAGCCATTCCATAGACCCCGGCATCTCCACTGGAAACAACTCCGAC

>c183_g59

CTCGCCTCCACAAGATTGGAGATTAAAGGAAAAGAAATAATGTTTTTTCTTGATATGCCAATATATCTGATTCCTCTCAAGCTCCTGACAGCTATAACCAGCCTTTCTCTTGGCCGATTCGGACGGCTTCTGTTCGATTTTCAGCATTTAGCTTGGTCAAAATAGCAGACATATAGTTACGAACCGTTCCATTTGACAGGTAGAGTTTTTCAGCAATTTCTTTATTGGATAGGCCAGTCGCCGCAGCTTGCAGGACTAGCCTCTCCTGCTGAGAGAGAGGGTTCCTACTGGTCATTAGCACCTCCATCAATTCCGGCGAATATTCCTTTTGCCCATCTAGAACGGTTTGGATGGTTTTCATCAAATCCGCAATGCTGCGCTCTTTGAGCACATAAGCATCTACATCCGCCTTAACAGCTCGCTCAAAGTAGCCCGGTCGCTTGAAGGTTGTGACGATGATGACCTTGATGTTGGGTCGATTGGCCTTGACCCATTCGAGAACGTCTAGGCCGGTTTGGTGGGGCATTTCCACGTCCAAAATAGCTACATCAACAGTTTCTGAGCTAAGACAGTCAATAGCCTTCTGCCCATCTGCGGCCTGATAGACTTCTTCGACATCTGGCTGCAGCTGCAAGAGCTGAGCTAAGGCATCTCTCAGCATACTTTGATCTTCTGCTAACAAAAGTTTCATGCTTCCTTCCCTCCGTATGGCAATTCAATTCGAATCCATGTTGGATCTTGGCTGCTAAGAATCTCAAGCCTTCCTGACAAGGCAGACAGTCGCTCTCGAATACTATGCAACTCCCGACCAGTCAGCTTTTGAAAACCACAACCATCATCCTGAATGTCTAAAATAAGCCTTTCATCTTTCTTCACCAAAGAGAAATTACTCTTTTTAGCCTTGGCATGCTTGATGATATTGGTCGCTGCCTCTAGTAAGATCATACTAATCGTTGACTGCTGACTCGGTGGGATACTGGCAACATTGAGCTGGTTATCTATCTCGACCTGAACACCGCTCATCTCCAGCATGGCTCGAATAGTCACTAGTTCCTCGTCCAACGTCCGATTCTTTAGATCGTTAATAATTCGCCGAACATCAGCCATGGACTTCTTGCTGATCTCTTGCACTTCCTGCAGTTCTTTTGCTGCCTTGTCGTAAGCTTCCATCTGCAAAAACTGCTGAGCCAGCTCCGCTTTAACACTGAGCATAGCAAAGGTATGCCCTAGACTATCATGTAAGTCTCGGCCAATCCGATTGCGCTCATTTTCTGCTAGAAAGAGATTCAGCTGGGCATTTTGTTTGGTCTTTTCTTCCTTGATTCGCTCGGTCGTTTGAATTCGATGGAGACCAAAAGTCAAACCATCCGAAAAGAGAAAAGTGACAATAAAGAAGAGTAGTTCTGCTGGACCAACATTACCCATCAAATAATTAGTCAGCACAATAGCAGGCTGCAAATCAATAAAGGTCCAAAAACGCCAGGATTTAAACGAGACATCATCCAGCTCATAAATCAGCAGATTAGATAGATAAAAAATATACCAGGTAAATCCTGTATTTAACCAGACCGAACCATAGTATATGTAAGCAACCAAAAAACACCAAGAAAAAAATTGAATAAGGCGATGGTTACTTAGCAAAACCGCATAGTAAGCCACAACAAAAATAGCCGTCCAGAGCAAGAGGAAAAGCGGGTATTCCCCGCTGATTACTCCCGCCACTGGAAAGATGATAAAGACCAGCGCAATATGAAACATATAGTGGACTTGTTTAAGCTTTTCCCACATGCTTTTATTTTACCTCAATTCGCTTTTTGAGCTGCAGCACAAGAATGCTGACCAAAACCGTATAAATCAGAACAATCAAAGCTGCCTTGCCATTGAACTGATGGTGTTCTAGATAAGAAGACACAACCTGCATCAGTTGATAACTTGGCGTCAACTTACCGATTGGCTGGAGCCATTTTGGGAACATGGTCAGAGGGAACCATAGACCGCCCAGAACAGCCAGTGCCATATAGACGATATTGCCCACTACAGACATGAGCTGAGCACTTGGCAAGAGACTGACCAATACACCCATAGCAATAAAGACCACACTTCCTACCAGAAGGATCAAGGCAATAAGAAACCAGTCCACCATTGGCAGATTAACCCCGCGAACAAAATGCCCAATCGAGAAGACTACGATGATAGAAAGCATAAAAGTCAACAGCGTACTGCAAAGCTTTGATATATAATATTTTACCATAGAAATCGGCGAGTGCTGAATCATTTTTTGCCAATTATTATTTTTATCAGACTGCAAAGTGCTTGGAATGCTAAAGAAGGCACTAGACATGATACTAAAAAGAGTCATAGAGAAGAGGTAGCCTCGAAGCAC

>c183_g6

CAAGGATTTCCGAGTCCGGTCACTCCCTTATTTTGTAGGTGGTTTATGAAACAATTTGTAAAAAGAGAGTTTCACTCTCGTTCAAAATTTTTTGCCTGCCTCTTGACCTTTTGTGCAGGATTTATCGATGCCTATACCTTCATTGAGAGGGGAGGCACCTTGGTTGCAGGTCAGACGGGAAATGTCGTTTTCCTTTCGGTTGAACTCATCAATCAAGAGACAAGAGGAATTGAAGTGAAATTGGCAACTATGTTGGCCTTTATGCTGGGTATTTTTCTGATGACCGTTTTTCAGCATCATTTTGAGCATTCTTGGCGCAGGCTGTCCAGCGTTTTTCCCTTGATATTGACGACAACTGTCGCAGGATTTTTGCCAGCAAATATCCCCCATCTCTATATCGTGCCGCCACTAGCTTTCTGCATGGGCCTGGTTGCGACGGCTTTTGGCGAGGTGGCTGGCATTGTCTATAACAACTCCTTTATGACGGGGAATATCAAGAAAACCATGGTAGCTTTTGGCAACTATGCTCGAAACAAGGAAGGAAAAGATTTGAAAGAAGGCCTTTTCTTTGTTGCCCTATTGGCAAGCTTTGTAGTCGGAGCCATCATCTCTACCTATCTGATTCAGTTTTACCTTTT

>c183_g60

TGTGATTGAAGCCACAATCTAAATTCATGCTGCGACAGTTTTTTCTTTAAGACAGCTTGATAGAGCACCTGGGCTAAGCGCCAGTCCTCATCATTTCTGAAACGGACTGGAACTCTTTTAAAAACTTGATGAATGACCTCTAAAACCACTGGCATCCTACTGGAGGGAAAGTCCGGATGACAAGCCACTTCTGTCAATAAGTCTGCTCCATGAGCGAAGGCATGAACCCAACCGTATTTCCTGGAGTAACCCCTTGTGTCTCTTTCTACTGACAAGTAGGTTAGCCCCTTATCTAGTAAATATTTTCTTTCTTGCACTGATAAAGCCTGATAATACGAAGAGTTTGGATTACCATCGCAGTTTAAGAGATTGGCATAAAGTAAAGCTGTGAAAGAACGTGTTAAGGTGGCTTGACCATTTTCGTCACTCTTGTATAAAAGTCCTTGTCTTTTGACTGCTTCTTGAGCTAAGAAACGAAACTGTTCAGCTGAAAACAGATCATCCTGAAGTCCCCTTGCTAAGGACGAAAAGACTAACTCATCACGGATTTTCGCTTCTGGATGACCAATATGATCCAGTAACCATAAGATTTCTTCCTCATAATATAAAGGAGAAGCTT

>c183_g61

CGAAGAATAAACGATGGTCACTCCCTGCTGCTTGAGCTGATTAACAATTTCCCAGAAATGCTGACGAGTGGAAGTGTCCATAGCTGAAGTCGGTTCGTCCAAGAACAGGATCTTAGGACGGCCAATCAGACTTAATACAAAAGAGAAGAGACGTTTTTGCCCACCAGACAGCTTGCTGGCCATCTGATTCTTTTGCTTGTCTGTAAATTGTAATAAATTATCAATTTCTTGATTGGATAAACTATTTGGATAAATTGCTTGGAAGAACGTCAAGAGTTCCTTGACCTTCAAATCCTCCACCACTGTATTTTCCTGTGGTAAAATCGCTACAGACTGCTTGAGATAATTGTCAGTTGGTTTGAGCCCCTGA

>c183_g62

CTTACAATTACATGGACCTCTCACCGGGTAAGAAAGCAGAAGATATTGACCTAGGTTATATCTTTATCGGCTCCTGTACCAATGCCAGGCTCAGTGACTTGCAGCTGGCAGCTAAGTTTGTCGCTGGTAAGCACATTGCTCCCAATCTGACAGCTATCGTCGTACCAGGCTCTCGTCCGGTCAAGCGGGCTGCTGAAAAGATGGGACTGGATAAAATTTTCATGGATGCAGGCTTTGAGTGGCGCGATCCGGGTTGTTCCATGTGCCTGGGGATGAATCCCGACAAGGTGCCAGACGGAGTTCACTGTGCCTCGACCAGTAATCGGAACTTTGAGGATCGGCAGGGATTTGGAGCAAAGACCCATCTTTGCAGTCCTGCTATGGCCGCAGCGGCAGCCATTGCTGGGCGATTTGTAGACGTCCGTCAGCTACCGGAGGTCCAGTAAGGAAGGTTTATGGAAAAATTTACAATCTACACGGGGACAACGGTTCCTCTCATGAACGATAATATTGACACGGACCAAATTTTGCCCAAGCAGTTTCTCAAGTTGATTGATAAAAAAGGCTTTGGTAAGTACCTCATGTACGCTTGGCGCTATCTGGACAATCAGTACACCGAAGATCCTGATTTCGTCTTTAACAGACCGGAGTATCGCAAGGCGACTATTCTGATTACAGGGGACAATTTCGGAGCGGGTTCCTCTCGGGAGCACGCTGCCTGGGCCTTGGCTGACTATGGCTTTAAGGTAGTCATTGCCGGATCTTTCGGGGATATTCACTACAACAACGAGCTTAACAACGGTATGCTGCCTATCGTCCAGCCACTGGAAGTCCGGCAAGCATTGGCTAATCTGAAGCCGACGGATCAAGTGACGGTGGATTTGGAGCAGCAGAAGATTTTTTCGCCGGTGGGGGAATTCTCCTTTGACATTGATGGTGAATGGAAGCACAAGCTCCTCAACGGACTGGATGACATCGGCATTACGCTGCAGTATGAGGATTTGATTACAGAATATGAAAAAAATCGTCCATCTTATTGGCAGTAACCATATTTTTGCTATATTGAGAAAATTCTGAAAAAACCTTGTCTCCGATAAAAATATATGGTAAAATAAATCTTAATTTAATAGAAAAGGAAGAAACTTATGACAAAACACATTCAATGGAACGGACAGCTTTCACAAGAAGGTTATGACATCTTGAAAGGTGATGGTGGCTGTATCGTTTGCCCTACCAAGGTGGGTTACATCATCATGACTAGCGATAAAGCTGGCTTGGAGCGTAAGTTCGAAGCTAAGTCTCGTAACCGTAATAAGCCAGGTGTTGTCCTCTGTGGTAGTATGGATGAGCTCCGTGCTTTGGCTCAGCTGAATCCGGAAATTGAAGCTTTTTATCAAAAGCATTGGGATGAAGACATCCTCTTGGGCTGTATCCTACCATGGAAGCCAGAAGCTTTTGAAAAGCTCAAGGCATTTGGCGACGGTCGGGAAGAGCTGATGACAGATGTTCGTGGCACTAGCTGTTTTGTTATTAAGTTTGGTAAGGCGGGCGAGCAGCTGGCAGCTAAGCTTTGGGAAGAAGGCAAGATGGTTTATGCATCCTCAGCTAACCCATCTGGTAAAGGAAACCGCGGCAAGGTTGAAGGAATTGGCGAGCGCATCGAAAATGCTGTTGACCTCGTCATTGAAGCAGATGATTATGTAGCCTCTATCCAGCCAGACAAGACCATTGAAACTCGCTATGAGCAAGGCGTTATGGTCTCTATGGTGGACAAGGATGGAAAACTGATTCCAGAACAAGGTGGGCAACGTTCCATCTCACCAGCACCAGTGGTCATCCGCAAAGGCTTGGACATTGACAAAATCATGATGCACCTGTCCGATACTTTCAACTCATGGGATTACCGTCAGGGTGAATATTACTAA

>c183_g63

AAGAAAGATAATATTTTAATGCGAGAATTACTATCTAAAAAGAGCCACAGACAATTAGAACTATTAGAACTACTATTTAAAAACAAACGCTGGTTTCATATTTCTGAACTGGCTGAACTATTGAATTGTACAGAACGTTCAGTAAAAGATGATTTATCCCATGTCAAGTCTGCGTTTCCTCAATTGATTTTTCACTCGTCCACTAATGGTATACGTATCATTAATACCGATGATAGTGATATTGAGATGGTCTATCACCATTTTTTTAAACATTCAACCCATTTTTCAATTTTAGAATTCATCTTCTTTAACGAAGGATATGAAACTGAGAGTCTTTGTAAAGAGTTTTATATAAGCTCTTCCTCACTCTACCGTATAATCAGTCATATTAACAAAATTATAAAAAAACAATATAATTTCAAAATTAGTCTCAATCCTGCTCGGATTATTGGAGATGAGATTGATATCCGTTATTTTTTTGCACAATATTTTTCGGAAAAATATTATTTTCTTGAATGGCCCTTTACAGATTTTTCAGTAGAACCTTTGTGTAAGCTGTTAGCACTGGTCTATAAAGAAACTGCATTTCCTGTCAATTTCGCAACTCAAAGAATGTTAAAATTGCTCCTAGTTACAAATTTATATCGAATAAAGTTTGGTCATTTCTTGGAAGTTGAGAAAGATTCTTTTAACAATCAATTGTTAGAATCTTTCATGCAGGCAGAAGGAATCGAAGACATTGTAGCGAGCTTTGATTCTGAATATCATATCTCTTTGAATAAAGAAGTGATAGGCCAACTATTTGTCTCCTATTTTCAAAAAATGTTTTTCATTGATGAAAATCTATTTATGAGCTGCGCAAAAACAGACAGCTATGTAAAAAATTCGTATCAATTATTAAGTGATTTAATTGATCAGATAGAAAGCAAATATAATCTAAAAATAGACAATAATGACAATCTTATTTGGCATTTGCATAATACAGCACATCTGCACCGTCAGGAATTATCTACAGAGTTTATTCTGTTTGATCAAAAAGGAAATACAATCAAGAACTTTCAAAATATTTTCCCTCAATTTGTTTCAGATATTAAAAAAGGGATTGAATATTACCTAGAGACTTTGGATATCCATAGCACACCAATGAAAGTTAACCACCTATCCTATACTTTTATCACTCACAGCAAACATTTAGTACTGAATCTTTTACAAAATCAGCCTAAATTAAAAGTTTTAGTCATGAGTAATTTTGATCAGTATCATGCAAAATCAGTAGCAGAGACGCTTTCTTATTATTGCAGTAATAATTTTGAACTTGAAGTTTGGAATAAATTAGAACTATCAATTGATTCTTTAAAAGAATCACCTTACGATATCATCATTTCTAATTTTATTATTCCACCCATTGAGAACAAGAGACTGATCTATTCCAATAATATTAATACGGTCGCACTCATCTCCTTACTTAACGCAATGATGTTTATTCGCTTAGACGAGTGATTTAGACTATTTTGATAACAAAAAAAACCGCAATCTAGCGGTTTTTATTAATCTATGAGTGCCTTTTGAACATTAATTGTCTAACTTTGTGGGAGCAGTACATTATTTAGTACATATTCTTTCTCTTGTTTTTACAAAACTCTGAGAAATCCTATTCAAACAAGCCATAATAATCCGCGATGGTCATCTTGGCTTTCTCGTTTTTGTTTAAGTCCTGAATAATCTGCCCGTTTTTCATAACGATTAATCGGTTGCCGTATTTGAGGGCATCCTCCATGTGATGAGTAATCATAAGGGCTGTGAGGCGGTCACGATTGACAAATTCATTGGTCAGCTCCATCAAGGCAACACTGGTCTTAGGATCTAGCGCCGCAGTATGCTCATCCAGAAGCAAAAGCTCTGGGCGCTTGAGCGTTGCCATGAGCAGACTCAAAGCCTGTCTTTGCCCGCCAGAAAGAAATTCAATCGGCGTATCCAGATGTTTGTCCAGACCATTACCGATTTTCTCAATCGTCGCCTGAAACTCTTCCCTGTAACTGTTGAGCCCACGGGGCACCAAGCCACGCTTTTCACCTCGGAATTTGGCGATAAGAAGATTTTCCGCCACTGTCATACGCGGAGCCGTTCCCATCTTAGGATCCTGAAAGACACGGGACAGGTACTTGGCCCTCTTTTCTGGTGAAAAATGAGTGACATTTTCCCCCAAGATATGAATGCTGCCGCTGGTTAAGGGGAGAGTGCCGGCAATAACATTGAATAGGGTCGACTTTCCTGCACCGTTTCCACCTAAAATCGTAATAAAATCATGCTCGTGAATGTCTAAAGAAACATCATTTAGGATGATTTTTTCTTCATCAAAGCCGCTCTTTACAAGCTTGGTTGCATTTCTTAATTCTACAATCGCTGTCATTTGCTTAACTTGGCTCCTTTAAAGAATTTATTTTTCAGGGTTGGAATCATGAGACAGACGGCCAGAATCACCGCGCTGTAAAGACGGAGATAGTTGGTATTGAAGCCGAGAGCAATGACACCCCAGATCAAGAACTGATAGGCAATCGCACCAACTACAATGGTAATCAGCCGCTCTGCCAAGGTCAGACTCTGGAAGAGTACCTCGCCAATGATAAGACTGGCCAGACCAACTACGATTACTCCAATCCCGCGGGAAACATCTGCATAGCCTTCCTGCTGGGCAATCAGCGCTCCAGCCAGAGCAATCAAACCGTTTGATAGAATCAGACCCATCAACTCCATACGGCCGGTATTGATCCCAAAACTGCGAGCCATGTCTGGATTATCTCCAGTCGCGATATAGGCCTGCCCTAGCTTGGTATCCAAGAAAAAGAGCAGAGCTGCAATCACTAGACTGACAAAAATCAAACCAGTCAGCAGGTCATTGACTTCCCCTGAAAATGGCAGAACATCCTGAATCTTACTGCTGCCTAAAAGCCCCAGATTGGCACGGCCCATTAGCATCAGCATGATAGAGTGGCAGGAGGTCATGACCAAAATCCCAGAAAGCAGAGTCGGAATTTTCCCCTTGGTATAAAGCAGACCTGTCACGGCACCCGCAGCACAGCCCGCTAAAACGGCTGCCGCTGTTGCCAGAAAAGGATTGACTCCCCTGGTAATCAAGGTAACAGCTACCGCTCCGCCCAAGGGGAAGGAGCCTTCTGTCGTCATATCAGGAAAGCCCAGAATCCGGAAGGTCATAAAAATACCTAGACCTAGGACGGCCCAAACCAATCCCTGAGAAATAATGGAAAGAATCATAACATTGTCCTAACTAATTAAAATCGTTTCTCTATTTTAACATAAAAAAGGAGCCAATTTCAATGCTGATGCGCAGACAAAGAAATTGACTTCCCAACTATTCCTCAACAACTGTCATTCGACCAGTATCTACATCATAAACCGCACCTGAGATTTTTACATCAGCTGGGATCAGCGGCGACTGCCTCAGCAAGGCCATATCCTCTCGCACACTTTCCTCTACATCTGTAAAGGGCAAAAAGTCCTGATCGCTGACATCGACACCTAGCTCTTTATGAAGATAGGCCGTAAACTCCTCATTCTTAAAAGTCTGTGCCCCGCAGTCTGTATGATGCAAAACCACGATTTCCCGCGTTCCTAGCTGCTGCTGAGAGATGACCAGTGAGCGAATCATGTCCTCCGTCACCCGACCGCCGGCATTGCGCAAGATATGGGCGTCTCCTAGTGCCAGTCCAAGAGCCTGAGCCACGTGAAGCCTAGAGTCCATACAGGTCACAATAGCTACCTTGGTCTTGGGATTGATGGGCAGATGTGCTGTCCCATGCAAATCAACATAAGCTTTATTGGCCTTCATAAAGTTTTCAAAATATGACATTCTTTCCCCTTGCTAGTACTTTGACCTTCAAATAATTTTATATTATCTTACCATCTTTTCTGCTATTTGTC

>c183_g64

CTTATCTTAAATTTTCCTAAAAGTAACTTAAAAAAATGAAATTATTTTTTACAGCTCTTGCAAGAGGTACTGAAACAGTTCCAAATTGCCCTTTTCTTCGTTTATTAAAAACTCCGGATGCCATTGAAGGCCGATGATACGATGATTGTCAACCGATTCAATCGCTTCGATAGTATTATCTCTTGGATCAAAGGCTGTCGCTCGGAAATTAGGTGCCAAGTCCTTGATGCTTTGGCGATGAACCGAATTAATTTGGCTAGCCTGACCAAACAAGCGCTCCACAACGCTGTCTTTCTCCGTGCGAATGGAGTGCGAAGTACCAAATGGCAAGCCCTGCCAGTGATTTTCAATATGCTGATTAAGAGTACCGCCAAAAGCAACATTTACCAACTGCAGACCTCGGCAAATCGCCATGACTGGCTTATTTTGGCGCAGAGCTTCTTTCAAGAGTGCCAGCTCAAACTCATCGCGGACAATATTGTAATCATCACTGTCAATGGTCTTTTCTTCCCCATAAAACTGCGGATGAACATTTTGTCCGCCTGACAAAATCAGCTTGTCAATCGTTTCTACATAATCCTGAACTAAACTTTTATCCCCAACGGGAATCACCATCGGCAGGCCACCAACCATTCGTACGCCGTCCACAAACTTGCGTGAGACAGAGGTGTGAATATTCTTTCCAGCTTCATCTACTGGACAAAGATTGGCTGAAATTCCAACAATTGTTCTACACA

>c183_g65
[truncated: 1,209,892 more chars]
