# Supplementary material for: Transcriptome, Phenotypic, and Virulence Analysis of Streptococcus sanguinis SK36 Wild Type and Its CcpA-Null Derivative (ΔCcpA)
Source: Front Cell Infect Microbiol. 2019 Dec 4;9:411. doi: 10.3389/fcimb.2019.00411 (PMC6904348; doi:10.3389/fcimb.2019.00411)
Supplement: Supplementary file 3 [file Data_Sheet_3.pdf]

Supplementary file 3

Regulon of CcpA in Streptococcus sanguinis SK36

Properties

Regulator type:	Transcription factor
TF locus tag:	SSA_1576
Regulator family:	LacI
Regulation mode:	
Biological process:	Global catabolite repression
Effector:	Fructose-1,6-bisphosphate; HPr, phosphocarrier protein
Regulog:	CcpA - Streptococcaceae

Statistics of regulated genes:

- Genes	133
- Operons	66

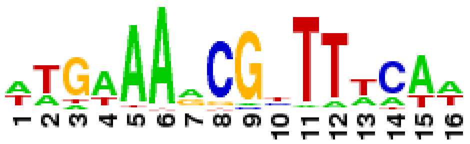

Built upon 968 sites [SEE MORE]

Member of regulog collections

- By taxonomy - Streptococcaceae
- By trascription factor - CcpA
- By TF family - LacI
- By effector - Fructose-1,6-bisphosphate
- By effector - HPr, phosphocarrier protein
- By pathway - Global catabolite repression

Regulated operons

Locus Tag	Name	Function
SSA_0282	celX	Cellobiose-specific PTS system, IIA component (EC 2.7.1.69)
SSA_0283	celY	Cellobiose-specific PTS system, cellobiose-specific IIB component (EC 2.7.1.69)
SSA_0284	celZ	Cellobiose-specific PTS system, IIC component (EC 2.7.1.69)
SSA_0572	idlD	L-iditol 2-dehydrogenase (EC 1.1.1.14)
SSA_0267	celQ	Predicted cellobiose repressor, ROK family
SSA_1960	SPy0278	Conserved hypothetical protein

Regulated operons

Locus Tag	Name	Function
SSA_1056	fbp	Fructose-1,6-bisphosphatase, Bacillus type (EC 3.1.3.11)
SSA_0052	manR	Predicted N-acetylgalactosamine utilization transcriptional regulator ManR, GntR family
↑SSA_2020	SUB1370	Hypothetical protein / zinc carboxypeptidase
↑SSA_0068	adhE	Alcohol dehydrogenase (EC 1.1.1.1); Acetaldehyde dehydrogenase (EC 1.2.1.10)
SSA_0456	scrA	Sucrose-specific PTS system, IIABC component (EC 2.7.1.69)
SSA_0457	scrK	Fructokinase (EC 2.7.1.4)
SSA_1826	glpK	Glycerol kinase (EC 2.7.1.30)
SSA_1827	glpD	Alpha-glycerophosphate oxidase (EC 1.1.3.21)
SSA_1828	glpF	Glycerol uptake facilitator protein
SSA_1577	pepQ	Xaa-Pro dipeptidase (EC 3.4.13.9)
↑SSA_1752	treP	Trehalose-specific PTS system, IIABC components (EC 2.7.1.69)
↑SSA_1751	treA	Trehalose-6-phosphate hydrolase (EC 3.2.1.93)
SSA_1178	acoA	Acetoin dehydrogenase E1 component alpha-subunit (EC 1.2.4.-)
SSA_1176	acoB	Acetoin dehydrogenase E1 component beta-subunit (EC 1.2.4.-)
↓SSA_0650	typA	GTP-binding protein TypA/BipA
↑SSA_1615		Alanine dehydrogenase (EC 1.4.1.1)
SSA_1137	pdhD	Dihydrolipoamide dehydrogenase of acetoin dehydrogenase (EC 1.8.1.4)
↑SSA_1138	pdhA	Acetoin dehydrogenase E1 component alpha-subunit (EC 1.2.4.-)
↑SSA_1139	pdhB	Acetoin dehydrogenase E1 component beta-subunit (EC 1.2.4.-)
↑SSA_1140	pdhC	Dihydrolipoamide acetyltransferase component (E2) of acetoin dehydrogenase complex (EC 2.3.1.-)
↑SSA_0736	arcR	Arginine catabolism (ADI pathway) transcriptional regulator ArcR, Crp-Fnr family
↓SSA_0847	pfk	6-phosphofructokinase (EC 2.7.1.11)
↓SSA_0848	pyk	Pyruvate kinase (EC 2.7.1.40)
↑SSA_1918	manL	PTS system, mannose-specific IIAB components
↑SSA_1919	manM	PTS system, mannose-specific IIC component (EC 2.7.1.69)
↑SSA_1920	manN	PTS system, mannose-specific IID component (EC 2.7.1.69)
↑SSA_1921	manO	Putative regulator of the mannose operon, ManO
↑SSA_0391	spxB	Pyruvate oxidase (EC 1.2.3.3)
↑SSA_1593	pepD2	Dipeptidase (EC 3.4.-.-)
↓SSA_1221	ldh	L-lactate dehydrogenase (EC 1.1.1.27)

Regulated operons

Locus Tag	Name	Function
↑SSA_1210	yjbM	GTP pyrophosphokinase (EC 2.7.6.5)
↑SSA_1209	ppnK	Predicted inorganic polyphosphate/ATP-NAD kinase
↑SSA_1208	yjbO	Similar to ribosomal large subunit pseudouridine synthase D, Bacillus subtilis YjbO type
SSA_1724	acuB	Acetoin utilization protein AcuB
SSA_0740	arcD	Arginine/ornithine antiporter
↑SSA_0741	arcT	Xaa-His dipeptidase (EC 3.4.13.3)
↑SSA_1809	ptsG	PTS system glucose-specific EIICBA component (EC 2.7.1.69)
↑SSA_1808	rgfB	Exodeoxyribonuclease III (EC 3.1.11.2)
↓SSA_1576	ccpA	Carbon catabolism global transcriptional regulator CcpA, LacI family
↑SSA_1753	treR	Trehalose utilization transcriptional regulator TreR, GntR family
↓SSA_0688	gpmA	2,3-bisphosphoglycerate-dependent phosphoglycerate (EC 5.4.2.1)
SSA_0782	atpE	ATP synthase C chain (EC 3.6.3.14)
SSA_0783	atpB	ATP synthase A chain (EC 3.6.3.14)
SSA_0784	atpF	ATP synthase B chain (EC 3.6.3.14)
SSA_0785	atpH	ATP synthase delta chain (EC 3.6.3.14)
SSA_0786	atpA	ATP synthase alpha chain (EC 3.6.3.14)
SSA_0787	atpG	ATP synthase gamma chain (EC 3.6.3.14)
SSA_0788	atpD	ATP synthase beta chain (EC 3.6.3.14)
SSA_0789	atpC	ATP synthase epsilon chain (EC 3.6.3.14)
SSA_0842	lrgA	Antiholin-like protein LrgA
SSA_0843	lrgB	LrgA-associated membrane protein LrgB
↑SSA_1298	malE	Maltose/maltodextrin ABC transporter, substrate-binding protein
↑SSA_1299	malF	Maltose/maltodextrin ABC transporter, permease protein 1
↑SSA_1300	malG	Maltose/maltodextrin ABC transporter, permease protein 2
SSA_0049	dhaK	Phosphoenolpyruvate-dihydroxyacetone phosphotransferase (EC 2.7.1.121), dihydroxyacetone binding subunit DhaK
SSA_0050	dhaL	Phosphoenolpyruvate-dihydroxyacetone phosphotransferase (EC 2.7.1.121), ADP-binding subunit DhaL
SSA_0051	dhaM	Phosphoenolpyruvate-dihydroxyacetone phosphotransferase (EC 2.7.1.121), subunit DhaM; DHA-specific IIA component
SSA_0053	bgaC	Beta-galactosidase (EC 3.2.1.23)
SSA_0054	agaB	Predicted galactosamine-specific PTS, EIIB component (EC 2.7.1.69)
SSA_0055	agaC	Predicted galactosamine-specific PTS, EIIC component (EC 2.7.1.69)

Regulated operons

Locus Tag	Name	Function
SSA_0056	agaD	Predicted galactosamine-specific PTS, EIID component (EC 2.7.1.69)
SSA_0057	agaE	Predicted galactosamine-specific PTS, EIIA component (EC 2.7.1.69)
SSA_1834	raiA	Ribosome-associated protein Y
SSA_1729	livJ	Branched-chain amino acid ABC transporter, substrate-binding protein
SSA_1728	livH	Branched-chain amino acid ABC transporter, permease protein 1
SSA_1727	livM	Branched-chain amino acid ABC transporter, permease protein 2
SSA_1726	livG	Branched-chain amino acid ABC transporter, ATP-binding protein 1
SSA_1725	livF	Branched-chain amino acid ABC transporter, ATP-binding protein 2
SSA_1204	pgm	Phosphoglucomutase (EC 5.4.2.2)
↑SSA_1917	adhB1	Alcohol dehydrogenase (EC 1.1.1.1)
↑SSA_0453	pulA	Pullulanase (EC 3.2.1.41)
SSA_0775	glgB	1,4-alpha-glucan (glycogen) branching enzyme, GH-13-type (EC 2.4.1.18)
↑SSA_0776	glgC	Glucose-1-phosphate adenylyltransferase (EC 2.7.7.27)
SSA_0777	glgD	Glucose-1-phosphate adenylyltransferase (EC 2.7.7.27)
↑SSA_0778	glgA	Glycogen synthase, ADP-glucose transglucosylase (EC 2.4.1.21)
SSA_1211	yjbK	Adenylate cyclase
SSA_0413	pabB	Para-aminobenzoate synthase component I (EC 4.1.3.-); Anthranilate/para-aminobenzoate synthases component I/ Chorismate binding enzyme, putative
SSA_1127	naoX	Predicted NADH oxidase
SSA_0772	ptsH	Phosphotransferase system, HPr-related protein
SSA_0773	ptsI	Phosphoenolpyruvate-protein kinase, PTS system component (EC 2.7.3.9)
SSA_2183	pgi	Glucose-6-phosphate isomerase (EC 5.3.1.9)
SSA_0219	fmpA	Predicted mannose specific PTS system, IIA component
SSA_0220	fmpB	Predicted mannose specific PTS system, IIB component
SSA_0221	fmpC	Predicted mannose specific PTS system, IIC component
SSA_0222	fmpD	Predicted mannose specific PTS system, IID component
SSA_0224	fmpE	Predicted mannose specific PTS system, putative EII component
SSA_0455	scrB	Sucrose-6-phosphate hydrolase (EC 3.2.1.26)
SSA_0454	scrR	Sucrose operon repressor ScrR, LacI family
↑SSA_1749	pflA	Pyruvate formate-lyase activating enzyme (EC 1.97.1.4)
SSA_1001	msmR	MSM (multiple sugar metabolism) operon regulatory protein
↑SSA_1949	hppG	Oligopeptide ABC transporter, periplasmic oligopeptide-binding protein OppA (TC 3.A.1.5.1)

Regulated operons

Locus Tag	Name	Function
SSA_1002	agaL	Alpha-galactosidase (EC 3.2.1.22)
SSA_1003	msmE	Multiple sugar ABC transporter, substrate-binding protein
SSA_1004	msmF	Multiple sugar ABC transporter, permease protein 2
SSA_1005	msmG	Multiple sugar ABC transporter, permease protein 1
SSA_0243		2',3'-cyclic-nucleotide 2'-phosphodiesterase (EC 3.1.4.16)
SSA_1008	galK	Galactokinase (EC 2.7.1.6)
SSA_1009	galT	Galactose-1-phosphate uridylyltransferase (EC 2.7.7.10)
SSA_0071	nanE	N-acetylmannosamine-6-phosphate 2-epimerase (EC 5.1.3.9)
SSA_0072	msmK	Multiple sugar ABC transporter, ATP-binding protein
SSA_0073	yjgK	Conserved hypothetical protein
SSA_0074	nanU	N-acetylneuraminate ABC transporter, substrate-binding protein
SSA_0075	nanV	N-acetylneuraminate ABC transporter, permease protein 1
SSA_0076	nanW	N-acetylneuraminate ABC transporter, permease protein 2
SSA_0077	yjhC	Predicted oxidoreductase (EC 1.1.1.-)
SSA_0078	nanA	N-acetylneuraminate lyase (EC 4.1.3.3)
SSA_0079	nanK	N-acetylmannosamine kinase (EC 2.7.1.60)
SSA_0081	nanR	Sialic acid utilization transcriptional regulator NanR, RpiR family
SSA_2093	sgaT	Ascorbate-specific PTS system, IIC component
SSA_2092	sgaB	Ascorbate-specific PTS system, IIB component (EC 2.7.1.69)
SSA_2091	sgaC	Ascorbate-specific PTS system, IIA component (EC 2.7.1.-)
SSA_2090	sgaH	3-keto-L-gulonate 6-phosphate decarboxylase (EC 4.1.1.85)
SSA_2089	sgaU	L-xylulose 5-phosphate 3-epimerase (EC 5.1.3.-)
SSA_2088	sgaE	L-ribulose-5-phosphate 4-epimerase (EC 5.1.3.4)
SSA_0395	ascB	6-phospho-beta-glucosidase (EC 3.2.1.86)
SSA_0886	eno	Enolase (EC 4.2.1.11)
SSA_0885	yuel	Conserved hypothetical protein
SSA_1065	hex	Beta-hexosaminidase
SSA_1098	yrhG	Predicted permease
SSA_0192	ackA	Acetate kinase (EC 2.7.2.1)
SSA_2040	msmK	Multiple sugar ABC transporter, ATP-binding protein
SSA_1261	rpiA	Ribose 5-phosphate isomerase A (EC 5.3.1.6)
SSA_1260	deoB	Phosphopentomutase (EC 5.4.2.7)
SSA_1259	punA	Purine nucleoside phosphorylase (EC 2.4.2.1)

Regulated operons

Locus Tag	Name	Function
↑ SSA_0737	arcA	Arginine deiminase (EC 3.5.3.6)
↑ SSA_0738	arcB	Ornithine carbamoyltransferase (EC 2.1.3.3)
SSA_1033	coaA	Pantothenate kinase (EC 2.7.1.33)

Rectangle reprenst an operon

The highlight represent the genes that were in the list of unigene but not among the genes found to be differentially expressed in a CcpA mutant

Arrows reprenst the genes that can be matched to the differentially expressed in a CcpA mutant
